# IPX203 vs Immediate-Release Carbidopa-Levodopa for the Treatment of Motor Fluctuations in Parkinson Disease: The RISE-PD Randomized Clinical Trial

**DOI:** 10.1001/jamaneurol.2023.2679

**Published:** 2023-08-14

**Authors:** Robert A. Hauser, Alberto J. Espay, Aaron L. Ellenbogen, Hubert H. Fernandez, Stuart H. Isaacson, Peter A. LeWitt, William G. Ondo, Rajesh Pahwa, Johannes Schwarz, Fabrizio Stocchi, Leonid Zeitlin, Ghazal Banisadr, Stanley Fisher, Hester Visser, Richard D’Souza

**Affiliations:** 1 University of South Florida Parkinson’s Disease and Movement Disorders Center/Parkinson Foundation Center of Excellence, Tampa; 2 James J. and Joan A. Gardner Center for Parkinson’s Disease and Movement Disorders, University of Cincinnati, Cincinnati, Ohio; 3 Quest Research Institute/Michigan Institute for Neurological Disorders, Farmington Hills; 4 Center for Neurological Restoration, Neurological Institute, Cleveland Clinic, Cleveland, Ohio; 5 Parkinson’s Disease and Movement Disorders Center of Boca Raton, Boca Raton, Florida; 6 Wayne State University School of Medicine and Henry Ford Hospital, Detroit, Michigan; 7 Methodist Hospital and Weill Cornell Medical School, Houston, Texas; 8 University of Kansas Medical Center, Kansas City; 9 Geriatric Hospital Haag and Technical University of Munich, Munich, Germany; 10 Istituto di Ricovero e Cura a Carattere Scientifico San Raffaele Pisana, Department of Neurology, Roma, Italy; 11 Quartesian, Princeton, New Jersey; 12 Amneal Pharmaceuticals, Bridgewater, New Jersey

## Abstract

**Importance:**

Levodopa has a short half-life and a limited window of opportunity for absorption in the proximal small intestine. IPX203 is an oral, extended-release formulation of carbidopa-levodopa developed to address these limitations.

**Objective:**

To assess the efficacy and safety of IPX203 vs immediate-release carbidopa-levodopa in patients with Parkinson disease who are experiencing motor fluctuations.

**Design, Setting, and Participants:**

RISE-PD was a 20-week, randomized, double-blind, double-dummy, active-controlled, phase 3 clinical trial. The study was conducted between November 6, 2018, and June 15, 2021, at 105 academic and clinical centers in the US and Europe. Patients with Parkinson disease taking a total daily dose of 400 mg or more of levodopa and experiencing an average of 2.5 hours or more daily off-time were included in the study. A total of 770 patients were screened, 140 were excluded (those taking controlled-release carbidopa-levodopa apart from a single daily bedtime dose, Rytary (Amneal Pharmaceuticals), additional carbidopa or benserazide, or catechol O-methyl transferase inhibitors or who had a history of psychosis within the past 10 years), and 630 were enrolled in the trial.

**Interventions:**

Following open-label immediate-release carbidopa-levodopa dose adjustment (3 weeks) and conversion to IPX203 (4 weeks), patients were randomized in a 1:1 ratio to double-blind, double-dummy treatment with immediate-release carbidopa-levodopa or IPX203 for 13 weeks.

**Main Outcome and Measures:**

The primary end point was mean change in daily good on-time (ie, on-time without troublesome dyskinesia) from baseline to the end of the double-blind treatment period.

**Results:**

A total of 630 patients (mean [SD] age, 66.5 [8.95] years; 396 [62.9%] men) were enrolled, and 506 patients were randomly assigned to receive IPX203 (n = 256) or immediate-release carbidopa-levodopa (n = 250). The study met its primary end point, demonstrating statistically significant improvement in daily good on-time for IPX203 compared to immediate-release carbidopa-levodopa (least squares mean, 0.53 hours; 95% CI, 0.09-0.97; *P* = .02), with IPX203 dosed a mean 3 times per day vs 5 times per day for immediate-release carbidopa-levodopa. Good on-time per dose increased by 1.55 hours with IPX203 compared to immediate-release carbidopa-levodopa (95% CI, 1.37-1.73; *P* < .001). IPX203 was well tolerated. The most common adverse events in the double-blind phase (IPX203 vs immediate-release carbidopa-levodopa) were nausea (4.3% vs 0.8%) and anxiety (2.7% vs 0.0%).

**Conclusions and Relevance:**

In this study, IPX203 provided more hours of good on-time per day than immediate-release carbidopa-levodopa, even as IPX203 was dosed less frequently.

**Trial Registration:**

ClinicalTrials.gov Identifier: NCT03670953

## Key Points

**Question:**

Can extended-release carbidopa-levodopa (IPX203) improve symptomatic control in patients with Parkinson disease who are experiencing motor fluctuations while taking immediate-release carbidopa-levodopa?

**Findings:**

In this phase 3 randomized clinical trial involving 506 participants, IPX203 showed statistically significant improvement in daily good on-time compared to immediate-release carbidopa-levodopa when dosed a mean of 3 times per day compared to 5 times per day for immediate-release carbidopa-levodopa.

**Meaning:**

The results of this study suggest that IPX203 vs immediate-release carbidopa-levodopa may be useful in patients with Parkinson disease and motor fluctuations by providing more sustained benefit throughout the day, even with fewer daily doses.

## Teaser

This randomized clinical trial evaluates the safety and efficacy of extended-release carbidopa-levodopa (IPX203) compared with immediate-release carbidopa-levodopa in patients with Parkinson disease.

## Introduction

Levodopa (LD) is the most effective oral therapy for the symptomatic treatment of Parkinson disease (PD), but its use is complicated by the development of motor complications.^1,2,3,4,5^ While the causes of motor complications are not fully understood, they are thought to arise from the short plasma elimination half-life of immediate-release (IR) LD, variable absorption due to gastrointestinal dysmotility, and changes within striatal brain mechanisms.^6,7^ Well-tolerated oral therapies that provide symptom relief quickly and maintain that benefit for many hours are needed.

IPX203, a new oral extended-release carbidopa-levodopa (CD-LD) capsule, was developed to address the short plasma half-life and limited absorption window for LD.^8,9^ IPX203 contains IR granules and extended-release coated beads.^10^ The IR granules consist of CD and LD for rapid dissolution. The extended-release beads consist of LD, coated with a sustained-release polymer to enable slow release of the drug, a mucoadhesive polymer to keep the beads adhered to the area of absorption longer, and an enteric polymer to prevent the beads from disintegrating too early in the stomach. Pharmacokinetic analyses confirmed a rapid increase in LD plasma level that was then sustained longer than current formulations of oral CD-LD.^9^ The Randomized Controlled Study to Compare the Safety and Efficacy of IPX203 With Immediate-Release Carbidopa-Levodopa in Parkinson’s Disease Patients With Motor Fluctuations (RISE-PD) aimed to assess the efficacy and safety of IPX203 compared with IR CD-LD in PD patients experiencing motor fluctuations.

## Methods

**Figure 1.**
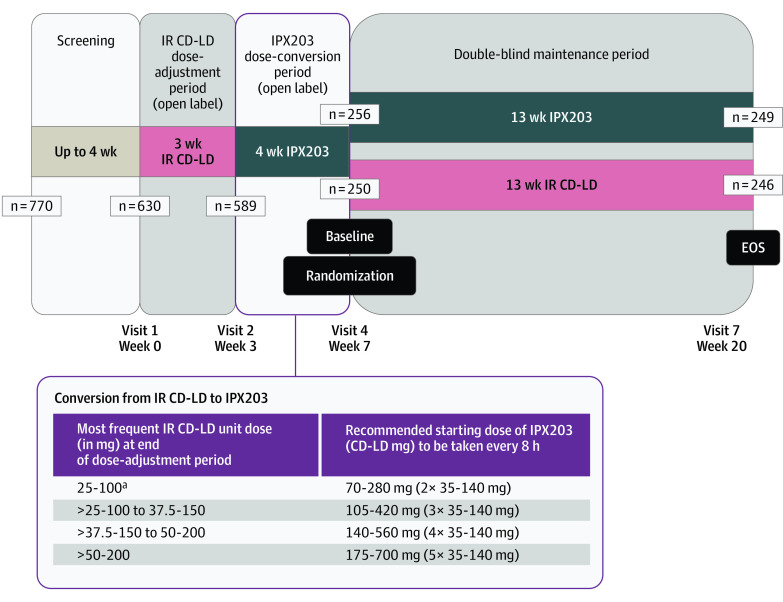
Study Design EOS indicates end of study; IR CD-LD, immediate-release carbidopa-levodopa. ^a^IPX203 was recommended to be dosed approximately every 8 hours. Participants who were taking a total daily dose of less than 125-500 mg CD-LD from IR CD-LD were advised to initially take IPX203 every 12 hours. The dosing interval could vary but could not be more frequent than every 6 hours.

RISE-PD was a 20-week, randomized, double-blind, double-dummy, active-controlled, parallel group, phase 3 trial conducted at 105 academic and clinical centers in the US and Europe. The trial protocol can be found in Supplement 1, and the trial design is shown in Figure 1. All participants provided written informed consent and all sites obtained approval from their appropriate institutional review board. The study was conducted in compliance with International Council for Harmonization (ICH) of Technical Requirements for Pharmaceuticals for Human Use harmonized tripartite guideline E6 (R2) and good clinical practice guidelines.^11^ Patients were screened between November 6, 2018, and June 15, 2021.

### Participants

Key inclusion criteria included a diagnosis of PD fulfilling the UK Parkinson disease Brain Bank Criteria, age 40 years or older at time of diagnosis, Hoehn-Yahr stage I to IV in the on-state, Montreal Cognitive Assessment score 24 or greater, treatment with a stable regimen of CD-LD for 4 weeks or longer before screening, a total daily LD dose 400 to 2400 mg, and a daily dosing frequency of 4 to 9 times per day, with a 3-day average of 2.5 hours or more off-time per day from PD diaries^12^ at screening and study entry. Concomitant therapy with dopamine agonists, monoamine oxidase type B inhibitors, amantadine, and anticholinergic drugs at stable doses was permitted.

Key exclusion criteria included atypical or secondary parkinsonism, previous neurosurgical treatment for PD, lack of response to LD, controlled-release CD-LD apart from a single daily bedtime dose, extended-release CD-LD (Rytary [Amneal Pharmaceuticals]), additional CD or benserazide, catechol O-methyl transferase inhibitors, or a history of psychosis within the past 10 years.

### Study Design

The study consisted of a screening period lasting up to 4 weeks, a 3-week open-label IR CD-LD dose-adjustment period, a 4-week open-label IPX203 dose-conversion period, and a 13-week double-blind, double-dummy treatment period (Figure 1). Following the end of the screening period (visit 1), patients entered the open-label IR CD-LD dose-adjustment period (weeks 1-3) during which investigators adjusted the dose and frequency of IR CD-LD as necessary to achieve optimum balance of efficacy and tolerability. IR CD-LD tablets could be split. For those receiving a single daily bedtime dose of CR CD-LD at study entry, the CR CD-LD dose was discontinued and initially substituted with a 1:1 mg-equivalent dose of IR CD-LD. Doses and regimens of other concomitant PD medications had to remain stable throughout the study. The IR CD-LD dosing regimen had to be stable for at least 5 days prior to returning for visit 2.

The IR CD-LD dose-adjustment period was followed by the open-label, IPX203 dose-conversion period (weeks 3-7). The initial dosing regimen of IPX203 was based on the most frequent unit dose (mg) of IR CD-LD taken at the end of the IR CD-LD dose-adjustment period (visit 2). As shown in Figure 1, if the most frequent unit dose per day of IR CD-LD was 25-100 mg, the patient was converted to a starting daily dosing regimen of IPX203, 70-280 mg, 3 times daily. It was recommended that IPX203 initially be dosed approximately every 8 hours. This dosing interval could be shortened but IPX203 could not be administered more frequently than every 6 hours. Patients on a total daily dose of less than 125-500–mg IR CD-LD at the end of the IR CD-LD dose-adjustment period were advised to initially take IPX203 every 12 hours. All patients were required to be on a stable dosing regimen of IPX203 at the end of the dose-conversion period and for at least 5 days prior to returning for visit 4 (randomization visit), which was considered the baseline visit.

Following the dose-conversion period, patients were randomized in a 1:1 ratio in the double-blind double-dummy maintenance period (weeks 7-20) to their dosing regimen established at the end of week 3 (visit 2) for IR CD-LD, and at the end of week 7 (visit 4) for IPX203, plus placebo for the other, using a double-dummy schema. Patients and site personnel were blinded to study drug assignment during the double-blind treatment period. No dosing regimen adjustments could be made during this period. Patients returned to the clinic for 3 visits (visits 5, 6, and 7) (Figure 1) and completed 3-day PD diaries on each of 3 consecutive days immediately prior to each of these visits.

### Study Medication

IPX203 capsules containing 35-mg CD and 140-mg LD (CD-LD ratio was 1:4) and matching placebo capsules were manufactured by Impax Laboratories. IR CD-LD tablets (25-100) were procured commercially and repackaged by PCI Pharma Services. Impax Laboratories manufactured matching placebos.

### Outcomes

The primary efficacy end point was the mean change from baseline in good on-time in hours per day, averaged over the 3 PD diary days,^12^ to the end of double-blind therapy (visit 7, end-of-study visit, or early termination). Good on-time was defined as the sum of on-time without dyskinesia and on-time with nontroublesome dyskinesia, equivalent to on-time without troublesome dyskinesia. The first secondary end point was the mean change from baseline in off-time in hours per day, averaged over the 3 PD diary days, to the end of double-blind therapy.

Other secondary end points were the proportion of patients’ self-ratings of much improved or very much improved on the Patient Global Impression of Change (PGI-C) scale, change from baseline in the Movement Disorders Society Version of the Unified Parkinson Disease Rating Scale (MDS-UPDRS) Part III, and change from baseline in the sum of MDS-UPDRS Parts II and III at the end of the double-blind treatment period.

Safety end points evaluated throughout the study included adverse events (AEs), clinical laboratory tests, vital signs (including supine and standing blood pressure and heart rate), physical examinations, electrocardiograms, and Columbia-Suicide Severity Rating Scale scores.

### Statistical Analysis

A sample size of approximately 210 per treatment arm was required to ensure at least 90% power at a .05 significance level. All statistical evaluations were conducted using SAS version 9.4 (SAS Institute). The intent-to-treat analysis set included all patients who were randomized and treated with any study drug and had at least 1 postbaseline efficacy assessment. The modified intent-to-treat analysis set included all patients who were randomized and treated, had a valid baseline PD diary, and had at least 1 valid postrandomization PD diary. The safety analysis set included all patients who were treated with any study drug (IPX203 or IR CD-LD).

The primary and secondary end points were tested in a single hierarchical order: (1) change from baseline in good on-time, (2) change from baseline in off-time, (3) proportion of patients reporting either much improved or very much improved on PGI-C, (4) change from baseline in MDS-UPDRS Part III, and (5) change from baseline in the sum of MDS-UPDRS Parts II and III.

A post hoc analysis was performed to evaluate the difference between IPX203 and IR CD-LD for the average duration of efficacy (ie, good on-time) in hours per dose. Least squares (LS) mean good on-time per dose at visit 7 or early termination for the modified intent-to-treat population for IPX203 and IR CD-LD treatment groups were calculated using a mixed model for repeated measures. Good on-time per dose was defined as daily good on-time (in hours) divided by the daily dosing frequency during the stable dosing regimen.

A retrospective analysis of pill burden was performed. For operational feasibility, IR CD-LD tablets were limited to 25-100 CD-LD and IPX203 capsules were limited to 35-140 CD-LD. The analysis considered each IPX203 capsule and each IR CD-LD tablet, or partial tablet, as 1 pill.

## Results

**Figure 2.**
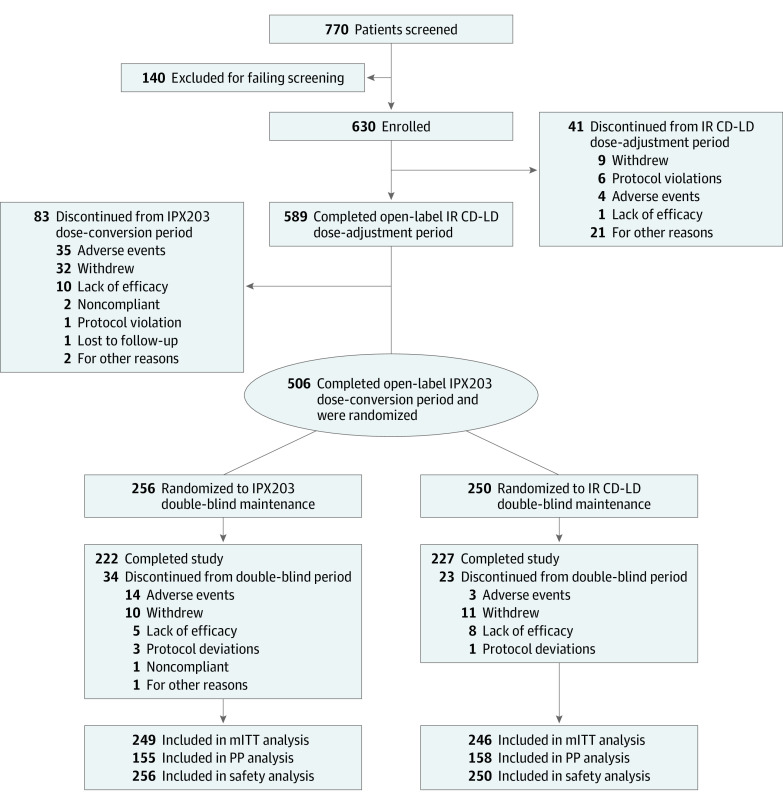
CONSORT Diagram IR CD-LD indicates immediate-release carbidopa-levodopa; mITT, modified intent-to-treat; PP, per-protocol.

Between November 6, 2018, and June 15, 2021, 770 patients were screened, and 630 (mean [SD] age, 66.5 [8.95] years; 396 men [62.9%] and 234 women [37.1%]) entered the IR CD-LD dose-adjustment period (Figure 2). Of these, 589 (93.5%) entered the IPX203 conversion period, 506 (80.3%) completed the open-label conversion and entered the randomized treatment period, and 449 (88.7%) completed the study. The primary reasons for discontinuation were withdrawal by patient, AEs, and lack of efficacy (Figure 2). At study entry, the mean (SD) age of PD onset was 58.0 (9.42) years (eTable 1 in Supplement 2).

**Figure 3.**
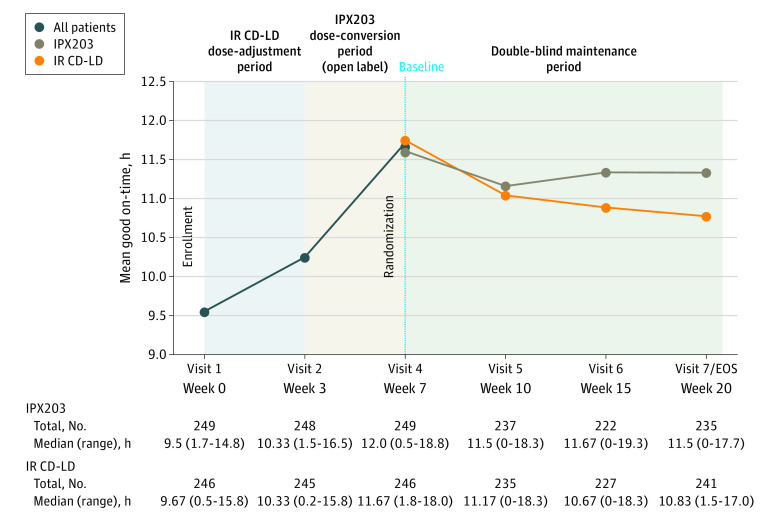
Change From Baseline in Good On-Time, Least Squares Mean Good on-time was defined as on-time without troublesome dyskinesia. EOS indicates end of study; IR CD-LD, immediate-release carbidopa-levodopa.

The study met its primary end point, demonstrating statistically significant improvement in good on-time in hours per day for IPX203 compared with IR CD-LD, even as IPX203 was dosed on average 3 times per day and IR CD-LD was dosed on average 5 times per day (LS mean change for IPX203, −0.50; LS mean change for IR CD-LD, −1.03; difference in LS means, 0.53; 95% CI, 0.09-0.97; *P* = .02) (Figure 3).

The secondary end point of change from baseline in off-time in hours per day showed that IPX203 treatment resulted in significantly less off-time compared with IR CD-LD (difference in LS means, −0.48; 95% CI, −0.90 to −0.06; *P* = .03) (eFigure and eTable 2 in Supplement 2). Analysis of the next secondary end point, PGI-C scores, showed that 76 of 256 patients treated with IPX203 (29.7%) rated themselves much improved or very much improved compared with 47 of 250 patients treated with IR CD-LD (18.8%) (*P* = .002) (eTable 2 in Supplement 2). There were no differences between groups in change from baseline to visit 7 or early termination in MDS-UPDRS Part III scores or in the sum of MDS-UPDRS Parts II and III scores (eTable 2 in Supplement 2).

During the IR CD-LD dose-adjustment period, 118 patients (18.7%) experienced treatment-emergent AEs (TEAEs), 31 of whom (4.9%) experienced treatment-related TEAEs. Six patients (1%) experienced TEAEs that led to discontinuation of the study drug, and 7 (1.1%) experienced serious TEAEs. The most frequently reported TEAEs were dyskinesia (11 [1.7%]) and headache (8 [1.3%]) (Table).

**Table.** Safety Summary

Category	No. (%)	
	IR CD-LD dose-adjustment period (n = 630)	IPX203 dose-conversion period (n = 589)
Total patients in the open-label period, No.	630	589
Patients with TEAEs	118 (18.7)	229 (38.9)
Patients with treatment-related TEAEs	31 (4.9)	140 (23.8)
Patients with TEAEs leading to study drug discontinuation	6 (1.0)	42 (7.1)
Patients with serious TEAEs	7 (1.1)	12 (2.0)
Most common TEAEs (≥1% in either treatment group)		
Dyskinesia	11 (1.7)	40 (6.8)
Nausea	7 (1.1)	29 (4.9)
Dry mouth	2 (0.3)	25 (4.2)
Dizziness	3 (0.5)	17 (2.9)
Fall	6 (1.0)	13 (2.2)
Insomnia	6 (1.0)	13 (2.2)
Vomiting	3 (0.5)	13 (2.2)
Constipation	7 (1.1)	12 (2.0)
Headache	8 (1.3)	9 (1.5)
Hypertension	7 (1.1)	4 (0.7)
Total patients in the double-blind maintenance period, No.	250	256
Patients with TEAEs	79 (31.6)	108 (42.2)
Patients with treatment-related TEAEs	17 (6.8)	42 (16.4)
Patients with TEAE leading to study drug discontinuation	3 (1.2)	14 (5.5)
Patients with serious TEAE	4 (1.6)	8 (3.1)
Most common TEAEs (≥2% in either treatment group)		
Nausea	2 (0.8)	11 (4.3)
Anxiety	0	7 (2.7)
Dizziness	2 (0.8)	6 (2.3)
Fall	9 (3.6)	5 (2.0)
Dyskinesia	1 (0.4)	5 (2.0)
Urinary tract infection	8 (3.2)	4 (1.6)

Abbreviations: IR CD-LD, immediate-release carbidopa-levodopa; TEAEs, treatment-emergent adverse events.

During the IPX203 dose-conversion period, 229 patients (38.9%) reported TEAEs, 140 of whom (23.8%) experienced treatment-related TEAEs. Forty-two patients (7.1%) experienced TEAEs that led to discontinuation of the study drug, and 12 (2%) experienced serious TEAEs. The most frequently reported TEAEs were dyskinesia (40 [6.8%]), nausea (29 [4.9%]), and dry mouth (25 [4.2%]) (Table).

During the double-blind period, 108 patients treated with IPX203 (42.2%) experienced TEAEs compared with 79 patients who received IR CD-LD (31.6%). Forty-two patients who received IPX203 (16.4%) and 17 who received IR CD-LD (6.8%) experienced treatment-related TEAEs. Fourteen patients receiving IPX203 (5.5%) and 3 receiving IR CD-LD (1.2%) experienced TEAEs that led to discontinuation of the study drug. Eight patients treated with IPX203 (3.1%) and 4 treated with IR CD-LD (1.6%) experienced serious TEAEs. As shown in the Table, the most frequently reported TEAEs among patients treated with IPX203 were nausea (11 [4.3%]), anxiety (7 [2.7%]), and dizziness (6 [2.3%]). The most frequently reported TEAEs among patients treated with IR CD-LD were fall (9 [3.6%]), urinary tract infection (8 [3.2%]), and back pain (7 [2.8%]). No clinically relevant treatment group differences were noted for laboratory parameters, vital signs, or electrocardiogram results. There were no deaths reported.

**Figure 4.**
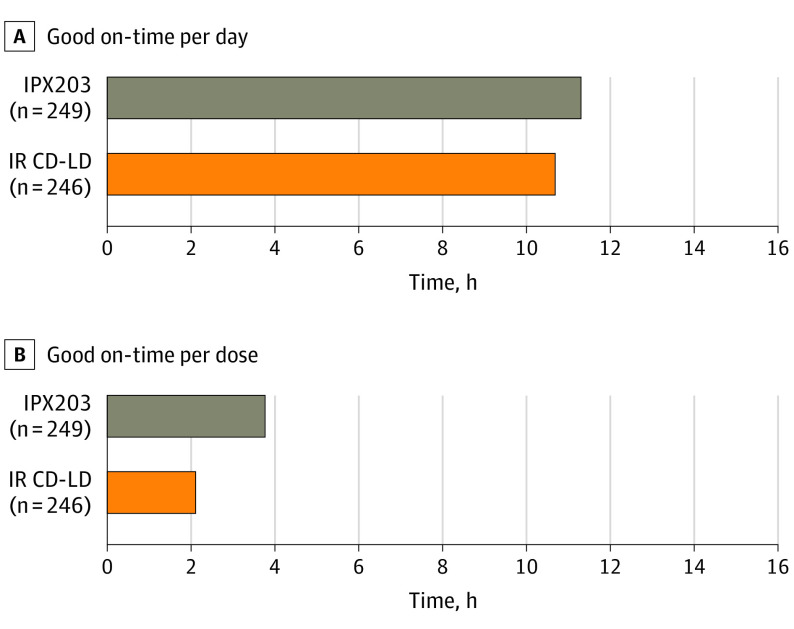
Good On-Time at Visit 7/Early Termination Good on-time was defined as on-time without troublesome dyskinesia. IR CD-LD indicates immediate-release carbidopa-levodopa.

Figure 4 depicts mean daily good on-time and good on-time per dose at visit 7, early termination, or end of study. Patients receiving IPX203 experienced a longer duration of good on-time per dose (LS mean, 3.76 hours; 95% CI, 3.62-3.91) vs those receiving IR CD-LD (LS mean, 2.21 hours; 95% CI, 2.07-2.36). IPX203 treatment increased good on-time per dose compared to IR CD-LD by a mean of 1.55 hours (difference in LS means, 1.55; 95% CI, 1.37-1.73; *P* < .001).

Details of doses and dosing frequencies of LD are summarized in eTable 3 in Supplement 2. Mean (SD) dosing frequency was 3.04 (0.40) hours for IPX203 vs 5.01 (1.19) hours for IR CD-LD. Pill burden analysis showed that in this clinical trial, mean (SD) daily number of pills was 10.6 (4.2) for IPX203 and 8.7 (3.8) for IR CD-LD.

## Discussion

In this phase 3 randomized clinical trial, IPX203 demonstrated superior efficacy on the primary and multiple secondary end points compared with IR CD-LD, even as IPX203 was dosed a mean of 3.04 times per day vs 5.01 times per day for IR CD-LD. IPX203 treatment resulted in 0.53 more hours of good on-time per day than IR CD-LD, comparing change from baseline to end of study. Good on-time has been shown to be associated with patients’ perceived duration of a good response throughout the day and provides an index of patients’ motor status that reflects the effects of both parkinsonism and dyskinesia.^12^ Thus, good on-time is considered an appropriate outcome measure in clinical trials of patients with PD who are experiencing motor complications. Though the frequency of pill administration was reduced in this study, the number of daily pills administered was higher at mean (SD) 10.6 (4.2) for the IPX203 group compared to 8.7 (3.8) for the IR CD-LD group. In this double-dummy clinical trial, study medications were limited to IPX203, 35-140 capsules, and IR CD-LD, 25-100 tablets, for operational feasibility. The New Drug Application for IPX203 includes 4 dose strengths (ie, 35-140 mg, 52.5-210 mg, 70-280 mg, and 87.5-350 mg) that should allow for a substantially reduced number of IPX203 pills per day for a similar population in clinical practice.

The beneficial effects of IPX203 appear clinically meaningful, as suggested by PGI-C measures showing that a significantly higher number of patients treated with IPX203 rated themselves much improved or very much improved compared with patients treated with IR CD-LD. These results are consistent with the notion that maintenance of LD plasma concentration translates into more sustained clinical benefit in patients with motor fluctuations. The study did not show a difference in MDS-UPDRS scores across the 2 treatment groups. This observation is not unexpected, as motor examinations were conducted when patients were in their best on-state and Part II scores query patients about their usual difficulty with motor aspects of experiences of daily living over the prior week, including both on- and off-periods.

A key metric to assess the benefit of a long-acting formulation is the mean duration of efficacy per dose. Post hoc analysis showed that IPX203 increased good on-time per dose by 1.55 hours more than IR CD-LD, representing a 70% increase. This provides additional important evidence of clinical efficacy and may help guide clinicians in making medication-management decisions. In addition, studies have shown that taking fewer doses per day is associated with greater compliance.^13^

Various pharmacological strategies have been developed to reduce variability of serum LD concentrations to mitigate motor fluctuations. One strategy is to administer a continuous enteral infusion of CD-LD. Pharmacokinetic-pharmacodynamic studies have shown that this approach is associated with reduced daily off-time and dyskinesia compared with IR CD-LD, but this delivery method is invasive and cumbersome and therefore impractical for many patients.^14^ Extended-release CD-LD (Rytary) was designed to provide initial rapid absorption of LD, followed by stable LD plasma concentration with reduced peak-to-trough fluctuations. Phase 3 studies of Rytary in patients with PD showed a significant reduction in daily off-time compared to both IR CD-LD and IR CD-LD plus entacapone.^15,16^

To our knowledge, there are no randomized blinded trials comparing Rytary and IPX203. Results from a single-dose, open-label study in patients with PD with motor fluctuations indicated that plasma LD concentrations were sustained longer with IPX203 than with Rytary.^9^ In addition, IPX203 provided a longer duration of clinical benefit.^9^ Analysis of good on-time per dose, derived from separate phase 3 randomized double-blind trials, showed an increase of 1.16 hours for Rytary compared to IR CD-LD,^17^ vs an increase of 1.55 hours for IPX203 compared to IR CD-LD.

In RISE-PD, IPX203 was usually administered every 8 hours and could be adjusted to every 6 hours. This dosing scheme was developed based on dose conversion data obtained in 2 phase 2 studies.^8,9^ However, in our study, IPX203 dosing a mean of 3 times per day still left patients with a mean 4.18 hours off-time per day. This raises the question as to whether off-time could have been further reduced, and good on-time further increased, with more frequent IPX203 dosing.

IPX203 was generally well tolerated and showed an acceptable safety profile. Most of the AEs reported in the dose-conversion period were expected with adjustment of dopaminergic therapy. In the randomized double-blind period, TEAEs were reported for both treatment groups with a higher frequency in the IPX203 group (42.2% of patients for IPX203 and 31.6% of patients for IR CD-LD), although all TEAEs occurred in a small percentage of patients. The reason for the higher percentages of TEAEs in the IPX203 treatment group could be related to the dosing frequency restrictions for IPX203. If the dosing frequency was not adequate for a patient, individual doses could be increased, and this could potentially result in more dopaminergic side effects. Anxiety was numerically more common in the double-blind maintenance period in the IPX203 group vs the IR CD-LD group. As anxiety is a common nonmotor wearing-off symptom, the higher incidence observed in the IPX203 group might be due to infrequent dosing and possible gradual wearing off.

### Limitations

This study has several limitations. The trial compared IPX203 to IR CD-LD in patients experiencing motor fluctuations while taking IR CD-LD. It did not evaluate IPX203 in patients with early PD vs placebo or as a means to potentially delay motor fluctuations and dyskinesia. Additional limitations include the fact that only 25-100 IR CD-LD tablets and 35-140 IPX203 capsules were used in the study. This allowed operational feasibility but increased overall pill burden compared to being able to use additional dosage formulations. Another limitation of the study design was the prohibition of administration of IPX203 more frequently than every 6 hours. Some patients may have experienced a greater increase in good on-time and reduction in off-time while receiving IPX203 if it could have been administered more frequently than every 6 hours and if dosing frequency could have been titrated to clinical response.

## Conclusions

In this phase 3 study, IPX203 resulted in significantly increased daily good on-time, even as IPX203 was administered 3 times per day and IR CD-LD was administered 5 times per day. These results suggest that IPX203 may be useful in patients with motor fluctuations to provide more sustained benefit throughout the day. IPX203 dose conversion based on the most frequent unit dose of IR CD-LD may represent a relatively easy dose-conversion strategy for use in clinical practice.

## Notes

Supplement 1. Trial protocol

Supplement 2. eTable 1. Demographics and Study Entry Characteristics eTable 2. Data Summary of Efficacy Endpoints eTable 3. Levodopa Dosages and Dosing Frequencies in the Double-Blind Period, Randomized Analysis Set eFigure. Change from Baseline in “Off” Time, mITT Analysis Set

Supplement 3. Data sharing statement
