# Supplementary material for: IPX203 vs Immediate-Release Carbidopa-Levodopa for the Treatment of Motor Fluctuations in Parkinson Disease: The RISE-PD Randomized Clinical Trial
Source: JAMA Neurol. 2023 Aug 14;80(10):1062–9. doi: 10.1001/jamaneurol.2023.2679 (PMC10425876; doi:10.1001/jamaneurol.2023.2679)
Supplement: Supplement 1. — Trial protocol [file jamaneurol-e232679-s001.pdf]

Table of Contents

IPX203-B16-02 Protocol Final: May 18, 2017 2

IPX203-B16-02 Amendment 1: August 30, 2017 144

IPX203-B16-02 Amendment 2: October 23, 2017 292

IPX203-B16-02 Amendment 3: Dececember 7, 2017 443

IPX203-B16-02 Amendment 4: September 28, 2018 594

IPX203 (CARBIDOPA-LEVODOPA) EXTENDED-RELEASE CAPSULES

IPX203-B16-02

A RANDOMIZED CONTROLLED STUDY TO COMPARE THE SAFETY AND EFFICACY OF IPX203 WITH IMMEDIATE-RELEASE CARBIDOPA-LEVODOPA IN PARKINSON'S DISEASE PATIENTS WITH MOTOR FLUCTUATIONS

SPONSOR

Impax Laboratories, Inc.,

acting through its Impax Specialty Pharma division (Impax)

30831 Huntwood Ave.

Hayward, CA 94544

Original Protocol, May 18, 2017

CONFIDENTIALITY STATEMENT

All information provided is the property of Impax Laboratories, Inc. and may not be divulged, published or otherwise disclosed without written consent of Impax. All information provided to the Investigator by the Sponsor, including clinical observations at the investigative site, shall be held strictly confidential and confined to the clinical personnel involved in conducting the study, under the supervision of the Investigator. This includes but is not limited to preclinical data, protocols, case report forms, and verbal or written communications. This information may be related in confidence to the Institutional Review Board or other committees functioning in a similar capacity. All reports, subject samples, data published or submitted to third parties will be identified by a coded number and initials only in order to maintain subject confidentiality.

SIGNATURE PAGE

Reviewed and approved by:

Robert Rubens, MD, MBA<br>Senior Director, Clinical Development	18 MAY 2017<br>Date
Barbara Pruitt, RN, BS<br>Associate Director, Clinical Operations	18 May 2017<br>Date
Yoshiko Stowell, PhD<br>Manager, Regulatory Affairs	18 May 17<br>Date
Phillip Dinh, PhD<br>Director, Statistics	18 MAY 17<br>Date
Aravind Mittur, PhD<br>Director, Clinical Pharmacology	05/18/2017<br>Date

INVESTIGATOR'S AGREEMENT

Protocol No.: IPX203-B16-02

Protocol Title: A Randomized Controlled Study to Compare the Safety and Efficacy of IPX203 with Immediate-Release Carbidopa-Levodopa in Parkinson's Disease Patients with Motor Fluctuations

I have read this protocol and agree to conduct the study as outlined herein, complying with the obligations and requirements of clinical investigators and all other requirements of International Conference on Harmonization (ICH), Good Clinical Practice (GCP), and the appropriate regulatory authority.

I will provide copies of the protocol and all pertinent information to all individuals responsible to me who assist in the conduct of this clinical study. I will discuss this material with them to ensure that they are fully informed regarding the study medication, the conduct of the study, and the obligations of confidentiality.

____ ____________________

Principal Investigator's signature Date

____

Principal Investigator's printed name

STUDY CONTACT INFORMATION

Changes in Impax study personnel listed on this page do not require a protocol amendment.

Role	Name and Contact Information
Sponsor	Impax Laboratories, Inc. acting through its Impax Specialty Pharma division (Impax)<br>30831 Huntwood Avenue<br>Hayward, CA 94544-7037<br>(510) 240-6000<br>FAX: (510) 240-6113 and (510) 240-6114
Medical Monitor	Robert Rubens, MD, MBA<br>Senior Director, Clinical Research & Development<br>Mobile: (510) 303-8207<br>Office: (510) 240-6012<br>E-mail: rrubens@impaxlabs.com
Statistician	Phillip Dinh, PhD<br>Director, Statistics<br>(510) 240-6402<br>E-mail: Phillip.Dinh@impaxlabs.com
Clinical Pharmacology	Aravind Mittur, PhD<br>Director, Clinical Pharmacology<br>(510) 240-6437<br>E-mail: amittur@impaxlabs.com
Clinical Research Associates	Barbara Pruitt, RN, BS<br>Associate Director, Clinical Operations<br>Office: (510) 240-6003<br>Mobile: (510) 329-8489<br>E-mail: bpruitt@impaxlabs.com
	Weiru Hong, MS<br>Senior CRA, Clinical Operations<br>Office: (510) 240-6078<br>Mobile: (510) 731-8309<br>E-mail: whong@impaxlabs.com
Serious Adverse Event Reporting	FAX: (866) 954-2064

1. SYNOPSIS

**Name of Sponsor/Company:** Impax Laboratories, Inc. acting through its Impax Specialty Pharma division (Impax)
**Name of Investigational Product:** IPX203 (carbidopa-levodopa) Extended-Release Capsules
**Name of Active Ingredients:** carbidopa (CD), levodopa (LD)
**Protocol Title:** A Randomized Controlled Study to Compare the Safety and Efficacy of IPX203 with Immediate-Release Carbidopa-Levodopa in Parkinson's Disease Patients with Motor Fluctuations
**Protocol No.:** IPX203-B16-02
**Study center(s):** Multicenter
**Phase of Development:** Phase 3
**Objectives:** To evaluate the safety and efficacy of IPX203 in comparison to IR CD-LD in the treatment of CD-LD-experienced subjects with Parkinson's disease (PD) who have motor fluctuations.
**Methodology:** This is a multicenter, randomized, double-blind, double-dummy, active-controlled, parallel-group study. The study will consist of a 3-week, open-label IR CD-LD dose adjustment period; a 4-week, open-label period for conversion to IPX203; followed by a 13-week double-blind treatment period with subjects randomized in a 1:1 ratio, stratified by center, to receive either IPX203 (with matching IR CD-LD placebo) or IR CD-LD (with matching IPX203 placebo).<br>Subjects may continue to take permitted non-CD-LD-based PD medications throughout the study if documented in their prestudy regimen and if dosing regimens have been stable for at least 4 weeks prior to Visit 1.<br>• Within 4 weeks following the Screening visit, eligible subjects will complete their PD Diaries on each of the 3 consecutive days immediately prior to Visit 1.<br>• Following Visit 1, qualified subjects will enter a 3-week, open-label IR CD-LD treatment period allowing for dose adjustment. Any adjustments to the IR CD-LD dosing regimen will be done in consultation with the Investigator or qualified site personnel and will be recorded. The IR CD-LD dosing regimen should be stable for at least 5 days prior to returning for Visit 2. Subjects will complete their 3-day PD Diaries on each of the 3 consecutive days immediately prior to Visit 2.<br>• Following completion of the IR CD-LD dose adjustment period, subjects will begin a 4-week open-label period for conversion to IPX203. The initial dosing regimen of IPX203 will be based on the most frequent dose of the subject's dosing regimen of IR CD-LD at the end of the dose adjustment period (Visit 2). A 100-mg unit dose of IR LD converts to approximately a 280-mg LD unit dose of IPX203 and a 50-mg unit dose of IR LD converts to approximately a 140-mg LD unit dose of IPX203, but with a longer duration of effect. It is recommended that IPX203 should be dosed approximately every 8 hours with the goal of optimizing the therapeutic effect (minimize "Off" time without causing troublesome dyskinesia or other dopaminergic side effects). Any adjustments to the IPX203 dosing regimen will be done in consultation with the Investigator or qualified site personnel and will be recorded. Subjects will return to the clinic in 2 weeks for Visit 3 followed by Visit 4, 2 weeks later. The IPX203 dosing regimen should be stable for at least 5 days prior to returning for Visit 4. Subjects will complete their 3-day PD Diaries on each of the 3 consecutive days immediately prior to Visit 4.

- Subjects who successfully complete the IPX203 dose conversion period will be randomized in 1:1 ratio, stratified by center, at Visit 4 into one of two parallel treatment arms of IPX203 (with matching IR CD-LD placebo) or IR CD-LD (with matching IPX203 placebo). The subjects will undergo 13 weeks of double-blind maintenance therapy with the stable dosing regimen established at the end of Week 3 (Visit 2) for IR CD-LD and at the end of Week 7 (Visit 4) for IPX203. Subjects will return to the clinic for 3 visits (Visits 5, 6, and 7) and will complete their 3-day PD Diaries on each of the 3 consecutive days immediately prior to each of these visits.

Number of patients (planned): Assuming approximately an 18% prerandomization drop-out, approximately 510 subjects will be enrolled to randomize 420 subjects.

Diagnosis and main criteria for inclusion:

Inclusion Criteria

- Male or female subjects diagnosed at age ≥ 40 years with PD, consistent with the United Kingdom Parkinson's Disease Society Brain Bank Diagnostic Criteria and who are being treated with stable regimens of CD-LD but experiencing motor fluctuations.
- Hoehn and Yahr Stages 1, 2, 3, or 4 in the "On" state (part of Movement Disorders Society version of the Unified Parkinson's Disease Rating Scale [MDS-UPDRS] Part III)
- Montreal Cognitive Assessment (MoCA) score ≥ 24 at Screening Visit in "On" state.
- By history, for the 4 weeks prior to Screening, the subject experiences daily "wearing-off" episodes with periods of bradykinesia in combination with at least one of rest tremor or rigidity, experiences an "Off" state upon awakening on most mornings, and reports an average of at least 2.5 cumulative hours per day of "Off" time during the waking hours.
- Able to differentiate "On" state from "Off" state as determined by at least 75% concordance with a trained rater in "On/Off" ratings for 8 ratings over a 4-hour training period. The concordance must include at least 1 "On" and 1 "Off" rating and must be achieved within two 4-hour training sessions.
- At Visit 1, review of the 3-day PD Diaries confirms the following: that the subject is able to properly complete the Diaries with valid entries; and that the subject has an average of at least 2.5 hours per day of "Off" time during waking hours over the 3 days with at least 1.5 hours of cumulative "Off" time on each day.
- Responsive to CD-LD therapy and currently being treated on a stable regimen with CD-LD for at least 4 weeks prior to Visit 1 and:
  - Requires at least 100 mg of IR CD-LD for the first morning dose
  - Requires a total daily dose of at least 300 mg of LD and takes a maximum total daily dose of 2400 mg LD, comprising IR CD-LD alone or IR CD-LD in combination with a single daily bedtime dose of CR CD-LD
  - Has a dosing frequency of 3 to 9 times daily of CD-LD
  - By history, typically experiences an "On" response with the first dose of IR CD-LD of the day but the efficacy of this dose typically lasts less than 4 hours.
- At Screening, the subject has predictable "Off" periods defined by a score of 1 or 2 on Item #4.5 (Complexity of Motor Fluctuations) of the MDS-UPDRS Part IV B (Motor Fluctuations).
- At Screening, the MDS-UPDRS Part III total score in the "Off" state is at least 20 units.

Exclusion Criteria

- Used any doses of controlled-release (CR) CD-LD apart from a single daily bedtime dose within 4 weeks prior to Visit 1.

- Used any dose of Rytary for the past 4 weeks prior to Visit 1 or were considered IPX066 or Rytary failures for reasons of efficacy or safety.
- Had prior neurosurgical treatment for PD or if such procedure is planned or anticipated during the study period.
- Allergic to any excipient in the study drugs.
- History of glaucoma with intraocular pressures that are elevated despite appropriate medical management.
- History of seizure or epilepsy and experienced at least 1 seizure during the past 12 months or has not been compliant with medically recommended therapy or visits.
- History of myocardial infarction with residual atrial, nodal, or ventricular arrhythmias that are not controlled with medical and/or surgical interventions. A recent (≤ 12 months) history of myocardial infarction with secondary arrhythmias is exclusionary regardless of the therapeutic control.
- Received within 4 weeks of Screening or planning to take during participation in the clinical study:
  - Any doses of a CR CD-LD apart from a single daily bedtime dose, any doses of Rytary, additional CD (eg, Lodosyn) or benserazide (eg, Serazide), or catechol-O-methyl transferase inhibitors (entacapone or tolcapone) or medications containing these inhibitors (Stalevo),
  - Nonselective monoamine oxidase inhibitors (MAOI), apomorphine, or antidopaminergic agents, including antiemetics.
- Subjects who have previously participated in an IPX203 study.

Investigational product, dosage and mode of administration: IPX203 (carbidopa-levodopa) Extended-Release capsules, containing 35-140 mg of CD-LD and matching placebo, for oral administration.

Reference therapy, dosage and mode of administration: Immediate-Release carbidopa-levodopa (IR CD-LD) tablets containing 25-100 mg of CD-LD, and matching placebo, for oral administration.

Duration of treatment: Approximately 24 weeks, including up to 4 weeks following Screening, 3 weeks of IR CD-LD dose adjustment, 4 weeks of IPX203 dose conversion, and 13 weeks of double-blind therapy following randomization.

Criteria for evaluation:

Baseline is defined as assessments done at Visit 4 (randomization visit). Study Entry is defined as assessments done at Visit 1 (study entry visit).

Efficacy:

- Primary endpoint: Change from baseline in “Good on” time in hours per day, averaged over 3 PD Diary days, at the end of double-blind treatment period (Visit 7 or early termination). “Good on” time is derived from the 3-day PD Diary and is defined as the sum of “On” time without dyskinesia and “On” time with nontroublesome dyskinesia.
- Key secondary endpoints:
  - Change from baseline in “Off” time in hours per day, averaged over 3 PD Diary days at the end of double-blind treatment period (Visit 7 or early termination)
  - Proportion of subjects with either “much improved” or “very much improved” in Patient Global Impression of Change (PGI-C) scores at the end of double-blind treatment period (Visit 7 or early termination)

  - Change from baseline in the average number of motor fluctuations per day averaged over the 3 PD Diary days. A motor fluctuation is defined as a change from “Off” to “On” state or from “On” to “Off” state.
  - Change from baseline in the sum of MDS-UPDRS Parts II and III at the end of double-blind treatment period (Visit 7 or early termination)
- Additional endpoints:

  The following endpoints will be evaluated (at the post-randomization visits) as change from baseline (Visit 4) as well as change from the study entry (Visit 1), when applicable, by visits:
  - Percent “Off” time during waking hours derived from the 3-day PD Diaries
  - Average time to “On” upon awakening
  - Average duration of each continuous “Good on”
  - Hours of (1) “Off” time (from Visit 1), (2) “Good on” time (from Visit 1) (3) “On” time with dyskinesia, (4) “On” time with troublesome dyskinesia, and (5) “On” time with nontroublesome dyskinesia and (6) asleep time derived from the 3-day PD Diaries
  - MDS-UPDRS total score (sum of Parts I, II, III, and IV) and each part separately
  - MDS-UPDRS Parts II and III combined (from Visit 1)
  - MDS-UPDRS Part II Question 2.9
  - Parkinson’s Disease Questionnaire-39 (PDQ-39) total score and individual domains
  - Non-Motor Symptom assessment scale for Parkinson’s Disease (NMSS) total score and individual domains
  - Parkinson’s Disease Sleep Scale-2 (PDSS-2) total score and individual domains
  - PDSS-2 items 9, 10, 11, 12, and 13 combined
  - Parkinson Anxiety Scale (PAS) total score and individual domains
  - Patient Global Impression of Severity Scale (PGI-S)
  - Clinical Global Impression of Severity Scale (CGI-S)
  - PGI-C scores
  - Clinical Global Impression of Change (CGI-C) scores

  Proportion of subjects will be tabulated for the following endpoints:
  - “Off” time reduction of 0.5, 1, 1.5, 2, 2.5, and 3 hours
  - “Good on” time improvement of at least 1, 1.5, 2, 2.5, and 3 hours
  - “On” upon awakening and “Good on” upon awakening
  - PGI-S: “severely ill” or “extremely severely ill”
  - CGI-S: “severely ill” or “among the most extremely ill of subjects”
  - CGI-C: “much improved” or “very much improved”
- **Safety:** electrocardiograms (ECGs), clinical laboratory tests, physical examinations, Columbia-Suicide Severity Rating Scale (C-SSRS), and supine and standing orthostatic vital signs; adverse events and concomitant medications evaluated throughout the course of the study.

Statistical methods: For the primary endpoint, assuming a difference of 1 hour between IPX203 and IR CD-LD in “Good on” time and a standard deviation of the treatment difference to be 3.0 hours, a sample size of 210 per arm will be needed to ensure at least 90% power at a 0.05 significance level. Assuming approximately an 18% prerandomization drop-out, approximately 510 subjects would need

to be enrolled to randomize 420 subjects.

The primary efficacy endpoint of change from baseline in "Good on" time will be analyzed using a mixed effects for repeated measures (MMRM) model. The model will include baseline (Visit 4) "Good on" as a covariate, treatment, visit, and pooled center as fixed effects, and a treatment-by-visit interaction. The model will employ an unstructured within subject covariance matrix and a restricted maximum likelihood (REML) estimation method.

The key secondary endpoints (change from baseline in "Off" time, change from baseline in motor fluctuations, and change from baseline in MDS-UPDRS Parts II and III combined) will be analyzed using MMRM models similar to the primary analysis model. The proportion of subjects with either "much improved" or "very much improved" on the PGI-C will be analyzed using Cochran-Mantel-Haenszel test to compare the two treatment groups with pooled center as a stratification factor.

In order to control the type I error rate, the primary and key secondary endpoints will be tested in a single hierarchical order: (1) change from baseline in "Good on" time, (2) change from baseline in "Off" time, (3) proportion of subjects with either "much improved" or "very much improved" in PGI-C, (4) change from baseline in the number of motor fluctuations, (5) change from baseline in the sum of MDS-UPDRS Parts II and III.

Quantitative safety data will be summarized using descriptive statistics and frequency distributions. Qualitative safety data will be summarized by frequencies and percentages. All summaries will be presented by treatment arms.

2. TABLE OF CONTENTS, LIST OF TABLES, AND LIST OF FIGURES

TABLE OF CONTENTS

1. SYNOPSIS 5
2. TABLE OF CONTENTS, LIST OF TABLES, AND LIST OF FIGURES 10
3. LIST OF ABBREVIATIONS AND DEFINITIONS OF TERMS....16
4. INTRODUCTION 18
5. TRIAL OBJECTIVES 22
6. INVESTIGATIONAL PLAN....23
6.1. Overall Study Design....23
6.2. Number of Subjects 24
6.3. Treatment Assignment....24
6.4. Dosing and Dose Determination Criteria 24
6.4.1. IR CD-LD Dose Adjustment Period....25
6.4.2. IPX203 Dose Conversion Period....25
6.4.3. Double-Blind Maintenance Period 26
6.5. Criteria for Study Termination 26
7. SELECTION AND WITHDRAWAL OF SUBJECTS....27
7.1. Subject Inclusion Criteria 27
7.2. Subject Exclusion Criteria 28
7.3. Subject Withdrawal Criteria 29
8. STUDY PROCEDURES....31
8.1. Screening Visit....35
8.2. Visit 1 – Start of IR CD-LD Dose Adjustment....36
8.2.1. Prior to Visit 1 36
8.2.2. At Visit 1....36
8.2.3. Post Visit 1....37
8.3. Visits 2 (Week 3) and Visit 3 (Week 5) – IPX203 Dose Conversion 37
8.3.1. Prior to Visit 2 37
8.3.2. Prior to Visit 3 37
8.3.3. At Visits 2 and 3 37

8.3.4. Post Visits 2 and 3 38
8.4. Visit 4 (Week 7) – Randomization 38
8.4.1. Prior to Visit 4 38
8.4.2. At Visit 4....38
8.5. Visit 5 (Week 10) and Visit 6 (Week 15) 39
8.5.1. Prior to Visit 5 and 6....39
8.5.2. At Visit 5 and 6....39
8.6. Visit 7 (Week 20) – End of Study/Study Exit 40
8.6.1. Prior to Visit 7 40
8.6.2. At Visit 7....40
8.7. Early Termination....41
8.7.1. Subjects who Terminate Prior to Randomization....41
8.7.2. Subjects who Terminate Early after Randomization 41
8.8. Blood Volume....41
9. TREATMENT OF SUBJECTS....42
9.1. Description of Study Drug....42
9.2. Concomitant Medications....42
9.2.1. Permitted PD Medications....42
9.2.2. Prohibited Medications and Procedures 42
9.3. Treatment Compliance....43
9.4. Randomization and Blinding 43
10. STUDY DRUG MATERIALS AND MANAGEMENT 44
10.1. Study Drug....44
10.2. Study Drug Packaging and Labeling 44
10.3. Study Drug Storage....44
10.4. Study Drug Administration....45
10.5. Study Drug Dispensing and Accountability 45
10.6. Study Drug Handling and Disposal 45
11. ASSESSMENT OF EFFICACY 46
11.1. Parkinson’s Disease Diary....46
11.2. Patient and Investigator Global Assessments 46

11.3. Movement Disorders Society Version of Unified Parkinson’s Disease Rating Scale....46
11.4. Additional Assessments....47
12. ASSESSMENT OF SAFETY....48
12.1. Safety Parameters....48
12.2. Adverse Events....48
12.2.1. Definition of Adverse Event....48
12.2.2. Recording Adverse Events....48
12.2.3. Follow-up....49
12.2.4. Relationship to Study Drug....49
12.2.5. Assessment of Severity....49
12.3. Serious Adverse Events....49
12.3.1. Definition of Serious Adverse Event....49
12.3.2. Reporting Serious Adverse Events....50
12.4. Pregnancy....50
12.5. Other Safety Parameters and Related Information....51
13. STATISTICS....52
13.1. Study Design and Sample Size Estimation....52
13.2. Demographics/Baseline Comparability....52
13.3. Analysis of Efficacy Data....52
13.3.1. Primary Efficacy Endpoint....52
13.3.2. Key Secondary Efficacy Endpoints....52
13.3.3. Additional Efficacy Endpoints....53
13.4. Sensitivity Analyses of the Primary Endpoint and Key Secondary Endpoints....54
13.5. Multiplicity Adjustments....55
13.6. Analysis Populations....55
13.6.1. Safety Analysis Set....55
13.6.2. Intent-to-treat Analysis Set....55
13.6.3. Modified Intent-to-treat Analysis Set....55
13.6.4. Completers Analysis Set....56
13.7. Handling of Missing Data....56
13.7.1. Missing Data for PD Diaries....56

13.7.2. Missing Data for Global Assessments (PGI-C, CGI-C, PGI-S and CGI-S)...............56
13.7.3. Missing Data for MDS-UPDRS Parts II and III...57
13.8. Analysis of Safety..57
14. ADMINISTRATIVE PROCEDURES 58
14.1. Guidelines for Good Clinical Practice...58
14.2. Institutional Review Board Approval...58
14.3. Informed Consent 58
14.4. Study Monitoring..58
14.5. Protocol Amendments 58
14.6. Termination of Study..59
14.7. Case Report Forms 59
14.8. Investigator's Final Conduct Report...59
14.9. Records Retention...59
15. PUBLICATION POLICY 60
16. LIST OF REFERENCES..61
17. APPENDICES 62
APPENDIX A. PRESCRIBING INFORMATION FOR IR CD-LD 62
APPENDIX B. UNITED KINGDOM PARKINSON'S DISEASE SOCIETY BRAIN BANK DIAGNOSTIC CRITERIA FOR PARKINSON'S DISEASE 74
APPENDIX C. MONTREAL COGNITIVE ASSESSMENT (MOCA) 75
APPENDIX D. MOVEMENT DISORDERS SOCIETY VERSION OF UNIFIED PARKINSON'S DISEASE RATING SCALE (MDS-UPDRS) 81
APPENDIX E. PATIENT GLOBAL IMPRESSION OF CHANGE (PGI-C) 114
APPENDIX F. CLINICAL GLOBAL IMPRESSION OF CHANGE (CGI-C).......................115
APPENDIX G. PATIENT GLOBAL IMPRESSION OF SEVERITY (PGI-S) 116
APPENDIX H. CLINICAL GLOBAL IMPRESSION OF SEVERITY (CGI-S)....................117
APPENDIX I. 39-ITEM PARKINSON'S DISEASE QUESTIONNAIRE (PDQ-39).............118
APPENDIX J. GASTROPARESIS CARDINAL SYMPTOM INDEX (GCSI)......................122
APPENDIX K. NON-MOTOR SYMPTOM ASSESSMENT SCALE FOR PARKINSON'S DISEASE (NMSS)...124
APPENDIX L. PARKINSON'S DISEASE SLEEP SCALE-2 (PDSS-2) 127
APPENDIX M. PARKINSON ANXIETY SCALE (PAS) 128
APPENDIX N. COLUMBIA-SUICIDE SEVERITY RATING SCALE (C-SSRS)................132

APPENDIX O. PARKINSON'S DISEASE DIARY 139

APPENDIX P. EXCIPIENTS IN IPX203, IPX203 PLACEBO, IR CD-LD, AND IR PLACEBO 140

APPENDIX Q. CLINICAL LABORATORY STUDIES 141

LIST OF TABLES

Table 1: Abbreviations and Specialist Terms 16

Table 2: LD Dosage in Study IPX203-B14-02 19

Table 3: Recommended Starting IPX203 LD Dosing Regimen Based on the Dosing Regimen of IR CD-LD at the End of the Dose Adjustment Period 25

Table 4: Events Schedule for Impax Study IPX203-B16-02 32

Table 5: Study Drugs for Study IPX203-B16-02 42

LIST OF FIGURES

Figure 1: Study Flow Chart 24

3. LIST OF ABBREVIATIONS AND DEFINITIONS OF TERMS

The following abbreviations and specialist terms are used in this study protocol.

Table 1: Abbreviations and Specialist Terms

Abbreviation or Specialist Term	Explanation
AADC	aromatic amino acid decarboxylase
ADL	activities of daily living
AE	adverse event
ANCOVA	analysis of covariance
ANOVA	analysis of variance
CD	carbidopa
CGI-C	Clinical Global Impression of Change
CGI-S	Clinical Global Impression of Severity
CR	controlled release
CRF	case report form
C-SSRS	Columbia-Suicide Severity Rating Scale
ECG	electrocardiogram
ER	extended release
FDA	Food and Drug Administration
GCP	Good Clinical Practice
GCSI	Gastroparesis Cardinal Symptom Index
HIPAA	Health Insurance Portability and Accountability Act
ICF	informed consent form
ICH	International Conference on Harmonization
IEC	independent ethics committee
IR	immediate release
IRB	institutional review board

Abbreviation or Specialist Term	Explanation
IWRS	interactive web response system
LD	levodopa
LOCF	last observation carried forward
MAOI	monoamine oxidase inhibitors
M-EDL	Motor Aspects of Experiences of Daily Living
MDS-UPDRS	Movement Disorders Society version of the Unified Parkinson's Disease Rating Scale
MMRM	mixed effect model for repeated measures
MoCA	Montreal Cognitive Assessment
nM-EDL	Non-Motor Aspects of Experiences of Daily Living
NMSS	Non-Motor Symptom Assessment Scale
PAS	Parkinson Anxiety Scale
PD	Parkinson's disease
PDQ-39	39-item Parkinson's Disease Questionnaire
PDSS-2	Parkinson's Disease Sleep Scale-2
PGI-C	Patient Global Impression of Change
PGI-S	Patient Global Impression of Severity
PK	pharmacokinetic (adjective)<br>pharmacokinetics (singular noun)
PI	principal investigator
REML	restricted maximum likelihood
SAE	serious adverse event
SAP	statistical analysis plan
SD	standard deviation
TEAE	treatment-emergent adverse event
US	United States

4. INTRODUCTION

Parkinson's disease (PD) is a progressive neurodegenerative disorder of the extrapyramidal nervous system. Levodopa (LD) used in combination with carbidopa (CD) is considered the gold standard for the symptomatic treatment of PD. LD is a dopamine precursor converted to dopamine by aromatic amino acid decarboxylase (AADC). Carbidopa is an AADC inhibitor that does not cross the blood-brain barrier. When used in combination with LD, CD increases the plasma half-life of LD from 50 minutes to 1.5 hours. Carbidopa inhibits the conversion of LD into dopamine in the periphery, thereby reducing the peripheral side-effects caused by dopamine and increasing the amount of LD available for transport into the brain. The administration of CD with LD reduces the dose of LD required to produce a dopaminergic response by about 75 percent (Sinemet prescribing information).

Due to its proven efficacy, LD is prescribed eventually to most subjects with PD. However, long-term use of LD is associated with certain complications, including "wearing off" or "end-of-dose effect," where symptom control decreases causing the drug effects to wear off sooner. As the disease progresses further, motor complications, namely dyskinesias and motor "On/Off" fluctuations, develop in about 50% of the patients after 5 years of treatment (Fahn 1999). Such motor complications can be a significant source of disability and their management is a major unmet need in the treatment of PD.

Mechanisms underlying motor complications involving dyskinesias and "On/Off" fluctuations in PD are unclear. The pulsatile nature of standard orally administered LD is thought to contribute to the appearance of motor complications. Chronic intermittent pulsatile stimulation of the dopamine receptors that are under tonic control contributes to the development of dyskinesia in PD animal models as compared to animals treated with continuous infusion (Juncos et al 1989, Engber et al 1989, Blanchet et al 1995). In addition, unreliable absorption of LD potentially due to erratic gastric empting and variable in vivo dissolution of LD products is thought to contribute to the delay or inadequate response after oral dosing with standard CD-LD products (Melamed et al 1986, Kurlan et al 1988, Stocchi et al 1994). These findings suggest that motor complications in patients with PD may be less likely to develop with continuous dopaminergic stimulation.

Intraduodenal infusion of LD has been shown to significantly reduce motor complications and to reduce "Off" time. The findings of infusion studies in PD patients indicate that the maintenance of stable plasma LD concentrations and the avoidance of low trough levels are effective in reducing "Off" hours, increasing "On" hours without disabling dyskinesia, and reducing the severity of dyskinesia versus standard oral LD formulations (Mizuno 2007, Nilsson et al 2001, Nyholm et al 2005, Stocchi et al 2005). These findings provide a strong rationale for the development of an extended-release (ER) oral dosage form that delivers a constant LD plasma concentration in order to optimize relief of PD symptoms, and to minimize "Off" time and dyskinesia.

IPX203 is an investigational product containing CD-LD that is being developed by Impax Laboratories, Inc. through its Impax Specialty Pharma division (Impax). The primary objective of the IPX203 program is to develop an extended-release product that can attain therapeutic LD plasma concentrations rapidly and maintain constant LD plasma concentrations for a longer

duration than currently approved products with minimal peak-to-trough fluctuations. IPX203 is designed to be dosed approximately every 8 hours.

Impax characterized the PK and pharmacodynamics of IPX203 in Study IPX203-B14-02, a single dose trial in subjects with advanced PD versus IR CD-LD and Rytary (carbidopa and levodopa) extended-release capsules. Twenty-six (26) subjects were randomized with 25 subjects completing all 3 treatments. One subject discontinued study early due to subject withdrawal. The doses of IPX203 and Rytary were determined on the basis of each subject's prestudy baseline morning dose of IR CD-LD (Table 2).

Table 2: LD Dosage in Study IPX203-B14-02

Prestudy Baseline Morning IR CD-LD (mg)	IR CD-LD (mg)	Rytary (mg)	IPX203 (mg)
100	100	340	360
150	150	485	540
200	200	630	720
250	250	780	810

Administration of IPX203 yielded an initial increase in LD plasma concentrations that was similar to IR CD-LD but maintained LD concentrations for a longer duration than either IR CD-LD or Rytary. The bioavailability of LD (based on AUC_{∞}) from IPX203 was 78% relative to IR CD-LD and about 14% more than Rytary. Plasma exposure to LD (C_{max} and AUC_{∞}) following IPX203 increased in an approximately dose-proportional manner. Pharmacodynamic effects as measured by change from baseline scores on MDS-UPDRS Part III were consistent with the PK profiles of LD. Following IPX203 treatment, decrements in the Part III total score (reflecting improvements in motor symptoms) lasted for a longer duration than either IR CD-LD or Rytary. IPX203 provided a longer duration of effect compared with IR CD-LD and Rytary, including "Off" time and "Good on" time based on the Assessment of Subject's Motor State and on a range of improvement thresholds of the MDS-UPDRS Part III. The results were consistent with the prolonged LD plasma concentration profile with IPX203 compared to IR CD-LD or Rytary and support a dosing interval of approximately 8 hours.

Of the 26 subjects who received at least one of the 3 treatments, 9 (34.6%) reported at least one treatment-emergent adverse event (AE). None of these subjects reported a serious AE (SAE) nor did any subjects prematurely discontinue the study because of an AE. Adverse events were reported by more subjects during IR (28.0%) and IPX203 (19.2%) than during Rytary (8.0%) treatment. None of the reported AEs were classified as "severe." Adverse events reported by 2 or more subjects include: Dizziness (3 subjects), nausea (2 subjects), and hypertension (2 subjects). The numbers of subjects reporting these AEs during any single treatment period were small (0 to 2 subjects). Two subjects reported dizziness during IR CD-LD treatment and one subject each during IPX203 and Rytary treatments. Hypertension was reported by a total of 2 subjects, both reporting this AE during IPX203 and IR CD-LD treatments and 1 subject during Rytary treatment. Two subjects reported nausea only during the IR CD-LD treatment period. Of the 9 subjects reporting AEs, 6/26 (23.1%) of subjects reported AEs that were assessed as related to treatment, including all of the reports of dizziness, nausea, and dyskinesia (1 subject).

In addition, more than 14 patients have received multiple doses (up to 2 weeks) of IPX203 in a multiple-dose PK, pharmacodynamics, efficacy and safety study (IPX203-B16-01). In this study, the dose regimen of IPX203 was based on the subject's prestudy morning dose of IR CD-LD and the most frequent prestudy dose of LD in the afternoon and evening, similar to the planned conversion for this phase 3 study.

Data from this multiple dose study confirmed the PK and pharmacodynamic results observed in the single dose study with IPX203:

- IPX203 shows a rapid increase in LD concentrations followed by extended-release characteristics. Following IPX203; initial increases in LD concentrations were comparable to that from IR CD-LD. Bioavailability of LD following IPX203 was ~ 100% relative to IR CD-LD. LD plasma concentrations were sustained longer after IPX203 treatment than after IR CD-LD and support dosing every 8 hours. No accumulation of LD was evident at steady-state following IPX203 or IR CD-LD. Plasma LD concentrations following IPX203 were characterized by lower peak-to-trough fluctuation. No time-variant or time-dependent changes were noted in PK of CD or LD following IPX203.
- IPX203 demonstrated an onset of effect that was comparable to IR CD-LD in MDS-UPDRS Part III scores. IPX203 prolonged the duration over which MDS-UPDRS Part III scores were improved by prespecified threshold changes from baseline (≥4, ≥7, and ≥13 units).
- IPX203 provides a significant decrease in "Off" time and a significant increase in "Good on" time compared to IR CD-LD treatment on Day 1 and Day 15 when assessed by the Investigator's Assessment of Subject's Motor State. Subjects treated with IPX203 did not experience a significant increase in "On" time with troublesome dyskinesia compared to IR CD-LD.
- Subjects achieved significant improvements in "Off time," "Good on time," frequency of motor state fluctuations, and proportion of subjects awakening with a "Good On" based on the 3-day PD Diary.
- For the cohort of subjects (15 subjects) who have been enrolled in the multiple dose study:
  - A total of 40% (6/15) of treated subjects reported at least 1 AE, including 33.3% (5/15) during IPX203 treatment and 15.4% (2/13) during IR CD-LD treatment. A total of 5 subjects reported AEs that were related to treatment (5 while taking IPX203 and 1 while taking IR CD-LD).
  - There have been no reports of SAEs, severe AEs, or subjects discontinuing the study because of an AE.
  - Dyskinesia was the only AE reported by more than 1 subject during any treatment period, with 26.7% (4/15) during IPX203 treatment vs. 0% (0/13) during IR CD-LD treatment. These reports of dyskinesia occurred during the dose conversion and resolved quickly with dose reductions.

The current protocol, Study IPX203-B16-02, is a multicenter, randomized, double-blind, double-dummy, active-controlled, parallel-group, phase 3 study. It is designed to compare the efficacy, safety and tolerability of IPX203 with IR CD-LD following multiple doses over 13 weeks. Based on results and observations available to-date, the following IPX203 dosing guideline will be utilized in the present study (IPX203-B16-02):

- Each 100-mg unit dose of IR CD-LD will be converted to a 280-mg LD unit dose of IPX203;
- The initial recommended dose of IPX203 will be based on an algorithm that determines the most frequent prestudy dose of CD-LD (IR CD-LD alone or in combination with a single bedtime dose of CR);
- IPX203 will be administered every 8 hours.

The recommended initial unit conversion doses of IPX203 in this study (100 mg of IR LD converts to 280 mg of IPX203) are comparable to the approximate unit dose conversion of Rytary (a marketed ER formulation of CD-LD: 100 mg of IR LD converts to 145 mg × 2 capsules = 290 mg of Rytary) with a reduced initial dosing frequency. However, initial Rytary dosing is frequently every 6 hours during waking hours plus a nighttime dose or dosing 4 times a day every 6 hours.

5. TRIAL OBJECTIVES

To evaluate the safety and efficacy of IPX203 in comparison to IR CD-LD in the treatment of CD-LD experienced subjects with Parkinson’s disease (PD) who have motor fluctuations.

6. INVESTIGATIONAL PLAN

6.1. Overall Study Design

This is a multicenter, randomized, double-blind, double-dummy, active-controlled, parallel-group study. Subjects may continue to take permitted non-CD-LD-based PD medications throughout the study if documented in their prestudy regimen and if dosing regimens have been stable for at least 4 weeks prior to Visit 1. Within 4 weeks following the Screening visit, eligible subjects will complete their PD Diaries on each of the 3 consecutive days immediately prior to Visit 1.

Following Visit 1, qualified subjects will enter a 3-week, open-label IR CD-LD treatment period allowing for dose adjustment. During the IR CD-LD dose adjustment period, the initial dosing regimen of IR CD-LD will be the same as the subject's stable prestudy regimen unless they were taking a single daily bedtime dose of CR CD-LD, either alone or within an hour of a dose of IR CD-LD, in which case, the CR CD-LD dose will be discontinued and substituted with a 1:1 milligram-equivalent dose of IR CD-LD. A "bedtime dose" is defined as the last daytime dose of CD-LD taken within 1 hour of onset of the subject's normal nighttime sleep period. The dosing regimen may be adjusted during the dose adjustment period. Any adjustments to the IR CD-LD dosing regimen will be recorded. The IR CD-LD dosing regimen should be stable for at least 5 days prior to returning for Visit 2. Subjects will complete their 3-day PD Diaries on each of the 3 consecutive days immediately prior to Visit 1.

Following completion of the IR CD-LD dose adjustment period, subjects will begin a 4-week open-label period for conversion to IPX203. The initial dosing regimen of IPX203 during the conversion period will be based on the subject's dosing regimen of IR CD-LD at the end of the dose adjustment period (Visit 2) according to Table 3. A 100-mg unit dose of IR LD converts to approximately a 280-mg LD unit dose of IPX203 but with a longer duration of effect. It is recommended that IPX203 should be dosed approximately every 8 hours with the goal of optimizing the therapeutic effect (minimize "Off" time without causing troublesome dyskinesia or other dopaminergic side effects). Any adjustments to the IPX203 dosing regimen will be recorded. Subjects will return to the clinic in two weeks for Visit 3 followed by Visit 4 two weeks later. The IPX203 dosing regimen should be stable for at least 5 days prior to returning for Visit 4. Subjects will also be instructed to complete their 3-day PD Diaries on each of the 3 consecutive days immediately prior to Visit 4.

The study staff will call the subjects approximately every 1 to 3 days during the IR CD-LD dose adjustment and IPX203 dose conversion periods. The calls are to ensure timely and appropriate dosing adjustments and to ensure that the subject is able to follow and adhere to the dosing instructions. The contacts may be less frequent after initial dose adjustments have been made. Any changes in the dosing regimen will be in consultation with the Investigator or qualified site personnel and will be documented.

Subjects who successfully complete the IPX203 dose conversion periods will be randomized, stratified by center, in a 1:1 ratio at Visit 4 into one of two parallel treatment arms of IPX203 (with matching IR CD-LD placebo) or IR CD-LD (with matching IPX203 placebo). The subjects will undergo 13 weeks of double-blind, double-dummy maintenance therapy with the

stable dosing regimen established at the end of Week 3 (Visit 2) for IR CD-LD and at the end of Week 7 (Visit 4) for IPX203. Subjects will return to the clinic for 3 visits (Visits 5, 6, and 7) and will be instructed to complete their 3-day PD Diaries on 3 consecutive days immediately prior to each of the 3 visits.

Figure 1: Study Flow Chart

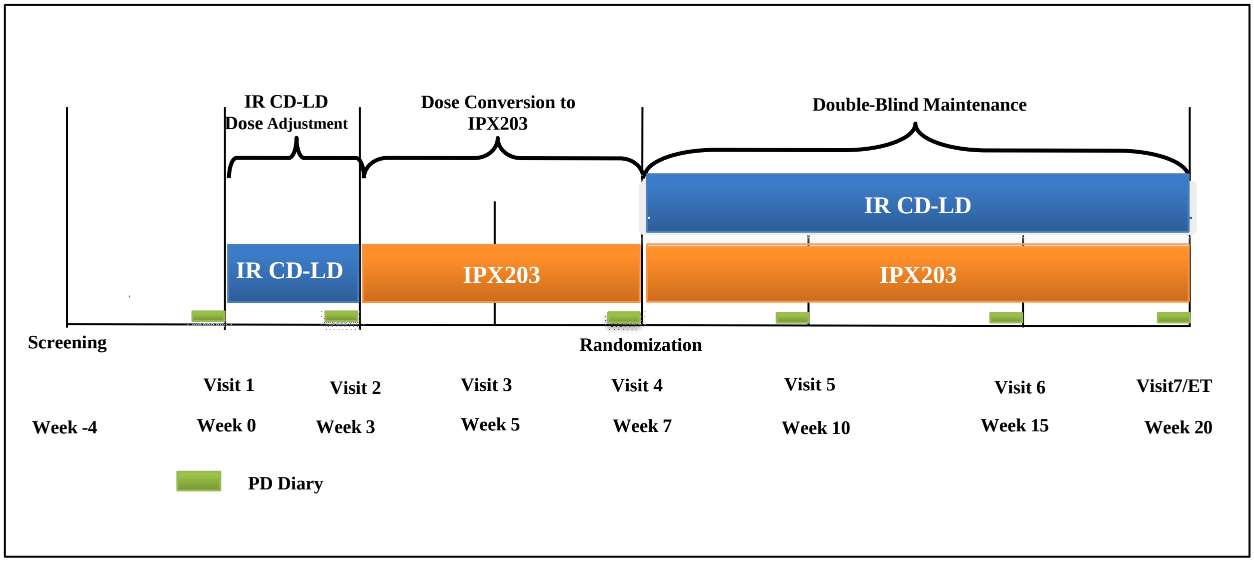

Abbreviations: IR=immediate-release, CD=carbidopa, LD=levodopa, ET=early termination

6.2. Number of Subjects

Assuming approximately an 18% prerandomization drop-out, approximately 510 subjects will be enrolled to randomize 420 subjects.

6.3. Treatment Assignment

Investigational product: IPX203 (carbidopa-levodopa) Extended-Release Capsules containing 35-140 mg of CD-LD, for oral administration.

Reference therapy: Immediate-Release carbidopa-levodopa (IR CD-LD) tablets containing 25-100 mg of CD-LD, for oral administration.

Subjects will be randomly assigned to one of two parallel treatment arms to receive either IPX203 (with matching IR CD-LD placebo) or IR CD-LD (with matching IPX203 placebo) during the double-blind maintenance therapy portion of the study.

6.4. Dosing and Dose Determination Criteria

IR CD-LD will be supplied as tablets containing 25-100 mg of CD-LD. IR CD-LD tablets may be split to achieve the required dose.

IPX203 will be supplied as capsules containing 35-140 mg of CD-LD. The suggested doses and regimen of IPX203 are intended to provide an onset of effect comparable to the subject’s

prestudy IR LD regimen and to extend the duration of effect. A 100-mg unit dose of IR LD converts to approximately a 280-mg LD unit dose of IPX203.

6.4.1. IR CD-LD Dose Adjustment Period

During the 3-week IR CD-LD dose adjustment period, the initial dosing regimen of IR CD-LD will be the same as the subject's stable prestudy regimen unless the subject is taking a single daily bedtime dose of CR CD-LD, in which case, the CR CD-LD dose will be discontinued and substituted with a 1:1 milligram-equivalent dose of IR CD-LD. A "bedtime dose" is defined as the last daytime dose of CD-LD taken within 1 hour of onset of the subject's normal nighttime sleep period. The dosing regimen may be adjusted during the dose adjustment period. Any adjustments to the IR CD-LD dosing regimen will be done in consultation with the Investigator or qualified site personnel and will be recorded. The subject must be on a stable dosing regimen of IR CD-LD for at least 5 days prior to returning for Visit 2.

6.4.2. IPX203 Dose Conversion Period

During the 4-week IPX203 dose conversion period, the suggested initial dosing regimen of IPX203 will be based on the subject's dosing regimen of IR CD-LD at the end of the dose adjustment period (Visit 2). A 100-mg unit dose of IR LD converts to approximately a 280-mg LD unit dose of IPX203, and a half tablet (50-mg unit dose of IR LD) converts to approximately a 140-mg LD unit dose of IPX203. To facilitate conversion of subjects from IR CD-LD to IPX203, Table 3 presents recommended starting dose regimens. It is recommended that IPX203 should be dosed approximately every 8 hours. The dosing regimen of IPX203 may be adjusted during the dose conversion period to achieve the optimal balance of efficacy and tolerability (minimize "Off" time without causing troublesome dyskinesia or other dopaminergic side effects). The subject must be on a stable dosing regimen of IPX203 for at least 5 days prior to returning for Visit 4.

Table 3: Recommended Starting IPX203 LD Dosing Regimen Based on the Dosing Regimen of IR CD-LD at the End of the Dose Adjustment Period

Most Frequent IR LD Unit Dose (mg)	Recommended Starting IPX203 Daily Dosing Regimen LD (mg) Every 8 Hours
100	280 mg (2 × 140 mg)
125 − 150	420 mg (3 × 140 mg)
175 − 200	560 mg (4 × 140 mg)
225 − 250	700 mg (5 × 140 mg)
≥ 275	840 mg (6 × 140 mg)

Any changes to the dosing regimen should be made by the Investigator or qualified site personnel.

When two or more IR LD doses correspond to the most frequent IR CD-LD dose, the suggested IPX203 conversion should correspond to the higher of the IR LD doses.

6.4.3. Double-Blind Maintenance Period

During the 13-week double-blind double-dummy maintenance period, subjects receive either IPX203 (with matching IR CD-LD placebo) or IR CD-LD (with matching IPX203 placebo) with the stable dosing regimen established at the end of Week 3 (Visit 2) for IR CD-LD and at the end of Week 7 (Visit 4) for IPX203.

6.5. Criteria for Study Termination

The Sponsor has the right to terminate this study and remove all study material from the study site at any time for medical or administrative reasons. The Sponsor will endeavor to give adequate notice to allow safe withdrawal of subjects from the study.

7. SELECTION AND WITHDRAWAL OF SUBJECTS

Each subject must meet all of the following inclusion and exclusion criteria to qualify for enrollment.

7.1. Subject Inclusion Criteria

1. Male or female subjects diagnosed at age ≥ 40 years with PD, consistent with the United Kingdom Parkinson's Disease Society Brain Bank Diagnostic Criteria (Appendix B) and who are being treated with stable regimens of CD-LD but experiencing motor fluctuations.
2. Able to provide written informed consent prior to the conduct of any study-specific procedures.
3. Female subjects of childbearing potential must have a negative urine pregnancy test at Screening Visit.
4. Negative urine screen for drugs of abuse and negative alcohol breath test at Screening.
5. Hoehn and Yahr Stages 1, 2, 3, or 4 in the "On" state (part of Movement Disorders Society version of the Unified Parkinson's Disease Rating Scale [MDS-UPDRS] Part III; Appendix D).
6. Agrees to use a medically acceptable method of contraception throughout the study and for 6 weeks after completing the study. Medically acceptable methods of contraception that may be used by the subject and/or partner include but are not limited to: abstinence, oral contraception, NuvaRing or transdermal systems, diaphragm with vaginal spermicide, intrauterine device, condom and partner using vaginal spermicide, surgical sterilization (6 months), progestin implant or injection, or postmenopausal female (no menstrual period for > 2 years) or vasectomy (> 6 months).
7. Montreal Cognitive Assessment (MoCA) score ≥ 24 at Screening Visit in "On" state (Appendix C).
8. By history, for the 4 weeks prior to Screening, the subject experiences daily "wearing-off" episodes with periods of bradykinesia in combination with at least one of rest tremor or rigidity, experiences an "Off" state upon awakening on most mornings, and reports an average of at least 2.5 cumulative hours per day of "Off" time during the waking hours.
9. Able to differentiate "On" state from "Off" state as determined by at least 75% concordance with a trained rater in "On/Off" ratings for 8 ratings over the 4-hour training period. The concordance must include at least 1 "On" and 1 "Off" rating and must be achieved within two 4-hour training sessions.
10. At Visit 1, review of the 3-day PD Diaries confirms the following: that the subject is able to properly complete the diaries with valid entries; and that the subject has an <u>average of at least 2.5 hours</u> per day of "Off" time during the waking hours over the 3 days with at least 1.5 hours of cumulative "Off" time on each day. Inability to properly complete the

diaries is indicated when more than 1 day of a diary is not returned or when more than 2 hours (4 half-hour periods) of one 24-hour diary day are missing (Appendix O).

11. Responsive to CD-LD therapy and currently being treated on a stable regimen with CD-LD for at least 4 weeks prior to Visit 1 and:
    a. Requires at least 100 mg of IR CD-LD for the first morning dose
    b. Requires a total daily dose of at least 300 mg of LD and takes a maximum total daily dose of 2400 mg LD, comprising IR CD-LD alone or IR CD-LD in combination with a single daily bedtime dose of CR CD-LD
    c. Has a dosing frequency of 3 to 9 times daily of CD-LD.
    d. By history, typically experiences an “On” response with the first dose of IR CD-LD of the day but the efficacy of this dose typically lasts less than 4 hours.
12. At Screening, the subject has predictable “Off” periods defined by a score of 1 or 2 on Item #4.5 (Complexity of Motor Fluctuations) of the MDS-UPDRS Part IV B (Motor Fluctuations) (Appendix D).
13. At Screening, the MDS-UPDRS Part III total score in the “Off” state is at least 20 units.
14. Able and willing to comply with the protocol, including completion of diaries and availability for all study visits.

7.2. Subject Exclusion Criteria

1. Used any doses of controlled-release (CR) CD-LD apart from a single daily bedtime dose within 4 weeks prior to Visit 1.
2. Used any doses of Rytary for the past 4 weeks prior to Visit 1 or considered IPX066 or Rytary failures for reasons of efficacy or safety.
3. Received any investigational medications within 30 days or 5 times the half-life, whichever is longer, prior to Visit 1.
4. Female subjects who are currently breastfeeding or lactating.
5. Had prior neurosurgical treatment for PD or if such procedure is planned or anticipated during the study period.
6. Allergic to any excipient in the study drugs (See Appendix P).
7. History of medical conditions or of a prior surgical procedure that would interfere with LD absorption, such as gastrectomy, proximal small-bowel resection, or bariatric surgery.
8. History of upper gastrointestinal hemorrhage in patients with peptic ulcer disease within the past 5 years.
9. History of glaucoma with intraocular pressures that are elevated despite appropriate medical management.
10. History of seizure or epilepsy and experienced at least 1 seizure during the past 12 months or has not been compliant with medically recommended therapy or visits.
11. History of myocardial infarction with residual atrial, nodal, or ventricular arrhythmias that are not controlled with medical and/or surgical interventions. A recent

(≤ 12 months) history of myocardial infarction with secondary arrhythmias is exclusionary regardless of the therapeutic control.

12. History of neuroleptic malignant syndrome or of nontraumatic rhabdomyolysis.
13. Liver enzyme values ≥ 2.5 times the upper limit of normal; or history of severe hepatic impairment.
14. Serum creatinine level ≥ 1.75 times the upper limit of normal; or requires dialysis at the time of Screening.
15. Subject with a history of malignant melanoma or with a suspicious undiagnosed skin lesion which in the opinion of the investigator could be melanoma.
16. History of drug or alcohol abuse within the 12 months prior to Screening.
17. Received within 4 weeks of Visit 1 or planning to take during participation in the clinical study:
    a. any doses of a controlled-release (CR) LD apart from a single daily bedtime dose, any doses of Rytary, additional CD (eg, Lodosyn) or benserazide (eg, Serazide), or catechol-O-methyl transferase inhibitors (entacapone or tolcapone) or medications containing these inhibitors (Stalevo),
    b. nonselective monoamine oxidase inhibitors (MAOI), apomorphine, or antidopaminergic agents, including antiemetics.
18. Treatment with any dopamine antagonist for the purposes of treating psychosis or bipolar disorder within the last 2 years or any history of psychosis within the past 10 years regardless of treatment. Mild PD-associated illusions are not exclusionary provided that they do not occur more than twice per week and the subject does not lose insight.
19. Employees or family members of the investigator, study site, or sponsor.
20. Subjects who have previously participated in an IPX203 study.
21. Subjects who, in the opinion of the clinical investigator, should not participate in the study.
22. Based on clinical assessment, subject does not adequately comprehend the terminology needed to complete the PD diary.

7.3. Subject Withdrawal Criteria

Site personnel should make every effort to conduct all protocol-specific procedures to complete the study. A subject may be discontinued from the study due to the following reasons:

1. Withdrawal by subject
2. Adverse event (AE)
3. Lack of efficacy
4. Study terminated by Sponsor
5. Protocol deviation

6. Noncompliance with study drug
7. Lost to follow-up
8. Death
9. Other

Subjects who withdraw early from the study will not be replaced. The reason or reasons for discontinuation will be specified and documented. Empty medication bottles and any unused study drug upon discontinuation will be collected. Study medication dispensed to a discontinued subject may not be redispensed to a different subject.

8. STUDY PROCEDURES

The procedures to be performed at each study visit are described below and summarized in Table 4.

Table 4: Events Schedule for Impax Study IPX203-B16-02

		3 Weeks of IR CD-LD Dose Adjustment	4 Weeks of IPX203 Dose Conversion			13 Weeks of Double-Blind Therapy		
**Assessment**	**Screening**	**Visit 1**	**Visit 2**	**Visit 3**	**Visit 4 Randomization**	**Visit 5**	**Visit 6**	**Visit 7 / Study Exit/Early Termination[b]**
Study Week[a]	**–4**	**0**	**3**	**5**	**7**	**10**	**15**	**20**
ICF & HIPAA Authorization[c]	X							
Contact IWRS	X	X	X	X	X	X	X	X
Randomization					X			
Inclusion/Exclusion	X	X						
Medical History	X							
Physical Examination	X							X
Vital Signs[d]	X	X	X	X	X	X	X	X
Height and Weight	X					X[e]		X[e]
C-SSRS[f]	X	X	X	X	X	X	X	X
Clinical Laboratory Tests[g]	X					X		X
Urine Pregnancy Test	X							
Urine Screen for Drug Abuse	X							
Alcohol Breath Test	X							
ECG	X					X		X
MoCA[h]	X							
MDS-UPDRS Parts I-IV[i]	X	X	X		X	X	X	X
PGI-C[j]						X	X	X
CGI-C[k]						X	X	X
PGI-S[l]		X			X			X

Assessment	Screening	3 Weeks of IR CD-LD Dose Adjustment	4 Weeks of IPX203 Dose Conversion			13 Weeks of Double-Blind Therapy		
		Visit 1	Visit 2	Visit 3	Visit 4 Randomization	Visit 5	Visit 6	Visit 7 / Study Exit/Early Termination[b]
Study Week[a]	–4	0	3	5	7	10	15	20
CGI-S[m]		X			X			X
PDQ-39[n]		X			X		X	X
GCSI[o]		X						X
NMSS[p]		X			X		X	X
PDSS-2[q]		X			X		X	X
PAS[r]		X			X		X	X
PD Diary Training; Perform Concordance Testing at Screening Only[s]	X	X	X	X	X	X	X	
Dispense PD Diaries[t]	X	X		X	X	X	X	
Review PD Diaries[u]		X	X		X	X	X	X
Reminder phone calls[v,w]	X[v]	X[w]	X[w]	X[w]	X[w]	X	X	X
Dispense study medication		X	X	X	X	X	X	
Collect empty medication bottles and any unused study drug/Perform study drug accountability			X	X	X	X	X	X
Adverse Events	X	X	X	X	X	X	X	X
Concomitant Medications	X	X	X	X	X	X	X	X

CGI-C = Clinical Global Impression of Change, CGI-S = Clinical Global Impression of Severity, C-SSRS = Columbia-Suicide Severity Rating Scale, ECG = electrocardiogram, GCSI = Gastroparesis Cardinal Symptom Index, HIPAA = Health Insurance Portability and Accountability Act, ICF = informed consent form, IWRS = interactive web response system, MDS-UPDRS = MDS version of Unified Parkinson's Disease Rating Scale, MoCA = Montreal Cognitive Assessment, PAS = Parkinson Anxiety Scale, PD = Parkinson's disease, NMSS = Non-Motor Symptom assessment scale for PD, PDQ-39 = 39-Item Parkinson's Disease Questionnaire, PDSS-2 = Parkinson's Disease Sleep Scale-2, PGI-C = Patient Global Impression of Change, PGI-S = Patient Global Impressions of Severity.

[a] The interval between Screening and Visit 1 (Day 1) should not exceed 4 weeks. Study visits should occur within ± 3 days of their specified timing.
[b] Study Exit procedures to be conducted at the end of Visit 7 or during an early termination visit.
[c] Subjects enrolled at sites in the United States (US) must sign HIPAA authorization prior to the conduct of any study-specific procedures.
[d] Record vital signs (blood pressure, heart rate, respiratory rate, and temperature [Screening and Study Exit only]) after subject has been resting supine for at least 5 minutes, then record orthostatic blood pressure and heart rate after subject has been standing for approximately 2 minutes.
[e] Weight only.
[f] C-SSRS: Columbia Suicide Severity Rating Scale. See Appendix N.
[g] See Appendix Q.
[h] Montreal Cognitive Assessment in the "On" state: see Appendix C.
[i] MDS-UPDRS Parts I through IV in the "On" and "Off" state (see Appendix D).
[j] See Appendix E.
[k] See Appendix F.
[l] See Appendix G.
[m] See Appendix H.
[n] See Appendix I.
[o] See Appendix J.
[p] See Appendix K.
[q] See Appendix L.
[r] See Appendix M.
[s] Train at Screening and then as needed at subsequent visits. Perform concordance testing at Screening.
[t] Dispense PD Diaries at Screening and Visits 1, 3, 4, 5, and 6. Call subjects 4 days prior to Visits 1, 2 and 4-7 to remind them to complete PD Diaries. Subjects record diary information for 3 consecutive days prior to each of the visits (Days -3, -2, and -1). Call subjects the day prior to each visit to remind them to bring the PD Diaries, empty medication bottles, and any unused study drug to the office.
[u] Review PD Diaries at Visits 1, 2, and 4-7.
[v] Post-Screening reminder phone call: Notify individuals who successfully complete screening procedures following review of all study entry criteria and clinical laboratory results that they may continue in the study. The interval between Screening and Visit 1 should not exceed 4 weeks.
[w] Reminder phone calls for Visits 1 through 4: In addition to the calls discussed above, make regular phone calls (approximately every 1 to 3 days) to subjects throughout the IR CD-LD dose-adjustment and IPX203 dose-conversion periods to evaluate each subject's adjustment to the study medication regimen.

8.1. Screening Visit

After the subject has signed the informed consent (and HIPAA authorization for US subjects only), complete the following procedures and assessments:

- Obtain an identification number from the Interactive Web Response System (IWRS). The IWRS will assign a 6-digit ID number to each subject, consisting of a 3-digit number representing the investigative site and a 3-digit sequential subject number.
- Review and record study entry criteria (Section 7).
- Perform urine pregnancy test for females of childbearing potential.
- Perform urine screen for drugs of abuse.
- Perform alcohol breath test.
- Complete medical history.
- Perform physical examination, including height and weight.
- Assess vital signs after subject is supine for at least 5 minutes (blood pressure, heart rate, temperature and respiratory rate) and then assess orthostatic blood pressure and heart rate after subject is standing (for approximately 2 minutes).
- Record current CD-LD regimen, other PD medications and their dosing schedule, and other concomitant medications.
- Record AEs.
- Perform a 12-lead ECG.
- Administer C-SSRS (Appendix N).
- Determine MoCA Score in the “On” state (Appendix C).
- Collect blood and urine samples for clinical laboratory studies (Appendix Q).
- Determine Hoehn and Yahr staging of PD in the “On” state (part of MDS-UPDRS Part III Motor Examination) (Appendix D).
- Administer MDS-UPDRS Parts I through IV in the “On” and “Off” state (Appendix D).
- Train the subject how to complete the PD Diaries to assess his/her “On” and “Off” states, including assessment of any dyskinesia. After training the subject, perform the concordance testing. The subject’s “On”/”Off” ratings must agree at least 75% of the time with the trained rater during the training sessions. That is, the subject’s “On”/”Off” ratings must agree with the trained rater’s ratings on at least 75% “On”/“Off” states in a single session to qualify for study inclusion. The 75% concordance rate must be based on at least four ratings, and must include at least one “On” and one “Off” state. The ratings should occur every 30 minutes and each session can last up to 4 hours. If the subject fails the first training session, the subject may be trained for one additional 4-hour training session.

- Dispense PD Diaries and instruct the subject to complete the PD Diaries on 3 consecutive days immediately prior to Visit 1.

Notify individuals who successfully complete screening procedures following review of all study entry criteria and clinical laboratory results.

The interval between Screening and Visit 1 (Day 1) should not exceed 4 weeks.

8.2. Visit 1 – Start of IR CD-LD Dose Adjustment

8.2.1. Prior to Visit 1

Contact the subject at least 4 days prior to Visit 1 to remind him/her to complete the 3-day PD Diaries starting 3 consecutive days immediately prior to Visit 1.

The day prior to Visit 1, remind subjects to:

- Bring their completed 3-day PD Diaries to the clinic.

8.2.2. At Visit 1

For Visit 1 complete the following procedures:

- Collect and review the subject's 3-day PD Diaries.
- Review inclusion and exclusion criteria to ensure that the subject continues to meet study inclusion criteria.
- Review instruction of 3-day PD Diaries if needed.
- Measure vital signs (respiratory rate, blood pressure, and heart rate after supine for at least 5 minutes, and orthostatic pulse and blood pressure after standing for approximately 2 minutes).
- Administer C-SSRS (Appendix N).
- Administer MDS-UPDRS Parts I through IV (Appendix D).
- Complete PDQ-39 (Appendix I).
- Complete PGI-S (Appendix G).
- Complete CGI-S (Appendix H).
- Complete GCSI (Appendix J).
- Complete Non-motor Symptom assessment scale for PD (NMSS) (Appendix K).
- Complete the Parkinson's Disease Sleep Scale-2 (PDSS-2) (Appendix L).
- Complete Parkinson Anxiety Scale (PAS) (Appendix M).
- Record any AEs and update changes in concomitant medication since the previous visit.
- Dispense PD Diaries.

- Contact IWRS and dispense study medication per IWRS instructions.

8.2.3. Post Visit 1

- Make regular phone calls (approximately every 1 to 3 days) while the IR CD-LD dose is being adjusted. The IR CD-LD dosing regimen should be stable for at least 5 days prior to returning for Visit 2.

8.3. Visits 2 (Week 3) and Visit 3 (Week 5) – IPX203 Dose Conversion

8.3.1. Prior to Visit 2

- Call subjects 4 days prior to Visit 2 and remind them to complete their PD Diaries.
- Call subjects the day prior to Visit 2 to remind them to bring the PD Diaries, empty medication bottles, and any unused study drug to the office.

8.3.2. Prior to Visit 3

- Call subjects the day prior to Visit 3 to remind them to bring back empty medication bottles and any unused study drug to the office.

8.3.3. At Visits 2 and 3

For Visits 2 and 3 complete the following procedures:

- Measure vital signs (respiratory rate, blood pressure, and heart rate after supine for at least 5 minutes, and orthostatic pulse and blood pressure after standing for approximately 2 minutes).
- Administer C-SSRS (Appendix N).
- Collect empty medication bottles and any unused study drug; perform study drug accountability.
- Record and update AEs and concomitant medications.
- Contact IWRS and dispense study medication per IWRS instructions.

Additional Assessments at Visit 2 Only

- Administer MDS-UPDRS Parts I through IV (Appendix D).
- Review PD Diaries. The subject will be terminated from the study if the subject does not average at least 2.5 hours per day of “Off” time over 3 days and at least 1.5 hours of “Off” time on each day based on the 3 day PD Diaries and/or if the subject cannot properly complete the diary, eg, if more than 1 day of the diary is not returned or if more than 1 day of the diary is not valid (ie, more than 2 hours [4 half periods] of the 24 hour diary are missing).
- Ensure that the subject has been on a stable dosing regimen for at least 5 days prior to dose conversion to IPX203. If the subject is not able to achieve a stable dosing regimen lasting at least 5 days, the subject will be discontinued.

- Conduct PD Diaries training, if needed.

Additional Activities at Visit 3 Only

- Dispense PD Diaries.
- Review instruction of 3-day PD Diaries if needed.

8.3.4. Post Visits 2 and 3

Make regular phone calls (approximately every 1 to 3 days) to subjects throughout the dose-conversion period, as needed, to evaluate each subject's adjustment to the study medication. The IPX203 dosing regimen should be stable for at least 5 days prior to returning for Visit 4.

8.4. Visit 4 (Week 7) – Randomization

8.4.1. Prior to Visit 4

- Call subjects 4 days prior to Visit 4 to remind them to complete their PD Diaries.
- Contact subjects 1 day prior to Visit 4 to remind them to bring back the PD Diaries, empty medication bottles, and any unused study drug to the office.

8.4.2. At Visit 4

For Visit 4 complete the following procedures:

- Review PD Diaries. The subject will be terminated from the study if the subject cannot properly complete the diary, eg, if more than 1 day of the diary is not returned or if more than 1 day of the diary is not valid (ie, more than 2 hours [4 half periods] of the 24 hour diary are missing).
- Ensure that the subject has been on a stable dosing regimen for at least 5 days prior to randomization. If the subject is not able to achieve a stable dosing regimen lasting at least 5 days, the subject will be discontinued.
- Measure vital signs (respiratory rate, blood pressure, and heart rate after supine for at least 5 minutes, and orthostatic pulse and blood pressure after standing for approximately 2 minutes).
- Administer C-SSRS (Appendix N).
- Administer MDS-UPDRS Parts I through IV (Appendix D).
- Complete PDQ-39 (Appendix I).
- Complete PGI-S (Appendix G).
- Complete CGI-S (Appendix H).
- Complete NMSS (Appendix K).
- Complete PDSS-2 (Appendix L).
- Complete PAS (Appendix M).

- Record and update AEs and concomitant medications.
- Conduct PD diaries training, if needed.
- Collect empty medication bottles and any unused study drug; perform study drug accountability.
- Contact IWRS for randomization and dispense medication per IWRS instructions.
- Dispense PD diaries.

8.5. Visit 5 (Week 10) and Visit 6 (Week 15)

8.5.1. Prior to Visit 5 and 6

- Call subjects 4 days prior to Visits 5 and 6 to remind them to begin recording in their PD diaries on each of the 3 consecutive days immediately prior to each of these visits.
- Call the subjects the day prior to Visits 5 and 6 to remind the subjects to bring in their PD diaries, empty medication bottles, and any unused study drug to the office.

8.5.2. At Visit 5 and 6

For Visits 5 and 6 complete the following procedures (note visit-specific tasks below):

- Measure vital signs (respiratory rate, blood pressure, and heart rate after supine for at least 5 minutes, and orthostatic pulse and blood pressure after standing for approximately 2 minutes).
- Administer C-SSRS (Appendix N).
- Administer MDS-UPDRS Parts I through IV (Appendix D).
- Complete PGI-C (Appendix E).
- Complete CGI-C (Appendix F).
- Review PD diaries.
- Conduct PD diaries training, if needed.
- Dispense PD diaries.
- Collect empty medication bottles and any unused study drug; perform study drug accountability.
- Contact IWRS and dispense study medication per IWRS instructions.
- Record any AEs and update changes in concomitant medication since the previous visit.

<u>Additional Activities at Visit 5 Only:</u>

- Record weight.
- Perform a 12-lead ECG.

- Collect blood and urine samples for clinical laboratory studies (Appendix Q).

<u>Additional Activities at Visit 6 Only:</u>

- Complete PDQ-39 questionnaire (Appendix I).
- Complete NMSS (Appendix K).
- Complete the PDSS-2 (Appendix L).
- Complete PAS (Appendix M).

8.6. Visit 7 (Week 20) – End of Study/Study Exit

8.6.1. Prior to Visit 7

- Call subjects 4 days prior to Visit 7 to remind them to begin recording in their PD Diaries on each of the 3 consecutive days immediately prior to Visit 7.
- Call the subjects the day prior to Visit 7 to remind the subjects to bring in their PD Diaries, empty medication bottles, and any unused study drug to the office.

8.6.2. At Visit 7

All enrolled subjects must complete Study Exit procedures at the end of Visit 7 or during an early termination visit:

- Perform physical examination, including weight.
- Measure vital signs (temperature, respiratory rate, blood pressure, and heart rate after supine for at least 5 minutes, and orthostatic pulse and blood pressure after standing for approximately 2 minutes).
- Collect and review 3-day PD Diaries.
- Administer C-SSRS (Appendix N).
- Administer MDS-UPDRS Parts I through IV (Appendix D).
- Complete PGI-C (Appendix E).
- Complete CGI-C (Appendix F).
- Complete PDQ-39 questionnaire (Appendix I).
- Complete NMSS (Appendix K).
- Complete PDSS-2 (Appendix L).
- Complete PAS (Appendix M).
- Complete PGI-S (Appendix G).
- Complete CGI-S (Appendix H).
- Complete GCSI (Appendix J).

- Collect empty medication bottles and any unused study drug; perform study drug accountability.
- Record and update AEs and concomitant medications.
- Collect blood and urine samples for clinical laboratory studies (Appendix Q).
- Perform a 12-lead ECG.
- Contact IWRS to record subject disposition.

8.7. Early Termination

8.7.1. Subjects who Terminate Prior to Randomization

If the subject discontinues the study prior to randomization (Visit 4) the subject should complete the following assessments:

- Perform physical examination, including weight.
- Measure vital signs (temperature, respiratory rate, blood pressure, and heart rate after supine for at least 5 minutes, and orthostatic pulse and blood pressure after standing for approximately 2 minutes).
- Collect and review 3-day PD Diaries when available.
- Administer C-SSRS (Appendix N).
- Administer MDS-UPDRS Parts I through IV (Appendix D).
- Collect empty medication bottles and any unused study drug; perform study drug accountability.
- Record and update AEs and concomitant medications.
- Collect blood and urine samples for clinical laboratory studies (Appendix Q).
- Perform a 12-lead ECG.
- Contact IWRS to record subject disposition.

8.7.2. Subjects who Terminate Early after Randomization

If the subject discontinues the study after randomization (Visit 4), the subject should complete all assessments described in Section 8.6.2.

8.8. Blood Volume

Safety blood draws: Approximately 10 mL of blood will be drawn at Screening, Visit 5, and at Study Exit, for a combined total of 30 mL.

9. TREATMENT OF SUBJECTS

9.1. Description of Study Drug

Study drugs will be provided by Impax for this study:

- IPX203 (carbidopa-levodopa) Extended-Release Capsules containing 35-140 mg of CD-LD for oral administration. The CD-LD ratio is 1:4. In addition, matching placebo capsules will also be provided.
- Immediate-release carbidopa-levodopa (IR CD-LD) tablet containing 25-100 mg of CD-LD, for oral administration. In addition, matching placebo tablets will also be provided.

Table 5: Study Drugs for Study IPX203-B16-02

Investigational Product	Dosage Strength and Form (mg CD-LD)	Manufacturer
IPX203 (carbidopa-levodopa) Extended-Release capsules	35-140 mg<br>Capsules for oral administration	Impax
IR CD-LD (carbidopa-levodopa) tablets	25-100 mg<br>Tablets for oral administration	Mylan
IPX203 Placebo capsules	Capsules for oral administration	Impax
IR CD-LD Placebo tablets	Tablets for oral administration	Impax

9.2. Concomitant Medications

9.2.1. Permitted PD Medications

Concomitant therapy with amantadine, selective monoamine oxidase (MAO) type B inhibitors (eg, selegiline, rasagiline), anticholinergic PD medications (eg, benztropine, trihexyphenidyl), and/or dopamine agonists (except apomorphine) is allowed provided the doses and regimens have been stable for at least 4 weeks prior to Visit 1 and the therapy is intended to be constant throughout the course of the study.

9.2.2. Prohibited Medications and Procedures

Prohibited medications and procedures include the following:

- Received within 4 weeks of Visit 1 or planning to take during participation in the clinical study: any doses of a controlled-release (CR) CD-LD apart from a single daily bedtime dose or any doses of Rytary, additional CD (eg, Lodosyn) or benserazide (eg, Serazide), or catechol-O-methyl transferase inhibitors (entacapone or tolcapone) or medications containing these inhibitors (Stalevo).

- Received within 4 weeks of Visit 1 or planning to take during participation in the clinical study: nonselective MAOI, selective MAO type A inhibitor (eg, phenelzine, moclobemide, pirilindole, bifemelane, toloxatone), apomorphine, or antidopaminergic agents including antiemetics.
- Treatment with any dopamine antagonist antipsychotic agents for the purposes of psychosis or bipolar disorder within the last 2 years. Use of antipsychotics to treat conditions other than psychosis or bipolar disorders may be allowed only after consultation with the medical monitor.
- Any neurosurgical procedure for the treatment of PD during the course of the study.

A subject who reports the use of any prohibited medications or procedure will be discontinued.

All medications taken within 30 days prior to signing the informed consent form (ICF) and all concomitant medications taken during the study will be recorded on the case report form (CRF).

9.3. Treatment Compliance

Study drug accountability and reconciliation will be performed by the study staff and the study monitor(s).

9.4. Randomization and Blinding

At Visit 4, subjects will be randomized, stratified by center, in a 1:1 ratio into one of two double-blind parallel treatment arms of IPX203 (and matching IR CD-LD placebo) or IR CD-LD (and matching IPX203 placebo).

10. STUDY DRUG MATERIALS AND MANAGEMENT

10.1. Study Drug

Study drugs include the investigational treatment IPX203 35-140 mg (and matching placebo capsules) and the active comparator treatment IR CD-LD (and matching placebo tablets).

IPX203 is an extended-release (ER) capsule formulation of CD-LD. Impax will manufacture and provide the IPX203 and matching placebo.

IR CD-LD is commercially available and will be provided by Impax. Matching placebo tablets will be manufactured and provided by Impax.

10.2. Study Drug Packaging and Labeling

Impax or designee will provide study medications in bottles with appropriate labeling affixed.

Labels on the study medication may include the following information:

- name, address, and phone number of the sponsor
- pharmaceutical dosage form/route of administration, quantity of dosage units, the name/identifier, and strength/potency
- batch and/or code number to identify the contents and packaging operation
- trial reference code (protocol number)
- trial subject identification number/treatment number and where relevant, the visit number
- name of investigator
- directions for use: Take tablet(s) or capsule(s) orally with water as directed.
- for clinical trial use only
- storage information: Store at 25°C (77°F), with excursions permitted to 15°C to 30°C (59°F to 86°F). Protect from light and moisture.
- period of use (use-by date, expiry date or retest date as applicable), in month/year format and in a manner that avoids any ambiguity.
- keep out of reach of children
- caution statement: Caution: New Drug—Limited by Federal (or United States) law to investigational use.

10.3. Study Drug Storage

The clinical site should store the study drug at 25°C (77°F), with excursions permitted to 15°C to 30°C (59°F to 86°F). The study drug should be stored in a tightly closed container, protected from light and moisture. Storage temperature excursions above 30°C (86°F) should be reported by the clinical site to Impax or its designee.

10.4. Study Drug Administration

Subjects will be instructed to take their medications with approximately 240 mL of room-temperature water. The capsules or tablets should not be crushed or chewed.

IR CD-LD tablets may be split to achieve the required doses.

10.5. Study Drug Dispensing and Accountability

The Investigator must ensure that all study medication received at the study site is inventoried and accounted for, and that dispensed study medication is recorded in the subject's source documents, the CRF, and the study medication inventory log. Site personnel must not relabel or reassign study medication to other subjects or to individuals not enrolled in the study. The study monitor verifies medication accountability during monitoring visits.

10.6. Study Drug Handling and Disposal

The Investigator must retain and properly store all partially used and unused study medication until authorized by Impax regarding disposition.

11. ASSESSMENT OF EFFICACY

11.1. Parkinson’s Disease Diary

Subjects are to record “asleep,” “Off,” and “On” without or with (nontroublesome or troublesome) dyskinesias during waking hours every 30 minutes over a 24-hour day. In the PD Diaries, subjects are instructed to indicate for each half-hour their predominant state during most of that period. “Off” is defined as the typical functional state when the medication is no longer providing benefit with regard to mobility, slowness, and stiffness in spite of taking medications. “On” is defined as the typical functional state when a subject has received medication and the medication is providing benefit with regard to mobility, slowness, and stiffness. Dyskinesias are defined as involuntary and irregular twisting and/or turning movements. Dyskinesia movements are usually an effect of medication and occur during “On” time. Nontroublesome dyskinesias do not interfere with function or do not cause meaningful discomfort. Troublesome dyskinesias do interfere with function or do cause meaningful discomfort.

11.2. Patient and Investigator Global Assessments

- Patient Global Impression of Change (Appendix E): The patient will compare his/her condition from the start of the study on a 7-point scale ranging from “Very much worse” (1) to “Very much improved” (7) at the time of the assessment.
- Clinical Global Impression of Change (Appendix F): The clinician will compare the subjects’ condition from the start of the study on a 7-point scale ranging from “Very much worse” (1) to “Very much improved” (7) at the time of the assessment.
- Patient Global Impression of Severity (Appendix G): The patient will determine the severity of the disease on a 7-point scale ranging from “Normal, not at all ill” (1) to “Extremely severely ill” (7) at the time of the assessment.
- Clinical Global Impression of Severity (Appendix H): The clinician will determine the severity of the disease on a 7-point scale ranging from “Normal, not at all ill” (1) to “Among the most extremely ill of subjects” (7) at the time of the assessment.

11.3. Movement Disorders Society Version of Unified Parkinson’s Disease Rating Scale

The MDS-UPDRS has 4 parts:

- Part I: Non-Motor Aspects of Experiences of Daily Living (nM-EDL) has 2 components. Component IA contains a number of behaviors assessed by the investigator with all pertinent information from the patients and caregivers. Component IB is completed by the patient with or without help from the caregiver but independent of the investigator. These sections can be reviewed by the rater to ensure all questions are answered clearly and the rater can help explain any ambiguities.

- Part II: Motor Aspects of Experiences of Daily Living (M-EDL) is a self-administered questionnaire but can be reviewed by the investigator to ensure completeness and clarity.
- Part III: Motor Examination assesses the motor signs of PD and has instructions for the rater to give to or to demonstrate to the patient. It is completed by the rater.
- Part IV: Motor Complications integrates patient-derived information with the rater's clinical observations and judgements and is completed by the rater. It contains instructions for the rater and instructions to be read to the patient.

11.4. Additional Assessments

- Parkinson's Disease Questionnaire-39 (PDQ-39) is a self-reported questionnaire. Using the 39-items, 8 domains are defined: mobility (Questions 1-10), activities of daily living (ADL) (Questions 11-16), emotional well-being (Questions 17-22), stigma (Questions 23-26), social support (Questions 27-29), cognition (Questions 30-33), communication (Questions 34-36) and bodily discomfort (Questions 37-39).
- Non-Motor Symptom assessment scale for Parkinson's Disease (NMSS) is a 30-item investigator rated questionnaire. The NMSS contains 9 domains: cardiovascular (Questions 1, 2), sleep/fatigue (Questions 3-6), mood/cognition (Questions 7-12), perceptual problems (Questions 13-15), attention/memory (Questions 16-18), gastrointestinal (Questions 19-21), urinary (Questions 22-24), sexual function (Questions 25, 26), and miscellaneous (Questions 27-30).
- Parkinson's Disease Sleep Scale-2 (PDSS-2) is 15-item self-reported questionnaire. Three domains are defined: disturbed sleep (Questions 1-3, 8, 14), motor symptoms at night (Questions 4-6, 12, 13), PD symptoms at night (Questions 7, 9-11, 15).
- Parkinson Anxiety Scale (PAS) is a 12-item patient or observer rated questionnaire with 3 domains: persistent anxiety (Questions A.1-A.5), episodic anxiety (Questions B.1-B.4) and avoidance anxiety (Questions C.1-C.3).

12. ASSESSMENT OF SAFETY

12.1. Safety Parameters

Safety will be assessed by the following parameters:

- Electrocardiograms (ECGs), clinical laboratory tests, physical examinations, the Columbia-Suicide Severity Rating Scale (C-SSRS), and supine and standing orthostatic vital signs.
- Adverse events and concomitant medications will be evaluated throughout the course of the study.

12.2. Adverse Events

12.2.1. Definition of Adverse Event

An adverse event (adverse experience) is any untoward medical occurrence in a patient or clinical trial subject administered a medicinal product and which does not necessarily have to have a causal relationship with this treatment. An adverse event can therefore be any unfavorable and unintended sign (eg, an abnormal laboratory finding), symptom, or disease temporally associated with the use of a medicinal product, whether or not considered related to the medicinal product.

All AEs and any clinically significant physical examination findings, 12-lead ECG abnormalities, or clinical laboratory measurements occurring during the study that were not present prior to administration of study medication and that continue at Study Exit should be followed and evaluated with additional tests, if necessary, until the AEs are medically stable or resolved. Follow-up on these AEs should be recorded on the source documents and reported to Impax.

12.2.2. Recording Adverse Events

Elicit information about AEs with nonselective questions such as: “Have you experienced any changes in your health status since your last visit?” Encourage subjects to report AEs at onset.

Record information for any AE that emerges from the time the subject signs the ICF until Study Exit.

Monitor each subject closely for the development of AEs and record all such events on the AE page of the CRF. Whenever possible, group signs and symptoms that constitute a single diagnosis. For example, cough, rhinitis, and sneezing might be grouped as upper respiratory infection.

For each AE, record the onset date, severity, seriousness, relationship to study medication, date of resolution (or continuing), action taken, and outcome in the CRF. The Investigator is to make a causality assessment (relationship to study medication) for every AE.

12.2.3. Follow-up

The Investigator must follow each AE until resolved or medically stable.

12.2.4. Relationship to Study Drug

The Investigator documents his/her opinion of the relationship of the AE to the study medication as follows:

- Not Related—the experience can be readily explained by the subject's underlying medical condition or concomitant medications and no relationship exists between the study medication and the experience.
- Unlikely Related—the temporal relationship between the AE and the administration of the study medication is uncertain and it is likely that the AE can be explained by the subject's medical condition or other therapies.
- Possibly Related—there is some logical temporal relationship between the AE and the administration of the study medication and the experience is unlikely to be explained by the subject's medical condition or other therapies.
- Related—the temporal relationship is compelling between the administration of the study medication and the AE cannot be explained by the subject's medical condition or other therapies.

12.2.5. Assessment of Severity

Grade each AE for severity and note in the description of the AE. Determine the severity category of mild, moderate, or severe, as defined below, and enter the information on the AE page of the CRF.

- Mild—causing no limitation of usual activities
- Moderate—causing some limitation of usual activities
- Severe—causing inability to carry out usual activities

12.3. Serious Adverse Events

12.3.1. Definition of Serious Adverse Event

A serious adverse event (SAE) is any AE occurring at any dose that results in any of the following outcomes, regardless of relationship to the study medication:

- Death
- A life-threatening adverse drug experience
- Inpatient hospitalization or prolongation of existing hospitalization
- A persistent or significant disability/incapacity
- A congenital anomaly/birth defect

- Important medical events that may not result in death, be life-threatening, or require hospitalization may be considered an SAE when, based upon appropriate medical judgment, they may jeopardize the subject and may require medical or surgical intervention to prevent one of the outcomes listed in this definition.

12.3.2. Reporting Serious Adverse Events

Any SAE that occurs from the time the subject signs an ICF until 30 days after taking the final dose of study medication must be reported by the investigative staff to the Sponsor or the Sponsor's representative within 24 hours of knowledge of the event (see Study Contact Information).

An SAE form must be completed and sent to the Sponsor and/or the Sponsor's representative. All SAEs must also be recorded on the AE page of the CRF. Additionally, all SAEs must be reported to the institutional review board (IRB) per the IRB's requirements.

Those SAEs that are considered both serious and unexpected and related to the study drug are subject to expedited reporting. An "unexpected AE" is any AE where the nature or severity is not consistent with the current investigator brochure (IB) or if an IB is not required or available, the specificity or severity is not consistent with the provided risk information.

Unexpected fatal or life-threatening SAEs related to the study drug must be reported by the Sponsor to the appropriate regulatory authority in an expedited manner (ie, first report within 7 days of first knowledge by the Sponsor). The Sponsor will provide a final written report to that authority within 15 days of initial receipt of information on the event. The Sponsor or the Sponsor's representative will also inform all participating Investigators of the SAE.

Unexpected SAEs that are not fatal or life-threatening must be reported by the Sponsor to the appropriate regulatory authority as soon as possible but no later than 15 calendar days after first knowledge of the SAE by the Sponsor. The Sponsor or the Sponsor's representative also informs all participating Investigators of the SAE.

Subjects withdrawn from the study due to any SAE will be followed until the SAE is resolved or medically stable. Record all SAEs, regardless of severity and whether or not related to the study medication, on the appropriate page of the CRF.

The Investigator must determine whether the seriousness of the event warrants removal of the subject from the study. He/she should, in any case, institute appropriate diagnostic and therapeutic measures and keep the subject under observation for as long as is medically indicated, or refer the subject to appropriate health professionals.

12.4. Pregnancy

Any pregnancy that occurs from the time the subject signs an ICF until 30 days after taking the final dose of study medication must be reported within 24 hours to the Sponsor or the Sponsor's representative and the subject should be terminated from the study. All pregnancies will be followed through to delivery of the infant. If the subject experiences a termination of the pregnancy, it should be reported as defined in Section 12.3.2.

12.5. Other Safety Parameters and Related Information

Additional safety parameters (laboratory tests, 12-lead ECGs, physical examinations, and vital signs), the C-SSRS, the GCSI, and concomitant medications are collected as shown in the Schedule of Assessments in Table 4. Clinical laboratory assessments are listed in Appendix Q.

13. STATISTICS

13.1. Study Design and Sample Size Estimation

This is a multicenter, randomized, double-blind, double-dummy, active-controlled, parallel-group study. Assuming a difference of 1 hour between IPX203 and IR CD-LD in "Good on" time and a standard deviation of the treatment differences to be 3.0 hours, a sample size of 210 subjects per arm will be needed to ensure at least 90% power at a 0.05 significance level.

Assuming approximately an 18% prerandomization drop-out, approximately 510 subjects would need to be enrolled to randomize 420 subjects.

13.2. Demographics/Baseline Comparability

The demographics and baseline characteristics will be summarized by treatment arms and overall using descriptive statistics. Demographics information includes age, sex, and race. Baseline disease characteristics include MDS-UPDRS Parts I, II, III, and IV, Hoehn and Yahr stage, MoCA scores, and age of onset of PD. Distributions of dosing information, including LD doses and years on LD, will also be summarized.

13.3. Analysis of Efficacy Data

In order to control the type I error rate, the primary efficacy endpoint and key secondary efficacy endpoints will be tested in a single hierarchical order as details in Section 13.5.

13.3.1. Primary Efficacy Endpoint

The primary efficacy endpoint is the mean change from baseline in "Good on" time in hours per day, averaged over 3 PD Diary days, at the end of the double-blind therapy (Visit 7 or early termination). "Good on" time is derived from the 3-day PD Diaries. For each day, "Good on" time is calculated by adding the number of half-hour intervals in which either an "On without dyskinesia" or "On with nontroublesome dyskinesia" is checked.

The primary efficacy endpoint will be analyzed using a mixed effects for repeated measures (MMRM) model. The model will include baseline (Visit 4) "Good on" time as a covariate, treatment, visit, and pooled center as fixed effects, and a treatment-by-visit interaction. The model will employ an unstructured within subject covariance matrix and a restricted maximum likelihood (REML) estimation method. The primary analysis population will be the modified intent-to-treat as defined in Section 13.6. Missing data will be handled as in Section 13.7. Further details, including pooling centers algorithm, will be provided in the Statistical Analysis Plan (SAP).

13.3.2. Key Secondary Efficacy Endpoints

The first key secondary endpoint is the mean change from baseline in "Off" time in hours per day, averaged over 3 PD Diary days, at the end of the double-blind therapy (Visit 7 or early termination). "Off" time is derived from the 3-day PD Diaries. For each day, "Off" time is calculated by adding the number of half-hour intervals in which an "Off" is checked. This

endpoint will be analyzed using a MMRM model with baseline (Visit 4) “Off” time as a covariate, treatment, visit, and pooled center as fixed effects, and a treatment-by-visit interaction.

The proportion of subjects with either “much improved” or “very much improved” in PGI-C scores at the end of the double-blind therapy (Visit 7 or early termination), the second key secondary endpoint, will be analyzed using a Cochran-Mantel-Haenszel test to compare the two treatment groups with pooled center as a stratification factor.

The mean change from baseline in the number of motor fluctuations per day at the end of the double-blind therapy (Visit 7 or early termination) is the third key secondary endpoint. A “motor fluctuation” is defined as a change from “Off” to “On” state or from “On” to “Off” state during waking hours from the 3-day PD Diary. The number of “motor fluctuation” over the 3-day PD Diary will be divided by 3 to obtain the number of motor fluctuations per day. This endpoint will be analyzed using an MMRM model with baseline (Visit 4) number of motor fluctuations as a covariate, treatment, visit, and pooled center as fixed effects, and a treatment-by-visit interaction.

The mean change from baseline in MDS-UPDRS Parts II and III combined at the end of the double-blind therapy (Visit 7 or early termination) is the fourth key secondary endpoint. This endpoint will be analyzed using an MMRM model with baseline (Visit 4) MDS-UPDRS Parts II and III combined as covariates, treatment, visit, and pooled center as fixed effects, and a treatment-by-visit interaction.

For the 4 key secondary endpoints, the analysis population will be the modified intent-to-treat as defined in Section 13.6. Missing data will be handled as in Section 13.7. Further details of these analyses, including pooling centers algorithm, will be provided in the SAP.

13.3.3. Additional Efficacy Endpoints

Other additional efficacy endpoints collected postrandomization will be analyzed by visit in a fashion similar to the primary and key secondary endpoints. The changes from Visit 1 and Visit 4 will be evaluated where applicable, unless other specified:

- Percent “Off” time during waking hours derived from the 3-day PD Diaries
- Average time to “On” upon awakening
- Average duration of each continuous “Good on”
- Hours of (1) “Off” time (from Visit 1), (2) “Good on” time (from Visit 1), (3) “On” time with dyskinesia, (4) “On” time with troublesome dyskinesia, and (5) “On” time with nontroublesome dyskinesia, and (6) asleep time derived from the 3-day PD Diaries
- Proportion of subjects with an improvement in “Good on” time of at least 1, 1.5, 2, 2.5, and 3 hours
- Proportion of subjects with a reduction in “Off” time of at least 0.5, 1, 1.5, 2, 2.5, and 3 hours
- Proportions of subjects who are “On” upon awakening and “Good on” upon awakening

- MDS-UPDRS total score (sum of Parts I, II, III, and IV) and each part separately
- MDS-UPDRS Parts II and III combined (from Visit 1)
- MDS-UPDRS Part II Question 2.9
- PDQ-39 total score and individual domain scores
- NMSS total score and individual domains
- PDSS-2 total score and individual domains
- PDSS-2 items 9, 10, 11, 12, and 13 combined
- PAS total score and individual domains
- PGI-S
- Proportion of subjects with either “severely ill” or “extremely severely ill” on the PGI-S
- CGI-S
- Proportion of subjects with either “severely ill” or “among the most extremely ill of subjects” on the CGI-S
- PGI-C scores
- CGI-C scores
- Proportion of subjects with either “much improved” or “very much improved” on the CGI-C

Additionally the PGI-C and CGI-C will be analyzed using analysis of variance (ANOVA) with treatment and pooled center as factors.

In general, continuous endpoints will be summarized by standard descriptive statistics (mean, standard deviation, median, minimum, and maximum). Categorical endpoints will be summarized by frequencies and percentages. Comparisons between the two arms will be explored using appropriate statistical methodologies. Details will be provided in the SAP.

13.4. Sensitivity Analyses of the Primary Endpoint and Key Secondary Endpoints

Sensitivity analyses will be performed with respect to the primary efficacy endpoint as follows.

- The primary efficacy endpoint will also be analyzed using an ANCOVA model with “Good on” time at Visit 4 as a covariate, pooled center and treatment as factors. Missing data will be imputed using the last observation carried forward (LOCF) method.
- Pattern-mixture model: If an overall dropout rate postrandomization is > 15%, pattern-mixture models (PMM) will be employed to assess the robustness of the results under the MMRM model. The pattern for PMM is defined by patients’ last visit with an observed primary efficacy endpoint. The observed “Good on” time is

assumed to have a linear relationship with the patient's prior assessment. The missing values will be imputed multiple times (multiple imputation) under the assumption that the distribution of the missing values is the same as that of the observed values. After imputations, the dataset will be analyzed using an MMRM model similar to the primary analysis model. The results will then be combined using the Rubin's rule (Rubin 1987).

Similar analyses will be done for the key secondary endpoints ("Off" time, motor fluctuations, and MDS-UPDRS Parts II and III combined).

13.5. Multiplicity Adjustments

The primary endpoint and 4 key secondary endpoints will be tested in a sequential hierarchical order as follows.

1. The primary endpoint, the mean change from baseline in "Good on" time (hours per day), will be tested first at a 0.05 level of significance.
2. If statistical significance is demonstrated, then the first key secondary endpoint, the mean change from baseline in "Off" time (hours per day), will be tested next at a 0.05 level of significance.
3. If statistical significance is demonstrated, then the second key secondary endpoint, the proportion of subjects with either "much improved" or "very much improved" on the PGI-C, will be tested next at a 0.05 level of significance.
4. If statistical significance is demonstrated, then the third key secondary endpoint, the mean change from baseline in the motor fluctuations per day, will be tested at a 0.05 level of significance.
5. If statistical significance is demonstrated, then the fourth key secondary endpoint, the mean change from baseline in the sum of the MDS-UPDRS Parts II and III combined will be tested next at a 0.05 level of significance.

For the other efficacy endpoints, no adjustment will be made.

13.6. Analysis Populations

13.6.1. Safety Analysis Set

The Safety Analysis set will include all subjects who were treated with any study drug.

13.6.2. Intent-to-treat Analysis Set

The Intent-to-treat Analysis Set will include all subjects who were randomized and treated with any study drug and have a baseline and at least one postbaseline efficacy assessment.

13.6.3. Modified Intent-to-treat Analysis Set

The Modified Intent-to-treat Analysis Set will include all subjects who were randomized and treated and have a valid baseline PD Diary and at least one valid postrandomization PD Diary. This analysis set will be used for the primary analysis and key secondary analyses.

13.6.4. Completers Analysis Set

The Completers Analysis Set will include all subjects who were randomized and treated and complete the study.

13.7. Handling of Missing Data

13.7.1. Missing Data for PD Diaries

An MMRM approach will be used to handle missing visit data. MMRM analysis will use all available valid visit data, including subjects with some missing visit data, in order to arrive at an estimate of the mean treatment effect.

A PD Diary is valid if at least 2 days of diary data are available using the rules defined below. If 2 or more PD Diary days are missing in a particular visit, then that visit will be excluded from the analysis of the primary and key secondary endpoints.

Imputation of missing data for a PD Diary day will be required if a PD Diary is not completed for a full day (6 am to 5:30 am). In this case, the method of imputation will be dependent upon the amount and pattern of missing data:

1. If more than 4 half-hour time intervals are missing, then that particular day will not be included in the analysis.
2. If a one-half hour time interval is missing and the observations on either side of the time interval are not missing, then the missing time interval will be imputed by assigning a value of the previous measurement for the first 15 minutes and the value of the next measurement for the second 15 minutes.
3. If 2, 3, or 4 consecutive half-hour time intervals are missing, and these time intervals are available from other days of the visit then the following rules will be applied:
    a. For missing time intervals on Day 1, data from Day 2 will be used for imputation for the same time intervals. If Day 2 data is also incomplete, then Day 3 data will be used.
    b. For missing time intervals on Day 2, data from Day 3 will be used for imputation if available; otherwise Day 1 data will be used.
    c. Data from Day 2 will be used for imputing missing time intervals on Day 3. If data from Day 2 is not available, then Day 1 data will be used for imputation.
    d. If data at the same time period are missing across all days, then the approach will be to split the individual missing half-hour intervals into 2 periods, with the first-half interval being imputed with data from the immediate previous nonmissing time period and the second-half interval being imputed with the next nonmissing time interval.

13.7.2. Missing Data for Global Assessments (PGI-C, CGI-C, PGI-S and CGI-S)

For subjects with missing PGI-C or CGI-C for a particular visit, the data will be imputed as nonresponders (ie, not being “much improved” or “very much improved”).

For subjects with missing PGI-S or CGI-S for a particular visit, the data will be imputed as nonresponders (ie, being “severely ill” or “extremely severely ill” for PGI-S and being “severely ill” or “among the most extremely ill of subjects” for CGI-S).

13.7.3. Missing Data for MDS-UPDRS Parts II and III

If the MDS-UPDRS Parts II or III are missing for the particular visit, the missing data will be handled via the MMRM model.

If < 20% of the component questions are missing for a particular part of the MDS-UPDRS questionnaire, the missing items are assigned the average value for other items in that part:

- For Part II (13 questions): up to 2 missing questions will be imputed using the average value of the remaining 11 questions.
- For Part III (33 questions): up to 6 missing questions will be imputed using the average value of the remaining 27 questions.

If ≥ 20% of the component questions are missing for a particular part of the MDS-UPDRS questionnaire, the entire questionnaire will not be included in the analysis for that particular assessment. Missing data will be handled in a fashion similar to PD Diary data (Section 13.7.1) using the MMRM model.

For quality-of-life endpoints, missing responses within a questionnaire will not be imputed.

13.8. Analysis of Safety

The safety analysis will include all subjects who receive at least 1 dose of study medication. Reported AEs will be coded using the Medical Dictionary for Regulatory Activities (MedDRA). All AEs will be summarized by system organ class and preferred terms within system organ class. The severity, seriousness, and relationship to study medication will also be summarized by treatment arms. Each AE (based on preferred term) is counted once for a given subject. If the same AE occurred on multiple occasions, the highest severity and least complimentary relationship will be assumed.

The incidence of treatment-emergent AEs and serious AEs will be summarized by treatment arms.

Additionally, laboratory test data, physical examinations, vital signs, ECGs, C-SSRS, and GCSI will be summarized by treatment arms.

14. ADMINISTRATIVE PROCEDURES

14.1. Guidelines for Good Clinical Practice

This study will be conducted in accordance with principles of Good Clinical Practice (GCP) as promulgated by the ICH. Good Clinical Practice is an international ethical and scientific quality standard for designing, conducting, recording, and reporting trials that involve the participation of human subjects. Compliance with this standard provides public assurance that the rights, safety, and well-being of human subjects are protected under current ethical principles, and that the clinical trial data are credible. Current GCP standards may be found in ICH Guidance E6 (Good Clinical Practice: Consolidated Guidance). This guidance describes the principles of GCP and the obligations of the institutional review board (IRB), the Investigator and the Sponsor in conducting this study in accordance with those principles.

14.2. Institutional Review Board Approval

The review of this protocol by an IRB and the performance of all aspects of the study, including the methods used for obtaining informed consent, must be in accordance with principles enunciated in the ICH and GCP Guidelines and by the appropriate regulatory authorities.

The Investigator is responsible for preparing documents for submission to the relevant IRB and obtaining written approval for this study. Institutional Review Board approval must be obtained prior to the initiation of the study. The Investigator’s continued participation in the study is contingent on renewing approval with the IRB at least annually.

14.3. Informed Consent

Site personnel should prepare an Informed Consent Form (ICF) incorporating the necessary elements of consent. The ICF is to be approved by Impax prior to submission to the IRB. The Investigator or his/her staff must explain the nature of the investigation and the risks involved to each subject prior to screening, and obtain a signed ICF. The subject should also be informed that he/she is free to voluntarily withdraw from the study at any time.

14.4. Study Monitoring

Impax representatives or designees will conduct site visits to the investigational facilities for the purpose of monitoring the study. The Investigator agrees to allow the monitor to inspect the drug storage area, study drug stocks, drug accountability records, subject charts and study source documents, and other records relevant to study conduct. The Investigator must permit access to such records if a regulatory or compliance audit is required.

14.5. Protocol Amendments

All amendments to the protocol must be documented in writing, reviewed and approved by the Sponsor and Investigator, and submitted to the IRB for approval prior to implementation. If the protocol amendment substantially alters the study design or potential risk to the subject, a new

written ICF for continued participation in the study must be obtained from each subject affected by the change.

14.6. Termination of Study

The Sponsor has the right to terminate this study and remove all study material from the site at any time for medical or administrative reasons. In this event, the Sponsor will endeavor to give adequate notice to allow safe withdrawal of subjects from the study.

14.7. Case Report Forms

Site personnel should collect and record data for the study as source documents, and transfer the data into the CRF.

The Investigator must ensure that complete data for the clinical study are collected and accurately documented in the appropriate sections of the CRF and adequately supported by the appropriate source documentation. In addition, it is the Investigator’s responsibility to provide signatures where requested indicating concurrence with data in the CRF.

14.8. Investigator’s Final Conduct Report

At the completion of the study, the Investigator must provide Impax a copy of the final conduct report that was submitted to their IRB, including a review of AEs.

14.9. Records Retention

International Conference on Harmonization, GCP, and US FDA guidelines require that essential documents be retained until at least 2 years after the last approval of a marketing application and until there are no pending or contemplated marketing applications, or at least 2 years have elapsed since the formal discontinuation of clinical development of the investigational product.

However, the essential documents should be retained for a longer period if required by the applicable regulatory requirements or by an agreement with the Sponsor. Records should never be destroyed without written approval from the Sponsor.

If an Investigator leaves the institution, he/she must transfer responsibilities for record retention to another individual willing to accept them. The Investigator must notify the Sponsor in writing of the transfer of study documents before the transfer of the study documents.

15. PUBLICATION POLICY

Study results may not be published without prior written approval from Impax.

16. LIST OF REFERENCES

Blanchet PJ, Calon F, Martel JC, et al. Continuous administration decreases and pulsatile administration increases behavioral sensitivity to a novel dopamine D2 agonist (U-91356A) in MPTP-exposed monkeys. J Pharmacol Exp Ther. 1995;272:854-859.

Engber TM, Susel Z, Juncos JL, Chase TN. Continuous and intermittent levodopa differentially affect rotation induced by D-1 and D-2 dopamine agonists. European Journal of Pharmacology. 1989;168:291-298.

Fahn S. Parkinson disease, the effect of levodopa, and the ELLDOPA trial. Earlier vs Later L DOPA. Arch Neurol. 1999 May;56(5):529-35.

Food and Drug Administration 2003. Guidance for Industry. Bioavailability and Bioequivalence Studies for Orally Administered Drug Products — General Considerations. U.S. Department of Health and Human Services. Food and Drug Administration. Center for Drug Evaluation and Research (CDER). March 2003.

Juncos JL, Engber TM, Raisman R, et al. Continuous and intermittent levodopa differentially affect basal ganglia function. Ann Neurol. 1989;25:473-478.

Kurlan R, Rothfield KP, Woodward WR, Nutt JG, Miller C, Lichter D, et al. Erratic gastric emptying of levodopa may cause “random” fluctuations of parkinsonian mobility. Neurology. 1988;38(3):419-21.

Melamed E, Bitton V, Zelig O. Delayed onset of responses to single doses of L-dopa in parkinsonian fluctuators on long-term L-dopa therapy. Clin Neuropharmacol. 1986;9(2):182-8.

Mizuno Y. Where do we stand in the treatment of Parkinson's disease? Neurology. 2007;254:13-18.

Nilsson D, Nyholm D, Aquilonius S-M. Duodenal levodopa infusion in Parkinson's disease--long-term experience. Acta Neurologica Scandinavica. 2001;104:343-348.

Nyholm D, Nilsson Remahl AI, Dizdar N, et al. Duodenal levodopa infusion monotherapy vs oral polypharmacy in advanced Parkinson disease. Neurology. 2005;64:216-223.

Rubin DB. Multiple Imputation in Nonresponse in Surveys. San Francisco, CA: Wiley; 1987.

Sinemet (Carbidopa-Levodopa) Tablets Prescribing Information. Whitehouse Station, NJ: Manufactured by Merck & Co., Inc.; Marketed by Bristol-Myers Squibb Company; 2013.

Stocchi F, Quinn NP, Barbato L, Patsalos PN, O’Connel MT, Ruggieri S, et al. Comparison between a fast and a slow release preparation of levodopa and a combination of the two: a clinical and pharmacokinetic study. Clinical Neuropharmacol. 1994;17(1):38-44.

Stocchi F, Vacca L, et al. Intermittent vs continuous levodopa administration in patients with advanced Parkinson disease: a clinical and pharmacokinetic study. Arch Neurol. 2005:62(6):905-910.

17. APPENDICES

APPENDIX A. PRESCRIBING INFORMATION FOR IR CD-LD

CARBIDOPA AND LEVODOPA- carbidopa and levodopa tablet
Mylan Pharmaceuticals Inc.

DESCRIPTION

Carbidopa and Levodopa Tablets, USP are a combination of carbidopa and levodopa for the treatment of Parkinson's disease and syndrome.

Carbidopa USP, an inhibitor of aromatic amino acid decarboxylation, is a white to creamy white powder. It is slightly soluble in water; freely soluble in 3 N hydrochloric acid; slightly soluble in methanol; practically insoluble in acetone, chloroform, ether and methylene chloride, with a molecular weight of 244.2. It is designated chemically as (-)-L-α-Hydrazino-3,4-dihydroxy-α-methylhydrocinnamic acid monohydrate. Its molecular formula is $C_{10}H_{14}N_2O_4 \cdot H_2O$ and its structural formula is:

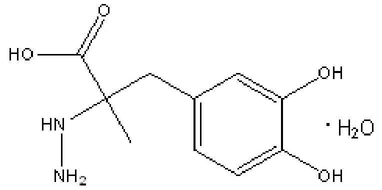

Tablet content is expressed in terms of anhydrous carbidopa which has a molecular weight of 226.2.

Levodopa USP, an aromatic amino acid, is a white to off-white crystalline powder. It is slightly soluble in water; freely soluble in 3 N hydrochloric acid; readily soluble in formic acid; practically insoluble in ethanol, benzene, chloroform and ethyl acetate; insoluble in acetone, acetic acid and methanol; with a molecular weight of 197.2. It is designated chemically as (-)-3-(3,4-Dihydroxyphenyl)-L-alanine. Its molecular formula is $C_9H_{11}NO_4$ and its structural formula is:

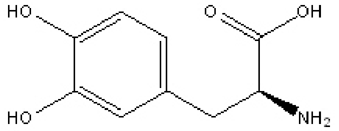

Carbidopa and Levodopa Tablets, USP are supplied as tablets in three strengths:

Carbidopa and Levodopa Tablets USP, 10 mg/100 mg contains 10 mg of carbidopa and 100 mg of levodopa.
Carbidopa and Levodopa Tablets USP, 25 mg/100 mg contains 25 mg of carbidopa and 100 mg of levodopa.
Carbidopa and Levodopa Tablets USP, 25 mg/250 mg contains 25 mg of carbidopa and 250 mg of levodopa.

Inactive ingredients are crospovidone, hydroxypropyl cellulose, magnesium stearate, microcrystalline cellulose and pregelatinized starch (corn). In addition, the 10 mg/100 mg and 25 mg/250 mg tablets contain FD&C Blue No. 2 Aluminum Lake and the 25 mg/100 mg tablets contain D&C Yellow No. 10

Aluminum Lake.

CLINICAL PHARMACOLOGY

Mechanism of Action

Parkinson's disease is a progressive, neurodegenerative disorder of the extrapyramidal nervous system affecting the mobility and control of the skeletal muscular system. Its characteristic features include resting tremor, rigidity and bradykinetic movements. Symptomatic treatments, such as levodopa therapies, may permit the patient better mobility.

Current evidence indicates that symptoms of Parkinson's disease are related to depletion of dopamine in the corpus striatum. Administration of dopamine is ineffective in the treatment of Parkinson's disease apparently because it does not cross the blood-brain barrier. However, levodopa, the metabolic precursor of dopamine, does cross the blood-brain barrier and presumably is converted to dopamine in the brain. This is thought to be the mechanism whereby levodopa relieves symptoms of Parkinson's disease.

Pharmacodynamics

When levodopa is administered orally, it is rapidly decarboxylated to dopamine in extracerebral tissues so that only a small portion of a given dose is transported unchanged to the central nervous system. For this reason, large doses of levodopa are required for adequate therapeutic effect and these may often be accompanied by nausea and other adverse reactions, some of which are attributable to dopamine formed in extracerebral tissues.

Since levodopa competes with certain amino acids for transport across the gut wall, the absorption of levodopa may be impaired in some patients on a high protein diet.

Carbidopa inhibits decarboxylation of peripheral levodopa. It does not cross the blood-brain barrier and does not affect the metabolism of levodopa within the central nervous system.

The incidence of levodopa-induced nausea and vomiting is less with carbidopa and levodopa than with levodopa. In many patients, this reduction in nausea and vomiting will permit more rapid dosage titration.

Since its decarboxylase inhibiting activity is limited to extracerebral tissues, administration of carbidopa with levodopa makes more levodopa available for transport to the brain.

Pharmacokinetics

Carbidopa reduces the amount of levodopa required to produce a given response by about 75% and, when administered with levodopa, increases both plasma levels and the plasma half-life of levodopa and decreases plasma and urinary dopamine and homovanillic acid.

The plasma half-life of levodopa is about 50 minutes, without carbidopa. When carbidopa and levodopa are administered together, the half-life of levodopa is increased to about 1.5 hours. At steady-state, the bioavailability of carbidopa from carbidopa and levodopa tablets is approximately 99% relative to the concomitant administration of carbidopa and levodopa.

In clinical pharmacologic studies, simultaneous administration of carbidopa and levodopa produced greater urinary excretion of levodopa in proportion to the excretion of dopamine than administration of the two drugs at separate times.

Pyridoxine hydrochloride (vitamin B_6), in oral doses of 10 mg to 25 mg, may reverse the effects of levodopa by increasing the rate of aromatic amino acid decarboxylation. Carbidopa inhibits this action of pyridoxine; therefore, carbidopa and levodopa tablets can be given to patients receiving supplemental pyridoxine (vitamin B_6).

Special Populations

Geriatric

A study in eight young healthy subjects (21 to 22 yr) and eight elderly healthy subjects (69 to 76 yr) showed that the absolute bioavailability of levodopa was similar between young and elderly subjects following oral administration of levodopa and carbidopa. However, the systemic exposure (AUC) of levodopa was increased by 55% in elderly subjects compared to young subjects. Based on another study in forty patients with Parkinson's disease, there was a correlation between age of patients and the increase of AUC of levodopa following administration of levodopa and an inhibitor of peripheral dopa decarboxylase. AUC of levodopa was increased by 28% in elderly patients (≥ 65 yr) compared to young patients (< 65 yr). Additionally, mean value of C_{max} for levodopa was increased by 24% in elderly patients (≥ 65 yr) compared to young patients (< 65 yr) (see *PRECAUTIONS: Geriatric Use*).

The AUC of carbidopa was increased in elderly subjects (n = 10, 65 to 76 yr) by 29% compared to young subjects (n = 24, 23 to 64 yr) following IV administration of 50 mg levodopa with carbidopa (50 mg). This increase is not considered a clinically significant impact.

INDICATIONS AND USAGE

Carbidopa and levodopa tablets, USP are indicated in the treatment of Parkinson's disease, post-encephalitic parkinsonism, and symptomatic parkinsonism that may follow carbon monoxide intoxication or manganese intoxication.

Carbidopa allows patients treated for Parkinson's disease to use much lower doses of levodopa. Some patients who responded poorly to levodopa have improved on carbidopa and levodopa tablets. This is most likely due to decreased peripheral decarboxylation of levodopa caused by administration of carbidopa rather than by a primary effect of carbidopa on the nervous system. Carbidopa has not been shown to enhance the intrinsic efficacy of levodopa.

Carbidopa may also reduce nausea and vomiting and permit more rapid titration of levodopa.

CONTRAINDICATIONS

Nonselective monoamine oxidase (MAO) inhibitors are contraindicated for use with carbidopa and levodopa tablets. These inhibitors must be discontinued at least 2 weeks prior to initiating therapy with carbidopa and levodopa tablets. Carbidopa and levodopa tablets may be administered concomitantly with the manufacturer's recommended dose of an MAO inhibitor with selectivity for MAO type B (e.g., selegiline hydrochloride) (see PRECAUTIONS: Drug Interactions).

Carbidopa and levodopa tablets are contraindicated in patients with known hypersensitivity to any component of this drug and in patients with narrow angle glaucoma.

WARNINGS

When carbidopa and levodopa is to be given to patients who are being treated with levodopa, levodopa must be discontinued at least 12 hours before therapy with carbidopa and levodopa tablets is started. In order to reduce adverse reactions, it is necessary to individualize therapy. See DOSAGE AND ADMINISTRATION section before initiating therapy.

The addition of carbidopa with levodopa in the form of carbidopa and levodopa tablets reduces the peripheral effects (nausea, vomiting) due to decarboxylation of levodopa; however, carbidopa does not decrease the adverse reactions due to the central effects of levodopa. Because carbidopa permits more levodopa to reach the brain and more dopamine to be formed, certain adverse central nervous system (CNS) effects, e.g., dyskinesias (involuntary movements), may occur at lower dosages and sooner with carbidopa and levodopa than with levodopa alone.

All patients should be observed carefully for the development of depression with concomitant suicidal

tendencies.

Carbidopa and levodopa tablets should be administered cautiously to patients with severe cardiovascular or pulmonary disease, bronchial asthma, renal, hepatic or endocrine disease.

As with levodopa, care should be exercised in administering carbidopa and levodopa tablets to patients with a history of myocardial infarction who have residual atrial, nodal or ventricular arrhythmias. In such patients, cardiac function should be monitored with particular care during the period of initial dosage adjustment, in a facility with provisions for intensive cardiac care.

As with levodopa, treatment with carbidopa and levodopa tablets may increase the possibility of upper gastrointestinal hemorrhage in patients with a history of peptic ulcer.

Falling Asleep During Activities of Daily Living and Somnolence

Patients taking carbidopa and levodopa tablets alone or with other dopaminergic drugs have reported suddenly falling asleep without prior warning of sleepiness while engaged in activities of daily living (includes operation of motor vehicles). Road traffic accidents attributed to sudden sleep onset have been reported. Although many patients reported somnolence while on dopaminergic medications, there have been reports of road traffic accidents attributed to sudden onset of sleep in which the patient did not perceive any warning signs, such as excessive drowsiness, and believed that they were alert immediately prior to the event. Sudden onset of sleep has been reported to occur as long as one year after the initiation of treatment.

Falling asleep while engaged in activities of daily living usually occurs in patients experiencing preexisting somnolence, although some patients may not give such a history. For this reason, prescribers should reassess patients for drowsiness or sleepiness especially since some of the events occur well after the start of treatment. Prescribers should be aware that patients may not acknowledge drowsiness or sleepiness until directly questioned about drowsiness or sleepiness during specific activities. Patients should be advised to exercise caution while driving or operating machines during treatment with carbidopa and levodopa tablets. Patients who have already experienced somnolence or an episode of sudden sleep onset should not participate in these activities during treatment with carbidopa and levodopa tablets.

Before initiating treatment with carbidopa and levodopa tablets, advise patients about the potential to develop drowsiness and ask specifically about factors that may increase the risk for somnolence with carbidopa and levodopa tablets such as the use of concomitant sedating medications and the presence of sleep disorders. Consider discontinuing carbidopa and levodopa tablets in patients who report significant daytime sleepiness or episodes of falling asleep during activities that require active participation (e.g., conversations, eating, etc.). If treatment with carbidopa and levodopa tablets continues, patients should be advised not to drive and to avoid other potentially dangerous activities that might result in harm if the patients become somnolent. There is insufficient information to establish that dose reduction will eliminate episodes of falling asleep while engaged in activities of daily living.

Hyperpyrexia and Confusion

Sporadic cases of a symptom complex resembling neuroleptic malignant syndrome (NMS) have been reported in association with dose reductions or withdrawal of certain antiparkinsonian agents such as levodopa, carbidopa levodopa, or carbidopa levodopa extended release. Therefore, patients should be observed carefully when the dosage of levodopa is reduced abruptly or discontinued, especially if the patient is receiving neuroleptics.

NMS is an uncommon but life threatening syndrome characterized by fever or hyperthermia. Neurological findings, including muscle rigidity, involuntary movements, altered consciousness, mental status changes; other disturbances, such as autonomic dysfunction, tachycardia, tachypnea, sweating, hyper- or hypotension; laboratory findings, such as creatine phosphokinase elevation, leukocytosis, myoglobinuria and increased serum myoglobin have been reported.

The early diagnosis of this condition is important for the appropriate management of these patients. Considering NMS as a possible diagnosis and ruling out other acute illnesses (e.g., pneumonia, systemic infection, etc.) is essential. This may be especially complex if the clinical presentation includes both serious medical illness and untreated or inadequately treated extrapyramidal signs and symptoms (EPS). Other important considerations in the differential diagnosis include central anticholinergic toxicity, heat stroke, drug fever and primary central nervous system (CNS) pathology.

The management of NMS should include: 1) intensive symptomatic treatment and medical monitoring and 2) treatment of any concomitant serious medical problems for which specific treatments are available. Dopamine agonists, such as bromocriptine and muscle relaxants, such as dantrolene, are often used in the treatment of NMS, however, their effectiveness has not been demonstrated in controlled studies.

PRECAUTIONS

General

As with levodopa, periodic evaluations of hepatic, hematopoietic, cardiovascular and renal function are recommended during extended therapy.

Patients with chronic wide-angle glaucoma may be treated cautiously with carbidopa and levodopa tablets provided the intraocular pressure is well controlled and the patient is monitored carefully for changes in intraocular pressure during therapy.

Dopaminergic agents, including levodopa, may be associated with somnolence and very rarely episodes of sudden onset of sleep. In some cases, these episodes may occur without awareness or warning during daily activities. Patients must be informed of this and advised to exercise caution while driving or operating machines while being treated with dopaminergic agents, including levodopa. Patients who have experienced somnolence and/or an episode of sudden sleep onset must refrain from driving or operating machines (see Information for Patients).

Dyskinesia

Levodopa alone, as well as carbidopa and levodopa tablets, are associated with dyskinesias. The occurrence of dyskinesias may require dosage reduction.

Hallucinations/Psychotic-Like Behavior

Hallucinations and psychotic-like behavior have been reported with dopaminergic medications. In general, hallucinations present shortly after the initiation of therapy and may be responsive to dose reduction in levodopa. Hallucinations may be accompanied by confusion and to a lesser extent sleep disorder (insomnia) and excessive dreaming.

Carbidopa and levodopa tablets may have similar effects on thinking and behavior. This abnormal thinking and behavior may present with one or more symptoms, including paranoid ideation, delusions, hallucinations, confusion, psychotic-like behavior, disorientation, aggressive behavior, agitation, and delirium.

Ordinarily, patients with a major psychotic disorder should not be treated with carbidopa and levodopa tablets, because of the risk of exacerbating psychosis. In addition, certain medications used to treat psychosis may exacerbate the symptoms of Parkinson's disease and may decrease the effectiveness of carbidopa and levodopa tablets.

Impulse Control/Compulsive Behaviors

Reports of patients taking dopaminergic medications (medications that increase central dopaminergic tone), suggest that patients may experience an intense urge to gamble, increased sexual urges, intense urges to spend money, binge eating, and/or other intense urges, and the inability to control these urges. In some cases, although not all, these urges were reported to have stopped when the dose was reduced

or the medication was discontinued. Because patients may not recognize these behaviors as abnormal, it is important for prescribers to specifically ask patients or the caregivers about the development of new or increased gambling urges, sexual urges, uncontrolled spending or other urges while being treated with carbidopa and levodopa tablets. Physicians should consider dose reduction or stopping the medication if a patient develops such urges while taking carbidopa and levodopa tablets (see Information for Patients).

Melanoma

Epidemiological studies have shown that patients with Parkinson's disease have a higher risk (2- to approximately 6-fold higher) of developing melanoma than the general population. Whether the increased risk observed was due to Parkinson's disease or other factors, such as drugs used to treat Parkinson's disease, is unclear.

For the reasons stated above, patients and providers are advised to monitor for melanomas frequently and on a regular basis when using carbidopa and levodopa for any indication. Ideally, periodic skin examinations should be performed by appropriately qualified individuals (e.g., dermatologists).

Information for Patients

The patient should be informed that carbidopa and levodopa tablets are an immediate-release formulation of carbidopa and levodopa that is designed to begin release of ingredients within 30 minutes. It is important that carbidopa and levodopa tablets be taken at regular intervals according to the schedule outlined by the physician. The patient should be cautioned not to change the prescribed dosage regimen and not to add any additional antiparkinson medications, including other carbidopa and levodopa preparations, without first consulting the physician.

Patients should be advised that sometimes a 'wearing-off' effect may occur at the end of the dosing interval. The physician should be notified if such response poses a problem to lifestyle.

Patients should be advised that occasionally, dark color (red, brown or black) may appear in saliva, urine or sweat after ingestion of carbidopa and levodopa. Although the color appears to be clinically insignificant, garments may become discolored.

The patient should be advised that a change in diet to foods that are high in protein may delay the absorption of levodopa and may reduce the amount taken up in the circulation. Excessive acidity also delays stomach emptying, thus delaying the absorption of levodopa. Iron salts (such as in multivitamin tablets) may also reduce the amount of levodopa available to the body. The above factors may reduce the clinical effectiveness of the levodopa or carbidopa and levodopa therapy.

Patients should be alerted to the possibility of sudden onset of sleep during daily activities, in some cases without awareness or warning signs, when they are taking dopaminergic agents, including levodopa. Patients should be advised to exercise caution while driving or operating machinery and that if they have experienced somnolence and/or sudden sleep onset, they must refrain from these activities (see WARNINGS: Falling Asleep During Activities of Daily Living and Somnolence).

There have been reports of patients experiencing intense urges to gamble, increased sexual urges and other intense urges and the inability to control these urges while taking one or more of the medications that increase central dopaminergic tone and that are generally used for the treatment of Parkinson's disease, including carbidopa and levodopa. Although it is not proven that the medications caused these events, these urges were reported to have stopped in some cases when the dose was reduced or the medication was stopped. Prescribers should ask patients about the development of new or increased gambling urges, sexual urges or other urges while being treated with carbidopa and levodopa. Patients should inform their physician if they experience new or increased gambling urges, increased sexual urges or other intense urges while taking carbidopa and levodopa. Physicians should consider dose reduction or stopping the medication if a patient develops such urges while taking carbidopa and levodopa (see PRECAUTIONS: Impulse Control/Compulsive Behaviors).

Laboratory Tests

Abnormalities in laboratory tests may include elevations of liver function tests such as alkaline phosphatase, SGOT (AST), SGPT (ALT), lactic dehydrogenase (LDH) and bilirubin. Abnormalities in blood urea nitrogen (BUN) and positive Coombs test have also been reported. Commonly, levels of blood urea nitrogen, creatinine and uric acid are lower during administration of carbidopa and levodopa tablets than with levodopa.

Carbidopa and levodopa may cause a false-positive reaction for urinary ketone bodies when a test tape is used for determination of ketonuria. This reaction will not be altered by boiling the urine specimen. False-negative tests may result with the use of glucose-oxidase methods of testing for glucosuria.

Cases of falsely diagnosed pheochromocytoma in patients on carbidopa and levodopa therapy have been reported very rarely. Caution should be exercised when interpreting the plasma and urine levels of catecholamines and their metabolites in patients on levodopa or carbidopa and levodopa therapy.

Drug Interactions

Caution should be exercised when the following drugs are administered concomitantly with carbidopa and levodopa.

Symptomatic postural hypotension occurred when carbidopa and levodopa was added to the treatment of a patient receiving antihypertensive drugs. Therefore, when therapy with carbidopa and levodopa tablets is started, dosage adjustment of the antihypertensive drug may be required.

For patients receiving MAO inhibitors (Type A or B), see CONTRAINDICATIONS. Concomitant therapy with selegiline and carbidopa and levodopa may be associated with severe orthostatic hypotension not attributable to carbidopa and levodopa alone (see CONTRAINDICATIONS).

There have been rare reports of adverse reactions, including hypertension and dyskinesia, resulting from the concomitant use of tricyclic antidepressants and carbidopa and levodopa.

Dopamine D_2 receptor antagonists (e.g., phenothiazines, butyrophenones, risperidone) and isoniazid may reduce the therapeutic effects of levodopa. In addition, the beneficial effects of levodopa in Parkinson's disease have been reported to be reversed by phenytoin and papaverine. Patients taking these drugs with carbidopa and levodopa tablets should be carefully observed for loss of therapeutic response.

Use of carbidopa and levodopa tablets with dopamine-depleting agents (e.g., reserpine and tetrabenazine) or other drugs known to deplete monoamine stores is not recommended.

Carbidopa and levodopa tablets and iron salts or multivitamins containing iron salts should be coadministered with caution. Iron salts can form chelates with levodopa and carbidopa and consequently reduce the bioavailability of carbidopa and levodopa.

Although metoclopramide may increase the bioavailability of levodopa by increasing gastric emptying, metoclopramide may also adversely affect disease control by its dopamine receptor antagonistic properties.

Carcinogenesis, Mutagenesis, Impairment of Fertility

In a 2 year bioassay of carbidopa and levodopa, no evidence of carcinogenicity was found in rats receiving doses of approximately 2 times the maximum daily human dose of carbidopa and 4 times the maximum daily human dose of levodopa.

In reproduction studies with carbidopa and levodopa, no effects on fertility were found in rats receiving doses of approximately 2 times the maximum daily human dose of carbidopa and 4 times the maximum daily human dose of levodopa.

Pregnancy

Teratogenic Effects. Pregnancy Category C

No teratogenic effects were observed in a study in mice receiving up to 20 times the maximum recommended human dose of carbidopa and levodopa tablets. There was a decrease in the number of live pups delivered by rats receiving approximately 2 times the maximum recommended human dose of carbidopa and approximately 5 times the maximum recommended human dose of levodopa during organogenesis. Carbidopa and levodopa caused both visceral and skeletal malformations in rabbits at all doses and ratios of carbidopa/levodopa tested, which ranged from 10 times/5 times the maximum recommended human dose of carbidopa/levodopa to 20 times/10 times the maximum recommended human dose of carbidopa/levodopa.

There are no adequate or well controlled studies in pregnant women. It has been reported from individual cases that levodopa crosses the human placental barrier, enters the fetus and is metabolized. Carbidopa concentrations in fetal tissue appeared to be minimal. Use of carbidopa and levodopa in women of childbearing potential requires that the anticipated benefits of the drug be weighed against possible hazards to mother and child.

Nursing Mothers

Levodopa has been detected in human milk. Caution should be exercised when carbidopa and levodopa tablets are administered to a nursing woman.

Pediatric Use

Safety and effectiveness in pediatric patients have not been established. Use of the drug in patients below the age of 18 is not recommended.

Geriatric Use

In the clinical efficacy trials for carbidopa and levodopa tablets, almost half of the patients were older than 65, but few were older than 75. No overall meaningful differences in safety or effectiveness were observed between these subjects and younger subjects, but greater sensitivity of some older individuals to adverse drug reactions such as hallucinations cannot be ruled out. There is no specific dosing recommendation based upon clinical pharmacology data as carbidopa and levodopa tablets are titrated as tolerated for clinical effect.

ADVERSE REACTIONS

The most common adverse reactions reported with carbidopa and levodopa have included dyskinesias, such as choreiform, dystonic and other involuntary movements and nausea.

The following other adverse reactions have been reported with carbidopa and levodopa:

Body as a Whole: chest pain, asthenia

Cardiovascular: cardiac irregularities, hypotension, orthostatic effects including orthostatic hypotension, hypertension, syncope, phlebitis, palpitation

Gastrointestinal: dark saliva, gastrointestinal bleeding, development of duodenal ulcer, anorexia, vomiting, diarrhea, constipation, dyspepsia, dry mouth, taste alterations

Hematologic: agranulocytosis, hemolytic and non-hemolytic anemia, thrombocytopenia, leukopenia

Hypersensitivity: angioedema, urticaria, pruritus, Henoch-Schonlein purpura, bullous lesions (including pemphigus-like reactions)

Musculoskeletal: back pain, shoulder pain, muscle cramps

Nervous System/Psychiatric: psychotic episodes including delusions, hallucinations and paranoid ideation, bradykinetic episodes ("on-off" phenomenon), confusion, agitation, dizziness, somnolence, dream abnormalities including nightmares, insomnia, paresthesia, headache, depression with or without development of suicidal tendencies, dementia, pathological gambling, increased libido including

hypersexuality, impulse control symptoms. Convulsions also have occurred; however, a causal relationship with carbidopa and levodopa has not been established.

Respiratory: dyspnea, upper respiratory infection

Skin: rash, increased sweating, alopecia, dark sweat

Urogenital: urinary tract infection, urinary frequency, dark urine

Laboratory Tests: decreased hemoglobin and hematocrit; abnormalities in alkaline phosphatase, SGOT (AST), SGPT (ALT), LDH, bilirubin, BUN, Coombs test; elevated serum glucose; white blood cells, bacteria and blood in the urine

Other adverse reactions that have been reported with levodopa alone and with various carbidopa and levodopa formulations and may occur with carbidopa and levodopa tablets are:

Body as a Whole: abdominal pain and distress, fatigue

Cardiovascular: myocardial infarction

Gastrointestinal: gastrointestinal pain, dysphagia, sialorrhea, flatulence, bruxism, burning sensation of the tongue, heartburn, hiccups

Metabolic: edema, weight gain, weight loss

Musculoskeletal: leg pain

Nervous System/Psychiatric: ataxia, extrapyramidal disorder, falling, anxiety, gait abnormalities, nervousness, decreased mental acuity, memory impairment, disorientation, euphoria, blepharospasm (which may be taken as an early sign of excess dosage; consideration of dosage reduction may be made at this time), trismus, increased tremor, numbness, muscle twitching, activation of latent Horner's syndrome, peripheral neuropathy

Respiratory: pharyngeal pain, cough

Skin: malignant melanoma (see also CONTRAINDICATIONS), flushing

Special Senses: oculogyric crises, diplopia, blurred vision, dilated pupils

Urogenital: urinary retention, urinary incontinence, priapism

Miscellaneous: bizarre breathing patterns, faintness, hoarseness, malaise, hot flashes, sense of stimulation

Laboratory Tests: decreased white blood cell count and serum potassium; increased serum creatinine and uric acid; protein and glucose in urine

OVERDOSAGE

Management of acute overdosage with carbidopa and levodopa is the same as management of acute overdosage with levodopa. Pyridoxine is not effective in reversing the actions of carbidopa and levodopa tablets.

General supportive measures should be employed, along with immediate gastric lavage. Intravenous fluids should be administered judiciously and an adequate airway maintained. Electrocardiographic monitoring should be instituted and the patient carefully observed for the development of arrhythmias; if required, appropriate antiarrhythmic therapy should be given. The possibility that the patient may have taken other drugs as well as carbidopa and levodopa tablets should be taken into consideration. To date, no experience has been reported with dialysis; hence, its value in overdosage is not known.

Based on studies in which high doses of levodopa and/or carbidopa were administered, a significant proportion of rats and mice given single oral doses of levodopa of approximately 1500 to 2000 mg/kg are expected to die. A significant proportion of infant rats of both sexes are expected to die at a dose of

800 mg/kg. A significant proportion of rats are expected to die after treatment with similar doses of carbidopa. The addition of carbidopa in a 1:10 ratio with levodopa increases the dose at which a significant proportion of mice are expected to die to 3360 mg/kg.

DOSAGE AND ADMINISTRATION

The optimum daily dosage of carbidopa and levodopa tablets must be determined by careful titration in each patient. Carbidopa and levodopa tablets are available in a 1:4 ratio of carbidopa to levodopa (carbidopa and levodopa tablets 25 mg/100 mg) as well as 1:10 ratio (carbidopa and levodopa tablets 25 mg/250 mg and carbidopa and levodopa tablets 10 mg/100 mg). Tablets of the two ratios may be given separately or combined as needed to provide the optimum dosage.

Studies show that peripheral dopa decarboxylase is saturated by carbidopa at approximately 70 mg to 100 mg a day. Patients receiving less than this amount of carbidopa are more likely to experience nausea and vomiting.

Usual Initial Dosage

Dosage is best initiated with one tablet of carbidopa and levodopa 25 mg/100 mg 3 times a day. This dosage schedule provides 75 mg of carbidopa per day. Dosage may be increased by one tablet every day or every other day, as necessary, until a dosage of eight tablets of carbidopa and levodopa 25 mg/100 mg a day is reached.

If carbidopa and levodopa 10 mg/100 mg is used, dosage may be initiated with one tablet 3 or 4 times a day. However, this will not provide an adequate amount of carbidopa for many patients. Dosage may be increased by one tablet every day or every other day until a total of eight tablets (two tablets q.i.d.) is reached.

How to Transfer Patients from Levodopa

Levodopa must be discontinued at least 12 hours before starting carbidopa and levodopa tablets. A daily dosage of carbidopa and levodopa should be chosen that will provide approximately 25% of the previous levodopa dosage. Patients who are taking less than 1500 mg of levodopa a day should be started on one tablet of carbidopa and levodopa 25 mg/100 mg 3 or 4 times a day. The suggested starting dosage for most patients taking more than 1500 mg of levodopa is one tablet of carbidopa and levodopa 25 mg/250 mg 3 or 4 times a day.

Maintenance

Therapy should be individualized and adjusted according to the desired therapeutic response. At least 70 mg to 100 mg of carbidopa per day should be provided. When a greater proportion of carbidopa is required, one tablet of carbidopa and levodopa 25 mg/100 mg may be substituted for each tablet of carbidopa and levodopa 10 mg/100 mg. When more levodopa is required, carbidopa and levodopa 25 mg/250 mg should be substituted for carbidopa and levodopa 25 mg/100 mg or carbidopa and levodopa 10 mg/100 mg. If necessary, the dosage of carbidopa and levodopa 25 mg/250 mg may be increased by one-half or one tablet every day or every other day to a maximum of eight tablets a day. Experience with total daily dosages of carbidopa greater than 200 mg is limited.

Because both therapeutic and adverse responses occur more rapidly with carbidopa and levodopa than with levodopa alone, patients should be monitored closely during the dose adjustment period. Specifically, involuntary movements will occur more rapidly with carbidopa and levodopa than with levodopa. The occurrence of involuntary movements may require dosage reduction. Blepharospasm may be a useful early sign of excess dosage in some patients.

Addition of Other Antiparkinsonian Medications

Standard drugs for Parkinson's disease, other than levodopa without a decarboxylase inhibitor, may be

used concomitantly while carbidopa and levodopa tablets are being administered, although dosage adjustments may be required.

Interruption of Therapy

Sporadic cases of hyperpyrexia and confusion have been associated with dose reductions and withdrawal of carbidopa and levodopa. Patients should be observed carefully if abrupt reduction or discontinuation of carbidopa and levodopa is required, especially if the patient is receiving neuroleptics. (See WARNINGS.)

If general anesthesia is required, carbidopa and levodopa tablets may be continued as long as the patient is permitted to take fluids and medication by mouth. If therapy is interrupted temporarily, the patient should be observed for symptoms resembling NMS and the usual daily dosage may be administered as soon as the patient is able to take oral medication.

HOW SUPPLIED

Carbidopa and Levodopa Tablets, USP are available containing 10 mg or 25 mg of carbidopa, USP and 100 mg or 250 mg of levodopa, USP.

The 10 mg/100 mg tablets are blue, round, scored and debossed with **M** above the score and **CL1** below the score on one side of the tablet and blank on the other side. They are available as follows:

NDC 0378-0078-01
bottles of 100 tablets

The 25 mg/100 mg tablets are yellow, round, scored and debossed with **M** above the score and **CL2** below the score on one side of the tablet and blank on the other side. They are available as follows:

NDC 0378-0085-01
bottles of 100 tablets

The 25 mg/250 mg tablets are blue, round, scored and debossed with **M** above the score and **CL3** below the score on one side of the tablet and blank on the other side. They are available as follows:

NDC 0378-1133-01
bottles of 100 tablets

Store at 20° to 25°C (68° to 77°F). [See USP Controlled Room Temperature.]

Protect from light.

Dispense in a tight, light-resistant container as defined in the USP using a child-resistant closure.

Mylan Pharmaceuticals Inc.
Morgantown, WV 26505 U.S.A.

REVISED OCTOBER 2014
CDLDT:R3

APPENDIX B. UNITED KINGDOM PARKINSON'S DISEASE SOCIETY BRAIN BANK DIAGNOSTIC CRITERIA FOR PARKINSON'S DISEASE

**Step 1: Diagnosis of Parkinsonism**
Bradykinesia **and** at least one of the following:<br>• Muscular rigidity<br>• 4–6 Hz resting tremor<br>• postural instability not caused by primary visual, vestibular, cerebellar or Proprioceptive dysfunction
**Step 2: Features tending to exclude Parkinson's disease as the cause of Parkinsonism**
• History of repeated strokes with stepwise progression of parkinsonian features<br>• History of repeated head injury<br>• History of definite encephalitis<br>• Neuroleptic treatment at onset of symptoms<br>• >1 affected relatives<br>• Sustained remission<br>• Strictly unilateral features after 3 years<br>• Supranuclear gaze palsy<br>• Cerebellar signs<br>• Early severe autonomic involvement<br>• Early severe dementia with disturbances of memory, language and praxis<br>• Babinski's sign<br>• Presence of a cerebral tumour or communicating hydrocephalus on computed tomography scan<br>• Negative response to large doses of levodopa (if malabsorption excluded)<br>• MPTP exposure
**Step 3: Features that support a diagnosis of Parkinson's disease (three or more required for diagnosis of definite Parkinson's disease)**
• Unilateral onset<br>• Rest tremor present<br>• Progressive disorder<br>• Persistent asymmetry affecting the side of onset most<br>• Excellent (70–100%) response to levodopa<br>• Severe levodopa-induced chorea<br>• Levodopa response for ≥5 years<br>• Clinical course of ≥10 years

APPENDIX C. MONTREAL COGNITIVE ASSESSMENT (MOCA)

Montreal Cognitive Assessment
(MoCA)

Administration and Scoring Instructions

The Montreal Cognitive Assessment (MoCA) was designed as a rapid screening instrument for mild cognitive dysfunction. It assesses different cognitive domains: attention and concentration, executive functions, memory, language, visuoconstructional skills, conceptual thinking, calculations, and orientation. Time to administer the MoCA is approximately 10 minutes. The total possible score is 30 points; a score of 26 or above is considered normal.

1. Alternating Trail Making:

Administration: The examiner instructs the subject: *"Please draw a line, going from a number to a letter in ascending order. Begin here* [point to (1)] *and draw a line from 1 then to A then to 2 and so on. End here* [point to (E)]."

Scoring: Allocate one point if the subject successfully draws the following pattern:
1 –A- 2- B- 3- C- 4- D- 5- E, without drawing any lines that cross. Any error that is not immediately self-corrected earns a score of 0.

2. Visuoconstructional Skills (Cube):

Administration: The examiner gives the following instructions, pointing to the **cube**: "*Copy this drawing as accurately as you can, in the space below*".

Scoring: One point is allocated for a correctly executed drawing.
• Drawing must be three-dimensional
• All lines are drawn
• No line is added
• Lines are relatively parallel and their length is similar (rectangular prisms are accepted)

A point is not assigned if any of the above-criteria are not met.

3. Visuoconstructional Skills (Clock):

Administration: Indicate the right third of the space and give the following instructions: *"Draw a clock. Put in all the numbers and set the time to 10 past 11".*

Scoring: One point is allocated for each of the following three criteria:
• Contour (1 pt.): the clock face must be a circle with only minor distortion acceptable (e.g., slight imperfection on closing the circle);
• Numbers (1 pt.): all clock numbers must be present with no additional numbers; numbers must be in the correct order and placed in the approximate quadrants on the clock face; Roman numerals are acceptable; numbers can be placed outside the circle contour;
• Hands (1 pt.): there must be two hands jointly indicating the correct time; the hour hand must be clearly shorter than the minute hand; hands must be centred within the clock face with their junction close to the clock centre.

A point is not assigned for a given element if any of the above-criteria are not met.

© Z. Nasreddine MD
www.mocatest.org

4. Naming:

Administration: Beginning on the left, point to each figure and say: *"Tell me the name of this animal".*

Scoring: One point each is given for the following responses: (1) lion (2) rhinoceros or rhino (3) camel or dromedary.

5. Memory:

Administration: The examiner reads a list of 5 words at a rate of one per second, giving the following instructions: *"This is a memory test. I am going to read a list of words that you will have to remember now and later on. Listen carefully. When I am through, tell me as many words as you can remember. It doesn't matter in what order you say them".* Mark a check in the allocated space for each word the subject produces on this first trial. When the subject indicates that (s)he has finished (has recalled all words), or can recall no more words, read the list a second time with the following instructions: *"I am going to read the same list for a second time. Try to remember and tell me as many words as you can, including words you said the first time."* Put a check in the allocated space for each word the subject recalls after the second trial.
At the end of the second trial, inform the subject that (s)he will be asked to recall these words again by saying, *"I will ask you to recall those words again at the end of the test."*

Scoring: No points are given for Trials One and Two.

6. Attention:

Forward Digit Span: Administration: Give the following instruction: "*I am going to say some numbers and when I am through, repeat them to me exactly as I said them*". Read the five number sequence at a rate of one digit per second.

Backward Digit Span: Administration: Give the following instruction: "*Now I am going to say some more numbers, but when I am through you must repeat them to me in the backwards order.*" Read the three number sequence at a rate of one digit per second.

Scoring: Allocate one point for each sequence correctly repeated, (*N.B.*: the correct response for the backwards trial is 2-4-7).

Vigilance: Administration: The examiner reads the list of letters at a rate of one per second, after giving the following instruction: "*I am going to read a sequence of letters. Every time I say the letter A, tap your hand once. If I say a different letter, do not tap your hand*".

Scoring: Give one point if there is zero to one errors (an error is a tap on a wrong letter or a failure to tap on letter A).

© Z. Nasreddine MD *www.mocatest.org*

Serial 7s: Administration: The examiner gives the following instruction: *"Now, I will ask you to count by subtracting seven from 100, and then, keep subtracting seven from your answer until I tell you to stop."* Give this instruction twice if necessary.

Scoring: This item is scored out of 3 points. Give no (0) points for no correct subtractions, 1 point for one correction subtraction, 2 points for two-to-three correct subtractions, and 3 points if the participant successfully makes four or five correct subtractions. Count each correct subtraction of 7 beginning at 100. Each subtraction is evaluated independently; that is, if the participant responds with an incorrect number but continues to correctly subtract 7 from it, give a point for each correct subtraction. For example, a participant may respond "92 – 85 – 78 – 71 – 64" where the "92" is incorrect, but all subsequent numbers are subtracted correctly. This is one error and the item would be given a score of 3.

7. Sentence repetition:

Administration: The examiner gives the following instructions: *"I am going to read you a sentence. Repeat it after me, exactly as I say it* [pause]: ***I only know that John is the one to help today."*** Following the response, say: *"Now I am going to read you another sentence. Repeat it after me, exactly as I say it* [pause]: ***The cat always hid under the couch when dogs were in the room."***

Scoring: Allocate 1 point for each sentence correctly repeated. Repetition must be exact. Be alert for errors that are omissions (e.g., omitting "only", "always") and substitutions/additions (e.g., "John is the one who helped today;" substituting "hides" for "hid", altering plurals, etc.).

8. Verbal fluency:

Administration: The examiner gives the following instruction: *"Tell me as many words as you can think of that begin with a certain letter of the alphabet that I will tell you in a moment. You can say any kind of word you want, except for proper nouns (like Bob or Boston), numbers, or words that begin with the same sound but have a different suffix, for example, love, lover, loving. I will tell you to stop after one minute. Are you ready?* [Pause] *Now, tell me as many words as you can think of that begin with the letter F.* [time for 60 sec]. *Stop."*

Scoring: Allocate one point if the subject generates 11 words or more in 60 sec. Record the subject's response in the bottom or side margins.

9. Abstraction:

Administration: The examiner asks the subject to explain what each pair of words has in common, starting with the example: *"Tell me how an orange and a banana are alike".* If the subject answers in a concrete manner, then say only one additional time: *"Tell me another way in which those items are alike".* If the subject does not give the appropriate response *(fruit)*, say, *"Yes, and they are also both fruit."* Do not give any additional instructions or clarification. After the practice trial, say: *"Now, tell me how a train and a bicycle are alike".* Following the response, administer the second trial, saying: *"Now tell me how a ruler and a watch are alike".* Do not give any additional instructions or prompts.

Scoring: Only the last two item pairs are scored. Give 1 point to each item pair correctly answered. The following responses are acceptable:
Train-bicycle = means of transportation, means of travelling, you take trips in both;
Ruler-watch = measuring instruments, used to measure.
The following responses are **not** acceptable: Train-bicycle = they have wheels; Ruler-watch = they have numbers.

10. Delayed recall:

Administration: The examiner gives the following instruction: *"I read some words to you earlier, which I asked you to remember. Tell me as many of those words as you can remember."* Make a check mark (√) for each of the words correctly recalled spontaneously without any cues, in the allocated space.

Scoring: **Allocate 1 point for each word recalled freely without any cues.**

Optional:
Following the delayed free recall trial, prompt the subject with the semantic category cue provided below for any word not recalled. Make a check mark (√) in the allocated space if the subject remembered the word with the help of a category or multiple-choice cue. Prompt all non-recalled words in this manner. If the subject does not recall the word after the category cue, give him/her a multiple choice trial, using the following example instruction, *"Which of the following words do you think it was, NOSE, FACE, or HAND?"*
Use the following category and/or multiple-choice cues for each word, when appropriate:

FACE:	category cue: part of the body	multiple choice: nose, face, hand
VELVET:	category cue: type of fabric	multiple choice: denim, cotton, velvet
CHURCH:	category cue: type of building	multiple choice: church, school, hospital
DAISY:	category cue: type of flower	multiple choice: rose, daisy, tulip
RED:	category cue: a colour	multiple choice: red, blue, green

Scoring: **No points are allocated for words recalled with a cue.** A cue is used for clinical information purposes only and can give the test interpreter additional information about the type of memory disorder. For memory deficits due to retrieval failures, performance can be improved with a cue. For memory deficits due to encoding failures, performance does not improve with a cue.

11. Orientation:

Administration: The examiner gives the following instructions: "Tell me the date today". If the subject does not give a complete answer, then prompt accordingly by saying: *"Tell me the [year, month, exact date, and day of the week]."* Then say: *"Now, tell me the name of this place, and which city it is in."*

Scoring: Give one point for each item correctly answered. The subject must tell the exact date and the exact place (name of hospital, clinic, office). No points are allocated if subject makes an error of one day for the day and date.

TOTAL SCORE: Sum all subscores listed on the right-hand side. Add one point for an individual who has 12 years or fewer of formal education, for a possible maximum of 30 points. A final total score of 26 and above is considered normal.

MONTREAL COGNITIVE ASSESSMENT (MOCA)
Version 7.1 Original Version

NAME :
Education : Date of birth :
Sex : DATE :

	POINTS
**VISUOSPATIAL / EXECUTIVE**	

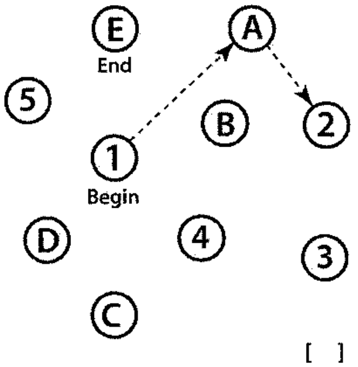

[ ]

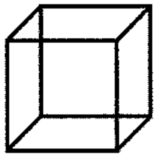

Copy cube [ ]

Draw CLOCK (Ten past eleven) (3 points)

[ ] Contour [ ] Numbers [ ] Hands ___/5

NAMING

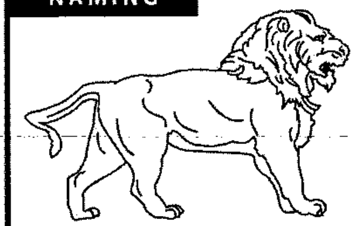
[ ]

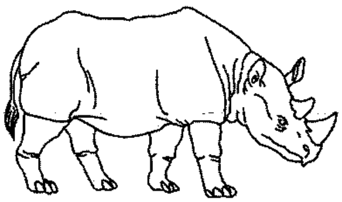
[ ]

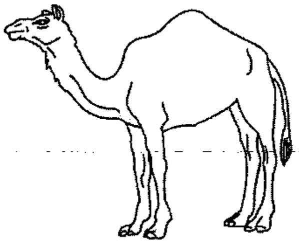
[ ] ___/3

MEMORY Read list of words, subject must repeat them. Do 2 trials, even if 1st trial is successful. Do a recall after 5 minutes.

	FACE	VELVET	CHURCH	DAISY	RED
1st trial					
2nd trial					

No points

ATTENTION Read list of digits (1 digit/ sec.). Subject has to repeat them in the forward order [ ] 2 1 8 5 4
Subject has to repeat them in the backward order [ ] 7 4 2 ___/2

Read list of letters. The subject must tap with his hand at each letter A. No points if ≥ 2 errors
[ ] F B A C M N A A J K L B A F A K D E A A A J A M O F A A B ___/1

Serial 7 subtraction starting at 100 [ ] 93 [ ] 86 [ ] 79 [ ] 72 [ ] 65
4 or 5 correct subtractions: **3 pts**, 2 or 3 correct: **2 pts**, 1 correct: **1 pt**, 0 correct: **0 pt** ___/3

LANGUAGE Repeat : I only know that John is the one to help today. [ ]
The cat always hid under the couch when dogs were in the room. [ ] ___/2

Fluency / Name maximum number of words in one minute that begin with the letter F [ ] _____ (N ≥ 11 words) ___/1

ABSTRACTION Similarity between e.g. banana - orange = fruit [ ] train – bicycle [ ] watch - ruler ___/2

DELAYED RECALL

Has to recall words WITH NO CUE	FACE [ ]	VELVET [ ]	CHURCH [ ]	DAISY [ ]	RED [ ]	Points for UNCUED recall only
**Optional** Category cue						
Multiple choice cue						

___/5

ORIENTATION [ ] Date [ ] Month [ ] Year [ ] Day [ ] Place [ ] City ___/6

© Z.Nasreddine MD www.mocatest.org Normal ≥26 / 30 TOTAL ___/30
Add 1 point if ≤ 12 yr edu

Administered by:______________________________

APPENDIX D. MOVEMENT DISORDERS SOCIETY VERSION OF UNIFIED PARKINSON'S DISEASE RATING SCALE (MDS-UPDRS)

MDS-UPDRS Permissions

Permission is required to use the MDS-developed Rating Scales (with the exception of personal/individual use). Reproduction, translation, modification, sale, or distribution of any portion of the MDS Rating Scales is strictly prohibited. MDS Rating Scales may not be incorporated into clinical trials, training or certification programs or materials, software programs, or otherwise except through use of the Permissions Request Form and payment of applicable fees.

Continue to p. 2 to view the MDS-UPDRS

MDS-UPDRS

The *Movement* Disorder Society (MDS)-sponsored new version of the UPDRS is founded on the critique that was formulated by the Task Force for Rating Scales in Parkinson's disease (*Mov Disord* 2003;18:738-750). Thereafter, the MDS recruited a Chairperson to organize a program to provide the Movement Disorder community with a new version of the UPDRS that would maintain the overall format of the original UPDRS, but address issues identified in the critique as weaknesses and ambiguities. The Chairperson identified subcommittees with chairs and members. Each part was written by the appropriate subcommittee members and then reviewed and ratified by the entire group. These members are listed below.

The MDS-UPDRS has four parts: Part I (non-motor experiences of daily living), Part II (motor experiences of daily living, Part III (motor examination) and Part IV (motor complications). Part I has two components: IA concerns a number of behaviors that are assessed by the investigator with all pertinent information from patients and caregivers, and IB is completed by the patient with or without the aid of the caregiver, but independently of the investigator. These sections can, however, be reviewed by the rater to ensure that all questions are answered clearly and the rater can help explain any perceived ambiguities. Part II is designed to be a self-administered questionnaire like Part IB, but can be reviewed by the investigator to ensure completeness and clarity. Of note, the official versions of Part IA, Part IB and Part II of the MDS-UPDRS do not have separate on or off ratings. However, for individual programs or protocols the same questions can be used separately for on and off. Part III has instructions for the rater to give or demonstrate to the patient; it is completed by the rater. Part IV has instructions for the rater and also instructions to be read to the patient. This part integrates patient-derived information with the rater's clinical observations and judgments and is completed by the rater.

The authors of this new version are:

Chairperson: Christopher G. Goetz
Part I: Werner Poewe (chair), Bruno Dubois, Anette Schrag
Part II: Matthew B. Stern (chair), Anthony E. Lang, Peter A. LeWitt
Part III: Stanley Fahn (chair), Joseph Jankovic, C. Warren Olanow
Part IV: Pablo Martinez-Martin (chair), Andrew Lees, Olivier Rascol, Bob van Hilten
Development Standards: Glenn T. Stebbins (chair), Robert Holloway, David Nyenhuis
Appendices: Cristina Sampaio (chair), Richard Dodel, Jaime Kulisevsky
Statistical Testing: Barbara Tilley (chair), Sue Leurgans, Jean Teresi,
Consultant: Stephanie Shaftman, Nancy LaPelle

Contact person: Christopher G. Goetz, MD
Rush University Medical Center
1725 W. Harrison Street, Suite 755
Chicago, IL USA 60612

Telephone 312-942-8016
Email: cgoetz@rush.edu

July 1, 2008

Copyright © 2008 Movement Disorder Society. All rights reserved.
This chart may not be copied, distributed or otherwise used in whole or in part without prior written consent of the Movement Disorder Society.

Part I: Non-Motor Aspects of Experiences of Daily Living (nM-EDL)

Overview: This portion of the scale assesses the non-motor impact of Parkinson's disease (PD) on patients' experiences of daily living. There are 13 questions. Part 1A is administered by the rater (six questions) and focuses on complex behaviors. Part 1B is a component of the self-administered Patient Questionnaire that covers seven questions on non-motor experiences of daily living.

Part 1A:
In administering Part IA, the examiner should use the following guidelines:

1. Mark at the top of the form the primary data source as patient, caregiver, or patient and caregiver in equal proportion.
2. The response to each item should refer to a period encompassing the prior week including the day on which the information is collected.
3. All items must have an integer rating (no half points, no missing scores). In the event that an item does not apply or cannot be rated (e.g., amputee who cannot walk), the item is marked UR for Unable to Rate.
4. The answers should reflect the usual level of function and words such as "usually", "generally", "most of the time" can be used with patients.
5. Each question has a text for you to read (Instructions to patients/caregiver). After that statement, you can elaborate and probe based on the target symptoms outlined in the Instructions to examiner. You should NOT READ the RATING OPTIONS to the patient/caregiver, because these are written in medical terminology. From the interview and probing, you will use your medical judgment to arrive at the best response.
6. Patients may have co-morbidities and other medical conditions that can affect their function. You and the patient must rate the problem as it exists and do not attempt to separate elements due to Parkinson's disease from other conditions.

EXAMPLE OF NAVIGATING THROUGH THE RESPONSE OPTIONS FOR PART 1A

Suggested strategies for obtaining the most accurate answer:
After reading the instructions to the patient, you will need to probe the entire domain under discussion to determine Normal vs. problematic: If your questions do not identify any problem in this domain, record 0 and move on to the next question.

If your questions identify a problem in this domain, you should work next with a reference anchor at the mid-range (option 2 or Mild) to find out if the patient functions at this level, better or worse. You will not be reading the choices of responses to the patient as the responses use clinical terminology. You will be asking enough probing questions to determine the response that should be coded.

Work up and down the options with the patient to identify the most accurate response, giving a final check by excluding the options above and below the selected response.

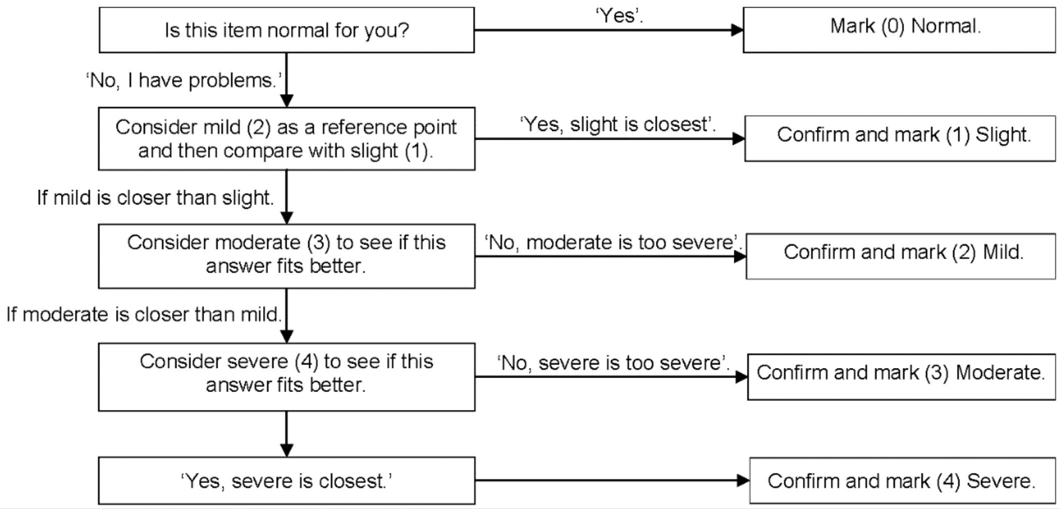

July 1, 2008 *Copyright © 2008 Movement Disorder Society. All rights reserved.* Page 2
This chart may not be copied, distributed or otherwise used in whole or in part without prior written consent of the Movement Disorder Society.

___ ___ ___ ___ ___ ___ ___ ___ Patient Name or Subject ID	___ ___ ___ Site ID	___ ___ - ___ ___ - ___ ___ ___ ___ (mm-dd-yyyy) Assessment Date	___ ___ ___ Investigator's Initials

MDS UPDRS
Part I: Non-Motor Aspects of Experiences of Daily Living (nM-EDL)

Part 1A: Complex behaviors: [completed by rater]

Primary source of information:

☐ Patient ☐ Caregiver ☐ Patient and Caregiver in Equal Proportion

To be read to the patient: I am going to ask you six questions about behaviors that you may or may not experience. Some questions concern common problems and some concern uncommon ones. If you have a problem in one of the areas, please choose the best response that describes how you have felt MOST OF THE TIME during the PAST WEEK. If you are not bothered by a problem, you can simply respond NO. I am trying to be thorough, so I may ask questions that have nothing to do with you.

1.1 COGNITIVE IMPAIRMENT **SCORE**

Instructions to examiner: Consider all types of altered level of cognitive function including cognitive slowing, impaired reasoning, memory loss, deficits in attention and orientation. Rate their impact on activities of daily living as perceived by the patient and/or caregiver.

Instructions to patients [and caregiver]: Over the past week have you had problems remembering things, following conversations, paying attention, thinking clearly, or finding your way around the house or in town? [If yes, examiner asks patient or caregiver to elaborate and probes for information]

0: Normal:	No cognitive impairment.	
1: Slight:	Impairment appreciated by patient or caregiver with no concrete interference with the patient's ability to carry out normal activities and social interactions.	☐
2: Mild:	Clinically evident cognitive dysfunction, but only minimal interference with the patient's ability to carry out normal activities and social interactions.	
3: Moderate:	Cognitive deficits interfere with but do not preclude the patient's ability to carry out normal activities and social interactions.	
4: Severe:	Cognitive dysfunction precludes the patient's ability to carry out normal activities and social interactions.	

Copyright © 2008 Movement Disorder Society. All rights reserved.

This chart may not be copied, distributed or otherwise used in whole or in part without prior written consent of the Movement Disorder Society.

	SCORE
**1.2 HALLUCINATIONS AND PSYCHOSIS**<br><br><u>Instructions to examiner:</u> Consider both illusions (misinterpretations of real stimuli) and hallucinations (spontaneous false sensations). Consider all major sensory domains (visual, auditory, tactile, olfactory and gustatory). Determine presence of unformed (for example sense of presence or fleeting false impressions) as well as formed (fully developed and detailed) sensations. Rate the patients insight into hallucinations and identify delusions and psychotic thinking.<br><br>*<u>Instructions to patients [and caregiver]:</u> Over the past week have you seen, heard, smelled or felt things that were not really there?* [If yes, examiner asks patient or caregiver to elaborate and probes for information]<br><br>0: Normal: No hallucinations or psychotic behaviour.<br>1: Slight: Illusions or non-formed hallucinations, but patient recognizes them without loss of insight.<br>2: Mild: Formed hallucinations independent of environmental stimuli. No loss of insight.<br>3: Moderate: Formed hallucinations with loss of insight.<br>4: Severe: Patient has delusions or paranoia.	☐
**1.3 DEPRESSED MOOD**<br><br><u>Instructions to examiner</u>: Consider low mood, sadness, hopelessness, feelings of emptiness or loss of enjoyment. Determine their presence and duration over the past week and rate their interference with the patient's ability to carry out daily routines and engage in social interactions.<br><br>*<u>Instruction to the patient (and caregiver)</u>: Over the past week have you felt low, sad, hopeless or unable to enjoy things? If yes, was this feeling for longer than one day at a time? Did it make it difficult for you carry out your usual activities or to be with people?* If yes, examiner asks patient or caregiver to elaborate and probes for information]<br><br>0: Normal: No depressed mood.<br>1: Slight: Episodes of depressed mood that are not sustained for more than one day at a time. No interference with patient's ability to carry out normal activities and social interactions.<br>2: Mild: Depressed mood that is sustained over days, but without interference with normal activities and social interactions.<br>3: Moderate: Depressed mood that interferes with, but does not preclude, the patient's ability to carry out normal activities and social interactions.<br>4: Severe: Depressed mood precludes patient's ability to carry out normal activities and social interactions.	☐

July 1, 2008 *Copyright © 2008 Movement Disorder Society. All rights reserved.* Page 4
This chart may not be copied, distributed or otherwise used in whole or in part without prior written consent of the Movement Disorder Society.

	SCORE
**1.4 ANXIOUS MOOD**<br><br><u>Instructions to examiner:</u> Determine nervous, tense, worried or anxious feelings (including panic attacks) over the past week and rate their duration and interference with the patient's ability to carry out daily routines and engage in social interactions.<br><br>*<u>Instructions to patients [and caregiver]:</u> Over the past week have you felt nervous, worried or tense? If yes, was this feeling for longer than one day at a time? Did it make it difficult for you to follow your usual activities or to be with other people*? [If yes, examiner asks patient or caregiver to elaborate and probes for information.]<br><br>0: Normal: No anxious feelings.<br><br>1: Slight: Anxious feelings present but not sustained for more than one day at a time. No interference with patient's ability to carry out normal activities and social interactions.<br><br>2: Mild: Anxious feelings are sustained over more than one day at a time, but without interference with patient's ability to carry out normal activities and social interactions.<br><br>3: Moderate: Anxious feelings interfere with, but do not preclude, the patient's ability to carry out normal activities and social interactions.<br><br>4: Severe: Anxious feelings preclude patient's ability to carry out normal activities and social interactions.	☐
**1.5 APATHY**<br><br><u>Instructions to examiner:</u> Consider level of spontaneous activity, assertiveness, motivation and initiative and rate the impact of reduced levels on performance of daily routines and social interactions. Here the examiner should attempt to distinguish between apathy and similar symptoms that are best explained by depression.<br><br>*<u>Instructions to patients (and caregiver):</u> Over the past week, have you felt indifferent to doing activities or being with people?* If yes, examiner asks patient or caregiver to elaborate and probes for information.]<br><br>0: Normal: No apathy.<br><br>1: Slight: Apathy appreciated by patient and/or caregiver, but no interference with daily activities and social interactions.<br><br>2: Mild: Apathy interferes with isolated activities and social interactions.<br><br>3: Moderate: Apathy interferes with most activities and social interactions.<br><br>4: Severe: Passive and withdrawn, complete loss of initiative.	☐

Copyright © 2008 Movement Disorder Society. All rights reserved.
This chart may not be copied, distributed or otherwise used in whole or in part without prior written consent of the Movement Disorder Society.

SCORE

1.6 FEATURES OF DOPAMINE DYSREGULATION SYNDROME

Instructions to examiner: Consider involvement in a variety of activities including atypical or excessive gambling (e.g. casinos or lottery tickets), atypical or excessive sexual drive or interests (e.g., unusual interest in pornography, masturbation, sexual demands on partner), other repetitive activities (e.g. hobbies, dismantling objects, sorting or organizing), or taking extra non-prescribed medication for non-physical reasons (i.e., addictive behavior). Rate the impact of such abnormal activities/behaviors on the patient's personal life and on his family and social relations (including need to borrow money or other financial difficulties like withdrawal of credit cards, major family conflicts, lost time from work, or missed meals or sleep because of the activity).

Instructions to patients [and caregiver]: Over the past week, have you had unusually strong urges that are hard to control? Do you feel driven to do or think about something and find it hard to stop? [Give patient examples such as gambling, cleaning, using the computer, taking extra medicine, obsessing about food or sex, all depending on the patients.

0: Normal: No problems present.

1: Slight: Problems are present but usually do not cause any difficulties for the patient or family/caregiver.

2: Mild: Problems are present and usually cause a few difficulties in the patient's personal and family life.

3: Moderate: Problems are present and usually cause a lot of difficulties in the patient's personal and family life.

4: Severe: Problems are present and preclude the patient's ability to carry out normal activities or social interactions or to maintain previous standards in personal and family life.

☐

The remaining questions in Part I (Non-motor Experiences of Daily Living) [Sleep, Daytime Sleepiness, Pain and Other Sensation, Urinary Problems, Constipation Problems, Lightheadedness on Standing, and Fatigue] are in the **Patient Questionnaire** along with all questions in Part II [Motor Experiences of Daily Living].

Copyright © 2008 Movement Disorder Society. All rights reserved.

This chart may not be copied, distributed or otherwise used in whole or in part without prior written consent of the Movement Disorder Society.

Patient Questionnaire:

Instructions:

This questionnaire will ask you about your experiences of daily living.

There are 20 questions. We are trying to be thorough, and some of these questions may therefore not apply to you now or ever. If you do not have the problem, simply mark 0 for NO.

Please read each one carefully and read all answers before selecting the one that best applies to you.

We are interested in your average or usual function over the past week including today. Some patients can do things better at one time of the day than at others. However, only one answer is allowed for each question, so please mark the answer that best describes what you can do most of the time.

You may have other medical conditions besides Parkinson's disease. Do not worry about separating Parkinson's disease from other conditions. Just answer the question with your best response.

Use only 0, 1, 2, 3, 4 for answers, nothing else. Do not leave any blanks.

Your doctor or nurse can review the questions with you, but this questionnaire is for patients to complete, either alone or with their caregivers.

Who is filling out this questionnaire (check the best answer):

☐ Patient ☐ Caregiver ☐ Patient and Caregiver in Equal Proportion

Copyright © 2008 Movement Disorder Society. All rights reserved.
This chart may not be copied, distributed or otherwise used in whole or in part without prior written consent of the Movement Disorder Society.

Part I: Non-Motor Aspects of Experiences of Daily Living (nM-EDL)	
**1.7 SLEEP PROBLEMS**<br><br>Over the past week, have you had trouble going to sleep at night or staying asleep through the night? Consider how rested you felt after waking up in the morning.<br><br>0: Normal: No problems.<br><br>1: Slight: Sleep problems are present but usually do not cause trouble getting a full night of sleep.<br><br>2: Mild: Sleep problems usually cause some difficulties getting a full night of sleep.<br><br>3: Moderate: Sleep problems cause a lot of difficulties getting a full night of sleep, but I still usually sleep for more than half the night.<br><br>4: Severe: I usually do not sleep for most of the night.	**SCORE**<br><br>☐
**1.8 DAYTIME SLEEPINESS**<br><br>Over the past week, have you had trouble staying awake during the daytime?<br><br>0: Normal: No daytime sleepiness.<br><br>1: Slight: Daytime sleepiness occurs but I can resist and I stay awake.<br><br>2: Mild: Sometimes I fall asleep when alone and relaxing. For example, while reading or watching TV.<br><br>3: Moderate: I sometimes fall asleep when I should not. For example, while eating or talking with other people.<br><br>4: Severe: I often fall asleep when I should not. For example, while eating or talking with other people.	☐

Copyright © 2008 Movement Disorder Society. All rights reserved.
This chart may not be copied, distributed or otherwise used in whole or in part without prior written consent of the Movement Disorder Society.

	SCORE
**1.9 PAIN AND OTHER SENSATIONS**<br><br>Over the past week, have you had uncomfortable feelings in your body like pain, aches tingling or cramps?<br><br>0: Normal: No uncomfortable feelings.<br><br>1: Slight: I have these feelings. However, I can do things and be with other people without difficulty.<br><br>2: Mild: These feelings cause some problems when I do things or am with other people.<br><br>3: Moderate: These feelings cause a lot of problems, but they do not stop me from doing things or being with other people.<br><br>4: Severe: These feelings stop me from doing things or being with other people.	☐
**1.10 URINARY PROBLEMS**<br><br>Over the past week, have you had trouble with urine control? For example, an urgent need to urinate, a need to urinate too often, or urine accidents?<br><br>0: Normal: No urine control problems.<br><br>1: Slight: I need to urinate often or urgently. However, these problems do not cause difficulties with my daily activities.<br><br>2: Mild: Urine problems cause some difficulties with my daily activities. However, I do not have urine accidents.<br><br>3: Moderate: Urine problems cause a lot of difficulties with my daily activities, including urine accidents.<br><br>4: Severe: I cannot control my urine and use a protective garment or have a bladder tube.	☐

Copyright © 2008 Movement Disorder Society. All rights reserved.
This chart may not be copied, distributed or otherwise used in whole or in part without prior written consent of the Movement Disorder Society.

	SCORE
**1.11 CONSTIPATION PROBLEMS**<br><br>Over the past week have you had constipation troubles that cause you difficulty moving your bowels?<br><br>0: Normal: No constipation.<br><br>1: Slight: I have been constipated. I use extra effort to move my bowels. However, this problem does not disturb my activities or my being comfortable.<br><br>2: Mild: Constipation causes me to have some troubles doing things or being comfortable.<br><br>3: Moderate: Constipation causes me to have a lot of trouble doing things or being comfortable. However, it does not stop me from doing anything.<br><br>4: Severe: I usually need physical help from someone else to empty my bowels.	☐
**1.12 LIGHT HEADEDNESS ON STANDING**<br><br>Over the past week, have you felt faint, dizzy or foggy when you stand up after sitting or lying down?<br><br>0: Normal: No dizzy or foggy feelings.<br><br>1: Slight: Dizzy or foggy feelings occur. However, they do not cause me troubles doing things.<br><br>2: Mild: Dizzy or foggy feelings cause me to hold on to something, but I do not need to sit or lie back down.<br><br>3: Moderate: Dizzy or foggy feelings cause me to sit or lie down to avoid fainting or falling.<br><br>4: Severe: Dizzy or foggy feelings cause me to fall or faint.	☐

Copyright © 2008 Movement Disorder Society. All rights reserved.

This chart may not be copied, distributed or otherwise used in whole or in part without prior written consent of the Movement Disorder Society.

SCORE

1.13 FATIGUE

Over the past week, have you usually felt fatigued? This feeling is not part of being sleepy or sad

0: Normal: No fatigue.

1: Slight: Fatigue occurs. However it does not cause me troubles doing things or being with people.

2: Mild: Fatigue causes me some troubles doing things or being with people.

3: Moderate: Fatigue causes me a lot of troubles doing things or being with people. However, it does not stop me from doing anything.

4: Severe: Fatigue stops me from doing things or being with people.

☐

Part II: Motor Aspects of Experiences of Daily Living (M-EDL)

2.1 SPEECH

Over the past week, have you had problems with your speech?

0: Normal: Not at all (no problems).

1: Slight: My speech is soft, slurred or uneven, but it does not cause others to ask me to repeat myself.

2: Mild: My speech causes people to ask me to occasionally repeat myself, but not everyday.

3: Moderate: My speech is unclear enough that others ask me to repeat myself every day even though most of my speech is understood.

4: Severe: Most or all of my speech cannot be understood.

☐

Copyright © 2008 Movement Disorder Society. All rights reserved.

This chart may not be copied, distributed or otherwise used in whole or in part without prior written consent of the Movement Disorder Society.

	SCORE
**2.2 SALIVA & DROOLING**<br><br>Over the past week, have you usually had too much saliva during when you are awake or when you sleep?<br><br>0: Normal: Not at all (no problems).<br>1: Slight: I have too much saliva, but do not drool.<br>2: Mild: I have some drooling during sleep, but none when I am awake.<br>3: Moderate: I have some drooling when I am awake, but I usually do not need tissues or a handkerchief.<br>4: Severe: I have so much drooling that I regularly need to use tissues or a handkerchief to protect my clothes.	☐
**2.3 CHEWING AND SWALLOWING**<br><br>Over the past week, have you usually had problems swallowing pills or eating meals? Do you need your pills cut or crushed or your meals to be made soft, chopped or blended to avoid choking?<br><br>0: Normal: No problems.<br>1: Slight: I am aware of slowness in my chewing or increased effort at swallowing, but I do not choke or need to have my food specially prepared.<br>2: Mild: I need to have my pills cut or my food specially prepared because of chewing or swallowing problems, but I have not choked over the past week.<br>3: Moderate. I choked at least once in the past week.<br>4: Severe: Because of chewing and swallowing problems, I need a feeding tube.	☐

Copyright © 2008 Movement Disorder Society. All rights reserved.

This chart may not be copied, distributed or otherwise used in whole or in part without prior written consent of the Movement Disorder Society.

SCORE

2.4 EATING TASKS

Over the past week, have you usually had troubles handling your food and using eating utensils? For example, do you have trouble handling finger foods or using forks, knifes, spoons, chopsticks?

0:	Normal:	Not at all (No problems).
1:	Slight:	I am slow, but I do not need any help handling my food and have not had food spills while eating.
2:	Mild:	I am slow with my eating and have occasional food spills. I may need help with a few tasks such as cutting meat.
3:	Moderate:	I need help with many eating tasks but can manage some alone.
4:	Severe:	I need help for most or all eating tasks.

☐

2.5 DRESSING

Over the past week, have you usually had problems dressing? For example, are you slow or do you need help with buttoning, using zippers, putting on or taking off your clothes or jewelry?

0:	Normal:	Not at all (no problems).
1:	Slight:	I am slow but I do not need help.
2:	Mild:	I am slow and need help for a few dressing tasks (buttons, bracelets).
3:	Moderate:	I need help for many dressing tasks.
4:	Severe:	I need help for most or all dressing tasks.

☐

Copyright © 2008 Movement Disorder Society. All rights reserved.
This chart may not be copied, distributed or otherwise used in whole or in part without prior written consent of the Movement Disorder Society.

	SCORE
**2.6 HYGIENE**<br><br>Over the past week, have you usually been slow or do you need help with washing, bathing, shaving, brushing teeth, combing your hair or with other personal hygiene?<br><br>0: Normal: Not at all (no problems).<br>1: Slight: I am slow but I do not need any help.<br>2: Mild: I need someone else to help me with some hygiene tasks.<br>3: Moderate: I need help for many hygiene tasks.<br>4: Severe: I need help for most or all of my hygiene tasks.	☐
**2.7 HANDWRITING**<br><br>Over the past week, have people usually had trouble reading your handwriting?<br><br>0: Normal: Not at all (no problems).<br>1: Slight: My writing is slow, clumsy or uneven, but all words are clear.<br>2: Mild: Some words are unclear and difficult to read.<br>3: Moderate: Many words are unclear and difficult to read.<br>4: Severe: Most or all words cannot be read.	☐
**2.8 DOING HOBBIES AND OTHER ACTIVITIES**<br><br>Over the past week, have you usually had trouble doing your hobbies or other things that you like to do?<br><br>0: Normal: Not at all (no problems).<br>1: Slight: I am a bit slow but do these activities easily.<br>2: Mild: I have some difficulty doing these activities.<br>3: Moderate: I have major problems doing these activities, but still do most.<br>4: Severe: I am unable to do most or all of these activities.	☐

Copyright © 2008 Movement Disorder Society. All rights reserved.
This chart may not be copied, distributed or otherwise used in whole or in part without prior written consent of the Movement Disorder Society.

	SCORE
**2.9 TURNING IN BED**<br><br>Over the past week, do you usually have trouble turning over in bed?<br><br>0: Normal: Not at all (no problems).<br>1: Slight: I have a bit of trouble turning, but I do not need any help.<br>2: Mild I have a lot of trouble turning and need occasional help from someone else.<br>3: Moderate: To turn over I often need help from someone else.<br>4: Severe: I am unable to turn over without help from someone else.	☐
**2.10 TREMOR**<br><br>Over the past week, have you usually had shaking or tremor?<br><br>0: Normal: Not at all. I have no shaking or tremor.<br>1: Slight: Shaking or tremor occurs but does not cause problems with any activities.<br>2: Mild: Shaking or tremor causes problems with only a few activities.<br>3: Moderate: Shaking or tremor causes problems with many of my daily activities.<br>4: Severe: Shaking or tremor causes problems with most or all activities.	☐
**2.11 GETTING OUT OF BED, A CAR, OR A DEEP CHAIR**<br><br>Over the past week, have you usually had trouble getting out of bed, a car seat, or a deep chair?<br><br>0: Normal: Not at all (no problems).<br>1: Slight: I am slow or awkward, but I usually can do it on my first try.<br>2: Mild: I need more than one try to get up or need occasional help.<br>3: Moderate: I sometimes need help to get up, but most times I can still do it on my own.<br>4: Severe: I need help most or all of the time.	☐

Copyright © 2008 Movement Disorder Society. All rights reserved.

This chart may not be copied, distributed or otherwise used in whole or in part without prior written consent of the Movement Disorder Society.

	SCORE
**2.12 WALKING AND BALANCE**<br><br>Over the past week, have you usually had problems with balance and walking?<br><br>0: Normal: Not at all (no problems).<br>1: Slight: I am slightly slow or may drag a leg. I never use a walking aid.<br>2: Mild: I occasionally use a walking aid, but I do not need any help from another person.<br>3: Moderate: I usually use a walking aid (cane, walker) to walk safely without falling. However, I do not usually need the support of another person.<br>4: Severe: I usually use the support of another persons to walk safely without falling.	☐
**2.13 FREEZING**<br><br>Over the past week, on your usual day when walking, do you suddenly stop or freeze as if your feet are stuck to the floor.<br><br>0: Normal: Not at all (no problems).<br>1: Slight: I briefly freeze but I can easily start walking again. I do not need help from someone else or a walking aid (cane or walker) because of freezing.<br>2: Mild: I freeze and have trouble starting to walk again, but I do not need someone's help or a walking aid (cane or walker) because of freezing.<br>3: Moderate: When I freeze I have a lot of trouble starting to walk again and, because of freezing, I sometimes need to use a walking aid or need someone else's help.<br>4: Severe: Because of freezing, most or all of the time, I need to use a walking aid or someone's help.	☐

This completes the questionnaire. We may have asked about problems you do not even have, and may have mentioned problems that you may never develop at all. Not all patients develop all these problems, but because they can occur, it is important to ask all the questions to every patient. Thank you for your time and attention in completing this questionnaire.

Copyright © 2008 Movement Disorder Society. All rights reserved.
This chart may not be copied, distributed or otherwise used in whole or in part without prior written consent of the Movement Disorder Society.

Part III: Motor Examination

Overview: This portion of the scale assesses the motor signs of PD. In administering Part III of the MDS-UPDRS the examiner should comply with the following guidelines:

At the top of the form, mark whether the patient is on medication for treating the symptoms of Parkinson's disease and, if on levodopa, the time since the last dose.

Also, if the patient is receiving medication for treating the symptoms of Parkinson's Disease, mark the patient's clinical state using the following definitions:

ON is the typical functional state when patients are receiving medication and have a good response.
OFF is the typical functional state when patients have a poor response in spite of taking medications.

The investigator should "rate what you see". Admittedly, concurrent medical problems such as stroke, paralysis, arthritis, contracture, and orthopedic problems such as hip or knee replacement and scoliosis may interfere with individual items in the motor examination. In situations where it is absolutely impossible to test (e.g., amputations, plegia, limb in a cast), use the notation "**UR**" for Unable to Rate. Otherwise, rate the performance of each task as the patient performs in the context of co-morbidities.

All items must have an integer rating (no half points, no missing ratings).

Specific instructions are provided for the testing of each item. These should be followed in all instances. The investigator demonstrates while describing tasks the patient is to perform and rates function immediately thereafter. For Global Spontaneous Movement and Rest Tremor items (3.14 and 3.17), these items have been placed purposefully at the end of the scale because clinical information pertinent to the score will be obtained throughout the entire examination.

At the end of the rating, indicate if dyskinesia (chorea or dystonia) was present at the time of the examination, and if so, whether these movements interfered with the motor examination.

3a Is the patient on medication for treating the symptoms of Parkinson's Disease? ☐ No ☐ Yes

3b If the patient is receiving medication for treating the symptoms of Parkinson's Disease, mark the patient's clinical state using the following definitions:

☐ ON: On is the typical functional state when patients are receiving medication and have a good response.

☐ OFF: Off is the typical functional state when patients have a poor response in spite of taking medications.

3c Is the patient on Levodopa ? ☐ No ☐ Yes

3.C1 If yes, minutes since last levodopa dose: __________

Copyright © 2008 Movement Disorder Society. All rights reserved.

This chart may not be copied, distributed or otherwise used in whole or in part without prior written consent of the Movement Disorder Society.

	SCORE
**3.1 SPEECH**<br><br>Instructions to examiner: Listen to the patient's free-flowing speech and engage in conversation if necessary. Suggested topics: ask about the patient's work, hobbies, exercise, or how he got to the doctor's office. Evaluate volume, modulation (prosody) and clarity, including slurring, palilalia (repetition of syllables) and tachyphemia (rapid speech, running syllables together).<br><br>0: Normal: No speech problems.<br>1: Slight: Loss of modulation, diction or volume, but still all words easy to understand.<br>2: Mild: Loss of modulation, diction, or volume, with a few words unclear, but the overall sentences easy to follow.<br>3: Moderate: Speech is difficult to understand to the point that some, but not most, sentences are poorly understood.<br>4: Severe: Most speech is difficult to understand or unintelligible.	☐
**3.2 FACIAL EXPRESSION**<br><br>Instructions to examiner: Observe the patient sitting at rest for 10 seconds, without talking and also while talking. Observe eye-blink frequency, masked facies or loss of facial expression, spontaneous smiling and parting of lips.<br><br>0: Normal: Normal facial expression.<br>1: Slight: Minimal masked facies manifested only by decreased frequency of blinking.<br>2: Mild: In addition to decreased eye-blink frequency, Masked facies present in the lower face as well, namely fewer movements around the mouth, such as less spontaneous smiling, but lips not parted.<br>3: Moderate: Masked facies with lips parted some of the time when the mouth is at rest.<br>4: Severe: Masked facies with lips parted most of the time when the mouth is at rest.	☐

Copyright © 2008 Movement Disorder Society. All rights reserved.
This chart may not be copied, distributed or otherwise used in whole or in part without prior written consent of the Movement Disorder Society.

	SCORE
**3.3 RIGIDITY**<br><br><u>Instructions to examiner</u>: Rigidity is judged on slow passive movement of major joints with the patient in a relaxed position and the examiner manipulating the limbs and neck. First, test without an activation maneuver. Test and rate neck and each limb separately. For arms, test the wrist and elbow joints simultaneously. For legs, test the hip and knee joints simultaneously. If no rigidity is detected, use an activation maneuver such as tapping fingers, fist opening/closing, or heel tapping in a limb not being tested. Explain to the patient to go as limp as possible as you test for rigidity.<br><br>0: Normal: No rigidity.<br>1: Slight: Rigidity only detected with activation maneuver.<br>2: Mild: Rigidity detected without the activation maneuver, but full range of motion is easily achieved.<br>3: Moderate: Rigidity detected without the activation maneuver; full range of motion is achieved with effort.<br>4: Severe: Rigidity detected without the activation maneuver and full range of motion not achieved.	☐ Neck<br>☐ RUE<br>☐ LUE<br>☐ RLE<br>☐ LLE
**3.4 FINGER TAPPING**<br><br><u>Instructions to examiner</u>: Each hand is tested separately. Demonstrate the task, but do not continue to perform the task while the patient is being tested. Instruct the patient to tap the index finger on the thumb 10 times as quickly AND as big as possible. Rate each side separately, evaluating speed, amplitude, hesitations, halts and decrementing amplitude.<br><br>0: Normal: No problems.<br>1: Slight: Any of the following: a) the regular rhythm is broken with one or two interruptions or hesitations of the tapping movement; b) slight slowing; c) the amplitude decrements near the end of the 10 taps.<br>2: Mild: Any of the following: a) 3 to 5 interruptions during tapping; b) mild slowing; c) the amplitude decrements midway in the 10-tap sequence.<br>3: Moderate: Any of the following: a) more than 5 interruptions during tapping or at least one longer arrest (freeze) in ongoing movement; b) moderate slowing; c) the amplitude decrements starting after the 1st tap.<br>4: Severe: Cannot or can only barely perform the task because of slowing, interruptions or decrements.	☐ R<br>☐ L

Copyright © 2008 Movement Disorder Society. All rights reserved.

This chart may not be copied, distributed or otherwise used in whole or in part without prior written consent of the Movement Disorder Society.

3.5 HAND MOVEMENTS

SCORE

Instructions to examiner: Test each hand separately. Demonstrate the task, but do not continue to perform the task while the patient is being tested. Instruct the patient to make a tight fist with the arm bent at the elbow so that the palm faces the examiner. Have the patient open the hand 10 times as fully AND as quickly as possible. If the patient fails to make a tight fist or to open the hand fully, remind him/her to do so. Rate each side separately, evaluating speed, amplitude, hesitations, halts and decrementing amplitude.

0: Normal: No problem.

1: Slight: Any of the following: a) the regular rhythm is broken with one or two interruptions or hesitations of the movement; b) slight slowing; c) the amplitude decrements near the end of the task.

2: Mild: Any of the following: a) 3 to 5 interruptions during the movements; b) mild slowing; c) the amplitude decrements midway in the task.

3: Moderate: Any of the following: a) more than 5 interruptions during the movement or at least one longer arrest (freeze) in ongoing movement; b) moderate slowing; c) the amplitude decrements starting after the 1st open-and-close sequence.

4: Severe: Cannot or can only barely perform the task because of slowing, interruptions or decrements.

☐ R

☐ L

3.6 PRONATION-SUPINATION MOVEMENTS OF HANDS

Instructions to examiner: Test each hand separately. Demonstrate the task, but do not continue to perform the task while the patient is being tested. Instruct the patient to extend the arm out in front of his/her body with the palms down; then to turn the palm up and down alternately 10 times as fast and as fully as possible. Rate each side separately, evaluating speed, amplitude, hesitations, halts and decrementing amplitude.

0: Normal: No problems.

1: Slight: Any of the following: a) the regular rhythm is broken with one or two interruptions or hesitations of the movement; b) slight slowing; c) the amplitude decrements near the end of the sequence.

2: Mild: Any of the following: a) 3 to 5 interruptions during the movements; b) mild slowing; c) the amplitude decrements midway in the sequence.

3: Moderate: Any of the following: a) more than 5 interruptions during the movement or at least one longer arrest (freeze) in ongoing movement; b) moderate slowing c) the amplitude decrements starting after the 1st supination-pronation sequence.

4: Severe: Cannot or can only barely perform the task because of slowing, interruptions or decrements.

☐ R

☐ L

Copyright © 2008 Movement Disorder Society. All rights reserved.

This chart may not be copied, distributed or otherwise used in whole or in part without prior written consent of the Movement Disorder Society.

SCORE

3.7 TOE TAPPING

Instructions to examiner: Have the patient sit in a straight-backed chair with arms, both feet on the floor. Test each foot separately. Demonstrate the task, but do not continue to perform the task while the patient is being tested. Instruct the patient to place the heel on the ground in a comfortable position and then tap the toes 10 times as big and as fast as possible. Rate each side separately, evaluating speed, amplitude, hesitations, halts and decrementing amplitude.

0: Normal: No problem.

1: Slight: Any of the following: a) the regular rhythm is broken with one or two interruptions or hesitations of the tapping movement; b) slight slowing; c) amplitude decrements near the end of the ten taps.

2: Mild: Any of the following: a) 3 to 5 interruptions during the tapping movements; b) mild slowing; c) amplitude decrements midway in the task.

3: Moderate: Any of the following: a) more than 5 interruptions during the tapping movements or at least one longer arrest (freeze) in ongoing movement; b) moderate slowing; c) amplitude decrements after the first tap.

4: Severe: Cannot or can only barely perform the task because of slowing, interruptions or decrements.

R

L

3.8 LEG AGILITY

Instructions to examiner: Have the patient sit in a straight-backed chair with arms. The patient should have both feet comfortably on the floor. Test each leg separately. Demonstrate the task, but do not continue to perform the task while the patient is being tested. Instruct the patient to place the foot on the ground in a comfortable position and then raise and stomp the foot on the ground 10 times as high and as fast as possible. Rate each side separately, evaluating speed, amplitude, hesitations, halts and decrementing amplitude.

0: Normal: No problems.

1: Slight: Any of the following: a) the regular rhythm is broken with one or two interruptions or hesitations of the movement; b) slight slowing; c) amplitude decrements near the end of the task.

2: Mild: Any of the following: a) 3 to 5 interruptions during the movements; b) mild slowness; c) amplitude decrements midway in the task.

3: Moderate: Any of the following: a) more than 5 interruptions during the movement or at least one longer arrest (freeze) in ongoing movement; b) moderate slowing in speed; c) amplitude decrements after the first tap.

4: Severe: Cannot or can only barely perform the task because of slowing, interruptions or decrements.

R

L

Copyright © 2008 Movement Disorder Society. All rights reserved.

This chart may not be copied, distributed or otherwise used in whole or in part without prior written consent of the Movement Disorder Society.

	SCORE
**3.9 ARISING FROM CHAIR**<br><br>Instructions to examiner: Have the patient sit in a straight-backed chair with arms, with both feet on the floor and sitting back in the chair (if the patient is not too short). Ask the patient to cross his/her arms across the chest and then to stand up. If the patient is not successful, repeat this attempt a maximum up to two more times. If still unsuccessful, allow the patient to move forward in the chair to arise with arms folded across the chest. Allow only one attempt in this situation. If unsuccessful, allow the patient to push off using his/her hands on the arms of the chair. Allow a maximum of three trials of pushing off. If still not successful, assist the patient to arise. After the patient stands up, observe the posture for item 3.13<br><br>0: Normal: No problems. Able to arise quickly without hesitation.<br><br>1: Slight: Arising is slower than normal; or may need more than one attempt; or may need to move forward in the chair to arise. No need to use the arms of the chair.<br><br>2: Mild: Pushes self up from arms of chair without difficulty.<br><br>3: Moderate: Needs to push off, but tends to fall back; or may have to try more than one time using arms of chair, but can get up without help.<br><br>4: Severe: Unable to arise without help.	☐
**3.10 GAIT**<br><br>Instructions to examiner: Testing gait is best performed by having the patient walking away from and towards the examiner so that both right and left sides of the body can be easily observed simultaneously. The patient should walk at least 10 meters (30 feet), then turn around and return to the examiner. This item measures multiple behaviors: stride amplitude, stride speed, height of foot lift, heel strike during walking, turning, and arm swing, but not freezing. Assess also for "freezing of gait" (next item 3.11) while patient is walking. Observe posture for item 3.13<br><br>0: Normal: No problems.<br><br>1: Slight: Independent walking with minor gait impairment.<br><br>2: Mild: Independent walking but with substantial gait impairment.<br><br>3: Moderate: Requires an assistance device for safe walking (walking stick, walker) but not a person.<br><br>4: Severe: Cannot walk at all or only with another person's assistance.	☐

Copyright © 2008 Movement Disorder Society. All rights reserved.

This chart may not be copied, distributed or otherwise used in whole or in part without prior written consent of the Movement Disorder Society.

	SCORE
**3.11 FREEZING OF GAIT**<br><br>Instructions to examiner: While assessing gait, also assess for the presence of any gait freezing episodes. Observe for start hesitation and stuttering movements especially when turning and reaching the end of the task. To the extent that safety permits, patients may NOT use sensory tricks during the assessment.<br><br>0: Normal: No freezing.<br><br>1: Slight: Freezes on starting, turning or walking through doorway with a single halt during any of these events, but then continues smoothly without freezing during straight walking.<br><br>2: Mild: Freezes on starting, turning or walking through doorway with more than one halt during any of these activities, but continues smoothly without freezing during straight walking.<br><br>3: Moderate: Freezes once during straight walking.<br><br>4: Severe: Freezes multiple times during straight walking.	☐
**3.12 POSTURAL STABILITY**<br><br>Instructions to examiner: The test examines the response to sudden body displacement produced by a quick, forceful pull on the shoulders while the patient is standing erect with eyes open and feet comfortably apart and parallel to each other. Test retropulsion. Stand behind the patient and instruct the patient on what is about to happen. Explain that s/he is allowed to take a step backwards to avoid falling. There should be a solid wall behind the examiner, at least 1-2 meters away to allow for the observation of the number of retropulsive steps. The first pull is an instructional demonstration and is purposely milder and not rated. The second time the shoulders are pulled briskly and forcefully towards the examiner with enough force to displace the center of gravity so that patient MUST take a step backwards. The examiner needs to be ready to catch the patient, but must stand sufficiently back so as to allow enough room for the patient to take several steps to recover independently. Do not allow the patient to flex the body abnormally forward in anticipation of the pull. Observe for the number of steps backwards or falling. Up to and including two steps for recovery is considered normal, so abnormal ratings begin with three steps. If the patient fails to understand the test, the examiner can repeat the test so that the rating is based on an assessment that the examiner feels reflects the patient's limitations rather than misunderstanding or lack of preparedness. Observe standing posture for item 3.13<br><br>0: Normal: No problems: Recovers with one or two steps.<br><br>1: Slight: 3-5 steps, but subject recovers unaided.<br><br>2: Mild: More than 5 steps, but subject recovers unaided.<br><br>3: Moderate: Stands safely, but with absence of postural response; falls if not caught by examiner.<br><br>4: Severe: Very unstable, tends to lose balance spontaneously or with just a gentle pull on the shoulders.	☐

Copyright © 2008 Movement Disorder Society. All rights reserved.
This chart may not be copied, distributed or otherwise used in whole or in part without prior written consent of the Movement Disorder Society.

	SCORE
**3.13 POSTURE**<br><br>Instructions to examiner: Posture is assessed with the patient standing erect after arising from a chair, during walking , and while being tested for postural reflexes. If you notice poor posture, tell the patient to stand up straight and see if the posture improves (see option 2 below). Rate the worst posture seen in these three observation points. Observe for flexion and side-to-side leaning.<br><br>0: Normal: No problems.<br>1: Slight: Not quite erect, but posture could be normal for older person.<br>2: Mild: Definite flexion, scoliosis or leaning to one side, but patient can correct posture to normal posture when asked to do so.<br>3: Moderate: Stooped posture, scoliosis or leaning to one side that cannot be corrected volitionally to a normal posture by the patient.<br>4: Severe: Flexion, scoliosis or leaning with extreme abnormality of posture.	☐
**3.14 GLOBAL SPONTANEITY OF MOVEMENT (BODY BRADYKINESIA)**<br><br>Instructions to examiner: This global rating combines all observations on slowness, hesitancy, and small amplitude and poverty of movement in general, including a reduction of gesturing and of crossing the legs. This assessment is based on the examiner's global impression after observing for spontaneous gestures while sitting, and the nature of arising and walking.<br><br>0: Normal: No problems.<br>1: Slight: Slight global slowness and poverty of spontaneous movements.<br>2: Mild: Mild global slowness and poverty of spontaneous movements.<br>3: Moderate: Moderate global slowness and poverty of spontaneous movements.<br>4: Severe: Severe global slowness and poverty of spontaneous movements.	☐
**3.15 POSTURAL TREMOR OF THE HANDS**<br><br>Instructions to examiner: All tremor, including re-emergent rest tremor, that is present in this posture is to be included in this rating. Rate each hand separately. Rate the highest amplitude seen. Instruct the patient to stretch the arms out in front of the body with palms down. The wrist should be straight and the fingers comfortably separated so that they do not touch each other. Observe this posture for 10 seconds.<br><br>0: Normal: No tremor.<br>1: Slight: Tremor is present but less than 1 cm in amplitude.<br>2: Mild: Tremor is at least 1 but less than 3 cm in amplitude.<br>3: Moderate: Tremor is at least 3 but less than 10 cm in amplitude.<br>4: Severe: Tremor is at least 10 cm in amplitude.	☐<br>R<br><br>☐<br>L

July 1, 2008

Copyright © 2008 Movement Disorder Society. All rights reserved.
This chart may not be copied, distributed or otherwise used in whole or in part without prior written consent of the Movement Disorder Society.

Page 24

SCORE

3.16 KINETIC TREMOR OF THE HANDS

Instructions to examiner: This is tested by the finger-to-nose maneuver. With the arm starting from the outstretched position, have the patient perform at least three finger-to-nose maneuvers with each hand reaching as far as possible to touch the examiner's finger. The finger-to-nose maneuver should be performed slowly enough not to hide any tremor that could occur with very fast arm movements. Repeat with the other hand, rating each hand separately. The tremor can be present throughout the movement or as the tremor reaches either target (nose or finger). Rate the highest amplitude seen.

0: Normal:	No tremor.	
1: Slight:	Tremor is present but less than 1 cm in amplitude.	R
2: Mild:	Tremor is at least 1 but less than 3 cm in amplitude.	
3: Moderate:	Tremor is at least 3 but less than 10 cm in amplitude.	
4: Severe:	Tremor is at least 10 cm in amplitude.	L

3.17 REST TREMOR AMPLITUDE

Instructions to examiner: This and the next item have been placed purposefully at the end of the examination to allow the rater to gather observations on rest tremor that may appear at any time during the exam, including when quietly sitting, during walking and during activities when some body parts are moving but others are at rest. Score the maximum amplitude that is seen at any time as the final score. Rate only the amplitude and not the persistence or the intermittency of the tremor.
As part of this rating, the patient should sit quietly in a chair with the hands placed on the arms of the chair (not in the lap) and the feet comfortably supported on the floor for 10 seconds with no other directives. Rest tremor is assessed separately for all four limbs and also for the lip/jaw. Rate only the maximum amplitude that is seen at any time as the final rating.

RUE

Extremity ratings

0: Normal:	No tremor.
1: Slight.:	< 1 cm in maximal amplitude.
2: Mild:	> 1 cm but < 3 cm in maximal amplitude.
3: Moderate:	3 - 10 cm in maximal amplitude.
4: Severe:	> 10 cm in maximal amplitude.

LUE

RLE

Lip/Jaw ratings

0: Normal:	No tremor.
1: Slight:	< 1 cm in maximal amplitude.
2: Mild:	> 1 cm but < 2 cm in maximal amplitude.
3: Moderate:	> 2 cm but < 3 cm in maximal amplitude.
4: Severe:	> 3 cm in maximal amplitude.

LLE

Lip/Jaw

Copyright © 2008 Movement Disorder Society. All rights reserved.

This chart may not be copied, distributed or otherwise used in whole or in part without prior written consent of the Movement Disorder Society.

3.18 CONSTANCY OF REST TREMOR

SCORE

Instructions to examiner: This item receives one rating for all rest tremor and focuses on the constancy of rest tremor during the examination period when different body parts are variously at rest. It is rated purposefully at the end of the examination so that several minutes of information can be coalesced into the rating.

0: Normal: No tremor.

1: Slight: Tremor at rest is present < 25% of the entire examination period.

2: Mild: Tremor at rest is present 26-50% of the entire examination period.

3: Moderate: Tremor at rest is present 51-75% of the entire examination period.

4: Severe: Tremor at rest is present > 75% of the entire examination period.

☐

DYSKINESIA IMPACT ON PART III RATINGS

A. Were dyskinesias (chorea or dystonia) present during examination? ☐ No ☐ Yes

B. If yes, did these movements interfere with your ratings? ☐ No ☐ Yes

HOEHN AND YAHR STAGE

0: Asymptomatic.

1: Unilateral involvement only.

2: Bilateral involvement without impairment of balance.

3: Mile to moderate involvement; some postural instability but physically independent; needs assistance to recover from pull test.

4: Severe disability; still able to walk or stand unassisted.

5: Wheelchair bound or bedridden unless aided.

☐

Copyright © 2008 Movement Disorder Society. All rights reserved.
This chart may not be copied, distributed or otherwise used in whole or in part without prior written consent of the Movement Disorder Society.

Part IV: Motor Complications

Overview and Instructions: In this section, the rater uses historical and objective information to assess two motor complications, dyskinesias and motor fluctuations that include OFF-state dystonia. Use all information from patient, caregiver, and the examination to answer the six questions that summarize function over the past week including today. As in the other sections, rate using only integers (no half points allowed) and leave no missing ratings. If the item cannot be rated, place UR for Unable to Rate. You will need to choose some answers based on percentages, and therefore you will need to establish how many hours generally are awake hours and use this figure as the denominator for "OFF" time and Dyskinesias. For "OFF dystonia", the total "Off" time will be the denominator. Operational definitions for examiner's use.

Dyskinesias: Involuntary random movements
Words that patients often recognize for dyskinesias include "irregular jerking", "wiggling", "twitching". It is essential to stress to the patient the difference between dyskinesias and tremor, a common error when patients are assessing dyskinesias.

Dystonia: contorted posture, often with a twisting component:
Words that patients often recognize for dystonia include "spasms", "cramps", "posture".

Motor fluctuation: Variable response to medication:
Words that patients often recognize for motor fluctuation include "wearing out", "wearing off", "roller-coaster effect", "on-off", "uneven medication effects".

OFF: Typical functional state when patients have a poor response in spite of taking mediation or the typical functional response when patients are on NO treatment for parkinsonism. Words that patients often recognize include "low time", "bad time", "shaking time", "slow time", "time when my medications don't work."

ON: Typical functional state when patients are receiving medication and have a good response:
Words that patients often recognize include "good time", "walking time", "time when my medications work."

A . DYSKINESIAS [exclusive of OFF-state dystonia]

4.1 TIME SPENT WITH DYSKINESIAS — SCORE: ☐

Instructions to examiner: Determine the hours in the usual waking day and then the hours of dyskinesias. Calculate the percentage. If the patient has dyskinesias in the office, you can point them out as a reference to ensure that patients and caregivers understand what they are rating. You may also use your own acting skills to enact the dyskinetic movements you have seen in the patient before or show them dyskinetic movements typical of other patients. Exclude from this question early morning and nighttime painful dystonia.

Instructions to patient [and caregiver]. Over the past week, how many hours do you usually sleep on a daily basis, including nighttime sleep and daytime napping? Alright, if you sleep ___ hrs, you are awake ____ hrs. Out of those awake hours, how many hours in total do you have wiggling, twitching or jerking movements? Do not count the times when you have tremor, which is a regular back and forth shaking or times when you have painful foot cramps or spasms in the early morning or at nighttime. I will ask about those later. Concentrate only on these types of wiggling, jerking and irregular movements. Add up all the time during the waking day when these usually occur. How many hours ____ (use this number for your calculation).

0: Normal:	No dyskinesias.	
1: Slight:	≤ 25% of waking day.	
2: Mild:	26 - 50% of waking day.	1. Total Hours Awake: _____
3: Moderate:	51 - 75% of waking day.	2. Total Hours with Dyskinesia: _____
4: Severe:	> 75% of waking day.	3. % Dyskinesia = ((2/1)*100): _____

Copyright © 2008 Movement Disorder Society. All rights reserved.
This chart may not be copied, distributed or otherwise used in whole or in part without prior written consent of the Movement Disorder Society.

4.2 FUNCTIONAL IMPACT OF DYSKINESIAS	SCORE
Instructions to examiner: Determine the degree to which dyskinesias impact on the patient's daily function in terms of activities and social interactions. Use the patient's and caregiver's response to your question and your own observations during the office visit to arrive at the best answer.<br><br>*Instructions to patient [and caregiver]: Over the past week, did you usually have trouble doing things or being with people when these jerking movements occurred? Did they stop you from doing things or from being with people?*<br><br>0: Normal: No dyskinesias or no impact by dyskinesias on activities or social interactions.<br>1: Slight: Dyskinesias impact on a few activities, but the patient usually performs all activities and participates in all social interactions during dyskinetic periods.<br>2: Mild: Dyskinesias impact on many activities, but the patient usually performs all activities and participates in all social interactions during dyskinetic periods.<br>3: Moderate: Dyskinesias impact on activities to the point that the patient usually does not perform some activities or does not usually participate in some social activities during dyskinetic episodes.<br>4: Severe: Dyskinesias impact on function to the point that the patient usually does not perform most activities or participate in most social interactions during dyskinetic episodes.	☐
**B . MOTOR FLUCTUATIONS**	
**4.3 TIME SPENT IN THE OFF STATE**<br><br>Instructions to examiner: Use the number of waking hours derived from 4.1 and determine the hours spent in the "OFF" state. Calculate the percentage. If the patient has an OFF period in the office, you can point to this state as a reference. You may also use your knowledge of the patient to describe a typical OFF period. Additionally you may use your own acting skills to enact an OFF period you have seen in the patient before or show them OFF function typical of other patients. Mark down the typical number of OFF hours, because you will need this number for completing 4.6<br><br>*Instructions to patient [and caregiver]: Some patients with Parkinson's disease have a good effect from their medications throughout their awake hours and we call that "ON" time. Other patients take their medications but still have some hours of low time, bad time, slow time or shaking time. Doctors call these low periods "OFF" time. Over the past week, you told me before that you are generally awake ____ hrs each day. Out of these awake hours, how many hours in total do you usually have this type of low level or OFF function ____ (Use this number for your calculations).*<br><br>0: Normal: No OFF time.<br>1: Slight: ≤ 25% of waking day.<br>2: Mild: 26 - 50% of waking day.<br>3: Moderate: 51 - 75% of waking day.<br>4: Severe: > 75% of waking day.<br><br>1. Total Hours Awake: _____<br>2. Total Hours OFF: _____<br>3. % OFF = ((2/1)*100): _____	☐

July 1, 2008

Copyright © 2008 Movement Disorder Society. All rights reserved.
This chart may not be copied, distributed or otherwise used in whole or in part without prior written consent of the Movement Disorder Society.

	SCORE
**4.4 FUNCTIONAL IMPACT OF FLUCTUATIONS**<br><br>Instructions to examiner: Determine the degree to which motor fluctuations impact on the patient's daily function in terms of activities and social interactions. This question concentrates on the difference between the ON state and the OFF state. If the patient has no OFF time, the rating must be 0, but if patients have very mild fluctuations, it is still possible to be rated 0 on this item if no impact on activities occurs. Use the patient's and caregiver's response to your question and your own observations during the office visit to arrive at the best answer.<br><br>*Instructions to patient [and caregiver]: Think about when those low or "OFF" periods have occurred over the past week. Do you usually have more problems doing things or being with people than compared to the rest of the day when you feel your medications working? Are there some things you usually do during a good period that you have trouble with or stop doing during a low period?*<br><br>0: Normal: No fluctuations or No impact by fluctuations on performance of activities or social interactions.<br><br>1: Slight: Fluctuations impact on a few activities, but during OFF, the patient usually performs all activities and participates in all social interactions that typically occur during the ON state.<br><br>2: Mild: Fluctuations impact many activities, but during OFF, the patient still usually performs all activities and participates in all social interactions that typically occur during the ON state.<br><br>3: Moderate: Fluctuations impact on the performance of activities during OFF to the point that the patient usually does not perform some activities or participate in some social interactions that are performed during ON periods.<br><br>4: Severe: Fluctuations impact on function to the point that, during OFF, the patient usually does not perform most activities or participate in most social interactions that are performed during ON periods.	☐
**4.5 COMPLEXITY OF MOTOR FLUCTUATIONS**<br><br>Instructions to examiner: Determine the usual predictability of OFF function whether due to dose, time of day, food intake or other factors. Use the information provided by the patients and caregiver and supplement with your own observations. You will ask if the patient can count on them always coming at a special time, mostly coming at a special time (in which case you will probe further to separate slight from mild), only sometimes coming at a special time or are they totally unpredictable? Narrowing down the percentage will allow you to find the correct answer.<br><br>*Instructions to patient [and caregiver]: For some patients, the low or "OFF" periods happen at certain times during day or when they do activities like eating or exercising. Over the past week, do you usually know when your low periods will occur? In other words, do your low periods always come at a certain time? Do they mostly come at a certain time? Do they only sometimes come at a certain time? Are your low periods totally unpredictable?"*<br><br>0: Normal: No motor fluctuations.<br><br>1: Slight: OFF times are predictable all or almost all of the time (> 75%).<br><br>2: Mild: OFF times are predictable most of the time (51-75%).<br><br>3: Moderate: OFF times are predictable some of the time (26-50%).<br><br>4: Severe: OFF episodes are rarely predictable. (≤ 25%).	☐

July 1, 2008 Page 29
Copyright © 2008 Movement Disorder Society. All rights reserved.
This chart may not be copied, distributed or otherwise used in whole or in part without prior written consent of the Movement Disorder Society.

C. "OFF" DYSTONIA

4.6 PAINFUL OFF-STATE DYSTONIA

Instructions to examiner: For patients who have motor fluctuations, determine what proportion of the OFF episodes usually includes painful dystonia? You have already determined the number of hours of "OFF" time (4.3). Of these hours, determine how many are associated with dystonia and calculate the percentage. If there is no OFF time, mark 0.

Instructions to patient [and caregiver]: In one of the questions I asked earlier, you said you generally have ___ hours of low or "OFF" time when your Parkinson's disease is under poor control. During these low or "OFF" periods, do you usually have painful cramps or spasms? Out of the total ____ hrs of this low time, if you add up all the time in a day when these painful cramps come, how many hours would this make?

0: Normal: No dystonia OR NO OFF TIME.

1: Slight: < 25% of time in OFF state.

2: Mild: 26-50% of time in OFF state.

3: Moderate: 51-75% of time in OFF state.

4: Severe: > 75% of time in OFF state.

☐

1. Total Hours Off: _____
2. Total Off Hours w/Dystonia: _____
3. % Off Dystonia = ((2/1)*100): _____

Summary statement to patient: READ TO PATIENT

This completes my rating of your Parkinson's disease. I know the questions and tasks have taken several minutes, but I wanted to be complete and cover all possibilities. In doing so, I may have asked about problems you do not even have, and I may have mentioned problems that you may never develop at all. Not all patients develop all these problems, but because they can occur, it is important to ask all the questions to every patient. Thank you for your time and attention in completing this scale with me.

July 1, 2008 *Copyright © 2008 Movement Disorder Society. All rights reserved.* Page 30
This chart may not be copied, distributed or otherwise used in whole or in part without prior written consent of the Movement Disorder Society.

______________________	___ ___ ___	___ - ___ - ___ (mm-dd-yyyy)	___ ___ ___
Patient Name or Subject ID	Site ID	Assessment Date	Investigator's Initials

MDS UPDRS Score Sheet

1.A	Source of information	☐ Patient ☐ Caregiver ☐ Patient + Caregiver	3.3b	Rigidity– RUE	
			3.3c	Rigidity– LUE	
**Part I**			3.3d	Rigidity– RLE	
1.1	Cognitive impairment		3.3e	Rigidity– LLE	
1.2	Hallucinations and psychosis		3.4a	Finger tapping– Right hand	
1.3	Depressed mood		3.4b	Finger tapping– Left hand	
1.4	Anxious mood		3.5a	Hand movements– Right hand	
1.5	Apathy		3.5b	Hand movements– Left hand	
1.6	Features of DDS		3.6a	Pronation- supination movements– Right hand	
1.6a	Who is filling out questionnaire	☐ Patient ☐ Caregiver ☐ Patient + Caregiver	3.6b	Pronation- supination movements– Left hand	
			3.7a	Toe tapping–Right foot	
1.7	Sleep problems		3.7b	Toe tapping– Left foot	
1.8	Daytime sleepiness		3.8a	Leg agility– Right leg	
1.9	Pain and other sensations		3.8b	Leg agility– Left leg	
1.10	Urinary problems		3.9	Arising from chair	
1.11	Constipation problems		3.10	Gait	
1.12	Light headedness on standing		3.11	Freezing of gait	
1.13	Fatigue		3.12	Postural stability	
**Part II**			3.13	Posture	
2.1	Speech		3.14	Global spontaneity of movement	
2.2	Saliva and drooling		3.15a	Postural tremor– Right hand	
2.3	Chewing and swallowing		3.15b	Postural tremor– Left hand	
2.4	Eating tasks		3.16a	Kinetic tremor– Right hand	
2.5	Dressing		3.16b	Kinetic tremor– Left hand	
2.6	Hygiene		3.17a	Rest tremor amplitude– RUE	
2.7	Handwriting		3.17b	Rest tremor amplitude– LUE	
2.8	Doing hobbies and other activities		3.17c	Rest tremor amplitude– RLE	
2.9	Turning in bed		3.17d	Rest tremor amplitude– LLE	
2.10	Tremor		3.17e	Rest tremor amplitude– Lip/jaw	
2.11	Getting out of bed		3.18	Constancy of rest	
2.12	Walking and balance			Were dyskinesias presen	☐ No ☐ Yes
2.13	Freezing			Did these movements interfere with ratings?	☐ No ☐ Yes
3a	Is the patient on medication?	☐ No ☐ Yes		Hoehn and Yahr Stage	
3b	Patient's clinical state	☐ Off ☐ On	**Part IV**		
3c	Is the patient on Levodopa?	☐ No ☐ Yes	4.1	Time spent with dyskinesias	
3.C1	If yes, minutes since last dose:		4.2	Functional impact of dyskinesias	
**Part III**			4.3	Time spent in the OFF state	
3.1	Speech		4.4	Functional impact of fluctuations	
3.2	Facial expression		4.5	Complexity of motor fluctuations	
3.3a	Rigidity– Neck		4.6	Painful OFF-state dystonia	

July 1, 2008

Copyright © 2008 Movement Disorder Society. All rights reserved.
This chart may not be copied, distributed or otherwise used in whole or in part without prior written consent of the Movement Disorder Society

APPENDIX E. PATIENT GLOBAL IMPRESSION OF CHANGE (PGI-C)

The subject will independently rate the following question of Patient Global Impression of Change (PGI-C) based on his/her overall impression at Visit 5 (Week 10), Visit 6 (Week 15), and Visit 7 (Week 20) or early discontinuation.

Patient Global Impression of Change:

Compared to your condition prior to your starting on this study, how much has your condition changed with your current treatment?

❑ 1	❑ 2	❑ 3	❑ 4	❑ 5	❑ 6	❑ 7
Very Much Worse	Much Worse	Minimally Worse	No Change	Minimally Improved	Much Improved	Very Much Improved

APPENDIX F. CLINICAL GLOBAL IMPRESSION OF CHANGE (CGI-C)

The Investigator rates each subject with the following question as part of Visit 5 (Week 10), Visit 6 (Week 15), and Visit 7 (Week 20) or early discontinuation:

Clinical Global Impression of Change:

In your opinion, how much has the subject’s overall condition and Parkinson’s disease symptoms changed since starting on the study?

❑ 1	❑ 2	❑ 3	❑ 4	❑ 5	❑ 6	❑ 7
Very Much Worse	Much Worse	Minimally Worse	Neutral	Minimally Improved	Much Improved	Very Much Improved

Guy W. ECDEU assessment manual for psychopharmacology publication; ADM, 76-338.

Washington DC, US. Department of health, education and welfare, 1976.

APPENDIX G. PATIENT GLOBAL IMPRESSION OF SEVERITY (PGI-S)

Patient Global Impression – Severity Scale

Severity of Illness

Considering the severity of your Parkinson’s disease, how severe is your condition at this time?

Severity Score:

❑ 1	❑ 2	❑ 3	❑ 4	❑ 5	❑ 6	❑ 7
Normal,<br>not at all ill	Borderline<br>ill	Mildly<br>ill	Moderately<br>ill	Markedly<br>ill	Severely<br>ill	Extremely<br>severely ill

APPENDIX H. CLINICAL GLOBAL IMPRESSION OF SEVERITY (CGI-S)

The Investigator will independently rate the following question of Clinical Global Impression of Severity (CGI-S) based on his/her overall impression of the study medication at Visit 1, Visit 4, and Visit 7 or early discontinuation.

Clinical Global Impression – Severity Scale

Severity of Illness

Considering your total clinical experience with this particular PD population, how ill is the patient at this time?

Severity Score:

❑ 1	❑ 2	❑ 3	❑ 4	❑ 5	❑ 6	❑ 7
Normal,<br>not at all ill	Borderline<br>ill	Mildly<br>ill	Moderately<br>ill	Markedly<br>ill	Severely<br>ill	Among the<br>most extremely<br>ill of subjects

Guy W. ECDEU assessment manual for psychopharmacology publication; ADM, 76-338.

Washington DC, US. Department of health, education and welfare, 1976.

APPENDIX I. 39-ITEM PARKINSON'S DISEASE QUESTIONNAIRE (PDQ-39)

PDQ-39 QUESTIONNAIRE

Please complete the following

Please tick one box for each question

Due to having Parkinson's disease, how often during the last month have you....		Never	Occasionally	Sometimes	Often	Always or cannot do at all
1	Had difficulty doing the leisure activities which you would like to do?	☐	☐	☐	☐	☐
2	Had difficulty looking after your home, e.g. DIY, housework, cooking?	☐	☐	☐	☐	☐
3	Had difficulty carrying bags of shopping?	☐	☐	☐	☐	☐
4	Had problems walking half a mile?	☐	☐	☐	☐	☐
5	Had problems walking 100 yards?	☐	☐	☐	☐	☐
6	Had problems getting around the house as easily as you would like?	☐	☐	☐	☐	☐
7	Had difficulty getting around in public?	☐	☐	☐	☐	☐
8	Needed someone else to accompany you when you went out?	☐	☐	☐	☐	☐
9	Felt frightened or worried about falling over in public?	☐	☐	☐	☐	☐
10	Been confined to the house more than you would like?	☐	☐	☐	☐	☐
11	Had difficulty washing yourself?	☐	☐	☐	☐	☐
12	Had difficulty dressing yourself?	☐	☐	☐	☐	☐
13	Had problems doing up your shoe laces?	☐	☐	☐	☐	☐

Please check that you have ticked ***one box for each question*** *before going on to the next page*

Due to having Parkinson's disease, how often <u>during the last month</u> have you....

Please tick <u>one</u> box for each question

		**Never**	**Occasionally**	**Sometimes**	**Often**	**Always** or cannot do at all
14	Had problems writing clearly?	☐	☐	☐	☐	☐
15	Had difficulty cutting up your food?	☐	☐	☐	☐	☐
16	Had difficulty holding a drink without spilling it?	☐	☐	☐	☐	☐
17	Felt depressed?	☐	☐	☐	☐	☐
18	Felt isolated and lonely?	☐	☐	☐	☐	☐
19	Felt weepy or tearful?	☐	☐	☐	☐	☐
20	Felt angry or bitter?	☐	☐	☐	☐	☐
21	Felt anxious?	☐	☐	☐	☐	☐
22	Felt worried about your future?	☐	☐	☐	☐	☐
23	Felt you had to conceal your Parkinson's from people?	☐	☐	☐	☐	☐
24	Avoided situations which involve eating or drinking in public?	☐	☐	☐	☐	☐
25	Felt embarrassed in public due to having Parkinson's disease?	☐	☐	☐	☐	☐
26	Felt worried by other people's reaction to you?	☐	☐	☐	☐	☐
27	Had problems with your close personal relationships?	☐	☐	☐	☐	☐
28	Lacked support in the ways you need from your spouse or partner?	☐	☐	☐	☐	☐
	If you do not have a spouse or partner tick here		☐			
29	Lacked support in the ways you need from your family or close friends?	☐	☐	☐	☐	☐

Please check that you have ticked ***one box for each question*** *before going on to the next page*

Due to having Parkinson's disease, how often during the last month have you....	***Please tick one box for each question*** Never	Occasionally	Sometimes	Often	Always
30 Unexpectedly fallen asleep during the day?	☐	☐	☐	☐	☐
31 Had problems with your concentration, e.g. when reading or watching TV?	☐	☐	☐	☐	☐
32 Felt your memory was bad?	☐	☐	☐	☐	☐
33 Had distressing dreams or hallucinations?	☐	☐	☐	☐	☐
34 Had difficulty with your speech?	☐	☐	☐	☐	☐
35 Felt unable to communicate with people properly?	☐	☐	☐	☐	☐
36 Felt ignored by people?	☐	☐	☐	☐	☐
37 Had painful muscle cramps or spasms?	☐	☐	☐	☐	☐
38 Had aches and pains in your joints or body?	☐	☐	☐	☐	☐
39 Felt unpleasantly hot or cold?	☐	☐	☐	☐	☐

Please check that you have ticked ***one box for each question*** *before going on to the next page*

Thank you for completing the PDQ 39 questionnaire

APPENDIX J. GASTROPARESIS CARDINAL SYMPTOM INDEX (GCSI)

GASTROPARESIS CARDINAL SYMPTOM INDEX

This questionnaire asks you about the severity of symptoms you may have related to your gastrointestinal problem. There are no right or wrong answers. Please answer each question as accurately as possible.

For each symptom, please circle the number that best describes how severe the symptom has been during the past 2 weeks.

- If you have not experienced this symptom, circle 0.
- If the symptom has been very mild, circle 1.
- If the symptom has been mild, circle 2.
- If it has been moderate, circle 3.
- If it has been severe, circle 4.
- If it has been very severe, circle 5.

Please be sure to answer every question.

Please rate the severity of the following symptoms during the past 2 weeks.

	None	Very mild	Mild	Moderate	Severe	Very severe
1. nausea (feeling sick to your stomach as if you were going to vomit or throw up)	0	1	2	3	4	5
2. retching (heaving as if to vomit, but nothing comes up)	0	1	2	3	4	5
3. vomiting	0	1	2	3	4	5
4. stomach fullness	0	1	2	3	4	5
5. not able to finish a normal-sized meal	0	1	2	3	4	5
6. feeling excessively full after meals	0	1	2	3	4	5
7. loss of appetite	0	1	2	3	4	5
8. bloating (feeling like you need to loosen your clothes)	0	1	2	3	4	5
9. stomach or belly visibly larger	0	1	2	3	4	5

©2002 Johnson & Johnson Pharmaceutical Services, LLC. Contact D. Dubois for permission to use.

APPENDIX K. NON-MOTOR SYMPTOM ASSESSMENT SCALE FOR PARKINSON'S DISEASE (NMSS)

Non-Motor Symptom assessment scale for Parkinson's Disease

Patient ID No: ______________________ **Initials:** _______________ **Age:** _________

Symptoms assessed over the last month. Each symptom scored with respect to:

Severity: 0 = None, 1 = Mild: symptoms present but causes little distress or disturbance to patient; 2 = Moderate: some distress or disturbance to patient; 3 = Severe: major source of distress or disturbance to patient.

Frequency: 1 = Rarely (<1/wk); 2 = Often (1/wk); 3 = Frequent (several times per week); 4 = Very Frequent (daily or all the time)

Domains will be weighed differentially. Yes/ No answers are not included in final frequency x severity calculation.
(Bracketed text in questions within the scale is included as an explanatory aid).

Domain 1: Cardiovascular including falls	Severity	Frequency	Frequency x Severity
1. Does the patient experience light-headedness, dizziness, weakness on standing from sitting or lying position?	☐	☐	☐
2. Does the patient fall because of fainting or blacking out?	☐	☐	☐
**SCORE:**			☐
**Domain 2: Sleep/fatigue**			
3. Does the patient doze off or fall asleep unintentionally during daytime activities? (For example, during conversation, during mealtimes, or while watching television or reding).	☐	☐	☐
4. Does fatigue (tiredness) or lack of energy (not slowness) limit the patient's daytime activities?	☐	☐	☐
5. Does the patient have difficulties falling or staying asleep?	☐	☐	☐
6. Does the patient experience an urge to move the legs or restlessness in legs that improves with movement when he/she is sitting or lying down inactive?	☐	☐	☐
**SCORE:**			☐
**Domain 3: Mood /Cognition**			
7. Has the patient lost interest in his/her surroundings?	☐	☐	☐
8. Has the patient lost interest in doing things or lack motivation to start new activities?	☐	☐	☐
9. Does the patient feel nervous, worried or frightened for no apparent reason?	☐	☐	☐
10. Does the patient seem sad or depressed or has he/she reported such feelings?	☐	☐	☐
11. Does the patient have flat moods without the normal "highs" and " lows"?	☐	☐	☐
12. Does the patient have difficulty in experiencing pleasure from their usual activities or report that they lack pleasure?	☐	☐	☐
**SCORE:**			☐
**Domain 4: Perceptual problems/hallucinations**			
13. Does the patient indicate that he/she sees things that are not there?	☐	☐	☐
14. Does the patient have beliefs that you know are not true? (For example, about being harmed, being robbed or being unfaithful)	☐	☐	☐
15. Does the patient experience double vision? (2 separate real objects and not blurred vision)	☐	☐	☐
**SCORE:**			☐

	Severity	Frequency	Frequency x Severity
**Domain 5: Attention/ Memory**			
16. Does the patient have problems sustaining concentration during activities? (For example, reading or having a conversation)	☐	☐	☐
17. Does the patient forget things that he/she has been told a short time ago or events that happened in the last few days?	☐	☐	☐
18. Does the patient forget to do things? (For example, take tablets or turn off domestic appliances?)	☐	☐	☐
**SCORE:**			☐
**Domain 6: Gastrointestinal tract**			
19. Does the patient dribble saliva during the day?	☐	☐	☐
20. Does the patient having difficulty swallowing?	☐	☐	☐
21. Does the patient suffer from constipation? (Bowel action less than three times weekly)	☐	☐	☐
**SCORE:**			☐
**Domain 7: Urinary**			
22. Does the patient have difficulty holding urine? (Urgency)	☐	☐	☐
23. Does the patient have to void within 2 hours of last voiding? (Frequency)	☐	☐	☐
24. Does the patient have to get up regularly at night to pass urine? (Nocturia)	☐	☐	☐
**SCORE:**			☐
**Domain 8: Sexual function**			
25. Does the patient have altered interest in sex? (Very much increased or decreased, please underline)	☐	☐	☐
26. Does the patient have problems having sex?	☐	☐	☐
**SCORE:**			☐
**Domain 9: Miscellaneous**			
27. Does the patient suffer from pain not explained by other known conditions? (Is it related to intake of drugs and is it relieved by antiparkinson drugs?)	☐	☐	☐
28. Does the patient report a change in ability to taste or smell?	☐	☐	☐
29. Does the patient report a recent change in weight (not related to dieting)?	☐	☐	☐
30. Does the patient experience excessive sweating? (not related to hot weather)	☐	☐	☐
**SCORE:**			☐

TOTAL SCORE: ☐

Developed by the International Parkinson's Disease Non- Motor Group.
Contacts: ray.chaudhuri@uhl.nhs.uk or alison.forbes@uhl.nhs.uk

APPENDIX L. PARKINSON'S DISEASE SLEEP SCALE-2 (PDSS-2)

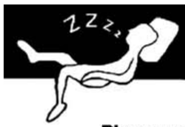

Parkinson's Disease Sleep Scale (PDSS-2)

Please rate the severity of the following based on your experiences during the past week (7 days). Please make a cross in the answer box

	Very often (This means 6 to 7 days a week)	Often (This means 4 to 5 days a week)	Sometimes (This means 2 to 3 days a week)	Occasionally (This means 1 day a week)	Never
1) Overall, did you sleep well during the last week?	☐ 0	☐ 1	☐ 2	☐ 3	☐ 4
2) Did you have difficulty falling asleep each night?	☐ 4	☐ 3	☐ 2	☐ 1	☐ 0
3) Did you have difficulty staying asleep?	☐ 4	☐ 3	☐ 2	☐ 1	☐ 0
4) Did you have restlessness of legs or arms at nights causing disruption of sleep?	☐ 4	☐ 3	☐ 2	☐ 1	☐ 0
5) Was your sleep disturbed due to an urge to move your legs or arms?	☐ 4	☐ 3	☐ 2	☐ 1	☐ 0
6) Did you suffer from distressing dreams at night?	☐ 4	☐ 3	☐ 2	☐ 1	☐ 0
7) Did you suffer from distressing hallucinations at night (seeing or hearing things that you are told do not exist)?	☐ 4	☐ 3	☐ 2	☐ 1	☐ 0
8) Did you get up at night to pass urine?	☐ 4	☐ 3	☐ 2	☐ 1	☐ 0
9) Did you feel uncomfortable at night because you were unable to turn around in bed or move due to immobility?	☐ 4	☐ 3	☐ 2	☐ 1	☐ 0
10) Did you feel pain in your arms or legs which woke you up from sleep at night?	☐ 4	☐ 3	☐ 2	☐ 1	☐ 0
11) Did you have muscle cramps in your arms or legs which woke you up whilst sleeping at night?	☐ 4	☐ 3	☐ 2	☐ 1	☐ 0
12) Did you wake early in the morning with painful posturing of arms and legs?	☐ 4	☐ 3	☐ 2	☐ 1	☐ 0
13) On waking, did you experience tremor?	☐ 4	☐ 3	☐ 2	☐ 1	☐ 0
14) Did you feel tired and sleepy after waking in the morning?	☐ 4	☐ 3	☐ 2	☐ 1	☐ 0
15) Did you wake up at night due to snoring or difficulties with breathing?	☐ 4	☐ 3	☐ 2	☐ 1	☐ 0

Trenkwalder C, Kohnen R, Högl B, Metta V, Sixel-Döring F, Frauscher B, Hülsmann J, Martinez-Martin P, Chaudhuri KR. Parkinson's disease sleep scale--validation of the revised version PDSS-2. Mov Disord. 2011;26(4):644-52.

APPENDIX M. PARKINSON ANXIETY SCALE (PAS)

The Parkinson Anxiety Scale (PAS)

(Please mark one circle for each item below)

In the past four weeks, to what extent did you experience the following symptoms?

A. Persistent Anxiety

A.1. Feeling anxious or nervous

- o Not at all, or never
- o Very mild, or rarely
- o Mild, or sometimes
- o Moderate, or often
- o Severe, or (nearly) always

A.2. Feeling tense or stressed

- o Not at all, or never
- o Very mild, or rarely
- o Mild, or sometimes
- o Moderate, or often
- o Severe, or (nearly) always

A.3. Being unable to relax

- o Not at all, or never
- o Very mild, or rarely
- o Mild, or sometimes
- o Moderate, or often
- o Severe, or (nearly) always

A.4. Excessive worrying about everyday matters

- o Not at all, or never
- o Very mild, or rarely
- o Mild, or sometimes
- o Moderate, or often
- o Severe, or (nearly) always

A.5. Fear of something bad, or even the worst, happening

- o Not at all, or never
- o Very mild, or rarely
- o Mild, or sometimes
- o Moderate, or often
- o Severe, or (nearly) always

B. Episodic Anxiety

B.1. Panic or intense fear

- o Never
- o Rarely
- o Sometimes
- o Often
- o Nearly always

B.2. Shortness of breath

- o Never
- o Rarely
- o Sometimes
- o Often
- o Nearly always

B.3. Heart palpitations or heart beating fast (not related to physical effort or activity)

- o Never
- o Rarely
- o Sometimes
- o Often
- o Nearly always

B.4. Fear of losing control

- o Never
- o Rarely
- o Sometimes
- o Often
- o Nearly always

C. Avoidance Behavior

C.1. Social situations (where one may be observed, or evaluated by others, such as speaking in public, or talking to unknown people)

- o Never
- o Rarely
- o Sometimes
- o Often
- o Nearly always

C.2. Public settings (situations from which it may be difficult or embarrassing to escape, such as queues or lines, crowds, bridges, or public transportation)

- o Never
- o Rarely
- o Sometimes
- o Often
- o Nearly always

C.3. Specific objects or situations (such as flying, heights, spiders or other animals, needles, or blood)

- o Never
- o Rarely
- o Sometimes
- o Often
- o Nearly always

Copyright of this scale and it translations is held by the authors (Leentjens AF, Dujardin K, Pontone GM, Starkstein SE, Weintraub D, and Martinez-Martin P. The Parkinson Anxiety Scale (PAS): development and validation of a new anxiety scale. Mov Disord. 2014;29(8):1035-43). The scale and its translations are in the public domain and may be used without additional permission and free of charge on the condition that its source is referenced.

APPENDIX N. COLUMBIA-SUICIDE SEVERITY RATING SCALE (C-SSRS)

COLUMBIA-SUICIDE SEVERITY RATING SCALE (C-SSRS)

Baseline/Screening Version

Version 1/14/09

Posner, K.; Brent, D.; Lucas, C.; Gould, M.; Stanley, B.; Brown, G.; Fisher, P.; Zelazny, J.; Burke, A.; Oquendo, M.; Mann, J.

Disclaimer:

This scale is intended to be used by individuals who have received training in its administration. The questions contained in the Columbia-Suicide Severity Rating Scale are suggested probes. Ultimately, the determination of the presence of suicidal ideation or behavior depends on the judgment of the individual administering the scale.

Definitions of behavioral suicidal events in this scale are based on those used in **The Columbia Suicide History Form**, *developed by John Mann, MD and Maria Oquendo, MD, Conte Center for the Neuroscience of Mental Disorders (CCNMD), New York State Psychiatric Institute, 1051 Riverside Drive, New York, NY, 10032. (Oquendo M. A., Halberstam B. & Mann J. J., Risk factors for suicidal behavior: utility and limitations of research instruments. In M.B. First [Ed.]* Standardized Evaluation in Clinical Practice, *pp. 103 -130, 2003.)*

For reprints of the C-SSRS contact Kelly Posner, Ph.D., New York State Psychiatric Institute, 1051 Riverside Drive, New York, New York, 10032; inquiries and training requirements contact posnerk@nyspi.columbia.edu

© 2008 The Research Foundation for Mental Hygiene, Inc.

SUICIDAL IDEATION		
Ask questions 1 and 2. If both are negative, proceed to "Suicidal Behavior" section. If the answer to question 2 is "yes", ask questions 3, 4 and 5. If the answer to question 1 and/or 2 is "yes", complete "Intensity of Ideation" section below.	**Lifetime: Time He/She Felt Most Suicidal**	**Past __ Months**
**1. Wish to be Dead**<br>Subject endorses thoughts about a wish to be dead or not alive anymore, or wish to fall asleep and not wake up.<br>***Have you wished you were dead or wished you could go to sleep and not wake up?***<br><br>If yes, describe:	**Yes** ☐ **No** ☐	**Yes** ☐ **No** ☐
**2. Non-Specific Active Suicidal Thoughts**<br>General non-specific thoughts of wanting to end one's life/commit suicide (e.g., *"I've thought about killing myself"*) without thoughts of ways to kill oneself/associated methods, intent, or plan during the assessment period.<br>***Have you actually had any thoughts of killing yourself?***<br><br>If yes, describe:	**Yes** ☐ **No** ☐	**Yes** ☐ **No** ☐
**3. Active Suicidal Ideation with Any Methods (Not Plan) without Intent to Act**<br>Subject endorses thoughts of suicide and has thought of at least one method during the assessment period. This is different than a specific plan with time, place or method details worked out (e.g. thought of method to kill self but not a specific plan). Includes person who would say, *"I thought about taking an overdose but I never made a specific plan as to when, where or how I would actually do it…and I would never go through with it."*<br>***Have you been thinking about how you might do this?***<br><br>If yes, describe:	**Yes** ☐ **No** ☐	**Yes** ☐ **No** ☐
**4. Active Suicidal Ideation with Some Intent to Act, without Specific Plan**<br>Active suicidal thoughts of killing oneself and subject reports having <u>some intent to act on such thoughts</u>, as opposed to *"I have the thoughts but I definitely will not do anything about them."*<br>***Have you had these thoughts and had some intention of acting on them?***<br><br>If yes, describe:	**Yes** ☐ **No** ☐	**Yes** ☐ **No** ☐
**5. Active Suicidal Ideation with Specific Plan and Intent**<br>Thoughts of killing oneself with details of plan fully or partially worked out and subject has some intent to carry it out.<br>***Have you started to work out or worked out the details of how to kill yourself? Do you intend to carry out this plan?***<br><br>If yes, describe:	**Yes** ☐ **No** ☐	**Yes** ☐ **No** ☐
INTENSITY OF IDEATION		
The following features should be rated with respect to the most severe type of ideation (i.e., 1-5 from above, with 1 being the least severe and 5 being the most severe). Ask about time he/she was feeling the most suicidal.<br><br><u>Lifetime</u> - ***Most Severe Ideation:*** ______ ______________<br>*Type # (1-5)* *Description of Ideation*<br><br><u>Past X Months</u> - ***Most Severe Ideation:*** ______ ______________<br>*Type # (1-5)* *Description of Ideation*	Most Severe	Most Severe
**Frequency**<br>***How many times have you had these thoughts?***<br>(1) Less than once a week (2) Once a week (3) 2-5 times in week (4) Daily or almost daily (5) Many times each day	___	___
**Duration**<br>***When you have the thoughts how long do they last?***<br>(1) Fleeting - few seconds or minutes<br>(2) Less than 1 hour/some of the time<br>(3) 1-4 hours/a lot of time<br>(4) 4-8 hours/most of day<br>(5) More than 8 hours/persistent or continuous	___	___
**Controllability**<br>***Could/can you stop thinking about killing yourself or wanting to die if you want to?***<br>(1) Easily able to control thoughts<br>(2) Can control thoughts with little difficulty<br>(3) Can control thoughts with some difficulty<br>(4) Can control thoughts with a lot of difficulty<br>(5) Unable to control thoughts<br>(0) Does not attempt to control thoughts	___	___
**Deterrents**<br>***Are there things - anyone or anything (e.g., family, religion, pain of death) - that stopped you from wanting to die or acting on thoughts of committing suicide?***<br>(1) Deterrents definitely stopped you from attempting suicide<br>(2) Deterrents probably stopped you<br>(3) Uncertain that deterrents stopped you<br>(4) Deterrents most likely did not stop you<br>(5) Deterrents definitely did not stop you<br>(0) Does not apply	___	___
**Reasons for Ideation**<br>***What sort of reasons did you have for thinking about wanting to die or killing yourself? Was it to end the pain or stop the way you were feeling (in other words you couldn't go on living with this pain or how you were feeling) or was it to get attention, revenge or a reaction from others? Or both?***<br>(1) Completely to get attention, revenge or a reaction from others<br>(2) Mostly to get attention, revenge or a reaction from others<br>(3) Equally to get attention, revenge or a reaction from others and to end/stop the pain<br>(4) Mostly to end or stop the pain (you couldn't go on living with the pain or how you were feeling)<br>(5) Completely to end or stop the pain (you couldn't go on living with the pain or how you were feeling)<br>(0) Does not apply	___	___

© 2008 Research Foundation for Mental Hygiene, Inc. C-SSRS—Baseline/Screening (Version 1/14/09) Page 1 of 2

SUICIDAL BEHAVIOR<br>*(Check all that apply, so long as these are separate events; must ask about all types)*	**Lifetime**	**Past __ Years**
**Actual Attempt:**<br>A potentially self-injurious act committed with at least some wish to die, *as a result of act.* Behavior was in part thought of as method to kill oneself. Intent does not have to be 100%. If there is ***any*** intent/desire to die associated with the act, then it can be considered an actual suicide attempt. ***There does not have to be any injury or harm,*** just the potential for injury or harm. If person pulls trigger while gun is in mouth but gun is broken so no injury results, this is considered an attempt.<br>Inferring Intent: Even if an individual denies intent/wish to die, it may be inferred clinically from the behavior or circumstances. For example, a highly lethal act that is clearly not an accident so no other intent but suicide can be inferred (e.g., gunshot to head, jumping from window of a high floor/story). Also, if someone denies intent to die, but they thought that what they did could be lethal, intent may be inferred.<br>***Have you made a suicide attempt?***<br>***Have you done anything to harm yourself?***<br>***Have you done anything dangerous where you could have died?***<br>***What did you do?***<br>***Did you______ as a way to end your life?***<br>***Did you want to die (even a little) when you_____?***<br>***Were you trying to end your life when you _____?***<br>***Or Did you think it was possible you could have died from_____?***<br>***Or did you do it purely for other reasons / without ANY intention of killing yourself (like to relieve stress, feel better, get sympathy, or get something else to happen)?*** (Self-Injurious Behavior without suicidal intent)<br>If yes, describe:	Yes No<br>☐ ☐<br>Total # of Attempts<br>_____	Yes No<br>☐ ☐<br>Total # of Attempts<br>_____
**Has subject engaged in Non-Suicidal Self-Injurious Behavior?**	Yes No<br>☐ ☐	Yes No<br>☐ ☐
**Interrupted Attempt:**<br>When the person is interrupted (by an outside circumstance) from starting the potentially self-injurious act *(if not for that, actual attempt would have occurred).*<br>Overdose: Person has pills in hand but is stopped from ingesting. Once they ingest any pills, this becomes an attempt rather than an interrupted attempt. Shooting: Person has gun pointed toward self, gun is taken away by someone else, or is somehow prevented from pulling trigger. Once they pull the trigger, even if the gun fails to fire, it is an attempt. Jumping: Person is poised to jump, is grabbed and taken down from ledge. Hanging: Person has noose around neck but has not yet started to hang - is stopped from doing so.<br>***Has there been a time when you started to do something to end your life but someone or something stopped you before you actually did anything?***<br>If yes, describe:	Yes No<br>☐ ☐<br>Total # of interrupted<br>_____	Yes No<br>☐ ☐<br>Total # of interrupted<br>_____
**Aborted Attempt:**<br>When person begins to take steps toward making a suicide attempt, but stops themselves before they actually have engaged in any self-destructive behavior. Examples are similar to interrupted attempts, except that the individual stops him/herself, instead of being stopped by something else.<br>***Has there been a time when you started to do something to try to end your life but you stopped yourself before you actually did anything?***<br>If yes, describe:	Yes No<br>☐ ☐<br>Total # of aborted<br>_____	Yes No<br>☐ ☐<br>Total # of aborted<br>_____
**Preparatory Acts or Behavior:**<br>Acts or preparation towards imminently making a suicide attempt. This can include anything beyond a verbalization or thought, such as assembling a specific method (e.g., buying pills, purchasing a gun) or preparing for one's death by suicide (e.g., giving things away, writing a suicide note).<br>***Have you taken any steps towards making a suicide attempt or preparing to kill yourself (such as collecting pills, getting a gun, giving valuables away or writing a suicide note)?***<br>If yes, describe:	Yes No<br>☐ ☐	Yes No<br>☐ ☐
**Suicidal Behavior:**<br>Suicidal behavior was present during the assessment period?	Yes No<br>☐ ☐	Yes No<br>☐ ☐

Answer for Actual Attempts Only	Most Recent Attempt Date:	Most Lethal Attempt Date:	Initial/First Attempt Date:
**Actual Lethality/Medical Damage:**<br>0. No physical damage or very minor physical damage (e.g., surface scratches).<br>1. Minor physical damage (e.g., lethargic speech; first-degree burns; mild bleeding; sprains).<br>2. Moderate physical damage; medical attention needed (e.g., conscious but sleepy, somewhat responsive; second-degree burns; bleeding of major vessel).<br>3. Moderately severe physical damage; *medical* hospitalization and likely intensive care required (e.g., comatose with reflexes intact; third-degree burns less than 20% of body; extensive blood loss but can recover; major fractures).<br>4. Severe physical damage; *medical* hospitalization with intensive care required (e.g., comatose without reflexes; third-degree burns over 20% of body; extensive blood loss with unstable vital signs; major damage to a vital area).<br>5. Death	*Enter Code*<br>_____	*Enter Code*<br>_____	*Enter Code*<br>_____
**Potential Lethality: Only Answer if Actual Lethality=0**<br>Likely lethality of actual attempt if no medical damage (the following examples, while having no actual medical damage, had potential for very serious lethality: put gun in mouth and pulled the trigger but gun fails to fire so no medical damage; laying on train tracks with oncoming train but pulled away before run over).<br>0 = Behavior not likely to result in injury<br>1 = Behavior likely to result in injury but not likely to cause death<br>2 = Behavior likely to result in death despite available medical care	*Enter Code*<br>_____	*Enter Code*<br>_____	*Enter Code*<br>_____

© 2008 Research Foundation for Mental Hygiene, Inc. C-SSRS—Baseline/Screening (Version 1/14/09) Page 2 of 2

COLUMBIA-SUICIDE SEVERITY RATING SCALE (C-SSRS)

Since Last Visit

Version 1/14/09

Posner, K.; Brent, D.; Lucas, C.; Gould, M.; Stanley, B.; Brown, G.; Fisher, P.; Zelazny, J.; Burke, A.; Oquendo, M.; Mann, J.

Disclaimer:

This scale is intended to be used by individuals who have received training in its administration. The questions contained in the Columbia-Suicide Severity Rating Scale are suggested probes. Ultimately, the determination of the presence of suicidal ideation or behavior depends on the judgment of the individual administering the scale.

Definitions of behavioral suicidal events in this scale are based on those used in **The Columbia Suicide History Form**, *developed by John Mann, MD and Maria Oquendo, MD, Conte Center for the Neuroscience of Mental Disorders (CCNMD), New York State Psychiatric Institute, 1051 Riverside Drive, New York, NY, 10032. (Oquendo M. A., Halberstam B. & Mann J. J., Risk factors for suicidal behavior: utility and limitations of research instruments. In M.B. First [Ed.]* Standardized Evaluation in Clinical Practice, *pp. 103 -130, 2003.)*

For reprints of the C-SSRS contact Kelly Posner, Ph.D., New York State Psychiatric Institute, 1051 Riverside Drive, New York, New York, 10032; inquiries and training requirements contact posnerk@nyspi.columbia.edu

© 2008 The Research Foundation for Mental Hygiene, Inc.

**SUICIDAL IDEATION**	
Ask questions 1 and 2. If both are negative, proceed to "Suicidal Behavior" section. If the answer to question 2 is "yes", ask questions 3, 4 and 5. If the answer to question 1 and/or 2 is "yes", complete "Intensity of Ideation" section below.	**Since Last Visit**
**1. Wish to be Dead**<br>Subject endorses thoughts about a wish to be dead or not alive anymore, or wish to fall asleep and not wake up.<br>***Have you wished you were dead or wished you could go to sleep and not wake up?***<br><br>If yes, describe:	**Yes** ☐ **No** ☐
**2. Non-Specific Active Suicidal Thoughts**<br>General, non-specific thoughts of wanting to end one's life/commit suicide (e.g., *"I've thought about killing myself"*) without thoughts of ways to kill oneself/associated methods, intent, or plan during the assessment period.<br>***Have you actually had any thoughts of killing yourself?***<br><br>If yes, describe:	**Yes** ☐ **No** ☐
**3. Active Suicidal Ideation with Any Methods (Not Plan) without Intent to Act**<br>Subject endorses thoughts of suicide and has thought of at least one method during the assessment period. This is different than a specific plan with time, place or method details worked out (e.g., thought of method to kill self but not a specific plan). Includes person who would say, *"I thought about taking an overdose but I never made a specific plan as to when, where or how I would actually do it…and I would never go through with it."*<br>***Have you been thinking about how you might do this?***<br><br>If yes, describe:	**Yes** ☐ **No** ☐
**4. Active Suicidal Ideation with Some Intent to Act, without Specific Plan**<br>Active suicidal thoughts of killing oneself and subject reports having some intent to act on such thoughts, as opposed to *"I have the thoughts but I definitely will not do anything about them."*<br>***Have you had these thoughts and had some intention of acting on them?***<br><br>If yes, describe:	**Yes** ☐ **No** ☐
**5. Active Suicidal Ideation with Specific Plan and Intent**<br>Thoughts of killing oneself with details of plan fully or partially worked out and subject has some intent to carry it out.<br>***Have you started to work out or worked out the details of how to kill yourself? Do you intend to carry out this plan?***<br><br>If yes, describe:	**Yes** ☐ **No** ☐

**INTENSITY OF IDEATION**	
The following features should be rated with respect to the most severe type of ideation (i.e., 1-5 from above, with 1 being the least severe and 5 being the most severe).<br><br>***Most Severe Ideation:*** _____ ______________________<br>***Type # (1-5)*** ***Description of Ideation***	Most Severe
**Frequency**<br>***How many times have you had these thoughts?***<br>(1) Less than once a week (2) Once a week (3) 2-5 times in week (4) Daily or almost daily (5) Many times each day	___
**Duration**<br>***When you have the thoughts, how long do they last?***<br>(1) Fleeting - few seconds or minutes<br>(2) Less than 1 hour/some of the time<br>(3) 1-4 hours/a lot of time<br>(4) 4-8 hours/most of day<br>(5) More than 8 hours/persistent or continuous	___
**Controllability**<br>***Could/can you stop thinking about killing yourself or wanting to die if you want to?***<br>(1) Easily able to control thoughts<br>(2) Can control thoughts with little difficulty<br>(3) Can control thoughts with some difficulty<br>(4) Can control thoughts with a lot of difficulty<br>(5) Unable to control thoughts<br>(0) Does not attempt to control thoughts	___
**Deterrents**<br>***Are there things - anyone or anything (e.g., family, religion, pain of death) - that stopped you from wanting to die or acting on thoughts of committing suicide?***<br>(1) Deterrents definitely stopped you from attempting suicide<br>(2) Deterrents probably stopped you<br>(3) Uncertain that deterrents stopped you<br>(4) Deterrents most likely did not stop you<br>(5) Deterrents definitely did not stop you<br>(0) Does not apply	___
**Reasons for Ideation**<br>***What sort of reasons did you have for thinking about wanting to die or killing yourself? Was it to end the pain or stop the way you were feeling (in other words you couldn't go on living with this pain or how you were feeling) or was it to get attention, revenge or a reaction from others? Or both?***<br>(1) Completely to get attention, revenge or a reaction from others<br>(2) Mostly to get attention, revenge or a reaction from others<br>(3) Equally to get attention, revenge or a reaction from others and to end/stop the pain<br>(4) Mostly to end or stop the pain (you couldn't go on living with the pain or how you were feeling)<br>(5) Completely to end or stop the pain (you couldn't go on living with the pain or how you were feeling)<br>(0) Does not apply	___

© 2008 Research Foundation for Mental Hygiene, Inc. C-SSRS—Since Last Visit (Version 1/14/09) Page 1 of 2

SUICIDAL BEHAVIOR *(Check all that apply, so long as these are separate events; must ask about all types)*	**Since Last Visit**
**Actual Attempt:** A potentially self-injurious act committed with at least some wish to die, *as a result of act.* Behavior was in part thought of as method to kill oneself. Intent does not have to be 100%. If there is ***any*** intent/desire to die associated with the act, then it can be considered an actual suicide attempt. ***There does not have to be any injury or harm,*** just the potential for injury or harm. If person pulls trigger while gun is in mouth but gun is broken so no injury results, this is considered an attempt.<br>Inferring Intent: Even if an individual denies intent/wish to die, it may be inferred clinically from the behavior or circumstances. For example, a highly lethal act that is clearly not an accident so no other intent but suicide can be inferred (e.g., gunshot to head, jumping from window of a high floor/story). Also, if someone denies intent to die, but they thought that what they did could be lethal, intent may be inferred.<br>***Have you made a suicide attempt?***<br>***Have you done anything to harm yourself?***<br>***Have you done anything dangerous where you could have died?***<br>***What did you do?***<br>***Did you______ as a way to end your life?***<br>***Did you want to die (even a little) when you_____?***<br>***Were you trying to end your life when you _____?***<br>***Or did you think it was possible you could have died from_____?***<br>***Or did you do it purely for other reasons / without ANY intention of killing yourself (like to relieve stress, feel better, get sympathy, or get something else to happen)?*** (Self-Injurious Behavior without suicidal intent)<br>If yes, describe:	**Yes** **No**<br>☐ ☐<br><br>Total # of Attempts<br>_____
**Has subject engaged in Non-Suicidal Self-Injurious Behavior?**	**Yes** **No**<br>☐ ☐
**Interrupted Attempt:** When the person is interrupted (by an outside circumstance) from starting the potentially self-injurious act *(if not for that, actual attempt would have occurred).*<br>Overdose: Person has pills in hand but is stopped from ingesting. Once they ingest any pills, this becomes an attempt rather than an interrupted attempt. Shooting: Person has gun pointed toward self, gun is taken away by someone else, or is somehow prevented from pulling trigger. Once they pull the trigger, even if the gun fails to fire, it is an attempt. Jumping: Person is poised to jump, is grabbed and taken down from ledge. Hanging: Person has noose around neck but has not yet started to hang - is stopped from doing so.<br>***Has there been a time when you started to do something to end your life but someone or something stopped you before you actually did anything?***<br>If yes, describe:	**Yes** **No**<br>☐ ☐<br><br>Total # of interrupted<br>_____
**Aborted Attempt:** When person begins to take steps toward making a suicide attempt, but stops themselves before they actually have engaged in any self-destructive behavior. Examples are similar to interrupted attempts, except that the individual stops him/herself, instead of being stopped by something else.<br>***Has there been a time when you started to do something to try to end your life but you stopped yourself before you actually did anything?***<br>If yes, describe:	**Yes** **No**<br>☐ ☐<br><br>Total # of aborted<br>_____
**Preparatory Acts or Behavior:** Acts or preparation towards imminently making a suicide attempt. This can include anything beyond a verbalization or thought, such as assembling a specific method (e.g., buying pills, purchasing a gun) or preparing for one's death by suicide (e.g., giving things away, writing a suicide note).<br>***Have you taken any steps towards making a suicide attempt or preparing to kill yourself (such as collecting pills, getting a gun, giving valuables away or writing a suicide note)?***<br>If yes, describe:	**Yes** **No**<br>☐ ☐
**Suicidal Behavior:** Suicidal behavior was present during the assessment period?	**Yes** **No**<br>☐ ☐
**Suicide:**	**Yes** **No**<br>☐ ☐
Answer for Actual Attempts Only	Most Lethal Attempt Date:
**Actual Lethality/Medical Damage:**<br>0. No physical damage or very minor physical damage (e.g., surface scratches).<br>1. Minor physical damage (e.g., lethargic speech; first-degree burns; mild bleeding; sprains).<br>2. Moderate physical damage; medical attention needed (e.g., conscious but sleepy, somewhat responsive; second-degree burns; bleeding of major vessel).<br>3. Moderately severe physical damage; *medical* hospitalization and likely intensive care required (e.g., comatose with reflexes intact; third-degree burns less than 20% of body; extensive blood loss but can recover; major fractures).<br>4. Severe physical damage; *medical* hospitalization with intensive care required (e.g., comatose without reflexes; third-degree burns over 20% of body; extensive blood loss with unstable vital signs; major damage to a vital area).<br>5. Death	*Enter Code*<br><br>_____
**Potential Lethality: Only Answer if Actual Lethality=0** Likely lethality of actual attempt if no medical damage (the following examples, while having no actual medical damage, had potential for very serious lethality: put gun in mouth and pulled the trigger but gun fails to fire so no medical damage; laying on train tracks with oncoming train but pulled away before run over).<br>0 = Behavior not likely to result in injury<br>1 = Behavior likely to result in injury but not likely to cause death<br>2 = Behavior likely to result in death despite available medical care	*Enter Code*<br><br>_____

© 2008 Research Foundation for Mental Hygiene, Inc. C-SSRS—Since Last Visit (Version 1/14/09) Page 2 of 2

APPENDIX O. PARKINSON'S DISEASE DIARY

PARKINSON'S DISEASE DIARY

NAME ______________________________ DATE ________________

Instructions: For each half-hour time period place one check mark to indicate your predominant states during most of that period.
ON = Time when medication is providing benefit with regard to mobility, slowness, and stiffness.
OFF = Time when medication has worn off and is no longer providing benefit with regard to mobility, slowness, and stiffness.
Dyskinesia = involuntary twisting, turning movements. These movements are an effect of medication and occur during ON time.
Non-troublesome dyskinesia does not interfere with function or cause meaningful discomfort. Troublesome dyskinesia interferes with function or causes meaningful discomfort.
Tremor is shaking back and forth and is not considered dyskinesia.

time	asleep	OFF	ON without dyskinesia	ON with non-troublesome dyskinesia	ON with troublesome dyskinesia
6:00 AM					
:30					
7:00 AM					
:30					
8:00 AM					
:30					
9:00 AM					
:30					
10:00 AM					
:30					
11:00 AM					
:30					
12:00 PM					
:30					
1:00 PM					
:30					
2:00 PM					
:30					
3:00 PM					
:30					
4:00 PM					
:30					
5:00 PM					
:30					

time	asleep	OFF	ON without dyskinesia	ON with non-troublesome dyskinesia	ON with troublesome dyskinesia
6:00 PM					
:30					
7:00 PM					
:30					
8:00 PM					
:30					
9:00 PM					
:30					
10:00 PM					
:30					
11:00 PM					
:30					
12:00 AM					
:30					
1:00 AM					
:30					
2:00 AM					
:30					
3:00 AM					
:30					
4:00 AM					
:30					
5:00 AM					
:30					

© RA Hauser 1995

APPENDIX P. EXCIPIENTS IN IPX203, IPX203 PLACEBO, IR CD-LD, AND IR PLACEBO

IPX203 (Various Strengths)	IPX203 Placebo	IR CD-LD (25-100 mg)	IR Placebo
Microcrystalline Cellulose, NF	Microcrystalline Cellulose, NF	Crospovidone	Microcrystalline cellulose, NF
Croscarmellose Sodium, NF	Talc, USP	Hydroxypropyl Cellulose	Magnesium Stearate, NF
Magnesium Stearate, NF	Magnesium Stearate, NF	Magnesium Stearate	Quinoline yellow E104
Mannitol, USP	Sugar Spheres, NF	Microcrystalline cellulose	
Sodium Lauryl Sulfate, NF	Methacrylic acid copolymer Type A, NF	Starch (corn)	
Povidone, USP	Triethyl citrate, NF	D&C Yellow No. 10	
Cellulose Acetate	Hard gelatin capsules		
Copovidone, NF			
Amino Methacrylate Copolymer, NF			
Methacrylic acid copolymer Type A, NF			
Triethyl Citrate, NF			
Talc, USP			
Hard gelatin capsules			

APPENDIX Q. CLINICAL LABORATORY STUDIES

HEMATOLOGY

hemoglobin	% lymphocytes	absolute lymphocytes
hematocrit	% monocytes	absolute monocytes
red blood cell count	% basophils	absolute basophils
white blood cell count	% eosinophils	absolute eosinophils
% neutrophils	absolute neutrophils	platelet count

CHEMISTRY

sodium	calcium	indirect bilirubin
potassium	phosphorous	alkaline phosphatase
chloride	albumin	alanine aminotransferase (ALT, SGPT)
carbon dioxide	total protein	aspartate aminotransferase (AST, SGOT)
blood urea nitrogen (BUN)	uric acid	creatine phosphokinase
creatinine	total bilirubin	lactate dehydrogenase
glucose	direct bilirubin	

URINALYSIS

pH	ketones	leukocyte esterase
specific gravity	microscopic exam (RBC and WBC, only when indicated)	protein
blood		
glucose		

URINE DRUG TEST

amphetamines	benzodiazepines
barbiturates	
cannabinoids	
cocaine metabolites	
opiates	
phencyclidines	

ALCOHOL BREATH TEST

PREGNANCY TEST
Urine pregnancy test (to be completed on site) for female subjects of childbearing potential.

IPX203 (CARBIDOPA-LEVODOPA) EXTENDED-RELEASE CAPSULES

IPX203-B16-02

A RANDOMIZED CONTROLLED STUDY TO COMPARE THE SAFETY AND EFFICACY OF IPX203 WITH IMMEDIATE-RELEASE CARBIDOPA-LEVODOPA IN PARKINSON'S DISEASE PATIENTS WITH MOTOR FLUCTUATIONS

SPONSOR
Impax Laboratories, Inc.,
acting through its Impax Specialty Pharma division (Impax)
30831 Huntwood Ave.
Hayward, CA 94544

Original Protocol, May 18, 2017
Amendment 1, August 30, 2017

CONFIDENTIALITY STATEMENT

All information provided is the property of Impax Laboratories, Inc. and may not be divulged, published or otherwise disclosed without written consent of Impax. All information provided to the Investigator by the Sponsor, including clinical observations at the investigative site, shall be held strictly confidential and confined to the clinical personnel involved in conducting the study, under the supervision of the Investigator. This includes but is not limited to preclinical data, protocols, case report forms, and verbal or written communications. This information may be related in confidence to the Institutional Review Board or other committees functioning in a similar capacity. All reports, subject samples, data published or submitted to third parties will be identified by a coded number and initials only in order to maintain subject confidentiality.

SIGNATURE PAGE

Reviewed and approved by:

for Robert Rubens, MD, MBA
Senior Director, Clinical Development

08/31/2017
Date

Barbara Pruitt, RN, BS
Associate Director, Clinical Operations

08/31/2017
Date

for: Yoshiko Stowell, PhD
Manager, Regulatory Affairs

31AUG2017
Date

Phillip Dinh, PhD
Director, Statistics

31AUG17
Date

Aravind Mittur, PhD
Director, Clinical Pharmacology

08/31/2017
Date

INVESTIGATOR'S AGREEMENT

Protocol No.: IPX203-B16-02

Protocol Title: A Randomized Controlled Study to Compare the Safety and Efficacy of IPX203 with Immediate-Release Carbidopa-Levodopa in Parkinson's Disease Patients with Motor Fluctuations

I have read this protocol and agree to conduct the study as outlined herein, complying with the obligations and requirements of clinical investigators and all other requirements of International Conference on Harmonization (ICH), Good Clinical Practice (GCP), and the appropriate regulatory authority.

I will provide copies of the protocol and all pertinent information to all individuals responsible to me who assist in the conduct of this clinical study. I will discuss this material with them to ensure that they are fully informed regarding the study medication, the conduct of the study, and the obligations of confidentiality.

____ ____________________

Principal Investigator's signature Date

____

Principal Investigator's printed name

STUDY CONTACT INFORMATION

Changes in Impax study personnel listed on this page do not require a protocol amendment.

Role	Name and Contact Information
Sponsor	Impax Laboratories, Inc. acting through its Impax Specialty Pharma division (Impax)<br>30831 Huntwood Avenue<br>Hayward, CA 94544-7037<br>(510) 240-6000<br>FAX: (510) 240-6113 and (510) 240-6114
Medical Monitor	Robert Rubens, MD, MBA<br>Senior Director, Clinical Research & Development<br>Mobile: (510) 303-8207<br>Office: (510) 240-6012<br>E-mail: rrubens@impaxlabs.com
Statistician	Phillip Dinh, PhD<br>Director, Statistics<br>(510) 240-6402<br>E-mail: Phillip.Dinh@impaxlabs.com
Clinical Pharmacology	Aravind Mittur, PhD<br>Director, Clinical Pharmacology<br>(510) 240-6437<br>E-mail: amittur@impaxlabs.com
Clinical Research Associates	Barbara Pruitt, RN, BS<br>Associate Director, Clinical Operations<br>Office: (510) 240-6003<br>Mobile: (510) 329-8489<br>E-mail: bpruitt@impaxlabs.com<br><br>Weiru Hong, MS<br>Senior CRA, Clinical Operations<br>Office: (510) 240-6078<br>Mobile: (510) 731-8309<br>E-mail: whong@impaxlabs.com
Serious Adverse Event Reporting	FAX: (866) 954-2064

1. SYNOPSIS

**Name of Sponsor/Company:** Impax Laboratories, Inc. acting through its Impax Specialty Pharma division (Impax)
**Name of Investigational Product:** IPX203 (carbidopa-levodopa) Extended-Release Capsules
**Name of Active Ingredients:** carbidopa (CD), levodopa (LD)
**Protocol Title:** A Randomized Controlled Study to Compare the Safety and Efficacy of IPX203 with Immediate-Release Carbidopa-Levodopa in Parkinson's Disease Patients with Motor Fluctuations
**Protocol No.:** IPX203-B16-02
**Study center(s):** Multicenter
**Phase of Development:** Phase 3
**Objectives:** To evaluate the safety and efficacy of IPX203 in comparison to IR CD-LD in the treatment of CD-LD-experienced subjects with Parkinson's disease (PD) who have motor fluctuations.
**Methodology:** This is a multicenter, randomized, double-blind, double-dummy, active-controlled, parallel-group study. The study will consist of a 3-week, open-label IR CD-LD dose adjustment period; a 4-week, open-label period for conversion to IPX203; followed by a 13-week double-blind treatment period with subjects randomized in a 1:1 ratio, stratified by center, to receive either IPX203 (with matching IR CD-LD placebo) or IR CD-LD (with matching IPX203 placebo).<br>Subjects will continue to take permitted non-CD-LD-based PD medications throughout the study if documented in their prestudy regimen and if dosing regimens have been stable for at least 4 weeks prior to Visit 1. A "stable dosing regimen" means no change in dose or in dosing frequency.<br>• Within 4 weeks following the Screening visit, eligible subjects will complete their PD Diaries on each of the 3 consecutive days immediately prior to Visit 1.<br>• Following Visit 1, qualified subjects will enter a 3-week, open-label IR CD-LD treatment period allowing for dose adjustment. Any adjustments to the IR CD-LD dosing regimen will be done in consultation with the Investigator or qualified site personnel and will be recorded. The IR CD-LD dosing regimen should be stable for at least 5 days prior to returning for Visit 2. Rescue with additional or modified doses of concomitant PD medications or with use of CD-LD products other than the dispensed study medications are not permitted and will trigger discontinuation from the study. Subjects will complete their 3-day PD Diaries on each of the 3 consecutive days immediately prior to Visit 2.<br>• Following completion of the IR CD-LD dose adjustment period, subjects will begin a 4-week open-label period for conversion to IPX203. The initial dosing regimen of IPX203 will be based on the most frequent dose of the subject's dosing regimen of IR CD-LD at the end of the dose adjustment period (Visit 2). A 25-100 mg dose of IR CD-LD converts to a 70-280 mg CD-LD dose of IPX203 (2 capsules of 35-140 mg CD-LD IPX203), and a 12.5-50 mg dose of IR CD-LD converts to-a 35-140 mg CD-LD dose of IPX203, but with a longer duration of effect. It is recommended that IPX203 should be dosed approximately every 8 hours with the goal of optimizing the therapeutic effect (minimize "Off" time without causing troublesome dyskinesia or other dopaminergic side effects). Any adjustments to the IPX203 dosing regimen will be done in consultation with the Investigator or qualified site personnel and will be recorded. Rescue with additional or modified doses of concomitant PD medications or with use of CD-LD products other than the dispensed study medications are not permitted and will

trigger discontinuation from the study. Subjects will return to the clinic in 2 weeks for Visit 3 followed by Visit 4, 2 weeks later. The IPX203 dosing regimen should be stable for at least 5 days prior to returning for Visit 4. Subjects will complete their 3-day PD Diaries on each of the 3 consecutive days immediately prior to Visit 4.

- Subjects who successfully complete the IPX203 dose conversion period will be randomized in 1:1 ratio, stratified by center, at Visit 4 into one of two parallel treatment arms of IPX203 (with matching IR CD-LD placebo) or IR CD-LD (with matching IPX203 placebo). The subjects will undergo 13 weeks of double-blind maintenance therapy with the stable dosing regimen established at the end of Week 3 (Visit 2) for IR CD-LD and at the end of Week 7 (Visit 4) for IPX203. Rescue with additional or modified doses of concomitant PD medications or with use of CD-LD products other than the dispensed study medications are not permitted and will trigger discontinuation from the study. Subjects will return to the clinic for 3 visits (Visits 5, 6, and 7) and will complete their 3-day PD Diaries on each of the 3 consecutive days immediately prior to each of these visits.

Number of patients (planned): Assuming approximately an 18% prerandomization drop-out, approximately 510 subjects will be enrolled to randomize 420 subjects.

Diagnosis and main criteria for inclusion:

Inclusion Criteria

- Male or female subjects diagnosed at age ≥ 40 years with PD, consistent with the United Kingdom Parkinson's Disease Society Brain Bank Diagnostic Criteria and who are being treated with stable regimens of CD-LD but experiencing motor fluctuations.
- Hoehn and Yahr Stages 1, 2, 3, or 4 in the "On" state (part of Movement Disorders Society version of the Unified Parkinson's Disease Rating Scale [MDS-UPDRS] Part III)
- Montreal Cognitive Assessment (MoCA) score ≥ 24 at Screening Visit in "On" state.
- By history, for the 4 weeks prior to Screening, the subject experiences daily "wearing-off" episodes with periods of bradykinesia in combination with at least one of rest tremor or rigidity, experiences an "Off" state upon awakening on most mornings, and reports an average of at least 2.5 cumulative hours per day of "Off" time during the waking hours.
- Able to differentiate "On" state from "Off" state as determined by at least 75% concordance with a trained rater in "On/Off" ratings for 8 ratings over a 4-hour training period. The concordance must include at least 1 "On" and 1 "Off" rating and must be achieved within two 4-hour training sessions.
- At Visit 1, review of the 3-day PD Diaries confirms the following: that the subject is able to properly complete the Diaries with valid entries; and that the subject has an average of at least 2.5 hours per day of "Off" time during waking hours over the 3 days with at least 1.5 hours of cumulative "Off" time on each day.
- Responsive to CD-LD therapy and currently being treated on a stable regimen with CD-LD for at least 4 weeks prior to Visit 1 and:
  - Requires at least 100 mg of LD from IR CD-LD for the first morning dose
  - Requires a total daily dose of at least 400 mg of LD and takes a maximum total daily dose of 2400 mg LD, from IR CD-LD alone or IR CD-LD in combination with a single daily bedtime dose of CR CD-LD
  - Has a dosing frequency of 4 to 9 times daily of CD-LD
  - By history, typically experiences an "On" response with the first dose of IR CD-LD of the day, but the efficacy of this dose typically lasts less than 4 hours.

- At Screening, the subject has predictable "Off" periods defined by a score of 1 or 2 on Item #4.5 (Complexity of Motor Fluctuations) of the MDS-UPDRS Part IV B (Motor Fluctuations).
- At Screening, the MDS-UPDRS Part III total score in the "Off" state is at least 20 units.

Exclusion Criteria

- Used any doses of controlled-release (CR) CD-LD apart from a single daily bedtime dose within 4 weeks prior to Visit 1.
- Used any dose of Rytary for the past 4 weeks prior to Visit 1 or were considered IPX066 or Rytary failures for reasons of efficacy or safety.
- Had prior neurosurgical treatment for PD or if such procedure is planned or anticipated during the study period.
- Allergic to any excipient in the study drugs.
- History of glaucoma with intraocular pressures that are elevated despite appropriate medical management.
- History of seizure or epilepsy and experienced at least 1 seizure during the past 12 months or has not been compliant with medically recommended therapy or visits.
- History of myocardial infarction with residual atrial, nodal, or ventricular arrhythmias that are not controlled with medical and/or surgical interventions. A recent (≤ 12 months) history of myocardial infarction with secondary arrhythmias is exclusionary regardless of the therapeutic control.
- Received within 4 weeks of Screening or planning to take during participation in the clinical study:
    - Any doses of a CR CD-LD apart from a single daily bedtime dose, any doses of Rytary, additional CD (eg, Lodosyn) or benserazide (eg, Serazide), or catechol-O-methyl transferase inhibitors (entacapone or tolcapone) or medications containing these inhibitors (Stalevo),
    - Nonselective monoamine oxidase inhibitors (MAOI), apomorphine, or antidopaminergic agents, including antiemetics.
- Subjects who have previously participated in an IPX203 study.

Investigational product, dosage and mode of administration: IPX203 (carbidopa-levodopa) Extended-Release capsules, containing 35-140 mg of CD-LD and matching placebo, for oral administration.

Reference therapy, dosage and mode of administration: Immediate-Release carbidopa-levodopa (IR CD-LD) tablets containing 25-100 mg of CD-LD, and matching placebo, for oral administration.

Duration of treatment: Approximately 24 weeks, including up to 4 weeks following Screening, 3 weeks of IR CD-LD dose adjustment, 4 weeks of IPX203 dose conversion, and 13 weeks of double-blind therapy following randomization.

Criteria for evaluation:

Baseline is defined as assessments done at Visit 4 (randomization visit). Study Entry is defined as assessments done at Visit 1 (study entry visit).

Efficacy:

- Primary endpoint: Change from baseline in "Good on" time in hours per day, averaged over the PD Diary days, at the end of double-blind treatment period (Visit 7 or early termination). "Good on" time is derived from the 3-day PD Diaries and is defined as the sum of "On" time without dyskinesia and "On" time with nontroublesome dyskinesia.

- Key secondary endpoints:
  - Change from baseline in "Off" time in hours per day, averaged over the PD Diary days at the end of double-blind treatment period (Visit 7 or early termination)
  - Proportion of subjects with either "much improved" or "very much improved" in Patient Global Impression of Change (PGI-C) scores at the end of double-blind treatment period (Visit 7 or early termination)
  - Change from baseline in the MDS-UPDRS Part III at the end of double-blind treatment period (Visit 7 or early termination)
  - Change from baseline in the sum of MDS-UPDRS Parts II and III at the end of double-blind treatment period (Visit 7 or early termination)
- Additional endpoints:

  The following endpoints will be evaluated (at the post-randomization visits) as change from baseline (Visit 4) as well as change from the study entry (Visit 1), when applicable, by visits:
  - Percent "Off" time during waking hours derived from the 3-day PD Diaries
  - Average duration of each continuous "Good on" and each continuous "On"
  - Hours of (1) "Off" time (from Visit 1), (2) "Good on" time (from Visit 1), (3) "On" time with dyskinesia, (4) "On" time with troublesome dyskinesia, and (5) "On" time with nontroublesome dyskinesia, and (6) asleep time derived from the 3-day PD Diaries
  - Proportion of subjects with an improvement in "Good on" time of at least 1, 1.5, 2, 2.5, and 3 hours
  - Proportion of subjects with a reduction in "Off" time of at least 0.5, 1, 1.5, 2, 2.5, and 3 hours
  - Proportions of subjects who are "On" upon awakening and "Good on" upon awakening
  - Average time to "On" upon awakening
  - Change from baseline in the average number of motor fluctuations per day averaged over the PD Diary days. A motor fluctuation is defined as a change from "Off" to "On" state or from "On" to "Off" state.
  - MDS-UPDRS total score (sum of Parts I, II, III, and IV) and Parts I, II, and IV separately
  - MDS-UPDRS Part III and Parts II + III combined (from Visit 1)
  - MDS-UPDRS Part II Question 2.9
  - PDQ-39 total score and individual domain scores
  - NMSS total score and individual domains
  - PDSS-2 total score and individual domains
  - PDSS-2 items 9, 10, 11, 12, and 13 combined
  - PAS total score and individual domains
  - PGI-S
  - Proportion of subjects with either "severely ill" or "extremely severely ill" on the PGI-S
  - CGI-S

- – Proportion of subjects with either “severely ill” or “among the most extremely ill of subjects” on the CGI-S
- – PGI-C scores
- – CGI-C scores
- – Proportion of subjects with either “much improved” or “very much improved” on the CGI-C

- **Safety:** electrocardiograms (ECGs), clinical laboratory tests, physical examinations, Columbia-Suicide Severity Rating Scale (C-SSRS), and supine and standing orthostatic vital signs; adverse events and concomitant medications evaluated throughout the course of the study.

Statistical methods: For the primary endpoint, assuming a difference of 1 hour between IPX203 and IR CD-LD in “Good on” time and a standard deviation of the treatment difference to be 3.0 hours, a sample size of 210 per arm will be needed to ensure at least 90% power at a 0.05 significance level. Assuming approximately an 18% prerandomization drop-out, approximately 510 subjects would need to be enrolled to randomize 420 subjects.

The primary efficacy endpoint of change from baseline in “Good on” time will be analyzed using a mixed model for repeated measures (MMRM) model. The model will include baseline (Visit 4) “Good on” time as a covariate, treatment, visit, and pooled center as fixed effects, and a treatment-by-visit interaction. The model will employ an unstructured within subject covariance matrix and a restricted maximum likelihood (ReML) estimation method. The degree-of-freedom of the denominator will be estimated using the Kenward-Roger method.

The key secondary endpoints (change from baseline in “Off” time, change from baseline in MDS-UPDRS Part III, and change from baseline in the sum of the MDS-UPDRS Parts II and III will be analyzed using MMRM models similar to the primary analysis model. The proportion of subjects with either “much improved” or “very much improved” on the PGI-C will be analyzed using Cochran-Mantel-Haenszel test to compare the two treatment groups with pooled center as a stratification factor.

In order to control the type I error rate, the primary and key secondary endpoints will be tested in a single hierarchical order: (1) change from baseline in “Good on” time, (2) change from baseline in “Off” time, (3) proportion of subjects with either “much improved” or “very much improved” in PGI-C, (4) change from baseline in the MDS-UPDRS Part III, (5) change from baseline in the sum of MDS-UPDRS Parts II and III.

Quantitative safety data will be summarized using descriptive statistics and frequency distributions. Qualitative safety data will be summarized by frequencies and percentages. All summaries will be presented by treatment arms.

2. TABLE OF CONTENTS, LIST OF TABLES, AND LIST OF FIGURES

TABLE OF CONTENTS

1. SYNOPSIS 5
2. TABLE OF CONTENTS, LIST OF TABLES, AND LIST OF FIGURES 10
3. LIST OF ABBREVIATIONS AND DEFINITIONS OF TERMS 16
4. INTRODUCTION 19
5. TRIAL OBJECTIVES 23
6. INVESTIGATIONAL PLAN 24
6.1. Overall Study Design 24
6.2. Number of Subjects 25
6.3. Treatment Assignment 25
6.4. Dosing and Dose Determination Criteria 26
6.4.1. IR CD-LD Dose Adjustment Period 26
6.4.2. IPX203 Dose Conversion Period 26
6.4.3. Double-Blind Maintenance Period 27
6.5. Criteria for Study Termination 27
7. SELECTION AND WITHDRAWAL OF SUBJECTS 28
7.1. Subject Inclusion Criteria 28
7.2. Subject Exclusion Criteria 29
7.3. Subject Withdrawal Criteria 30
8. STUDY PROCEDURES 32
8.1. Screening Visit 36
8.2. Visit 1 – Start of IR CD-LD Dose Adjustment 37
8.2.1. Prior to Visit 1 37
8.2.2. At Visit 1 37
8.2.3. Post Visit 1 38
8.3. Visits 2 (Week 3) and Visit 3 (Week 5) – IPX203 Dose Conversion 38
8.3.1. Prior to Visit 2 38
8.3.2. Prior to Visit 3 38
8.3.3. At Visits 2 and 3 38

8.3.4.	Post Visits 2 and 3	39
8.4.	Visit 4 (Week 7) – Randomization	39
8.4.1.	Prior to Visit 4	39
8.4.2.	At Visit 4	39
8.5.	Visit 5 (Week 10) and Visit 6 (Week 15)	40
8.5.1.	Prior to Visit 5 and 6	40
8.5.2.	At Visit 5 and 6	40
8.6.	Visit 7 (Week 20) – End of Study/Study Exit	41
8.6.1.	Prior to Visit 7	41
8.6.2.	At Visit 7	41
8.7.	Early Termination	42
8.7.1.	Subjects Who Terminate Prior to Randomization	42
8.7.2.	Subjects Who Terminate Early after Randomization	42
8.8.	Blood Volume	43
9.	TREATMENT OF SUBJECTS	44
9.1.	Description of Study Drug	44
9.2.	Concomitant Medications	44
9.2.1.	Permitted PD Medications	44
9.2.2.	Prohibited Medications and Procedures	44
9.2.3.	Rescue Medications	45
9.3.	Treatment Compliance	45
9.4.	Randomization and Blinding	45
10.	STUDY DRUG MATERIALS AND MANAGEMENT	46
10.1.	Study Drug	46
10.2.	Study Drug Packaging and Labeling	46
10.3.	Study Drug Storage	46
10.4.	Study Drug Administration	47
10.5.	Study Drug Dispensing and Accountability	47
10.6.	Study Drug Handling and Disposal	47
11.	ASSESSMENT OF EFFICACY	48
11.1.	Parkinson’s Disease Diary	48
11.2.	Patient and Investigator Global Assessments	48

11.3. Movement Disorders Society Version of Unified Parkinson's Disease Rating Scale....48
11.4. Additional Assessments....49
12. ASSESSMENT OF SAFETY....50
12.1. Safety Parameters....50
12.2. Adverse Events....50
12.2.1. Definition of Adverse Event....50
12.2.2. Recording Adverse Events....50
12.2.3. Follow-up....51
12.2.4. Relationship to Study Drug....51
12.2.5. Assessment of Severity....51
12.3. Serious Adverse Events....51
12.3.1. Definition of Serious Adverse Event....51
12.3.2. Reporting Serious Adverse Events....52
12.4. Pregnancy....52
12.5. Other Safety Parameters and Related Information....53
13. STATISTICS....54
13.1. Study Design and Sample Size Estimation....54
13.2. Demographics/Baseline Comparability....54
13.3. Efficacy Endpoints....54
13.4. Analysis of Efficacy Data....56
13.4.1. Primary Efficacy Endpoint....56
13.4.2. Key Secondary Efficacy Endpoints....56
13.4.3. Additional Efficacy Endpoints....57
13.5. Center Pooling Algorithm....57
13.6. Sensitivity Analyses of the Primary Endpoint and Key Secondary Endpoints....58
13.6.1. Assessing Assumptions of the Mixed Model for Repeated Measures (MMRM)....58
13.6.2. Complete Case Analysis....58
13.6.3. Single LOCF/BLOCF Imputation....58
13.6.4. Pattern-Mixture Model....59
13.7. Subgroup Analyses....60
13.8. Multiplicity Adjustments....60

13.9. Analysis Populations 61
13.9.1. Safety Analysis Set 61
13.9.2. Intent-to-Treat Analysis Set 61
13.9.3. Modified Intent-to-Treat Analysis Set 61
13.9.4. Completers Analysis Set 61
13.10. Handling of Missing Data 61
13.10.1. Missing Data for PD Diaries 61
13.10.2. Missing Data for Global Assessments (PGI-C, CGI-C, PGI-S and CGI-S) 62
13.10.3. Missing Data for MDS-UPDRS 62
13.11. Analysis of Safety 63
14. ADMINISTRATIVE PROCEDURES 64
14.1. Guidelines for Good Clinical Practice 64
14.2. Institutional Review Board Approval 64
14.3. Informed Consent 64
14.4. Study Monitoring 64
14.5. Protocol Amendments 64
14.6. Termination of Study 65
14.7. Case Report Forms 65
14.8. Investigator’s Final Conduct Report 65
14.9. Records Retention 65
15. PUBLICATION POLICY 66
16. LIST OF REFERENCES 67
17. APPENDICES 69
APPENDIX A. PRESCRIBING INFORMATION FOR IR CD-LD 69
APPENDIX B. UNITED KINGDOM PARKINSON’S DISEASE SOCIETY BRAIN BANK DIAGNOSTIC CRITERIA FOR PARKINSON’S DISEASE 80
APPENDIX C. MONTREAL COGNITIVE ASSESSMENT (MOCA) 81
APPENDIX D. MOVEMENT DISORDERS SOCIETY VERSION OF UNIFIED PARKINSON’S DISEASE RATING SCALE (MDS-UPDRS) 87
APPENDIX E. PATIENT GLOBAL IMPRESSION OF CHANGE (PGI-C) 120
APPENDIX F. CLINICAL GLOBAL IMPRESSION OF CHANGE (CGI-C) 121
APPENDIX G. PATIENT GLOBAL IMPRESSION OF SEVERITY (PGI-S) 122
APPENDIX H. CLINICAL GLOBAL IMPRESSION OF SEVERITY (CGI-S) 123

APPENDIX I. 39-ITEM PARKINSON'S DISEASE QUESTIONNAIRE (PDQ-39) 124

APPENDIX J. GASTROPARESIS CARDINAL SYMPTOM INDEX (GCSI)......................128

APPENDIX K. NON-MOTOR SYMPTOM ASSESSMENT SCALE FOR PARKINSON'S DISEASE (NMSS)..130

APPENDIX L. PARKINSON'S DISEASE SLEEP SCALE-2 (PDSS-2) 133

APPENDIX M. PARKINSON ANXIETY SCALE (PAS) 134

APPENDIX N. COLUMBIA-SUICIDE SEVERITY RATING SCALE (C-SSRS)................138

APPENDIX O. PARKINSON'S DISEASE DIARY...145

APPENDIX P. EXCIPIENTS IN IPX203, IPX203 PLACEBO, IR CD-LD, AND IR PLACEBO 146

APPENDIX Q. CLINICAL LABORATORY STUDIES...147

LIST OF TABLES

Table 1: Abbreviations and Specialist Terms 16
Table 2: LD Dosage in Study IPX203-B14-02 20
Table 3: Recommended Starting IPX203 LD Dosing Regimen Based on the Dosing Regimen of IR CD-LD at the End of the Dose Adjustment Period 27
Table 4: Events Schedule for Impax Study IPX203-B16-02 33
Table 5: Study Drugs for Study IPX203-B16-02 44

LIST OF FIGURES

Figure 1: Study Flow Chart 25

3. LIST OF ABBREVIATIONS AND DEFINITIONS OF TERMS

The following abbreviations and specialist terms are used in this study protocol.

Table 1: Abbreviations and Specialist Terms

Abbreviation or Specialist Term	Explanation
AADC	aromatic amino acid decarboxylase
ADL	activities of daily living
AE	adverse event
ANCOVA	analysis of covariance
ANOVA	analysis of variance
BLOCF	baseline observation carried forward
BMI	body mass index
CD	carbidopa
CGI-C	Clinical Global Impression of Change
CGI-S	Clinical Global Impression of Severity
CR	controlled release
CRF	case report form
C-SSRS	Columbia-Suicide Severity Rating Scale
ECG	electrocardiogram
ER	extended release
FDA	Food and Drug Administration
GCP	Good Clinical Practice
GCSI	Gastroparesis Cardinal Symptom Index
HIPAA	Health Insurance Portability and Accountability Act
ICF	informed consent form
ICH	International Conference on Harmonization
IEC	independent ethics committee

Abbreviation or Specialist Term	Explanation
IR	immediate release
IRB	institutional review board
IWRS	interactive web response system
LD	levodopa
LOCF	last observation carried forward
MAOI	monoamine oxidase inhibitors
MAR	missing at random
M-EDL	Motor Aspects of Experiences of Daily Living
MedDRA	Medical Dictionary for Regulatory Activities
MDS-UPDRS	Movement Disorders Society version of the Unified Parkinson's Disease Rating Scale
mITT	modified intent-to-treat
MMRM	mixed model for repeated measures
MNAR	missing not at random
MoCA	Montreal Cognitive Assessment
nM-EDL	Non-Motor Aspects of Experiences of Daily Living
NMSS	Non-Motor Symptom Assessment Scale
PAS	Parkinson Anxiety Scale
PD	Parkinson's disease
PDQ-39	39-item Parkinson's Disease Questionnaire
PDSS-2	Parkinson's Disease Sleep Scale-2
PGI-C	Patient Global Impression of Change
PGI-S	Patient Global Impression of Severity
PK	pharmacokinetic (adjective)<br>pharmacokinetics (singular noun)
PI	principal investigator

Abbreviation or Specialist Term	Explanation
PMM	pattern-mixture models
ReML	restricted maximum likelihood
SAE	serious adverse event
SAP	statistical analysis plan
SD	standard deviation
TEAE	treatment-emergent adverse event
US	United States

4. INTRODUCTION

Parkinson's disease (PD) is a progressive neurodegenerative disorder of the extrapyramidal nervous system. Levodopa (LD) used in combination with carbidopa (CD) is considered the gold standard for the symptomatic treatment of PD. LD is a dopamine precursor converted to dopamine by aromatic amino acid decarboxylase (AADC). Carbidopa is an AADC inhibitor that does not cross the blood-brain barrier. When used in combination with LD, CD increases the plasma half-life of LD from 50 minutes to 1.5 hours. Carbidopa inhibits the conversion of LD into dopamine in the periphery, thereby reducing the peripheral side-effects caused by dopamine and increasing the amount of LD available for transport into the brain. The administration of CD with LD reduces the dose of LD required to produce a dopaminergic response by about 75 percent (Sinemet prescribing information).

Due to its proven efficacy, LD is prescribed eventually to most subjects with PD. However, long-term use of LD is associated with certain complications, including "wearing-off" or "end-of-dose effect," where symptom control decreases causing the drug effects to wear off sooner. As the disease progresses further, motor complications, namely dyskinesias and motor "On/Off" fluctuations, develop in about 50% of the patients after 5 years of treatment (Fahn 1999). Such motor complications can be a significant source of disability and their management is a major unmet need in the treatment of PD.

Mechanisms underlying motor complications involving dyskinesias and "On/Off" fluctuations in PD are unclear. The pulsatile nature of standard orally administered LD is thought to contribute to the appearance of motor complications. Chronic intermittent pulsatile stimulation of the dopamine receptors that are under tonic control contributes to the development of dyskinesia in PD animal models as compared to animals treated with continuous infusion (Juncos et al 1989, Engber et al 1989, Blanchet et al 1995). In addition, unreliable absorption of LD potentially due to erratic gastric empting and variable in vivo dissolution of LD products is thought to contribute to the delay or inadequate response after oral dosing with standard CD-LD products (Melamed et al 1986, Kurlan et al 1988, Stocchi et al 1994). These findings suggest that motor complications in patients with PD may be less likely to develop with continuous dopaminergic stimulation.

Intraduodenal infusion of LD has been shown to significantly reduce motor complications and to reduce "Off" time. The findings of infusion studies in PD patients indicate that the maintenance of stable plasma LD concentrations and the avoidance of low trough levels are effective in reducing "Off" hours, increasing "On" hours without disabling dyskinesia, and reducing the severity of dyskinesia versus standard oral LD formulations (Mizuno 2007, Nilsson et al 2001, Nyholm et al 2005, Stocchi et al 2005). These findings provide a strong rationale for the development of an extended-release (ER) oral dosage form that delivers a constant LD plasma concentration in order to optimize relief of PD symptoms, and to minimize "Off" time and dyskinesia.

IPX203 is an investigational product containing CD-LD that is being developed by Impax Laboratories, Inc. through its Impax Specialty Pharma division (Impax). The primary objective of the IPX203 program is to develop an extended-release product that can attain therapeutic LD

plasma concentrations rapidly and maintain constant LD plasma concentrations for a longer duration than currently approved products with minimal peak-to-trough fluctuations. IPX203 is designed to be dosed approximately every 8 hours.

Impax characterized the PK and pharmacodynamics of IPX203 in Study IPX203-B14-02, a single dose trial in subjects with advanced PD versus IR CD-LD and Rytary (carbidopa and levodopa) extended-release capsules. Twenty-six (26) subjects were randomized with 25 subjects completing all 3 treatments. One subject discontinued study early due to subject withdrawal. The doses of IPX203 and Rytary were determined on the basis of each subject's prestudy baseline morning dose of IR CD-LD (Table 2).

Table 2: LD Dosage in Study IPX203-B14-02

Prestudy Baseline Morning IR LD (mg)	IR LD (mg)	Rytary (mg)	IPX203 (mg)
100	100	340	360
150	150	485	540
200	200	630	720
250	250	780	810

Administration of IPX203 yielded an initial increase in LD plasma concentrations that was similar to IR CD-LD but maintained LD concentrations for a longer duration than either IR CD-LD or Rytary. The bioavailability of LD (based on AUC_{∞}) from IPX203 was 78% relative to IR CD-LD and about 14% more than Rytary. Plasma exposure to LD (C_{max} and AUC_{∞}) following IPX203 increased in an approximately dose-proportional manner. Pharmacodynamic effects as measured by change from baseline scores on MDS-UPDRS Part III were consistent with the PK profiles of LD. Following IPX203 treatment, decrements in the Part III total score (reflecting improvements in motor symptoms) lasted for a longer duration than either IR CD-LD or Rytary. IPX203 provided a longer duration of effect compared with IR CD-LD and Rytary, including "Off" time and "Good on" time based on the Assessment of Subject's Motor State and on a range of improvement thresholds of the MDS-UPDRS Part III. The results were consistent with the prolonged LD plasma concentration profile with IPX203 compared to IR CD-LD or Rytary and support a dosing interval of approximately 8 hours.

Of the 26 subjects who received at least one of the 3 treatments, 9 (34.6%) reported at least one treatment-emergent adverse event (AE). None of these subjects reported a serious AE (SAE) nor did any subjects prematurely discontinue the study because of an AE. Adverse events were reported by more subjects during IR (28.0%) and IPX203 (19.2%) than during Rytary (8.0%) treatment. None of the reported AEs were classified as "severe." Adverse events reported by 2 or more subjects include: Dizziness (3 subjects), nausea (2 subjects), and hypertension (2 subjects). The numbers of subjects reporting these AEs during any single treatment period were small (0 to 2 subjects). Two subjects reported dizziness during IR CD-LD treatment and one subject each during IPX203 and Rytary treatments. Hypertension was reported by a total of 2 subjects, both reporting this AE during IPX203 and IR CD-LD treatments and 1 subject during Rytary treatment. Two subjects reported nausea only during the IR CD-LD treatment period. Of the 9 subjects reporting AEs, 6/26 (23.1%) of subjects reported AEs that were assessed as related to treatment, including all of the reports of dizziness, nausea, and dyskinesia (1 subject).

Study IPX203-B16-01 is a randomized, open-label, rater-blinded, multicenter, 2-treatment, 2-period, multiple-dose crossover study that has completed dosing. Twenty-eight (N=28) advanced PD subjects were randomized to 1 of 2 dosing sequences, with each treatment period lasting 15 days and separated by a 1-week wash-out period where subjects return to their usual stable pre-study CD-LD regimen. The objectives of this study are to compare the PK, pharmacodynamics, efficacy, and safety of IPX203 with IR CD-LD after single and multiple dosing. Subjects were permitted to take allowed non-CD-LD based PD medications throughout the study if dosing regimens had been stable for at least 4 weeks. Subjects were instructed to take their last dose of CD-LD no later than 10:00 PM on the evening prior to Day 1 of each treatment period and to withhold dosing for at least 5 hours before arriving at the site on Day 15 of each treatment period. On Day 1 of the IR CD-LD treatment period, subjects were started with a single dose of their usual prestudy first morning IR CD-LD dose. On Day 1 of the IPX203 treatment period, subjects were started with a single dose of IPX203 based on their usual prestudy first morning IR CD-LD dose using a LD conversion of 100 mg IR LD to 360 mg of IPX203 LD. During the IR CD-LD treatment period, the initial dosing regimen of IR CD-LD was the same as the subject’s stable prestudy regimen. During the IPX203 treatment period, the IPX203 regimen for subsequent doses for the day was determined by identifying the most frequent prestudy IR LD dose in milligrams that the subject received in the afternoon and evening and administering IPX203 using a LD conversion of 100 mg IR LD to 270 mg of IPX203 LD. The protocol recommended that IPX203 be dosed approximately every 7 to 8 hours. During Days 1 through 9 of both treatment periods, investigators had the opportunity to adjust each subject’s study medication regimen if necessary to optimize efficacy and safety. Pharmacokinetics and pharmacodynamics (MDS-UPDRS Part III and Assessments of Subject’s Motor State) were periodically evaluated on Day 1 and Day 15 of each treatment period by qualified clinical staff who were blinded to dosing.

Data from this multiple-dose study confirmed the PK and pharmacodynamic results observed in the single dose study with IPX203:

- PK data from 18 subjects indicates IPX203 shows a rapid increase in LD concentrations followed by extended-release characteristics. Following IPX203; initial increases in LD concentrations were comparable to that from IR CD-LD. Bioavailability of LD following IPX203 was ~93% relative to IR CD-LD. LD plasma concentrations were sustained longer after IPX203 treatment than after IR CD-LD and support dosing every 8 hours. No accumulation of LD was evident at steady-state following IPX203 or IR CD-LD. Plasma LD concentrations following IPX203 were characterized by lower peak-to-trough fluctuation. No time-variant or time-dependent changes were noted in PK of CD or LD following IPX203.
- IPX203 demonstrated an onset of effect that was comparable to IR CD-LD in MDS-UPDRS Part III scores. IPX203 prolonged the duration over which MDS-UPDRS Part III scores were improved by prespecified threshold changes from baseline (≥4, ≥7, and ≥13 units).
- IPX203 provides a significant decrease in “Off” time and a significant increase in “Good on” time compared to IR CD-LD treatment on Day 1 and Day 15 when assessed by the Investigator’s Assessment of Subject’s Motor State. Subjects treated

with IPX203 did not experience a significant increase in "On" time with troublesome dyskinesia compared to IR CD-LD.

- Subjects achieved significant improvements in "Off" time, "Good on" time, and frequency of motor state fluctuations based on the 3-day PD Diaries.
- Twenty-eight subjects were enrolled in the multiple dose study and 27 subjects completed both treatments. Safety results were as follows:
  - One subject discontinued during the IPX203 treatment period due to an AE (orthostatic hypertension) that was considered possibly related to treatment.
  - A total of 42.9% (12/28) of treated subjects reported at least one treatment emergent AE, including 39.3% (11/28) during IPX203 treatment and 7.4% (2/27) during IR CD-LD treatment. Eight subjects reported AEs that were related to treatment (8 subjects during IPX203 treatment and 1 during IR CD-LD treatment).
  - Two subjects experienced serious adverse events (SAEs). One subject reported increased hypertension of mild severity during IPX203 treatment that was considered unrelated to treatment and resolved. A second subject reported moderate to severe dehydration, diarrhea, and atrial fibrillation during the washout period that were considered unrelated to treatment and resolved.
  - AEs reported in 2 or more subjects included nausea (2), dizziness (2), and dyskinesia (5), all of mild or moderate severity, and all during the IPX203 treatment.

The current protocol, Study IPX203-B16-02, is a multicenter, randomized, double-blind, double-dummy, active-controlled, parallel-group, Phase 3 study. It is designed to compare the efficacy, safety and tolerability of IPX203 with IR CD-LD following multiple doses over 13 weeks. The following IPX203 dosing guidelines will be utilized in the present study (IPX203-B16-02):

- The initial regimen of IPX203 is based on the most frequent dosing regimen of IR CD-LD at the end of dose adjustment period;
- A 25-100 mg dose of IR CD-LD will be converted to a 70-280 mg CD-LD dose of IPX203;
- IPX203 will be administered approximately every 8 hours;
- Investigators may adjust the IPX203 regimen during the dose conversion period to optimize the therapeutic effect (minimize "Off" time without causing troublesome dyskinesia or other dopaminergic side effects).

The proposed dose conversion scheme for this study has been developed based on a similar dose conversion from IR CD-LD to IPX203 that was studied in the completed Phase 2a study (IPX203-B14-02, n=25) and the Phase 2b study (IPX203-B16-01, n=28), both conducted in subjects with advanced PD using similar entry criteria to the present study. The doses of IPX203 are expected to be comparable to other ER CD-LD products, such as Rytary and Duopa.

5. TRIAL OBJECTIVES

To evaluate the safety and efficacy of IPX203 in comparison to IR CD-LD in the treatment of CD-LD experienced subjects with Parkinson's disease (PD) who have motor fluctuations.

6. INVESTIGATIONAL PLAN

6.1. Overall Study Design

This is a multicenter, randomized, double-blind, double-dummy, active-controlled, parallel-group study. Subjects will continue to take permitted non-CD-LD-based PD medications throughout the study if documented in their prestudy regimen and if dosing regimens have been stable for at least 4 weeks prior to Visit 1. A "stable dosing regimen" means no change in dose or in dosing frequency. Within 4 weeks following the Screening visit, eligible subjects will complete their PD Diaries on each of the 3 consecutive days immediately prior to Visit 1.

Following Visit 1, qualified subjects will enter a 3-week, open-label IR CD-LD treatment period allowing for dose adjustment. During the IR CD-LD dose adjustment period, the initial dosing regimen of IR CD-LD will be the same as the subject's stable prestudy regimen unless they were taking a single daily bedtime dose of CR CD-LD, either alone or within an hour of a dose of IR CD-LD, in which case, the CR CD-LD dose will be discontinued and substituted with a 1:1 milligram-equivalent dose of IR CD-LD. A "bedtime dose" is defined as the last daytime dose of CD-LD taken within 1 hour of onset of the subject's normal nighttime sleep period. The dosing regimen may be adjusted during the dose adjustment period. Any adjustments to the IR CD-LD dosing regimen will be recorded. The IR CD-LD dosing regimen should be stable (no change in dose or in dosing frequency) for at least 5 days prior to returning for Visit 2. Rescue with additional or modified doses of concomitant PD medications or with use of CD-LD products other than the dispensed study medications are not permitted and will trigger discontinuation from the study. Subjects will complete their 3-day PD Diaries on each of the 3 consecutive days immediately prior to Visit 1.

Following completion of the IR CD-LD dose adjustment period, subjects will begin a 4-week open-label period for conversion to IPX203. The initial dosing regimen of IPX203 during the conversion period will be based on the subject's dosing regimen of IR CD-LD at the end of the dose adjustment period (Visit 2) according to Table 3. A 25-100 mg dose of IR CD-LD converts to a 70-280 mg CD-LD dose of IPX203 but with a longer duration of effect. It is recommended that IPX203 should be dosed approximately every 8 hours with the goal of optimizing the therapeutic effect (minimize "Off" time without causing troublesome dyskinesia or other dopaminergic side effects). Any adjustments to the IPX203 dosing regimen will be recorded. Rescue with additional or modified doses of concomitant PD medications or with use of CD-LD products other than the dispensed study medications are not permitted and will trigger discontinuation from the study. Subjects will return to the clinic in two weeks for Visit 3 followed by Visit 4 two weeks later. The IPX203 dosing regimen should be stable for at least 5 days prior to returning for Visit 4. Subjects will also be instructed to complete their 3-day PD Diaries on each of the 3 consecutive days immediately prior to Visit 4.

The study staff will call the subjects approximately every 1 to 3 days during the IR CD-LD dose adjustment and IPX203 dose conversion periods. The calls are to ensure timely and appropriate dosing adjustments and to ensure that the subject is able to follow and adhere to the dosing instructions. The contacts may be less frequent after initial dose adjustments have been made.

Any changes in the dosing regimen will be in consultation with the Investigator or qualified site personnel and will be documented.

Subjects who successfully complete the IPX203 dose conversion periods will be randomized, stratified by center, in a 1:1 ratio at Visit 4 into one of two parallel treatment arms of IPX203 (with matching IR CD-LD placebo) or IR CD-LD (with matching IPX203 placebo). The subjects will undergo 13 weeks of double-blind, double-dummy maintenance therapy with the stable dosing regimen established at the end of Week 3 (Visit 2) for IR CD-LD and at the end of Week 7 (Visit 4) for IPX203. Subjects will return to the clinic for 3 visits (Visits 5, 6, and 7) and will be instructed to complete their 3-day PD Diaries on 3 consecutive days immediately prior to each of the 3 visits. Rescue with additional or modified doses of concomitant PD medications or use of CD-LD products other than the dispensed study medication are not permitted and will trigger discontinuation from the study.

Figure 1: Study Flow Chart

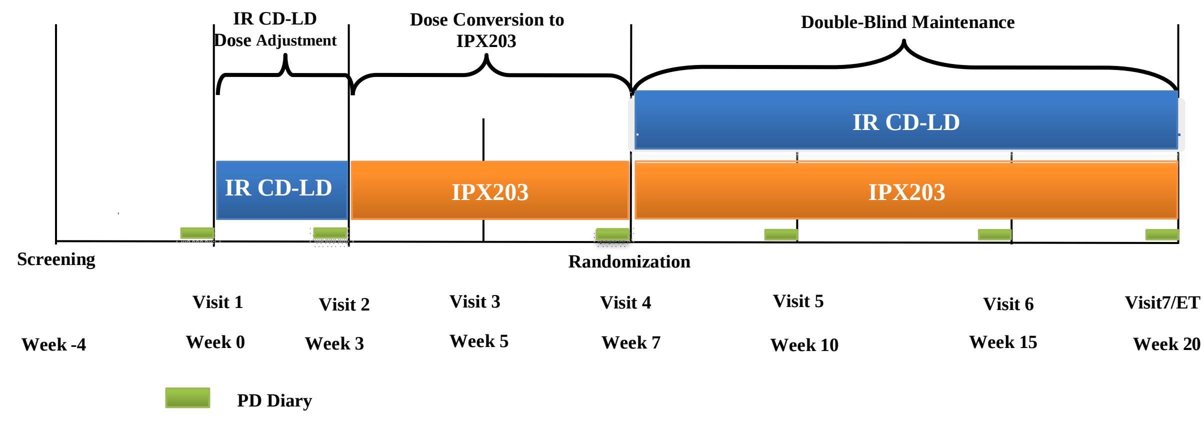

Abbreviations: IR=immediate-release, CD=carbidopa, LD=levodopa, ET=early termination

6.2. Number of Subjects

Assuming approximately an 18% prerandomization drop-out, approximately 510 subjects will be enrolled to randomize 420 subjects.

6.3. Treatment Assignment

Investigational product: IPX203 (carbidopa-levodopa) Extended-Release Capsules containing 35-140 mg of CD-LD, for oral administration.

Reference therapy: Immediate-Release carbidopa-levodopa (IR CD-LD) tablets containing 25-100 mg of CD-LD, for oral administration.

Subjects will be randomly assigned to one of two parallel treatment arms to receive either IPX203 (with matching IR CD-LD placebo) or IR CD-LD (with matching IPX203 placebo) during the double-blind maintenance therapy portion of the study.

6.4. Dosing and Dose Determination Criteria

IR CD-LD will be supplied as tablets containing 25-100 mg of CD-LD. IR CD-LD tablets may be split to achieve the required dose.

IPX203 will be supplied as capsules containing 35-140 mg of CD-LD. The suggested doses and regimen of IPX203 are intended to provide an onset of effect comparable to the subject's prestudy IR LD regimen and to extend the duration of effect. A 25-100 mg dose of IR CD-LD converts to a 70-280 mg CD-LD dose of IPX203.

6.4.1. IR CD-LD Dose Adjustment Period

During the 3-week IR CD-LD dose adjustment period, the initial dosing regimen of IR CD-LD will be the same as the subject's stable prestudy regimen unless the subject is taking a single daily bedtime dose of CR CD-LD, in which case, the CR CD-LD dose will be discontinued and substituted with a 1:1 milligram-equivalent dose of IR CD-LD. A "bedtime dose" is defined as the last daytime dose of CD-LD taken within 1 hour of onset of the subject's normal nighttime sleep period. Subjects who were receiving IR CD-LD as a 1:10 CD-LD formulation will be started on IR CD-LD with a 1:4 ratio at the same frequency and LD dose. The dosing regimen may be adjusted during the dose adjustment period. Any adjustments to the IR CD-LD dosing regimen will be done in consultation with the Investigator or qualified site personnel and will be recorded. The subject must be on a stable dosing regimen (no change in dose or in dosing frequency) of IR CD-LD for at least 5 days prior to returning for Visit 2.

6.4.2. IPX203 Dose Conversion Period

During the 4-week IPX203 dose conversion period, the suggested initial dosing regimen of IPX203 will be based on the subject's dosing regimen of IR CD-LD at the end of the dose adjustment period (Visit 2). A 25-100 mg dose of IR CD-LD converts to a 70-280 mg CD-LD dose of IPX203, and a half tablet (12.5-50-mg dose of IR CD-LD) converts to a 35-140 mg CD-LD dose of IPX203. To facilitate conversion of subjects from IR CD-LD to IPX203, Table 3 presents recommended starting dose regimens. It is recommended that IPX203 should be dosed approximately every 8 hours. The dosing regimen of IPX203 may be adjusted during the dose conversion period to achieve the optimal balance of efficacy and tolerability (minimize "Off" time without causing troublesome dyskinesia or other dopaminergic side effects). The subject must be on a stable dosing regimen of IPX203 (no change in dose or in dosing frequency) for at least 5 days prior to returning for Visit 4.

Table 3: Recommended Starting IPX203 LD Dosing Regimen Based on the Dosing Regimen of IR CD-LD at the End of the Dose Adjustment Period

Most Frequent IR CD-LD Unit Dose (mg)	Recommended Starting IPX203 Daily Dosing Regimen CD-LD (mg) Every 8 Hours
25-100	70-280 mg (2 × 35-140 mg)
31.25-125 – 37.5-150	105-420 mg (3 × 35-140 mg)
43.75-175 – 50-200	140-560 mg (4 × 35-140 mg)
56.25-225 – 62.5-250	175-700 mg (5 × 35-140 mg)
≥ 68.75-275	210-840 mg (6 × 35-140 mg)

Any changes to the dosing regimen should only be made by the Investigator or qualified site personnel.

When two or more IR CD-LD doses correspond to the most frequent IR CD-LD dose, the suggested IPX203 conversion should be based on the higher of the IR CD-LD doses.

6.4.3. Double-Blind Maintenance Period

During the 13-week double-blind double-dummy maintenance period, subjects receive either IPX203 (with matching IR CD-LD placebo) or IR CD-LD (with matching IPX203 placebo) with the stable dosing regimen established at the end of Week 3 (Visit 2) for IR CD-LD and at the end of Week 7 (Visit 4) for IPX203.

6.5. Criteria for Study Termination

The Sponsor has the right to terminate this study and remove all study material from the study site at any time for medical or administrative reasons. The Sponsor will endeavor to give adequate notice to allow safe withdrawal of subjects from the study.

7. SELECTION AND WITHDRAWAL OF SUBJECTS

Each subject must meet all of the following inclusion and exclusion criteria to qualify for enrollment.

7.1. Subject Inclusion Criteria

1. Male or female subjects diagnosed at age ≥ 40 years with PD, consistent with the United Kingdom Parkinson's Disease Society Brain Bank Diagnostic Criteria (Appendix B) and who are being treated with stable regimens of CD-LD but experiencing motor fluctuations.
2. Able to provide written informed consent prior to the conduct of any study-specific procedures.
3. Female subjects of childbearing potential must have a negative urine pregnancy test at Screening Visit.
4. Negative urine screen for drugs of abuse and negative alcohol breath test at Screening.
5. Hoehn and Yahr Stages 1, 2, 3, or 4 in the "On" state (part of Movement Disorders Society version of the Unified Parkinson's Disease Rating Scale [MDS-UPDRS] Part III; Appendix D).
6. Agrees to use a medically acceptable method of contraception throughout the study and for 6 weeks after completing the study. Medically acceptable methods of contraception that may be used by the subject and/or partner include but are not limited to: abstinence, oral contraception, NuvaRing or transdermal systems, diaphragm with vaginal spermicide, intrauterine device, condom and partner using vaginal spermicide, surgical sterilization (6 months), progestin implant or injection, or postmenopausal female (no menstrual period for > 2 years) or vasectomy (> 6 months).
7. Montreal Cognitive Assessment (MoCA) score ≥ 24 at Screening Visit in "On" state (Appendix C).
8. By history, for the 4 weeks prior to Screening, the subject experiences daily "wearing-off" episodes with periods of bradykinesia in combination with at least one of rest tremor or rigidity, experiences an "Off" state upon awakening on most mornings, and reports an average of at least 2.5 cumulative hours per day of "Off" time during the waking hours.
9. Able to differentiate "On" state from "Off" state as determined by at least 75% concordance with a trained rater in "On/Off" ratings for 8 ratings over the 4-hour training period. The concordance must include at least 1 "On" and 1 "Off" rating and must be achieved within two 4-hour training sessions.
10. At Visit 1, review of the 3-day PD Diaries confirms the following: that the subject is able to properly complete the diaries with valid entries; and that the subject has an <u>average of at least 2.5 hours</u> per day of "Off" time during the waking hours over the 3 days with at least 1.5 hours of cumulative "Off" time on each day. Inability to properly complete the

diaries is indicated when more than 1 day of a diary is not returned or when more than 2 hours (4 half-hour periods) of one 24-hour diary day are missing (Appendix O).

11. Responsive to CD-LD therapy and currently being treated on a stable regimen with CD-LD for at least 4 weeks prior to Visit 1 and:
    a. Requires at least 100 mg of LD from IR CD-LD for the first morning dose
    b. Requires a total daily dose of at least 400 mg of LD and takes a maximum total daily dose of 2400 mg LD, from IR CD-LD alone or IR CD-LD in combination with a single daily bedtime dose of CR CD-LD
    c. Has a dosing frequency of 4 to 9 times daily of CD-LD
    d. By history, typically experiences an "On" response with the first dose of IR CD-LD of the day, but the efficacy of this dose typically lasts less than 4 hours.
12. At Screening, the subject has predictable "Off" periods defined by a score of 1 or 2 on Item #4.5 (Complexity of Motor Fluctuations) of the MDS-UPDRS Part IV B (Motor Fluctuations) (Appendix D).
13. At Screening, the MDS-UPDRS Part III total score in the "Off" state is at least 20 units.
14. Able and willing to comply with the protocol, including completion of diaries and availability for all study visits.

7.2. Subject Exclusion Criteria

1. Used any doses of controlled-release (CR) CD-LD apart from a single daily bedtime dose within 4 weeks prior to Visit 1.
2. Used any doses of Rytary for the past 4 weeks prior to Visit 1 or considered IPX066 or Rytary failures for reasons of efficacy or safety.
3. Received any investigational medications within 30 days or 5 times the half-life, whichever is longer, prior to Visit 1.
4. Female subjects who are currently breastfeeding or lactating.
5. Had prior neurosurgical treatment for PD or if such procedure is planned or anticipated during the study period.
6. Allergic to any excipient in the study drugs (See Appendix P).
7. History of medical conditions or of a prior surgical procedure that would interfere with LD absorption, such as gastrectomy, proximal small-bowel resection, or bariatric surgery.
8. History of upper gastrointestinal hemorrhage in patients with peptic ulcer disease within the past 5 years.
9. History of glaucoma with intraocular pressures that are elevated despite appropriate medical management.
10. History of seizure or epilepsy and experienced at least 1 seizure during the past 12 months or has not been compliant with medically recommended therapy or visits.
11. History of myocardial infarction with residual atrial, nodal, or ventricular arrhythmias that are not controlled with medical and/or surgical interventions. A recent

(≤ 12 months) history of myocardial infarction with secondary arrhythmias is exclusionary regardless of the therapeutic control.

12. History of neuroleptic malignant syndrome or of nontraumatic rhabdomyolysis.
13. Liver enzyme values ≥ 2.5 times the upper limit of normal; or history of severe hepatic impairment.
14. Serum creatinine level ≥ 1.75 times the upper limit of normal; or requires dialysis at the time of Screening.
15. Subject with a history of malignant melanoma or with a suspicious undiagnosed skin lesion which in the opinion of the investigator could be melanoma.
16. History of drug or alcohol abuse within the 12 months prior to Screening.
17. Received within 4 weeks of Visit 1 or planning to take during participation in the clinical study:
    a. any doses of a controlled-release (CR) LD apart from a single daily bedtime dose, any doses of Rytary, additional CD (eg, Lodosyn) or benserazide (eg, Serazide), or catechol-O-methyl transferase inhibitors (entacapone or tolcapone) or medications containing these inhibitors (Stalevo),
    b. nonselective monoamine oxidase inhibitors (MAOI), apomorphine, or antidopaminergic agents, including antiemetics.
18. Treatment with any dopamine antagonist for the purposes of treating psychosis or bipolar disorder within the last 2 years or any history of psychosis within the past 10 years regardless of treatment. A brief, self-limited episode of psychosis precipitated by a medical intervention with return to normal mentation is not exclusionary. Mild PD-associated illusions are not exclusionary provided that they do not occur more than twice per week and the subject does not lose insight.
19. Employees or family members of the investigator, study site, or sponsor.
20. Subjects who have previously participated in an IPX203 study.
21. Subjects who, in the opinion of the clinical investigator, should not participate in the study.
22. Based on clinical assessment, subject does not adequately comprehend the terminology needed to complete the PD diary.

7.3. Subject Withdrawal Criteria

Site personnel should make every effort to conduct all protocol-specific procedures to complete the study. A subject may be discontinued from the study due to the following reasons:

1. Withdrawal by subject
2. Adverse event (AE)
3. Lack of efficacy
4. Study terminated by Sponsor

5. Protocol deviation
6. Noncompliance with study drug
7. Lost to follow-up
8. Death
9. Other

Subjects who withdraw early from the study will not be replaced. The reason or reasons for discontinuation will be specified and documented. Empty medication bottles and any unused study drug upon discontinuation will be collected. Study medication dispensed to a discontinued subject may not be redispensed to a different subject.

8. STUDY PROCEDURES

The procedures to be performed at each study visit are described below and summarized in Table 4.

Table 4: Events Schedule for Impax Study IPX203-B16-02

		3 Weeks of IR CD-LD Dose Adjustment	4 Weeks of IPX203 Dose Conversion			13 Weeks of Double-Blind Therapy		
**Assessment**	**Screening**	**Visit 1**	**Visit 2**	**Visit 3**	**Visit 4 Randomization**	**Visit 5**	**Visit 6**	**Visit 7 / Study Exit/Early Termination[b]**
Study Week[a]	**–4**	**0**	**3**	**5**	**7**	**10**	**15**	**20**
ICF & HIPAA Authorization[c]	X							
Contact IWRS	X	X	X	X	X	X	X	X
Randomization					X			
Inclusion/Exclusion	X	X						
Medical History	X							
Physical Examination	X							X
Vital Signs[d]	X	X	X	X	X	X	X	X
Height and Weight	X					X[e]		X[e]
C-SSRS[f]	X	X	X	X	X	X	X	X
Clinical Laboratory Tests[g]	X					X		X
Urine Pregnancy Test	X							
Urine Screen for Drug Abuse	X							
Alcohol Breath Test	X							
ECG	X					X		X
MoCA[h]	X							
MDS-UPDRS Parts I-IV	X[i]	X	X		X	X	X	X
PGI-C[j]						X	X	X
CGI-C[k]						X	X	X
PGI-S[l]		X			X			X

Assessment	Screening	3 Weeks of IR CD-LD Dose Adjustment	4 Weeks of IPX203 Dose Conversion			13 Weeks of Double-Blind Therapy		
		**Visit 1**	**Visit 2**	**Visit 3**	**Visit 4 Randomization**	**Visit 5**	**Visit 6**	**Visit 7 / Study Exit/Early Termination[b]**
Study Week[a]	**–4**	**0**	**3**	**5**	**7**	**10**	**15**	**20**
CGI-S[m]		X			X			X
PDQ-39[n]		X			X		X	X
GCSI[o]		X						X
NMSS[p]		X			X		X	X
PDSS-2[q]		X			X		X	X
PAS[r]		X			X		X	X
PD Diary Training; Perform Concordance Testing at Screening Only[s]	X	X	X	X	X	X	X	
Dispense PD Diaries[t]	X	X		X	X	X	X	
Review PD Diaries[u]		X	X		X	X	X	X
Reminder phone calls[v,w]	X[v]	X[w]	X[w]	X[w]	X[w]	X	X	X
Dispense study medication		X	X	X	X	X	X	
Collect empty medication bottles and any unused study drug/Perform study drug accountability			X	X	X	X	X	X
Adverse Events	X	X	X	X	X	X	X	X
Concomitant Medications	X	X	X	X	X	X	X	X

CGI-C = Clinical Global Impression of Change, CGI-S = Clinical Global Impression of Severity, C-SSRS = Columbia-Suicide Severity Rating Scale, ECG = electrocardiogram, GCSI = Gastroparesis Cardinal Symptom Index, HIPAA = Health Insurance Portability and Accountability Act, ICF = informed consent form, IWRS = interactive web response system, MDS-UPDRS = MDS version of Unified Parkinson's Disease Rating Scale, MoCA = Montreal Cognitive Assessment, PAS = Parkinson Anxiety Scale, PD = Parkinson's disease, NMSS = Non-Motor Symptom assessment scale for PD, PDQ-39 = 39-Item Parkinson's Disease Questionnaire, PDSS-2 = Parkinson's Disease Sleep Scale-2, PGI-C = Patient Global Impression of Change, PGI-S = Patient Global Impressions of Severity.

[a] The interval between Screening and Visit 1 (Day 1) should not exceed 4 weeks. Study visits should occur within ± 3 days of their specified timing.
[b] Study Exit procedures to be conducted at the end of Visit 7 or during an early termination visit.
[c] Subjects enrolled at sites in the United States (US) must sign HIPAA authorization prior to the conduct of any study-specific procedures.
[d] Record vital signs (blood pressure, heart rate, respiratory rate, and temperature [Screening and Study Exit only]) after subject has been resting supine for at least 5 minutes, then record orthostatic blood pressure and heart rate after subject has been standing for approximately 2 minutes. At Visits 1 and 4, orthostatic vital signs (blood pressure and heart rate) will be performed in triplicate, each set separated by at least 15 minutes from the previous set.
[e] Weight only.
[f] C-SSRS: Columbia Suicide Severity Rating Scale. See Appendix N.
[g] See Appendix Q.
[h] Montreal Cognitive Assessment in the "On" state: see Appendix C.
[i] At Screening MDS-UPDRS Parts I through IV will be done in both the "On" and "Off" state (see Appendix D).
[j] See Appendix E.
[k] See Appendix F.
[l] See Appendix G.
[m] See Appendix H.
[n] See Appendix I.
[o] See Appendix J.
[p] See Appendix K.
[q] See Appendix L.
[r] See Appendix M.
[s] Train at Screening and then as needed at subsequent visits. Perform concordance testing at Screening.
[t] Dispense PD Diaries at Screening and Visits 1, 3, 4, 5, and 6. Call subjects 4 days prior to Visits 1, 2 and 4-7 to remind them to complete PD Diaries. Subjects record diary information for 3 consecutive days immediately prior to each of the visits (Days -3, -2, and -1). Call subjects the day prior to each visit to remind them to bring the PD Diaries, empty medication bottles, and any unused study drug to the office.
[u] Review PD Diaries at Visits 1, 2, and 4-7.
[v] Post-Screening reminder phone call: Notify individuals who successfully complete screening procedures following review of all study entry criteria and clinical laboratory results that they may continue in the study. The interval between Screening and Visit 1 should not exceed 4 weeks.
[w] Reminder phone calls for Visits 1 through 4: In addition to the calls discussed above, make regular phone calls (approximately every 1 to 3 days) to subjects throughout the IR CD-LD dose-adjustment and IPX203 dose-conversion periods to evaluate each subject's adjustment to the study medication regimen.

8.1. Screening Visit

After the subject has signed the informed consent (and HIPAA authorization for US subjects only), complete the following procedures and assessments:

- Obtain an identification number from the Interactive Web Response System (IWRS). The IWRS will assign a 6-digit ID number to each subject, consisting of a 3-digit number representing the investigative site and a 3-digit sequential subject number.
- Review and record study entry criteria (Section 7).
- Perform urine pregnancy test for females of childbearing potential.
- Perform urine screen for drugs of abuse.
- Perform alcohol breath test.
- Complete medical history.
- Perform physical examination, including height and weight.
- Assess vital signs after subject is supine for at least 5 minutes (blood pressure, heart rate, temperature and respiratory rate) and then assess orthostatic blood pressure and heart rate after subject is standing (for approximately 2 minutes).
- Record current CD-LD regimen, other PD medications and their dosing schedule, and other concomitant medications.
- Record AEs.
- Perform a 12-lead ECG.
- Administer C-SSRS (Appendix N).
- Determine MoCA Score in the "On" state (Appendix C).
- Collect blood and urine samples for clinical laboratory studies (Appendix Q).
- Determine Hoehn and Yahr staging of PD in the "On" state (part of MDS-UPDRS Part III Motor Examination) (Appendix D).
- Administer MDS-UPDRS Parts I through IV in the "On" and "Off" state (Appendix D).
- Train the subject how to complete the PD Diaries to assess his/her "On" and "Off" states, including assessment of any dyskinesia. After training the subject, perform the concordance testing. The subject's "On"/"Off" ratings must agree at least 75% of the time with the trained rater during the training sessions. That is, the subject's "On"/"Off" ratings must agree with the trained rater's ratings on at least 75% "On"/"Off" states in a single session to qualify for study inclusion. The 75% concordance rate must be based on at least four ratings, and must include at least one "On" and one "Off" state. The ratings should occur every 30 minutes and each session can last up to 4 hours. If the subject fails the first training session, the subject may be trained for one additional 4-hour training session.

- Dispense PD Diaries and instruct the subject to complete the PD Diaries on 3 consecutive days immediately prior to Visit 1.

Notify individuals who successfully complete screening procedures following review of all study entry criteria and clinical laboratory results.

The interval between Screening and Visit 1 (Day 1) should not exceed 4 weeks.

8.2. Visit 1 – Start of IR CD-LD Dose Adjustment

8.2.1. Prior to Visit 1

Contact the subject at least 4 days prior to Visit 1 to remind him/her to complete the 3-day PD Diaries starting 3 consecutive days immediately prior to Visit 1.

The day prior to Visit 1, remind subjects to:

- Bring their completed 3-day PD Diaries to the clinic.

8.2.2. At Visit 1

For Visit 1 complete the following procedures:

- Collect and review the subject's 3-day PD Diaries. Ensure that the subject is averaging at least 2.5 hours per day of "Off" time over 3 days and at least 1.5 hours of "Off" time on each day based on the 3 day PD Diaries. If the subject cannot properly complete the diary, eg, if more than 1 day of the diary is not returned or if more than 1 day of the diary is not valid (ie, more than 2 hours [4 half periods] of the 24-hour diary are missing), he/she will not continue in the study.
- Review inclusion and exclusion criteria to ensure that the subject continues to meet study inclusion criteria.
- Review instruction of 3-day PD Diaries if needed.
- Measure vital signs (respiratory rate, blood pressure, and heart rate after supine for at least 5 minutes, and orthostatic pulse and blood pressure after standing for approximately 2 minutes). Orthostatic vital sign measurements (blood pressure and heart rate) will be performed in triplicate, each set separated by at least 15 minutes from the previous set.
- Administer C-SSRS (Appendix N).
- Administer MDS-UPDRS Parts I through IV (Appendix D).
- Complete PDQ-39 (Appendix I).
- Complete PGI-S (Appendix G).
- Complete CGI-S (Appendix H).
- Complete GCSI (Appendix J).
- Complete Non-motor Symptom assessment scale for PD (NMSS) (Appendix K).

- Complete the Parkinson's Disease Sleep Scale-2 (PDSS-2) (Appendix L).
- Complete Parkinson Anxiety Scale (PAS) (Appendix M).
- Record any AEs and update changes in concomitant medication since the previous visit.
- Dispense PD Diaries.
- Contact IWRS and dispense study medication per IWRS instructions.

8.2.3. Post Visit 1

- Make regular phone calls (approximately every 1 to 3 days) while the IR CD-LD dose is being adjusted. The IR CD-LD dosing regimen should be stable for at least 5 days prior to returning for Visit 2.

8.3. Visits 2 (Week 3) and Visit 3 (Week 5) – IPX203 Dose Conversion

8.3.1. Prior to Visit 2

- Call subjects 4 days prior to Visit 2 and remind them to complete their PD Diaries.
- Call subjects the day prior to Visit 2 to remind them to bring the PD Diaries, empty medication bottles, and any unused study drug to the office.

8.3.2. Prior to Visit 3

- Call subjects the day prior to Visit 3 to remind them to bring back empty medication bottles and any unused study drug to the office.

8.3.3. At Visits 2 and 3

For Visits 2 and 3 complete the following procedures:

- Measure vital signs (respiratory rate, blood pressure, and heart rate after supine for at least 5 minutes, and orthostatic pulse and blood pressure after standing for approximately 2 minutes).
- Administer C-SSRS (Appendix N).
- Collect empty medication bottles and any unused study drug; perform study drug accountability.
- Record and update AEs and concomitant medications.
- Contact IWRS and dispense study medication per IWRS instructions.

Additional Assessments at Visit 2 Only

- Administer MDS-UPDRS Parts I through IV (Appendix D).
- Review PD Diaries. The subject will be terminated from the study if the subject does not average at least 2.5 hours per day of "Off" time over 3 days and at least 1.5 hours of "Off" time on each day based on the 3 day PD Diaries and/or if the subject cannot

properly complete the diary, eg, if more than 1 day of the diary is not returned or if more than 1 day of the diary is not valid (ie, more than 2 hours [4 half periods] of the 24 hour diary are missing).

- Ensure that the subject has been on a stable dosing regimen for at least 5 days prior to dose conversion to IPX203. If the subject is not able to achieve a stable dosing regimen lasting at least 5 days, the subject will be discontinued.
- Conduct PD Diaries training, if needed.

Additional Activities at Visit 3 Only

- Dispense PD Diaries.
- Review instruction of 3-day PD Diaries if needed.

8.3.4. Post Visits 2 and 3

Make regular phone calls (approximately every 1 to 3 days) to subjects throughout the dose conversion period, as needed, to evaluate each subject's adjustment to the study medication. The IPX203 dosing regimen should be stable for at least 5 days prior to returning for Visit 4.

8.4. Visit 4 (Week 7) – Randomization

8.4.1. Prior to Visit 4

- Call subjects 4 days prior to Visit 4 to remind them to complete their PD Diaries.
- Contact subjects 1 day prior to Visit 4 to remind them to bring back the PD Diaries, empty medication bottles, and any unused study drug to the office.

8.4.2. At Visit 4

For Visit 4 complete the following procedures:

- Review PD Diaries. At least 1 day of valid diary data (ie, less than 2 hours [4 half periods] of the 24-hour diary are missing) must be available, otherwise the subject will be terminated from the study.
- Ensure that the subject has been on a stable dosing regimen for at least 5 days prior to randomization. If the subject is not able to achieve a stable dosing regimen lasting at least 5 days, the subject will be discontinued.
- Measure vital signs (respiratory rate, blood pressure, and heart rate after supine for at least 5 minutes, and orthostatic pulse and blood pressure after standing for approximately 2 minutes). Orthostatic vital sign measurements (blood pressure and heart rate) will be performed in triplicate, each set separated by at least 15 minutes from the previous set.
- Administer C-SSRS (Appendix N).
- Administer MDS-UPDRS Parts I through IV (Appendix D).
- Complete PDQ-39 (Appendix I).

- Complete PGI-S (Appendix G).
- Complete CGI-S (Appendix H).
- Complete NMSS (Appendix K).
- Complete PDSS-2 (Appendix L).
- Complete PAS (Appendix M).
- Record and update AEs and concomitant medications.
- Conduct PD diaries training, if needed.
- Collect empty medication bottles and any unused study drug; perform study drug accountability.
- Contact IWRS for randomization and dispense medication per IWRS instructions.
- Dispense PD diaries.

8.5. Visit 5 (Week 10) and Visit 6 (Week 15)

8.5.1. Prior to Visit 5 and 6

- Call subjects 4 days prior to Visits 5 and 6 to remind them to begin recording in their PD diaries on each of the 3 consecutive days immediately prior to each of these visits.
- Call the subjects the day prior to Visits 5 and 6 to remind the subjects to bring in their PD diaries, empty medication bottles, and any unused study drug to the office.

8.5.2. At Visit 5 and 6

For Visits 5 and 6 complete the following procedures (note visit-specific tasks below):

- Measure vital signs (respiratory rate, blood pressure, and heart rate after supine for at least 5 minutes, and orthostatic pulse and blood pressure after standing for approximately 2 minutes).
- Administer C-SSRS (Appendix N).
- Administer MDS-UPDRS Parts I through IV (Appendix D).
- Complete PGI-C (Appendix E).
- Complete CGI-C (Appendix F).
- Review PD diaries.
- Conduct PD diaries training, if needed.
- Dispense PD diaries.
- Collect empty medication bottles and any unused study drug; perform study drug accountability.
- Contact IWRS and dispense study medication per IWRS instructions.

- Record any AEs and update changes in concomitant medication since the previous visit.

<u>Additional Activities at Visit 5 Only:</u>

- Record weight.
- Perform a 12-lead ECG.
- Collect blood and urine samples for clinical laboratory studies (Appendix Q).

<u>Additional Activities at Visit 6 Only:</u>

- Complete PDQ-39 questionnaire (Appendix I).
- Complete NMSS (Appendix K).
- Complete the PDSS-2 (Appendix L).
- Complete PAS (Appendix M).

8.6. Visit 7 (Week 20) – End of Study/Study Exit

8.6.1. Prior to Visit 7

- Call subjects 4 days prior to Visit 7 to remind them to begin recording in their PD Diaries on each of the 3 consecutive days immediately prior to Visit 7.
- Call the subjects the day prior to Visit 7 to remind the subjects to bring in their PD Diaries, empty medication bottles, and any unused study drug to the office.

8.6.2. At Visit 7

All enrolled subjects must complete Study Exit procedures at the end of Visit 7 or during an early termination visit:

- Perform physical examination, including weight.
- Measure vital signs (temperature, respiratory rate, blood pressure, and heart rate after supine for at least 5 minutes, and orthostatic pulse and blood pressure after standing for approximately 2 minutes).
- Collect and review 3-day PD Diaries.
- Administer C-SSRS (Appendix N).
- Administer MDS-UPDRS Parts I through IV (Appendix D).
- Complete PGI-C (Appendix E).
- Complete CGI-C (Appendix F).
- Complete PDQ-39 questionnaire (Appendix I).
- Complete NMSS (Appendix K).
- Complete PDSS-2 (Appendix L).

- Complete PAS (Appendix M).
- Complete PGI-S (Appendix G).
- Complete CGI-S (Appendix H).
- Complete GCSI (Appendix J).
- Collect empty medication bottles and any unused study drug; perform study drug accountability.
- Record and update AEs and concomitant medications.
- Collect blood and urine samples for clinical laboratory studies (Appendix Q).
- Perform a 12-lead ECG.
- Contact IWRS to record subject disposition.

8.7. Early Termination

8.7.1. Subjects Who Terminate Prior to Randomization

If the subject discontinues the study prior to randomization (Visit 4) the subject should complete the following assessments:

- Perform physical examination, including weight.
- Measure vital signs (temperature, respiratory rate, blood pressure, and heart rate after supine for at least 5 minutes, and orthostatic pulse and blood pressure after standing for approximately 2 minutes).
- Collect and review 3-day PD Diaries when available.
- Administer C-SSRS (Appendix N).
- Administer MDS-UPDRS Parts I through IV (Appendix D).
- Collect empty medication bottles and any unused study drug; perform study drug accountability.
- Record and update AEs and concomitant medications.
- Collect blood and urine samples for clinical laboratory studies (Appendix Q).
- Perform a 12-lead ECG.
- Contact IWRS to record subject disposition.

8.7.2. Subjects Who Terminate Early after Randomization

If the subject discontinues the study after randomization (Visit 4), the subject should complete all assessments described in Section 8.6.2.

8.8. Blood Volume

Safety blood draws: Approximately 10 mL of blood will be drawn at Screening, Visit 5, and at Study Exit, for a combined total of 30 mL.

9. TREATMENT OF SUBJECTS

9.1. Description of Study Drug

Study drugs will be provided by Impax for this study:

- IPX203 (carbidopa-levodopa) Extended-Release Capsules containing 35-140 mg of CD-LD for oral administration. The CD-LD ratio is 1:4. In addition, matching placebo capsules will also be provided.
- Immediate-release carbidopa-levodopa (IR CD-LD) tablet containing 25-100 mg of CD-LD, for oral administration. In addition, matching placebo tablets will also be provided.

Table 5: Study Drugs for Study IPX203-B16-02

Investigational Product	Dosage Strength (mg CD-LD) and Form
IPX203 (carbidopa-levodopa) Extended-Release capsules	35-140 mg<br>Capsules for oral administration
IR CD-LD (carbidopa-levodopa) tablets	25-100 mg<br>Tablets for oral administration
IPX203 Placebo capsules	Capsules for oral administration
IR CD-LD Placebo tablets	Tablets for oral administration

9.2. Concomitant Medications

9.2.1. Permitted PD Medications

Concomitant therapy with amantadine, selective monoamine oxidase (MAO) type B inhibitors (eg, selegiline, rasagiline), anticholinergic PD medications (eg, benztropine, trihexyphenidyl), and/or dopamine agonists (except apomorphine) is allowed provided the doses and regimens have been stable for at least 4 weeks prior to Visit 1 and the therapy is intended to be constant throughout the course of the study.

9.2.2. Prohibited Medications and Procedures

Prohibited medications and procedures include the following:

- Received within 4 weeks of Visit 1 or planning to take during participation in the clinical study: any doses of a controlled-release (CR) CD-LD apart from a single daily bedtime dose or any doses of Rytary, additional CD (eg, Lodosyn) or benserazide (eg, Serazide), or catechol-O-methyl transferase inhibitors (entacapone or tolcapone) or medications containing these inhibitors (Stalevo).

- Rescue with additional or modified doses of concomitant PD medications or with use of CD-LD products other than the dispensed study medications are not permitted and will trigger discontinuation from the study.
- Received within 4 weeks of Visit 1 or planning to take during participation in the clinical study: nonselective MAOI, selective MAO type A inhibitor (eg, phenelzine, moclobemide, pirlindole, bifemelane, toloxatone), apomorphine, or antidopaminergic agents including antiemetics.
- Treatment with any dopamine antagonist antipsychotic agents for the purposes of psychosis or bipolar disorder within the last 2 years. Use of antipsychotics to treat conditions other than psychosis or bipolar disorders may be allowed only after consultation with the medical monitor.
- Any neurosurgical procedure for the treatment of PD during the course of the study.

A subject who reports the use of any prohibited medications or procedure will be discontinued.

All medications taken within 30 days prior to signing the informed consent form (ICF) and all concomitant medications taken during the study will be recorded on the case report form (CRF).

9.2.3. Rescue Medications

Subjects who need rescue medications or need to change treatment will be discontinued from the study. Rescue with additional or modified doses of concomitant PD medications or with use of CD-LD products other than the dispensed study medications are not permitted and will trigger discontinuation from the study. Rescue medications are not allowed during the dose adjustment, dose conversion or double-blind treatment periods.

9.3. Treatment Compliance

Study drug accountability and reconciliation will be performed by the study staff and the study monitor(s).

9.4. Randomization and Blinding

At Visit 4, subjects will be randomized, stratified by center, in a 1:1 ratio into one of two double-blind parallel treatment arms of IPX203 (and matching IR CD-LD placebo) or IR CD-LD (and matching IPX203 placebo).

10. STUDY DRUG MATERIALS AND MANAGEMENT

10.1. Study Drug

Study drugs include the investigational treatment IPX203 35-140 mg CD-LD (and matching placebo capsules) and the active comparator treatment IR 25-100 mg CD-LD, (and matching placebo tablets).

IPX203 is an extended-release (ER) capsule formulation of CD-LD. Impax will manufacture and provide the IPX203 and matching placebo.

IR CD-LD is commercially available and will be provided by Impax. Matching placebo tablets will be manufactured and provided by Impax.

10.2. Study Drug Packaging and Labeling

Impax or designee will provide study medications in bottles with appropriate labeling affixed.

Labels on the study medication may include the following information:

- name, address, and phone number of the sponsor
- pharmaceutical dosage form/route of administration, quantity of dosage units, the name/identifier, and strength/potency
- batch and/or code number to identify the contents and packaging operation
- trial reference code (protocol number)
- trial subject identification number/treatment number and where relevant, the visit number
- name of investigator
- directions for use: Take tablet(s) or capsule(s) orally with water as directed.
- for clinical trial use only
- storage information: Store at 25°C (77°F), with excursions permitted to 15°C to 30°C (59°F to 86°F). Protect from light and moisture.
- period of use (use-by date, expiry date or retest date as applicable), in month/year format and in a manner that avoids any ambiguity.
- keep out of reach of children
- caution statement: Caution: New Drug—Limited by Federal (or United States) law to investigational use.

10.3. Study Drug Storage

The clinical site should store the study drug at 25°C (77°F), with excursions permitted to 15°C to 30°C (59°F to 86°F). The study drug should be stored in a tightly closed container, protected

from light and moisture. Storage temperature excursions above 30°C (86°F) should be reported by the clinical site to Impax or its designee.

10.4. Study Drug Administration

Subjects will be instructed to take their medications with approximately 240 mL of room-temperature water. The capsules or tablets should not be crushed or chewed.

IR CD-LD tablets may be split to achieve the required doses.

10.5. Study Drug Dispensing and Accountability

The Investigator must ensure that all study medication received at the study site is inventoried and accounted for, and that dispensed study medication is recorded in the subject's source documents, the CRF, and the study medication inventory log. Site personnel must not relabel or reassign study medication to other subjects or to individuals not enrolled in the study. The study monitor verifies medication accountability during monitoring visits.

10.6. Study Drug Handling and Disposal

The Investigator must retain and properly store all partially used and unused study medication until authorized by Impax regarding disposition.

11. ASSESSMENT OF EFFICACY

11.1. Parkinson's Disease Diary

Subjects are to record "asleep," "Off," and "On" without or with (nontroublesome or troublesome) dyskinesias during waking hours every 30 minutes over a 24-hour day. In the PD Diaries, subjects are instructed to indicate for each half-hour their predominant state during most of that period. "Off" is defined as the typical functional state when the medication is no longer providing benefit with regard to mobility, slowness, and stiffness in spite of taking medications. "On" is defined as the typical functional state when a subject has received medication and the medication is providing benefit with regard to mobility, slowness, and stiffness. Dyskinesias are defined as involuntary and irregular twisting and/or turning movements. Dyskinesia movements are usually an effect of medication and occur during "On" time. Nontroublesome dyskinesias do not interfere with function or do not cause meaningful discomfort. Troublesome dyskinesias do interfere with function or do cause meaningful discomfort.

11.2. Patient and Investigator Global Assessments

- Patient Global Impression of Change (Appendix E): The patient will compare his/her condition from the start of the study on a 7-point scale ranging from "Very much worse" (1) to "Very much improved" (7) at the time of the assessment.
- Clinical Global Impression of Change (Appendix F): The clinician will compare the subjects' condition from the start of the study on a 7-point scale ranging from "Very much worse" (1) to "Very much improved" (7) at the time of the assessment.
- Patient Global Impression of Severity (Appendix G): The patient will determine the severity of the disease on a 7-point scale ranging from "Normal, not at all ill" (1) to "Extremely severely ill" (7) at the time of the assessment.
- Clinical Global Impression of Severity (Appendix H): The clinician will determine the severity of the disease on a 7-point scale ranging from "Normal, not at all ill" (1) to "Among the most extremely ill of subjects" (7) at the time of the assessment.

11.3. Movement Disorders Society Version of Unified Parkinson's Disease Rating Scale

The MDS-UPDRS has 4 parts:

- Part I: Non-Motor Aspects of Experiences of Daily Living (nM-EDL) has 2 components. Component IA contains a number of behaviors assessed by the investigator with all pertinent information from the patients and caregivers. Component IB is completed by the patient with or without help from the caregiver but independent of the investigator. These sections can be reviewed by the rater to ensure all questions are answered clearly and the rater can help explain any ambiguities.

- Part II: Motor Aspects of Experiences of Daily Living (M-EDL) is a self-administered questionnaire but can be reviewed by the investigator to ensure completeness and clarity.
- Part III: Motor Examination assesses the motor signs of PD and has instructions for the rater to give to or to demonstrate to the patient. It is completed by the rater.
- Part IV: Motor Complications integrates patient-derived information with the rater's clinical observations and judgements and is completed by the rater. It contains instructions for the rater and instructions to be read to the patient.

11.4. Additional Assessments

- Parkinson's Disease Questionnaire-39 (PDQ-39) is a self-reported questionnaire. Using the 39-items, 8 domains are defined: mobility (Questions 1-10), activities of daily living (ADL) (Questions 11-16), emotional well-being (Questions 17-22), stigma (Questions 23-26), social support (Questions 27-29), cognition (Questions 30-33), communication (Questions 34-36) and bodily discomfort (Questions 37-39).
- Non-Motor Symptom assessment scale for Parkinson's Disease (NMSS) is a 30-item investigator rated questionnaire. The NMSS contains 9 domains: cardiovascular (Questions 1, 2), sleep/fatigue (Questions 3-6), mood/cognition (Questions 7-12), perceptual problems (Questions 13-15), attention/memory (Questions 16-18), gastrointestinal (Questions 19-21), urinary (Questions 22-24), sexual function (Questions 25, 26), and miscellaneous (Questions 27-30).
- Parkinson's Disease Sleep Scale-2 (PDSS-2) is 15-item self-reported questionnaire. Three domains are defined: disturbed sleep (Questions 1-3, 8, 14), motor symptoms at night (Questions 4-6, 12, 13), PD symptoms at night (Questions 7, 9-11, 15).
- Parkinson Anxiety Scale (PAS) is a 12-item patient or observer rated questionnaire with 3 domains: persistent anxiety (Questions A.1-A.5), episodic anxiety (Questions B.1-B.4) and avoidance anxiety (Questions C.1-C.3).

12. ASSESSMENT OF SAFETY

12.1. Safety Parameters

Safety will be assessed by the following parameters:

- Electrocardiograms (ECGs), clinical laboratory tests, physical examinations, the Columbia-Suicide Severity Rating Scale (C-SSRS), and vital signs, including supine and standing orthostatic blood pressure and heart rate.
- Adverse events and concomitant medications will be evaluated throughout the course of the study.

12.2. Adverse Events

12.2.1. Definition of Adverse Event

An adverse event (adverse experience) is any untoward medical occurrence in a patient or clinical trial subject administered a medicinal product and which does not necessarily have to have a causal relationship with this treatment. An adverse event can therefore be any unfavorable and unintended sign (eg, an abnormal laboratory finding), symptom, or disease temporally associated with the use of a medicinal product, whether or not considered related to the medicinal product.

All AEs and any clinically significant physical examination findings, 12-lead ECG abnormalities, or clinical laboratory measurements occurring during the study that were not present prior to administration of study medication and that continue at Study Exit should be followed and evaluated with additional tests, if necessary, until the AEs are medically stable or resolved. Follow-up on these AEs should be recorded on the source documents and reported to Impax.

12.2.2. Recording Adverse Events

Elicit information about AEs with nonselective questions such as: “Have you experienced any changes in your health status since your last visit?” Encourage subjects to report AEs at onset.

Record information for any AE that emerges from the time the subject signs the ICF until Study Exit.

Monitor each subject closely for the development of AEs and record all such events on the AE page of the CRF. Whenever possible, group signs and symptoms that constitute a single diagnosis. For example, cough, rhinitis, and sneezing might be grouped as upper respiratory infection.

For each AE, record the onset date, severity, seriousness, relationship to study medication, date of resolution (or continuing), action taken, and outcome in the CRF. The Investigator is to make a causality assessment (relationship to study medication) for every AE.

12.2.3. Follow-up

The Investigator must follow each AE until resolved or medically stable.

12.2.4. Relationship to Study Drug

The Investigator documents his/her opinion of the relationship of the AE to the study medication as follows:

- Not Related—the experience can be readily explained by the subject's underlying medical condition or concomitant medications and no relationship exists between the study medication and the experience.
- Unlikely Related—the temporal relationship between the AE and the administration of the study medication is uncertain and it is likely that the AE can be explained by the subject's medical condition or other therapies.
- Possibly Related—there is some logical temporal relationship between the AE and the administration of the study medication and the experience is unlikely to be explained by the subject's medical condition or other therapies.
- Related—the temporal relationship is compelling between the administration of the study medication and the AE cannot be explained by the subject's medical condition or other therapies.

12.2.5. Assessment of Severity

Grade each AE for severity and note in the description of the AE. Determine the severity category of mild, moderate, or severe, as defined below, and enter the information on the AE page of the CRF.

- Mild—causing no limitation of usual activities
- Moderate—causing some limitation of usual activities
- Severe—causing inability to carry out usual activities

12.3. Serious Adverse Events

12.3.1. Definition of Serious Adverse Event

A serious adverse event (SAE) is any AE occurring at any dose that results in any of the following outcomes, regardless of relationship to the study medication:

- Death
- A life-threatening adverse drug experience
- Inpatient hospitalization or prolongation of existing hospitalization
- A persistent or significant disability/incapacity
- A congenital anomaly/birth defect

- Important medical events that may not result in death, be life-threatening, or require hospitalization may be considered an SAE when, based upon appropriate medical judgment, they may jeopardize the subject and may require medical or surgical intervention to prevent one of the outcomes listed in this definition.

12.3.2. Reporting Serious Adverse Events

Any SAE that occurs from the time the subject signs an ICF until 30 days after taking the final dose of study medication must be reported by the investigative staff to the Sponsor or the Sponsor's representative within 24 hours of knowledge of the event (see Study Contact Information).

An SAE form must be completed and sent to the Sponsor and/or the Sponsor's representative. All SAEs must also be recorded on the AE page of the CRF. Additionally, all SAEs must be reported to the institutional review board (IRB) per the IRB's requirements.

Those SAEs that are considered both serious and unexpected and related to the study drug are subject to expedited reporting. An "unexpected AE" is any AE where the nature or severity is not consistent with the current investigator brochure (IB) or if an IB is not required or available, the specificity or severity is not consistent with the provided risk information.

Unexpected fatal or life-threatening SAEs related to the study drug must be reported by the Sponsor to the appropriate regulatory authority in an expedited manner (ie, first report within 7 days of first knowledge by the Sponsor). The Sponsor will provide a final written report to that authority within 15 days of initial receipt of information on the event. The Sponsor or the Sponsor's representative will also inform all participating Investigators of the SAE.

Unexpected SAEs that are not fatal or life-threatening must be reported by the Sponsor to the appropriate regulatory authority as soon as possible but no later than 15 calendar days after first knowledge of the SAE by the Sponsor. The Sponsor or the Sponsor's representative also informs all participating Investigators of the SAE.

Subjects withdrawn from the study due to any SAE will be followed until the SAE is resolved or medically stable. Record all SAEs, regardless of severity and whether or not related to the study medication, on the appropriate page of the CRF.

The Investigator must determine whether the seriousness of the event warrants removal of the subject from the study. He/she should, in any case, institute appropriate diagnostic and therapeutic measures and keep the subject under observation for as long as is medically indicated, or refer the subject to appropriate health professionals.

12.4. Pregnancy

Any pregnancy that occurs from the time the subject signs an ICF until 30 days after taking the final dose of study medication must be reported within 24 hours to the Sponsor or the Sponsor's representative and the subject should be terminated from the study. All pregnancies will be followed through to delivery of the infant. If the subject experiences a termination of the pregnancy, it should be reported as defined in Section 12.3.2.

12.5. Other Safety Parameters and Related Information

Additional safety parameters (laboratory tests, 12-lead ECGs, physical examinations, and vital signs), the C-SSRS, the GCSI, and concomitant medications are collected as shown in the Schedule of Assessments in Table 4. Clinical laboratory assessments are listed in Appendix Q.

13. STATISTICS

13.1. Study Design and Sample Size Estimation

This is a multicenter, randomized, double-blind, double-dummy, active-controlled, parallel-group study. Assuming a difference of 1 hour between IPX203 and IR CD-LD in “Good on” time and a standard deviation of the treatment differences to be 3.0 hours, a sample size of 210 subjects per arm will be needed to ensure at least 90% power at a 0.05 significance level.

Assuming approximately an 18% prerandomization drop-out, approximately 510 subjects would need to be enrolled to randomize 420 subjects.

13.2. Demographics/Baseline Comparability

The demographics and baseline characteristics will be summarized by treatment arms and overall using descriptive statistics. Demographics information includes age, sex, and race. Baseline disease characteristics include MDS-UPDRS Parts I, II, III, and IV, Hoehn and Yahr stage, MoCA scores, and age of onset of PD. Distributions of dosing information, including LD doses and years on LD, will also be summarized.

13.3. Efficacy Endpoints

- Primary endpoint: Change from baseline in “Good on” time in hours per day, averaged over the PD Diary days, at the end of double-blind treatment period (Visit 7 or early termination). “Good on” time is derived from the 3-day PD Diaries and is defined as the sum of “On” time without dyskinesia and “On” time with nontroublesome dyskinesia.
- Key secondary endpoints:
  - Change from baseline in “Off” time in hours per day, averaged over the PD Diary days at the end of double-blind treatment period (Visit 7 or early termination)
  - Proportion of subjects with either “much improved” or “very much improved” in Patient Global Impression of Change (PGI-C) scores at the end of double-blind treatment period (Visit 7 or early termination)
  - Change from baseline in the MDS-UPDRS Part III at the end of double-blind treatment period (Visit 7 or early termination)
  - Change from baseline in the sum of MDS-UPDRS Parts II and III at the end of double-blind treatment period (Visit 7 or early termination)
- Additional endpoints:

  The following endpoints will be evaluated (at the post-randomization visits) as change from baseline (Visit 4) as well as change from the study entry (Visit 1), when applicable, by visits:
  - Percent “Off” time during waking hours derived from the 3-day PD Diaries
  - Average duration of each continuous “Good on” and each continuous “On”

- Hours of (1) "Off" time (from Visit 1), (2) "Good on" time (from Visit 1), (3) "On" time with dyskinesia, (4) "On" time with troublesome dyskinesia, and (5) "On" time with nontroublesome dyskinesia, and (6) asleep time derived from the 3-day PD Diaries
- Proportion of subjects with an improvement in "Good on" time of at least 1, 1.5, 2, 2.5, and 3 hours
- Proportion of subjects with a reduction in "Off" time of at least 0.5, 1, 1.5, 2, 2.5, and 3 hours
- Proportions of subjects who are "On" upon awakening and "Good on" upon awakening
- Average time to "On" upon awakening
- Change from baseline in the average number of motor fluctuations per day averaged over the PD Diary days. A motor fluctuation is defined as a change from "Off" to "On" state or from "On" to "Off" state.
- MDS-UPDRS total score (sum of Parts I, II, III, and IV) and Parts I, II, and IV separately
- MDS-UPDRS Part III and Parts II + III combined (from Visit 1)
- MDS-UPDRS Part II Question 2.9
- PDQ-39 total score and individual domain scores
- NMSS total score and individual domains
- PDSS-2 total score and individual domains
- PDSS-2 items 9, 10, 11, 12, and 13 combined
- PAS total score and individual domains
- PGI-S
- Proportion of subjects with either "severely ill" or "extremely severely ill" on the PGI-S
- CGI-S
- Proportion of subjects with either "severely ill" or "among the most extremely ill of subjects" on the CGI-S
- PGI-C scores
- CGI-C scores
- Proportion of subjects with either "much improved" or "very much improved" on the CGI-C

13.4. Analysis of Efficacy Data

In order to control the type I error rate, the primary efficacy endpoint and key secondary efficacy endpoints will be tested in a single hierarchical order as detailed in Section 13.8.

13.4.1. Primary Efficacy Endpoint

The primary efficacy endpoint is the mean change from baseline in "Good on" time in hours per day, averaged over the PD Diary days, at the end of the double-blind therapy (Visit 7 or early termination). "Good on" time is derived from the 3-day PD Diaries. For each day, "Good on" time is calculated by adding the number of half-hour intervals in which either an "On without dyskinesia" or "On with nontroublesome dyskinesia" is checked.

The primary efficacy endpoint will be analyzed using a mixed model for repeated measures (MMRM). The model will include baseline (Visit 4) "Good on" time as a covariate, treatment, visit, and pooled center as fixed effects, and a treatment-by-visit interaction. The model will employ an unstructured within subject covariance matrix and a restricted maximum likelihood (ReML) estimation method. The degree-of-freedom of the denominator will be estimated using the Kenward-Roger method (Kenward and Roger 1997). The primary analysis population will be the modified intent-to-treat as defined in Section 13.9. Missing data will be handled as in Section 13.10.

13.4.2. Key Secondary Efficacy Endpoints

The first key secondary endpoint is the mean change from baseline in "Off" time in hours per day, averaged over the PD Diary days, at the end of the double-blind therapy (Visit 7 or early termination). "Off" time is derived from the 3-day PD Diaries. For each day, "Off" time is calculated by adding the number of half-hour intervals in which an "Off" is checked. This endpoint will be analyzed using a MMRM model with baseline (Visit 4) "Off" time as a covariate, treatment, visit, and pooled center as fixed effects, and a treatment-by-visit interaction. The model will employ an unstructured within subject covariance matrix and a restricted maximum likelihood (ReML) estimation method. The degree-of-freedom of the denominator will be estimated using the Kenward-Roger method (Kenward and Roger 1997).

The proportion of subjects with either "much improved" or "very much improved" in PGI-C scores at the end of the double-blind therapy (Visit 7 or early termination), the second key secondary endpoint, will be analyzed using a Cochran-Mantel-Haenszel test to compare the two treatment groups with pooled center as a stratification factor.

The mean change from baseline in the MDS-UPDRS Part III at the end of the double-blind therapy (Visit 7 or early termination) is the third key secondary endpoint. This endpoint will be analyzed using an MMRM model with baseline (Visit 4) MDS-UPDRS Part III as a covariate, treatment, visit, and pooled center as fixed effects, and a treatment-by-visit interaction. The model will employ an unstructured within subject covariance matrix and a restricted maximum likelihood (ReML) estimation method. The degree-of-freedom of the denominator will be estimated using the Kenward-Roger method (Kenward and Roger 1997).

The mean change from baseline in sum of the MDS-UPDRS Parts II and III at the end of the double-blind therapy (Visit 7 or early termination) is the fourth key secondary endpoint. This

endpoint will be analyzed using an MMRM model with baseline (Visit 4) MDS-UPDRS Parts II and III combined as covariates, treatment, visit, and pooled center as fixed effects, and a treatment-by-visit interaction. The model will employ an unstructured within subject covariance matrix and a restricted maximum likelihood (ReML) estimation method. The degree-of-freedom of the denominator will be estimated using the Kenward-Roger method (Kenward and Roger 1997).

For the 4 key secondary endpoints, the analysis population will be the modified intent-to-treat as defined in Section 13.9. Missing data will be handled as in Section 13.10.

13.4.3. Additional Efficacy Endpoints

In general, continuous endpoints will be summarized by standard descriptive statistics (mean, standard deviation, median, minimum, and maximum). Categorical endpoints will be summarized by frequencies and percentages. Comparisons between the two arms will be explored using appropriate statistical methodologies. Details will be provided in the SAP.

The primary endpoint, key secondary endpoints, as well as other efficacy endpoints will be presented by visit over the whole blinded treatment period from Baseline (Visit 4) to the end of the double-blind treatment period (Visit 7).

Other additional efficacy endpoints collected postrandomization will be analyzed by visit in a fashion similar to the primary and key secondary endpoints.

Additionally the PGI-C and CGI-C will be analyzed using analysis of variance (ANOVA) with treatment and pooled center as factors.

13.5. Center Pooling Algorithm

The center pooling algorithm is as follows.

1. Sort centers from each country from smallest to largest based on the number of subjects in the modified intent-to-treat analysis set (mITT).
2. Centers with less than 5 mITT subjects or at least one mITT subject per treatment group will be pooled with the next smallest center in the same country until the combined center (namely, pseudo-center) has more than 5 mITT subjects and at least one mITT subject per treatment group.
3. If after pooling within the same country, the pseudo-center still has less than 5 mITT subjects or still has zero subjects in one of the treatment group, that pseudo-center will be pooled with the next smallest center in the same geographical region (Western Europe, Eastern Europe, North America).
4. If after pooling within the same geographical region, the pseudo-center still has less than 5 mITT subjects or still has zero subjects in one of the treatment group, that pseudo-center will be pooled with the next smallest center in any region.

The process continues until all pooled pseudo-centers have at least 5 mITT subjects and at least one mITT subject per treatment group. These pooled centers will be used in analyses that adjust for pooled centers.

This pooling algorithm will be detailed in the Statistical Analysis Plan (SAP).

13.6. Sensitivity Analyses of the Primary Endpoint and Key Secondary Endpoints

Sensitivity analyses will be performed with respect to the primary efficacy endpoint and continuous key secondary endpoints ("Off" time, MDS-UPDRS Part III, and MDS-UPDRS Parts II and III combined) as follows.

13.6.1. Assessing Assumptions of the Mixed Model for Repeated Measures (MMRM)

a. The normality and homoscedasticity assumptions will be examined through residual analyses. The normality and homoscedasticity assumptions will further be tested via Shapiro-Wilk (Shapiro and Wilk 1965) and Levene (Levene 1960) tests, respectively. If normality and/or homoscedasticity assumption appears violated, then:

   i. Nonparametric Wilcoxon Rank Sum test will be performed to compare the two treatment groups, with missing data imputed by the last observation carried forward (LOCF) method.

   ii. Multiple imputation rank based analysis: instead of missing data imputed by the LOCF method, in this analysis, missing data at Visit 7 will be imputed multiple times to create 50 complete datasets. The multiple imputation procedure is described in Section 13.6.4 (part of the pattern-mixture model), using $f = 0\%$. The Wilcoxon Rank Sum test will be performed on each of the 50 datasets. The results are then combined using Rubin's rule (Rubin 1987) via SAS PROC MIANALYZE.

b. If the model fails to converge due to the unstructured covariance matrix, a simpler covariance matrix will be employed in the order of 1) heterogeneous Toeplitz [SAS PROC MIXED type = TOEPH], 2) heterogeneous autoregressive of order 1 [type = ARH(1)], 3) heterogeneous compound symmetry [type = CSH], 4) Toeplitz [type = TOEP], 5) autoregressive of order 1 [type = AR(1)], 6) compound symmetry [type = CS]. The first covariance structure which does not have the convergence problem will be the one used for the primary analysis.

c. Missing at Random (MAR) assumption will be evaluated as discussed in Section 13.6.4.

13.6.2. Complete Case Analysis

The primary endpoint will be analyzed using an ANCOVA model with "Good on" time at baseline (Visit 4) as a covariate, pooled center and treatment as factors. The model will be performed on subjects with <u>both</u> baseline "Good on" time and Visit 7 "Good on" time.

13.6.3. Single LOCF/BLOCF Imputation

The primary efficacy endpoint will be analyzed using an ANCOVA model with "Good on" time at Visit 4 as a covariate, pooled center and treatment as factors. Missing data will be imputed by the LOCF and baseline observation carried forward (BLOCF) methods. These analyses will be performed on the mITT population.

13.6.4. Pattern-Mixture Model

If an overall dropout rate postrandomization is > 15%, pattern-mixture models (PMM) will be employed to assess the robustness of the results under the missing not at random (MNAR) assumption. The pattern for PMM is defined by patients' last visit with an observed primary efficacy endpoint and the reason for dropout.

Multiple imputation with mixed missing data mechanism (MNAR for a missing data pattern and MAR for others) will be used to investigate the robustness of the primary result. Four specific data patterns will be examined:

1. Dropout at Visit 5 and reason = Lack of efficacy in IPX203 treatment arm,
2. Dropout at Visit 5 and reason = Lack of efficacy or adverse events in IPX203 treatment arm,
3. Dropout at Visit 6 and reason = Lack of efficacy in IPX203 treatment arm,
4. Dropout at Visit 6 and reason = Lack of efficacy or adverse events in IPX203 treatment arm.

The missing values will be imputed 50 times (multiple imputation) under the assumption that the distribution of the missing values is the same as that of the observed values. The PMM then investigates the departure from the MAR assumption by progressively decreasing the outcome (the "penalty") for those on IPX203 arm who fall into an assumed MNAR pattern above. For the dropout subjects on IPX203 arm that fall into one of the patterns above, the "penalty" is obtained by subtracting the imputed missing data after dropout by a factor *f*, with *f* starts from 0%, 5%, 10%, 15%, 20%, 25%, 30%, …, 100% of the treatment difference seen in the primary model. This process continues until the conclusion from the primary analysis is overturned (a tipping point). In other words, if the dropout subject is from IPX203 arm and the dropout pattern falls into one of the 4 patterns above, then the subject's imputed value will be adjusted downward by a factor *f*, where *f* goes from 0% to 100% of the treatment difference seen in the primary model. Note that if 0% is used, the analysis is essentially multiple imputation under MAR assumption. On the other hand, if 100% is used, then the analysis is essentially a "jump to reference" where outcome on IPX203 arm is assumed to be the same as outcome on IR CD-LD. After imputations, the dataset will be analyzed using an MMRM model similar to the primary analysis model. The results will then be combined using the Rubin's rule (Rubin 1987) via SAS PROC MIANALYZE.

The procedure will be carried out in SAS as follows:

a. Use Monte Carlo Markov Chain (MCMC) method in SAS PROC MI by treatment group to impute the intermittent missing data to form monotone missingness.
b. Use MAR-based multiple imputation in SAS PROC MI to impute the missing data (SAS MONOTONE statement).
c. For dropout subjects in IPX203 arm who fall into an MNAR pattern specified above, a delta which equals to *f* times the treatment difference obtained from the primary MMRM analysis at Visit 7 will be subtracted from their imputed values for all visits after the dropout ("penalizing" IPX203 arm).

d. After imputation, use the MMRM model as in the primary analysis model to analyze the complete data along with the imputed data.

e. Repeat steps a through d 50 times.

Combine results using Rubin's rule (Rubin 1987) via SAS PROC MIANALYZE.

13.7. Subgroup Analyses

The primary, key secondary endpoints, as well as overall summary of adverse events, will be examined for the following subgroups.

- Age: < 65, ≥ 65 years old at study entry
- Sex: Males, Females
- Race: Caucasians, non-Caucasians

Additionally, the following subgroups may be examined:

- Region
- Ethnicity
- Concomitant medications
- Weight
- Body mass index (BMI)
- PD duration
- Age of PD onset
- "Good On" time and "Off" time at study entry.

For all subgroup efficacy analyses, the same analysis methods as the primary and key secondary endpoints will be applied, unless the sample size in one of the subgroups becomes too small to hinder the statistical analysis. In that case, no inferential statistics will be provided for such a subgroup. The details for final subgroup analyses will be documented in the SAP.

13.8. Multiplicity Adjustments

The primary endpoint and 4 key secondary endpoints will be tested in a sequential hierarchical order as follows.

1. The primary endpoint, the mean change from baseline in "Good on" time (hours per day), will be tested first at a 0.05 level of significance.
2. If statistical significance is demonstrated, then the first key secondary endpoint, the mean change from baseline in "Off" time (hours per day), will be tested next at a 0.05 level of significance.
3. If statistical significance is demonstrated, then the second key secondary endpoint, the proportion of subjects with either "much improved" or "very much improved" on the PGI-C, will be tested next at a 0.05 level of significance.

4. If statistical significance is demonstrated, then the third key secondary endpoint, the mean change from baseline in the MDS-UPDRS Part III, will be tested at a 0.05 level of significance.
5. If statistical significance is demonstrated, then the fourth key secondary endpoint, the mean change from baseline in the sum of the MDS-UPDRS Parts II and III combined will be tested next at a 0.05 level of significance.

For the other efficacy endpoints, no adjustment will be made.

13.9. Analysis Populations

13.9.1. Safety Analysis Set

The Safety Analysis set will include all subjects who were treated with any study drug.

13.9.2. Intent-to-Treat Analysis Set

The Intent-to-treat Analysis Set will include all subjects who were randomized and treated with any study drug and have a baseline and at least one postbaseline efficacy assessment.

13.9.3. Modified Intent-to-Treat Analysis Set

The Modified Intent-to-treat Analysis Set will include all subjects who were randomized and treated and have a valid baseline PD Diary and at least one valid postrandomization PD Diary. This analysis set will be used for the primary analysis and key secondary analyses.

13.9.4. Completers Analysis Set

The Completers Analysis Set will include all subjects who were randomized and treated and complete the study.

13.10. Handling of Missing Data

13.10.1. Missing Data for PD Diaries

An MMRM approach will be used to handle missing visit data. MMRM analysis will use all available valid visit data, including subjects with some missing visit data, in order to arrive at an estimate of the mean treatment effect.

A PD Diary is valid if at least 1 day of diary data are available using the rules defined below.

Imputation of missing data for a PD Diary day will be required if a PD Diary is not completed for a full day (6 am to 5:30 am). In this case, the method of imputation will be dependent upon the amount and pattern of missing data:

1. If more than 4 half-hour time intervals are missing, then that particular day will not be included in the analysis.
2. If a one-half hour time interval is missing and the observations on either side of the time interval are not missing, then the missing time interval will be imputed by assigning a

value of the previous measurement for the first 15 minutes and the value of the next measurement for the second 15 minutes.

3. If 2, 3, or 4 consecutive half-hour time intervals are missing, and these time intervals are available from other days of the visit then the following rules will be applied:
   a. For missing time intervals on Day 1, data from Day 2 will be used for imputation for the same time intervals. If Day 2 data is also incomplete, then Day 3 data will be used.
   b. For missing time intervals on Day 2, data from Day 3 will be used for imputation if available; otherwise Day 1 data will be used.
   c. Data from Day 2 will be used for imputing missing time intervals on Day 3. If data from Day 2 is not available, then Day 1 data will be used for imputation.
   d. If data at the same time period are missing across all days, then the approach will be to split the individual missing half-hour intervals into 2 periods, with the first-half interval being imputed with data from the immediate previous nonmissing time period and the second-half interval being imputed with the next nonmissing time interval.

13.10.2. Missing Data for Global Assessments (PGI-C, CGI-C, PGI-S and CGI-S)

For subjects with missing PGI-C or CGI-C for a particular visit, the data will be imputed as nonresponders (ie, not being “much improved” or “very much improved”).

For subjects with missing PGI-S or CGI-S for a particular visit, the data will be imputed as nonresponders (ie, being “severely ill” or “extremely severely ill” for PGI-S and being “severely ill” or “among the most extremely ill of subjects” for CGI-S).

13.10.3. Missing Data for MDS-UPDRS

If the MDS-UPDRS are missing for the particular visit, the missing data will be handled via the MMRM model.

If component questions are missing for a particular part of the MDS-UPDRS questionnaire, the missing items are assigned the average value for other items in that part as follows (Goetz 2015):

- For Part I (13 questions): up to 1 missing question will be imputed using the average value of the remaining 12 questions.
- For Part II (13 questions): up to 2 missing questions will be imputed using the average value of the remaining 11 questions.
- For Part III (33 questions): up to 7 missing questions will be imputed using the average value of the remaining 26 questions.
- Part IV (6 questions): no imputation is done.

If more component questions are missing than above for a particular part of the MDS-UPDRS questionnaire, the entire questionnaire will not be included in the analysis for that particular

assessment. Missing data will be handled in a fashion similar to PD Diary data (Section 13.10.1) using the MMRM model.

For quality-of-life endpoints, missing responses within a questionnaire will not be imputed.

13.11. Analysis of Safety

The safety analysis will include all subjects who receive at least 1 dose of study medication. Reported AEs will be coded using the Medical Dictionary for Regulatory Activities (MedDRA). All AEs will be summarized by system organ class and preferred terms within system organ class. The severity, seriousness, and relationship to study medication will also be summarized by treatment arms. Each AE (based on preferred term) is counted once for a given subject. If the same AE occurred on multiple occasions, the highest severity and least complimentary relationship will be assumed.

The incidence of treatment-emergent AEs and serious AEs will be summarized by treatment arms.

Additionally, laboratory test data, physical examinations, vital signs, ECGs, C-SSRS, and GCSI will be summarized by treatment arms.

14. ADMINISTRATIVE PROCEDURES

14.1. Guidelines for Good Clinical Practice

This study will be conducted in accordance with principles of Good Clinical Practice (GCP) as promulgated by the ICH. Good Clinical Practice is an international ethical and scientific quality standard for designing, conducting, recording, and reporting trials that involve the participation of human subjects. Compliance with this standard provides public assurance that the rights, safety, and well-being of human subjects are protected under current ethical principles, and that the clinical trial data are credible. Current GCP standards may be found in ICH Guidance E6 (Good Clinical Practice: Consolidated Guidance). This guidance describes the principles of GCP and the obligations of the institutional review board (IRB), the Investigator and the Sponsor in conducting this study in accordance with those principles.

14.2. Institutional Review Board Approval

The review of this protocol by an IRB and the performance of all aspects of the study, including the methods used for obtaining informed consent, must be in accordance with principles enunciated in the ICH and GCP Guidelines and by the appropriate regulatory authorities.

The Investigator is responsible for preparing documents for submission to the relevant IRB and obtaining written approval for this study. Institutional Review Board approval must be obtained prior to the initiation of the study. The Investigator's continued participation in the study is contingent on renewing approval with the IRB at least annually.

14.3. Informed Consent

Site personnel should prepare an Informed Consent Form (ICF) incorporating the necessary elements of consent. The ICF is to be approved by Impax prior to submission to the IRB. The Investigator or his/her staff must explain the nature of the investigation and the risks involved to each subject prior to screening, and obtain a signed ICF. The subject should also be informed that he/she is free to voluntarily withdraw from the study at any time.

14.4. Study Monitoring

Impax representatives or designees will conduct site visits to the investigational facilities for the purpose of monitoring the study. The Investigator agrees to allow the monitor to inspect the drug storage area, study drug stocks, drug accountability records, subject charts and study source documents, and other records relevant to study conduct. The Investigator must permit access to such records if a regulatory or compliance audit is required.

14.5. Protocol Amendments

All amendments to the protocol must be documented in writing, reviewed and approved by the Sponsor and Investigator, and submitted to the IRB for approval prior to implementation. If the protocol amendment substantially alters the study design or potential risk to the subject, a new

written ICF for continued participation in the study must be obtained from each subject affected by the change.

14.6. Termination of Study

The Sponsor has the right to terminate this study and remove all study material from the site at any time for medical or administrative reasons. In this event, the Sponsor will endeavor to give adequate notice to allow safe withdrawal of subjects from the study.

14.7. Case Report Forms

Site personnel should collect and record data for the study as source documents, and transfer the data into the CRF.

The Investigator must ensure that complete data for the clinical study are collected and accurately documented in the appropriate sections of the CRF and adequately supported by the appropriate source documentation. In addition, it is the Investigator's responsibility to provide signatures where requested indicating concurrence with data in the CRF.

14.8. Investigator's Final Conduct Report

At the completion of the study, the Investigator must provide Impax a copy of the final conduct report that was submitted to their IRB, including a review of AEs.

14.9. Records Retention

International Conference on Harmonization, GCP, and US FDA guidelines require that essential documents be retained until at least 2 years after the last approval of a marketing application and until there are no pending or contemplated marketing applications, or at least 2 years have elapsed since the formal discontinuation of clinical development of the investigational product.

However, the essential documents should be retained for a longer period if required by the applicable regulatory requirements or by an agreement with the Sponsor. Records should never be destroyed without written approval from the Sponsor.

If an Investigator leaves the institution, he/she must transfer responsibilities for record retention to another individual willing to accept them. The Investigator must notify the Sponsor in writing of the transfer of study documents before the transfer of the study documents.

15. PUBLICATION POLICY

Study results may not be published without prior written approval from Impax.

16. LIST OF REFERENCES

Blanchet PJ, Calon F, Martel JC, et al. Continuous administration decreases and pulsatile administration increases behavioral sensitivity to a novel dopamine D2 agonist (U-91356A) in MPTP-exposed monkeys. J Pharmacol Exp Ther. 1995;272:854-859.

Engber TM, Susel Z, Juncos JL, Chase TN. Continuous and intermittent levodopa differentially affect rotation induced by D-1 and D-2 dopamine agonists. European Journal of Pharmacology. 1989;168:291-298.

Fahn S. Parkinson disease, the effect of levodopa, and the ELLDOPA trial. Earlier vs Later L DOPA. Arch Neurol. 1999 May;56(5):529-35.

Food and Drug Administration 2003. Guidance for Industry. Bioavailability and Bioequivalence Studies for Orally Administered Drug Products — General Considerations. U.S. Department of Health and Human Services. Food and Drug Administration. Center for Drug Evaluation and Research (CDER). March 2003.

Goetz CG, Luo S, Wang L, Tilley BC, LaPelle NR, Stebbins GT. Handling missing values in the MDS-UPDRS. Mov Disord. 2015 Oct;30(12):1632-8.

Juncos JL, Engber TM, Raisman R, et al. Continuous and intermittent levodopa differentially affect basal ganglia function. Ann Neurol. 1989;25:473-478.

Kenward MG and Roger JH. Small sample inference for fixed effects from restricted maximum likelihood. Biometrics. 1997;53:983-997.

Kurlan R, Rothfield KP, Woodward WR, Nutt JG, Miller C, Lichter D, et al. Erratic gastric emptying of levodopa may cause “random” fluctuations of parkinsonian mobility. Neurology. 1988;38(3):419-21.

Levene H. In contributions to probability and statistics: Essays in honor of Harold Hotelling, I. Olkin et al. eds. Stanford University Press. 1960: 278-292.

Melamed E, Bitton V, Zelig O. Delayed onset of responses to single doses of L-dopa in parkinsonian fluctuators on long-term L-dopa therapy. Clin Neuropharmacol. 1986;9(2):182-8.

Mizuno Y. Where do we stand in the treatment of Parkinson's disease? Neurology. 2007;254:13-18.

Nilsson D, Nyholm D, Aquilonius S-M. Duodenal levodopa infusion in Parkinson's disease--long-term experience. Acta Neurologica Scandinavica. 2001;104:343-348.

Nyholm D, Nilsson Remahl AI, Dizdar N, et al. Duodenal levodopa infusion monotherapy vs oral polypharmacy in advanced Parkinson disease. Neurology. 2005;64:216-223.

Rubin DB. Multiple Imputation in Nonresponse in Surveys. San Francisco, CA: Wiley; 1987.

Shapiro SS and Wilk MB. An analysis of variance test for normality (complete samples). Biometrika. 1965;52(3-4): 591-611.

Sinemet (Carbidopa-Levodopa) Tablets Prescribing Information. Whitehouse Station, NJ: Manufactured by Merck & Co., Inc.; Marketed by Bristol-Myers Squibb Company; 2013.

Stocchi F, Quinn NP, Barbato L, Patsalos PN, O'Connel MT, Ruggieri S, et al. Comparison between a fast and a slow release preparation of levodopa and a combination of the two: a clinical and pharmacokinetic study. Clinical Neuropharmacol. 1994;17(1):38-44.

Stocchi F, Vacca L, et al. Intermittent vs continuous levodopa administration in patients with advanced Parkinson disease: a clinical and pharmacokinetic study. Arch Neurol. 2005:62(6):905-910.

17. APPENDICES

APPENDIX A. PRESCRIBING INFORMATION FOR IR CD-LD

SINEMET®
(carbidopa levodopa)
Tablets

DESCRIPTION

SINEMET® (carbidopa levodopa) is a combination of carbidopa and levodopa for the treatment of Parkinson's disease and syndrome.

Carbidopa, an inhibitor of aromatic amino acid decarboxylation, is a white, crystalline compound, slightly soluble in water, with a molecular weight of 244.3. It is designated chemically as (—)-L-α-hydrazino-α-methyl-β-(3,4-dihydroxybenzene) propanoic acid monohydrate. Its empirical formula is $C_{10}H_{14}N_2O_4 \cdot H_2O$, and its structural formula is:

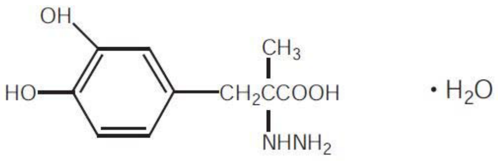

Tablet content is expressed in terms of anhydrous carbidopa which has a molecular weight of 226.3.

Levodopa, an aromatic amino acid, is a white, crystalline compound, slightly soluble in water, with a molecular weight of 197.2. It is designated chemically as (—)-L-α-amino-β-(3,4-dihydroxybenzene) propanoic acid. Its empirical formula is $C_9H_{11}NO_4$, and its structural formula is:

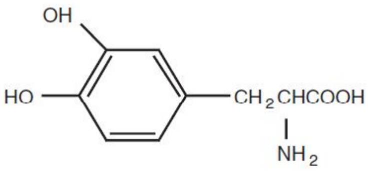

SINEMET is supplied as tablets in three strengths:

SINEMET 25-100, containing 25 mg of carbidopa and 100 mg of levodopa.

SINEMET 10-100, containing 10 mg of carbidopa and 100 mg of levodopa.

SINEMET 25-250, containing 25 mg of carbidopa and 250 mg of levodopa.

Inactive ingredients are hydroxypropyl cellulose, pregelatinized starch, crospovidone, microcrystalline cellulose, and magnesium stearate. SINEMET 10-100 and 25-250 Tablets also contain FD&C Blue #2/Indigo Carmine AL. SINEMET 25-100 Tablets also contain D&C Yellow #10 Lake.

CLINICAL PHARMACOLOGY

Mechanism of Action

Parkinson's disease is a progressive, neurodegenerative disorder of the extrapyramidal nervous system affecting the mobility and control of the skeletal muscular system. Its characteristic features include resting tremor, rigidity, and bradykinetic movements. Symptomatic treatments, such as levodopa therapies, may permit the patient better mobility.

Current evidence indicates that symptoms of Parkinson's disease are related to depletion of dopamine in the corpus striatum. Administration of dopamine is ineffective in the treatment of Parkinson's disease apparently because it does not cross the blood-brain barrier. However, levodopa, the metabolic precursor of dopamine, does cross the blood-brain barrier, and presumably is converted to dopamine in the brain. This is thought to be the mechanism whereby levodopa relieves symptoms of Parkinson's disease.

1

Pharmacodynamics

When levodopa is administered orally, it is rapidly decarboxylated to dopamine in extracerebral tissues so that only a small portion of a given dose is transported unchanged to the central nervous system. For this reason, large doses of levodopa are required for adequate therapeutic effect, and these may often be accompanied by nausea and other adverse reactions, some of which are attributable to dopamine formed in extracerebral tissues.

Since levodopa competes with certain amino acids for transport across the gut wall, the absorption of levodopa may be impaired in some patients on a high protein diet.

Carbidopa inhibits decarboxylation of peripheral levodopa. It does not cross the blood-brain barrier and does not affect the metabolism of levodopa within the central nervous system.

The incidence of levodopa-induced nausea and vomiting is less with SINEMET than with levodopa. In many patients, this reduction in nausea and vomiting will permit more rapid dosage titration.

Since its decarboxylase inhibiting activity is limited to extracerebral tissues, administration of carbidopa with levodopa makes more levodopa available for transport to the brain.

Pharmacokinetics

Carbidopa reduces the amount of levodopa required to produce a given response by about 75% and, when administered with levodopa, increases both plasma levels and the plasma half-life of levodopa, and decreases plasma and urinary dopamine and homovanillic acid.

The plasma half-life of levodopa is about 50 minutes, without carbidopa. When carbidopa and levodopa are administered together, the half-life of levodopa is increased to about 1.5 hours. At steady state, the bioavailability of carbidopa from SINEMET tablets is approximately 99% relative to the concomitant administration of carbidopa and levodopa.

In clinical pharmacologic studies, simultaneous administration of carbidopa and levodopa produced greater urinary excretion of levodopa in proportion to the excretion of dopamine than administration of the two drugs at separate times.

Pyridoxine hydrochloride (vitamin B_6), in oral doses of 10 mg to 25 mg, may reverse the effects of levodopa by increasing the rate of aromatic amino acid decarboxylation. Carbidopa inhibits this action of pyridoxine; therefore, SINEMET can be given to patients receiving supplemental pyridoxine (vitamin B_6).

Special Populations

Geriatric: A study in eight young healthy subjects (21-22 yr) and eight elderly healthy subjects (69-76 yr) showed that the absolute bioavailability of levodopa was similar between young and elderly subjects following oral administration of levodopa and carbidopa. However, the systemic exposure (AUC) of levodopa was increased by 55% in elderly subjects compared to young subjects. Based on another study in forty patients with Parkinson's disease, there was a correlation between age of patients and the increase of AUC of levodopa following administration of levodopa and an inhibitor of peripheral dopa decarboxylase. AUC of levodopa was increased by 28% in elderly patients (≥ 65 yr) compared to young patients (< 65 yr). Additionally, mean value of Cmax for levodopa was increased by 24% in elderly patients (≥ 65 yr) compared to young patients (< 65 yr) (see PRECAUTIONS, *Geriatric Use*).

The AUC of carbidopa was increased in elderly subjects (n=10, 65-76 yr) by 29% compared to young subjects (n=24, 23-64 yr) following IV administration of 50 mg levodopa with carbidopa (50 mg). This increase is not considered a clinically significant impact.

INDICATIONS AND USAGE

SINEMET is indicated in the treatment of Parkinson's disease, post-encephalitic parkinsonism, and symptomatic parkinsonism that may follow carbon monoxide intoxication or manganese intoxication.

Carbidopa allows patients treated for Parkinson's disease to use much lower doses of levodopa. Some patients who responded poorly to levodopa have improved on SINEMET. This is most likely due to decreased peripheral decarboxylation of levodopa caused by administration of carbidopa rather than by a primary effect of carbidopa on the nervous system. Carbidopa has not been shown to enhance the intrinsic efficacy of levodopa.

Carbidopa may also reduce nausea and vomiting and permit more rapid titration of levodopa.

CONTRAINDICATIONS

Nonselective monoamine oxidase (MAO) inhibitors are contraindicated for use with SINEMET. These inhibitors must be discontinued at least two weeks prior to initiating therapy with SINEMET. SINEMET may be administered concomitantly with the manufacturer's recommended dose of an MAO inhibitor with selectivity for MAO type B (e.g., selegiline HCl) (see PRECAUTIONS, *Drug Interactions*).

SINEMET is contraindicated in patients with known hypersensitivity to any component of this drug, and in patients with narrow-angle glaucoma.

WARNINGS

When SINEMET is to be given to patients who are being treated with levodopa, levodopa must be discontinued at least twelve hours before therapy with SINEMET is started. In order to reduce adverse reactions, it is necessary to individualize therapy. See DOSAGE AND ADMINISTRATION section before initiating therapy.

The addition of carbidopa with levodopa in the form of SINEMET reduces the peripheral effects (nausea, vomiting) due to decarboxylation of levodopa; however, carbidopa does not decrease the adverse reactions due to the central effects of levodopa. Because carbidopa permits more levodopa to reach the brain and more dopamine to be formed, certain adverse central nervous system (CNS) effects, e.g., dyskinesias (involuntary movements), may occur at lower dosages and sooner with SINEMET than with levodopa alone.

All patients should be observed carefully for the development of depression with concomitant suicidal tendencies.

SINEMET should be administered cautiously to patients with severe cardiovascular or pulmonary disease, bronchial asthma, renal, hepatic or endocrine disease.

As with levodopa, care should be exercised in administering SINEMET to patients with a history of myocardial infarction who have residual atrial, nodal, or ventricular arrhythmias. In such patients, cardiac function should be monitored with particular care during the period of initial dosage adjustment, in a facility with provisions for intensive cardiac care.

As with levodopa, treatment with SINEMET may increase the possibility of upper gastrointestinal hemorrhage in patients with a history of peptic ulcer.

Falling Asleep During Activities of Daily Living and Somnolence

Patients taking SINEMET alone or with other dopaminergic drugs have reported suddenly falling asleep without prior warning of sleepiness while engaged in activities of daily living (includes operation of motor vehicles). Road traffic accidents attributed to sudden sleep onset have been reported. Although many patients reported somnolence while on dopaminergic medications, there have been reports of road traffic accidents attributed to sudden onset of sleep in which the patient did not perceive any warning signs, such as excessive drowsiness, and believed that they were alert immediately prior to the event. Sudden onset of sleep has been reported to occur as long as one year after the initiation of treatment.

Falling asleep while engaged in activities of daily living usually occurs in patients experiencing pre-existing somnolence, although some patients may not give such a history. For this reason, prescribers should reassess patients for drowsiness or sleepiness especially since some of the events occur well after the start of treatment. Prescribers should be aware that patients may not acknowledge drowsiness or sleepiness until directly questioned about drowsiness or sleepiness during specific activities. Patients should be advised to exercise caution while driving or operating machines during treatment with SINEMET. Patients who have already experienced somnolence or an episode of sudden sleep onset should not participate in these activities during treatment with SINEMET.

Before initiating treatment with SINEMET, advise patients about the potential to develop drowsiness and ask specifically about factors that may increase the risk for somnolence with SINEMET such as the use of concomitant sedating medications and the presence of sleep disorders. Consider discontinuing SINEMET in patients who report significant daytime sleepiness or episodes of falling asleep during

activities that require active participation (e.g., conversations, eating, etc.). If treatment with SINEMET continues, patients should be advised not to drive and to avoid other potentially dangerous activities that might result in harm if the patients become somnolent. There is insufficient information to establish that dose reduction will eliminate episodes of falling asleep while engaged in activities of daily living.

Hyperpyrexia and Confusion

Sporadic cases of a symptom complex resembling neuroleptic malignant syndrome (NMS) have been reported in association with dose reductions or withdrawal of certain antiparkinsonian agents such as levodopa, carbidopa levodopa, or carbidopa levodopa extended release. Therefore, patients should be observed carefully when the dosage of levodopa is reduced abruptly or discontinued, especially if the patient is receiving neuroleptics.

NMS is an uncommon but life-threatening syndrome characterized by fever or hyperthermia. Neurological findings, including muscle rigidity, involuntary movements, altered consciousness, mental status changes; other disturbances, such as autonomic dysfunction, tachycardia, tachypnea, sweating, hyper- or hypotension; laboratory findings, such as creatine phosphokinase elevation, leukocytosis, myoglobinuria, and increased serum myoglobin have been reported.

The early diagnosis of this condition is important for the appropriate management of these patients. Considering NMS as a possible diagnosis and ruling out other acute illnesses (e.g., pneumonia, systemic infection, etc.) is essential. This may be especially complex if the clinical presentation includes both serious medical illness and untreated or inadequately treated extrapyramidal signs and symptoms (EPS). Other important considerations in the differential diagnosis include central anticholinergic toxicity, heat stroke, drug fever, and primary central nervous system (CNS) pathology.

The management of NMS should include: 1) intensive symptomatic treatment and medical monitoring and 2) treatment of any concomitant serious medical problems for which specific treatments are available. Dopamine agonists, such as bromocriptine, and muscle relaxants, such as dantrolene, are often used in the treatment of NMS; however, their effectiveness has not been demonstrated in controlled studies.

PRECAUTIONS

General

As with levodopa, periodic evaluations of hepatic, hematopoietic, cardiovascular, and renal function are recommended during extended therapy.

Patients with chronic wide-angle glaucoma may be treated cautiously with SINEMET provided the intraocular pressure is well-controlled and the patient is monitored carefully for changes in intraocular pressure during therapy.

Dyskinesia

Levodopa alone, as well as SINEMET, is associated with dyskinesias. The occurrence of dyskinesias may require dosage reduction.

Hallucinations / Psychotic-Like Behavior

Hallucinations and psychotic-like behavior have been reported with dopaminergic medications. In general, hallucinations present shortly after the initiation of therapy and may be responsive to dose reduction in levodopa. Hallucinations may be accompanied by confusion and to a lesser extent sleep disorder (insomnia) and excessive dreaming.

SINEMET may have similar effects on thinking and behavior. This abnormal thinking and behavior may present with one or more symptoms, including paranoid ideation, delusions, hallucinations, confusion, psychotic-like behavior, disorientation, aggressive behavior, agitation, and delirium.

Ordinarily, patients with a major psychotic disorder should not be treated with SINEMET, because of the risk of exacerbating psychosis. In addition, certain medications used to treat psychosis may exacerbate the symptoms of Parkinson's disease and may decrease the effectiveness of SINEMET.

Impulse Control / Compulsive Behaviors

Reports of patients taking dopaminergic medications (medications that increase central dopaminergic tone), suggest that patients may experience an intense urge to gamble, increased sexual urges, intense

urges to spend money, binge eating, and/or other intense urges, and the inability to control these urges. In some cases, although not all, these urges were reported to have stopped when the dose was reduced or the medication was discontinued. Because patients may not recognize these behaviors as abnormal, it is important for prescribers to specifically ask patients or the caregivers about the development of new or increased gambling urges, sexual urges, uncontrolled spending or other urges while being treated with SINEMET. Physicians should consider dose reduction or stopping the medication if a patient develops such urges while taking SINEMET [see *Information for Patients*].

Melanoma

Epidemiological studies have shown that patients with Parkinson's disease have a higher risk (2- to approximately 6-fold higher) of developing melanoma than the general population. Whether the increased risk observed was due to Parkinson's disease or other factors, such as drugs used to treat Parkinson's disease, is unclear.

For the reasons stated above, patients and providers are advised to monitor for melanomas frequently and on a regular basis when using SINEMET for any indication. Ideally, periodic skin examinations should be performed by appropriately qualified individuals (e.g., dermatologists).

Information for Patients

The patient should be informed that SINEMET is an immediate-release formulation of carbidopa levodopa that is designed to begin release of ingredients within 30 minutes. It is important that SINEMET be taken at regular intervals according to the schedule outlined by the physician. The patient should be cautioned not to change the prescribed dosage regimen and not to add any additional antiparkinson medications, including other carbidopa levodopa preparations, without first consulting the physician.

Patients should be advised that sometimes a 'wearing-off' effect may occur at the end of the dosing interval. The physician should be notified if such response poses a problem to lifestyle.

Patients should be advised that occasionally, dark color (red, brown, or black) may appear in saliva, urine, or sweat after ingestion of SINEMET. Although the color appears to be clinically insignificant, garments may become discolored.

The patient should be advised that a change in diet to foods that are high in protein may delay the absorption of levodopa and may reduce the amount taken up in the circulation. Excessive acidity also delays stomach emptying, thus delaying the absorption of levodopa. Iron salts (such as in multivitamin tablets) may also reduce the amount of levodopa available to the body. The above factors may reduce the clinical effectiveness of the levodopa or carbidopa levodopa therapy.

Patients should be alerted to the possibility of sudden onset of sleep during daily activities, in some cases without awareness or warning signs, when they are taking dopaminergic agents, including levodopa. Patients should be advised to exercise caution while driving or operating machinery and that if they have experienced somnolence and/or sudden sleep onset, they must refrain from these activities. (See WARNINGS, Falling Asleep During Activities of Daily Living and Somnolence.)

There have been reports of patients experiencing intense urges to gamble, increased sexual urges, and other intense urges, and the inability to control these urges while taking one or more of the medications that increase central dopaminergic tone and that are generally used for the treatment of Parkinson's disease, including SINEMET. Although it is not proven that the medications caused these events, these urges were reported to have stopped in some cases when the dose was reduced or the medication was stopped. Prescribers should ask patients about the development of new or increased gambling urges, sexual urges or other urges while being treated with SINEMET. Patients should inform their physician if they experience new or increased gambling urges, increased sexual urges, or other intense urges while taking SINEMET. Physicians should consider dose reduction or stopping the medication if a patient develops such urges while taking SINEMET (See PRECAUTIONS, Impulse Control / Compulsive Behaviors).

Laboratory Tests

Abnormalities in laboratory tests may include elevations of liver function tests such as alkaline phosphatase, SGOT (AST), SGPT (ALT), lactic dehydrogenase (LDH), and bilirubin. Abnormalities in blood urea nitrogen (BUN) and positive Coombs test have also been reported. Commonly, levels of blood urea nitrogen, creatinine, and uric acid are lower during administration of SINEMET than with levodopa.

SINEMET may cause a false-positive reaction for urinary ketone bodies when a test tape is used for determination of ketonuria. This reaction will not be altered by boiling the urine specimen. False-negative tests may result with the use of glucose-oxidase methods of testing for glucosuria.

Cases of falsely diagnosed pheochromocytoma in patients on carbidopa levodopa therapy have been reported very rarely. Caution should be exercised when interpreting the plasma and urine levels of catecholamines and their metabolites in patients on levodopa or carbidopa levodopa therapy.

Drug Interactions

Caution should be exercised when the following drugs are administered concomitantly with SINEMET.

Symptomatic postural hypotension occurred when SINEMET was added to the treatment of a patient receiving antihypertensive drugs. Therefore, when therapy with SINEMET is started, dosage adjustment of the antihypertensive drug may be required.

For patients receiving MAO inhibitors (Type A or B), see CONTRAINDICATIONS. Concomitant therapy with selegiline and carbidopa levodopa may be associated with severe orthostatic hypotension not attributable to carbidopa levodopa alone (see CONTRAINDICATIONS).

There have been rare reports of adverse reactions, including hypertension and dyskinesia, resulting from the concomitant use of tricyclic antidepressants and SINEMET.

Dopamine D_2 receptor antagonists (e.g., phenothiazines, butyrophenones, risperidone) and isoniazid may reduce the therapeutic effects of levodopa. In addition, the beneficial effects of levodopa in Parkinson's disease have been reported to be reversed by phenytoin and papaverine. Patients taking these drugs with SINEMET should be carefully observed for loss of therapeutic response.

Use of SINEMET with dopamine-depleting agents (e.g., reserpine and tetrabenazine) or other drugs known to deplete monoamine stores is not recommended.

SINEMET and iron salts or multivitamins containing iron salts should be coadministered with caution. Iron salts can form chelates with levodopa and carbidopa and consequently reduce the bioavailability of carbidopa and levodopa.

Although metoclopramide may increase the bioavailability of levodopa by increasing gastric emptying, metoclopramide may also adversely affect disease control by its dopamine receptor antagonistic properties.

Carcinogenesis, Mutagenesis, Impairment of Fertility

In a two-year bioassay of SINEMET, no evidence of carcinogenicity was found in rats receiving doses of approximately two times the maximum daily human dose of carbidopa and four times the maximum daily human dose of levodopa.

In reproduction studies with SINEMET, no effects on fertility were found in rats receiving doses of approximately two times the maximum daily human dose of carbidopa and four times the maximum daily human dose of levodopa.

Pregnancy

Pregnancy Category C. No teratogenic effects were observed in a study in mice receiving up to 20 times the maximum recommended human dose of SINEMET. There was a decrease in the number of live pups delivered by rats receiving approximately two times the maximum recommended human dose of carbidopa and approximately five times the maximum recommended human dose of levodopa during organogenesis. SINEMET caused both visceral and skeletal malformations in rabbits at all doses and ratios of carbidopa/levodopa tested, which ranged from 10 times/5 times the maximum recommended human dose of carbidopa/levodopa to 20 times/10 times the maximum recommended human dose of carbidopa/levodopa.

There are no adequate or well-controlled studies in pregnant women. It has been reported from individual cases that levodopa crosses the human placental barrier, enters the fetus, and is metabolized. Carbidopa concentrations in fetal tissue appeared to be minimal. Use of SINEMET in women of childbearing potential requires that the anticipated benefits of the drug be weighed against possible hazards to mother and child.

Nursing Mothers

Levodopa has been detected in human milk. Caution should be exercised when SINEMET is administered to a nursing woman.

Pediatric Use

Safety and effectiveness in pediatric patients have not been established. Use of the drug in patients below the age of 18 is not recommended.

Geriatric Use

In the clinical efficacy trials for SINEMET, almost half of the patients were older than 65, but few were older than 75. No overall meaningful differences in safety or effectiveness were observed between these subjects and younger subjects, but greater sensitivity of some older individuals to adverse drug reactions such as hallucinations cannot be ruled out. There is no specific dosing recommendation based upon clinical pharmacology data as SINEMET is titrated as tolerated for clinical effect.

ADVERSE REACTIONS

The most common adverse reactions reported with SINEMET have included dyskinesias, such as choreiform, dystonic, and other involuntary movements, and nausea.

The following other adverse reactions have been reported with SINEMET:

Body as a Whole

Chest pain, asthenia.

Cardiovascular

Cardiac irregularities, hypotension, orthostatic effects including orthostatic hypotension, hypertension, syncope, phlebitis, palpitation.

Gastrointestinal

Dark saliva, gastrointestinal bleeding, development of duodenal ulcer, anorexia, vomiting, diarrhea, constipation, dyspepsia, dry mouth, taste alterations.

Hematologic

Agranulocytosis, hemolytic and non-hemolytic anemia, thrombocytopenia, leukopenia.

Hypersensitivity

Angioedema, urticaria, pruritus, Henoch-Schönlein purpura, bullous lesions (including pemphigus-like reactions).

Musculoskeletal

Back pain, shoulder pain, muscle cramps.

Nervous System/Psychiatric

Psychotic episodes including delusions, hallucinations, and paranoid ideation, bradykinetic episodes ("on-off" phenomenon), confusion, agitation, dizziness, somnolence, dream abnormalities including nightmares, insomnia, paresthesia, headache, depression with or without development of suicidal tendencies, dementia, pathological gambling, increased libido including hypersexuality, impulse control symptoms. Convulsions also have occurred; however, a causal relationship with SINEMET has not been established.

Respiratory

Dyspnea, upper respiratory infection.

Skin

Rash, increased sweating, alopecia, dark sweat.

Urogenital

Urinary tract infection, urinary frequency, dark urine.

Laboratory Tests

Decreased hemoglobin and hematocrit; abnormalities in alkaline phosphatase, SGOT (AST), SGPT (ALT), LDH, bilirubin, BUN, Coombs test; elevated serum glucose; white blood cells, bacteria, and blood in the urine.

Other adverse reactions that have been reported with levodopa alone and with various carbidopa levodopa formulations, and may occur with SINEMET are:

Body as a Whole

Abdominal pain and distress, fatigue.

Cardiovascular
Myocardial infarction.
Gastrointestinal
Gastrointestinal pain, dysphagia, sialorrhea, flatulence, bruxism, burning sensation of the tongue, heartburn, hiccups.
Metabolic
Edema, weight gain, weight loss.
Musculoskeletal
Leg pain.
Nervous System/Psychiatric
Ataxia, extrapyramidal disorder, falling, anxiety, gait abnormalities, nervousness, decreased mental acuity, memory impairment, disorientation, euphoria, blepharospasm (which may be taken as an early sign of excess dosage; consideration of dosage reduction may be made at this time), trismus, increased tremor, numbness, muscle twitching, activation of latent Horner's syndrome, peripheral neuropathy.
Respiratory
Pharyngeal pain, cough.
Skin
Malignant melanoma (see also CONTRAINDICATIONS), flushing.
Special Senses
Oculogyric crises, diplopia, blurred vision, dilated pupils.
Urogenital
Urinary retention, urinary incontinence, priapism.
Miscellaneous
Bizarre breathing patterns, faintness, hoarseness, malaise, hot flashes, sense of stimulation.
Laboratory Tests
Decreased white blood cell count and serum potassium; increased serum creatinine and uric acid; protein and glucose in urine.

OVERDOSAGE

Management of acute overdosage with SINEMET is the same as management of acute overdosage with levodopa. Pyridoxine is not effective in reversing the actions of SINEMET.

General supportive measures should be employed, along with immediate gastric lavage. Intravenous fluids should be administered judiciously and an adequate airway maintained. Electrocardiographic monitoring should be instituted and the patient carefully observed for the development of arrhythmias; if required, appropriate antiarrhythmic therapy should be given. The possibility that the patient may have taken other drugs as well as SINEMET should be taken into consideration. To date, no experience has been reported with dialysis; hence, its value in overdosage is not known.

Based on studies in which high doses of levodopa and/or carbidopa were administered, a significant proportion of rats and mice given single oral doses of levodopa of approximately 1500-2000 mg/kg are expected to die. A significant proportion of infant rats of both sexes are expected to die at a dose of 800 mg/kg. A significant proportion of rats are expected to die after treatment with similar doses of carbidopa. The addition of carbidopa in a 1:10 ratio with levodopa increases the dose at which a significant proportion of mice are expected to die to 3360 mg/kg.

DOSAGE AND ADMINISTRATION

The optimum daily dosage of SINEMET must be determined by careful titration in each patient. SINEMET tablets are available in a 1:4 ratio of carbidopa to levodopa (SINEMET 25-100) as well as 1:10 ratio (SINEMET 25-250 and SINEMET 10-100). Tablets of the two ratios may be given separately or combined as needed to provide the optimum dosage.

Studies show that peripheral dopa decarboxylase is saturated by carbidopa at approximately 70 to 100 mg a day. Patients receiving less than this amount of carbidopa are more likely to experience nausea and vomiting.

Usual Initial Dosage

Dosage is best initiated with one tablet of SINEMET 25-100 three times a day. This dosage schedule provides 75 mg of carbidopa per day. Dosage may be increased by one tablet every day or every other day, as necessary, until a dosage of eight tablets of SINEMET 25-100 a day is reached.

If SINEMET 10-100 is used, dosage may be initiated with one tablet three or four times a day. However, this will not provide an adequate amount of carbidopa for many patients. Dosage may be increased by one tablet every day or every other day until a total of eight tablets (2 tablets q.i.d.) is reached.

How to Transfer Patients from Levodopa

Levodopa must be discontinued at least twelve hours before starting SINEMET. A daily dosage of SINEMET should be chosen that will provide approximately 25% of the previous levodopa dosage. Patients who are taking less than 1500 mg of levodopa a day should be started on one tablet of SINEMET 25-100 three or four times a day. The suggested starting dosage for most patients taking more than 1500 mg of levodopa is one tablet of SINEMET 25-250 three or four times a day.

Maintenance

Therapy should be individualized and adjusted according to the desired therapeutic response. At least 70 to 100 mg of carbidopa per day should be provided. When a greater proportion of carbidopa is required, one tablet of SINEMET 25-100 may be substituted for each tablet of SINEMET 10-100. When more levodopa is required, SINEMET 25-250 should be substituted for SINEMET 25-100 or SINEMET 10-100. If necessary, the dosage of carbidopa levodopa 25-250 may be increased by one-half or one tablet every day or every other day to a maximum of eight tablets a day. Experience with total daily dosages of carbidopa greater than 200 mg is limited.

Because both therapeutic and adverse responses occur more rapidly with SINEMET than with levodopa alone, patients should be monitored closely during the dose adjustment period. Specifically, involuntary movements will occur more rapidly with SINEMET than with levodopa. The occurrence of involuntary movements may require dosage reduction. Blepharospasm may be a useful early sign of excess dosage in some patients.

Addition of Other Antiparkinsonian Medications

Standard drugs for Parkinson's disease, other than levodopa without a decarboxylase inhibitor, may be used concomitantly while SINEMET is being administered, although dosage adjustments may be required.

Interruption of Therapy

Sporadic cases of hyperpyrexia and confusion have been associated with dose reductions and withdrawal of SINEMET. Patients should be observed carefully if abrupt reduction or discontinuation of SINEMET is required, especially if the patient is receiving neuroleptics. (See WARNINGS.)

If general anesthesia is required, SINEMET may be continued as long as the patient is permitted to take fluids and medication by mouth. If therapy is interrupted temporarily, the patient should be observed for symptoms resembling NMS, and the usual daily dosage may be administered as soon as the patient is able to take oral medication.

HOW SUPPLIED

No. 3916A — SINEMET 25-100 Tablets are yellow, round, uncoated tablets, that are coded "650" on one side and plain on the other. They are supplied as follows:

NDC 0006-3916-68 bottles of 100.

No. 3915 — SINEMET 10-100 Tablets are light dapple-blue, round, uncoated tablets, that are coded "647" on one side and plain on the other. They are supplied as follows:

NDC 0006-3915-68 bottles of 100.

No. 3917 — SINEMET 25-250 Tablets are light dapple-blue, round, uncoated tablets, that are coded "654" on one side and plain on the other. They are supplied as follows:

NDC 0006-3917-68 bottles of 100.

Storage and Handling

Store at 25°C (77°F), excursions permitted to 15-30°C (59-86°F) [see USP Controlled Room Temperature]. Store in a tightly closed container, protected from light and moisture.

Dispense in a tightly closed, light-resistant container.

Manufactured for: Merck Sharp & Dohme Corp., a subsidiary of
MERCK & CO., INC., Whitehouse Station, NJ 08889, USA

Manufactured by:
Mylan Pharmaceuticals, Inc.
Morgantown, WV 26505, USA

Copyright © 1996-2014 Merck Sharp & Dohme Corp., a subsidiary of **Merck & Co., Inc.**
All rights reserved.

Revised: 07/2014

uspi-mk0295b-t-1407r003

Rx Only

APPENDIX B. UNITED KINGDOM PARKINSON'S DISEASE SOCIETY BRAIN BANK DIAGNOSTIC CRITERIA FOR PARKINSON'S DISEASE

**Step 1: Diagnosis of Parkinsonism**
Bradykinesia **and** at least one of the following:<br>• Muscular rigidity<br>• 4–6 Hz resting tremor<br>• postural instability not caused by primary visual, vestibular, cerebellar or Proprioceptive dysfunction
**Step 2: Features tending to exclude Parkinson's disease as the cause of Parkinsonism**
• History of repeated strokes with stepwise progression of parkinsonian features<br>• History of repeated head injury<br>• History of definite encephalitis<br>• Neuroleptic treatment at onset of symptoms<br>• >1 affected relatives<br>• Sustained remission<br>• Strictly unilateral features after 3 years<br>• Supranuclear gaze palsy<br>• Cerebellar signs<br>• Early severe autonomic involvement<br>• Early severe dementia with disturbances of memory, language and praxis<br>• Babinski's sign<br>• Presence of a cerebral tumour or communicating hydrocephalus on computed tomography scan<br>• Negative response to large doses of levodopa (if malabsorption excluded)<br>• MPTP exposure
**Step 3: Features that support a diagnosis of Parkinson's disease (three or more required for diagnosis of definite Parkinson's disease)**
• Unilateral onset<br>• Rest tremor present<br>• Progressive disorder<br>• Persistent asymmetry affecting the side of onset most<br>• Excellent (70–100%) response to levodopa<br>• Severe levodopa-induced chorea<br>• Levodopa response for ≥5 years<br>• Clinical course of ≥10 years

APPENDIX C. MONTREAL COGNITIVE ASSESSMENT (MOCA)

Montreal Cognitive Assessment
(MoCA)

Administration and Scoring Instructions

The Montreal Cognitive Assessment (MoCA) was designed as a rapid screening instrument for mild cognitive dysfunction. It assesses different cognitive domains: attention and concentration, executive functions, memory, language, visuoconstructional skills, conceptual thinking, calculations, and orientation. Time to administer the MoCA is approximately 10 minutes. The total possible score is 30 points; a score of 26 or above is considered normal.

1. Alternating Trail Making:

Administration: The examiner instructs the subject: *"Please draw a line, going from a number to a letter in ascending order. Begin here* [point to (1)] *and draw a line from 1 then to A then to 2 and so on. End here* [point to (E)]."

Scoring: Allocate one point if the subject successfully draws the following pattern:
1 –A- 2- B- 3- C- 4- D- 5- E, without drawing any lines that cross. Any error that is not immediately self-corrected earns a score of 0.

2. Visuoconstructional Skills (Cube):

Administration: The examiner gives the following instructions, pointing to the **cube**: "*Copy this drawing as accurately as you can, in the space below".*

Scoring: One point is allocated for a correctly executed drawing.
• Drawing must be three-dimensional
• All lines are drawn
• No line is added
• Lines are relatively parallel and their length is similar (rectangular prisms are accepted)

A point is not assigned if any of the above-criteria are not met.

3. Visuoconstructional Skills (Clock):

Administration: Indicate the right third of the space and give the following instructions: *"Draw a clock. Put in all the numbers and set the time to 10 past 11".*

Scoring: One point is allocated for each of the following three criteria:
• Contour (1 pt.): the clock face must be a circle with only minor distortion acceptable (e.g., slight imperfection on closing the circle);
• Numbers (1 pt.): all clock numbers must be present with no additional numbers; numbers must be in the correct order and placed in the approximate quadrants on the clock face; Roman numerals are acceptable; numbers can be placed outside the circle contour;
• Hands (1 pt.): there must be two hands jointly indicating the correct time; the hour hand must be clearly shorter than the minute hand; hands must be centred within the clock face with their junction close to the clock centre.

A point is not assigned for a given element if any of the above-criteria are not met.

© Z. Nasreddine MD

4. Naming:

Administration: Beginning on the left, point to each figure and say: *"Tell me the name of this animal".*

Scoring: One point each is given for the following responses: (1) lion (2) rhinoceros or rhino (3) camel or dromedary.

5. Memory:

Administration: The examiner reads a list of 5 words at a rate of one per second, giving the following instructions: *"This is a memory test. I am going to read a list of words that you will have to remember now and later on. Listen carefully. When I am through, tell me as many words as you can remember. It doesn't matter in what order you say them".* Mark a check in the allocated space for each word the subject produces on this first trial. When the subject indicates that (s)he has finished (has recalled all words), or can recall no more words, read the list a second time with the following instructions: *"I am going to read the same list for a second time. Try to remember and tell me as many words as you can, including words you said the first time."* Put a check in the allocated space for each word the subject recalls after the second trial.
At the end of the second trial, inform the subject that (s)he will be asked to recall these words again by saying, *"I will ask you to recall those words again at the end of the test."*

Scoring: No points are given for Trials One and Two.

6. Attention:

Forward Digit Span: Administration: Give the following instruction: "*I am going to say some numbers and when I am through, repeat them to me exactly as I said them*". Read the five number sequence at a rate of one digit per second.

Backward Digit Span: Administration: Give the following instruction: "*Now I am going to say some more numbers, but when I am through you must repeat them to me in the backwards order.*" Read the three number sequence at a rate of one digit per second.

Scoring: Allocate one point for each sequence correctly repeated, (*N.B.*: the correct response for the backwards trial is 2-4-7).

Vigilance: Administration: The examiner reads the list of letters at a rate of one per second, after giving the following instruction: "*I am going to read a sequence of letters. Every time I say the letter A, tap your hand once. If I say a different letter, do not tap your hand*".

Scoring: Give one point if there is zero to one errors (an error is a tap on a wrong letter or a failure to tap on letter A).

© Z. Nasreddine MD
www.mocatest.org

Serial 7s: Administration: The examiner gives the following instruction: *"Now, I will ask you to count by subtracting seven from 100, and then, keep subtracting seven from your answer until I tell you to stop."* Give this instruction twice if necessary.

Scoring: This item is scored out of 3 points. Give no (0) points for no correct subtractions, 1 point for one correction subtraction, 2 points for two-to-three correct subtractions, and 3 points if the participant successfully makes four or five correct subtractions. Count each correct subtraction of 7 beginning at 100. Each subtraction is evaluated independently; that is, if the participant responds with an incorrect number but continues to correctly subtract 7 from it, give a point for each correct subtraction. For example, a participant may respond "92 – 85 – 78 – 71 – 64" where the "92" is incorrect, but all subsequent numbers are subtracted correctly. This is one error and the item would be given a score of 3.

7. **Sentence repetition:**

Administration: The examiner gives the following instructions: *"I am going to read you a sentence. Repeat it after me, exactly as I say it* [pause]: ***I only know that John is the one to help today."*** Following the response, say: *"Now I am going to read you another sentence. Repeat it after me, exactly as I say it* [pause]: ***The cat always hid under the couch when dogs were in the room."***

Scoring: Allocate 1 point for each sentence correctly repeated. Repetition must be exact. Be alert for errors that are omissions (e.g., omitting "only", "always") and substitutions/additions (e.g., "John is the one who helped today;" substituting "hides" for "hid", altering plurals, etc.).

8. **Verbal fluency:**

Administration: The examiner gives the following instruction: *"Tell me as many words as you can think of that begin with a certain letter of the alphabet that I will tell you in a moment. You can say any kind of word you want, except for proper nouns (like Bob or Boston), numbers, or words that begin with the same sound but have a different suffix, for example, love, lover, loving. I will tell you to stop after one minute. Are you ready?* [Pause] *Now, tell me as many words as you can think of that begin with the letter F.* [time for 60 sec]. *Stop."*

Scoring: Allocate one point if the subject generates 11 words or more in 60 sec. Record the subject's response in the bottom or side margins.

9. **Abstraction:**

Administration: The examiner asks the subject to explain what each pair of words has in common, starting with the example: *"Tell me how an orange and a banana are alike"*. If the subject answers in a concrete manner, then say only one additional time: *"Tell me another way in which those items are alike"*. If the subject does not give the appropriate response *(fruit)*, say, *"Yes, and they are also both fruit."* Do not give any additional instructions or clarification. After the practice trial, say: *"Now, tell me how a train and a bicycle are alike"*. Following the response, administer the second trial, saying: *"Now tell me how a ruler and a watch are alike"*. Do not give any additional instructions or prompts.

Scoring: Only the last two item pairs are scored. Give 1 point to each item pair correctly answered. The following responses are acceptable:
Train-bicycle = means of transportation, means of travelling, you take trips in both;
Ruler-watch = measuring instruments, used to measure.
The following responses are **not** acceptable: Train-bicycle = they have wheels; Ruler-watch = they have numbers.

10. Delayed recall:

Administration: The examiner gives the following instruction: *"I read some words to you earlier, which I asked you to remember. Tell me as many of those words as you can remember."* Make a check mark (√) for each of the words correctly recalled spontaneously without any cues, in the allocated space.

Scoring: **Allocate 1 point for each word recalled freely without any cues.**

Optional:
Following the delayed free recall trial, prompt the subject with the semantic category cue provided below for any word not recalled. Make a check mark (√) in the allocated space if the subject remembered the word with the help of a category or multiple-choice cue. Prompt all non-recalled words in this manner. If the subject does not recall the word after the category cue, give him/her a multiple choice trial, using the following example instruction, *"Which of the following words do you think it was, NOSE, FACE, or HAND?"*
Use the following category and/or multiple-choice cues for each word, when appropriate:

FACE:	category cue: part of the body	multiple choice: nose, face, hand
VELVET:	category cue: type of fabric	multiple choice: denim, cotton, velvet
CHURCH:	category cue: type of building	multiple choice: church, school, hospital
DAISY:	category cue: type of flower	multiple choice: rose, daisy, tulip
RED:	category cue: a colour	multiple choice: red, blue, green

Scoring: **No points are allocated for words recalled with a cue.** A cue is used for clinical information purposes only and can give the test interpreter additional information about the type of memory disorder. For memory deficits due to retrieval failures, performance can be improved with a cue. For memory deficits due to encoding failures, performance does not improve with a cue.

11. Orientation:

Administration: The examiner gives the following instructions: "Tell me the date today". If the subject does not give a complete answer, then prompt accordingly by saying: *"Tell me the [year, month, exact date, and day of the week]."* Then say: *"Now, tell me the name of this place, and which city it is in."*

Scoring: Give one point for each item correctly answered. The subject must tell the exact date and the exact place (name of hospital, clinic, office). No points are allocated if subject makes an error of one day for the day and date.

TOTAL SCORE: Sum all subscores listed on the right-hand side. Add one point for an individual who has 12 years or fewer of formal education, for a possible maximum of 30 points. A final total score of 26 and above is considered normal.

© Z. Nasreddine MD *www.mocatest.org*

MONTREAL COGNITIVE ASSESSMENT (MOCA)
Version 7.1 Original Version

NAME :
Education : Date of birth :
Sex : DATE :

VISUOSPATIAL / EXECUTIVE

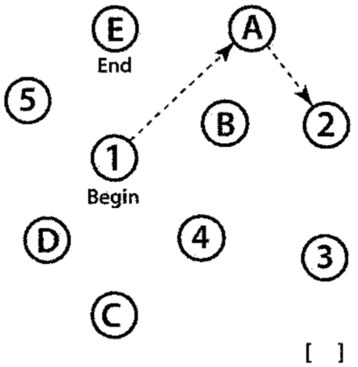

[ ]

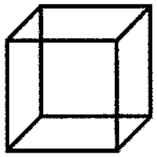

Copy cube

[ ]

Draw CLOCK (Ten past eleven)
(3 points)

[ ] Contour [ ] Numbers [ ] Hands

POINTS ___/5

NAMING

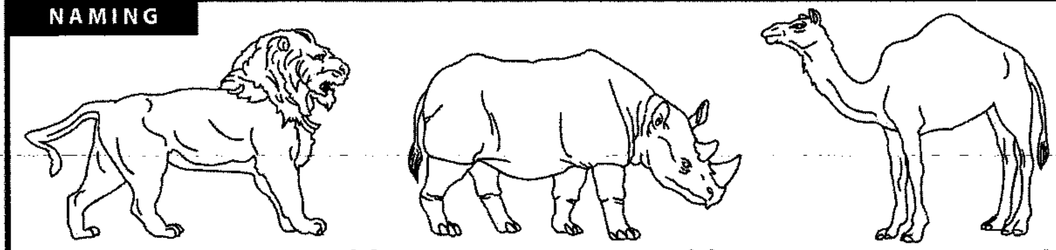

[ ] [ ] [ ] ___/3

MEMORY

Read list of words, subject must repeat them. Do 2 trials, even if 1st trial is successful. Do a recall after 5 minutes.

	FACE	VELVET	CHURCH	DAISY	RED
1st trial					
2nd trial					

No points

ATTENTION

Read list of digits (1 digit/ sec.). Subject has to repeat them in the forward order [ ] 2 1 8 5 4
Subject has to repeat them in the backward order [ ] 7 4 2 ___/2

Read list of letters. The subject must tap with his hand at each letter A. No points if ≥ 2 errors
[ ] F B A C M N A A J K L B A F A K D E A A A J A M O F A A B ___/1

Serial 7 subtraction starting at 100 [ ] 93 [ ] 86 [ ] 79 [ ] 72 [ ] 65
4 or 5 correct subtractions: **3 pts**, 2 or 3 correct: **2 pts**, 1 correct: **1 pt**, 0 correct: **0 pt** ___/3

LANGUAGE

Repeat : I only know that John is the one to help today. [ ]
The cat always hid under the couch when dogs were in the room. [ ] ___/2

Fluency / Name maximum number of words in one minute that begin with the letter F [ ] _____ (N ≥ 11 words) ___/1

ABSTRACTION

Similarity between e.g. banana - orange = fruit [ ] train – bicycle [ ] watch - ruler ___/2

DELAYED RECALL

Has to recall words WITH NO CUE	FACE [ ]	VELVET [ ]	CHURCH [ ]	DAISY [ ]	RED [ ]	Points for UNCUED recall only
**Optional** Category cue						
Multiple choice cue						

___/5

ORIENTATION

[ ] Date [ ] Month [ ] Year [ ] Day [ ] Place [ ] City ___/6

© Z.Nasreddine MD www.mocatest.org Normal ≥26 / 30 TOTAL ___/30
Add 1 point if ≤ 12 yr edu

Administered by:________________________________

APPENDIX D. MOVEMENT DISORDERS SOCIETY VERSION OF UNIFIED PARKINSON'S DISEASE RATING SCALE (MDS-UPDRS)

MDS-UPDRS Permissions

Permission is required to use the MDS-developed Rating Scales (with the exception of personal/individual use). Reproduction, translation, modification, sale, or distribution of any portion of the MDS Rating Scales is strictly prohibited. MDS Rating Scales may not be incorporated into clinical trials, training or certification programs or materials, software programs, or otherwise except through use of the Permissions Request Form and payment of applicable fees.

Continue to p. 2 to view the MDS-UPDRS

MDS-UPDRS

The *Movement* Disorder Society (MDS)-sponsored new version of the UPDRS is founded on the critique that was formulated by the Task Force for Rating Scales in Parkinson's disease (*Mov Disord* 2003;18:738-750). Thereafter, the MDS recruited a Chairperson to organize a program to provide the Movement Disorder community with a new version of the UPDRS that would maintain the overall format of the original UPDRS, but address issues identified in the critique as weaknesses and ambiguities. The Chairperson identified subcommittees with chairs and members. Each part was written by the appropriate subcommittee members and then reviewed and ratified by the entire group. These members are listed below.

The MDS-UPDRS has four parts: Part I (non-motor experiences of daily living), Part II (motor experiences of daily living, Part III (motor examination) and Part IV (motor complications). Part I has two components: IA concerns a number of behaviors that are assessed by the investigator with all pertinent information from patients and caregivers, and IB is completed by the patient with or without the aid of the caregiver, but independently of the investigator. These sections can, however, be reviewed by the rater to ensure that all questions are answered clearly and the rater can help explain any perceived ambiguities. Part II is designed to be a self-administered questionnaire like Part IB, but can be reviewed by the investigator to ensure completeness and clarity. Of note, the official versions of Part IA, Part IB and Part II of the MDS-UPDRS do not have separate on or off ratings. However, for individual programs or protocols the same questions can be used separately for on and off. Part III has instructions for the rater to give or demonstrate to the patient; it is completed by the rater. Part IV has instructions for the rater and also instructions to be read to the patient. This part integrates patient-derived information with the rater's clinical observations and judgments and is completed by the rater.

The authors of this new version are:

Chairperson: Christopher G. Goetz
Part I: Werner Poewe (chair), Bruno Dubois, Anette Schrag
Part II: Matthew B. Stern (chair), Anthony E. Lang, Peter A. LeWitt
Part III: Stanley Fahn (chair), Joseph Jankovic, C. Warren Olanow
Part IV: Pablo Martinez-Martin (chair), Andrew Lees, Olivier Rascol, Bob van Hilten
Development Standards: Glenn T. Stebbins (chair), Robert Holloway, David Nyenhuis
Appendices: Cristina Sampaio (chair), Richard Dodel, Jaime Kulisevsky
Statistical Testing: Barbara Tilley (chair), Sue Leurgans, Jean Teresi,
Consultant: Stephanie Shaftman, Nancy LaPelle

Contact person: Christopher G. Goetz, MD
Rush University Medical Center
1725 W. Harrison Street, Suite 755
Chicago, IL USA 60612

Telephone 312-942-8016
Email: cgoetz@rush.edu

July 1, 2008

Copyright © 2008 Movement Disorder Society. All rights reserved.
This chart may not be copied, distributed or otherwise used in whole or in part without prior written consent of the Movement Disorder Society.

Part I: Non-Motor Aspects of Experiences of Daily Living (nM-EDL)

Overview: This portion of the scale assesses the non-motor impact of Parkinson's disease (PD) on patients' experiences of daily living. There are 13 questions. Part 1A is administered by the rater (six questions) and focuses on complex behaviors. Part 1B is a component of the self-administered Patient Questionnaire that covers seven questions on non-motor experiences of daily living.

Part 1A:
In administering Part IA, the examiner should use the following guidelines:

1. Mark at the top of the form the primary data source as patient, caregiver, or patient and caregiver in equal proportion.
2. The response to each item should refer to a period encompassing the prior week including the day on which the information is collected.
3. All items must have an integer rating (no half points, no missing scores). In the event that an item does not apply or cannot be rated (e.g., amputee who cannot walk), the item is marked UR for Unable to Rate.
4. The answers should reflect the usual level of function and words such as “usually”, “generally”, “most of the time” can be used with patients.
5. Each question has a text for you to read (Instructions to patients/caregiver). After that statement, you can elaborate and probe based on the target symptoms outlined in the Instructions to examiner. You should NOT READ the RATING OPTIONS to the patient/caregiver, because these are written in medical terminology. From the interview and probing, you will use your medical judgment to arrive at the best response.
6. Patients may have co-morbidities and other medical conditions that can affect their function. You and the patient must rate the problem as it exists and do not attempt to separate elements due to Parkinson’s disease from other conditions.

EXAMPLE OF NAVIGATING THROUGH THE RESPONSE OPTIONS FOR PART 1A

Suggested strategies for obtaining the most accurate answer:
After reading the instructions to the patient, you will need to probe the entire domain under discussion to determine Normal vs. problematic: If your questions do not identify any problem in this domain, record 0 and move on to the next question.

If your questions identify a problem in this domain, you should work next with a reference anchor at the mid-range (option 2 or Mild) to find out if the patient functions at this level, better or worse. You will not be reading the choices of responses to the patient as the responses use clinical terminology. You will be asking enough probing questions to determine the response that should be coded.

Work up and down the options with the patient to identify the most accurate response, giving a final check by excluding the options above and below the selected response.

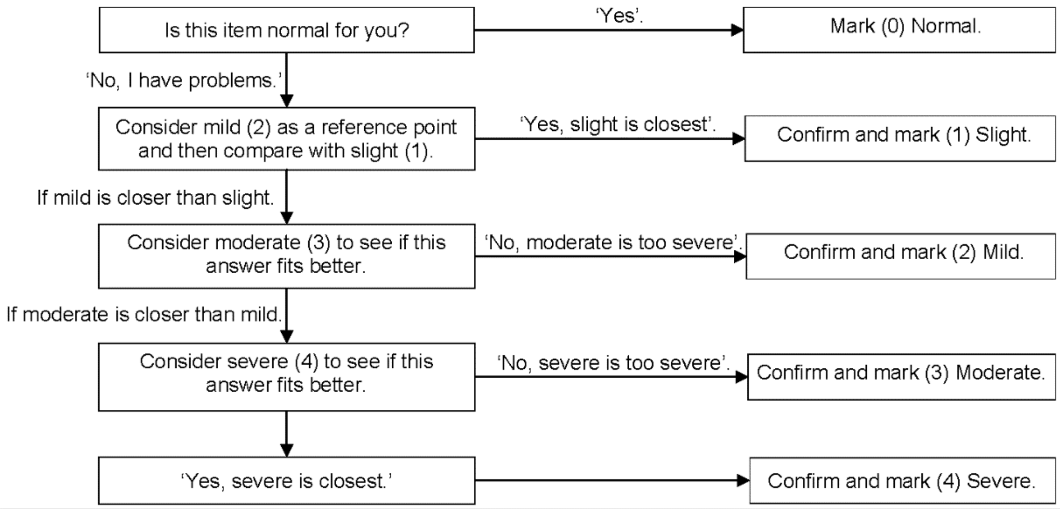

July 1, 2008 *Copyright © 2008 Movement Disorder Society. All rights reserved.* Page 2
This chart may not be copied, distributed or otherwise used in whole or in part without prior written consent of the Movement Disorder Society.

___ ___ ___ ___ ___ ___ ___ ___ Patient Name or Subject ID	___ ___ ___ Site ID	___ ___ - ___ ___ - ___ ___ ___ ___ (mm-dd-yyyy) Assessment Date	___ ___ ___ Investigator's Initials

MDS UPDRS

Part I: Non-Motor Aspects of Experiences of Daily Living (nM-EDL)

Part 1A: Complex behaviors: [completed by rater]

Primary source of information:

☐ Patient ☐ Caregiver ☐ Patient and Caregiver in Equal Proportion

To be read to the patient: I am going to ask you six questions about behaviors that you may or may not experience. Some questions concern common problems and some concern uncommon ones. If you have a problem in one of the areas, please choose the best response that describes how you have felt MOST OF THE TIME during the PAST WEEK. If you are not bothered by a problem, you can simply respond NO. I am trying to be thorough, so I may ask questions that have nothing to do with you.

1.1 COGNITIVE IMPAIRMENT **SCORE**

Instructions to examiner: Consider all types of altered level of cognitive function including cognitive slowing, impaired reasoning, memory loss, deficits in attention and orientation. Rate their impact on activities of daily living as perceived by the patient and/or caregiver.

Instructions to patients [and caregiver]: Over the past week have you had problems remembering things, following conversations, paying attention, thinking clearly, or finding your way around the house or in town? [If yes, examiner asks patient or caregiver to elaborate and probes for information]

0: Normal: No cognitive impairment.

1: Slight: Impairment appreciated by patient or caregiver with no concrete interference with the patient's ability to carry out normal activities and social interactions.

2: Mild: Clinically evident cognitive dysfunction, but only minimal interference with the patient's ability to carry out normal activities and social interactions.

3: Moderate: Cognitive deficits interfere with but do not preclude the patient's ability to carry out normal activities and social interactions.

4: Severe: Cognitive dysfunction precludes the patient's ability to carry out normal activities and social interactions.

☐

Copyright © 2008 Movement Disorder Society. All rights reserved.

This chart may not be copied, distributed or otherwise used in whole or in part without prior written consent of the Movement Disorder Society.

1.2 HALLUCINATIONS AND PSYCHOSIS	SCORE
Instructions to examiner: Consider both illusions (misinterpretations of real stimuli) and hallucinations (spontaneous false sensations). Consider all major sensory domains (visual, auditory, tactile, olfactory and gustatory). Determine presence of unformed (for example sense of presence or fleeting false impressions) as well as formed (fully developed and detailed) sensations. Rate the patients insight into hallucinations and identify delusions and psychotic thinking.<br><br>*Instructions to patients [and caregiver]: Over the past week have you seen, heard, smelled or felt things that were not really there?* [If yes, examiner asks patient or caregiver to elaborate and probes for information]<br><br>0: Normal: No hallucinations or psychotic behaviour.<br>1: Slight: Illusions or non-formed hallucinations, but patient recognizes them without loss of insight.<br>2: Mild: Formed hallucinations independent of environmental stimuli. No loss of insight.<br>3: Moderate: Formed hallucinations with loss of insight.<br>4: Severe: Patient has delusions or paranoia.	☐
**1.3 DEPRESSED MOOD**	
Instructions to examiner: Consider low mood, sadness, hopelessness, feelings of emptiness or loss of enjoyment. Determine their presence and duration over the past week and rate their interference with the patient's ability to carry out daily routines and engage in social interactions.<br><br>*Instruction to the patient (and caregiver): Over the past week have you felt low, sad, hopeless or unable to enjoy things? If yes, was this feeling for longer than one day at a time? Did it make it difficult for you carry out your usual activities or to be with people?* If yes, examiner asks patient or caregiver to elaborate and probes for information]<br><br>0: Normal: No depressed mood.<br>1: Slight: Episodes of depressed mood that are not sustained for more than one day at a time. No interference with patient's ability to carry out normal activities and social interactions.<br>2: Mild: Depressed mood that is sustained over days, but without interference with normal activities and social interactions.<br>3: Moderate: Depressed mood that interferes with, but does not preclude, the patient's ability to carry out normal activities and social interactions.<br>4: Severe: Depressed mood precludes patient's ability to carry out normal activities and social interactions.	☐

July 1, 2008 *Copyright © 2008 Movement Disorder Society. All rights reserved.*
This chart may not be copied, distributed or otherwise used in whole or in part without prior written consent of the Movement Disorder Society. Page 4

	SCORE
**1.4 ANXIOUS MOOD**<br><br><u>Instructions to examiner:</u> Determine nervous, tense, worried or anxious feelings (including panic attacks) over the past week and rate their duration and interference with the patient's ability to carry out daily routines and engage in social interactions.<br><br><u>*Instructions to patients [and caregiver]:*</u> *Over the past week have you felt nervous, worried or tense? If yes, was this feeling for longer than one day at a time? Did it make it difficult for you to follow your usual activities or to be with other people*? [If yes, examiner asks patient or caregiver to elaborate and probes for information.]<br><br>0: Normal: No anxious feelings.<br><br>1: Slight: Anxious feelings present but not sustained for more than one day at a time. No interference with patient's ability to carry out normal activities and social interactions.<br><br>2: Mild: Anxious feelings are sustained over more than one day at a time, but without interference with patient's ability to carry out normal activities and social interactions.<br><br>3: Moderate: Anxious feelings interfere with, but do not preclude, the patient's ability to carry out normal activities and social interactions.<br><br>4: Severe: Anxious feelings preclude patient's ability to carry out normal activities and social interactions.	☐
**1.5 APATHY**<br><br><u>Instructions to examiner:</u> Consider level of spontaneous activity, assertiveness, motivation and initiative and rate the impact of reduced levels on performance of daily routines and social interactions. Here the examiner should attempt to distinguish between apathy and similar symptoms that are best explained by depression.<br><br><u>*Instructions to patients (and caregiver):*</u> *Over the past week, have you felt indifferent to doing activities or being with people?* If yes, examiner asks patient or caregiver to elaborate and probes for information.]<br><br>0: Normal: No apathy.<br><br>1: Slight: Apathy appreciated by patient and/or caregiver, but no interference with daily activities and social interactions.<br><br>2: Mild: Apathy interferes with isolated activities and social interactions.<br><br>3: Moderate: Apathy interferes with most activities and social interactions.<br><br>4: Severe: Passive and withdrawn, complete loss of initiative.	☐

 Copyright © 2008 Movement Disorder Society. All rights reserved.
This chart may not be copied, distributed or otherwise used in whole or in part without prior written consent of the Movement Disorder Society.

1.6 FEATURES OF DOPAMINE DYSREGULATION SYNDROME	SCORE
Instructions to examiner: Consider involvement in a variety of activities including atypical or excessive gambling (e.g. casinos or lottery tickets), atypical or excessive sexual drive or interests (e.g., unusual interest in pornography, masturbation, sexual demands on partner), other repetitive activities (e.g. hobbies, dismantling objects, sorting or organizing), or taking extra non-prescribed medication for non-physical reasons (i.e., addictive behavior). Rate the impact of such abnormal activities/behaviors on the patient's personal life and on his family and social relations (including need to borrow money or other financial difficulties like withdrawal of credit cards, major family conflicts, lost time from work, or missed meals or sleep because of the activity).<br>*Instructions to patients [and caregiver]: Over the past week, have you had unusually strong urges that are hard to control? Do you feel driven to do or think about something and find it hard to stop?* [Give patient examples such as gambling, cleaning, using the computer, taking extra medicine, obsessing about food or sex, all depending on the patients.<br><br>0: Normal: No problems present.<br>1: Slight: Problems are present but usually do not cause any difficulties for the patient or family/caregiver.<br>2: Mild: Problems are present and usually cause a few difficulties in the patient's personal and family life.<br>3: Moderate: Problems are present and usually cause a lot of difficulties in the patient's personal and family life.<br>4: Severe: Problems are present and preclude the patient's ability to carry out normal activities or social interactions or to maintain previous standards in personal and family life.	☐

The remaining questions in Part I (Non-motor Experiences of Daily Living) [Sleep, Daytime Sleepiness, Pain and Other Sensation, Urinary Problems, Constipation Problems, Lightheadedness on Standing, and Fatigue] are in the **Patient Questionnaire** along with all questions in Part II [Motor Experiences of Daily Living].

July 1, 2008 *Copyright © 2008 Movement Disorder Society. All rights reserved.* Page 6
This chart may not be copied, distributed or otherwise used in whole or in part without prior written consent of the Movement Disorder Society.

Patient Questionnaire:

Instructions:

This questionnaire will ask you about your experiences of daily living.

There are 20 questions. We are trying to be thorough, and some of these questions may therefore not apply to you now or ever. If you do not have the problem, simply mark 0 for NO.

Please read each one carefully and read all answers before selecting the one that best applies to you.

We are interested in your average or usual function over the past week including today. Some patients can do things better at one time of the day than at others. However, only one answer is allowed for each question, so please mark the answer that best describes what you can do most of the time.

You may have other medical conditions besides Parkinson's disease. Do not worry about separating Parkinson's disease from other conditions. Just answer the question with your best response.

Use only 0, 1, 2, 3, 4 for answers, nothing else. Do not leave any blanks.

Your doctor or nurse can review the questions with you, but this questionnaire is for patients to complete, either alone or with their caregivers.

Who is filling out this questionnaire (check the best answer):

☐ Patient ☐ Caregiver ☐ Patient and Caregiver in Equal Proportion

Copyright © 2008 Movement Disorder Society. All rights reserved.
This chart may not be copied, distributed or otherwise used in whole or in part without prior written consent of the Movement Disorder Society.

Part I: Non-Motor Aspects of Experiences of Daily Living (nM-EDL)

SCORE

1.7 SLEEP PROBLEMS

Over the past week, have you had trouble going to sleep at night or staying asleep through the night? Consider how rested you felt after waking up in the morning.

0: Normal: No problems.

1: Slight: Sleep problems are present but usually do not cause trouble getting a full night of sleep.

2: Mild: Sleep problems usually cause some difficulties getting a full night of sleep.

3: Moderate: Sleep problems cause a lot of difficulties getting a full night of sleep, but I still usually sleep for more than half the night.

4: Severe: I usually do not sleep for most of the night.

☐

1.8 DAYTIME SLEEPINESS

Over the past week, have you had trouble staying awake during the daytime?

0: Normal: No daytime sleepiness.

1: Slight: Daytime sleepiness occurs but I can resist and I stay awake.

2: Mild: Sometimes I fall asleep when alone and relaxing. For example, while reading or watching TV.

3: Moderate: I sometimes fall asleep when I should not. For example, while eating or talking with other people.

4: Severe: I often fall asleep when I should not. For example, while eating or talking with other people.

☐

July 1, 2008
Copyright © 2008 Movement Disorder Society. All rights reserved.
This chart may not be copied, distributed or otherwise used in whole or in part without prior written consent of the Movement Disorder Society.
Page 8

	SCORE
**1.9 PAIN AND OTHER SENSATIONS**<br><br>Over the past week, have you had uncomfortable feelings in your body like pain, aches tingling or cramps?<br><br>0: Normal: No uncomfortable feelings.<br><br>1: Slight: I have these feelings. However, I can do things and be with other people without difficulty.<br><br>2: Mild: These feelings cause some problems when I do things or am with other people.<br><br>3: Moderate: These feelings cause a lot of problems, but they do not stop me from doing things or being with other people.<br><br>4: Severe: These feelings stop me from doing things or being with other people.	☐
**1.10 URINARY PROBLEMS**<br><br>Over the past week, have you had trouble with urine control? For example, an urgent need to urinate, a need to urinate too often, or urine accidents?<br><br>0: Normal: No urine control problems.<br><br>1: Slight: I need to urinate often or urgently. However, these problems do not cause difficulties with my daily activities.<br><br>2: Mild: Urine problems cause some difficulties with my daily activities. However, I do not have urine accidents.<br><br>3: Moderate: Urine problems cause a lot of difficulties with my daily activities, including urine accidents.<br><br>4: Severe: I cannot control my urine and use a protective garment or have a bladder tube.	☐

July 1, 2008

Copyright © 2008 Movement Disorder Society. All rights reserved.
This chart may not be copied, distributed or otherwise used in whole or in part without prior written consent of the Movement Disorder Society.

	SCORE
**1.11 CONSTIPATION PROBLEMS**<br><br>Over the past week have you had constipation troubles that cause you difficulty moving your bowels?<br><br>0: Normal: No constipation.<br><br>1: Slight: I have been constipated. I use extra effort to move my bowels. However, this problem does not disturb my activities or my being comfortable.<br><br>2: Mild: Constipation causes me to have some troubles doing things or being comfortable.<br><br>3: Moderate: Constipation causes me to have a lot of trouble doing things or being comfortable. However, it does not stop me from doing anything.<br><br>4: Severe: I usually need physical help from someone else to empty my bowels.	☐
**1.12 LIGHT HEADEDNESS ON STANDING**<br><br>Over the past week, have you felt faint, dizzy or foggy when you stand up after sitting or lying down?<br><br>0: Normal: No dizzy or foggy feelings.<br><br>1: Slight: Dizzy or foggy feelings occur. However, they do not cause me troubles doing things.<br><br>2: Mild: Dizzy or foggy feelings cause me to hold on to something, but I do not need to sit or lie back down.<br><br>3: Moderate: Dizzy or foggy feelings cause me to sit or lie down to avoid fainting or falling.<br><br>4: Severe: Dizzy or foggy feelings cause me to fall or faint.	☐

Copyright © 2008 Movement Disorder Society. All rights reserved.
This chart may not be copied, distributed or otherwise used in whole or in part without prior written consent of the Movement Disorder Society.

	SCORE
**1.13 FATIGUE**<br><br>Over the past week, have you usually felt fatigued? This feeling is not part of being sleepy or sad<br><br>0: Normal: No fatigue.<br><br>1: Slight: Fatigue occurs. However it does not cause me troubles doing things or being with people.<br><br>2: Mild: Fatigue causes me some troubles doing things or being with people.<br><br>3: Moderate: Fatigue causes me a lot of troubles doing things or being with people. However, it does not stop me from doing anything.<br><br>4: Severe: Fatigue stops me from doing things or being with people.	☐
**Part II: Motor Aspects of Experiences of Daily Living (M-EDL)**	
**2.1 SPEECH**<br><br>Over the past week, have you had problems with your speech?<br><br>0: Normal: Not at all (no problems).<br><br>1: Slight: My speech is soft, slurred or uneven, but it does not cause others to ask me to repeat myself.<br><br>2: Mild: My speech causes people to ask me to occasionally repeat myself, but not everyday.<br><br>3: Moderate: My speech is unclear enough that others ask me to repeat myself every day even though most of my speech is understood.<br><br>4: Severe: Most or all of my speech cannot be understood.	☐

July 1, 2008 *Copyright © 2008 Movement Disorder Society. All rights reserved.* Page 11
This chart may not be copied, distributed or otherwise used in whole or in part without prior written consent of the Movement Disorder Society.

	SCORE
**2.2 SALIVA & DROOLING**<br><br>Over the past week, have you usually had too much saliva during when you are awake or when you sleep?<br><br>0: Normal: Not at all (no problems).<br>1: Slight: I have too much saliva, but do not drool.<br>2: Mild: I have some drooling during sleep, but none when I am awake.<br>3: Moderate: I have some drooling when I am awake, but I usually do not need tissues or a handkerchief.<br>4: Severe: I have so much drooling that I regularly need to use tissues or a handkerchief to protect my clothes.	☐
**2.3 CHEWING AND SWALLOWING**<br><br>Over the past week, have you usually had problems swallowing pills or eating meals? Do you need your pills cut or crushed or your meals to be made soft, chopped or blended to avoid choking?<br><br>0: Normal: No problems.<br>1: Slight: I am aware of slowness in my chewing or increased effort at swallowing, but I do not choke or need to have my food specially prepared.<br>2: Mild: I need to have my pills cut or my food specially prepared because of chewing or swallowing problems, but I have not choked over the past week.<br>3: Moderate. I choked at least once in the past week.<br>4: Severe: Because of chewing and swallowing problems, I need a feeding tube.	☐

July 1, 2008
Copyright © 2008 Movement Disorder Society. All rights reserved.
This chart may not be copied, distributed or otherwise used in whole or in part without prior written consent of the Movement Disorder Society.
Page 12

SCORE

2.4 EATING TASKS

Over the past week, have you usually had troubles handling your food and using eating utensils? For example, do you have trouble handling finger foods or using forks, knifes, spoons, chopsticks?

0:	Normal:	Not at all (No problems).
1:	Slight:	I am slow, but I do not need any help handling my food and have not had food spills while eating.
2:	Mild:	I am slow with my eating and have occasional food spills. I may need help with a few tasks such as cutting meat.
3:	Moderate:	I need help with many eating tasks but can manage some alone.
4:	Severe:	I need help for most or all eating tasks.

☐

2.5 DRESSING

Over the past week, have you usually had problems dressing? For example, are you slow or do you need help with buttoning, using zippers, putting on or taking off your clothes or jewelry?

0:	Normal:	Not at all (no problems).
1:	Slight:	I am slow but I do not need help.
2:	Mild:	I am slow and need help for a few dressing tasks (buttons, bracelets).
3:	Moderate:	I need help for many dressing tasks.
4:	Severe:	I need help for most or all dressing tasks.

☐

Copyright © 2008 Movement Disorder Society. All rights reserved.

This chart may not be copied, distributed or otherwise used in whole or in part without prior written consent of the Movement Disorder Society.

	SCORE
**2.6 HYGIENE**<br><br>Over the past week, have you usually been slow or do you need help with washing, bathing, shaving, brushing teeth, combing your hair or with other personal hygiene?<br><br>0: Normal: Not at all (no problems).<br>1: Slight: I am slow but I do not need any help.<br>2: Mild: I need someone else to help me with some hygiene tasks.<br>3: Moderate: I need help for many hygiene tasks.<br>4: Severe: I need help for most or all of my hygiene tasks.	☐
**2.7 HANDWRITING**<br><br>Over the past week, have people usually had trouble reading your handwriting?<br><br>0: Normal: Not at all (no problems).<br>1: Slight: My writing is slow, clumsy or uneven, but all words are clear.<br>2: Mild: Some words are unclear and difficult to read.<br>3: Moderate: Many words are unclear and difficult to read.<br>4: Severe: Most or all words cannot be read.	☐
**2.8 DOING HOBBIES AND OTHER ACTIVITIES**<br><br>Over the past week, have you usually had trouble doing your hobbies or other things that you like to do?<br><br>0: Normal: Not at all (no problems).<br>1: Slight: I am a bit slow but do these activities easily.<br>2: Mild: I have some difficulty doing these activities.<br>3: Moderate: I have major problems doing these activities, but still do most.<br>4: Severe: I am unable to do most or all of these activities.	☐

July 1, 2008 *Copyright © 2008 Movement Disorder Society. All rights reserved.* Page 14
This chart may not be copied, distributed or otherwise used in whole or in part without prior written consent of the Movement Disorder Society.

	SCORE
**2.9 TURNING IN BED**<br><br>Over the past week, do you usually have trouble turning over in bed?<br><br>0: Normal: Not at all (no problems).<br>1: Slight: I have a bit of trouble turning, but I do not need any help.<br>2: Mild I have a lot of trouble turning and need occasional help from someone else.<br>3: Moderate: To turn over I often need help from someone else.<br>4: Severe: I am unable to turn over without help from someone else.	☐
**2.10 TREMOR**<br><br>Over the past week, have you usually had shaking or tremor?<br><br>0: Normal: Not at all. I have no shaking or tremor.<br>1: Slight: Shaking or tremor occurs but does not cause problems with any activities.<br>2: Mild: Shaking or tremor causes problems with only a few activities.<br>3: Moderate: Shaking or tremor causes problems with many of my daily activities.<br>4: Severe: Shaking or tremor causes problems with most or all activities.	☐
**2.11 GETTING OUT OF BED, A CAR, OR A DEEP CHAIR**<br><br>Over the past week, have you usually had trouble getting out of bed, a car seat, or a deep chair?<br><br>0: Normal: Not at all (no problems).<br>1: Slight: I am slow or awkward, but I usually can do it on my first try.<br>2: Mild: I need more than one try to get up or need occasional help.<br>3: Moderate: I sometimes need help to get up, but most times I can still do it on my own.<br>4: Severe: I need help most or all of the time.	☐

Copyright © 2008 Movement Disorder Society. All rights reserved.

This chart may not be copied, distributed or otherwise used in whole or in part without prior written consent of the Movement Disorder Society.

	SCORE
**2.12 WALKING AND BALANCE**<br><br>Over the past week, have you usually had problems with balance and walking?<br><br>0: Normal: Not at all (no problems).<br><br>1: Slight: I am slightly slow or may drag a leg. I never use a walking aid.<br><br>2: Mild: I occasionally use a walking aid, but I do not need any help from another person.<br><br>3: Moderate: I usually use a walking aid (cane, walker) to walk safely without falling. However, I do not usually need the support of another person.<br><br>4: Severe: I usually use the support of another persons to walk safely without falling.	☐
**2.13 FREEZING**<br><br>Over the past week, on your usual day when walking, do you suddenly stop or freeze as if your feet are stuck to the floor.<br><br>0: Normal: Not at all (no problems).<br><br>1: Slight: I briefly freeze but I can easily start walking again. I do not need help from someone else or a walking aid (cane or walker) because of freezing.<br><br>2: Mild: I freeze and have trouble starting to walk again, but I do not need someone's help or a walking aid (cane or walker) because of freezing.<br><br>3: Moderate: When I freeze I have a lot of trouble starting to walk again and, because of freezing, I sometimes need to use a walking aid or need someone else's help.<br><br>4: Severe: Because of freezing, most or all of the time, I need to use a walking aid or someone's help.	☐

This completes the questionnaire. We may have asked about problems you do not even have, and may have mentioned problems that you may never develop at all. Not all patients develop all these problems, but because they can occur, it is important to ask all the questions to every patient. Thank you for your time and attention in completing this questionnaire.

Copyright © 2008 Movement Disorder Society. All rights reserved.
This chart may not be copied, distributed or otherwise used in whole or in part without prior written consent of the Movement Disorder Society.

Part III: Motor Examination

Overview: This portion of the scale assesses the motor signs of PD. In administering Part III of the MDS-UPDRS the examiner should comply with the following guidelines:

At the top of the form, mark whether the patient is on medication for treating the symptoms of Parkinson's disease and, if on levodopa, the time since the last dose.

Also, if the patient is receiving medication for treating the symptoms of Parkinson's Disease, mark the patient's clinical state using the following definitions:

ON is the typical functional state when patients are receiving medication and have a good response.
OFF is the typical functional state when patients have a poor response in spite of taking medications.

The investigator should "rate what you see". Admittedly, concurrent medical problems such as stroke, paralysis, arthritis, contracture, and orthopedic problems such as hip or knee replacement and scoliosis may interfere with individual items in the motor examination. In situations where it is absolutely impossible to test (e.g., amputations, plegia, limb in a cast), use the notation "**UR**" for Unable to Rate. Otherwise, rate the performance of each task as the patient performs in the context of co-morbidities.

All items must have an integer rating (no half points, no missing ratings).

Specific instructions are provided for the testing of each item. These should be followed in all instances. The investigator demonstrates while describing tasks the patient is to perform and rates function immediately thereafter. For Global Spontaneous Movement and Rest Tremor items (3.14 and 3.17), these items have been placed purposefully at the end of the scale because clinical information pertinent to the score will be obtained throughout the entire examination.

At the end of the rating, indicate if dyskinesia (chorea or dystonia) was present at the time of the examination, and if so, whether these movements interfered with the motor examination.

3a Is the patient on medication for treating the symptoms of Parkinson's Disease? ☐ No ☐ Yes

3b If the patient is receiving medication for treating the symptoms of Parkinson's Disease, mark the patient's clinical state using the following definitions:

☐ ON: On is the typical functional state when patients are receiving medication and have a good response.

☐ OFF: Off is the typical functional state when patients have a poor response in spite of taking medications.

3c Is the patient on Levodopa ? ☐ No ☐ Yes

3.C1 If yes, minutes since last levodopa dose: __________

Copyright © 2008 Movement Disorder Society. All rights reserved.

This chart may not be copied, distributed or otherwise used in whole or in part without prior written consent of the Movement Disorder Society.

	SCORE
**3.1 SPEECH**<br><br><u>Instructions to examiner</u>: Listen to the patient's free-flowing speech and engage in conversation if necessary. Suggested topics: ask about the patient's work, hobbies, exercise, or how he got to the doctor's office. Evaluate volume, modulation (prosody) and clarity, including slurring, palilalia (repetition of syllables) and tachyphemia (rapid speech, running syllables together).<br><br>0: Normal: No speech problems.<br>1: Slight: Loss of modulation, diction or volume, but still all words easy to understand.<br>2: Mild: Loss of modulation, diction, or volume, with a few words unclear, but the overall sentences easy to follow.<br>3: Moderate: Speech is difficult to understand to the point that some, but not most, sentences are poorly understood.<br>4: Severe: Most speech is difficult to understand or unintelligible.	☐
**3.2 FACIAL EXPRESSION**<br><br><u>Instructions to examiner</u>: Observe the patient sitting at rest for 10 seconds, without talking and also while talking. Observe eye-blink frequency, masked facies or loss of facial expression, spontaneous smiling and parting of lips.<br><br>0: Normal: Normal facial expression.<br>1: Slight: Minimal masked facies manifested only by decreased frequency of blinking.<br>2: Mild: In addition to decreased eye-blink frequency, Masked facies present in the lower face as well, namely fewer movements around the mouth, such as less spontaneous smiling, but lips not parted.<br>3: Moderate: Masked facies with lips parted some of the time when the mouth is at rest.<br>4: Severe: Masked facies with lips parted most of the time when the mouth is at rest.	☐

Copyright © 2008 Movement Disorder Society. All rights reserved.
This chart may not be copied, distributed or otherwise used in whole or in part without prior written consent of the Movement Disorder Society.

SCORE

3.3 RIGIDITY

Instructions to examiner: Rigidity is judged on slow passive movement of major joints with the patient in a relaxed position and the examiner manipulating the limbs and neck. First, test without an activation maneuver. Test and rate neck and each limb separately. For arms, test the wrist and elbow joints simultaneously. For legs, test the hip and knee joints simultaneously. If no rigidity is detected, use an activation maneuver such as tapping fingers, fist opening/closing, or heel tapping in a limb not being tested. Explain to the patient to go as limp as possible as you test for rigidity.

0:	Normal:	No rigidity.
1:	Slight:	Rigidity only detected with activation maneuver.
2:	Mild:	Rigidity detected without the activation maneuver, but full range of motion is easily achieved.
3:	Moderate:	Rigidity detected without the activation maneuver; full range of motion is achieved with effort.
4:	Severe:	Rigidity detected without the activation maneuver and full range of motion not achieved.

Score: Neck ☐ RUE ☐ LUE ☐ RLE ☐ LLE ☐

3.4 FINGER TAPPING

Instructions to examiner: Each hand is tested separately. Demonstrate the task, but do not continue to perform the task while the patient is being tested. Instruct the patient to tap the index finger on the thumb 10 times as quickly AND as big as possible. Rate each side separately, evaluating speed, amplitude, hesitations, halts and decrementing amplitude.

0:	Normal:	No problems.
1:	Slight:	Any of the following: a) the regular rhythm is broken with one or two interruptions or hesitations of the tapping movement; b) slight slowing; c) the amplitude decrements near the end of the 10 taps.
2:	Mild:	Any of the following: a) 3 to 5 interruptions during tapping; b) mild slowing; c) the amplitude decrements midway in the 10-tap sequence.
3:	Moderate:	Any of the following: a) more than 5 interruptions during tapping or at least one longer arrest (freeze) in ongoing movement; b) moderate slowing; c) the amplitude decrements starting after the 1st tap.
4:	Severe:	Cannot or can only barely perform the task because of slowing, interruptions or decrements.

Score: R ☐ L ☐

July 1, 2008
Copyright © 2008 Movement Disorder Society. All rights reserved.
This chart may not be copied, distributed or otherwise used in whole or in part without prior written consent of the Movement Disorder Society.
Page 19

	SCORE
**3.5 HAND MOVEMENTS**<br><br><u>Instructions to examiner</u>: Test each hand separately. Demonstrate the task, but do not continue to perform the task while the patient is being tested. Instruct the patient to make a tight fist with the arm bent at the elbow so that the palm faces the examiner. Have the patient open the hand 10 times as fully AND as quickly as possible. If the patient fails to make a tight fist or to open the hand fully, remind him/ her to do so. Rate each side separately, evaluating speed, amplitude, hesitations, halts and decrementing amplitude.<br><br>0: Normal: No problem.<br><br>1: Slight: Any of the following: a) the regular rhythm is broken with one or two interruptions or hesitations of the movement; b) slight slowing; c) the amplitude decrements near the end of the task.<br><br>2: Mild: Any of the following: a) 3 to 5 interruptions during the movements; b) mild slowing; c) the amplitude decrements midway in the task.<br><br>3: Moderate: Any of the following: a) more than 5 interruptions during the movement or at least one longer arrest (freeze) in ongoing movement; b) moderate slowing; c) the amplitude decrements starting after the 1st open-and-close sequence.<br><br>4: Severe: Cannot or can only barely perform the task because of slowing, interruptions or decrements.	☐ R<br><br>☐ L
**3.6 PRONATION-SUPINATION MOVEMENTS OF HANDS**<br><br><u>Instructions to examiner</u>: Test each hand separately. Demonstrate the task, but do not continue to perform the task while the patient is being tested. Instruct the patient to extend the arm out in front of his/her body with the palms down; then to turn the palm up and down alternately 10 times as fast and as fully as possible. Rate each side separately, evaluating speed, amplitude, hesitations, halts and decrementing amplitude.<br><br>0: Normal: No problems.<br><br>1: Slight: Any of the following: a) the regular rhythm is broken with one or two interruptions or hesitations of the movement; b) slight slowing; c) the amplitude decrements near the end of the sequence.<br><br>2: Mild: Any of the following: a) 3 to 5 interruptions during the movements; b) mild slowing; c) the amplitude decrements midway in the sequence.<br><br>3: Moderate: Any of the following: a) more than 5 interruptions during the movement or at least one longer arrest (freeze) in ongoing movement; b) moderate slowing c) the amplitude decrements starting after the 1st supination-pronation sequence.<br><br>4: Severe: Cannot or can only barely perform the task because of slowing, interruptions or decrements.	☐ R<br><br>☐ L

Copyright © 2008 Movement Disorder Society. All rights reserved.

This chart may not be copied, distributed or otherwise used in whole or in part without prior written consent of the Movement Disorder Society.

SCORE

3.7 TOE TAPPING

Instructions to examiner: Have the patient sit in a straight-backed chair with arms, both feet on the floor. Test each foot separately. Demonstrate the task, but do not continue to perform the task while the patient is being tested. Instruct the patient to place the heel on the ground in a comfortable position and then tap the toes 10 times as big and as fast as possible. Rate each side separately, evaluating speed, amplitude, hesitations, halts and decrementing amplitude.

0: Normal: No problem.

1: Slight: Any of the following: a) the regular rhythm is broken with one or two interruptions or hesitations of the tapping movement; b) slight slowing; c) amplitude decrements near the end of the ten taps.

2: Mild: Any of the following: a) 3 to 5 interruptions during the tapping movements; b) mild slowing; c) amplitude decrements midway in the task.

3: Moderate: Any of the following: a) more than 5 interruptions during the tapping movements or at least one longer arrest (freeze) in ongoing movement; b) moderate slowing; c) amplitude decrements after the first tap.

4: Severe: Cannot or can only barely perform the task because of slowing, interruptions or decrements.

R

L

3.8 LEG AGILITY

Instructions to examiner: Have the patient sit in a straight-backed chair with arms. The patient should have both feet comfortably on the floor. Test each leg separately. Demonstrate the task, but do not continue to perform the task while the patient is being tested. Instruct the patient to place the foot on the ground in a comfortable position and then raise and stomp the foot on the ground 10 times as high and as fast as possible. Rate each side separately, evaluating speed, amplitude, hesitations, halts and decrementing amplitude.

0: Normal: No problems.

1: Slight: Any of the following: a) the regular rhythm is broken with one or two interruptions or hesitations of the movement; b) slight slowing; c) amplitude decrements near the end of the task.

2: Mild: Any of the following: a) 3 to 5 interruptions during the movements; b) mild slowness; c) amplitude decrements midway in the task.

3: Moderate: Any of the following: a) more than 5 interruptions during the movement or at least one longer arrest (freeze) in ongoing movement; b) moderate slowing in speed; c) amplitude decrements after the first tap.

4: Severe: Cannot or can only barely perform the task because of slowing, interruptions or decrements.

R

L

Copyright © 2008 Movement Disorder Society. All rights reserved.
This chart may not be copied, distributed or otherwise used in whole or in part without prior written consent of the Movement Disorder Society.

SCORE

3.9 ARISING FROM CHAIR

Instructions to examiner: Have the patient sit in a straight-backed chair with arms, with both feet on the floor and sitting back in the chair (if the patient is not too short). Ask the patient to cross his/her arms across the chest and then to stand up. If the patient is not successful, repeat this attempt a maximum up to two more times. If still unsuccessful, allow the patient to move forward in the chair to arise with arms folded across the chest. Allow only one attempt in this situation. If unsuccessful, allow the patient to push off using his/her hands on the arms of the chair. Allow a maximum of three trials of pushing off. If still not successful, assist the patient to arise. After the patient stands up, observe the posture for item 3.13

0: Normal: No problems. Able to arise quickly without hesitation.

1: Slight: Arising is slower than normal; or may need more than one attempt; or may need to move forward in the chair to arise. No need to use the arms of the chair.

2: Mild: Pushes self up from arms of chair without difficulty.

3: Moderate: Needs to push off, but tends to fall back; or may have to try more than one time using arms of chair, but can get up without help.

4: Severe: Unable to arise without help.

☐

3.10 GAIT

Instructions to examiner: Testing gait is best performed by having the patient walking away from and towards the examiner so that both right and left sides of the body can be easily observed simultaneously. The patient should walk at least 10 meters (30 feet), then turn around and return to the examiner. This item measures multiple behaviors: stride amplitude, stride speed, height of foot lift, heel strike during walking, turning, and arm swing, but not freezing. Assess also for "freezing of gait" (next item 3.11) while patient is walking. Observe posture for item 3.13

0: Normal: No problems.

1: Slight: Independent walking with minor gait impairment.

2: Mild: Independent walking but with substantial gait impairment.

3: Moderate: Requires an assistance device for safe walking (walking stick, walker) but not a person.

4: Severe: Cannot walk at all or only with another person's assistance.

☐

Copyright © 2008 Movement Disorder Society. All rights reserved.

This chart may not be copied, distributed or otherwise used in whole or in part without prior written consent of the Movement Disorder Society.

	SCORE
**3.11 FREEZING OF GAIT**<br><br><u>Instructions to examiner:</u> While assessing gait, also assess for the presence of any gait freezing episodes. Observe for start hesitation and stuttering movements especially when turning and reaching the end of the task. To the extent that safety permits, patients may NOT use sensory tricks during the assessment.<br><br>0: Normal: No freezing.<br><br>1: Slight: Freezes on starting, turning or walking through doorway with a single halt during any of these events, but then continues smoothly without freezing during straight walking.<br><br>2: Mild: Freezes on starting, turning or walking through doorway with more than one halt during any of these activities, but continues smoothly without freezing during straight walking.<br><br>3: Moderate: Freezes once during straight walking.<br><br>4: Severe: Freezes multiple times during straight walking.	☐
**3.12 POSTURAL STABILITY**<br><br><u>Instructions to examiner</u>: The test examines the response to sudden body displacement produced by a <u>quick, forceful</u> pull on the shoulders while the patient is standing erect with eyes open and feet comfortably apart and parallel to each other. Test retropulsion. Stand behind the patient and instruct the patient on what is about to happen. Explain that s/he is allowed to take a step backwards to avoid falling. There should be a solid wall behind the examiner, at least 1-2 meters away to allow for the observation of the number of retropulsive steps. The first pull is an instructional demonstration and is purposely milder and not rated. The second time the shoulders are pulled briskly and forcefully towards the examiner with enough force to displace the center of gravity so that patient MUST take a step backwards. The examiner needs to be ready to catch the patient, but must stand sufficiently back so as to allow enough room for the patient to take several steps to recover independently. Do not allow the patient to flex the body abnormally forward in anticipation of the pull. Observe for the number of steps backwards or falling. Up to and including two steps for recovery is considered normal, so abnormal ratings begin with three steps. If the patient fails to understand the test, the examiner can repeat the test so that the rating is based on an assessment that the examiner feels reflects the patient's limitations rather than misunderstanding or lack of preparedness. Observe standing posture for item 3.13<br><br>0: Normal: No problems: Recovers with one or two steps.<br><br>1: Slight: 3-5 steps, but subject recovers unaided.<br><br>2: Mild: More than 5 steps, but subject recovers unaided.<br><br>3: Moderate: Stands safely, but with absence of postural response; falls if not caught by examiner.<br><br>4: Severe: Very unstable, tends to lose balance spontaneously or with just a gentle pull on the shoulders.	☐

Copyright © 2008 Movement Disorder Society. All rights reserved.

This chart may not be copied, distributed or otherwise used in whole or in part without prior written consent of the Movement Disorder Society.

SCORE

3.13 POSTURE

Instructions to examiner: Posture is assessed with the patient standing erect after arising from a chair, during walking , and while being tested for postural reflexes. If you notice poor posture, tell the patient to stand up straight and see if the posture improves (see option 2 below). Rate the worst posture seen in these three observation points. Observe for flexion and side-to-side leaning.

0: Normal: No problems.

1: Slight: Not quite erect, but posture could be normal for older person.

2: Mild: Definite flexion, scoliosis or leaning to one side, but patient can correct posture to normal posture when asked to do so.

3: Moderate: Stooped posture, scoliosis or leaning to one side that cannot be corrected volitionally to a normal posture by the patient.

4: Severe: Flexion, scoliosis or leaning with extreme abnormality of posture.

3.14 GLOBAL SPONTANEITY OF MOVEMENT (BODY BRADYKINESIA)

Instructions to examiner: This global rating combines all observations on slowness, hesitancy, and small amplitude and poverty of movement in general, including a reduction of gesturing and of crossing the legs. This assessment is based on the examiner's global impression after observing for spontaneous gestures while sitting, and the nature of arising and walking.

0: Normal: No problems.

1: Slight: Slight global slowness and poverty of spontaneous movements.

2: Mild: Mild global slowness and poverty of spontaneous movements.

3: Moderate: Moderate global slowness and poverty of spontaneous movements.

4: Severe: Severe global slowness and poverty of spontaneous movements.

3.15 POSTURAL TREMOR OF THE HANDS

Instructions to examiner: All tremor, including re-emergent rest tremor, that is present in this posture is to be included in this rating. Rate each hand separately. Rate the highest amplitude seen. Instruct the patient to stretch the arms out in front of the body with palms down. The wrist should be straight and the fingers comfortably separated so that they do not touch each other. Observe this posture for 10 seconds.

0: Normal: No tremor.

1: Slight: Tremor is present but less than 1 cm in amplitude.

2: Mild: Tremor is at least 1 but less than 3 cm in amplitude.

3: Moderate: Tremor is at least 3 but less than 10 cm in amplitude.

4: Severe: Tremor is at least 10 cm in amplitude.

R

L

Copyright © 2008 Movement Disorder Society. All rights reserved.
This chart may not be copied, distributed or otherwise used in whole or in part without prior written consent of the Movement Disorder Society.

	SCORE
**3.16 KINETIC TREMOR OF THE HANDS**<br><br>Instructions to examiner: This is tested by the finger-to-nose maneuver. With the arm starting from the outstretched position, have the patient perform at least three finger-to-nose maneuvers with each hand reaching as far as possible to touch the examiner's finger. The finger-to-nose maneuver should be performed slowly enough not to hide any tremor that could occur with very fast arm movements. Repeat with the other hand, rating each hand separately. The tremor can be present throughout the movement or as the tremor reaches either target (nose or finger). Rate the highest amplitude seen.<br><br>0: Normal: No tremor.<br>1: Slight: Tremor is present but less than 1 cm in amplitude.<br>2: Mild: Tremor is at least 1 but less than 3 cm in amplitude.<br>3: Moderate: Tremor is at least 3 but less than 10 cm in amplitude.<br>4: Severe: Tremor is at least 10 cm in amplitude.	R<br><br>L
**3.17 REST TREMOR AMPLITUDE**<br><br>Instructions to examiner: This and the next item have been placed purposefully at the end of the examination to allow the rater to gather observations on rest tremor that may appear at any time during the exam, including when quietly sitting, during walking and during activities when some body parts are moving but others are at rest. Score the maximum amplitude that is seen at any time as the final score. Rate only the amplitude and not the persistence or the intermittency of the tremor.<br>As part of this rating, the patient should sit quietly in a chair with the hands placed on the arms of the chair (not in the lap) and the feet comfortably supported on the floor for 10 seconds with no other directives. Rest tremor is assessed separately for all four limbs and also for the lip/jaw. Rate only the maximum amplitude that is seen at any time as the final rating.<br><br>Extremity ratings<br>0: Normal: No tremor.<br>1: Slight.: < 1 cm in maximal amplitude.<br>2: Mild: > 1 cm but < 3 cm in maximal amplitude.<br>3: Moderate: 3 - 10 cm in maximal amplitude.<br>4: Severe: > 10 cm in maximal amplitude.<br><br>Lip/Jaw ratings<br>0: Normal: No tremor.<br>1: Slight: < 1 cm in maximal amplitude.<br>2: Mild: > 1 cm but < 2 cm in maximal amplitude.<br>3: Moderate: > 2 cm but < 3 cm in maximal amplitude.<br>4: Severe: > 3 cm in maximal amplitude.	RUE<br><br>LUE<br><br>RLE<br><br>LLE<br><br>Lip/Jaw

Copyright © 2008 Movement Disorder Society. All rights reserved.

This chart may not be copied, distributed or otherwise used in whole or in part without prior written consent of the Movement Disorder Society.

SCORE

3.18 CONSTANCY OF REST TREMOR

Instructions to examiner: This item receives one rating for all rest tremor and focuses on the constancy of rest tremor during the examination period when different body parts are variously at rest. It is rated purposefully at the end of the examination so that several minutes of information can be coalesced into the rating.

0: Normal: No tremor.

1: Slight: Tremor at rest is present < 25% of the entire examination period.

2: Mild: Tremor at rest is present 26-50% of the entire examination period.

3: Moderate: Tremor at rest is present 51-75% of the entire examination period.

4: Severe: Tremor at rest is present > 75% of the entire examination period.

☐

DYSKINESIA IMPACT ON PART III RATINGS

A. Were dyskinesias (chorea or dystonia) present during examination? ☐ No ☐ Yes

B. If yes, did these movements interfere with your ratings? ☐ No ☐ Yes

HOEHN AND YAHR STAGE

0: Asymptomatic.

1: Unilateral involvement only.

2: Bilateral involvement without impairment of balance.

3: Mile to moderate involvement; some postural instability but physically independent; needs assistance to recover from pull test.

4: Severe disability; still able to walk or stand unassisted.

5: Wheelchair bound or bedridden unless aided.

☐

July 1, 2008
Copyright © 2008 Movement Disorder Society. All rights reserved.
This chart may not be copied, distributed or otherwise used in whole or in part without prior written consent of the Movement Disorder Society.
Page 26

Part IV: Motor Complications

Overview and Instructions: In this section, the rater uses historical and objective information to assess two motor complications, dyskinesias and motor fluctuations that include OFF-state dystonia. Use all information from patient, caregiver, and the examination to answer the six questions that summarize function over the past week including today. As in the other sections, rate using only integers (no half points allowed) and leave no missing ratings. If the item cannot be rated, place UR for Unable to Rate. You will need to choose some answers based on percentages, and therefore you will need to establish how many hours generally are awake hours and use this figure as the denominator for "OFF" time and Dyskinesias. For "OFF dystonia", the total "Off" time will be the denominator. Operational definitions for examiner's use.

Dyskinesias: Involuntary random movements
Words that patients often recognize for dyskinesias include "irregular jerking", "wiggling", "twitching". It is essential to stress to the patient the difference between dyskinesias and tremor, a common error when patients are assessing dyskinesias.

Dystonia: contorted posture, often with a twisting component:
Words that patients often recognize for dystonia include "spasms", "cramps", "posture".

Motor fluctuation: Variable response to medication:
Words that patients often recognize for motor fluctuation include "wearing out", "wearing off", "roller-coaster effect", "on-off", "uneven medication effects".

OFF: Typical functional state when patients have a poor response in spite of taking mediation or the typical functional response when patients are on NO treatment for parkinsonism. Words that patients often recognize include "low time", "bad time", "shaking time", "slow time", "time when my medications don't work."

ON: Typical functional state when patients are receiving medication and have a good response:
Words that patients often recognize include "good time", "walking time", "time when my medications work."

A . DYSKINESIAS [exclusive of OFF-state dystonia]

4.1 TIME SPENT WITH DYSKINESIAS — SCORE ☐

Instructions to examiner: Determine the hours in the usual waking day and then the hours of dyskinesias. Calculate the percentage. If the patient has dyskinesias in the office, you can point them out as a reference to ensure that patients and caregivers understand what they are rating. You may also use your own acting skills to enact the dyskinetic movements you have seen in the patient before or show them dyskinetic movements typical of other patients. Exclude from this question early morning and nighttime painful dystonia.

Instructions to patient [and caregiver]. Over the past week, how many hours do you usually sleep on a daily basis, including nighttime sleep and daytime napping? Alright, if you sleep ___ hrs, you are awake ____ hrs. Out of those awake hours, how many hours in total do you have wiggling, twitching or jerking movements? Do not count the times when you have tremor, which is a regular back and forth shaking or times when you have painful foot cramps or spasms in the early morning or at nighttime. I will ask about those later. Concentrate only on these types of wiggling, jerking and irregular movements. Add up all the time during the waking day when these usually occur. How many hours ____ (use this number for your calculation).

0: Normal:	No dyskinesias.	
1: Slight:	≤ 25% of waking day.	
2: Mild:	26 - 50% of waking day.	1. Total Hours Awake: _____
3: Moderate:	51 - 75% of waking day.	2. Total Hours with Dyskinesia: _____
4: Severe:	> 75% of waking day.	3. % Dyskinesia = ((2/1)*100): _____

July 1, 2008 Copyright © 2008 Movement Disorder Society. All rights reserved. Page 27
This chart may not be copied, distributed or otherwise used in whole or in part without prior written consent of the Movement Disorder Society.

SCORE

4.2 FUNCTIONAL IMPACT OF DYSKINESIAS

Instructions to examiner: Determine the degree to which dyskinesias impact on the patient's daily function in terms of activities and social interactions. Use the patient's and caregiver's response to your question and your own observations during the office visit to arrive at the best answer.

Instructions to patient [and caregiver]: Over the past week, did you usually have trouble doing things or being with people when these jerking movements occurred? Did they stop you from doing things or from being with people?

0: Normal:	No dyskinesias or no impact by dyskinesias on activities or social interactions.
1: Slight:	Dyskinesias impact on a few activities, but the patient usually performs all activities and participates in all social interactions during dyskinetic periods.
2: Mild:	Dyskinesias impact on many activities, but the patient usually performs all activities and participates in all social interactions during dyskinetic periods.
3: Moderate:	Dyskinesias impact on activities to the point that the patient usually does not perform some activities or does not usually participate in some social activities during dyskinetic episodes.
4: Severe:	Dyskinesias impact on function to the point that the patient usually does not perform most activities or participate in most social interactions during dyskinetic episodes.

☐

B . MOTOR FLUCTUATIONS

4.3 TIME SPENT IN THE OFF STATE

Instructions to examiner: Use the number of waking hours derived from 4.1 and determine the hours spent in the "OFF" state. Calculate the percentage. If the patient has an OFF period in the office, you can point to this state as a reference. You may also use your knowledge of the patient to describe a typical OFF period. Additionally you may use your own acting skills to enact an OFF period you have seen in the patient before or show them OFF function typical of other patients. Mark down the typical number of OFF hours, because you will need this number for completing 4.6

Instructions to patient [and caregiver]: Some patients with Parkinson's disease have a good effect from their medications throughout their awake hours and we call that "ON" time. Other patients take their medications but still have some hours of low time, bad time, slow time or shaking time. Doctors call these low periods "OFF" time. Over the past week, you told me before that you are generally awake ____ hrs each day. Out of these awake hours, how many hours in total do you usually have this type of low level or OFF function ____ (Use this number for your calculations).

0: Normal:	No OFF time.
1: Slight:	≤ 25% of waking day.
2: Mild:	26 - 50% of waking day.
3: Moderate:	51 - 75% of waking day.
4: Severe:	> 75% of waking day.

1. Total Hours Awake: _____
2. Total Hours OFF: _____
3. % OFF = ((2/1)*100): _____

☐

July 1, 2008

Copyright © 2008 Movement Disorder Society. All rights reserved.
This chart may not be copied, distributed or otherwise used in whole or in part without prior written consent of the Movement Disorder Society.

Page 28

	SCORE
**4.4 FUNCTIONAL IMPACT OF FLUCTUATIONS**<br><br><u>Instructions to examiner:</u> Determine the degree to which motor fluctuations impact on the patient's daily function in terms of activities and social interactions. This question concentrates on the difference between the ON state and the OFF state. If the patient has no OFF time, the rating must be 0, but if patients have very mild fluctuations, it is still possible to be rated 0 on this item if no impact on activities occurs. Use the patient's and caregiver's response to your question and your own observations during the office visit to arrive at the best answer.<br><br>*<u>Instructions to patient [and caregiver]:</u> Think about when those low or "OFF" periods have occurred over the past week. Do you usually have more problems doing things or being with people than compared to the rest of the day when you feel your medications working? Are there some things you usually do during a good period that you have trouble with or stop doing during a low period?*<br><br>0: Normal: No fluctuations or No impact by fluctuations on performance of activities or social interactions.<br><br>1: Slight: Fluctuations impact on a few activities, but during OFF, the patient usually performs all activities and participates in all social interactions that typically occur during the ON state.<br><br>2: Mild: Fluctuations impact many activities, but during OFF, the patient still usually performs all activities and participates in all social interactions that typically occur during the ON state.<br><br>3: Moderate: Fluctuations impact on the performance of activities during OFF to the point that the patient usually does not perform some activities or participate in some social interactions that are performed during ON periods.<br><br>4: Severe: Fluctuations impact on function to the point that, during OFF, the patient usually does not perform most activities or participate in most social interactions that are performed during ON periods.	☐
**4.5 COMPLEXITY OF MOTOR FLUCTUATIONS**<br><br><u>Instructions to examiner:</u> Determine the usual predictability of OFF function whether due to dose, time of day, food intake or other factors. Use the information provided by the patients and caregiver and supplement with your own observations. You will ask if the patient can count on them always coming at a special time, mostly coming at a special time (in which case you will probe further to separate slight from mild), only sometimes coming at a special time or are they totally unpredictable? Narrowing down the percentage will allow you to find the correct answer.<br><br>*<u>Instructions to patient [and caregiver]:</u> For some patients, the low or "OFF" periods happen at certain times during day or when they do activities like eating or exercising. Over the past week, do you usually know when your low periods will occur? In other words, do your low periods <u>always</u> come at a certain time? Do they <u>mostly</u> come at a certain time? Do they <u>only sometimes</u> come at a certain time? Are your low periods totally unpredictable?"*<br><br>0: Normal: No motor fluctuations.<br><br>1: Slight: OFF times are predictable all or almost all of the time (> 75%).<br><br>2: Mild: OFF times are predictable most of the time (51-75%).<br><br>3: Moderate: OFF times are predictable some of the time (26-50%).<br><br>4: Severe: OFF episodes are rarely predictable. (<u><</u> 25%).	☐

Copyright © 2008 Movement Disorder Society. All rights reserved.

This chart may not be copied, distributed or otherwise used in whole or in part without prior written consent of the Movement Disorder Society.

C. "OFF" DYSTONIA

4.6 PAINFUL OFF-STATE DYSTONIA

Instructions to examiner: For patients who have motor fluctuations, determine what proportion of the OFF episodes usually includes painful dystonia? You have already determined the number of hours of "OFF" time (4.3). Of these hours, determine how many are associated with dystonia and calculate the percentage. If there is no OFF time, mark 0.

Instructions to patient [and caregiver]: In one of the questions I asked earlier, you said you generally have ___ hours of low or "OFF" time when your Parkinson's disease is under poor control. During these low or "OFF" periods, do you usually have painful cramps or spasms? Out of the total ____ hrs of this low time, if you add up all the time in a day when these painful cramps come, how many hours would this make?

0: Normal: No dystonia OR NO OFF TIME.

1: Slight: < 25% of time in OFF state.

2: Mild: 26-50% of time in OFF state.

3: Moderate: 51-75% of time in OFF state.

4: Severe: > 75% of time in OFF state.

☐

1. Total Hours Off: _____
2. Total Off Hours w/Dystonia: _____
3. % Off Dystonia = ((2/1)*100): _____

Summary statement to patient: READ TO PATIENT

This completes my rating of your Parkinson's disease. I know the questions and tasks have taken several minutes, but I wanted to be complete and cover all possibilities. In doing so, I may have asked about problems you do not even have, and I may have mentioned problems that you may never develop at all. Not all patients develop all these problems, but because they can occur, it is important to ask all the questions to every patient. Thank you for your time and attention in completing this scale with me.

July 1, 2008 Copyright © 2008 Movement Disorder Society. All rights reserved. Page 30
This chart may not be copied, distributed or otherwise used in whole or in part without prior written consent of the Movement Disorder Society.

______________	__ __ __	__ __ - __ __ - __ __ __ __ (mm-dd-yyyy)	__ __ __
Patient Name or Subject ID	Site ID	Assessment Date	Investigator's Initials

MDS UPDRS Score Sheet

1.A	Source of information	☐ Patient ☐ Caregiver ☐ Patient + Caregiver	3.3b	Rigidity– RUE	
			3.3c	Rigidity– LUE	
**Part I**			3.3d	Rigidity– RLE	
1.1	Cognitive impairment		3.3e	Rigidity– LLE	
1.2	Hallucinations and psychosis		3.4a	Finger tapping– Right hand	
1.3	Depressed mood		3.4b	Finger tapping– Left hand	
1.4	Anxious mood		3.5a	Hand movements– Right hand	
1.5	Apathy		3.5b	Hand movements– Left hand	
1.6	Features of DDS		3.6a	Pronation- supination movements– Right hand	
1.6a	Who is filling out questionnaire	☐ Patient ☐ Caregiver ☐ Patient + Caregiver	3.6b	Pronation- supination movements– Left hand	
			3.7a	Toe tapping–Right foot	
1.7	Sleep problems		3.7b	Toe tapping– Left foot	
1.8	Daytime sleepiness		3.8a	Leg agility– Right leg	
1.9	Pain and other sensations		3.8b	Leg agility– Left leg	
1.10	Urinary problems		3.9	Arising from chair	
1.11	Constipation problems		3.10	Gait	
1.12	Light headedness on standing		3.11	Freezing of gait	
1.13	Fatigue		3.12	Postural stability	
**Part II**			3.13	Posture	
2.1	Speech		3.14	Global spontaneity of movement	
2.2	Saliva and drooling		3.15a	Postural tremor– Right hand	
2.3	Chewing and swallowing		3.15b	Postural tremor– Left hand	
2.4	Eating tasks		3.16a	Kinetic tremor– Right hand	
2.5	Dressing		3.16b	Kinetic tremor– Left hand	
2.6	Hygiene		3.17a	Rest tremor amplitude– RUE	
2.7	Handwriting		3.17b	Rest tremor amplitude– LUE	
2.8	Doing hobbies and other activities		3.17c	Rest tremor amplitude– RLE	
2.9	Turning in bed		3.17d	Rest tremor amplitude– LLE	
2.10	Tremor		3.17e	Rest tremor amplitude– Lip/jaw	
2.11	Getting out of bed		3.18	Constancy of rest	
2.12	Walking and balance			Were dyskinesias presen	☐ No ☐ Yes
2.13	Freezing			Did these movements interfere with ratings?	☐ No ☐ Yes
3a	Is the patient on medication?	☐ No ☐ Yes		Hoehn and Yahr Stage	
3b	Patient's clinical state	☐ Off ☐ On	**Part IV**		
3c	Is the patient on Levodopa?	☐ No ☐ Yes	4.1	Time spent with dyskinesias	
3.C1	If yes, minutes since last dose:		4.2	Functional impact of dyskinesias	
**Part III**			4.3	Time spent in the OFF state	
3.1	Speech		4.4	Functional impact of fluctuations	
3.2	Facial expression		4.5	Complexity of motor fluctuations	
3.3a	Rigidity– Neck		4.6	Painful OFF-state dystonia	

July 1, 2008

Copyright © 2008 Movement Disorder Society. All rights reserved.
This chart may not be copied, distributed or otherwise used in whole or in part without prior written consent of the Movement Disorder Society

APPENDIX E. PATIENT GLOBAL IMPRESSION OF CHANGE (PGI-C)

The subject will independently rate the following question of Patient Global Impression of Change (PGI-C) based on his/her overall impression at Visit 5 (Week 10), Visit 6 (Week 15), and Visit 7 (Week 20) or early discontinuation.

Patient Global Impression of Change:

Compared to your condition prior to your starting on this study, how much has your condition changed with your current treatment?

❑ 1	❑ 2	❑ 3	❑ 4	❑ 5	❑ 6	❑ 7
Very Much Worse	Much Worse	Minimally Worse	No Change	Minimally Improved	Much Improved	Very Much Improved

APPENDIX F. CLINICAL GLOBAL IMPRESSION OF CHANGE (CGI-C)

The Investigator rates each subject with the following question as part of Visit 5 (Week 10), Visit 6 (Week 15), and Visit 7 (Week 20) or early discontinuation:

Clinical Global Impression of Change:

In your opinion, how much has the subject's overall condition and Parkinson's disease symptoms changed since starting on the study?

❑ 1	❑ 2	❑ 3	❑ 4	❑ 5	❑ 6	❑ 7
Very Much Worse	Much Worse	Minimally Worse	Neutral	Minimally Improved	Much Improved	Very Much Improved

Guy W. ECDEU assessment manual for psychopharmacology publication; ADM, 76-338.

Washington DC, US. Department of health, education and welfare, 1976.

APPENDIX G. PATIENT GLOBAL IMPRESSION OF SEVERITY (PGI-S)

Patient Global Impression – Severity Scale

Severity of Illness

Considering the severity of your Parkinson's disease, how severe is your condition at this time?

Severity Score:

❑ 1	❑ 2	❑ 3	❑ 4	❑ 5	❑ 6	❑ 7
Normal, not at all ill	Borderline ill	Mildly ill	Moderately ill	Markedly ill	Severely ill	Extremely severely ill

APPENDIX H. CLINICAL GLOBAL IMPRESSION OF SEVERITY (CGI-S)

The Investigator will independently rate the following question of Clinical Global Impression of Severity (CGI-S) based on his/her overall impression of the study medication at Visit 1, Visit 4, and Visit 7 or early discontinuation.

Clinical Global Impression – Severity Scale

Severity of Illness

Considering your total clinical experience with this particular PD population, how ill is the patient at this time?

Severity Score:

❑ 1	❑ 2	❑ 3	❑ 4	❑ 5	❑ 6	❑ 7
Normal, not at all ill	Borderline ill	Mildly ill	Moderately ill	Markedly ill	Severely ill	Among the most extremely ill of subjects

Guy W. ECDEU assessment manual for psychopharmacology publication; ADM, 76-338.

Washington DC, US. Department of health, education and welfare, 1976.

APPENDIX I. 39-ITEM PARKINSON'S DISEASE QUESTIONNAIRE (PDQ-39)

PDQ-39 QUESTIONNAIRE

Please complete the following

Please tick one box for each question

Due to having Parkinson's disease, how often during the last month have you....		Never	Occasionally	Sometimes	Often	Always or cannot do at all
1	Had difficulty doing the leisure activities which you would like to do?	☐	☐	☐	☐	☐
2	Had difficulty looking after your home, e.g. DIY, housework, cooking?	☐	☐	☐	☐	☐
3	Had difficulty carrying bags of shopping?	☐	☐	☐	☐	☐
4	Had problems walking half a mile?	☐	☐	☐	☐	☐
5	Had problems walking 100 yards?	☐	☐	☐	☐	☐
6	Had problems getting around the house as easily as you would like?	☐	☐	☐	☐	☐
7	Had difficulty getting around in public?	☐	☐	☐	☐	☐
8	Needed someone else to accompany you when you went out?	☐	☐	☐	☐	☐
9	Felt frightened or worried about falling over in public?	☐	☐	☐	☐	☐
10	Been confined to the house more than you would like?	☐	☐	☐	☐	☐
11	Had difficulty washing yourself?	☐	☐	☐	☐	☐
12	Had difficulty dressing yourself?	☐	☐	☐	☐	☐
13	Had problems doing up your shoe laces?	☐	☐	☐	☐	☐

Please check that you have ticked ***one box for each question*** *before going on to the next page*

Please tick one box for each question

Due to having Parkinson's disease, how often during the last month have you....		Never	Occasionally	Sometimes	Often	Always or cannot do at all
14	Had problems writing clearly?	☐	☐	☐	☐	☐
15	Had difficulty cutting up your food?	☐	☐	☐	☐	☐
16	Had difficulty holding a drink without spilling it?	☐	☐	☐	☐	☐
17	Felt depressed?	☐	☐	☐	☐	☐
18	Felt isolated and lonely?	☐	☐	☐	☐	☐
19	Felt weepy or tearful?	☐	☐	☐	☐	☐
20	Felt angry or bitter?	☐	☐	☐	☐	☐
21	Felt anxious?	☐	☐	☐	☐	☐
22	Felt worried about your future?	☐	☐	☐	☐	☐
23	Felt you had to conceal your Parkinson's from people?	☐	☐	☐	☐	☐
24	Avoided situations which involve eating or drinking in public?	☐	☐	☐	☐	☐
25	Felt embarrassed in public due to having Parkinson's disease?	☐	☐	☐	☐	☐
26	Felt worried by other people's reaction to you?	☐	☐	☐	☐	☐
27	Had problems with your close personal relationships?	☐	☐	☐	☐	☐
28	Lacked support in the ways you need from your spouse or partner?	☐	☐	☐	☐	☐
	If you do not have a spouse or partner tick here		☐			
29	Lacked support in the ways you need from your family or close friends?	☐	☐	☐	☐	☐

Please check that you have ticked ***one box for each question*** *before going on to the next page*

Due to having Parkinson's disease, how often during the last month have you....	***Please tick one box for each question*** Never	Occasionally	Sometimes	Often	Always
30 Unexpectedly fallen asleep during the day?	☐	☐	☐	☐	☐
31 Had problems with your concentration, e.g. when reading or watching TV?	☐	☐	☐	☐	☐
32 Felt your memory was bad?	☐	☐	☐	☐	☐
33 Had distressing dreams or hallucinations?	☐	☐	☐	☐	☐
34 Had difficulty with your speech?	☐	☐	☐	☐	☐
35 Felt unable to communicate with people properly?	☐	☐	☐	☐	☐
36 Felt ignored by people?	☐	☐	☐	☐	☐
37 Had painful muscle cramps or spasms?	☐	☐	☐	☐	☐
38 Had aches and pains in your joints or body?	☐	☐	☐	☐	☐
39 Felt unpleasantly hot or cold?	☐	☐	☐	☐	☐

*Please check that you have ticked **one box for each question** before going on to the next page*

Thank you for completing the PDQ 39 questionnaire

APPENDIX J. GASTROPARESIS CARDINAL SYMPTOM INDEX (GCSI)

GASTROPARESIS CARDINAL SYMPTOM INDEX

This questionnaire asks you about the severity of symptoms you may have related to your gastrointestinal problem. There are no right or wrong answers. Please answer each question as accurately as possible.

For each symptom, please circle the number that best describes how severe the symptom has been during the past 2 weeks.

- If you have not experienced this symptom, circle 0.
- If the symptom has been very mild, circle 1.
- If the symptom has been mild, circle 2.
- If it has been moderate, circle 3.
- If it has been severe, circle 4.
- If it has been very severe, circle 5.

Please be sure to answer every question.

Please rate the severity of the following symptoms during the past 2 weeks.

		None	Very mild	Mild	Moderate	Severe	Very severe
1.	nausea (feeling sick to your stomach as if you were going to vomit or throw up)	0	1	2	3	4	5
2.	retching (heaving as if to vomit, but nothing comes up)	0	1	2	3	4	5
3.	vomiting	0	1	2	3	4	5
4.	stomach fullness	0	1	2	3	4	5
5.	not able to finish a normal-sized meal	0	1	2	3	4	5
6.	feeling excessively full after meals	0	1	2	3	4	5
7.	loss of appetite	0	1	2	3	4	5
8.	bloating (feeling like you need to loosen your clothes)	0	1	2	3	4	5
9.	stomach or belly visibly larger	0	1	2	3	4	5

©2002 Johnson & Johnson Pharmaceutical Services, LLC. Contact D. Dubois for permission to use.

APPENDIX K. NON-MOTOR SYMPTOM ASSESSMENT SCALE FOR PARKINSON'S DISEASE (NMSS)

Non-Motor Symptom assessment scale for Parkinson's Disease

Patient ID No: ______________________ **Initials:** _______________ **Age:** _________

Symptoms assessed over the last month. Each symptom scored with respect to:

Severity: 0 = None, 1 = Mild: symptoms present but causes little distress or disturbance to patient; 2 = Moderate: some distress or disturbance to patient; 3 = Severe: major source of distress or disturbance to patient.

Frequency: 1 = Rarely (<1/wk); 2 = Often (1/wk); 3 = Frequent (several times per week); 4 = Very Frequent (daily or all the time)

Domains will be weighed differentially. Yes/ No answers are not included in final frequency x severity calculation.
(Bracketed text in questions within the scale is included as an explanatory aid).

**Domain 1: Cardiovascular including falls**	Severity	Frequency	Frequency x Severity
1. Does the patient experience light-headedness, dizziness, weakness on standing from sitting or lying position?	☐	☐	☐
2. Does the patient fall because of fainting or blacking out?	☐	☐	☐
**SCORE:**			☐

**Domain 2: Sleep/fatigue**			
3. Does the patient doze off or fall asleep unintentionally during daytime activities? (For example, during conversation, during mealtimes, or while watching television or reding).	☐	☐	☐
4. Does fatigue (tiredness) or lack of energy (not slowness) limit the patient's daytime activities?	☐	☐	☐
5. Does the patient have difficulties falling or staying asleep?	☐	☐	☐
6. Does the patient experience an urge to move the legs or restlessness in legs that improves with movement when he/she is sitting or lying down inactive?	☐	☐	☐
**SCORE:**			☐

**Domain 3: Mood /Cognition**			
7. Has the patient lost interest in his/her surroundings?	☐	☐	☐
8. Has the patient lost interest in doing things or lack motivation to start new activities?	☐	☐	☐
9. Does the patient feel nervous, worried or frightened for no apparent reason?	☐	☐	☐
10. Does the patient seem sad or depressed or has he/she reported such feelings?	☐	☐	☐
11. Does the patient have flat moods without the normal "highs" and " lows"?	☐	☐	☐
12. Does the patient have difficulty in experiencing pleasure from their usual activities or report that they lack pleasure?	☐	☐	☐
**SCORE:**			☐

**Domain 4: Perceptual problems/hallucinations**			
13. Does the patient indicate that he/she sees things that are not there?	☐	☐	☐
14. Does the patient have beliefs that you know are not true? (For example, about being harmed, being robbed or being unfaithful)	☐	☐	☐
15. Does the patient experience double vision? (2 separate real objects and not blurred vision)	☐	☐	☐
**SCORE:**			☐

	Severity	Frequency	Frequency x Severity
**Domain 5: Attention/ Memory**			
16. Does the patient have problems sustaining concentration during activities? (For example, reading or having a conversation)	☐	☐	☐
17. Does the patient forget things that he/she has been told a short time ago or events that happened in the last few days?	☐	☐	☐
18. Does the patient forget to do things? (For example, take tablets or turn off domestic appliances?)	☐	☐	☐
**SCORE:**			☐
**Domain 6: Gastrointestinal tract**			
19. Does the patient dribble saliva during the day?	☐	☐	☐
20. Does the patient having difficulty swallowing?	☐	☐	☐
21. Does the patient suffer from constipation? (Bowel action less than three times weekly)	☐	☐	☐
**SCORE:**			☐
**Domain 7: Urinary**			
22. Does the patient have difficulty holding urine? (Urgency)	☐	☐	☐
23. Does the patient have to void within 2 hours of last voiding? (Frequency)	☐	☐	☐
24. Does the patient have to get up regularly at night to pass urine? (Nocturia)	☐	☐	☐
**SCORE:**			☐
**Domain 8: Sexual function**			
25. Does the patient have altered interest in sex? (Very much increased or decreased, please underline)	☐	☐	☐
26. Does the patient have problems having sex?	☐	☐	☐
**SCORE:**			☐
**Domain 9: Miscellaneous**			
27. Does the patient suffer from pain not explained by other known conditions? (Is it related to intake of drugs and is it relieved by antiparkinson drugs?)	☐	☐	☐
28. Does the patient report a change in ability to taste or smell?	☐	☐	☐
29. Does the patient report a recent change in weight (not related to dieting)?	☐	☐	☐
30. Does the patient experience excessive sweating? (not related to hot weather)	☐	☐	☐
**SCORE:**			☐
**TOTAL SCORE:**			☐

Developed by the International Parkinson's Disease Non- Motor Group.
Contacts: ray.chaudhuri@uhl.nhs.uk or alison.forbes@uhl.nhs.uk

APPENDIX L. PARKINSON'S DISEASE SLEEP SCALE-2 (PDSS-2)

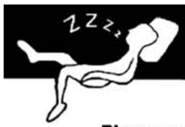

Parkinson's Disease Sleep Scale (PDSS-2)

Please rate the severity of the following based on your experiences during the past week (7 days). Please make a cross in the answer box

	Very often (This means 6 to 7 days a week)	Often (This means 4 to 5 days a week)	Sometimes (This means 2 to 3 days a week)	Occasionally (This means 1 day a week)	Never
1) Overall, did you sleep well during the last week?	☐ 0	☐ 1	☐ 2	☐ 3	☐ 4
2) Did you have difficulty falling asleep each night?	☐ 4	☐ 3	☐ 2	☐ 1	☐ 0
3) Did you have difficulty staying asleep?	☐ 4	☐ 3	☐ 2	☐ 1	☐ 0
4) Did you have restlessness of legs or arms at nights causing disruption of sleep?	☐ 4	☐ 3	☐ 2	☐ 1	☐ 0
5) Was your sleep disturbed due to an urge to move your legs or arms?	☐ 4	☐ 3	☐ 2	☐ 1	☐ 0
6) Did you suffer from distressing dreams at night?	☐ 4	☐ 3	☐ 2	☐ 1	☐ 0
7) Did you suffer from distressing hallucinations at night (seeing or hearing things that you are told do not exist)?	☐ 4	☐ 3	☐ 2	☐ 1	☐ 0
8) Did you get up at night to pass urine?	☐ 4	☐ 3	☐ 2	☐ 1	☐ 0
9) Did you feel uncomfortable at night because you were unable to turn around in bed or move due to immobility?	☐ 4	☐ 3	☐ 2	☐ 1	☐ 0
10) Did you feel pain in your arms or legs which woke you up from sleep at night?	☐ 4	☐ 3	☐ 2	☐ 1	☐ 0
11) Did you have muscle cramps in your arms or legs which woke you up whilst sleeping at night?	☐ 4	☐ 3	☐ 2	☐ 1	☐ 0
12) Did you wake early in the morning with painful posturing of arms and legs?	☐ 4	☐ 3	☐ 2	☐ 1	☐ 0
13) On waking, did you experience tremor?	☐ 4	☐ 3	☐ 2	☐ 1	☐ 0
14) Did you feel tired and sleepy after waking in the morning?	☐ 4	☐ 3	☐ 2	☐ 1	☐ 0
15) Did you wake up at night due to snoring or difficulties with breathing?	☐ 4	☐ 3	☐ 2	☐ 1	☐ 0

Trenkwalder C, Kohnen R, Högl B, Metta V, Sixel-Döring F, Frauscher B, Hülsmann J, Martinez-Martin P, Chaudhuri KR. Parkinson's disease sleep scale--validation of the revised version PDSS-2. Mov Disord. 2011;26(4):644-52.

APPENDIX M. PARKINSON ANXIETY SCALE (PAS)

The Parkinson Anxiety Scale (PAS)

(Please mark one circle for each item below)

In the past four weeks, to what extent did you experience the following symptoms?

A. Persistent Anxiety

A.1. Feeling anxious or nervous

- o Not at all, or never
- o Very mild, or rarely
- o Mild, or sometimes
- o Moderate, or often
- o Severe, or (nearly) always

A.2. Feeling tense or stressed

- o Not at all, or never
- o Very mild, or rarely
- o Mild, or sometimes
- o Moderate, or often
- o Severe, or (nearly) always

A.3. Being unable to relax

- o Not at all, or never
- o Very mild, or rarely
- o Mild, or sometimes
- o Moderate, or often
- o Severe, or (nearly) always

A.4. Excessive worrying about everyday matters

- o Not at all, or never
- o Very mild, or rarely
- o Mild, or sometimes
- o Moderate, or often
- o Severe, or (nearly) always

A.5. Fear of something bad, or even the worst, happening

- o Not at all, or never
- o Very mild, or rarely
- o Mild, or sometimes
- o Moderate, or often
- o Severe, or (nearly) always

B. Episodic Anxiety

B.1. Panic or intense fear

- Never
- Rarely
- Sometimes
- Often
- Nearly always

B.2. Shortness of breath

- Never
- Rarely
- Sometimes
- Often
- Nearly always

B.3. Heart palpitations or heart beating fast (not related to physical effort or activity)

- Never
- Rarely
- Sometimes
- Often
- Nearly always

B.4. Fear of losing control

- Never
- Rarely
- Sometimes
- Often
- Nearly always

C. Avoidance Behavior

C.1. Social situations (where one may be observed, or evaluated by others, such as speaking in public, or talking to unknown people)

- Never
- Rarely
- Sometimes
- Often
- Nearly always

C.2. Public settings (situations from which it may be difficult or embarrassing to escape, such as queues or lines, crowds, bridges, or public transportation)

- Never
- Rarely
- Sometimes
- Often
- Nearly always

C.3. Specific objects or situations (such as flying, heights, spiders or other animals, needles, or blood)

- Never
- Rarely
- Sometimes
- Often
- Nearly always

Copyright of this scale and it translations is held by the authors (Leentjens AF, Dujardin K, Pontone GM, Starkstein SE, Weintraub D, and Martinez-Martin P. The Parkinson Anxiety Scale (PAS): development and validation of a new anxiety scale. Mov Disord. 2014;29(8):1035-43). The scale and its translations are in the public domain and may be used without additional permission and free of charge on the condition that its source is referenced.

APPENDIX N. COLUMBIA-SUICIDE SEVERITY RATING SCALE (C-SSRS)

COLUMBIA-SUICIDE SEVERITY RATING SCALE (C-SSRS)

Baseline/Screening Version

Version 1/14/09

Posner, K.; Brent, D.; Lucas, C.; Gould, M.; Stanley, B.; Brown, G.; Fisher, P.; Zelazny, J.; Burke, A.; Oquendo, M.; Mann, J.

Disclaimer:

This scale is intended to be used by individuals who have received training in its administration. The questions contained in the Columbia-Suicide Severity Rating Scale are suggested probes. Ultimately, the determination of the presence of suicidal ideation or behavior depends on the judgment of the individual administering the scale.

Definitions of behavioral suicidal events in this scale are based on those used in **The Columbia Suicide History Form**, *developed by John Mann, MD and Maria Oquendo, MD, Conte Center for the Neuroscience of Mental Disorders (CCNMD), New York State Psychiatric Institute, 1051 Riverside Drive, New York, NY, 10032. (Oquendo M. A., Halberstam B. & Mann J. J., Risk factors for suicidal behavior: utility and limitations of research instruments. In M.B. First [Ed.]* Standardized Evaluation in Clinical Practice, *pp. 103 -130, 2003.)*

For reprints of the C-SSRS contact Kelly Posner, Ph.D., New York State Psychiatric Institute, 1051 Riverside Drive, New York, New York, 10032; inquiries and training requirements contact posnerk@nyspi.columbia.edu

© 2008 The Research Foundation for Mental Hygiene, Inc.

SUICIDAL IDEATION		
Ask questions 1 and 2. If both are negative, proceed to "Suicidal Behavior" section. If the answer to question 2 is "yes", ask questions 3, 4 and 5. If the answer to question 1 and/or 2 is "yes", complete "Intensity of Ideation" section below.	**Lifetime: Time He/She Felt Most Suicidal**	**Past __ Months**
**1. Wish to be Dead**<br>Subject endorses thoughts about a wish to be dead or not alive anymore, or wish to fall asleep and not wake up.<br>***Have you wished you were dead or wished you could go to sleep and not wake up?***<br><br>If yes, describe:	**Yes** ☐ **No** ☐	**Yes** ☐ **No** ☐
**2. Non-Specific Active Suicidal Thoughts**<br>General non-specific thoughts of wanting to end one's life/commit suicide (e.g., *"I've thought about killing myself"*) without thoughts of ways to kill oneself/associated methods, intent, or plan during the assessment period.<br>***Have you actually had any thoughts of killing yourself?***<br><br>If yes, describe:	**Yes** ☐ **No** ☐	**Yes** ☐ **No** ☐
**3. Active Suicidal Ideation with Any Methods (Not Plan) without Intent to Act**<br>Subject endorses thoughts of suicide and has thought of at least one method during the assessment period. This is different than a specific plan with time, place or method details worked out (e.g. thought of method to kill self but not a specific plan). Includes person who would say, *"I thought about taking an overdose but I never made a specific plan as to when, where or how I would actually do it…and I would never go through with it."*<br>***Have you been thinking about how you might do this?***<br><br>If yes, describe:	**Yes** ☐ **No** ☐	**Yes** ☐ **No** ☐
**4. Active Suicidal Ideation with Some Intent to Act, without Specific Plan**<br>Active suicidal thoughts of killing oneself and subject reports having some intent to act on such thoughts, as opposed to *"I have the thoughts but I definitely will not do anything about them."*<br>***Have you had these thoughts and had some intention of acting on them?***<br><br>If yes, describe:	**Yes** ☐ **No** ☐	**Yes** ☐ **No** ☐
**5. Active Suicidal Ideation with Specific Plan and Intent**<br>Thoughts of killing oneself with details of plan fully or partially worked out and subject has some intent to carry it out.<br>***Have you started to work out or worked out the details of how to kill yourself? Do you intend to carry out this plan?***<br><br>If yes, describe:	**Yes** ☐ **No** ☐	**Yes** ☐ **No** ☐
INTENSITY OF IDEATION		
The following features should be rated with respect to the most severe type of ideation (i.e., 1-5 from above, with 1 being the least severe and 5 being the most severe). Ask about time he/she was feeling the most suicidal.<br><br>Lifetime - ***Most Severe Ideation:*** ____ (*Type # (1-5)*) ____________ (*Description of Ideation*)<br><br>Past X Months - ***Most Severe Ideation:*** ____ (*Type # (1-5)*) ____________ (*Description of Ideation*)	Most Severe	Most Severe
**Frequency**<br>***How many times have you had these thoughts?***<br>(1) Less than once a week (2) Once a week (3) 2-5 times in week (4) Daily or almost daily (5) Many times each day	___	___
**Duration**<br>***When you have the thoughts how long do they last?***<br>(1) Fleeting - few seconds or minutes<br>(2) Less than 1 hour/some of the time<br>(3) 1-4 hours/a lot of time<br>(4) 4-8 hours/most of day<br>(5) More than 8 hours/persistent or continuous	___	___
**Controllability**<br>***Could/can you stop thinking about killing yourself or wanting to die if you want to?***<br>(1) Easily able to control thoughts<br>(2) Can control thoughts with little difficulty<br>(3) Can control thoughts with some difficulty<br>(4) Can control thoughts with a lot of difficulty<br>(5) Unable to control thoughts<br>(0) Does not attempt to control thoughts	___	___
**Deterrents**<br>***Are there things - anyone or anything (e.g., family, religion, pain of death) - that stopped you from wanting to die or acting on thoughts of committing suicide?***<br>(1) Deterrents definitely stopped you from attempting suicide<br>(2) Deterrents probably stopped you<br>(3) Uncertain that deterrents stopped you<br>(4) Deterrents most likely did not stop you<br>(5) Deterrents definitely did not stop you<br>(0) Does not apply	___	___
**Reasons for Ideation**<br>***What sort of reasons did you have for thinking about wanting to die or killing yourself? Was it to end the pain or stop the way you were feeling (in other words you couldn't go on living with this pain or how you were feeling) or was it to get attention, revenge or a reaction from others? Or both?***<br>(1) Completely to get attention, revenge or a reaction from others<br>(2) Mostly to get attention, revenge or a reaction from others<br>(3) Equally to get attention, revenge or a reaction from others and to end/stop the pain<br>(4) Mostly to end or stop the pain (you couldn't go on living with the pain or how you were feeling)<br>(5) Completely to end or stop the pain (you couldn't go on living with the pain or how you were feeling)<br>(0) Does not apply	___	___

© 2008 Research Foundation for Mental Hygiene, Inc. C-SSRS—Baseline/Screening (Version 1/14/09) Page 1 of 2

SUICIDAL BEHAVIOR<br>*(Check all that apply, so long as these are separate events; must ask about all types)*	**Lifetime**	**Past __ Years**
**Actual Attempt:**<br>A potentially self-injurious act committed with at least some wish to die, *as a result of act.* Behavior was in part thought of as method to kill oneself. Intent does not have to be 100%. If there is ***any*** intent/desire to die associated with the act, then it can be considered an actual suicide attempt. ***There does not have to be any injury or harm,*** just the potential for injury or harm. If person pulls trigger while gun is in mouth but gun is broken so no injury results, this is considered an attempt.<br>Inferring Intent: Even if an individual denies intent/wish to die, it may be inferred clinically from the behavior or circumstances. For example, a highly lethal act that is clearly not an accident so no other intent but suicide can be inferred (e.g., gunshot to head, jumping from window of a high floor/story). Also, if someone denies intent to die, but they thought that what they did could be lethal, intent may be inferred.<br>***Have you made a suicide attempt?***<br>***Have you done anything to harm yourself?***<br>***Have you done anything dangerous where you could have died?***<br>***What did you do?***<br>***Did you______ as a way to end your life?***<br>***Did you want to die (even a little) when you_____?***<br>***Were you trying to end your life when you _____?***<br>***Or Did you think it was possible you could have died from_____?***<br>***Or did you do it purely for other reasons / without ANY intention of killing yourself (like to relieve stress, feel better, get sympathy, or get something else to happen)?*** (Self-Injurious Behavior without suicidal intent)<br>If yes, describe:	Yes No<br>☐ ☐<br><br>Total # of Attempts<br>_____	Yes No<br>☐ ☐<br><br>Total # of Attempts<br>_____
**Has subject engaged in Non-Suicidal Self-Injurious Behavior?**	Yes No<br>☐ ☐	Yes No<br>☐ ☐
**Interrupted Attempt:**<br>When the person is interrupted (by an outside circumstance) from starting the potentially self-injurious act *(if not for that, actual attempt would have occurred).*<br>Overdose: Person has pills in hand but is stopped from ingesting. Once they ingest any pills, this becomes an attempt rather than an interrupted attempt. Shooting: Person has gun pointed toward self, gun is taken away by someone else, or is somehow prevented from pulling trigger. Once they pull the trigger, even if the gun fails to fire, it is an attempt. Jumping: Person is poised to jump, is grabbed and taken down from ledge. Hanging: Person has noose around neck but has not yet started to hang - is stopped from doing so.<br>***Has there been a time when you started to do something to end your life but someone or something stopped you before you actually did anything?***<br>If yes, describe:	Yes No<br>☐ ☐<br><br>Total # of interrupted<br>_____	Yes No<br>☐ ☐<br><br>Total # of interrupted<br>_____
**Aborted Attempt:**<br>When person begins to take steps toward making a suicide attempt, but stops themselves before they actually have engaged in any self-destructive behavior. Examples are similar to interrupted attempts, except that the individual stops him/herself, instead of being stopped by something else.<br>***Has there been a time when you started to do something to try to end your life but you stopped yourself before you actually did anything?***<br>If yes, describe:	Yes No<br>☐ ☐<br><br>Total # of aborted<br>_____	Yes No<br>☐ ☐<br><br>Total # of aborted<br>_____
**Preparatory Acts or Behavior:**<br>Acts or preparation towards imminently making a suicide attempt. This can include anything beyond a verbalization or thought, such as assembling a specific method (e.g., buying pills, purchasing a gun) or preparing for one's death by suicide (e.g., giving things away, writing a suicide note).<br>***Have you taken any steps towards making a suicide attempt or preparing to kill yourself (such as collecting pills, getting a gun, giving valuables away or writing a suicide note)?***<br>If yes, describe:	Yes No<br>☐ ☐	Yes No<br>☐ ☐
**Suicidal Behavior:**<br>Suicidal behavior was present during the assessment period?	Yes No<br>☐ ☐	Yes No<br>☐ ☐

Answer for Actual Attempts Only	Most Recent Attempt<br>Date:	Most Lethal Attempt<br>Date:	Initial/First Attempt<br>Date:
**Actual Lethality/Medical Damage:**<br>0. No physical damage or very minor physical damage (e.g., surface scratches).<br>1. Minor physical damage (e.g., lethargic speech; first-degree burns; mild bleeding; sprains).<br>2. Moderate physical damage; medical attention needed (e.g., conscious but sleepy, somewhat responsive; second-degree burns; bleeding of major vessel).<br>3. Moderately severe physical damage; *medical* hospitalization and likely intensive care required (e.g., comatose with reflexes intact; third-degree burns less than 20% of body; extensive blood loss but can recover; major fractures).<br>4. Severe physical damage; *medical* hospitalization with intensive care required (e.g., comatose without reflexes; third-degree burns over 20% of body; extensive blood loss with unstable vital signs; major damage to a vital area).<br>5. Death	*Enter Code*<br>_____	*Enter Code*<br>_____	*Enter Code*<br>_____
**Potential Lethality: Only Answer if Actual Lethality=0**<br>Likely lethality of actual attempt if no medical damage (the following examples, while having no actual medical damage, had potential for very serious lethality: put gun in mouth and pulled the trigger but gun fails to fire so no medical damage; laying on train tracks with oncoming train but pulled away before run over).<br>0 = Behavior not likely to result in injury<br>1 = Behavior likely to result in injury but not likely to cause death<br>2 = Behavior likely to result in death despite available medical care	*Enter Code*<br>_____	*Enter Code*<br>_____	*Enter Code*<br>_____

© 2008 Research Foundation for Mental Hygiene, Inc. C-SSRS—Baseline/Screening (Version 1/14/09) Page 2 of 2

COLUMBIA-SUICIDE SEVERITY RATING SCALE (C-SSRS)

Since Last Visit

Version 1/14/09

Posner, K.; Brent, D.; Lucas, C.; Gould, M.; Stanley, B.; Brown, G.; Fisher, P.; Zelazny, J.; Burke, A.; Oquendo, M.; Mann, J.

Disclaimer:

This scale is intended to be used by individuals who have received training in its administration. The questions contained in the Columbia-Suicide Severity Rating Scale are suggested probes. Ultimately, the determination of the presence of suicidal ideation or behavior depends on the judgment of the individual administering the scale.

Definitions of behavioral suicidal events in this scale are based on those used in **The Columbia Suicide History Form**, *developed by John Mann, MD and Maria Oquendo, MD, Conte Center for the Neuroscience of Mental Disorders (CCNMD), New York State Psychiatric Institute, 1051 Riverside Drive, New York, NY, 10032. (Oquendo M. A., Halberstam B. & Mann J. J., Risk factors for suicidal behavior: utility and limitations of research instruments. In M.B. First [Ed.]* Standardized Evaluation in Clinical Practice, *pp. 103 -130, 2003.)*

For reprints of the C-SSRS contact Kelly Posner, Ph.D., New York State Psychiatric Institute, 1051 Riverside Drive, New York, New York, 10032; inquiries and training requirements contact posnerk@nyspi.columbia.edu

© 2008 The Research Foundation for Mental Hygiene, Inc.

SUICIDAL IDEATION	
Ask questions 1 and 2. If both are negative, proceed to "Suicidal Behavior" section. If the answer to question 2 is "yes", ask questions 3, 4 and 5. If the answer to question 1 and/or 2 is "yes", complete "Intensity of Ideation" section below.	**Since Last Visit**
**1. Wish to be Dead**<br>Subject endorses thoughts about a wish to be dead or not alive anymore, or wish to fall asleep and not wake up.<br>***Have you wished you were dead or wished you could go to sleep and not wake up?***<br><br>If yes, describe:	**Yes** ☐ **No** ☐
**2. Non-Specific Active Suicidal Thoughts**<br>General, non-specific thoughts of wanting to end one's life/commit suicide (e.g., *"I've thought about killing myself"*) without thoughts of ways to kill oneself/associated methods, intent, or plan during the assessment period.<br>***Have you actually had any thoughts of killing yourself?***<br><br>If yes, describe:	**Yes** ☐ **No** ☐
**3. Active Suicidal Ideation with Any Methods (Not Plan) without Intent to Act**<br>Subject endorses thoughts of suicide and has thought of at least one method during the assessment period. This is different than a specific plan with time, place or method details worked out (e.g., thought of method to kill self but not a specific plan). Includes person who would say, *"I thought about taking an overdose but I never made a specific plan as to when, where or how I would actually do it…and I would never go through with it."*<br>***Have you been thinking about how you might do this?***<br><br>If yes, describe:	**Yes** ☐ **No** ☐
**4. Active Suicidal Ideation with Some Intent to Act, without Specific Plan**<br>Active suicidal thoughts of killing oneself and subject reports having some intent to act on such thoughts, as opposed to *"I have the thoughts but I definitely will not do anything about them."*<br>***Have you had these thoughts and had some intention of acting on them?***<br><br>If yes, describe:	**Yes** ☐ **No** ☐
**5. Active Suicidal Ideation with Specific Plan and Intent**<br>Thoughts of killing oneself with details of plan fully or partially worked out and subject has some intent to carry it out.<br>***Have you started to work out or worked out the details of how to kill yourself? Do you intend to carry out this plan?***<br><br>If yes, describe:	**Yes** ☐ **No** ☐

INTENSITY OF IDEATION	
The following features should be rated with respect to the most severe type of ideation (i.e., 1-5 from above, with 1 being the least severe and 5 being the most severe).<br><br>***Most Severe Ideation:*** ____ ________________<br>***Type # (1-5)*** ***Description of Ideation***	Most Severe
**Frequency**<br>***How many times have you had these thoughts?***<br>(1) Less than once a week (2) Once a week (3) 2-5 times in week (4) Daily or almost daily (5) Many times each day	___
**Duration**<br>***When you have the thoughts, how long do they last?***<br>(1) Fleeting - few seconds or minutes<br>(2) Less than 1 hour/some of the time<br>(3) 1-4 hours/a lot of time<br>(4) 4-8 hours/most of day<br>(5) More than 8 hours/persistent or continuous	___
**Controllability**<br>***Could/can you stop thinking about killing yourself or wanting to die if you want to?***<br>(1) Easily able to control thoughts<br>(2) Can control thoughts with little difficulty<br>(3) Can control thoughts with some difficulty<br>(4) Can control thoughts with a lot of difficulty<br>(5) Unable to control thoughts<br>(0) Does not attempt to control thoughts	___
**Deterrents**<br>***Are there things - anyone or anything (e.g., family, religion, pain of death) - that stopped you from wanting to die or acting on thoughts of committing suicide?***<br>(1) Deterrents definitely stopped you from attempting suicide<br>(2) Deterrents probably stopped you<br>(3) Uncertain that deterrents stopped you<br>(4) Deterrents most likely did not stop you<br>(5) Deterrents definitely did not stop you<br>(0) Does not apply	___
**Reasons for Ideation**<br>***What sort of reasons did you have for thinking about wanting to die or killing yourself? Was it to end the pain or stop the way you were feeling (in other words you couldn't go on living with this pain or how you were feeling) or was it to get attention, revenge or a reaction from others? Or both?***<br>(1) Completely to get attention, revenge or a reaction from others<br>(2) Mostly to get attention, revenge or a reaction from others<br>(3) Equally to get attention, revenge or a reaction from others and to end/stop the pain<br>(4) Mostly to end or stop the pain (you couldn't go on living with the pain or how you were feeling)<br>(5) Completely to end or stop the pain (you couldn't go on living with the pain or how you were feeling)<br>(0) Does not apply	___

© 2008 Research Foundation for Mental Hygiene, Inc. C-SSRS—Since Last Visit (Version 1/14/09) Page 1 of 2

SUICIDAL BEHAVIOR *(Check all that apply, so long as these are separate events; must ask about all types)*	**Since Last Visit**
**Actual Attempt:** A potentially self-injurious act committed with at least some wish to die, *as a result of act.* Behavior was in part thought of as method to kill oneself. Intent does not have to be 100%. If there is ***any*** intent/desire to die associated with the act, then it can be considered an actual suicide attempt. ***There does not have to be any injury or harm,*** just the potential for injury or harm. If person pulls trigger while gun is in mouth but gun is broken so no injury results, this is considered an attempt.<br>Inferring Intent: Even if an individual denies intent/wish to die, it may be inferred clinically from the behavior or circumstances. For example, a highly lethal act that is clearly not an accident so no other intent but suicide can be inferred (e.g., gunshot to head, jumping from window of a high floor/story). Also, if someone denies intent to die, but they thought that what they did could be lethal, intent may be inferred.<br>***Have you made a suicide attempt?***<br>***Have you done anything to harm yourself?***<br>***Have you done anything dangerous where you could have died?***<br>***What did you do?***<br>***Did you______ as a way to end your life?***<br>***Did you want to die (even a little) when you_____?***<br>***Were you trying to end your life when you _____?***<br>***Or did you think it was possible you could have died from_____?***<br>***Or did you do it purely for other reasons / without ANY intention of killing yourself (like to relieve stress, feel better, get sympathy, or get something else to happen)?*** (Self-Injurious Behavior without suicidal intent)<br>If yes, describe:	**Yes** ☐ **No** ☐<br>Total # of Attempts<br>____
**Has subject engaged in Non-Suicidal Self-Injurious Behavior?**	**Yes** ☐ **No** ☐
**Interrupted Attempt:** When the person is interrupted (by an outside circumstance) from starting the potentially self-injurious act *(if not for that, actual attempt would have occurred).*<br>Overdose: Person has pills in hand but is stopped from ingesting. Once they ingest any pills, this becomes an attempt rather than an interrupted attempt. Shooting: Person has gun pointed toward self, gun is taken away by someone else, or is somehow prevented from pulling trigger. Once they pull the trigger, even if the gun fails to fire, it is an attempt. Jumping: Person is poised to jump, is grabbed and taken down from ledge. Hanging: Person has noose around neck but has not yet started to hang - is stopped from doing so.<br>***Has there been a time when you started to do something to end your life but someone or something stopped you before you actually did anything?***<br>If yes, describe:	**Yes** ☐ **No** ☐<br>Total # of interrupted<br>____
**Aborted Attempt:** When person begins to take steps toward making a suicide attempt, but stops themselves before they actually have engaged in any self-destructive behavior. Examples are similar to interrupted attempts, except that the individual stops him/herself, instead of being stopped by something else.<br>***Has there been a time when you started to do something to try to end your life but you stopped yourself before you actually did anything?***<br>If yes, describe:	**Yes** ☐ **No** ☐<br>Total # of aborted<br>____
**Preparatory Acts or Behavior:** Acts or preparation towards imminently making a suicide attempt. This can include anything beyond a verbalization or thought, such as assembling a specific method (e.g., buying pills, purchasing a gun) or preparing for one's death by suicide (e.g., giving things away, writing a suicide note).<br>***Have you taken any steps towards making a suicide attempt or preparing to kill yourself (such as collecting pills, getting a gun, giving valuables away or writing a suicide note)?***<br>If yes, describe:	**Yes** ☐ **No** ☐
**Suicidal Behavior:** Suicidal behavior was present during the assessment period?	**Yes** ☐ **No** ☐
**Suicide:**	**Yes** ☐ **No** ☐
Answer for Actual Attempts Only	Most Lethal Attempt Date:
**Actual Lethality/Medical Damage:**<br>0. No physical damage or very minor physical damage (e.g., surface scratches).<br>1. Minor physical damage (e.g., lethargic speech; first-degree burns; mild bleeding; sprains).<br>2. Moderate physical damage; medical attention needed (e.g., conscious but sleepy, somewhat responsive; second-degree burns; bleeding of major vessel).<br>3. Moderately severe physical damage; *medical* hospitalization and likely intensive care required (e.g., comatose with reflexes intact; third-degree burns less than 20% of body; extensive blood loss but can recover; major fractures).<br>4. Severe physical damage; *medical* hospitalization with intensive care required (e.g., comatose without reflexes; third-degree burns over 20% of body; extensive blood loss with unstable vital signs; major damage to a vital area).<br>5. Death	*Enter Code*<br>____
**Potential Lethality: Only Answer if Actual Lethality=0** Likely lethality of actual attempt if no medical damage (the following examples, while having no actual medical damage, had potential for very serious lethality: put gun in mouth and pulled the trigger but gun fails to fire so no medical damage; laying on train tracks with oncoming train but pulled away before run over).<br>0 = Behavior not likely to result in injury<br>1 = Behavior likely to result in injury but not likely to cause death<br>2 = Behavior likely to result in death despite available medical care	*Enter Code*<br>____

© 2008 Research Foundation for Mental Hygiene, Inc. C-SSRS—Since Last Visit (Version 1/14/09) Page 2 of 2

APPENDIX O. PARKINSON'S DISEASE DIARY

PARKINSON'S DISEASE DIARY

NAME ______________________________ DATE __________________

Instructions: For each half-hour time period place one check mark to indicate your predominant states during most of that period.
ON = Time when medication is providing benefit with regard to mobility, slowness, and stiffness.
OFF = Time when medication has worn off and is no longer providing benefit with regard to mobility, slowness, and stiffness.
Dyskinesia = involuntary twisting, turning movements. These movements are an effect of medication and occur during ON time.
Non-troublesome dyskinesia does not interfere with function or cause meaningful discomfort. Troublesome dyskinesia interferes with function or causes meaningful discomfort.
Tremor is shaking back and forth and is not considered dyskinesia.

time	asleep	OFF	ON without dyskinesia	ON with non-troublesome dyskinesia	ON with troublesome dyskinesia
6:00 AM					
:30					
7:00 AM					
:30					
8:00 AM					
:30					
9:00 AM					
:30					
10:00 AM					
:30					
11:00 AM					
:30					
12:00 PM					
:30					
1:00 PM					
:30					
2:00 PM					
:30					
3:00 PM					
:30					
4:00 PM					
:30					
5:00 PM					
:30					

time	asleep	OFF	ON without dyskinesia	ON with non-troublesome dyskinesia	ON with troublesome dyskinesia
6:00 PM					
:30					
7:00 PM					
:30					
8:00 PM					
:30					
9:00 PM					
:30					
10:00 PM					
:30					
11:00 PM					
:30					
12:00 AM					
:30					
1:00 AM					
:30					
2:00 AM					
:30					
3:00 AM					
:30					
4:00 AM					
:30					
5:00 AM					
:30					

© RA Hauser 1995

APPENDIX P. EXCIPIENTS IN IPX203, IPX203 PLACEBO, IR CD-LD, AND IR PLACEBO

IPX203 (Various Strengths)	IPX203 Placebo	IR CD-LD (25-100 mg)	IR Placebo
Microcrystalline Cellulose, NF	Microcrystalline Cellulose, NF	Crospovidone	Microcrystalline cellulose, NF
Croscarmellose Sodium, NF	Talc, USP	Hydroxypropyl Cellulose	Magnesium Stearate, NF
Magnesium Stearate, NF	Magnesium Stearate, NF	Magnesium Stearate	Quinoline yellow E104
Mannitol, USP	Sugar Spheres, NF	Microcrystalline cellulose	
Sodium Lauryl Sulfate, NF	Methacrylic acid copolymer Type A, NF	Starch (corn)	
Povidone, USP	Triethyl citrate, NF	D&C Yellow No. 10	
Cellulose Acetate	Hard gelatin capsules	Aluminum Oxide	
Copovidone, NF			
Amino Methacrylate Copolymer, NF			
Methacrylic acid copolymer Type A, NF			
Triethyl Citrate, NF			
Talc, USP			
Hard gelatin capsules			

APPENDIX Q. CLINICAL LABORATORY STUDIES

HEMATOLOGY

hemoglobin	% lymphocytes	absolute lymphocytes
hematocrit	% monocytes	absolute monocytes
red blood cell count	% basophils	absolute basophils
white blood cell count	% eosinophils	absolute eosinophils
% neutrophils	absolute neutrophils	platelet count

CHEMISTRY

sodium	calcium	indirect bilirubin
potassium	phosphorous	alkaline phosphatase
chloride	albumin	alanine aminotransferase (ALT, SGPT)
carbon dioxide	total protein	aspartate aminotransferase (AST, SGOT)
blood urea nitrogen (BUN)	uric acid	creatine phosphokinase
creatinine	total bilirubin	lactate dehydrogenase
glucose	direct bilirubin	

URINALYSIS

pH	ketones	leukocyte esterase
specific gravity	microscopic exam (RBC and WBC, only when indicated)	protein
blood		
glucose		

URINE DRUG TEST

amphetamines	benzodiazepines
barbiturates	
cannabinoids	
cocaine metabolites	
opiates	
phencyclidines	

ALCOHOL BREATH TEST

PREGNANCY TEST
Urine pregnancy test (to be completed on site) for female subjects of childbearing potential.

IPX203 (CARBIDOPA-LEVODOPA) EXTENDED-RELEASE CAPSULES

IPX203-B16-02

A RANDOMIZED CONTROLLED STUDY TO COMPARE THE SAFETY AND EFFICACY OF IPX203 WITH IMMEDIATE-RELEASE CARBIDOPA-LEVODOPA IN PARKINSON'S DISEASE PATIENTS WITH MOTOR FLUCTUATIONS

SPONSOR

Impax Laboratories, Inc.,

acting through its Impax Specialty Pharma division (Impax)

30831 Huntwood Ave.

Hayward, CA 94544

Original Protocol, May 18, 2017

Amendment 1, August 30, 2017

Amendment 2, October 23, 2017

CONFIDENTIALITY STATEMENT

All information provided is the property of Impax Laboratories, Inc. and may not be divulged, published or otherwise disclosed without written consent of Impax. All information provided to the Investigator by the Sponsor, including clinical observations at the investigative site, shall be held strictly confidential and confined to the clinical personnel involved in conducting the study, under the supervision of the Investigator. This includes but is not limited to preclinical data, protocols, case report forms, and verbal or written communications. This information may be related in confidence to the Institutional Review Board or other committees functioning in a similar capacity. All reports, subject samples, data published or submitted to third parties will be identified by a coded number and initials only in order to maintain subject confidentiality.

SIGNATURE PAGE

Reviewed and approved by:

Robert Rubens, MD, MBA<br>Senior Director, Clinical Development	10/23/17<br>Date
Barbara Pruitt, RN, BS<br>Associate Director, Clinical Operations	10/23/17<br>Date
Yoshiko Stowell, PhD<br>Manager, Regulatory Affairs	23 Oct 17<br>Date
Phillip Dinh, PhD<br>Director, Statistics	23 Oct 17<br>Date
Aravind Mittur, PhD<br>Director, Clinical Pharmacology	23 OCT 17<br>Date

INVESTIGATOR'S AGREEMENT

Protocol No.: IPX203-B16-02

Protocol Title: A Randomized Controlled Study to Compare the Safety and Efficacy of IPX203 with Immediate-Release Carbidopa-Levodopa in Parkinson's Disease Patients with Motor Fluctuations

I have read this protocol and agree to conduct the study as outlined herein, complying with the obligations and requirements of clinical investigators and all other requirements of International Conference on Harmonization (ICH), Good Clinical Practice (GCP), and the appropriate regulatory authority.

I will provide copies of the protocol and all pertinent information to all individuals responsible to me who assist in the conduct of this clinical study. I will discuss this material with them to ensure that they are fully informed regarding the study medication, the conduct of the study, and the obligations of confidentiality.

____ ____________________

Principal Investigator's signature Date

____

Principal Investigator's printed name

STUDY CONTACT INFORMATION

Changes in Impax study personnel listed on this page do not require a protocol amendment.

**Role**	**Name and Contact Information**
Sponsor	Impax Laboratories, Inc. acting through its Impax Specialty Pharma division (Impax)<br>30831 Huntwood Avenue<br>Hayward, CA 94544-7037<br>(510) 240-6000<br>FAX: (510) 240-6113 and (510) 240-6114
Medical Monitor	Robert Rubens, MD, MBA<br>Senior Director, Clinical Research & Development<br>Mobile: (510) 303-8207<br>Office: (510) 240-6012<br>E-mail: rrubens@impaxlabs.com
Statistician	Phillip Dinh, PhD<br>Director, Statistics<br>(510) 240-6402<br>E-mail: Phillip.Dinh@impaxlabs.com
Clinical Pharmacology	Aravind Mittur, PhD<br>Director, Clinical Pharmacology<br>(510) 240-6437<br>E-mail: amittur@impaxlabs.com
Clinical Research Associates	Barbara Pruitt, RN, BS<br>Associate Director, Clinical Operations<br>Office: (510) 240-6003<br>Mobile: (510) 329-8489<br>E-mail: bpruitt@impaxlabs.com
	Weiru Hong, MS<br>Senior CRA, Clinical Operations<br>Office: (510) 240-6078<br>Mobile: (510) 731-8309<br>E-mail: whong@impaxlabs.com
Serious Adverse Event Reporting	FAX: (866) 954-2064

1. SYNOPSIS

**Name of Sponsor/Company:** Impax Laboratories, Inc. acting through its Impax Specialty Pharma division (Impax)
**Name of Investigational Product:** IPX203 (carbidopa-levodopa) Extended-Release Capsules
**Name of Active Ingredients:** carbidopa (CD), levodopa (LD)
**Protocol Title:** A Randomized Controlled Study to Compare the Safety and Efficacy of IPX203 with Immediate-Release Carbidopa-Levodopa in Parkinson's Disease Patients with Motor Fluctuations
**Protocol No.:** IPX203-B16-02
**Study center(s):** Multicenter
**Phase of Development:** Phase 3
**Objectives:** To evaluate the safety and efficacy of IPX203 in comparison to IR CD-LD in the treatment of CD-LD-experienced subjects with Parkinson's disease (PD) who have motor fluctuations.
**Methodology:** This is a multicenter, randomized, double-blind, double-dummy, active-controlled, parallel-group study. The study will consist of a 3-week, open-label IR CD-LD dose adjustment period; a 4-week, open-label period for conversion to IPX203; followed by a 13-week double-blind treatment period with subjects randomized in a 1:1 ratio, stratified by center, to receive either IPX203 (with matching IR CD-LD placebo) or IR CD-LD (with matching IPX203 placebo).<br>Subjects will continue to take permitted non-CD-LD-based PD medications throughout the study if documented in their prestudy regimen and if dosing regimens have been stable for at least 4 weeks prior to Visit 1. A "stable dosing regimen" means no change in dose or in dosing frequency.<br>• Within 4 weeks following the Screening visit, eligible subjects will complete their PD Diaries on each of the 3 consecutive days immediately prior to Visit 1.<br>• Following Visit 1, qualified subjects will enter a 3-week, open-label IR CD-LD treatment period allowing for dose adjustment. **The dosing regimen of IR CD-LD may be adjusted during the dose adjustment period** to minimize "Off" time without causing troublesome dyskinesia. The doses and regimens of the subject's other non-CD-LD PD medications (dopamine agonists, MAO-B inhibitors, amantadine, anticholinergics) should remain stable throughout this study. Any adjustments to the IR CD-LD dosing regimen will be done in consultation with the Investigator or qualified site personnel and will be recorded. The IR CD-LD dosing regimen should be stable for at least 5 days prior to returning for Visit 2. Rescue with additional or modified doses of concomitant PD medications or with use of CD-LD products other than the dispensed study medications is not permitted and will trigger discontinuation from the study. Subjects will complete their 3-day PD Diaries on each of the 3 consecutive days immediately prior to Visit 2.<br>• Following completion of the IR CD-LD dose adjustment period, subjects will begin a 4-week open-label period for conversion to IPX203. The initial dosing regimen of IPX203 will be based on the most frequent dose of the subject's dosing regimen of IR CD-LD at the end of the dose adjustment period (Visit 2). A 25-100 mg dose of IR CD-LD converts to a 70-280 mg CD-LD dose of IPX203 (2 capsules of 35-140 mg CD-LD IPX203), and a 12.5-50 mg dose of IR CD-LD converts to a 35-140 mg CD-LD dose of IPX203, but with a longer duration of effect. It is recommended that IPX203 should be dosed approximately every 8 hours with the exception that subjects who are currently receiving a total daily dose of less than 125-500 mg

IR CD-LD at the end of the dose adjustment period will be initially administered every 12 hours. The dosing interval may be reduced to approximately every 8 hours if the subject does not achieve an acceptable duration of effect. **The dosing regimen of IPX203 may be adjusted during the dose conversion period** to achieve the optimal balance of efficacy and tolerability (minimize "Off" time without causing troublesome dyskinesia or other dopaminergic side effects). The doses and regimens of the subject's other non-CD-LD PD medications (dopamine agonists, MAO-B inhibitors, amantadine, anticholinergics) should remain stable throughout this study. The subject must be on a stable dosing regimen of IPX203 (no change in dose or in dosing frequency) for at least 5 days prior to returning for Visit 4. Any adjustments to the IPX203 dosing regimen will be done in consultation with the Investigator or qualified site personnel and will be recorded. Rescue with additional or modified doses of concomitant PD medications or with use of CD-LD products other than the dispensed study medications is not permitted and will trigger discontinuation from the study. Subjects will return to the clinic in 2 weeks for Visit 3 followed by Visit 4, 2 weeks later. Subjects will complete their 3-day PD Diaries on each of the 3 consecutive days immediately prior to Visit 4.

- Subjects who successfully complete the IPX203 dose conversion period will be randomized in 1:1 ratio, stratified by center, at Visit 4 into one of two parallel treatment arms of IPX203 (with matching IR CD-LD placebo) or IR CD-LD (with matching IPX203 placebo). The subjects will undergo 13 weeks of double-blind maintenance therapy with the stable dosing regimen established at the end of Week 3 (Visit 2) for IR CD-LD and at the end of Week 7 (Visit 4) for IPX203. Rescue with additional or modified doses of concomitant PD medications or with use of CD-LD products other than the dispensed study medications is not permitted and will trigger discontinuation from the study. Subjects will return to the clinic for 3 visits (Visits 5, 6, and 7) and will complete their 3-day PD Diaries on each of the 3 consecutive days immediately prior to each of these visits.

Number of patients (planned): Assuming approximately an 18% prerandomization drop-out, approximately 510 subjects will be enrolled to randomize 420 subjects.

Diagnosis and main criteria for inclusion:

<u>Inclusion Criteria</u>

- Male or female subjects diagnosed at age ≥ 40 years with PD, consistent with the United Kingdom Parkinson's Disease Society Brain Bank Diagnostic Criteria and who are being treated with stable regimens of CD-LD but experiencing motor fluctuations.
- Hoehn and Yahr Stages 1, 2, 3, or 4 in the "On" state (part of Movement Disorders Society version of the Unified Parkinson's Disease Rating Scale [MDS-UPDRS] Part III)
- Montreal Cognitive Assessment (MoCA) score ≥ 24 at Screening Visit in "On" state.
- By history, for the 4 weeks prior to Screening, the subject experiences daily "wearing-off" episodes with periods of bradykinesia in combination with at least one of rest tremor or rigidity, experiences an "Off" state upon awakening on most mornings, and reports an average of at least 2.5 cumulative hours per day of "Off" time during the waking hours.
- Able to differentiate "On" state from "Off" state as determined by at least 75% concordance with a trained rater in "On/Off" ratings for 8 ratings over a 4-hour training period. The concordance must include at least 1 "On" and 1 "Off" rating and must be achieved within two 4-hour training sessions.
- At Visit 1, review of the 3-day PD Diaries confirms the following: that the subject is able to properly complete the Diaries with valid entries; and that the subject has an <u>average of at least 2.5 hours</u> per day of "Off" time during waking hours over the 3 days with at least 1.5 hours of

cumulative “Off” time on each day.

- Responsive to CD-LD therapy and currently being treated on a stable regimen with CD-LD for at least 4 weeks prior to Visit 1 and:
  - Requires at least 100 mg of LD from IR CD-LD for the first morning dose
  - Requires a total daily dose of at least 400 mg of LD and takes a maximum total daily dose of 2400 mg LD, from IR CD-LD alone or IR CD-LD in combination with a single daily bedtime dose of CR CD-LD
  - Has a dosing frequency of 4 to 9 times daily of CD-LD
  - By history, typically experiences an “On” response with the first dose of IR CD-LD of the day, but the efficacy of this dose typically lasts less than 4 hours.
- At Screening, the subject has predictable “Off” periods defined by a score of 1 or 2 on Item #4.5 (Complexity of Motor Fluctuations) of the MDS-UPDRS Part IV B (Motor Fluctuations).
- At Screening, the MDS-UPDRS Part III total score in the “Off” state is at least 20 units.

Exclusion Criteria

- Used any doses of controlled-release (CR) CD-LD apart from a single daily bedtime dose within 4 weeks prior to Visit 1.
- Used any dose of Rytary for the past 4 weeks prior to Visit 1 or were considered IPX066 or Rytary failures for reasons of efficacy or safety.
- Had prior neurosurgical treatment for PD or if such procedure is planned or anticipated during the study period.
- Allergic to any excipient in the study drugs.
- History of glaucoma with intraocular pressures that are elevated despite appropriate medical management.
- History of seizure or epilepsy and experienced at least 1 seizure during the past 12 months or has not been compliant with medically recommended therapy or visits.
- History of myocardial infarction with residual atrial, nodal, or ventricular arrhythmias that are not controlled with medical and/or surgical interventions. A recent (≤ 12 months) history of myocardial infarction with secondary arrhythmias is exclusionary regardless of the therapeutic control.
- Received within 4 weeks of Screening or planning to take during participation in the clinical study:
  - Any doses of a CR CD-LD apart from a single daily bedtime dose, any doses of Rytary, additional CD (eg, Lodosyn) or benserazide (eg, Serazide), or catechol-O-methyl transferase inhibitors (entacapone or tolcapone) or medications containing these inhibitors (Stalevo),
  - Nonselective monoamine oxidase inhibitors (MAOI), apomorphine, or antidopaminergic agents, including antiemetics.
- Subjects who have previously participated in an IPX203 study.

Investigational product, dosage and mode of administration: IPX203 (carbidopa-levodopa) Extended-Release capsules, containing 35-140 mg of CD-LD and matching placebo, for oral administration.

Reference therapy, dosage and mode of administration: Immediate-Release carbidopa-levodopa (IR CD-LD) tablets containing 25-100 mg of CD-LD, and matching placebo, for oral administration.

Duration of treatment: Approximately 24 weeks, including up to 4 weeks following Screening,

3 weeks of IR CD-LD dose adjustment, 4 weeks of IPX203 dose conversion, and 13 weeks of double-blind therapy following randomization.

Criteria for evaluation:

Baseline is defined as assessments done at Visit 4 (randomization visit). Study Entry is defined as assessments done at Visit 1 (study entry visit).

Efficacy:

- Primary endpoint: Change from baseline in "Good on" time in hours per day, averaged over the PD Diary days, at the end of double-blind treatment period (Visit 7 or early termination). "Good on" time is derived from the 3-day PD Diaries and is defined as the sum of "On" time without dyskinesia and "On" time with nontroublesome dyskinesia.
- Key secondary endpoints:
  - Change from baseline in "Off" time in hours per day, averaged over the PD Diary days at the end of double-blind treatment period (Visit 7 or early termination)
  - Proportion of subjects with either "much improved" or "very much improved" in Patient Global Impression of Change (PGI-C) scores at the end of double-blind treatment period (Visit 7 or early termination)
  - Change from baseline in the MDS-UPDRS Part III at the end of double-blind treatment period (Visit 7 or early termination)
  - Change from baseline in the sum of MDS-UPDRS Parts II and III at the end of double-blind treatment period (Visit 7 or early termination)
- Additional endpoints:

  The following endpoints will be evaluated (at the post-randomization visits) as change from baseline (Visit 4) as well as change from the study entry (Visit 1), when applicable, by visits:
  - Percent "Off" time during waking hours derived from the 3-day PD Diaries
  - Average duration of each continuous "Good on" and each continuous "On"
  - Hours of (1) "Off" time (from Visit 1), (2) "Good on" time (from Visit 1), (3) "On" time with dyskinesia, (4) "On" time with troublesome dyskinesia, and (5) "On" time with nontroublesome dyskinesia, and (6) asleep time derived from the 3-day PD Diaries
  - Proportion of subjects with an improvement in "Good on" time of at least 1, 1.5, 2, 2.5, and 3 hours
  - Proportion of subjects with a reduction in "Off" time of at least 0.5, 1, 1.5, 2, 2.5, and 3 hours
  - Proportions of subjects who are "On" upon awakening and "Good on" upon awakening
  - Average time to "On" upon awakening
  - Change from baseline in the average number of motor fluctuations per day averaged over the PD Diary days. A motor fluctuation is defined as a change from "Off" to "On" state or from "On" to "Off" state.
  - MDS-UPDRS total score (sum of Parts I, II, III, and IV) and Parts I, II, and IV separately
  - MDS-UPDRS Part III and Parts II + III combined (from Visit 1)
  - MDS-UPDRS Part II Question 2.9

- PDQ-39 total score and individual domain scores
- NMSS total score and individual domains
- PDSS-2 total score and individual domains
- PDSS-2 items 9, 10, 11, 12, and 13 combined
- PAS total score and individual domains
- PGI-S
- Proportion of subjects with either “severely ill” or “extremely severely ill” on the PGI-S
- CGI-S
- Proportion of subjects with either “severely ill” or “among the most extremely ill of subjects” on the CGI-S
- PGI-C scores
- CGI-C scores
- Proportion of subjects with either “much improved” or “very much improved” on the CGI-C

- **Safety:** electrocardiograms (ECGs), clinical laboratory tests, physical examinations, Columbia-Suicide Severity Rating Scale (C-SSRS), and supine and standing orthostatic vital signs; adverse events and concomitant medications evaluated throughout the course of the study.

Statistical methods: For the primary endpoint, assuming a difference of 1 hour between IPX203 and IR CD-LD in “Good on” time and a standard deviation of the treatment difference to be 3.0 hours, a sample size of 210 per arm will be needed to ensure at least 90% power at a 0.05 significance level. Assuming approximately an 18% prerandomization drop-out, approximately 510 subjects would need to be enrolled to randomize 420 subjects.

The primary efficacy endpoint of change from baseline in “Good on” time will be analyzed using a mixed model for repeated measures (MMRM) model. The model will include baseline (Visit 4) “Good on” time as a covariate, treatment, visit, and pooled center as fixed effects, and a treatment-by-visit interaction. The model will employ an unstructured within subject covariance matrix and a restricted maximum likelihood (ReML) estimation method. The degree-of-freedom of the denominator will be estimated using the Kenward-Roger method.

The key secondary endpoints (change from baseline in “Off” time, change from baseline in MDS-UPDRS Part III, and change from baseline in the sum of the MDS-UPDRS Parts II and III) will be analyzed using MMRM models similar to the primary analysis model. The proportion of subjects with either “much improved” or “very much improved” on the PGI-C will be analyzed using Cochran-Mantel-Haenszel test to compare the two treatment groups with pooled center as a stratification factor.

In order to control the type I error rate, the primary and key secondary endpoints will be tested in a single hierarchical order: (1) change from baseline in “Good on” time, (2) change from baseline in “Off” time, (3) proportion of subjects with either “much improved” or “very much improved” in PGI-C, (4) change from baseline in the MDS-UPDRS Part III, (5) change from baseline in the sum of MDS-UPDRS Parts II and III.

Quantitative safety data will be summarized using descriptive statistics and frequency distributions. Qualitative safety data will be summarized by frequencies and percentages. All summaries will be presented by treatment arms.

2. TABLE OF CONTENTS, LIST OF TABLES, AND LIST OF FIGURES

TABLE OF CONTENTS

1. SYNOPSIS 5
2. TABLE OF CONTENTS, LIST OF TABLES, AND LIST OF FIGURES 10
3. LIST OF ABBREVIATIONS AND DEFINITIONS OF TERMS 16
4. INTRODUCTION 19
5. TRIAL OBJECTIVES 23
6. INVESTIGATIONAL PLAN 24
6.1. Overall Study Design 24
6.2. Number of Subjects 25
6.3. Treatment Assignment 26
6.4. Dosing and Dose Determination Criteria 26
6.4.1. IR CD-LD Dose Adjustment Period 26
6.4.2. IPX203 Dose Conversion Period 26
6.4.3. Double-Blind Maintenance Period 27
6.5. Criteria for Study Termination 28
7. SELECTION AND WITHDRAWAL OF SUBJECTS 29
7.1. Subject Inclusion Criteria 29
7.2. Subject Exclusion Criteria 30
7.3. Subject Withdrawal Criteria 31
8. STUDY PROCEDURES 33
8.1. Screening Visit 37
8.2. Visit 1 – Start of IR CD-LD Dose Adjustment 38
8.2.1. Prior to Visit 1 38
8.2.2. At Visit 1 38
8.2.3. Post Visit 1 39
8.3. Visits 2 (Week 3) and Visit 3 (Week 5) – IPX203 Dose Conversion 39
8.3.1. Prior to Visit 2 39
8.3.2. Prior to Visit 3 39
8.3.3. At Visits 2 and 3 39

8.3.4. Post Visits 2 and 3 40
8.4. Visit 4 (Week 7) – Randomization 40
8.4.1. Prior to Visit 4 40
8.4.2. At Visit 4 40
8.5. Visit 5 (Week 10) and Visit 6 (Week 15) 41
8.5.1. Prior to Visit 5 and 6 41
8.5.2. At Visit 5 and 6 41
8.6. Visit 7 (Week 20) – End of Study/Study Exit 42
8.6.1. Prior to Visit 7 42
8.6.2. At Visit 7 42
8.7. Early Termination 43
8.7.1. Subjects Who Terminate Prior to Randomization 43
8.7.2. Subjects Who Terminate Early after Randomization 43
8.8. Blood Volume 44
9. TREATMENT OF SUBJECTS 45
9.1. Description of Study Drug 45
9.2. Concomitant Medications 45
9.2.1. Permitted PD Medications 45
9.2.2. Prohibited Medications and Procedures 45
9.2.3. Rescue Medications 46
9.3. Treatment Compliance 46
9.4. Randomization and Blinding 46
10. STUDY DRUG MATERIALS AND MANAGEMENT 47
10.1. Study Drug 47
10.2. Study Drug Packaging and Labeling 47
10.3. Study Drug Storage 47
10.4. Study Drug Administration 48
10.5. Study Drug Dispensing and Accountability 48
10.6. Study Drug Handling and Disposal 48
11. ASSESSMENT OF EFFICACY 49
11.1. Parkinson's Disease Diary 49
11.2. Patient and Investigator Global Assessments 49

11.3. Movement Disorders Society Version of Unified Parkinson's Disease Rating Scale 49
11.4. Additional Assessments 50
12. ASSESSMENT OF SAFETY 51
12.1. Safety Parameters 51
12.2. Adverse Events 51
12.2.1. Definition of Adverse Event 51
12.2.2. Recording Adverse Events 51
12.2.3. Follow-up 52
12.2.4. Relationship to Study Drug 52
12.2.5. Assessment of Severity 52
12.3. Serious Adverse Events 52
12.3.1. Definition of Serious Adverse Event 52
12.3.2. Reporting Serious Adverse Events 53
12.4. Pregnancy 53
12.5. Other Safety Parameters and Related Information 54
13. STATISTICS 55
13.1. Study Design and Sample Size Estimation 55
13.2. Demographics/Baseline Comparability 55
13.3. Efficacy Endpoints 55
13.4. Analysis of Efficacy Data 57
13.4.1. Primary Efficacy Endpoint 57
13.4.2. Key Secondary Efficacy Endpoints 57
13.4.3. Additional Efficacy Endpoints 58
13.5. Center Pooling Algorithm 58
13.6. Sensitivity Analyses of the Primary Endpoint and Key Secondary Endpoints 59
13.6.1. Assessing Assumptions of the Mixed Model for Repeated Measures (MMRM) 59
13.6.2. Complete Case Analysis 59
13.6.3. Single LOCF/BLOCF Imputation 59
13.6.4. Pattern-Mixture Model 60
13.7. Subgroup Analyses 61
13.8. Multiplicity Adjustments 61

13.9. Analysis Populations 62
13.9.1. Safety Analysis Set 62
13.9.2. Intent-to-Treat Analysis Set 62
13.9.3. Modified Intent-to-Treat Analysis Set 62
13.9.4. Completers Analysis Set 62
13.10. Handling of Missing Data 62
13.10.1. Missing Data for PD Diaries 62
13.10.2. Missing Data for Global Assessments (PGI-C, CGI-C, PGI-S and CGI-S) 63
13.10.3. Missing Data for MDS-UPDRS 63
13.11. Analysis of Safety 64
14. ADMINISTRATIVE PROCEDURES 65
14.1. Guidelines for Good Clinical Practice 65
14.2. Institutional Review Board Approval 65
14.3. Informed Consent 65
14.4. Study Monitoring 65
14.5. Protocol Amendments 65
14.6. Termination of Study 66
14.7. Case Report Forms 66
14.8. Investigator's Final Conduct Report 66
14.9. Records Retention 66
15. PUBLICATION POLICY 67
16. LIST OF REFERENCES 68
17. APPENDICES 70
APPENDIX A. PRESCRIBING INFORMATION FOR IR CD-LD 70
APPENDIX B. INSTRUCTIONS FOR DOSE CONVERSION TO IPX203 81
APPENDIX C. UNITED KINGDOM PARKINSON'S DISEASE SOCIETY BRAIN BANK DIAGNOSTIC CRITERIA FOR PARKINSON'S DISEASE 83
APPENDIX D. MONTREAL COGNITIVE ASSESSMENT (MOCA) 84
APPENDIX E. MOVEMENT DISORDERS SOCIETY VERSION OF UNIFIED PARKINSON'S DISEASE RATING SCALE (MDS-UPDRS) 90
APPENDIX F. PATIENT GLOBAL IMPRESSION OF CHANGE (PGI-C) 123
APPENDIX G. CLINICAL GLOBAL IMPRESSION OF CHANGE (CGI-C) 124
APPENDIX H. PATIENT GLOBAL IMPRESSION OF SEVERITY (PGI-S) 125

APPENDIX I. CLINICAL GLOBAL IMPRESSION OF SEVERITY (CGI-S)......................126
APPENDIX J. 39-ITEM PARKINSON'S DISEASE QUESTIONNAIRE (PDQ-39) 127
APPENDIX K. GASTROPARESIS CARDINAL SYMPTOM INDEX (GCSI).....................131
APPENDIX L. NON-MOTOR SYMPTOM ASSESSMENT SCALE FOR PARKINSON'S DISEASE (NMSS)..133
APPENDIX M. PARKINSON'S DISEASE SLEEP SCALE-2 (PDSS-2) 136
APPENDIX N. PARKINSON ANXIETY SCALE (PAS)...137
APPENDIX O. COLUMBIA-SUICIDE SEVERITY RATING SCALE (C-SSRS)................141
APPENDIX P. PARKINSON'S DISEASE DIARY 148
APPENDIX Q. EXCIPIENTS IN IPX203, IPX203 PLACEBO, IR CD-LD, AND IR PLACEBO 149
APPENDIX R. CLINICAL LABORATORY STUDIES 150

LIST OF TABLES

Table 1: Abbreviations and Specialist Terms 16
Table 2: LD Dosage in Study IPX203-B14-02 20
Table 3: Recommended Starting IPX203 LD Dosing Regimen Based on the Dosing Regimen of IR CD-LD at the End of the Dose Adjustment Period....................27
Table 4: Events Schedule for Impax Study IPX203-B16-02 34
Table 5: Study Drugs for Study IPX203-B16-02 45

LIST OF FIGURES

Figure 1: Study Flow Chart 25

3. LIST OF ABBREVIATIONS AND DEFINITIONS OF TERMS

The following abbreviations and specialist terms are used in this study protocol.

Table 1: Abbreviations and Specialist Terms

Abbreviation or Specialist Term	Explanation
AADC	aromatic amino acid decarboxylase
ADL	activities of daily living
AE	adverse event
ANCOVA	analysis of covariance
ANOVA	analysis of variance
BLOCF	baseline observation carried forward
BMI	body mass index
CD	carbidopa
CGI-C	Clinical Global Impression of Change
CGI-S	Clinical Global Impression of Severity
CR	controlled release
CRF	case report form
C-SSRS	Columbia-Suicide Severity Rating Scale
ECG	electrocardiogram
ER	extended release
FDA	Food and Drug Administration
GCP	Good Clinical Practice
GCSI	Gastroparesis Cardinal Symptom Index
HIPAA	Health Insurance Portability and Accountability Act
ICF	informed consent form
ICH	International Conference on Harmonization
IEC	independent ethics committee

Abbreviation or Specialist Term	Explanation
IR	immediate release
IRB	institutional review board
IWRS	interactive web response system
LD	levodopa
LOCF	last observation carried forward
MAOI	monoamine oxidase inhibitors
MAR	missing at random
M-EDL	Motor Aspects of Experiences of Daily Living
MedDRA	Medical Dictionary for Regulatory Activities
MDS-UPDRS	Movement Disorders Society version of the Unified Parkinson's Disease Rating Scale
mITT	modified intent-to-treat
MMRM	mixed model for repeated measures
MNAR	missing not at random
MoCA	Montreal Cognitive Assessment
nM-EDL	Non-Motor Aspects of Experiences of Daily Living
NMSS	Non-Motor Symptom Assessment Scale
PAS	Parkinson Anxiety Scale
PD	Parkinson's disease
PDQ-39	39-item Parkinson's Disease Questionnaire
PDSS-2	Parkinson's Disease Sleep Scale-2
PGI-C	Patient Global Impression of Change
PGI-S	Patient Global Impression of Severity
PK	pharmacokinetic (adjective)<br>pharmacokinetics (singular noun)
PI	principal investigator

Abbreviation or Specialist Term	Explanation
PMM	pattern-mixture models
ReML	restricted maximum likelihood
SAE	serious adverse event
SAP	statistical analysis plan
SD	standard deviation
TEAE	treatment-emergent adverse event
US	United States

4. INTRODUCTION

Parkinson's disease (PD) is a progressive neurodegenerative disorder of the extrapyramidal nervous system. Levodopa (LD) used in combination with carbidopa (CD) is considered the gold standard for the symptomatic treatment of PD. LD is a dopamine precursor converted to dopamine by aromatic amino acid decarboxylase (AADC). Carbidopa is an AADC inhibitor that does not cross the blood-brain barrier. When used in combination with LD, CD increases the plasma half-life of LD from 50 minutes to 1.5 hours. Carbidopa inhibits the conversion of LD into dopamine in the periphery, thereby reducing the peripheral side-effects caused by dopamine and increasing the amount of LD available for transport into the brain. The administration of CD with LD reduces the dose of LD required to produce a dopaminergic response by about 75 percent (Sinemet prescribing information; Appendix A).

Due to its proven efficacy, LD is prescribed eventually to most subjects with PD. However, long-term use of LD is associated with certain complications, including "wearing-off" or "end-of-dose effect," where symptom control decreases causing the drug effects to wear off sooner. As the disease progresses further, motor complications, namely dyskinesias and motor "On/Off" fluctuations, develop in about 50% of the patients after 5 years of treatment (Fahn 1999). Such motor complications can be a significant source of disability and their management is a major unmet need in the treatment of PD.

Mechanisms underlying motor complications involving dyskinesias and "On/Off" fluctuations in PD are unclear. The pulsatile nature of standard orally administered LD is thought to contribute to the appearance of motor complications. Chronic intermittent pulsatile stimulation of the dopamine receptors that are under tonic control contributes to the development of dyskinesia in PD animal models as compared to animals treated with continuous infusion (Juncos et al 1989, Engber et al 1989, Blanchet et al 1995). In addition, unreliable absorption of LD potentially due to erratic gastric empting and variable in vivo dissolution of LD products is thought to contribute to the delay or inadequate response after oral dosing with standard CD-LD products (Melamed et al 1986, Kurlan et al 1988, Stocchi et al 1994). These findings suggest that motor complications in patients with PD may be less likely to develop with continuous dopaminergic stimulation.

Intraduodenal infusion of LD has been shown to significantly reduce motor complications and to reduce "Off" time. The findings of infusion studies in PD patients indicate that the maintenance of stable plasma LD concentrations and the avoidance of low trough levels are effective in reducing "Off" hours, increasing "On" hours without disabling dyskinesia, and reducing the severity of dyskinesia versus standard oral LD formulations (Mizuno 2007, Nilsson et al 2001, Nyholm et al 2005, Stocchi et al 2005). These findings provide a strong rationale for the development of an extended-release (ER) oral dosage form that delivers a constant LD plasma concentration in order to optimize relief of PD symptoms, and to minimize "Off" time and dyskinesia.

IPX203 is an investigational product containing CD-LD that is being developed by Impax Laboratories, Inc. through its Impax Specialty Pharma division (Impax). The primary objective of the IPX203 program is to develop an extended-release product that can attain therapeutic LD

plasma concentrations rapidly and maintain constant LD plasma concentrations for a longer duration than currently approved products with minimal peak-to-trough fluctuations. IPX203 is designed to be dosed approximately every 8 hours.

Impax characterized the PK and pharmacodynamics of IPX203 in Study IPX203-B14-02, a single dose trial in subjects with advanced PD versus IR CD-LD and Rytary (carbidopa and levodopa) extended-release capsules. Twenty-six (26) subjects were randomized with 25 subjects completing all 3 treatments. One subject discontinued study early due to subject withdrawal. The doses of IPX203 and Rytary were determined on the basis of each subject's prestudy baseline morning dose of IR CD-LD (Table 2).

Table 2: LD Dosage in Study IPX203-B14-02

Prestudy Baseline Morning IR LD (mg)	IR LD (mg)	Rytary LD (mg)	IPX203 LD (mg)
100	100	340	360
150	150	485	540
200	200	630	720
250	250	780	810

Administration of IPX203 yielded an initial increase in LD plasma concentrations that was similar to IR CD-LD but maintained LD concentrations for a longer duration than either IR CD-LD or Rytary. The bioavailability of LD (based on AUC_{∞}) from IPX203 was 78% relative to IR CD-LD and about 14% more than Rytary. Plasma exposure to LD (C_{max} and AUC_{∞}) following IPX203 increased in an approximately dose-proportional manner. Pharmacodynamic effects as measured by change from baseline scores on MDS-UPDRS Part III were consistent with the PK profiles of LD. Following IPX203 treatment, decrements in the Part III total score (reflecting improvements in motor symptoms) lasted for a longer duration than either IR CD-LD or Rytary. IPX203 provided a longer duration of effect compared with IR CD-LD and Rytary, including "Off" time and "Good on" time based on the Assessment of Subject's Motor State and on a range of improvement thresholds of the MDS-UPDRS Part III. The results were consistent with the prolonged LD plasma concentration profile with IPX203 compared to IR CD-LD or Rytary and support a dosing interval of approximately 8 hours.

Of the 26 subjects who received at least one of the 3 treatments, 9 (34.6%) reported at least one treatment-emergent adverse event (AE). None of these subjects reported a serious AE (SAE) nor did any subjects prematurely discontinue the study because of an AE. Adverse events were reported by more subjects during IR (28.0%) and IPX203 (19.2%) than during Rytary (8.0%) treatment. None of the reported AEs were classified as "severe." Adverse events reported by 2 or more subjects include: Dizziness (3 subjects), nausea (2 subjects), and hypertension (2 subjects). The numbers of subjects reporting these AEs during any single treatment period were small (0 to 2 subjects). Two subjects reported dizziness during IR CD-LD treatment and one subject each during IPX203 and Rytary treatments. Hypertension was reported by a total of 2 subjects, both reporting this AE during IPX203 and IR CD-LD treatments and 1 subject during Rytary treatment. Two subjects reported nausea only during the IR CD-LD treatment period. Of the 9 subjects reporting AEs, 6/26 (23.1%) of subjects reported AEs that were assessed as related to treatment, including all of the reports of dizziness, nausea, and dyskinesia (1 subject).

Study IPX203-B16-01 is a randomized, open-label, rater-blinded, multicenter, 2-treatment, 2-period, multiple-dose crossover study that has completed dosing. Twenty-eight (N=28) advanced PD subjects were randomized to 1 of 2 dosing sequences, with each treatment period lasting 15 days and separated by a 1-week wash-out period where subjects return to their usual stable pre-study CD-LD regimen. The objectives of this study are to compare the PK, pharmacodynamics, efficacy, and safety of IPX203 with IR CD-LD after single and multiple dosing. Subjects were permitted to take allowed non-CD-LD based PD medications throughout the study if dosing regimens had been stable for at least 4 weeks. Subjects were instructed to take their last dose of CD-LD no later than 10:00 PM on the evening prior to Day 1 of each treatment period and to withhold dosing for at least 5 hours before arriving at the site on Day 15 of each treatment period. On Day 1 of the IR CD-LD treatment period, subjects were started with a single dose of their usual prestudy first morning IR CD-LD dose. On Day 1 of the IPX203 treatment period, subjects were started with a single dose of IPX203 based on their usual prestudy first morning IR CD-LD dose using a LD conversion of 100 mg IR LD to 360 mg of IPX203 LD. During the IR CD-LD treatment period, the initial dosing regimen of IR CD-LD was the same as the subject's stable prestudy regimen. During the IPX203 treatment period, the IPX203 regimen for subsequent doses for the day was determined by identifying the most frequent prestudy IR LD dose in milligrams that the subject received in the afternoon and evening and administering IPX203 using a LD conversion of 100 mg IR LD to 270 mg of IPX203 LD. The protocol recommended that IPX203 be dosed approximately every 7 to 8 hours. During Days 1 through 9 of both treatment periods, investigators had the opportunity to adjust each subject's study medication regimen if necessary to optimize efficacy and safety. Pharmacokinetics and pharmacodynamics (MDS-UPDRS Part III and Assessments of Subject's Motor State) were periodically evaluated on Day 1 and Day 15 of each treatment period by qualified clinical staff who were blinded to dosing.

Data from this multiple-dose study confirmed the PK and pharmacodynamic results observed in the single dose study with IPX203:

- PK data from 27 subjects indicates IPX203 shows a rapid increase in LD concentrations followed by extended-release characteristics. Following IPX203; initial increases in LD concentrations were comparable to that from IR CD-LD. Bioavailability of LD following IPX203 was ~89% relative to IR CD-LD. LD plasma concentrations were sustained longer after IPX203 treatment than after IR CD-LD and support dosing every 8 hours. No accumulation of LD was evident at steady-state following IPX203 or IR CD-LD. Plasma LD concentrations following IPX203 were characterized by lower peak-to-trough fluctuation. No time-variant or time-dependent changes were noted in PK of CD or LD following IPX203.
- IPX203 demonstrated an onset of effect that was comparable to IR CD-LD in MDS-UPDRS Part III scores. IPX203 prolonged the duration over which MDS-UPDRS Part III scores were improved by prespecified threshold changes from baseline (≥4, ≥7, and ≥13 units).
- IPX203 provides a significant decrease in "Off" time and a significant increase in "Good on" time compared to IR CD-LD treatment on Day 1 and Day 15 when assessed by the Investigator's Assessment of Subject's Motor State. Subjects treated

with IPX203 did not experience a significant increase in “On” time with troublesome dyskinesia compared to IR CD-LD.

- Subjects achieved significant improvements in “Off” time, “Good on” time, and frequency of motor state fluctuations based on the 3-day PD Diaries.
- Twenty-eight subjects were enrolled in the multiple dose study and 27 subjects completed both treatments. Safety results were as follows:
  - One subject discontinued during the IPX203 treatment period due to an AE (orthostatic hypertension) that was considered possibly related to treatment.
  - A total of 39.3% (11/28) of treated subjects reported at least one treatment emergent AE, including 35.7% (10/28) during IPX203 treatment and 7.4% (2/27) during IR CD-LD treatment. Eight subjects reported AEs that were related to treatment (8 subjects during IPX203 treatment and 1 during IR CD-LD treatment).
  - Two subjects experienced serious adverse events (SAEs). One subject reported increased hypertension of mild severity during IPX203 treatment that was considered unrelated to treatment and resolved. A second subject reported moderate to severe dehydration, diarrhea, and atrial fibrillation during the washout period that were considered unrelated to treatment and resolved.
  - AEs reported in 2 or more subjects included nausea (2), dizziness (2), and dyskinesia (5), all of mild or moderate severity, and all during the IPX203 treatment.

The current protocol, Study IPX203-B16-02, is a multicenter, randomized, double-blind, double-dummy, active-controlled, parallel-group, Phase 3 study. It is designed to compare the efficacy, safety and tolerability of IPX203 with IR CD-LD following multiple doses over 13 weeks. The following IPX203 dosing guidelines will be utilized in the present study (IPX203-B16-02):

- The initial regimen of IPX203 is based on the most frequent dose of the subject’s dosing regimen of IR CD-LD at the end of dose adjustment period (Visit 2);
- A 25-100 mg dose of IR CD-LD will be converted to a 70-280 mg CD-LD dose of IPX203;
- IPX203 will be administered approximately every 8 hours for most subjects;
- Investigators may adjust the IPX203 regimen during the dose conversion period to optimize the therapeutic effect (minimize “Off” time without causing troublesome dyskinesia or other dopaminergic side effects).

The proposed dose conversion scheme for this study has been developed based on a similar dose conversion from IR CD-LD to IPX203 that was studied in the completed Phase 2a study (IPX203-B14-02, n=25) and the Phase 2b study (IPX203-B16-01, n=28), both conducted in subjects with advanced PD using similar entry criteria to the present study. The doses of IPX203 are expected to be comparable to other ER CD-LD products, such as Rytary and Duopa.

5. TRIAL OBJECTIVES

To evaluate the safety and efficacy of IPX203 in comparison to IR CD-LD in the treatment of CD-LD experienced subjects with Parkinson’s disease (PD) who have motor fluctuations.

6. INVESTIGATIONAL PLAN

6.1. Overall Study Design

This is a multicenter, randomized, double-blind, double-dummy, active-controlled, parallel-group study. Subjects will continue to take permitted non-CD-LD-based PD medications throughout the study if documented in their prestudy regimen and if dosing regimens have been stable for at least 4 weeks prior to Visit 1. A "stable dosing regimen" means no change in dose or in dosing frequency. Within 4 weeks following the Screening visit, eligible subjects will complete their PD Diaries on each of the 3 consecutive days immediately prior to Visit 1.

Following Visit 1, qualified subjects will enter a 3-week, open-label IR CD-LD treatment period allowing for dose adjustment. During the IR CD-LD dose adjustment period, the initial dosing regimen of IR CD-LD will be the same as the subject's stable prestudy regimen unless the subject is taking a single daily bedtime dose of CR CD-LD, in which case, the CR CD-LD dose will be discontinued and substituted with a 1:1 milligram-equivalent dose of IR CD-LD. A "bedtime dose" is defined as the last daytime dose of CD-LD taken within 1 hour of onset of the subject's normal nighttime sleep period. **The dosing regimen of IR CD-LD may be adjusted during the dose adjustment period** to minimize "Off" time without causing troublesome dyskinesia. The doses and regimens of the subject's other non-CD-LD Parkinson's disease medications (dopamine agonists, MAO-B inhibitors, amantadine, anticholinergics) should remain stable throughout this study. Any adjustments to the IR CD-LD dosing regimen will be done in consultation with the Investigator or qualified site personnel and will be recorded. The subject must be on a stable dosing regimen (no change in dose or in dosing frequency) of IR CD-LD for at least 5 days prior to returning for Visit 2. Rescue with additional or modified doses of concomitant PD medications or with use of CD-LD products other than the dispensed study medications is not permitted and will trigger discontinuation from the study. Subjects will complete their 3-day PD Diaries on each of the 3 consecutive days immediately prior to Visit 1.

Following completion of the IR CD-LD dose adjustment period, subjects will begin a 4-week open-label period for conversion to IPX203. The initial dosing regimen of IPX203 during the conversion period will be based on the subject's dosing regimen of IR CD-LD at the end of the dose adjustment period (Visit 2) selecting the most frequent dose according to Table 3. A 25-100 mg dose of IR CD-LD converts to a 70-280 mg CD-LD dose of IPX203 but with a longer duration of effect. It is recommended that IPX203 should be dosed approximately every 8 hours with the exception that subjects who are currently receiving a total daily dose of less than 125-500 mg IR CD-LD at the end of the dose adjustment period will be initially administered IPX203 every 12 hours. The dosing interval may be reduced to approximately every 8 hours if the subject does not achieve an acceptable duration of effect. **The dosing regimen of IPX203 may be adjusted during the dose conversion period** to achieve the optimal balance of efficacy and tolerability (minimize "Off" time without causing troublesome dyskinesia or other dopaminergic side effects). Any adjustments to the IPX203 dosing regimen will be recorded. Rescue with additional or modified doses of concomitant PD medications or with use of CD-LD products other than the dispensed study medications is not permitted and will trigger discontinuation from the study. Subjects will return to the clinic in two weeks for Visit 3 followed by Visit 4 two weeks later. The subject must be on a stable dosing regimen of IPX203

(no change in dose or in dosing frequency) for at least 5 days prior to returning for Visit 4. Subjects will also be instructed to complete their 3-day PD Diaries on each of the 3 consecutive days immediately prior to Visit 4.

The study staff will call the subjects frequently (approximately every 1 to 3 days) during the IR CD-LD dose adjustment and IPX203 dose conversion periods. The calls are to ensure timely and appropriate dosing adjustments and to ensure that the subject is able to follow and adhere to the dosing instructions. The contacts may be less frequent after initial dose adjustments have been made. Any changes in the dosing regimen will be in consultation with the Investigator or qualified site personnel and will be documented.

Subjects who successfully complete the IPX203 dose conversion period will be randomized, stratified by center, in a 1:1 ratio at Visit 4 into one of two parallel treatment arms of IPX203 (with matching IR CD-LD placebo) or IR CD-LD (with matching IPX203 placebo). The subjects will undergo 13 weeks of double-blind, double-dummy maintenance therapy with the stable dosing regimen established at the end of Week 3 (Visit 2) for IR CD-LD and at the end of Week 7 (Visit 4) for IPX203. Subjects will return to the clinic for 3 visits (Visits 5, 6, and 7) and will be instructed to complete their 3-day PD Diaries on 3 consecutive days immediately prior to each of the 3 visits. Rescue with additional or modified doses of concomitant PD medications or use of CD-LD products other than the dispensed study medication is not permitted and will trigger discontinuation from the study.

Figure 1: Study Flow Chart

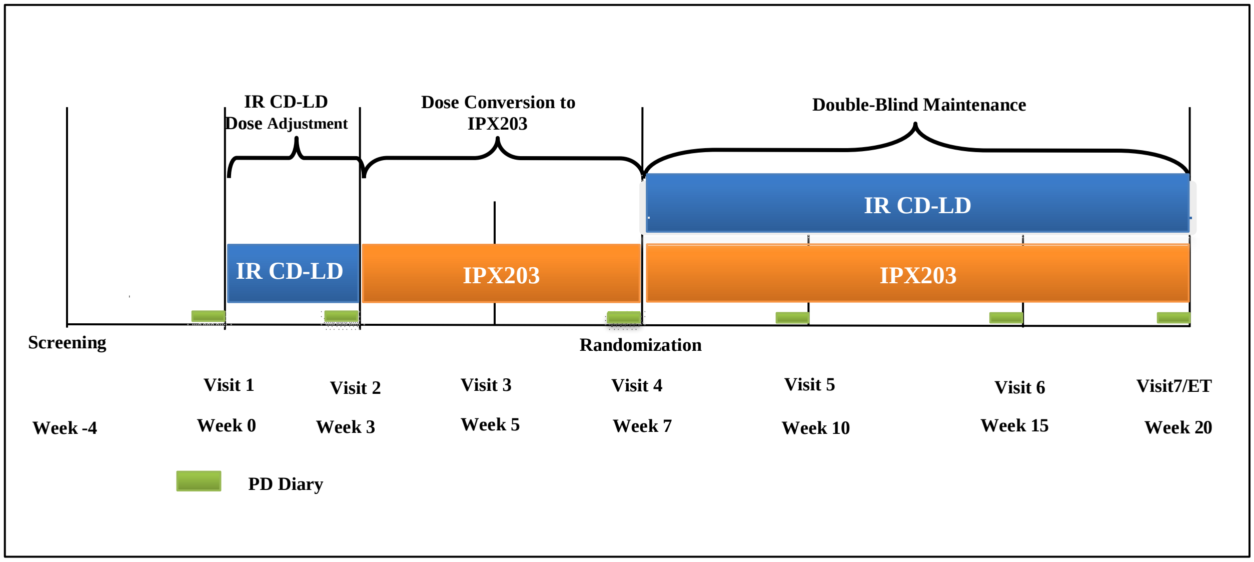

Abbreviations: IR=immediate-release, CD=carbidopa, LD=levodopa, ET=early termination

6.2. Number of Subjects

Assuming approximately an 18% prerandomization drop-out, approximately 510 subjects will be enrolled to randomize 420 subjects.

6.3. Treatment Assignment

Investigational product: IPX203 (carbidopa-levodopa) Extended-Release Capsules containing 35-140 mg of CD-LD, for oral administration.

Reference therapy: Immediate-Release carbidopa-levodopa (IR CD-LD) tablets containing 25-100 mg of CD-LD, for oral administration.

Subjects will be randomly assigned to one of two parallel treatment arms to receive either IPX203 (with matching IR CD-LD placebo) or IR CD-LD (with matching IPX203 placebo) during the double-blind maintenance therapy portion of the study.

6.4. Dosing and Dose Determination Criteria

IR CD-LD will be supplied as tablets containing 25-100 mg of CD-LD. IR CD-LD tablets may be split to achieve the required dose.

IPX203 will be supplied as capsules containing 35-140 mg of CD-LD. The suggested doses and regimen of IPX203 are intended to provide an onset of effect comparable to the subject's prestudy IR LD regimen and to extend the duration of effect. A 25-100 mg dose of IR CD-LD converts to a 70-280 mg CD-LD dose of IPX203.

6.4.1. IR CD-LD Dose Adjustment Period

During the 3-week IR CD-LD dose adjustment period, the initial dosing regimen of IR CD-LD will be the same as the subject's stable prestudy regimen unless the subject is taking a single daily bedtime dose of CR CD-LD, in which case, the CR CD-LD dose will be discontinued and substituted with a 1:1 milligram-equivalent dose of IR CD-LD. A "bedtime dose" is defined as the last daytime dose of CD-LD taken within 1 hour of onset of the subject's normal nighttime sleep period. Subjects who were receiving IR CD-LD as a 1:10 CD-LD formulation will be started on IR CD-LD with a 1:4 ratio at the same frequency and LD dose. **The dosing regimen of IR CD-LD may be adjusted during the dose adjustment period** to minimize "Off" time without causing troublesome dyskinesia. The doses and regimens of the subject's other non-CD-LD Parkinson's disease medications (dopamine agonists, MAO-B inhibitors, amantadine, anticholinergics) should remain stable throughout this study. Any adjustments to the IR CD-LD dosing regimen will be done in consultation with the Investigator or qualified site personnel and will be recorded. The subject must be on a stable dosing regimen (no change in dose or in dosing frequency) of IR CD-LD for at least 5 days prior to returning for Visit 2.

6.4.2. IPX203 Dose Conversion Period

During the 4-week IPX203 dose conversion period, the suggested initial dosing regimen of IPX203 will be based on the most frequent dose of the subject's dosing regimen of IR CD-LD at the end of the dose adjustment period (Visit 2). A 25-100 mg dose of IR CD-LD converts to a 70-280 mg CD-LD dose of IPX203, and a half tablet (12.5-50 mg dose of IR CD-LD) converts to a 35-140 mg CD-LD dose of IPX203. To facilitate conversion of subjects from IR CD-LD to IPX203, Table 3 presents recommended starting dose regimens. It is recommended that IPX203 should be dosed approximately every 8 hours with the exception that subjects who are currently receiving a total daily dose of less than 125-500 mg IR CD-LD at the end of the dose adjustment

period will be initially administered IPX203 every 12 hours. The dosing interval may be reduced to approximately every 8 hours if the subject does not achieve an acceptable duration of effect. **The dosing regimen of IPX203 may be adjusted during the dose conversion period** to achieve the optimal balance of efficacy and tolerability (minimize “Off” time without causing troublesome dyskinesia or other dopaminergic side effects). The doses and regimens of the subject’s other non-CD-LD Parkinson’s disease medications (dopamine agonists, MAO-B inhibitors, amantadine, anticholinergics) should remain stable throughout this study. The subject must be on a stable dosing regimen of IPX203 (no change in dose or in dosing frequency) for at least 5 days prior to returning for Visit 4.

Table 3: Recommended Starting IPX203 LD Dosing Regimen Based on the Dosing Regimen of IR CD-LD at the End of the Dose Adjustment Period

Most Frequent IR CD-LD Unit Dose (mg)	Recommended Starting IPX203 Daily Dosing Regimen CD-LD (mg) Every 8 Hours
25-100	70-280 mg (2 × 35-140 mg)
>25-100 – 37.5-150	105-420 mg (3 × 35-140 mg)
>37.5-150 – 50-200	140-560 mg (4 × 35-140 mg)
>50-200	175-700 mg (5 × 35-140 mg)

[a] Subjects who are on a total daily dose of less than 125-500 mg CD-LD from IR CD-LD should be advised to initially take IPX203 every 12 hours. The dosing interval may be reduced to approximately every 8 hours if the subject does not achieve an acceptable duration of effect.

During the dose conversion to IPX203, the Investigator or site staff are advised to be in frequent contact (every 1 to 3 days) with the subject especially during the initial dose conversion to assess the need for dosage adjustment with the goal of minimizing “Off” time without causing troublesome dyskinesia. Any changes to the dosing regimen should only be made by the Investigator or qualified site personnel. If the subject experiences troublesome dyskinesias during initial dose conversion, consider reducing the dose by one capsule (35-140 mg IPX203 CD-LD) before increasing the dosing interval. If turning “On” is slow following the first morning dose, consider taking the morning dose in the fasted state and/or increasing the dose by one capsule (35-140 mg IPX203 CD-LD). If turning “On” is slow later in the day or to reduce “end-of-dose” “Off” time, consider increasing the dose by one capsule (35-140 mg IPX203 CD-LD).

When two or more IR CD-LD doses correspond to the most frequent IR CD-LD dose, the suggested IPX203 conversion should be based on the higher of the IR CD-LD doses.

A summary of the instructions for dose conversion to IPX203 is provided in Appendix B.

6.4.3. Double-Blind Maintenance Period

During the 13-week double-blind double-dummy maintenance period, subjects receive either IPX203 (with matching IR CD-LD placebo) or IR CD-LD (with matching IPX203 placebo) with

the stable dosing regimen established at the end of Week 3 (Visit 2) for IR CD-LD and at the end of Week 7 (Visit 4) for IPX203.

6.5. Criteria for Study Termination

The Sponsor has the right to terminate this study and remove all study material from the study site at any time for medical or administrative reasons. The Sponsor will endeavor to give adequate notice to allow safe withdrawal of subjects from the study.

7. SELECTION AND WITHDRAWAL OF SUBJECTS

Each subject must meet all of the following inclusion and exclusion criteria to qualify for enrollment.

7.1. Subject Inclusion Criteria

1. Male or female subjects diagnosed at age ≥ 40 years with PD, consistent with the United Kingdom Parkinson's Disease Society Brain Bank Diagnostic Criteria (Appendix C) and who are being treated with stable regimens of CD-LD but experiencing motor fluctuations.
2. Able to provide written informed consent prior to the conduct of any study-specific procedures.
3. Female subjects of childbearing potential must have a negative urine pregnancy test at Screening Visit.
4. Negative urine screen for drugs of abuse and negative alcohol breath test at Screening.
5. Hoehn and Yahr Stages 1, 2, 3, or 4 in the "On" state (part of Movement Disorders Society version of the Unified Parkinson's Disease Rating Scale [MDS-UPDRS] Part III; Appendix E).
6. Agrees to use a medically acceptable method of contraception throughout the study and for 6 weeks after completing the study. Medically acceptable methods of contraception that may be used by the subject and/or partner include but are not limited to: abstinence, oral contraception, NuvaRing or transdermal systems, diaphragm with vaginal spermicide, intrauterine device, condom and partner using vaginal spermicide, surgical sterilization (6 months), progestin implant or injection, or postmenopausal female (no menstrual period for > 2 years) or vasectomy (> 6 months).
7. Montreal Cognitive Assessment (MoCA) score ≥ 24 at Screening Visit in "On" state (Appendix D).
8. By history, for the 4 weeks prior to Screening, the subject experiences daily "wearing-off" episodes with periods of bradykinesia in combination with at least one of rest tremor or rigidity, experiences an "Off" state upon awakening on most mornings, and reports an average of at least 2.5 cumulative hours per day of "Off" time during the waking hours.
9. Able to differentiate "On" state from "Off" state as determined by at least 75% concordance with a trained rater in "On/Off" ratings for 8 ratings over the 4-hour training period. The concordance must include at least 1 "On" and 1 "Off" rating and must be achieved within two 4-hour training sessions.
10. At Visit 1, review of the 3-day PD Diaries confirms the following: that the subject is able to properly complete the diaries with valid entries; and that the subject has an <u>average of at least 2.5 hours</u> per day of "Off" time during the waking hours over the 3 days with at least 1.5 hours of cumulative "Off" time on each day. Inability to properly complete the

diaries is indicated when more than 1 day of a diary is not returned or when more than 2 hours (4 half-hour periods) of one 24-hour diary day are missing (Appendix P).

11. Responsive to CD-LD therapy and currently being treated on a stable regimen with CD-LD for at least 4 weeks prior to Visit 1 and:
    a. Requires at least 100 mg of LD from IR CD-LD for the first morning dose
    b. Requires a total daily dose of at least 400 mg of LD and takes a maximum total daily dose of 2400 mg LD, from IR CD-LD alone or IR CD-LD in combination with a single daily bedtime dose of CR CD-LD
    c. Has a dosing frequency of 4 to 9 times daily of CD-LD
    d. By history, typically experiences an "On" response with the first dose of IR CD-LD of the day, but the efficacy of this dose typically lasts less than 4 hours.
12. At Screening, the subject has predictable "Off" periods defined by a score of 1 or 2 on Item #4.5 (Complexity of Motor Fluctuations) of the MDS-UPDRS Part IV B (Motor Fluctuations) (Appendix E).
13. At Screening, the MDS-UPDRS Part III total score in the "Off" state is at least 20 units.
14. Able and willing to comply with the protocol, including completion of diaries and availability for all study visits.

7.2. Subject Exclusion Criteria

1. Used any doses of controlled-release (CR) CD-LD apart from a single daily bedtime dose within 4 weeks prior to Visit 1.
2. Used any doses of Rytary for the past 4 weeks prior to Visit 1 or considered IPX066 or Rytary failures for reasons of efficacy or safety.
3. Received any investigational medications within 30 days or 5 times the half-life, whichever is longer, prior to Visit 1.
4. Female subjects who are currently breastfeeding or lactating.
5. Had prior neurosurgical treatment for PD or if such procedure is planned or anticipated during the study period.
6. Allergic to any excipient in the study drugs (See Appendix Q).
7. History of medical conditions or of a prior surgical procedure that would interfere with LD absorption, such as gastrectomy, proximal small-bowel resection, or bariatric surgery.
8. History of upper gastrointestinal hemorrhage in patients with peptic ulcer disease within the past 5 years.
9. History of glaucoma with intraocular pressures that are elevated despite appropriate medical management.
10. History of seizure or epilepsy and experienced at least 1 seizure during the past 12 months or has not been compliant with medically recommended therapy or visits.
11. History of myocardial infarction with residual atrial, nodal, or ventricular arrhythmias that are not controlled with medical and/or surgical interventions. A recent

(≤ 12 months) history of myocardial infarction with secondary arrhythmias is exclusionary regardless of the therapeutic control.

12. History of neuroleptic malignant syndrome or of nontraumatic rhabdomyolysis.
13. Liver enzyme values ≥ 2.5 times the upper limit of normal; or history of severe hepatic impairment.
14. Serum creatinine level ≥ 1.75 times the upper limit of normal; or requires dialysis at the time of Screening.
15. Subject with a history of malignant melanoma or with a suspicious undiagnosed skin lesion which in the opinion of the investigator could be melanoma.
16. History of drug or alcohol abuse within the 12 months prior to Screening.
17. Received within 4 weeks of Visit 1 or planning to take during participation in the clinical study:
    a. any doses of a controlled-release (CR) LD apart from a single daily bedtime dose, any doses of Rytary, additional CD (eg, Lodosyn) or benserazide (eg, Serazide), or catechol-O-methyl transferase inhibitors (entacapone or tolcapone) or medications containing these inhibitors (Stalevo),
    b. nonselective monoamine oxidase inhibitors (MAOI), apomorphine, or antidopaminergic agents, including antiemetics.
18. Treatment with any dopamine antagonist for the purposes of treating psychosis or bipolar disorder within the last 2 years or any history of psychosis within the past 10 years regardless of treatment. A brief, self-limited episode of psychosis precipitated by a medical intervention with return to normal mentation is not exclusionary. Mild PD-associated illusions are not exclusionary provided that they do not occur more than twice per week and the subject does not lose insight.
19. Employees or family members of the investigator, study site, or sponsor.
20. Subjects who have previously participated in an IPX203 study.
21. Subjects who, in the opinion of the clinical investigator, should not participate in the study.
22. Based on clinical assessment, subject does not adequately comprehend the terminology needed to complete the PD diary.

7.3. Subject Withdrawal Criteria

Site personnel should make every effort to conduct all protocol-specific procedures to complete the study. A subject may be discontinued from the study due to the following reasons:

1. Withdrawal by subject
2. Adverse event (AE)
3. Lack of efficacy
4. Study terminated by Sponsor

5. Protocol deviation
6. Noncompliance with study drug
7. Lost to follow-up
8. Death
9. Other

Subjects who withdraw early from the study will not be replaced. The reason or reasons for discontinuation will be specified and documented. Empty medication bottles and any unused study drug upon discontinuation will be collected. Study medication dispensed to a discontinued subject may not be redispensed to a different subject.

8. STUDY PROCEDURES

The procedures to be performed at each study visit are described below and summarized in Table 4.

Table 4: Events Schedule for Impax Study IPX203-B16-02

Assessment	Screening	3 Weeks of IR CD-LD Dose Adjustment	4 Weeks of IPX203 Dose Conversion			13 Weeks of Double-Blind Therapy		
		Visit 1	Visit 2	Visit 3	Visit 4 Randomization	Visit 5	Visit 6	Visit 7 / Study Exit/Early Termination[b]
Study Week[a]	**–4**	**0**	**3**	**5**	**7**	**10**	**15**	**20**
ICF & HIPAA Authorization[c]	X							
Contact IWRS	X	X	X	X	X	X	X	X
Randomization					X			
Inclusion/Exclusion	X	X						
Medical History	X							
Physical Examination	X							X
Vital Signs[d]	X	X	X	X	X	X	X	X
Height and Weight	X					X[e]		X[e]
C-SSRS[f]	X	X	X	X	X	X	X	X
Clinical Laboratory Tests[g]	X					X		X
Urine Pregnancy Test	X							
Urine Screen for Drug Abuse	X							
Alcohol Breath Test	X							
ECG	X					X		X
MoCA[h]	X							
MDS-UPDRS Parts I-IV	X[i]	X	X		X	X	X	X
PGI-C[j]						X	X	X
CGI-C[k]						X	X	X
PGI-S[l]		X			X			X

Assessment	Screening	3 Weeks of IR CD-LD Dose Adjustment	4 Weeks of IPX203 Dose Conversion			13 Weeks of Double-Blind Therapy		
		Visit 1	Visit 2	Visit 3	Visit 4 Randomization	Visit 5	Visit 6	Visit 7 / Study Exit/Early Termination[b]
Study Week[a]	**–4**	**0**	**3**	**5**	**7**	**10**	**15**	**20**
CGI-S[m]		X			X			X
PDQ-39[n]		X			X		X	X
GCSI[o]		X						X
NMSS[p]		X			X		X	X
PDSS-2[q]		X			X		X	X
PAS[r]		X			X		X	X
PD Diary Training; Perform Concordance Testing at Screening Only[s]	X	X	X	X	X	X	X	
Dispense PD Diaries[t]	X	X		X	X	X	X	
Review PD Diaries[u]		X	X		X	X	X	X
Reminder phone calls[v,w]	X[v]	X[w]	X[w]	X[w]	X[w]	X	X	X
Dispense study medication		X	X	X	X	X	X	
Collect empty medication bottles and any unused study drug/Perform study drug accountability			X	X	X	X	X	X
Adverse Events	X	X	X	X	X	X	X	X
Concomitant Medications	X	X	X	X	X	X	X	X

CGI-C = Clinical Global Impression of Change, CGI-S = Clinical Global Impression of Severity, C-SSRS = Columbia-Suicide Severity Rating Scale, ECG = electrocardiogram, GCSI = Gastroparesis Cardinal Symptom Index, HIPAA = Health Insurance Portability and Accountability Act, ICF = informed consent form, IWRS = interactive web response system, MDS-UPDRS = MDS version of Unified Parkinson's Disease Rating Scale, MoCA = Montreal Cognitive Assessment, PAS = Parkinson Anxiety Scale, PD = Parkinson's disease, NMSS = Non-Motor Symptom assessment scale for PD, PDQ-39 = 39-Item Parkinson's Disease Questionnaire, PDSS-2 = Parkinson's Disease Sleep Scale-2, PGI-C = Patient Global Impression of Change, PGI-S = Patient Global Impressions of Severity.

[a] The interval between Screening and Visit 1 (Day 1) should not exceed 4 weeks. Study visits should occur within ± 3 days of their specified timing.

[b] Study Exit procedures to be conducted at the end of Visit 7 or during an early termination visit.
[c] Subjects enrolled at sites in the United States (US) must sign HIPAA authorization prior to the conduct of any study-specific procedures.
[d] Record vital signs (blood pressure, heart rate, respiratory rate, and temperature [Screening and Study Exit only]) after subject has been resting supine for at least 5 minutes, then record orthostatic blood pressure and heart rate after subject has been standing for approximately 2 minutes. At Visits 1 and 4, orthostatic vital signs (blood pressure and heart rate) will be performed in triplicate, each set separated by at least 15 minutes from the previous set.
[e] Weight only.
[f] C-SSRS: Columbia Suicide Severity Rating Scale. See Appendix O.
[g] See Appendix R.
[h] Montreal Cognitive Assessment in the "On" state: see Appendix D.
[i] At Screening MDS-UPDRS Parts I through IV will be done in both the "On" and "Off" state (see Appendix E).
[j] See Appendix F.
[k] See Appendix G.
[l] See Appendix H.
[m] See Appendix I.
[n] See Appendix J.
[o] See Appendix K.
[p] See Appendix L.
[q] See Appendix M.
[r] See Appendix N.
[s] Train at Screening and then as needed at subsequent visits. Perform concordance testing at Screening.
[t] Dispense PD Diaries at Screening and Visits 1, 3, 4, 5, and 6. Call subjects 4 days prior to Visits 1, 2 and 4-7 to remind them to complete PD Diaries. Subjects record diary information for 3 consecutive days immediately prior to each of the visits (Days -3, -2, and -1). Call subjects the day prior to each visit to remind them to bring the PD Diaries, empty medication bottles, and any unused study drug to the office.
[u] Review PD Diaries at Visits 1, 2, and 4-7.
[v] Post-Screening reminder phone call: Notify individuals who successfully complete screening procedures following review of all study entry criteria and clinical laboratory results that they may continue in the study. The interval between Screening and Visit 1 should not exceed 4 weeks.
[w] Reminder phone calls for Visits 1 through 4: In addition to the calls discussed above, make regular phone calls (approximately every 1 to 3 days) to subjects throughout the IR CD-LD dose-adjustment and IPX203 dose-conversion periods to evaluate each subject's adjustment to the study medication regimen.

8.1. Screening Visit

After the subject has signed the informed consent (and HIPAA authorization for US subjects only), complete the following procedures and assessments:

- Obtain an identification number from the Interactive Web Response System (IWRS). The IWRS will assign a 6-digit ID number to each subject, consisting of a 3-digit number representing the investigative site and a 3-digit sequential subject number.
- Review and record study entry criteria (Section 7).
- Perform urine pregnancy test for females of childbearing potential.
- Perform urine screen for drugs of abuse.
- Perform alcohol breath test.
- Complete medical history.
- Perform physical examination, including height and weight.
- Assess vital signs after subject is supine for at least 5 minutes (blood pressure, heart rate, temperature and respiratory rate) and then assess orthostatic blood pressure and heart rate after subject is standing (for approximately 2 minutes).
- Record current CD-LD regimen, other PD medications and their dosing schedule, and other concomitant medications.
- Record AEs.
- Perform a 12-lead ECG.
- Administer C-SSRS (Appendix O).
- Determine MoCA Score in the “On” state (Appendix D).
- Collect blood and urine samples for clinical laboratory studies (Appendix R).
- Determine Hoehn and Yahr staging of PD in the “On” state (part of MDS-UPDRS Part III Motor Examination) (Appendix E).
- Administer MDS-UPDRS Parts I through IV in the “On” and “Off” state (Appendix E).
- Train the subject how to complete the PD Diaries to assess his/her “On” and “Off” states, including assessment of any dyskinesia. After training the subject, perform the concordance testing. The subject’s “On”/”Off” ratings must agree at least 75% of the time with the trained rater during the training sessions. That is, the subject’s “On”/”Off” ratings must agree with the trained rater’s ratings on at least 75% “On”/“Off” states in a single session to qualify for study inclusion. The 75% concordance rate must be based on at least four ratings, and must include at least one “On” and one “Off” state. The ratings should occur every 30 minutes and each session can last up to 4 hours. If the subject fails the first training session, the subject may be trained for one additional 4-hour training session.

- Dispense PD Diaries and instruct the subject to complete the PD Diaries on 3 consecutive days immediately prior to Visit 1.

Notify individuals who successfully complete screening procedures following review of all study entry criteria and clinical laboratory results.

The interval between Screening and Visit 1 (Day 1) should not exceed 4 weeks.

8.2. Visit 1 – Start of IR CD-LD Dose Adjustment

8.2.1. Prior to Visit 1

Contact the subject at least 4 days prior to Visit 1 to remind him/her to complete the 3-day PD Diaries starting 3 consecutive days immediately prior to Visit 1.

The day prior to Visit 1, remind subjects to:

- Bring their completed 3-day PD Diaries to the clinic.

8.2.2. At Visit 1

For Visit 1 complete the following procedures:

- Collect and review the subject's 3-day PD Diaries. Ensure that the subject is averaging at least 2.5 hours per day of "Off" time over 3 days and at least 1.5 hours of "Off" time on each day based on the 3 day PD Diaries. If the subject cannot properly complete the diary, eg, if more than 1 day of the diary is not returned or if more than 1 day of the diary is not valid (ie, more than 2 hours [4 half periods] of the 24-hour diary are missing), he/she will not continue in the study.
- Review inclusion and exclusion criteria to ensure that the subject continues to meet study inclusion criteria.
- Review instruction of 3-day PD Diaries if needed.
- Measure vital signs (respiratory rate, blood pressure, and heart rate after supine for at least 5 minutes, and orthostatic pulse and blood pressure after standing for approximately 2 minutes). Orthostatic vital sign measurements (blood pressure and heart rate) will be performed in triplicate, each set separated by at least 15 minutes from the previous set.
- Administer C-SSRS (Appendix O).
- Administer MDS-UPDRS Parts I through IV (Appendix E).
- Complete PDQ-39 (Appendix J).
- Complete PGI-S (Appendix H).
- Complete CGI-S (Appendix I).
- Complete GCSI (Appendix K).
- Complete Non-motor Symptom assessment scale for PD (NMSS) (Appendix L).

- Complete the Parkinson's Disease Sleep Scale-2 (PDSS-2) (Appendix M).
- Complete Parkinson Anxiety Scale (PAS) (Appendix N).
- Record any AEs and update changes in concomitant medication since the previous visit.
- Dispense PD Diaries.
- Contact IWRS and dispense study medication per IWRS instructions.

8.2.3. Post Visit 1

- Make regular phone calls (approximately every 1 to 3 days) while the IR CD-LD dose is being adjusted. The IR CD-LD dosing regimen should be stable for at least 5 days prior to returning for Visit 2.

8.3. Visits 2 (Week 3) and Visit 3 (Week 5) – IPX203 Dose Conversion

8.3.1. Prior to Visit 2

- Call subjects 4 days prior to Visit 2 and remind them to complete their PD Diaries.
- Call subjects the day prior to Visit 2 to remind them to bring the PD Diaries, empty medication bottles, and any unused study drug to the office.

8.3.2. Prior to Visit 3

- Call subjects the day prior to Visit 3 to remind them to bring back empty medication bottles and any unused study drug to the office.

8.3.3. At Visits 2 and 3

For Visits 2 and 3 complete the following procedures:

- Measure vital signs (respiratory rate, blood pressure, and heart rate after supine for at least 5 minutes, and orthostatic pulse and blood pressure after standing for approximately 2 minutes).
- Administer C-SSRS (Appendix O).
- Collect empty medication bottles and any unused study drug; perform study drug accountability.
- Record and update AEs and concomitant medications.
- Contact IWRS and dispense study medication per IWRS instructions.

<u>Additional Assessments at Visit 2 Only</u>

- Administer MDS-UPDRS Parts I through IV (Appendix E).
- Review PD Diaries. The subject will be terminated from the study if the subject does not average at least 2.5 hours per day of "Off" time over 3 days and at least 1.5 hours of "Off" time on each day based on the 3 day PD Diaries and/or if the subject cannot

properly complete the diary, eg, if more than 1 day of the diary is not returned or if more than 1 day of the diary is not valid (ie, more than 2 hours [4 half periods] of the 24 hour diary are missing).

- Ensure that the subject has been on a stable dosing regimen for at least 5 days prior to dose conversion to IPX203. If the subject is not able to achieve a stable dosing regimen lasting at least 5 days, the subject will be discontinued.
- Conduct PD Diaries training, if needed.

Additional Activities at Visit 3 Only

- Dispense PD Diaries.
- Review instruction of 3-day PD Diaries if needed.

8.3.4. Post Visits 2 and 3

Make regular phone calls (approximately every 1 to 3 days) to subjects throughout the dose conversion period, as needed, to evaluate each subject's adjustment to the study medication. The IPX203 dosing regimen should be stable for at least 5 days prior to returning for Visit 4.

8.4. Visit 4 (Week 7) – Randomization

8.4.1. Prior to Visit 4

- Call subjects 4 days prior to Visit 4 to remind them to complete their PD Diaries.
- Contact subjects 1 day prior to Visit 4 to remind them to bring back the PD Diaries, empty medication bottles, and any unused study drug to the office.

8.4.2. At Visit 4

For Visit 4 complete the following procedures:

- Review PD Diaries. At least 1 day of valid diary data (ie, less than 2 hours [4 half periods] of the 24-hour diary are missing) must be available, otherwise the subject will be terminated from the study.
- Ensure that the subject has been on a stable dosing regimen for at least 5 days prior to randomization. If the subject is not able to achieve a stable dosing regimen lasting at least 5 days, the subject will be discontinued.
- Measure vital signs (respiratory rate, blood pressure, and heart rate after supine for at least 5 minutes, and orthostatic pulse and blood pressure after standing for approximately 2 minutes). Orthostatic vital sign measurements (blood pressure and heart rate) will be performed in triplicate, each set separated by at least 15 minutes from the previous set.
- Administer C-SSRS (Appendix O).
- Administer MDS-UPDRS Parts I through IV (Appendix E).
- Complete PDQ-39 (Appendix J).

- Complete PGI-S (Appendix H).
- Complete CGI-S (Appendix I).
- Complete NMSS (Appendix L).
- Complete PDSS-2 (Appendix M).
- Complete PAS (Appendix N).
- Record and update AEs and concomitant medications.
- Conduct PD diaries training, if needed.
- Collect empty medication bottles and any unused study drug; perform study drug accountability.
- Contact IWRS for randomization and dispense medication per IWRS instructions.
- Dispense PD diaries.

8.5. Visit 5 (Week 10) and Visit 6 (Week 15)

8.5.1. Prior to Visit 5 and 6

- Call subjects 4 days prior to Visits 5 and 6 to remind them to begin recording in their PD diaries on each of the 3 consecutive days immediately prior to each of these visits.
- Call the subjects the day prior to Visits 5 and 6 to remind the subjects to bring in their PD diaries, empty medication bottles, and any unused study drug to the office.

8.5.2. At Visit 5 and 6

For Visits 5 and 6 complete the following procedures (note visit-specific tasks below):

- Measure vital signs (respiratory rate, blood pressure, and heart rate after supine for at least 5 minutes, and orthostatic pulse and blood pressure after standing for approximately 2 minutes).
- Administer C-SSRS (Appendix O).
- Administer MDS-UPDRS Parts I through IV (Appendix E).
- Complete PGI-C (Appendix F).
- Complete CGI-C (Appendix G).
- Review PD diaries.
- Conduct PD diaries training, if needed.
- Dispense PD diaries.
- Collect empty medication bottles and any unused study drug; perform study drug accountability.
- Contact IWRS and dispense study medication per IWRS instructions.

- Record any AEs and update changes in concomitant medication since the previous visit.

Additional Activities at Visit 5 Only:

- Record weight.
- Perform a 12-lead ECG.
- Collect blood and urine samples for clinical laboratory studies (Appendix R).

Additional Activities at Visit 6 Only:

- Complete PDQ-39 questionnaire (Appendix J).
- Complete NMSS (Appendix L).
- Complete the PDSS-2 (Appendix M).
- Complete PAS (Appendix N).

8.6. Visit 7 (Week 20) – End of Study/Study Exit

8.6.1. Prior to Visit 7

- Call subjects 4 days prior to Visit 7 to remind them to begin recording in their PD Diaries on each of the 3 consecutive days immediately prior to Visit 7.
- Call the subjects the day prior to Visit 7 to remind the subjects to bring in their PD Diaries, empty medication bottles, and any unused study drug to the office.

8.6.2. At Visit 7

All enrolled subjects must complete Study Exit procedures at the end of Visit 7 or during an early termination visit:

- Perform physical examination, including weight.
- Measure vital signs (temperature, respiratory rate, blood pressure, and heart rate after supine for at least 5 minutes, and orthostatic pulse and blood pressure after standing for approximately 2 minutes).
- Collect and review 3-day PD Diaries.
- Administer C-SSRS (Appendix O).
- Administer MDS-UPDRS Parts I through IV (Appendix E).
- Complete PGI-C (Appendix F).
- Complete CGI-C (Appendix G).
- Complete PDQ-39 questionnaire (Appendix J).
- Complete NMSS (Appendix L).
- Complete PDSS-2 (Appendix M).

- Complete PAS (Appendix N).
- Complete PGI-S (Appendix H).
- Complete CGI-S (Appendix I).
- Complete GCSI (Appendix K).
- Collect empty medication bottles and any unused study drug; perform study drug accountability.
- Record and update AEs and concomitant medications.
- Collect blood and urine samples for clinical laboratory studies (Appendix R).
- Perform a 12-lead ECG.
- Contact IWRS to record subject disposition.

8.7. Early Termination

8.7.1. Subjects Who Terminate Prior to Randomization

If the subject discontinues the study prior to randomization (Visit 4) the subject should complete the following assessments:

- Perform physical examination, including weight.
- Measure vital signs (temperature, respiratory rate, blood pressure, and heart rate after supine for at least 5 minutes, and orthostatic pulse and blood pressure after standing for approximately 2 minutes).
- Collect and review 3-day PD Diaries when available.
- Administer C-SSRS (Appendix O).
- Administer MDS-UPDRS Parts I through IV (Appendix E).
- Collect empty medication bottles and any unused study drug; perform study drug accountability.
- Record and update AEs and concomitant medications.
- Collect blood and urine samples for clinical laboratory studies (Appendix R).
- Perform a 12-lead ECG.
- Contact IWRS to record subject disposition.

8.7.2. Subjects Who Terminate Early after Randomization

If the subject discontinues the study after randomization (Visit 4), the subject should complete all assessments described in Section 8.6.2.

8.8. Blood Volume

Safety blood draws: Approximately 10 mL of blood will be drawn at Screening, Visit 5, and at Study Exit, for a combined total of 30 mL.

9. TREATMENT OF SUBJECTS

9.1. Description of Study Drug

Study drugs will be provided by Impax for this study:

- IPX203 (carbidopa-levodopa) Extended-Release Capsules containing 35-140 mg of CD-LD for oral administration. The CD-LD ratio is 1:4. In addition, matching placebo capsules will also be provided.
- Immediate-release carbidopa-levodopa (IR CD-LD) tablet containing 25-100 mg of CD-LD, for oral administration. In addition, matching placebo tablets will also be provided.

Table 5: Study Drugs for Study IPX203-B16-02

Investigational Product	Dosage Strength (mg CD-LD) and Form
IPX203 (carbidopa-levodopa) Extended-Release capsules	35-140 mg<br>Capsules for oral administration
IR CD-LD (carbidopa-levodopa) tablets	25-100 mg<br>Tablets for oral administration
IPX203 Placebo capsules	Capsules for oral administration
IR CD-LD Placebo tablets	Tablets for oral administration

9.2. Concomitant Medications

9.2.1. Permitted PD Medications

Concomitant therapy with amantadine, selective monoamine oxidase (MAO) type B inhibitors (eg, selegiline, rasagiline), anticholinergic PD medications (eg, benztropine, trihexyphenidyl), and/or dopamine agonists (except apomorphine) is allowed provided the doses and regimens have been stable for at least 4 weeks prior to Visit 1 and the therapy is intended to be constant throughout the course of the study.

9.2.2. Prohibited Medications and Procedures

Prohibited medications and procedures include the following:

- Received within 4 weeks of Visit 1 or planning to take during participation in the clinical study: any doses of a controlled-release (CR) CD-LD apart from a single daily bedtime dose or any doses of Rytary, additional CD (eg, Lodosyn) or benserazide (eg, Serazide), or catechol-O-methyl transferase inhibitors (entacapone or tolcapone) or medications containing these inhibitors (Stalevo).

- Rescue with additional or modified doses of concomitant PD medications or with use of CD-LD products other than the dispensed study medications is not permitted and will trigger discontinuation from the study.
- Received within 4 weeks of Visit 1 or planning to take during participation in the clinical study: nonselective MAOI, selective MAO type A inhibitor (eg, phenelzine, moclobemide, pirlindole, bifemelane, toloxatone), apomorphine, or antidopaminergic agents including antiemetics.
- Treatment with any dopamine antagonist antipsychotic agents for the purposes of psychosis or bipolar disorder within the last 2 years. Use of antipsychotics to treat conditions other than psychosis or bipolar disorders may be allowed only after consultation with the medical monitor.
- Any neurosurgical procedure for the treatment of PD during the course of the study.

A subject who reports the use of any prohibited medications or procedure will be discontinued.

All medications taken within 30 days prior to signing the informed consent form (ICF) and all concomitant medications taken during the study will be recorded on the case report form (CRF).

9.2.3. Rescue Medications

Rescue with additional or modified doses of concomitant non-CD-LD PD medications is not permitted and will trigger discontinuation from the study. During the dose adjustment and dose conversion periods, rescue with CD-LD products other than the dispensed study medications is not permitted and will trigger discontinuation from the study. No medication adjustments are allowed following randomization and during the double-blind phase of the study and will trigger discontinuation from the study.

9.3. Treatment Compliance

Study drug accountability and reconciliation will be performed by the study staff and the study monitor(s).

9.4. Randomization and Blinding

At Visit 4, subjects will be randomized, stratified by center, in a 1:1 ratio into one of two double-blind parallel treatment arms of IPX203 (and matching IR CD-LD placebo) or IR CD-LD (and matching IPX203 placebo).

10. STUDY DRUG MATERIALS AND MANAGEMENT

10.1. Study Drug

Study drugs include the investigational treatment IPX203 35-140 mg CD-LD (and matching placebo capsules) and the active comparator treatment IR 25-100 mg CD-LD, (and matching placebo tablets).

IPX203 is an extended-release (ER) capsule formulation of CD-LD. Impax will manufacture and provide the IPX203 and matching placebo.

IR CD-LD is commercially available and will be provided by Impax. Matching placebo tablets will be manufactured and provided by Impax.

10.2. Study Drug Packaging and Labeling

Impax or designee will provide study medications in bottles with appropriate labeling affixed.

Labels on the study medication may include the following information:

- name, address, and phone number of the sponsor
- pharmaceutical dosage form/route of administration, quantity of dosage units, the name/identifier, and strength/potency
- batch and/or code number to identify the contents and packaging operation
- trial reference code (protocol number)
- trial subject identification number/treatment number and where relevant, the visit number
- name of investigator
- directions for use: Take tablet(s) or capsule(s) orally with water as directed.
- for clinical trial use only
- storage information: Store at 25°C (77°F), with excursions permitted to 15°C to 30°C (59°F to 86°F). Protect from light and moisture.
- period of use (use-by date, expiry date or retest date as applicable), in month/year format and in a manner that avoids any ambiguity.
- keep out of reach of children
- caution statement: Caution: New Drug—Limited by Federal (or United States) law to investigational use.

10.3. Study Drug Storage

The clinical site should store the study drug at 25°C (77°F), with excursions permitted to 15°C to 30°C (59°F to 86°F). The study drug should be stored in a tightly closed container, protected

from light and moisture. Storage temperature excursions above 30°C (86°F) should be reported by the clinical site to Impax or its designee.

10.4. Study Drug Administration

Subjects will be instructed to take their medications with approximately 240 mL of room-temperature water. The capsules or tablets should not be crushed or chewed.

IR CD-LD tablets may be split to achieve the required doses.

10.5. Study Drug Dispensing and Accountability

The Investigator must ensure that all study medication received at the study site is inventoried and accounted for, and that dispensed study medication is recorded in the subject's source documents, the CRF, and the study medication inventory log. Site personnel must not relabel or reassign study medication to other subjects or to individuals not enrolled in the study. The study monitor verifies medication accountability during monitoring visits.

10.6. Study Drug Handling and Disposal

The Investigator must retain and properly store all partially used and unused study medication until authorized by Impax regarding disposition.

11. ASSESSMENT OF EFFICACY

11.1. Parkinson's Disease Diary

Subjects are to record "asleep," "Off," and "On" without or with (nontroublesome or troublesome) dyskinesias during waking hours every 30 minutes over a 24-hour day. In the PD Diaries, subjects are instructed to indicate for each half-hour their predominant state during most of that period. "Off" is defined as the typical functional state when the medication is no longer providing benefit with regard to mobility, slowness, and stiffness in spite of taking medications. "On" is defined as the typical functional state when a subject has received medication and the medication is providing benefit with regard to mobility, slowness, and stiffness. Dyskinesias are defined as involuntary and irregular twisting and/or turning movements. Dyskinesia movements are usually an effect of medication and occur during "On" time. Nontroublesome dyskinesias do not interfere with function or do not cause meaningful discomfort. Troublesome dyskinesias do interfere with function or do cause meaningful discomfort.

11.2. Patient and Investigator Global Assessments

- Patient Global Impression of Change (Appendix F): The patient will compare his/her condition from the start of the study on a 7-point scale ranging from "Very much worse" (1) to "Very much improved" (7) at the time of the assessment.
- Clinical Global Impression of Change (Appendix G): The clinician will compare the subjects' condition from the start of the study on a 7-point scale ranging from "Very much worse" (1) to "Very much improved" (7) at the time of the assessment.
- Patient Global Impression of Severity (Appendix H): The patient will determine the severity of the disease on a 7-point scale ranging from "Normal, not at all ill" (1) to "Extremely severely ill" (7) at the time of the assessment.
- Clinical Global Impression of Severity (Appendix I): The clinician will determine the severity of the disease on a 7-point scale ranging from "Normal, not at all ill" (1) to "Among the most extremely ill of subjects" (7) at the time of the assessment.

11.3. Movement Disorders Society Version of Unified Parkinson's Disease Rating Scale

The MDS-UPDRS has 4 parts:

- Part I: Non-Motor Aspects of Experiences of Daily Living (nM-EDL) has 2 components. Component IA contains a number of behaviors assessed by the investigator with all pertinent information from the patients and caregivers. Component IB is completed by the patient with or without help from the caregiver but independent of the investigator. These sections can be reviewed by the rater to ensure all questions are answered clearly and the rater can help explain any ambiguities.

- Part II: Motor Aspects of Experiences of Daily Living (M-EDL) is a self-administered questionnaire but can be reviewed by the investigator to ensure completeness and clarity.
- Part III: Motor Examination assesses the motor signs of PD and has instructions for the rater to give to or to demonstrate to the patient. It is completed by the rater.
- Part IV: Motor Complications integrates patient-derived information with the rater's clinical observations and judgements and is completed by the rater. It contains instructions for the rater and instructions to be read to the patient.

11.4. Additional Assessments

- Parkinson's Disease Questionnaire-39 (PDQ-39) is a self-reported questionnaire. Using the 39-items, 8 domains are defined: mobility (Questions 1-10), activities of daily living (ADL) (Questions 11-16), emotional well-being (Questions 17-22), stigma (Questions 23-26), social support (Questions 27-29), cognition (Questions 30-33), communication (Questions 34-36) and bodily discomfort (Questions 37-39).
- Non-Motor Symptom assessment scale for Parkinson's Disease (NMSS) is a 30-item investigator rated questionnaire. The NMSS contains 9 domains: cardiovascular (Questions 1, 2), sleep/fatigue (Questions 3-6), mood/cognition (Questions 7-12), perceptual problems (Questions 13-15), attention/memory (Questions 16-18), gastrointestinal (Questions 19-21), urinary (Questions 22-24), sexual function (Questions 25, 26), and miscellaneous (Questions 27-30).
- Parkinson's Disease Sleep Scale-2 (PDSS-2) is 15-item self-reported questionnaire. Three domains are defined: disturbed sleep (Questions 1-3, 8, 14), motor symptoms at night (Questions 4-6, 12, 13), PD symptoms at night (Questions 7, 9-11, 15).
- Parkinson Anxiety Scale (PAS) is a 12-item patient or observer rated questionnaire with 3 domains: persistent anxiety (Questions A.1-A.5), episodic anxiety (Questions B.1-B.4) and avoidance anxiety (Questions C.1-C.3).

12. ASSESSMENT OF SAFETY

12.1. Safety Parameters

Safety will be assessed by the following parameters:

- Electrocardiograms (ECGs), clinical laboratory tests, physical examinations, the Columbia-Suicide Severity Rating Scale (C-SSRS), and vital signs, including supine and standing orthostatic blood pressure and heart rate.
- Adverse events and concomitant medications will be evaluated throughout the course of the study.

12.2. Adverse Events

12.2.1. Definition of Adverse Event

An adverse event (adverse experience) is any untoward medical occurrence in a patient or clinical trial subject administered a medicinal product and which does not necessarily have to have a causal relationship with this treatment. An adverse event can therefore be any unfavorable and unintended sign (eg, an abnormal laboratory finding), symptom, or disease temporally associated with the use of a medicinal product, whether or not considered related to the medicinal product.

All AEs and any clinically significant physical examination findings, 12-lead ECG abnormalities, or clinical laboratory measurements occurring during the study that were not present prior to administration of study medication and that continue at Study Exit should be followed and evaluated with additional tests, if necessary, until the AEs are medically stable or resolved. Follow-up on these AEs should be recorded on the source documents and reported to Impax.

12.2.2. Recording Adverse Events

Elicit information about AEs with nonselective questions such as: “Have you experienced any changes in your health status since your last visit?” Encourage subjects to report AEs at onset.

Record information for any AE that emerges from the time the subject signs the ICF until Study Exit.

Monitor each subject closely for the development of AEs and record all such events on the AE page of the CRF. Whenever possible, group signs and symptoms that constitute a single diagnosis. For example, cough, rhinitis, and sneezing might be grouped as upper respiratory infection.

For each AE, record the onset date, severity, seriousness, relationship to study medication, date of resolution (or continuing), action taken, and outcome in the CRF. The Investigator is to make a causality assessment (relationship to study medication) for every AE.

12.2.3. Follow-up

The Investigator must follow each AE until resolved or medically stable.

12.2.4. Relationship to Study Drug

The Investigator documents his/her opinion of the relationship of the AE to the study medication as follows:

- Not Related—the experience can be readily explained by the subject's underlying medical condition or concomitant medications and no relationship exists between the study medication and the experience.
- Unlikely Related—the temporal relationship between the AE and the administration of the study medication is uncertain and it is likely that the AE can be explained by the subject's medical condition or other therapies.
- Possibly Related—there is some logical temporal relationship between the AE and the administration of the study medication and the experience is unlikely to be explained by the subject's medical condition or other therapies.
- Related—the temporal relationship is compelling between the administration of the study medication and the AE cannot be explained by the subject's medical condition or other therapies.

12.2.5. Assessment of Severity

Grade each AE for severity and note in the description of the AE. Determine the severity category of mild, moderate, or severe, as defined below, and enter the information on the AE page of the CRF.

- Mild—causing no limitation of usual activities
- Moderate—causing some limitation of usual activities
- Severe—causing inability to carry out usual activities

12.3. Serious Adverse Events

12.3.1. Definition of Serious Adverse Event

A serious adverse event (SAE) is any AE occurring at any dose that results in any of the following outcomes, regardless of relationship to the study medication:

- Death
- A life-threatening adverse drug experience
- Inpatient hospitalization or prolongation of existing hospitalization
- A persistent or significant disability/incapacity
- A congenital anomaly/birth defect

- Important medical events that may not result in death, be life-threatening, or require hospitalization may be considered an SAE when, based upon appropriate medical judgment, they may jeopardize the subject and may require medical or surgical intervention to prevent one of the outcomes listed in this definition.

12.3.2. Reporting Serious Adverse Events

Any SAE that occurs from the time the subject signs an ICF until 30 days after taking the final dose of study medication must be reported by the investigative staff to the Sponsor or the Sponsor's representative within 24 hours of knowledge of the event (see Study Contact Information).

An SAE form must be completed and sent to the Sponsor and/or the Sponsor's representative. All SAEs must also be recorded on the AE page of the CRF. Additionally, all SAEs must be reported to the institutional review board (IRB) per the IRB's requirements.

Those SAEs that are considered both serious and unexpected and related to the study drug are subject to expedited reporting. An "unexpected AE" is any AE where the nature or severity is not consistent with the current investigator brochure (IB) or if an IB is not required or available, the specificity or severity is not consistent with the provided risk information.

Unexpected fatal or life-threatening SAEs related to the study drug must be reported by the Sponsor to the appropriate regulatory authority in an expedited manner (ie, first report within 7 days of first knowledge by the Sponsor). The Sponsor will provide a final written report to that authority within 15 days of initial receipt of information on the event. The Sponsor or the Sponsor's representative will also inform all participating Investigators of the SAE.

Unexpected SAEs that are not fatal or life-threatening must be reported by the Sponsor to the appropriate regulatory authority as soon as possible but no later than 15 calendar days after first knowledge of the SAE by the Sponsor. The Sponsor or the Sponsor's representative also informs all participating Investigators of the SAE.

Subjects withdrawn from the study due to any SAE will be followed until the SAE is resolved or medically stable. Record all SAEs, regardless of severity and whether or not related to the study medication, on the appropriate page of the CRF.

The Investigator must determine whether the seriousness of the event warrants removal of the subject from the study. He/she should, in any case, institute appropriate diagnostic and therapeutic measures and keep the subject under observation for as long as is medically indicated, or refer the subject to appropriate health professionals.

12.4. Pregnancy

Any pregnancy that occurs from the time the subject signs an ICF until 30 days after taking the final dose of study medication must be reported within 24 hours to the Sponsor or the Sponsor's representative and the subject should be terminated from the study. All pregnancies will be followed through to delivery of the infant. If the subject experiences a termination of the pregnancy, it should be reported as defined in Section 12.3.2.

12.5. Other Safety Parameters and Related Information

Additional safety parameters (laboratory tests, 12-lead ECGs, physical examinations, and vital signs), the C-SSRS, the GCSI, and concomitant medications are collected as shown in the Schedule of Assessments in Table 4. Clinical laboratory assessments are listed in Appendix R.

13. STATISTICS

13.1. Study Design and Sample Size Estimation

This is a multicenter, randomized, double-blind, double-dummy, active-controlled, parallel-group study. Assuming a difference of 1 hour between IPX203 and IR CD-LD in "Good on" time and a standard deviation of the treatment differences to be 3.0 hours, a sample size of 210 subjects per arm will be needed to ensure at least 90% power at a 0.05 significance level.

Assuming approximately an 18% prerandomization drop-out, approximately 510 subjects would need to be enrolled to randomize 420 subjects.

13.2. Demographics/Baseline Comparability

The demographics and baseline characteristics will be summarized by treatment arms and overall using descriptive statistics. Demographics information includes age, sex, and race. Baseline disease characteristics include MDS-UPDRS Parts I, II, III, and IV, Hoehn and Yahr stage, MoCA scores, and age of onset of PD. Distributions of dosing information, including LD doses and years on LD, will also be summarized.

13.3. Efficacy Endpoints

- Primary endpoint: Change from baseline in "Good on" time in hours per day, averaged over the PD Diary days, at the end of double-blind treatment period (Visit 7 or early termination). "Good on" time is derived from the 3-day PD Diaries and is defined as the sum of "On" time without dyskinesia and "On" time with nontroublesome dyskinesia.
- Key secondary endpoints:
  - Change from baseline in "Off" time in hours per day, averaged over the PD Diary days at the end of double-blind treatment period (Visit 7 or early termination)
  - Proportion of subjects with either "much improved" or "very much improved" in Patient Global Impression of Change (PGI-C) scores at the end of double-blind treatment period (Visit 7 or early termination)
  - Change from baseline in the MDS-UPDRS Part III at the end of double-blind treatment period (Visit 7 or early termination)
  - Change from baseline in the sum of MDS-UPDRS Parts II and III at the end of double-blind treatment period (Visit 7 or early termination)
- Additional endpoints:

  The following endpoints will be evaluated (at the post-randomization visits) as change from baseline (Visit 4) as well as change from the study entry (Visit 1), when applicable, by visits:
  - Percent "Off" time during waking hours derived from the 3-day PD Diaries
  - Average duration of each continuous "Good on" and each continuous "On"

- Hours of (1) “Off” time (from Visit 1), (2) “Good on” time (from Visit 1), (3) “On” time with dyskinesia, (4) “On” time with troublesome dyskinesia, and (5) “On” time with nontroublesome dyskinesia, and (6) asleep time derived from the 3-day PD Diaries
- Proportion of subjects with an improvement in “Good on” time of at least 1, 1.5, 2, 2.5, and 3 hours
- Proportion of subjects with a reduction in “Off” time of at least 0.5, 1, 1.5, 2, 2.5, and 3 hours
- Proportions of subjects who are “On” upon awakening and “Good on” upon awakening
- Average time to “On” upon awakening
- Change from baseline in the average number of motor fluctuations per day averaged over the PD Diary days. A motor fluctuation is defined as a change from “Off” to “On” state or from “On” to “Off” state.
- MDS-UPDRS total score (sum of Parts I, II, III, and IV) and Parts I, II, and IV separately
- MDS-UPDRS Part III and Parts II + III combined (from Visit 1)
- MDS-UPDRS Part II Question 2.9
- PDQ-39 total score and individual domain scores
- NMSS total score and individual domains
- PDSS-2 total score and individual domains
- PDSS-2 items 9, 10, 11, 12, and 13 combined
- PAS total score and individual domains
- PGI-S
- Proportion of subjects with either “severely ill” or “extremely severely ill” on the PGI-S
- CGI-S
- Proportion of subjects with either “severely ill” or “among the most extremely ill of subjects” on the CGI-S
- PGI-C scores
- CGI-C scores
- Proportion of subjects with either “much improved” or “very much improved” on the CGI-C

13.4. Analysis of Efficacy Data

In order to control the type I error rate, the primary efficacy endpoint and key secondary efficacy endpoints will be tested in a single hierarchical order as detailed in Section 13.8.

13.4.1. Primary Efficacy Endpoint

The primary efficacy endpoint is the mean change from baseline in "Good on" time in hours per day, averaged over the PD Diary days, at the end of the double-blind therapy (Visit 7 or early termination). "Good on" time is derived from the 3-day PD Diaries. For each day, "Good on" time is calculated by adding the number of half-hour intervals in which either an "On without dyskinesia" or "On with nontroublesome dyskinesia" is checked.

The primary efficacy endpoint will be analyzed using a mixed model for repeated measures (MMRM). The model will include baseline (Visit 4) "Good on" time as a covariate, treatment, visit, and pooled center as fixed effects, and a treatment-by-visit interaction. The model will employ an unstructured within subject covariance matrix and a restricted maximum likelihood (ReML) estimation method. The degree-of-freedom of the denominator will be estimated using the Kenward-Roger method (Kenward and Roger 1997). The primary analysis population will be the modified intent-to-treat as defined in Section 13.9. Missing data will be handled as in Section 13.10.

If the model fails to converge due to the unstructured covariance matrix, a simpler covariance matrix will be employed in the order of 1) heterogeneous Toeplitz [SAS PROC MIXED type = TOEPH], 2) heterogeneous autoregressive of order 1 [type = ARH(1)], 3) heterogeneous compound symmetry [type = CSH], 4) Toeplitz [type = TOEP], 5) autoregressive of order 1 [type = AR(1)], 6) compound symmetry [type = CS]. The first covariance structure that does not have a convergence problem will be the one used for the primary analysis.

13.4.2. Key Secondary Efficacy Endpoints

The first key secondary endpoint is the mean change from baseline in "Off" time in hours per day, averaged over the PD Diary days, at the end of the double-blind therapy (Visit 7 or early termination). "Off" time is derived from the 3-day PD Diaries. For each day, "Off" time is calculated by adding the number of half-hour intervals in which an "Off" is checked. This endpoint will be analyzed using a MMRM model with baseline (Visit 4) "Off" time as a covariate, treatment, visit, and pooled center as fixed effects, and a treatment-by-visit interaction. The model will employ an unstructured within subject covariance matrix and a restricted maximum likelihood (ReML) estimation method. The degree-of-freedom of the denominator will be estimated using the Kenward-Roger method (Kenward and Roger 1997).

The proportion of subjects with either "much improved" or "very much improved" in PGI-C scores at the end of the double-blind therapy (Visit 7 or early termination), the second key secondary endpoint, will be analyzed using a Cochran-Mantel-Haenszel test to compare the two treatment groups with pooled center as a stratification factor.

The mean change from baseline in the MDS-UPDRS Part III at the end of the double-blind therapy (Visit 7 or early termination) is the third key secondary endpoint. This endpoint will be analyzed using an MMRM model with baseline (Visit 4) MDS-UPDRS Part III as a covariate,

treatment, visit, and pooled center as fixed effects, and a treatment-by-visit interaction. The model will employ an unstructured within subject covariance matrix and a restricted maximum likelihood (ReML) estimation method. The degree-of-freedom of the denominator will be estimated using the Kenward-Roger method (Kenward and Roger 1997).

The mean change from baseline in sum of the MDS-UPDRS Parts II and III at the end of the double-blind therapy (Visit 7 or early termination) is the fourth key secondary endpoint. This endpoint will be analyzed using an MMRM model with baseline (Visit 4) MDS-UPDRS Parts II and III combined as covariates, treatment, visit, and pooled center as fixed effects, and a treatment-by-visit interaction. The model will employ an unstructured within subject covariance matrix and a restricted maximum likelihood (ReML) estimation method. The degree-of-freedom of the denominator will be estimated using the Kenward-Roger method (Kenward and Roger 1997).

For the 4 key secondary endpoints, the analysis population will be the modified intent-to-treat as defined in Section 13.9. Missing data will be handled as in Section 13.10.

13.4.3. Additional Efficacy Endpoints

In general, continuous endpoints will be summarized by standard descriptive statistics (mean, standard deviation, median, minimum, and maximum). Categorical endpoints will be summarized by frequencies and percentages. Comparisons between the two arms will be explored using appropriate statistical methodologies. Details will be provided in the SAP.

The primary endpoint, key secondary endpoints, as well as other efficacy endpoints will be presented by visit over the whole blinded treatment period from Baseline (Visit 4) to the end of the double-blind treatment period (Visit 7).

Other additional efficacy endpoints collected postrandomization will be analyzed by visit in a fashion similar to the primary and key secondary endpoints.

Additionally the PGI-C and CGI-C will be analyzed using analysis of variance (ANOVA) with treatment and pooled center as factors.

13.5. Center Pooling Algorithm

The center pooling algorithm is as follows.

1. Sort centers from each country from smallest to largest based on the number of subjects in the modified intent-to-treat analysis set (mITT).
2. Centers with less than 5 mITT subjects or at least one mITT subject per treatment group will be pooled with the next smallest center in the same country until the combined center (namely, pseudo-center) has more than 5 mITT subjects and at least one mITT subject per treatment group.
3. If after pooling within the same country, the pseudo-center still has less than 5 mITT subjects or still has zero subjects in one of the treatment group, that pseudo-center will be pooled with the next smallest center in the same geographical region (Western Europe, Eastern Europe, North America).

4. If after pooling within the same geographical region, the pseudo-center still has less than 5 mITT subjects or still has zero subjects in one of the treatment group, that pseudo-center will be pooled with the next smallest center in any region.

The process continues until all pooled pseudo-centers have at least 5 mITT subjects and at least one mITT subject per treatment group. These pooled centers will be used in analyses that adjust for pooled centers.

This pooling algorithm will be detailed in the Statistical Analysis Plan (SAP).

13.6. Sensitivity Analyses of the Primary Endpoint and Key Secondary Endpoints

Sensitivity analyses will be performed with respect to the primary efficacy endpoint and continuous key secondary endpoints ("Off" time, MDS-UPDRS Part III, and MDS-UPDRS Parts II and III combined) as follows.

13.6.1. Assessing Assumptions of the Mixed Model for Repeated Measures (MMRM)

a. The normality and homoscedasticity assumptions will be examined through residual analyses. The normality and homoscedasticity assumptions will further be tested via Shapiro-Wilk (Shapiro and Wilk 1965) and Levene (Levene 1960) tests, respectively, at a 0.05 level of significance. If normality and/or homoscedasticity assumption appears violated, then:

   i. Nonparametric Wilcoxon Rank Sum test will be performed to compare the two treatment groups, with missing data imputed by the last observation carried forward (LOCF) method.

   ii. Multiple imputation rank based analysis: instead of missing data imputed by the LOCF method, in this analysis, missing data at Visit 7 will be imputed multiple times to create 50 complete datasets. The multiple imputation procedure is described in Section 13.6.4 (part of the pattern-mixture model), using $f = 0\%$. The Wilcoxon Rank Sum test will be performed on each of the 50 datasets. The results are then combined using Rubin's rule (Rubin 1987) via SAS PROC MIANALYZE.

b. Missing at Random (MAR) assumption will be evaluated as discussed in Section 13.6.4.

13.6.2. Complete Case Analysis

The primary endpoint will be analyzed using an ANCOVA model with "Good on" time at baseline (Visit 4) as a covariate, pooled center and treatment as factors. The model will be performed on subjects with <u>both</u> baseline "Good on" time and Visit 7 "Good on" time.

13.6.3. Single LOCF/BLOCF Imputation

The primary efficacy endpoint will be analyzed using an ANCOVA model with "Good on" time at Visit 4 as a covariate, pooled center and treatment as factors. Missing data will be imputed by the LOCF and baseline observation carried forward (BLOCF) methods. These analyses will be performed on the mITT population.

13.6.4. Pattern-Mixture Model

If an overall dropout rate postrandomization is > 15%, pattern-mixture models (PMM) will be employed to assess the robustness of the results under the missing not at random (MNAR) assumption. The pattern for PMM is defined by patients' last visit with an observed primary efficacy endpoint and the reason for dropout.

Multiple imputation with mixed missing data mechanism (MNAR for a missing data pattern and MAR for others) will be used to investigate the robustness of the primary result. Four specific data patterns will be examined:

1. Dropout at Visit 5 and reason = Lack of efficacy in IPX203 treatment arm,
2. Dropout at Visit 5 and reason = Lack of efficacy or adverse events in IPX203 treatment arm,
3. Dropout at Visit 6 and reason = Lack of efficacy in IPX203 treatment arm,
4. Dropout at Visit 6 and reason = Lack of efficacy or adverse events in IPX203 treatment arm.

The missing values will be imputed 50 times (multiple imputation) under the assumption that the distribution of the missing values is the same as that of the observed values. The PMM then investigates the departure from the MAR assumption by progressively decreasing the outcome (the "penalty") for those on IPX203 arm who fall into an assumed MNAR pattern above. For the dropout subjects on IPX203 arm that fall into one of the patterns above, the "penalty" is obtained by subtracting the imputed missing data after dropout by a factor *f*, with *f* starts from 0%, 5%, 10%, 15%, 20%, 25%, 30%, …, 100% of the treatment difference seen in the primary model. This process continues until the conclusion from the primary analysis is overturned (a tipping point). In other words, if the dropout subject is from IPX203 arm and the dropout pattern falls into one of the 4 patterns above, then the subject's imputed value will be adjusted downward by a factor *f*, where *f* goes from 0% to 100% of the treatment difference seen in the primary model. Note that if 0% is used, the analysis is essentially multiple imputation under MAR assumption. On the other hand, if 100% is used, then the analysis is essentially a "jump to reference" where outcome on IPX203 arm is assumed to be the same as outcome on IR CD-LD. After imputations, the dataset will be analyzed using an MMRM model similar to the primary analysis model. The results will then be combined using the Rubin's rule (Rubin 1987) via SAS PROC MIANALYZE.

The procedure will be carried out in SAS as follows:

a. Use Monte Carlo Markov Chain (MCMC) method in SAS PROC MI by treatment group to impute the intermittent missing data to form monotone missingness.
b. Use MAR-based multiple imputation in SAS PROC MI to impute the missing data (SAS MONOTONE statement).
c. For dropout subjects in IPX203 arm who fall into an MNAR pattern specified above, a delta which equals to *f* times the treatment difference obtained from the primary MMRM analysis at Visit 7 will be subtracted from their imputed values for all visits after the dropout ("penalizing" IPX203 arm).

d. After imputation, use the MMRM model as in the primary analysis model to analyze the complete data along with the imputed data.

e. Repeat steps a through d 50 times.

Combine results using Rubin's rule (Rubin 1987) via SAS PROC MIANALYZE.

13.7. Subgroup Analyses

The primary, key secondary endpoints, as well as overall summary of adverse events, will be examined for the following subgroups.

- Age: < 65, ≥ 65 years old at study entry
- Sex: Males, Females
- Race: Caucasians, non-Caucasians

Additionally, the following subgroups may be examined:

- Region
- Ethnicity
- Concomitant medications
- Weight
- Body mass index (BMI)
- PD duration
- Age of PD onset
- "Good On" time and "Off" time at study entry.

For all subgroup efficacy analyses, the same analysis methods as the primary and key secondary endpoints will be applied, unless the sample size in one of the subgroups becomes too small to hinder the statistical analysis. In that case, no inferential statistics will be provided for such a subgroup. The details for final subgroup analyses will be documented in the SAP.

13.8. Multiplicity Adjustments

The primary endpoint and 4 key secondary endpoints will be tested in a sequential hierarchical order as follows.

1. The primary endpoint, the mean change from baseline in "Good on" time (hours per day), will be tested first at a 0.05 level of significance.
2. If statistical significance is demonstrated, then the first key secondary endpoint, the mean change from baseline in "Off" time (hours per day), will be tested next at a 0.05 level of significance.
3. If statistical significance is demonstrated, then the second key secondary endpoint, the proportion of subjects with either "much improved" or "very much improved" on the PGI-C, will be tested next at a 0.05 level of significance.

4. If statistical significance is demonstrated, then the third key secondary endpoint, the mean change from baseline in the MDS-UPDRS Part III, will be tested at a 0.05 level of significance.
5. If statistical significance is demonstrated, then the fourth key secondary endpoint, the mean change from baseline in the sum of the MDS-UPDRS Parts II and III combined will be tested next at a 0.05 level of significance.

For the other efficacy endpoints, no adjustment will be made.

13.9. Analysis Populations

13.9.1. Safety Analysis Set

The Safety Analysis set will include all subjects who were treated with any study drug.

13.9.2. Intent-to-Treat Analysis Set

The Intent-to-treat Analysis Set will include all subjects who were randomized and treated with any study drug and have a baseline and at least one postbaseline efficacy assessment.

13.9.3. Modified Intent-to-Treat Analysis Set

The Modified Intent-to-treat Analysis Set will include all subjects who were randomized and treated and have a valid baseline PD Diary and at least one valid postrandomization PD Diary. This analysis set will be used for the primary analysis and key secondary analyses.

13.9.4. Completers Analysis Set

The Completers Analysis Set will include all subjects who were randomized and treated and complete the study.

13.10. Handling of Missing Data

13.10.1. Missing Data for PD Diaries

An MMRM approach will be used to handle missing visit data. MMRM analysis will use all available valid visit data, including subjects with some missing visit data, in order to arrive at an estimate of the mean treatment effect.

A PD Diary is valid if at least 1 day of diary data is available using the rules defined below.

Imputation of missing data for a PD Diary day will be required if a PD Diary is not completed for a full day (6 am to 5:30 am). In this case, the method of imputation will be dependent upon the amount and pattern of missing data.

- For subjects with more than 1 day of diary data, the following rules will apply:
  1. If more than 4 half-hour time intervals are missing, then that particular day will not be included in the analysis. The missing data will be handled in the MMRM model.
  2. If a one-half hour time interval is missing and the observations on either side of the time interval are not missing, then the missing time interval will be imputed by

assigning a value of the previous measurement for the first 15 minutes and the value of the next measurement for the second 15 minutes.

3. If 2, 3, or 4 consecutive half-hour time intervals are missing, and these time intervals are available from other days of the visit then the following rules will be applied:
   a. For missing time intervals on Day 1, data from Day 2 will be used for imputation for the same time intervals. If Day 2 data is also incomplete or not available, then Day 3 data will be used.
   b. For missing time intervals on Day 2, data from Day 3 will be used for imputation if available; otherwise Day 1 data will be used.
   c. Data from Day 2 will be used for imputing missing time intervals on Day 3. If data from Day 2 is not available, then Day 1 data will be used for imputation.
   d. If data at the same time period are missing across all days, then the approach will be to split the individual missing half-hour intervals into 2 periods, with the first-half interval being imputed with data from the immediate previous nonmissing time period and the second-half interval being imputed with the next nonmissing time interval.

- For subjects with only 1 day of diary data, the following rules will apply:
  1. If more than 4 half-hour time intervals are missing, then that particular day will not be included in the analysis. The missing data will be handled in the MMRM model.
  2. If a one-half hour time interval is missing and the observations on either side of the time interval are not missing, then the missing time interval will be imputed by assigning a value of the previous measurement for the first 15 minutes and the value of the next measurement for the second 15 minutes.
  3. If 2, 3, or 4 consecutive half-hour intervals are missing, then the approach will be to split the individual missing half-hour intervals into 2 periods, with the first-half interval being imputed with data from the immediate previous nonmissing time period and the second-half interval being imputed with the next nonmissing time interval.

13.10.2. Missing Data for Global Assessments (PGI-C, CGI-C, PGI-S and CGI-S)

For subjects with missing PGI-C or CGI-C for a particular visit, the data will be imputed as nonresponders (ie, not being “much improved” or “very much improved”).

For subjects with missing PGI-S or CGI-S for a particular visit, the data will be imputed as nonresponders (ie, being “severely ill” or “extremely severely ill” for PGI-S and being “severely ill” or “among the most extremely ill of subjects” for CGI-S).

13.10.3. Missing Data for MDS-UPDRS

If the MDS-UPDRS are missing for the particular visit, the missing data will be handled via the MMRM model.

If component questions are missing for a particular part of the MDS-UPDRS questionnaire, the missing items are assigned the average value for other items in that part as follows (Goetz 2015):

- For Part I (13 questions): up to 1 missing question will be imputed using the average value of the remaining 12 questions.
- For Part II (13 questions): up to 2 missing questions will be imputed using the average value of the remaining 11 questions.
- For Part III (33 questions): up to 7 missing questions will be imputed using the average value of the remaining 26 questions.
- Part IV (6 questions): no imputation is done.

If more component questions are missing than above for a particular part of the MDS-UPDRS questionnaire, the entire questionnaire will not be included in the analysis for that particular assessment. Missing data will be handled in a fashion similar to PD Diary data (Section 13.10.1) using the MMRM model.

For quality-of-life endpoints, missing responses within a questionnaire will not be imputed.

13.11. Analysis of Safety

The safety analysis will include all subjects who receive at least 1 dose of study medication. Reported AEs will be coded using the Medical Dictionary for Regulatory Activities (MedDRA). All AEs will be summarized by system organ class and preferred terms within system organ class. The severity, seriousness, and relationship to study medication will also be summarized by treatment arms. Each AE (based on preferred term) is counted once for a given subject. If the same AE occurred on multiple occasions, the highest severity and least complimentary relationship will be assumed.

The incidence of treatment-emergent AEs and serious AEs will be summarized by treatment arms.

Additionally, laboratory test data, physical examinations, vital signs, ECGs, C-SSRS, and GCSI will be summarized by treatment arms.

14. ADMINISTRATIVE PROCEDURES

14.1. Guidelines for Good Clinical Practice

This study will be conducted in accordance with principles of Good Clinical Practice (GCP) as promulgated by the ICH. Good Clinical Practice is an international ethical and scientific quality standard for designing, conducting, recording, and reporting trials that involve the participation of human subjects. Compliance with this standard provides public assurance that the rights, safety, and well-being of human subjects are protected under current ethical principles, and that the clinical trial data are credible. Current GCP standards may be found in ICH Guidance E6 (Good Clinical Practice: Consolidated Guidance). This guidance describes the principles of GCP and the obligations of the institutional review board (IRB), the Investigator and the Sponsor in conducting this study in accordance with those principles.

14.2. Institutional Review Board Approval

The review of this protocol by an IRB and the performance of all aspects of the study, including the methods used for obtaining informed consent, must be in accordance with principles enunciated in the ICH and GCP Guidelines and by the appropriate regulatory authorities.

The Investigator is responsible for preparing documents for submission to the relevant IRB and obtaining written approval for this study. Institutional Review Board approval must be obtained prior to the initiation of the study. The Investigator’s continued participation in the study is contingent on renewing approval with the IRB at least annually.

14.3. Informed Consent

Site personnel should prepare an Informed Consent Form (ICF) incorporating the necessary elements of consent. The ICF is to be approved by Impax prior to submission to the IRB. The Investigator or his/her staff must explain the nature of the investigation and the risks involved to each subject prior to screening, and obtain a signed ICF. The subject should also be informed that he/she is free to voluntarily withdraw from the study at any time.

14.4. Study Monitoring

Impax representatives or designees will conduct site visits to the investigational facilities for the purpose of monitoring the study. The Investigator agrees to allow the monitor to inspect the drug storage area, study drug stocks, drug accountability records, subject charts and study source documents, and other records relevant to study conduct. The Investigator must permit access to such records if a regulatory or compliance audit is required.

14.5. Protocol Amendments

All amendments to the protocol must be documented in writing, reviewed and approved by the Sponsor and Investigator, and submitted to the IRB for approval prior to implementation. If the protocol amendment substantially alters the study design or potential risk to the subject, a new

written ICF for continued participation in the study must be obtained from each subject affected by the change.

14.6. Termination of Study

The Sponsor has the right to terminate this study and remove all study material from the site at any time for medical or administrative reasons. In this event, the Sponsor will endeavor to give adequate notice to allow safe withdrawal of subjects from the study.

14.7. Case Report Forms

Site personnel should collect and record data for the study as source documents, and transfer the data into the CRF.

The Investigator must ensure that complete data for the clinical study are collected and accurately documented in the appropriate sections of the CRF and adequately supported by the appropriate source documentation. In addition, it is the Investigator's responsibility to provide signatures where requested indicating concurrence with data in the CRF.

14.8. Investigator's Final Conduct Report

At the completion of the study, the Investigator must provide Impax a copy of the final conduct report that was submitted to their IRB, including a review of AEs.

14.9. Records Retention

International Conference on Harmonization, GCP, and US FDA guidelines require that essential documents be retained until at least 2 years after the last approval of a marketing application and until there are no pending or contemplated marketing applications, or at least 2 years have elapsed since the formal discontinuation of clinical development of the investigational product.

However, the essential documents should be retained for a longer period if required by the applicable regulatory requirements or by an agreement with the Sponsor. Records should never be destroyed without written approval from the Sponsor.

If an Investigator leaves the institution, he/she must transfer responsibilities for record retention to another individual willing to accept them. The Investigator must notify the Sponsor in writing of the transfer of study documents before the transfer of the study documents.

15. PUBLICATION POLICY

Study results may not be published without prior written approval from Impax.

16. LIST OF REFERENCES

Blanchet PJ, Calon F, Martel JC, et al. Continuous administration decreases and pulsatile administration increases behavioral sensitivity to a novel dopamine D2 agonist (U-91356A) in MPTP-exposed monkeys. J Pharmacol Exp Ther. 1995;272:854-859.

Engber TM, Susel Z, Juncos JL, Chase TN. Continuous and intermittent levodopa differentially affect rotation induced by D-1 and D-2 dopamine agonists. European Journal of Pharmacology. 1989;168:291-298.

Fahn S. Parkinson disease, the effect of levodopa, and the ELLDOPA trial. Earlier vs Later L DOPA. Arch Neurol. 1999 May;56(5):529-35.

Food and Drug Administration 2003. Guidance for Industry. Bioavailability and Bioequivalence Studies for Orally Administered Drug Products — General Considerations. U.S. Department of Health and Human Services. Food and Drug Administration. Center for Drug Evaluation and Research (CDER). March 2003.

Goetz CG, Luo S, Wang L, Tilley BC, LaPelle NR, Stebbins GT. Handling missing values in the MDS-UPDRS. Mov Disord. 2015 Oct;30(12):1632-8.

Juncos JL, Engber TM, Raisman R, et al. Continuous and intermittent levodopa differentially affect basal ganglia function. Ann Neurol. 1989;25:473-478.

Kenward MG and Roger JH. Small sample inference for fixed effects from restricted maximum likelihood. Biometrics. 1997;53:983-997.

Kurlan R, Rothfield KP, Woodward WR, Nutt JG, Miller C, Lichter D, et al. Erratic gastric emptying of levodopa may cause “random” fluctuations of parkinsonian mobility. Neurology. 1988;38(3):419-21.

Levene H. In contributions to probability and statistics: Essays in honor of Harold Hotelling, I. Olkin et al. eds. Stanford University Press. 1960: 278-292.

Melamed E, Bitton V, Zelig O. Delayed onset of responses to single doses of L-dopa in parkinsonian fluctuators on long-term L-dopa therapy. Clin Neuropharmacol. 1986;9(2):182-8.

Mizuno Y. Where do we stand in the treatment of Parkinson's disease? Neurology. 2007;254:13-18.

Nilsson D, Nyholm D, Aquilonius S-M. Duodenal levodopa infusion in Parkinson's disease--long-term experience. Acta Neurologica Scandinavica. 2001;104:343-348.

Nyholm D, Nilsson Remahl AI, Dizdar N, et al. Duodenal levodopa infusion monotherapy vs oral polypharmacy in advanced Parkinson disease. Neurology. 2005;64:216-223.

Rubin DB. Multiple Imputation in Nonresponse in Surveys. San Francisco, CA: Wiley; 1987.

Shapiro SS and Wilk MB. An analysis of variance test for normality (complete samples). Biometrika. 1965;52(3-4): 591-611.

Sinemet (Carbidopa-Levodopa) Tablets Prescribing Information. Whitehouse Station, NJ: Manufactured by Merck & Co., Inc.; Marketed by Bristol-Myers Squibb Company; 2013.

Stocchi F, Quinn NP, Barbato L, Patsalos PN, O'Connel MT, Ruggieri S, et al. Comparison between a fast and a slow release preparation of levodopa and a combination of the two: a clinical and pharmacokinetic study. Clinical Neuropharmacol. 1994;17(1):38-44.

Stocchi F, Vacca L, et al. Intermittent vs continuous levodopa administration in patients with advanced Parkinson disease: a clinical and pharmacokinetic study. Arch Neurol. 2005:62(6):905-910.

17. APPENDICES

APPENDIX A. PRESCRIBING INFORMATION FOR IR CD-LD

SINEMET®

(carbidopa levodopa)

Tablets

DESCRIPTION

SINEMET® (carbidopa levodopa) is a combination of carbidopa and levodopa for the treatment of Parkinson's disease and syndrome.

Carbidopa, an inhibitor of aromatic amino acid decarboxylation, is a white, crystalline compound, slightly soluble in water, with a molecular weight of 244.3. It is designated chemically as (—)-L-α-hydrazino-α-methyl-β-(3,4-dihydroxybenzene) propanoic acid monohydrate. Its empirical formula is $C_{10}H_{14}N_2O_4 \cdot H_2O$, and its structural formula is:

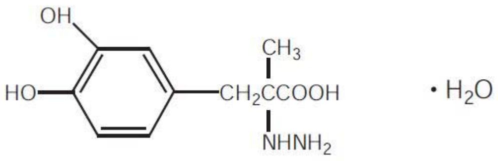

Tablet content is expressed in terms of anhydrous carbidopa which has a molecular weight of 226.3.

Levodopa, an aromatic amino acid, is a white, crystalline compound, slightly soluble in water, with a molecular weight of 197.2. It is designated chemically as (—)-L-α-amino-β-(3,4-dihydroxybenzene) propanoic acid. Its empirical formula is $C_9H_{11}NO_4$, and its structural formula is:

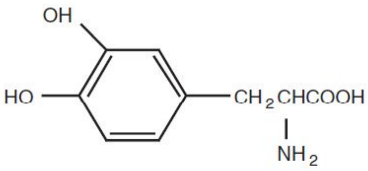

SINEMET is supplied as tablets in three strengths:

SINEMET 25-100, containing 25 mg of carbidopa and 100 mg of levodopa.

SINEMET 10-100, containing 10 mg of carbidopa and 100 mg of levodopa.

SINEMET 25-250, containing 25 mg of carbidopa and 250 mg of levodopa.

Inactive ingredients are hydroxypropyl cellulose, pregelatinized starch, crospovidone, microcrystalline cellulose, and magnesium stearate. SINEMET 10-100 and 25-250 Tablets also contain FD&C Blue #2/Indigo Carmine AL. SINEMET 25-100 Tablets also contain D&C Yellow #10 Lake.

CLINICAL PHARMACOLOGY

Mechanism of Action

Parkinson's disease is a progressive, neurodegenerative disorder of the extrapyramidal nervous system affecting the mobility and control of the skeletal muscular system. Its characteristic features include resting tremor, rigidity, and bradykinetic movements. Symptomatic treatments, such as levodopa therapies, may permit the patient better mobility.

Current evidence indicates that symptoms of Parkinson's disease are related to depletion of dopamine in the corpus striatum. Administration of dopamine is ineffective in the treatment of Parkinson's disease apparently because it does not cross the blood-brain barrier. However, levodopa, the metabolic precursor of dopamine, does cross the blood-brain barrier, and presumably is converted to dopamine in the brain. This is thought to be the mechanism whereby levodopa relieves symptoms of Parkinson's disease.

Pharmacodynamics

When levodopa is administered orally, it is rapidly decarboxylated to dopamine in extracerebral tissues so that only a small portion of a given dose is transported unchanged to the central nervous system. For this reason, large doses of levodopa are required for adequate therapeutic effect, and these may often be accompanied by nausea and other adverse reactions, some of which are attributable to dopamine formed in extracerebral tissues.

Since levodopa competes with certain amino acids for transport across the gut wall, the absorption of levodopa may be impaired in some patients on a high protein diet.

Carbidopa inhibits decarboxylation of peripheral levodopa. It does not cross the blood-brain barrier and does not affect the metabolism of levodopa within the central nervous system.

The incidence of levodopa-induced nausea and vomiting is less with SINEMET than with levodopa. In many patients, this reduction in nausea and vomiting will permit more rapid dosage titration.

Since its decarboxylase inhibiting activity is limited to extracerebral tissues, administration of carbidopa with levodopa makes more levodopa available for transport to the brain.

Pharmacokinetics

Carbidopa reduces the amount of levodopa required to produce a given response by about 75% and, when administered with levodopa, increases both plasma levels and the plasma half-life of levodopa, and decreases plasma and urinary dopamine and homovanillic acid.

The plasma half-life of levodopa is about 50 minutes, without carbidopa. When carbidopa and levodopa are administered together, the half-life of levodopa is increased to about 1.5 hours. At steady state, the bioavailability of carbidopa from SINEMET tablets is approximately 99% relative to the concomitant administration of carbidopa and levodopa.

In clinical pharmacologic studies, simultaneous administration of carbidopa and levodopa produced greater urinary excretion of levodopa in proportion to the excretion of dopamine than administration of the two drugs at separate times.

Pyridoxine hydrochloride (vitamin B_6), in oral doses of 10 mg to 25 mg, may reverse the effects of levodopa by increasing the rate of aromatic amino acid decarboxylation. Carbidopa inhibits this action of pyridoxine; therefore, SINEMET can be given to patients receiving supplemental pyridoxine (vitamin B_6).

Special Populations

Geriatric: A study in eight young healthy subjects (21-22 yr) and eight elderly healthy subjects (69-76 yr) showed that the absolute bioavailability of levodopa was similar between young and elderly subjects following oral administration of levodopa and carbidopa. However, the systemic exposure (AUC) of levodopa was increased by 55% in elderly subjects compared to young subjects. Based on another study in forty patients with Parkinson's disease, there was a correlation between age of patients and the increase of AUC of levodopa following administration of levodopa and an inhibitor of peripheral dopa decarboxylase. AUC of levodopa was increased by 28% in elderly patients (≥ 65 yr) compared to young patients (< 65 yr). Additionally, mean value of Cmax for levodopa was increased by 24% in elderly patients (≥ 65 yr) compared to young patients (< 65 yr) (see PRECAUTIONS, *Geriatric Use*).

The AUC of carbidopa was increased in elderly subjects (n=10, 65-76 yr) by 29% compared to young subjects (n=24, 23-64 yr) following IV administration of 50 mg levodopa with carbidopa (50 mg). This increase is not considered a clinically significant impact.

INDICATIONS AND USAGE

SINEMET is indicated in the treatment of Parkinson's disease, post-encephalitic parkinsonism, and symptomatic parkinsonism that may follow carbon monoxide intoxication or manganese intoxication.

Carbidopa allows patients treated for Parkinson's disease to use much lower doses of levodopa. Some patients who responded poorly to levodopa have improved on SINEMET. This is most likely due to decreased peripheral decarboxylation of levodopa caused by administration of carbidopa rather than by a primary effect of carbidopa on the nervous system. Carbidopa has not been shown to enhance the intrinsic efficacy of levodopa.

Carbidopa may also reduce nausea and vomiting and permit more rapid titration of levodopa.

CONTRAINDICATIONS

Nonselective monoamine oxidase (MAO) inhibitors are contraindicated for use with SINEMET. These inhibitors must be discontinued at least two weeks prior to initiating therapy with SINEMET. SINEMET may be administered concomitantly with the manufacturer's recommended dose of an MAO inhibitor with selectivity for MAO type B (e.g., selegiline HCl) (see PRECAUTIONS, *Drug Interactions*).

SINEMET is contraindicated in patients with known hypersensitivity to any component of this drug, and in patients with narrow-angle glaucoma.

WARNINGS

When SINEMET is to be given to patients who are being treated with levodopa, levodopa must be discontinued at least twelve hours before therapy with SINEMET is started. In order to reduce adverse reactions, it is necessary to individualize therapy. See DOSAGE AND ADMINISTRATION section before initiating therapy.

The addition of carbidopa with levodopa in the form of SINEMET reduces the peripheral effects (nausea, vomiting) due to decarboxylation of levodopa; however, carbidopa does not decrease the adverse reactions due to the central effects of levodopa. Because carbidopa permits more levodopa to reach the brain and more dopamine to be formed, certain adverse central nervous system (CNS) effects, e.g., dyskinesias (involuntary movements), may occur at lower dosages and sooner with SINEMET than with levodopa alone.

All patients should be observed carefully for the development of depression with concomitant suicidal tendencies.

SINEMET should be administered cautiously to patients with severe cardiovascular or pulmonary disease, bronchial asthma, renal, hepatic or endocrine disease.

As with levodopa, care should be exercised in administering SINEMET to patients with a history of myocardial infarction who have residual atrial, nodal, or ventricular arrhythmias. In such patients, cardiac function should be monitored with particular care during the period of initial dosage adjustment, in a facility with provisions for intensive cardiac care.

As with levodopa, treatment with SINEMET may increase the possibility of upper gastrointestinal hemorrhage in patients with a history of peptic ulcer.

Falling Asleep During Activities of Daily Living and Somnolence

Patients taking SINEMET alone or with other dopaminergic drugs have reported suddenly falling asleep without prior warning of sleepiness while engaged in activities of daily living (includes operation of motor vehicles). Road traffic accidents attributed to sudden sleep onset have been reported. Although many patients reported somnolence while on dopaminergic medications, there have been reports of road traffic accidents attributed to sudden onset of sleep in which the patient did not perceive any warning signs, such as excessive drowsiness, and believed that they were alert immediately prior to the event. Sudden onset of sleep has been reported to occur as long as one year after the initiation of treatment.

Falling asleep while engaged in activities of daily living usually occurs in patients experiencing pre-existing somnolence, although some patients may not give such a history. For this reason, prescribers should reassess patients for drowsiness or sleepiness especially since some of the events occur well after the start of treatment. Prescribers should be aware that patients may not acknowledge drowsiness or sleepiness until directly questioned about drowsiness or sleepiness during specific activities. Patients should be advised to exercise caution while driving or operating machines during treatment with SINEMET. Patients who have already experienced somnolence or an episode of sudden sleep onset should not participate in these activities during treatment with SINEMET.

Before initiating treatment with SINEMET, advise patients about the potential to develop drowsiness and ask specifically about factors that may increase the risk for somnolence with SINEMET such as the use of concomitant sedating medications and the presence of sleep disorders. Consider discontinuing SINEMET in patients who report significant daytime sleepiness or episodes of falling asleep during

activities that require active participation (e.g., conversations, eating, etc.). If treatment with SINEMET continues, patients should be advised not to drive and to avoid other potentially dangerous activities that might result in harm if the patients become somnolent. There is insufficient information to establish that dose reduction will eliminate episodes of falling asleep while engaged in activities of daily living.

Hyperpyrexia and Confusion

Sporadic cases of a symptom complex resembling neuroleptic malignant syndrome (NMS) have been reported in association with dose reductions or withdrawal of certain antiparkinsonian agents such as levodopa, carbidopa levodopa, or carbidopa levodopa extended release. Therefore, patients should be observed carefully when the dosage of levodopa is reduced abruptly or discontinued, especially if the patient is receiving neuroleptics.

NMS is an uncommon but life-threatening syndrome characterized by fever or hyperthermia. Neurological findings, including muscle rigidity, involuntary movements, altered consciousness, mental status changes; other disturbances, such as autonomic dysfunction, tachycardia, tachypnea, sweating, hyper- or hypotension; laboratory findings, such as creatine phosphokinase elevation, leukocytosis, myoglobinuria, and increased serum myoglobin have been reported.

The early diagnosis of this condition is important for the appropriate management of these patients. Considering NMS as a possible diagnosis and ruling out other acute illnesses (e.g., pneumonia, systemic infection, etc.) is essential. This may be especially complex if the clinical presentation includes both serious medical illness and untreated or inadequately treated extrapyramidal signs and symptoms (EPS). Other important considerations in the differential diagnosis include central anticholinergic toxicity, heat stroke, drug fever, and primary central nervous system (CNS) pathology.

The management of NMS should include: 1) intensive symptomatic treatment and medical monitoring and 2) treatment of any concomitant serious medical problems for which specific treatments are available. Dopamine agonists, such as bromocriptine, and muscle relaxants, such as dantrolene, are often used in the treatment of NMS; however, their effectiveness has not been demonstrated in controlled studies.

PRECAUTIONS

General

As with levodopa, periodic evaluations of hepatic, hematopoietic, cardiovascular, and renal function are recommended during extended therapy.

Patients with chronic wide-angle glaucoma may be treated cautiously with SINEMET provided the intraocular pressure is well-controlled and the patient is monitored carefully for changes in intraocular pressure during therapy.

Dyskinesia

Levodopa alone, as well as SINEMET, is associated with dyskinesias. The occurrence of dyskinesias may require dosage reduction.

Hallucinations / Psychotic-Like Behavior

Hallucinations and psychotic-like behavior have been reported with dopaminergic medications. In general, hallucinations present shortly after the initiation of therapy and may be responsive to dose reduction in levodopa. Hallucinations may be accompanied by confusion and to a lesser extent sleep disorder (insomnia) and excessive dreaming.

SINEMET may have similar effects on thinking and behavior. This abnormal thinking and behavior may present with one or more symptoms, including paranoid ideation, delusions, hallucinations, confusion, psychotic-like behavior, disorientation, aggressive behavior, agitation, and delirium.

Ordinarily, patients with a major psychotic disorder should not be treated with SINEMET, because of the risk of exacerbating psychosis. In addition, certain medications used to treat psychosis may exacerbate the symptoms of Parkinson's disease and may decrease the effectiveness of SINEMET.

Impulse Control / Compulsive Behaviors

Reports of patients taking dopaminergic medications (medications that increase central dopaminergic tone), suggest that patients may experience an intense urge to gamble, increased sexual urges, intense

urges to spend money, binge eating, and/or other intense urges, and the inability to control these urges. In some cases, although not all, these urges were reported to have stopped when the dose was reduced or the medication was discontinued. Because patients may not recognize these behaviors as abnormal, it is important for prescribers to specifically ask patients or the caregivers about the development of new or increased gambling urges, sexual urges, uncontrolled spending or other urges while being treated with SINEMET. Physicians should consider dose reduction or stopping the medication if a patient develops such urges while taking SINEMET [see *Information for Patients*].

Melanoma

Epidemiological studies have shown that patients with Parkinson's disease have a higher risk (2- to approximately 6-fold higher) of developing melanoma than the general population. Whether the increased risk observed was due to Parkinson's disease or other factors, such as drugs used to treat Parkinson's disease, is unclear.

For the reasons stated above, patients and providers are advised to monitor for melanomas frequently and on a regular basis when using SINEMET for any indication. Ideally, periodic skin examinations should be performed by appropriately qualified individuals (e.g., dermatologists).

Information for Patients

The patient should be informed that SINEMET is an immediate-release formulation of carbidopa levodopa that is designed to begin release of ingredients within 30 minutes. It is important that SINEMET be taken at regular intervals according to the schedule outlined by the physician. The patient should be cautioned not to change the prescribed dosage regimen and not to add any additional antiparkinson medications, including other carbidopa levodopa preparations, without first consulting the physician.

Patients should be advised that sometimes a 'wearing-off' effect may occur at the end of the dosing interval. The physician should be notified if such response poses a problem to lifestyle.

Patients should be advised that occasionally, dark color (red, brown, or black) may appear in saliva, urine, or sweat after ingestion of SINEMET. Although the color appears to be clinically insignificant, garments may become discolored.

The patient should be advised that a change in diet to foods that are high in protein may delay the absorption of levodopa and may reduce the amount taken up in the circulation. Excessive acidity also delays stomach emptying, thus delaying the absorption of levodopa. Iron salts (such as in multivitamin tablets) may also reduce the amount of levodopa available to the body. The above factors may reduce the clinical effectiveness of the levodopa or carbidopa levodopa therapy.

Patients should be alerted to the possibility of sudden onset of sleep during daily activities, in some cases without awareness or warning signs, when they are taking dopaminergic agents, including levodopa. Patients should be advised to exercise caution while driving or operating machinery and that if they have experienced somnolence and/or sudden sleep onset, they must refrain from these activities. (See WARNINGS, Falling Asleep During Activities of Daily Living and Somnolence.)

There have been reports of patients experiencing intense urges to gamble, increased sexual urges, and other intense urges, and the inability to control these urges while taking one or more of the medications that increase central dopaminergic tone and that are generally used for the treatment of Parkinson's disease, including SINEMET. Although it is not proven that the medications caused these events, these urges were reported to have stopped in some cases when the dose was reduced or the medication was stopped. Prescribers should ask patients about the development of new or increased gambling urges, sexual urges or other urges while being treated with SINEMET. Patients should inform their physician if they experience new or increased gambling urges, increased sexual urges, or other intense urges while taking SINEMET. Physicians should consider dose reduction or stopping the medication if a patient develops such urges while taking SINEMET (See PRECAUTIONS, Impulse Control / Compulsive Behaviors).

Laboratory Tests

Abnormalities in laboratory tests may include elevations of liver function tests such as alkaline phosphatase, SGOT (AST), SGPT (ALT), lactic dehydrogenase (LDH), and bilirubin. Abnormalities in blood urea nitrogen (BUN) and positive Coombs test have also been reported. Commonly, levels of blood urea nitrogen, creatinine, and uric acid are lower during administration of SINEMET than with levodopa.

SINEMET may cause a false-positive reaction for urinary ketone bodies when a test tape is used for determination of ketonuria. This reaction will not be altered by boiling the urine specimen. False-negative tests may result with the use of glucose-oxidase methods of testing for glucosuria.

Cases of falsely diagnosed pheochromocytoma in patients on carbidopa levodopa therapy have been reported very rarely. Caution should be exercised when interpreting the plasma and urine levels of catecholamines and their metabolites in patients on levodopa or carbidopa levodopa therapy.

Drug Interactions

Caution should be exercised when the following drugs are administered concomitantly with SINEMET.

Symptomatic postural hypotension occurred when SINEMET was added to the treatment of a patient receiving antihypertensive drugs. Therefore, when therapy with SINEMET is started, dosage adjustment of the antihypertensive drug may be required.

For patients receiving MAO inhibitors (Type A or B), see CONTRAINDICATIONS. Concomitant therapy with selegiline and carbidopa levodopa may be associated with severe orthostatic hypotension not attributable to carbidopa levodopa alone (see CONTRAINDICATIONS).

There have been rare reports of adverse reactions, including hypertension and dyskinesia, resulting from the concomitant use of tricyclic antidepressants and SINEMET.

Dopamine D_2 receptor antagonists (e.g., phenothiazines, butyrophenones, risperidone) and isoniazid may reduce the therapeutic effects of levodopa. In addition, the beneficial effects of levodopa in Parkinson's disease have been reported to be reversed by phenytoin and papaverine. Patients taking these drugs with SINEMET should be carefully observed for loss of therapeutic response.

Use of SINEMET with dopamine-depleting agents (e.g., reserpine and tetrabenazine) or other drugs known to deplete monoamine stores is not recommended.

SINEMET and iron salts or multivitamins containing iron salts should be coadministered with caution. Iron salts can form chelates with levodopa and carbidopa and consequently reduce the bioavailability of carbidopa and levodopa.

Although metoclopramide may increase the bioavailability of levodopa by increasing gastric emptying, metoclopramide may also adversely affect disease control by its dopamine receptor antagonistic properties.

Carcinogenesis, Mutagenesis, Impairment of Fertility

In a two-year bioassay of SINEMET, no evidence of carcinogenicity was found in rats receiving doses of approximately two times the maximum daily human dose of carbidopa and four times the maximum daily human dose of levodopa.

In reproduction studies with SINEMET, no effects on fertility were found in rats receiving doses of approximately two times the maximum daily human dose of carbidopa and four times the maximum daily human dose of levodopa.

Pregnancy

Pregnancy Category C. No teratogenic effects were observed in a study in mice receiving up to 20 times the maximum recommended human dose of SINEMET. There was a decrease in the number of live pups delivered by rats receiving approximately two times the maximum recommended human dose of carbidopa and approximately five times the maximum recommended human dose of levodopa during organogenesis. SINEMET caused both visceral and skeletal malformations in rabbits at all doses and ratios of carbidopa/levodopa tested, which ranged from 10 times/5 times the maximum recommended human dose of carbidopa/levodopa to 20 times/10 times the maximum recommended human dose of carbidopa/levodopa.

There are no adequate or well-controlled studies in pregnant women. It has been reported from individual cases that levodopa crosses the human placental barrier, enters the fetus, and is metabolized. Carbidopa concentrations in fetal tissue appeared to be minimal. Use of SINEMET in women of childbearing potential requires that the anticipated benefits of the drug be weighed against possible hazards to mother and child.

Nursing Mothers

Levodopa has been detected in human milk. Caution should be exercised when SINEMET is administered to a nursing woman.

Pediatric Use

Safety and effectiveness in pediatric patients have not been established. Use of the drug in patients below the age of 18 is not recommended.

Geriatric Use

In the clinical efficacy trials for SINEMET, almost half of the patients were older than 65, but few were older than 75. No overall meaningful differences in safety or effectiveness were observed between these subjects and younger subjects, but greater sensitivity of some older individuals to adverse drug reactions such as hallucinations cannot be ruled out. There is no specific dosing recommendation based upon clinical pharmacology data as SINEMET is titrated as tolerated for clinical effect.

ADVERSE REACTIONS

The most common adverse reactions reported with SINEMET have included dyskinesias, such as choreiform, dystonic, and other involuntary movements, and nausea.

The following other adverse reactions have been reported with SINEMET:

Body as a Whole

Chest pain, asthenia.

Cardiovascular

Cardiac irregularities, hypotension, orthostatic effects including orthostatic hypotension, hypertension, syncope, phlebitis, palpitation.

Gastrointestinal

Dark saliva, gastrointestinal bleeding, development of duodenal ulcer, anorexia, vomiting, diarrhea, constipation, dyspepsia, dry mouth, taste alterations.

Hematologic

Agranulocytosis, hemolytic and non-hemolytic anemia, thrombocytopenia, leukopenia.

Hypersensitivity

Angioedema, urticaria, pruritus, Henoch-Schönlein purpura, bullous lesions (including pemphigus-like reactions).

Musculoskeletal

Back pain, shoulder pain, muscle cramps.

Nervous System/Psychiatric

Psychotic episodes including delusions, hallucinations, and paranoid ideation, bradykinetic episodes ("on-off" phenomenon), confusion, agitation, dizziness, somnolence, dream abnormalities including nightmares, insomnia, paresthesia, headache, depression with or without development of suicidal tendencies, dementia, pathological gambling, increased libido including hypersexuality, impulse control symptoms. Convulsions also have occurred; however, a causal relationship with SINEMET has not been established.

Respiratory

Dyspnea, upper respiratory infection.

Skin

Rash, increased sweating, alopecia, dark sweat.

Urogenital

Urinary tract infection, urinary frequency, dark urine.

Laboratory Tests

Decreased hemoglobin and hematocrit; abnormalities in alkaline phosphatase, SGOT (AST), SGPT (ALT), LDH, bilirubin, BUN, Coombs test; elevated serum glucose; white blood cells, bacteria, and blood in the urine.

Other adverse reactions that have been reported with levodopa alone and with various carbidopa levodopa formulations, and may occur with SINEMET are:

Body as a Whole

Abdominal pain and distress, fatigue.

Cardiovascular
Myocardial infarction.
Gastrointestinal
Gastrointestinal pain, dysphagia, sialorrhea, flatulence, bruxism, burning sensation of the tongue, heartburn, hiccups.
Metabolic
Edema, weight gain, weight loss.
Musculoskeletal
Leg pain.
Nervous System/Psychiatric
Ataxia, extrapyramidal disorder, falling, anxiety, gait abnormalities, nervousness, decreased mental acuity, memory impairment, disorientation, euphoria, blepharospasm (which may be taken as an early sign of excess dosage; consideration of dosage reduction may be made at this time), trismus, increased tremor, numbness, muscle twitching, activation of latent Horner's syndrome, peripheral neuropathy.
Respiratory
Pharyngeal pain, cough.
Skin
Malignant melanoma (see also CONTRAINDICATIONS), flushing.
Special Senses
Oculogyric crises, diplopia, blurred vision, dilated pupils.
Urogenital
Urinary retention, urinary incontinence, priapism.
Miscellaneous
Bizarre breathing patterns, faintness, hoarseness, malaise, hot flashes, sense of stimulation.
Laboratory Tests
Decreased white blood cell count and serum potassium; increased serum creatinine and uric acid; protein and glucose in urine.

OVERDOSAGE

Management of acute overdosage with SINEMET is the same as management of acute overdosage with levodopa. Pyridoxine is not effective in reversing the actions of SINEMET.

General supportive measures should be employed, along with immediate gastric lavage. Intravenous fluids should be administered judiciously and an adequate airway maintained. Electrocardiographic monitoring should be instituted and the patient carefully observed for the development of arrhythmias; if required, appropriate antiarrhythmic therapy should be given. The possibility that the patient may have taken other drugs as well as SINEMET should be taken into consideration. To date, no experience has been reported with dialysis; hence, its value in overdosage is not known.

Based on studies in which high doses of levodopa and/or carbidopa were administered, a significant proportion of rats and mice given single oral doses of levodopa of approximately 1500-2000 mg/kg are expected to die. A significant proportion of infant rats of both sexes are expected to die at a dose of 800 mg/kg. A significant proportion of rats are expected to die after treatment with similar doses of carbidopa. The addition of carbidopa in a 1:10 ratio with levodopa increases the dose at which a significant proportion of mice are expected to die to 3360 mg/kg.

DOSAGE AND ADMINISTRATION

The optimum daily dosage of SINEMET must be determined by careful titration in each patient. SINEMET tablets are available in a 1:4 ratio of carbidopa to levodopa (SINEMET 25-100) as well as 1:10 ratio (SINEMET 25-250 and SINEMET 10-100). Tablets of the two ratios may be given separately or combined as needed to provide the optimum dosage.

Studies show that peripheral dopa decarboxylase is saturated by carbidopa at approximately 70 to 100 mg a day. Patients receiving less than this amount of carbidopa are more likely to experience nausea and vomiting.

Usual Initial Dosage

Dosage is best initiated with one tablet of SINEMET 25-100 three times a day. This dosage schedule provides 75 mg of carbidopa per day. Dosage may be increased by one tablet every day or every other day, as necessary, until a dosage of eight tablets of SINEMET 25-100 a day is reached.

If SINEMET 10-100 is used, dosage may be initiated with one tablet three or four times a day. However, this will not provide an adequate amount of carbidopa for many patients. Dosage may be increased by one tablet every day or every other day until a total of eight tablets (2 tablets q.i.d.) is reached.

How to Transfer Patients from Levodopa

Levodopa must be discontinued at least twelve hours before starting SINEMET. A daily dosage of SINEMET should be chosen that will provide approximately 25% of the previous levodopa dosage. Patients who are taking less than 1500 mg of levodopa a day should be started on one tablet of SINEMET 25-100 three or four times a day. The suggested starting dosage for most patients taking more than 1500 mg of levodopa is one tablet of SINEMET 25-250 three or four times a day.

Maintenance

Therapy should be individualized and adjusted according to the desired therapeutic response. At least 70 to 100 mg of carbidopa per day should be provided. When a greater proportion of carbidopa is required, one tablet of SINEMET 25-100 may be substituted for each tablet of SINEMET 10-100. When more levodopa is required, SINEMET 25-250 should be substituted for SINEMET 25-100 or SINEMET 10-100. If necessary, the dosage of carbidopa levodopa 25-250 may be increased by one-half or one tablet every day or every other day to a maximum of eight tablets a day. Experience with total daily dosages of carbidopa greater than 200 mg is limited.

Because both therapeutic and adverse responses occur more rapidly with SINEMET than with levodopa alone, patients should be monitored closely during the dose adjustment period. Specifically, involuntary movements will occur more rapidly with SINEMET than with levodopa. The occurrence of involuntary movements may require dosage reduction. Blepharospasm may be a useful early sign of excess dosage in some patients.

Addition of Other Antiparkinsonian Medications

Standard drugs for Parkinson's disease, other than levodopa without a decarboxylase inhibitor, may be used concomitantly while SINEMET is being administered, although dosage adjustments may be required.

Interruption of Therapy

Sporadic cases of hyperpyrexia and confusion have been associated with dose reductions and withdrawal of SINEMET. Patients should be observed carefully if abrupt reduction or discontinuation of SINEMET is required, especially if the patient is receiving neuroleptics. (See WARNINGS.)

If general anesthesia is required, SINEMET may be continued as long as the patient is permitted to take fluids and medication by mouth. If therapy is interrupted temporarily, the patient should be observed for symptoms resembling NMS, and the usual daily dosage may be administered as soon as the patient is able to take oral medication.

HOW SUPPLIED

No. 3916A — SINEMET 25-100 Tablets are yellow, round, uncoated tablets, that are coded "650" on one side and plain on the other. They are supplied as follows:

NDC 0006-3916-68 bottles of 100.

No. 3915 — SINEMET 10-100 Tablets are light dapple-blue, round, uncoated tablets, that are coded "647" on one side and plain on the other. They are supplied as follows:

NDC 0006-3915-68 bottles of 100.

No. 3917 — SINEMET 25-250 Tablets are light dapple-blue, round, uncoated tablets, that are coded "654" on one side and plain on the other. They are supplied as follows:

NDC 0006-3917-68 bottles of 100.

Storage and Handling

Store at 25°C (77°F), excursions permitted to 15-30°C (59-86°F) [see USP Controlled Room Temperature]. Store in a tightly closed container, protected from light and moisture.

Dispense in a tightly closed, light-resistant container.

Manufactured for: Merck Sharp & Dohme Corp., a subsidiary of
MERCK & CO., INC., Whitehouse Station, NJ 08889, USA

Manufactured by:
Mylan Pharmaceuticals, Inc.
Morgantown, WV 26505, USA

Copyright © 1996-2014 Merck Sharp & Dohme Corp., a subsidiary of **Merck & Co., Inc.**
All rights reserved.

Revised: 07/2014

uspi-mk0295b-t-1407r003

Rx Only

APPENDIX B. INSTRUCTIONS FOR DOSE CONVERSION TO IPX203

The goal of the dose conversion period is to establish a dosing regimen for IPX203 that minimizes "Off" time without causing troublesome dyskinesias.

The initial dose of IPX203 is based on the subject's most frequent IR CD-LD dose established during the 3-week IR CD-LD dose adjustment period.

Table B-1 Recommended Starting IPX203 LD Dosing Regimen Based on the Dosing Regimen of IR CD-LD at the End of the Dose Adjustment Period

Most Frequent IR CD-LD Unit Dose (mg)	Recommended Starting IPX203 Daily Dosing Regimen CD-LD (mg) Every 8 Hours
25-100[a]	70-280 mg (2 × 35-140 mg)
>25-100 – 37.5-150	105-420 mg (3 × 35-140 mg)
>37.5-150 – 50-200	140-560 mg (4 × 35-140 mg)
>50-200	175-700 mg (5 × 35-140 mg)

[a] Subjects who are on a total daily dose of less than 125-500 mg CD-LD from IR CD-LD should be advised to initially take IPX203 every 12 hours. The dosing interval may be reduced to approximately every 8 hours if the subject does not achieve an acceptable duration of effect.

Conversion Instructions:

1. Convert the subject's most frequent daily dose of IR CD-LD to the corresponding dose of IPX203 according to the above table. It is recommended that the subject takes IPX203 doses approximately every 8 hours apart (for example, a subject may take IPX203 at 6 AM, 2 PM, and 10 PM). Some subjects may benefit from a shorter or longer dosing interval. The dosing interval may vary but should not be more frequent than every 6 hours.
2. Subjects who are on a total daily dose of less than 125-500 mg CD-LD from IR CD-LD should be advised to initially take IPX203 every 12 hours. The dosing interval may be reduced to approximately every 8 hours if the subject does not achieve an acceptable duration of effect.
3. The Investigator or their staff are advised to be in telephone contact with the subject, especially during the initial dose conversion to assess the need for dosage adjustment with the goal of minimizing "Off" time without causing troublesome dyskinesia or other dopaminergic side effects. Calls to the subject can be reduced appropriately when the subject reaches a stable dosing regimen.
4. If dose adjustment is necessary, consider the following options recognizing that the number of capsules at each dose may be varied to achieve an optimal response.

   a. If turning “On” is slow following the first morning dose, consider taking the morning IPX203 dose in the fasted state and/or increasing the dose by one capsule (35-140 mg IPX203 CD-LD).
   b. If turning “On” is slow later in the day or to reduce “end-of-dose” “Off” time, consider increasing the dose by one capsule (35-140 mg IPX203 CD-LD) before reducing the dosing interval.

5. In case of troublesome dyskinesias, use the following guidelines:
   a. Consider reducing the dose by one capsule (35-140 mg IPX203 CD-LD).
   b. Consider increasing the dosing interval.
6. The subject must be on a stable dosing regimen of IPX203 (no change in dose or in dosing frequency) for at least 5 days prior to Visit 4 (randomization).

APPENDIX C. UNITED KINGDOM PARKINSON'S DISEASE SOCIETY BRAIN BANK DIAGNOSTIC CRITERIA FOR PARKINSON'S DISEASE

**Step 1: Diagnosis of Parkinsonism**
Bradykinesia **and** at least one of the following:<br>• Muscular rigidity<br>• 4–6 Hz resting tremor<br>• postural instability not caused by primary visual, vestibular, cerebellar or Proprioceptive dysfunction
**Step 2: Features tending to exclude Parkinson's disease as the cause of Parkinsonism**
• History of repeated strokes with stepwise progression of parkinsonian features<br>• History of repeated head injury<br>• History of definite encephalitis<br>• Neuroleptic treatment at onset of symptoms<br>• >1 affected relatives<br>• Sustained remission<br>• Strictly unilateral features after 3 years<br>• Supranuclear gaze palsy<br>• Cerebellar signs<br>• Early severe autonomic involvement<br>• Early severe dementia with disturbances of memory, language and praxis<br>• Babinski's sign<br>• Presence of a cerebral tumour or communicating hydrocephalus on computed tomography scan<br>• Negative response to large doses of levodopa (if malabsorption excluded)<br>• MPTP exposure
**Step 3: Features that support a diagnosis of Parkinson's disease (three or more required for diagnosis of definite Parkinson's disease)**
• Unilateral onset<br>• Rest tremor present<br>• Progressive disorder<br>• Persistent asymmetry affecting the side of onset most<br>• Excellent (70–100%) response to levodopa<br>• Severe levodopa-induced chorea<br>• Levodopa response for ≥5 years<br>• Clinical course of ≥10 years

APPENDIX D. MONTREAL COGNITIVE ASSESSMENT (MOCA)

Montreal Cognitive Assessment
(MoCA)

Administration and Scoring Instructions

The Montreal Cognitive Assessment (MoCA) was designed as a rapid screening instrument for mild cognitive dysfunction. It assesses different cognitive domains: attention and concentration, executive functions, memory, language, visuoconstructional skills, conceptual thinking, calculations, and orientation. Time to administer the MoCA is approximately 10 minutes. The total possible score is 30 points; a score of 26 or above is considered normal.

1. Alternating Trail Making:

Administration: The examiner instructs the subject: *"Please draw a line, going from a number to a letter in ascending order. Begin here* [point to (1)] *and draw a line from 1 then to A then to 2 and so on. End here* [point to (E)]."

Scoring: Allocate one point if the subject successfully draws the following pattern:
1 –A- 2- B- 3- C- 4- D- 5- E, without drawing any lines that cross. Any error that is not immediately self-corrected earns a score of 0.

2. Visuoconstructional Skills (Cube):

Administration: The examiner gives the following instructions, pointing to the **cube**: "*Copy this drawing as accurately as you can, in the space below*".

Scoring: One point is allocated for a correctly executed drawing.
• Drawing must be three-dimensional
• All lines are drawn
• No line is added
• Lines are relatively parallel and their length is similar (rectangular prisms are accepted)

A point is not assigned if any of the above-criteria are not met.

3. Visuoconstructional Skills (Clock):

Administration: Indicate the right third of the space and give the following instructions: *"Draw a clock. Put in all the numbers and set the time to 10 past 11".*

Scoring: One point is allocated for each of the following three criteria:
• Contour (1 pt.): the clock face must be a circle with only minor distortion acceptable (e.g., slight imperfection on closing the circle);
• Numbers (1 pt.): all clock numbers must be present with no additional numbers; numbers must be in the correct order and placed in the approximate quadrants on the clock face; Roman numerals are acceptable; numbers can be placed outside the circle contour;
• Hands (1 pt.): there must be two hands jointly indicating the correct time; the hour hand must be clearly shorter than the minute hand; hands must be centred within the clock face with their junction close to the clock centre.

A point is not assigned for a given element if any of the above-criteria are not met.

© Z. Nasreddine MD

4. Naming:

Administration: Beginning on the left, point to each figure and say: *"Tell me the name of this animal"*.

Scoring: One point each is given for the following responses: (1) lion (2) rhinoceros or rhino (3) camel or dromedary.

5. Memory:

Administration: The examiner reads a list of 5 words at a rate of one per second, giving the following instructions: *"This is a memory test. I am going to read a list of words that you will have to remember now and later on. Listen carefully. When I am through, tell me as many words as you can remember. It doesn't matter in what order you say them"*. Mark a check in the allocated space for each word the subject produces on this first trial. When the subject indicates that (s)he has finished (has recalled all words), or can recall no more words, read the list a second time with the following instructions: *"I am going to read the same list for a second time. Try to remember and tell me as many words as you can, including words you said the first time."* Put a check in the allocated space for each word the subject recalls after the second trial.
At the end of the second trial, inform the subject that (s)he will be asked to recall these words again by saying, *"I will ask you to recall those words again at the end of the test."*

Scoring: No points are given for Trials One and Two.

6. Attention:

Forward Digit Span: Administration: Give the following instruction: "*I am going to say some numbers and when I am through, repeat them to me exactly as I said them*". Read the five number sequence at a rate of one digit per second.

Backward Digit Span: Administration: Give the following instruction: "*Now I am going to say some more numbers, but when I am through you must repeat them to me in the backwards order.*" Read the three number sequence at a rate of one digit per second.

Scoring: Allocate one point for each sequence correctly repeated, (*N.B.*: the correct response for the backwards trial is 2-4-7).

Vigilance: Administration: The examiner reads the list of letters at a rate of one per second, after giving the following instruction: "*I am going to read a sequence of letters. Every time I say the letter A, tap your hand once. If I say a different letter, do not tap your hand*".

Scoring: Give one point if there is zero to one errors (an error is a tap on a wrong letter or a failure to tap on letter A).

© Z. Nasreddine MD
www.mocatest.org

Serial 7s: Administration: The examiner gives the following instruction: *"Now, I will ask you to count by subtracting seven from 100, and then, keep subtracting seven from your answer until I tell you to stop."* Give this instruction twice if necessary.

Scoring: This item is scored out of 3 points. Give no (0) points for no correct subtractions, 1 point for one correction subtraction, 2 points for two-to-three correct subtractions, and 3 points if the participant successfully makes four or five correct subtractions. Count each correct subtraction of 7 beginning at 100. Each subtraction is evaluated independently; that is, if the participant responds with an incorrect number but continues to correctly subtract 7 from it, give a point for each correct subtraction. For example, a participant may respond "92 – 85 – 78 – 71 – 64" where the "92" is incorrect, but all subsequent numbers are subtracted correctly. This is one error and the item would be given a score of 3.

7. **Sentence repetition:**

Administration: The examiner gives the following instructions: *"I am going to read you a sentence. Repeat it after me, exactly as I say it* [pause]: ***I only know that John is the one to help today."*** Following the response, say: *"Now I am going to read you another sentence. Repeat it after me, exactly as I say it* [pause]: ***The cat always hid under the couch when dogs were in the room."***

Scoring: Allocate 1 point for each sentence correctly repeated. Repetition must be exact. Be alert for errors that are omissions (e.g., omitting "only", "always") and substitutions/additions (e.g., "John is the one who helped today;" substituting "hides" for "hid", altering plurals, etc.).

8. **Verbal fluency:**

Administration: The examiner gives the following instruction: *"Tell me as many words as you can think of that begin with a certain letter of the alphabet that I will tell you in a moment. You can say any kind of word you want, except for proper nouns (like Bob or Boston), numbers, or words that begin with the same sound but have a different suffix, for example, love, lover, loving. I will tell you to stop after one minute. Are you ready?* [Pause] *Now, tell me as many words as you can think of that begin with the letter F.* [time for 60 sec]. *Stop."*

Scoring: Allocate one point if the subject generates 11 words or more in 60 sec. Record the subject's response in the bottom or side margins.

9. **Abstraction:**

Administration: The examiner asks the subject to explain what each pair of words has in common, starting with the example: *"Tell me how an orange and a banana are alike".* If the subject answers in a concrete manner, then say only one additional time: *"Tell me another way in which those items are alike".* If the subject does not give the appropriate response *(fruit)*, say, *"Yes, and they are also both fruit."* Do not give any additional instructions or clarification. After the practice trial, say: *"Now, tell me how a train and a bicycle are alike".* Following the response, administer the second trial, saying: *"Now tell me how a ruler and a watch are alike".* Do not give any additional instructions or prompts.

© Z. Nasreddine MD

Scoring: Only the last two item pairs are scored. Give 1 point to each item pair correctly answered. The following responses are acceptable:
Train-bicycle = means of transportation, means of travelling, you take trips in both;
Ruler-watch = measuring instruments, used to measure.
The following responses are **not** acceptable: Train-bicycle = they have wheels; Ruler-watch = they have numbers.

10. Delayed recall:

Administration: The examiner gives the following instruction: *"I read some words to you earlier, which I asked you to remember. Tell me as many of those words as you can remember."* Make a check mark (√) for each of the words correctly recalled spontaneously without any cues, in the allocated space.

Scoring: **Allocate 1 point for each word recalled freely without any cues.**

Optional:
Following the delayed free recall trial, prompt the subject with the semantic category cue provided below for any word not recalled. Make a check mark (√) in the allocated space if the subject remembered the word with the help of a category or multiple-choice cue. Prompt all non-recalled words in this manner. If the subject does not recall the word after the category cue, give him/her a multiple choice trial, using the following example instruction, *"Which of the following words do you think it was, NOSE, FACE, or HAND?"*
Use the following category and/or multiple-choice cues for each word, when appropriate:

FACE:	category cue: part of the body	multiple choice: nose, face, hand
VELVET:	category cue: type of fabric	multiple choice: denim, cotton, velvet
CHURCH:	category cue: type of building	multiple choice: church, school, hospital
DAISY:	category cue: type of flower	multiple choice: rose, daisy, tulip
RED:	category cue: a colour	multiple choice: red, blue, green

Scoring: **No points are allocated for words recalled with a cue.** A cue is used for clinical information purposes only and can give the test interpreter additional information about the type of memory disorder. For memory deficits due to retrieval failures, performance can be improved with a cue. For memory deficits due to encoding failures, performance does not improve with a cue.

11. Orientation:

Administration: The examiner gives the following instructions: "Tell me the date today". If the subject does not give a complete answer, then prompt accordingly by saying: *"Tell me the [year, month, exact date, and day of the week]."* Then say: *"Now, tell me the name of this place, and which city it is in."*

Scoring: Give one point for each item correctly answered. The subject must tell the exact date and the exact place (name of hospital, clinic, office). No points are allocated if subject makes an error of one day for the day and date.

TOTAL SCORE: Sum all subscores listed on the right-hand side. Add one point for an individual who has 12 years or fewer of formal education, for a possible maximum of 30 points. A final total score of 26 and above is considered normal.

NAME :
Education : Date of birth :
Sex : DATE :

MONTREAL COGNITIVE ASSESSMENT (MOCA)

Version 7.1 Original Version

VISUOSPATIAL / EXECUTIVE

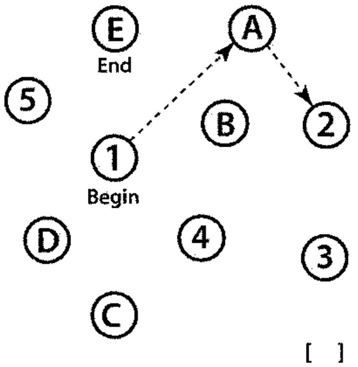

[ ]

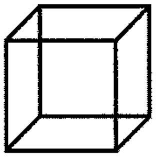

Copy cube

[ ]

Draw CLOCK (Ten past eleven)
(3 points)

[ ] Contour [ ] Numbers [ ] Hands

POINTS: ___/5

NAMING

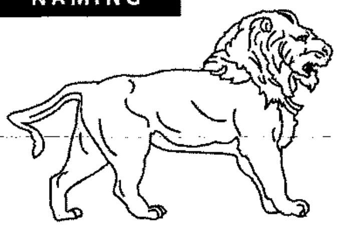

[ ]

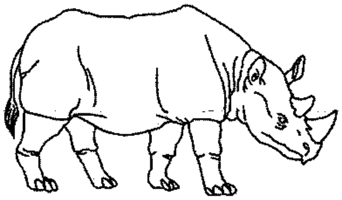

[ ]

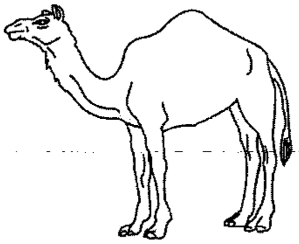

[ ]

___/3

MEMORY

Read list of words, subject must repeat them. Do 2 trials, even if 1st trial is successful. Do a recall after 5 minutes.

	FACE	VELVET	CHURCH	DAISY	RED
1st trial					
2nd trial					

No points

ATTENTION

Read list of digits (1 digit/ sec.).
Subject has to repeat them in the forward order [ ] 2 1 8 5 4
Subject has to repeat them in the backward order [ ] 7 4 2
___/2

Read list of letters. The subject must tap with his hand at each letter A. No points if ≥ 2 errors
[ ] F B A C M N A A J K L B A F A K D E A A A J A M O F A A B
___/1

Serial 7 subtraction starting at 100 [ ] 93 [ ] 86 [ ] 79 [ ] 72 [ ] 65
4 or 5 correct subtractions: **3 pts**, 2 or 3 correct: **2 pts**, 1 correct: **1 pt**, 0 correct: **0 pt**
___/3

LANGUAGE

Repeat : I only know that John is the one to help today. [ ]
The cat always hid under the couch when dogs were in the room. [ ]
___/2

Fluency / Name maximum number of words in one minute that begin with the letter F [ ] _____ (N ≥ 11 words)
___/1

ABSTRACTION

Similarity between e.g. banana - orange = fruit [ ] train – bicycle [ ] watch - ruler
___/2

DELAYED RECALL

	FACE	VELVET	CHURCH	DAISY	RED	
Has to recall words WITH NO CUE	[ ]	[ ]	[ ]	[ ]	[ ]	Points for UNCUED recall only
**Optional** Category cue						
Multiple choice cue						

___/5

ORIENTATION

[ ] Date [ ] Month [ ] Year [ ] Day [ ] Place [ ] City
___/6

© Z.Nasreddine MD **www.mocatest.org** Normal ≥26 / 30 TOTAL ___/30
Add 1 point if ≤ 12 yr edu

Administered by:________________________________

APPENDIX E. MOVEMENT DISORDERS SOCIETY VERSION OF UNIFIED PARKINSON'S DISEASE RATING SCALE (MDS-UPDRS)

MDS-UPDRS Permissions

Permission is required to use the MDS-developed Rating Scales (with the exception of personal/individual use). Reproduction, translation, modification, sale, or distribution of any portion of the MDS Rating Scales is strictly prohibited. MDS Rating Scales may not be incorporated into clinical trials, training or certification programs or materials, software programs, or otherwise except through use of the Permissions Request Form and payment of applicable fees.

Continue to p. 2 to view the MDS-UPDRS

MDS-UPDRS

The *Movement* Disorder Society (MDS)-sponsored new version of the UPDRS is founded on the critique that was formulated by the Task Force for Rating Scales in Parkinson's disease (*Mov Disord* 2003;18:738-750). Thereafter, the MDS recruited a Chairperson to organize a program to provide the Movement Disorder community with a new version of the UPDRS that would maintain the overall format of the original UPDRS, but address issues identified in the critique as weaknesses and ambiguities. The Chairperson identified subcommittees with chairs and members. Each part was written by the appropriate subcommittee members and then reviewed and ratified by the entire group. These members are listed below.

The MDS-UPDRS has four parts: Part I (non-motor experiences of daily living), Part II (motor experiences of daily living, Part III (motor examination) and Part IV (motor complications). Part I has two components: IA concerns a number of behaviors that are assessed by the investigator with all pertinent information from patients and caregivers, and IB is completed by the patient with or without the aid of the caregiver, but independently of the investigator. These sections can, however, be reviewed by the rater to ensure that all questions are answered clearly and the rater can help explain any perceived ambiguities. Part II is designed to be a self-administered questionnaire like Part IB, but can be reviewed by the investigator to ensure completeness and clarity. Of note, the official versions of Part IA, Part IB and Part II of the MDS-UPDRS do not have separate on or off ratings. However, for individual programs or protocols the same questions can be used separately for on and off. Part III has instructions for the rater to give or demonstrate to the patient; it is completed by the rater. Part IV has instructions for the rater and also instructions to be read to the patient. This part integrates patient-derived information with the rater's clinical observations and judgments and is completed by the rater.

The authors of this new version are:

Chairperson: Christopher G. Goetz
Part I: Werner Poewe (chair), Bruno Dubois, Anette Schrag
Part II: Matthew B. Stern (chair), Anthony E. Lang, Peter A. LeWitt
Part III: Stanley Fahn (chair), Joseph Jankovic, C. Warren Olanow
Part IV: Pablo Martinez-Martin (chair), Andrew Lees, Olivier Rascol, Bob van Hilten
Development Standards: Glenn T. Stebbins (chair), Robert Holloway, David Nyenhuis
Appendices: Cristina Sampaio (chair), Richard Dodel, Jaime Kulisevsky
Statistical Testing: Barbara Tilley (chair), Sue Leurgans, Jean Teresi,
Consultant: Stephanie Shaftman, Nancy LaPelle

Contact person: Christopher G. Goetz, MD
Rush University Medical Center
1725 W. Harrison Street, Suite 755
Chicago, IL USA 60612

Telephone 312-942-8016
Email: cgoetz@rush.edu

July 1, 2008

Copyright © 2008 Movement Disorder Society. All rights reserved.
This chart may not be copied, distributed or otherwise used in whole or in part without prior written consent of the Movement Disorder Society.

Part I: Non-Motor Aspects of Experiences of Daily Living (nM-EDL)

Overview: This portion of the scale assesses the non-motor impact of Parkinson's disease (PD) on patients' experiences of daily living. There are 13 questions. Part 1A is administered by the rater (six questions) and focuses on complex behaviors. Part 1B is a component of the self-administered Patient Questionnaire that covers seven questions on non-motor experiences of daily living.

Part 1A:
In administering Part IA, the examiner should use the following guidelines:

1. Mark at the top of the form the primary data source as patient, caregiver, or patient and caregiver in equal proportion.
2. The response to each item should refer to a period encompassing the prior week including the day on which the information is collected.
3. All items must have an integer rating (no half points, no missing scores). In the event that an item does not apply or cannot be rated (e.g., amputee who cannot walk), the item is marked UR for Unable to Rate.
4. The answers should reflect the usual level of function and words such as "usually", "generally", "most of the time" can be used with patients.
5. Each question has a text for you to read (Instructions to patients/caregiver). After that statement, you can elaborate and probe based on the target symptoms outlined in the Instructions to examiner. You should NOT READ the RATING OPTIONS to the patient/caregiver, because these are written in medical terminology. From the interview and probing, you will use your medical judgment to arrive at the best response.
6. Patients may have co-morbidities and other medical conditions that can affect their function. You and the patient must rate the problem as it exists and do not attempt to separate elements due to Parkinson's disease from other conditions.

EXAMPLE OF NAVIGATING THROUGH THE RESPONSE OPTIONS FOR PART 1A

Suggested strategies for obtaining the most accurate answer:
After reading the instructions to the patient, you will need to probe the entire domain under discussion to determine Normal vs. problematic: If your questions do not identify any problem in this domain, record 0 and move on to the next question.

If your questions identify a problem in this domain, you should work next with a reference anchor at the mid-range (option 2 or Mild) to find out if the patient functions at this level, better or worse. <u>You will not be reading the choices of responses to the patient as the responses use clinical terminology. You will be asking enough probing questions to determine the response that should be coded.</u>

Work up and down the options with the patient to identify the most accurate response, giving a final check by excluding the options above and below the selected response.

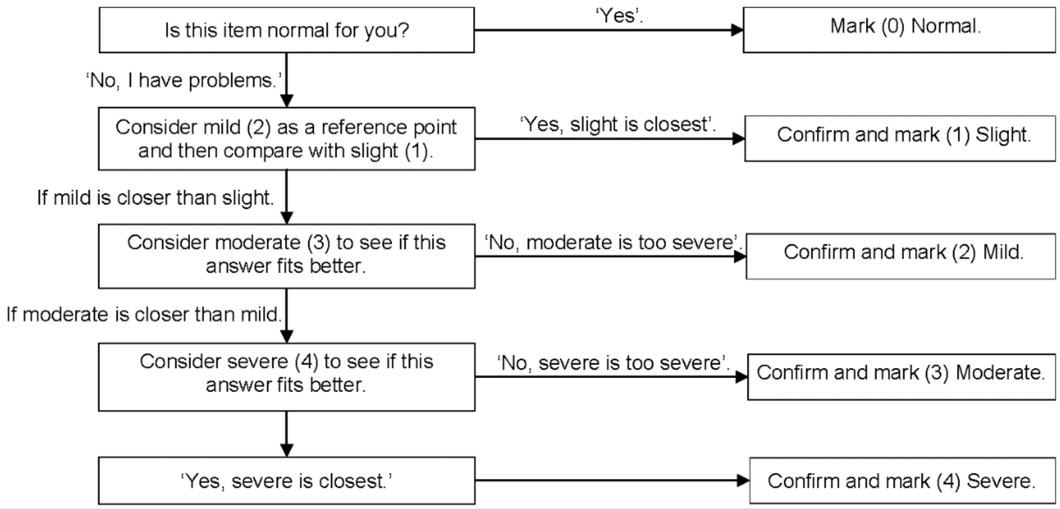

July 1, 2008 *Copyright © 2008 Movement Disorder Society. All rights reserved.* Page 2

This chart may not be copied, distributed or otherwise used in whole or in part without prior written consent of the Movement Disorder Society.

___ ___ ___ ___ ___ ___ ___ ___ Patient Name or Subject ID	___ ___ ___ Site ID	___ ___ - ___ ___ - ___ ___ ___ ___ (mm-dd-yyyy) Assessment Date	___ ___ ___ Investigator's Initials

MDS UPDRS
Part I: Non-Motor Aspects of Experiences of Daily Living (nM-EDL)

Part 1A: Complex behaviors: [completed by rater]

Primary source of information:

☐ Patient ☐ Caregiver ☐ Patient and Caregiver in Equal Proportion

To be read to the patient: I am going to ask you six questions about behaviors that you may or may not experience. Some questions concern common problems and some concern uncommon ones. If you have a problem in one of the areas, please choose the best response that describes how you have felt MOST OF THE TIME during the PAST WEEK. If you are not bothered by a problem, you can simply respond NO. I am trying to be thorough, so I may ask questions that have nothing to do with you.

1.1 COGNITIVE IMPAIRMENT — **SCORE**

Instructions to examiner: Consider all types of altered level of cognitive function including cognitive slowing, impaired reasoning, memory loss, deficits in attention and orientation. Rate their impact on activities of daily living as perceived by the patient and/or caregiver.

Instructions to patients [and caregiver]: Over the past week have you had problems remembering things, following conversations, paying attention, thinking clearly, or finding your way around the house or in town? [If yes, examiner asks patient or caregiver to elaborate and probes for information]

0: Normal: No cognitive impairment.

1: Slight: Impairment appreciated by patient or caregiver with no concrete interference with the patient's ability to carry out normal activities and social interactions.

2: Mild: Clinically evident cognitive dysfunction, but only minimal interference with the patient's ability to carry out normal activities and social interactions.

3: Moderate: Cognitive deficits interfere with but do not preclude the patient's ability to carry out normal activities and social interactions.

4: Severe: Cognitive dysfunction precludes the patient's ability to carry out normal activities and social interactions.

☐

July 1, 2008 Copyright © 2008 Movement Disorder Society. All rights reserved. Page 3

This chart may not be copied, distributed or otherwise used in whole or in part without prior written consent of the Movement Disorder Society.

	SCORE
**1.2 HALLUCINATIONS AND PSYCHOSIS**<br><br><u>Instructions to examiner:</u> Consider both illusions (misinterpretations of real stimuli) and hallucinations (spontaneous false sensations). Consider all major sensory domains (visual, auditory, tactile, olfactory and gustatory). Determine presence of unformed (for example sense of presence or fleeting false impressions) as well as formed (fully developed and detailed) sensations. Rate the patients insight into hallucinations and identify delusions and psychotic thinking.<br><br>*<u>Instructions to patients [and caregiver]:</u> Over the past week have you seen, heard, smelled or felt things that were not really there?* [If yes, examiner asks patient or caregiver to elaborate and probes for information]<br><br>0: Normal: No hallucinations or psychotic behaviour.<br>1: Slight: Illusions or non-formed hallucinations, but patient recognizes them without loss of insight.<br>2: Mild: Formed hallucinations independent of environmental stimuli. No loss of insight.<br>3: Moderate: Formed hallucinations with loss of insight.<br>4: Severe: Patient has delusions or paranoia.	☐
**1.3 DEPRESSED MOOD**<br><br><u>Instructions to examiner</u>: Consider low mood, sadness, hopelessness, feelings of emptiness or loss of enjoyment. Determine their presence and duration over the past week and rate their interference with the patient's ability to carry out daily routines and engage in social interactions.<br><br>*<u>Instruction to the patient (and caregiver)</u>: Over the past week have you felt low, sad, hopeless or unable to enjoy things? If yes, was this feeling for longer than one day at a time? Did it make it difficult for you carry out your usual activities or to be with people?* If yes, examiner asks patient or caregiver to elaborate and probes for information]<br><br>0: Normal: No depressed mood.<br>1: Slight: Episodes of depressed mood that are not sustained for more than one day at a time. No interference with patient's ability to carry out normal activities and social interactions.<br>2: Mild: Depressed mood that is sustained over days, but without interference with normal activities and social interactions.<br>3: Moderate: Depressed mood that interferes with, but does not preclude, the patient's ability to carry out normal activities and social interactions.<br>4: Severe: Depressed mood precludes patient's ability to carry out normal activities and social interactions.	☐

Copyright © 2008 Movement Disorder Society. All rights reserved.

This chart may not be copied, distributed or otherwise used in whole or in part without prior written consent of the Movement Disorder Society.

	SCORE
**1.4 ANXIOUS MOOD**<br><br>Instructions to examiner: Determine nervous, tense, worried or anxious feelings (including panic attacks) over the past week and rate their duration and interference with the patient's ability to carry out daily routines and engage in social interactions.<br><br>*Instructions to patients [and caregiver]: Over the past week have you felt nervous, worried or tense? If yes, was this feeling for longer than one day at a time? Did it make it difficult for you to follow your usual activities or to be with other people*? [If yes, examiner asks patient or caregiver to elaborate and probes for information.]<br><br>0: Normal: No anxious feelings.<br><br>1: Slight: Anxious feelings present but not sustained for more than one day at a time. No interference with patient's ability to carry out normal activities and social interactions.<br><br>2: Mild: Anxious feelings are sustained over more than one day at a time, but without interference with patient's ability to carry out normal activities and social interactions.<br><br>3: Moderate: Anxious feelings interfere with, but do not preclude, the patient's ability to carry out normal activities and social interactions.<br><br>4: Severe: Anxious feelings preclude patient's ability to carry out normal activities and social interactions.	☐
**1.5 APATHY**<br><br>Instructions to examiner: Consider level of spontaneous activity, assertiveness, motivation and initiative and rate the impact of reduced levels on performance of daily routines and social interactions. Here the examiner should attempt to distinguish between apathy and similar symptoms that are best explained by depression.<br><br>*Instructions to patients (and caregiver): Over the past week, have you felt indifferent to doing activities or being with people?* If yes, examiner asks patient or caregiver to elaborate and probes for information.]<br><br>0: Normal: No apathy.<br><br>1: Slight: Apathy appreciated by patient and/or caregiver, but no interference with daily activities and social interactions.<br><br>2: Mild: Apathy interferes with isolated activities and social interactions.<br><br>3: Moderate: Apathy interferes with most activities and social interactions.<br><br>4: Severe: Passive and withdrawn, complete loss of initiative.	☐

July 1, 2008 *Copyright © 2008 Movement Disorder Society. All rights reserved.* Page 5
This chart may not be copied, distributed or otherwise used in whole or in part without prior written consent of the Movement Disorder Society.

SCORE

1.6 FEATURES OF DOPAMINE DYSREGULATION SYNDROME

Instructions to examiner: Consider involvement in a variety of activities including atypical or excessive gambling (e.g. casinos or lottery tickets), atypical or excessive sexual drive or interests (e.g., unusual interest in pornography, masturbation, sexual demands on partner), other repetitive activities (e.g. hobbies, dismantling objects, sorting or organizing), or taking extra non-prescribed medication for non-physical reasons (i.e., addictive behavior). Rate the impact of such abnormal activities/behaviors on the patient's personal life and on his family and social relations (including need to borrow money or other financial difficulties like withdrawal of credit cards, major family conflicts, lost time from work, or missed meals or sleep because of the activity).

Instructions to patients [and caregiver]: Over the past week, have you had unusually strong urges that are hard to control? Do you feel driven to do or think about something and find it hard to stop? [Give patient examples such as gambling, cleaning, using the computer, taking extra medicine, obsessing about food or sex, all depending on the patients.

0: Normal: No problems present.

1: Slight: Problems are present but usually do not cause any difficulties for the patient or family/caregiver.

2: Mild: Problems are present and usually cause a few difficulties in the patient's personal and family life.

3: Moderate: Problems are present and usually cause a lot of difficulties in the patient's personal and family life.

4: Severe: Problems are present and preclude the patient's ability to carry out normal activities or social interactions or to maintain previous standards in personal and family life.

☐

The remaining questions in Part I (Non-motor Experiences of Daily Living) [Sleep, Daytime Sleepiness, Pain and Other Sensation, Urinary Problems, Constipation Problems, Lightheadedness on Standing, and Fatigue] are in the **Patient Questionnaire** along with all questions in Part II [Motor Experiences of Daily Living].

 Copyright © 2008 Movement Disorder Society. All rights reserved.
This chart may not be copied, distributed or otherwise used in whole or in part without prior written consent of the Movement Disorder Society.

Patient Questionnaire:

Instructions:

This questionnaire will ask you about your experiences of daily living.

There are 20 questions. We are trying to be thorough, and some of these questions may therefore not apply to you now or ever. If you do not have the problem, simply mark 0 for NO.

Please read each one carefully and read all answers before selecting the one that best applies to you.

We are interested in your average or usual function over the past week including today. Some patients can do things better at one time of the day than at others. However, only one answer is allowed for each question, so please mark the answer that best describes what you can do most of the time.

You may have other medical conditions besides Parkinson's disease. Do not worry about separating Parkinson's disease from other conditions. Just answer the question with your best response.

Use only 0, 1, 2, 3, 4 for answers, nothing else. Do not leave any blanks.

Your doctor or nurse can review the questions with you, but this questionnaire is for patients to complete, either alone or with their caregivers.

Who is filling out this questionnaire (check the best answer):

☐ Patient ☐ Caregiver ☐ Patient and Caregiver in Equal Proportion

Copyright © 2008 Movement Disorder Society. All rights reserved.

This chart may not be copied, distributed or otherwise used in whole or in part without prior written consent of the Movement Disorder Society.

Part I: Non-Motor Aspects of Experiences of Daily Living (nM-EDL)

	SCORE
**1.7 SLEEP PROBLEMS**<br><br>Over the past week, have you had trouble going to sleep at night or staying asleep through the night? Consider how rested you felt after waking up in the morning.<br><br>0: Normal: No problems.<br>1: Slight: Sleep problems are present but usually do not cause trouble getting a full night of sleep.<br>2: Mild: Sleep problems usually cause some difficulties getting a full night of sleep.<br>3: Moderate: Sleep problems cause a lot of difficulties getting a full night of sleep, but I still usually sleep for more than half the night.<br>4: Severe: I usually do not sleep for most of the night.	☐
**1.8 DAYTIME SLEEPINESS**<br><br>Over the past week, have you had trouble staying awake during the daytime?<br><br>0: Normal: No daytime sleepiness.<br>1: Slight: Daytime sleepiness occurs but I can resist and I stay awake.<br>2: Mild: Sometimes I fall asleep when alone and relaxing. For example, while reading or watching TV.<br>3: Moderate: I sometimes fall asleep when I should not. For example, while eating or talking with other people.<br>4: Severe: I often fall asleep when I should not. For example, while eating or talking with other people.	☐

Copyright © 2008 Movement Disorder Society. All rights reserved.
This chart may not be copied, distributed or otherwise used in whole or in part without prior written consent of the Movement Disorder Society.

SCORE

1.9 PAIN AND OTHER SENSATIONS

Over the past week, have you had uncomfortable feelings in your body like pain, aches tingling or cramps?

0: Normal: No uncomfortable feelings.

1: Slight: I have these feelings. However, I can do things and be with other people without difficulty.

2: Mild: These feelings cause some problems when I do things or am with other people.

3: Moderate: These feelings cause a lot of problems, but they do not stop me from doing things or being with other people.

4: Severe: These feelings stop me from doing things or being with other people.

☐

1.10 URINARY PROBLEMS

Over the past week, have you had trouble with urine control? For example, an urgent need to urinate, a need to urinate too often, or urine accidents?

0: Normal: No urine control problems.

1: Slight: I need to urinate often or urgently. However, these problems do not cause difficulties with my daily activities.

2: Mild: Urine problems cause some difficulties with my daily activities. However, I do not have urine accidents.

3: Moderate: Urine problems cause a lot of difficulties with my daily activities, including urine accidents.

4: Severe: I cannot control my urine and use a protective garment or have a bladder tube.

☐

Copyright © 2008 Movement Disorder Society. All rights reserved.
This chart may not be copied, distributed or otherwise used in whole or in part without prior written consent of the Movement Disorder Society.

SCORE

1.11 CONSTIPATION PROBLEMS

Over the past week have you had constipation troubles that cause you difficulty moving your bowels?

0: Normal: No constipation.

1: Slight: I have been constipated. I use extra effort to move my bowels. However, this problem does not disturb my activities or my being comfortable.

2: Mild: Constipation causes me to have some troubles doing things or being comfortable.

3: Moderate: Constipation causes me to have a lot of trouble doing things or being comfortable. However, it does not stop me from doing anything.

4: Severe: I usually need physical help from someone else to empty my bowels.

☐

1.12 LIGHT HEADEDNESS ON STANDING

Over the past week, have you felt faint, dizzy or foggy when you stand up after sitting or lying down?

0: Normal: No dizzy or foggy feelings.

1: Slight: Dizzy or foggy feelings occur. However, they do not cause me troubles doing things.

2: Mild: Dizzy or foggy feelings cause me to hold on to something, but I do not need to sit or lie back down.

3: Moderate: Dizzy or foggy feelings cause me to sit or lie down to avoid fainting or falling.

4: Severe: Dizzy or foggy feelings cause me to fall or faint.

☐

Copyright © 2008 Movement Disorder Society. All rights reserved.

This chart may not be copied, distributed or otherwise used in whole or in part without prior written consent of the Movement Disorder Society.

SCORE

1.13 FATIGUE

Over the past week, have you usually felt fatigued? This feeling is not part of being sleepy or sad

0: Normal: No fatigue.

1: Slight: Fatigue occurs. However it does not cause me troubles doing things or being with people.

2: Mild: Fatigue causes me some troubles doing things or being with people.

3: Moderate: Fatigue causes me a lot of troubles doing things or being with people. However, it does not stop me from doing anything.

4: Severe: Fatigue stops me from doing things or being with people.

☐

Part II: Motor Aspects of Experiences of Daily Living (M-EDL)

2.1 SPEECH

Over the past week, have you had problems with your speech?

0: Normal: Not at all (no problems).

1: Slight: My speech is soft, slurred or uneven, but it does not cause others to ask me to repeat myself.

2: Mild: My speech causes people to ask me to occasionally repeat myself, but not everyday.

3: Moderate: My speech is unclear enough that others ask me to repeat myself every day even though most of my speech is understood.

4: Severe: Most or all of my speech cannot be understood.

☐

Copyright © 2008 Movement Disorder Society. All rights reserved.

This chart may not be copied, distributed or otherwise used in whole or in part without prior written consent of the Movement Disorder Society.

SCORE

2.2 SALIVA & DROOLING

Over the past week, have you usually had too much saliva during when you are awake or when you sleep?

0: Normal: Not at all (no problems).

1: Slight: I have too much saliva, but do not drool.

2: Mild: I have some drooling during sleep, but none when I am awake.

3: Moderate: I have some drooling when I am awake, but I usually do not need tissues or a handkerchief.

4: Severe: I have so much drooling that I regularly need to use tissues or a handkerchief to protect my clothes.

☐

2.3 CHEWING AND SWALLOWING

Over the past week, have you usually had problems swallowing pills or eating meals? Do you need your pills cut or crushed or your meals to be made soft, chopped or blended to avoid choking?

0: Normal: No problems.

1: Slight: I am aware of slowness in my chewing or increased effort at swallowing, but I do not choke or need to have my food specially prepared.

2: Mild: I need to have my pills cut or my food specially prepared because of chewing or swallowing problems, but I have not choked over the past week.

3: Moderate. I choked at least once in the past week.

4: Severe: Because of chewing and swallowing problems, I need a feeding tube.

☐

Copyright © 2008 Movement Disorder Society. All rights reserved.
This chart may not be copied, distributed or otherwise used in whole or in part without prior written consent of the Movement Disorder Society.

SCORE

2.4 EATING TASKS

Over the past week, have you usually had troubles handling your food and using eating utensils? For example, do you have trouble handling finger foods or using forks, knifes, spoons, chopsticks?

0:	Normal:	Not at all (No problems).
1:	Slight:	I am slow, but I do not need any help handling my food and have not had food spills while eating.
2:	Mild:	I am slow with my eating and have occasional food spills. I may need help with a few tasks such as cutting meat.
3:	Moderate:	I need help with many eating tasks but can manage some alone.
4:	Severe:	I need help for most or all eating tasks.

☐

2.5 DRESSING

Over the past week, have you usually had problems dressing? For example, are you slow or do you need help with buttoning, using zippers, putting on or taking off your clothes or jewelry?

0:	Normal:	Not at all (no problems).
1:	Slight:	I am slow but I do not need help.
2:	Mild:	I am slow and need help for a few dressing tasks (buttons, bracelets).
3:	Moderate:	I need help for many dressing tasks.
4:	Severe:	I need help for most or all dressing tasks.

☐

Copyright © 2008 Movement Disorder Society. All rights reserved.
This chart may not be copied, distributed or otherwise used in whole or in part without prior written consent of the Movement Disorder Society.

	SCORE
**2.6 HYGIENE**<br><br>Over the past week, have you usually been slow or do you need help with washing, bathing, shaving, brushing teeth, combing your hair or with other personal hygiene?<br><br>0: Normal: Not at all (no problems).<br>1: Slight: I am slow but I do not need any help.<br>2: Mild: I need someone else to help me with some hygiene tasks.<br>3: Moderate: I need help for many hygiene tasks.<br>4: Severe: I need help for most or all of my hygiene tasks.	☐
**2.7 HANDWRITING**<br><br>Over the past week, have people usually had trouble reading your handwriting?<br><br>0: Normal: Not at all (no problems).<br>1: Slight: My writing is slow, clumsy or uneven, but all words are clear.<br>2: Mild: Some words are unclear and difficult to read.<br>3: Moderate: Many words are unclear and difficult to read.<br>4: Severe: Most or all words cannot be read.	☐
**2.8 DOING HOBBIES AND OTHER ACTIVITIES**<br><br>Over the past week, have you usually had trouble doing your hobbies or other things that you like to do?<br><br>0: Normal: Not at all (no problems).<br>1: Slight: I am a bit slow but do these activities easily.<br>2: Mild: I have some difficulty doing these activities.<br>3: Moderate: I have major problems doing these activities, but still do most.<br>4: Severe: I am unable to do most or all of these activities.	☐

Copyright © 2008 Movement Disorder Society. All rights reserved.

This chart may not be copied, distributed or otherwise used in whole or in part without prior written consent of the Movement Disorder Society.

	SCORE
**2.9 TURNING IN BED**<br><br>Over the past week, do you usually have trouble turning over in bed?<br><br>0: Normal: Not at all (no problems).<br>1: Slight: I have a bit of trouble turning, but I do not need any help.<br>2: Mild I have a lot of trouble turning and need occasional help from someone else.<br>3: Moderate: To turn over I often need help from someone else.<br>4: Severe: I am unable to turn over without help from someone else.	
**2.10 TREMOR**<br><br>Over the past week, have you usually had shaking or tremor?<br><br>0: Normal: Not at all. I have no shaking or tremor.<br>1: Slight: Shaking or tremor occurs but does not cause problems with any activities.<br>2: Mild: Shaking or tremor causes problems with only a few activities.<br>3: Moderate: Shaking or tremor causes problems with many of my daily activities.<br>4: Severe: Shaking or tremor causes problems with most or all activities.	☐
**2.11 GETTING OUT OF BED, A CAR, OR A DEEP CHAIR**<br><br>Over the past week, have you usually had trouble getting out of bed, a car seat, or a deep chair?<br><br>0: Normal: Not at all (no problems).<br>1: Slight: I am slow or awkward, but I usually can do it on my first try.<br>2: Mild: I need more than one try to get up or need occasional help.<br>3: Moderate: I sometimes need help to get up, but most times I can still do it on my own.<br>4: Severe: I need help most or all of the time.	☐

Copyright © 2008 Movement Disorder Society. All rights reserved.

This chart may not be copied, distributed or otherwise used in whole or in part without prior written consent of the Movement Disorder Society.

	SCORE
**2.12 WALKING AND BALANCE**<br><br>Over the past week, have you usually had problems with balance and walking?<br><br>0: Normal: Not at all (no problems).<br><br>1: Slight: I am slightly slow or may drag a leg. I never use a walking aid.<br><br>2: Mild: I occasionally use a walking aid, but I do not need any help from another person.<br><br>3: Moderate: I usually use a walking aid (cane, walker) to walk safely without falling. However, I do not usually need the support of another person.<br><br>4: Severe: I usually use the support of another persons to walk safely without falling.	☐
**2.13 FREEZING**<br><br>Over the past week, on your usual day when walking, do you suddenly stop or freeze as if your feet are stuck to the floor.<br><br>0: Normal: Not at all (no problems).<br><br>1: Slight: I briefly freeze but I can easily start walking again. I do not need help from someone else or a walking aid (cane or walker) because of freezing.<br><br>2: Mild: I freeze and have trouble starting to walk again, but I do not need someone's help or a walking aid (cane or walker) because of freezing.<br><br>3: Moderate: When I freeze I have a lot of trouble starting to walk again and, because of freezing, I sometimes need to use a walking aid or need someone else's help.<br><br>4: Severe: Because of freezing, most or all of the time, I need to use a walking aid or someone's help.	☐

This completes the questionnaire. We may have asked about problems you do not even have, and may have mentioned problems that you may never develop at all. Not all patients develop all these problems, but because they can occur, it is important to ask all the questions to every patient. Thank you for your time and attention in completing this questionnaire.

Copyright © 2008 Movement Disorder Society. All rights reserved.
This chart may not be copied, distributed or otherwise used in whole or in part without prior written consent of the Movement Disorder Society.

Part III: Motor Examination

Overview: This portion of the scale assesses the motor signs of PD. In administering Part III of the MDS-UPDRS the examiner should comply with the following guidelines:

At the top of the form, mark whether the patient is on medication for treating the symptoms of Parkinson's disease and, if on levodopa, the time since the last dose.

Also, if the patient is receiving medication for treating the symptoms of Parkinson's Disease, mark the patient's clinical state using the following definitions:

ON is the typical functional state when patients are receiving medication and have a good response.
OFF is the typical functional state when patients have a poor response in spite of taking medications.

The investigator should "rate what you see". Admittedly, concurrent medical problems such as stroke, paralysis, arthritis, contracture, and orthopedic problems such as hip or knee replacement and scoliosis may interfere with individual items in the motor examination. In situations where it is absolutely impossible to test (e.g., amputations, plegia, limb in a cast), use the notation "**UR**" for Unable to Rate. Otherwise, rate the performance of each task as the patient performs in the context of co-morbidities.

All items must have an integer rating (no half points, no missing ratings).

Specific instructions are provided for the testing of each item. These should be followed in all instances. The investigator demonstrates while describing tasks the patient is to perform and rates function immediately thereafter. For Global Spontaneous Movement and Rest Tremor items (3.14 and 3.17), these items have been placed purposefully at the end of the scale because clinical information pertinent to the score will be obtained throughout the entire examination.

At the end of the rating, indicate if dyskinesia (chorea or dystonia) was present at the time of the examination, and if so, whether these movements interfered with the motor examination.

3a Is the patient on medication for treating the symptoms of Parkinson's Disease? ☐ No ☐ Yes

3b If the patient is receiving medication for treating the symptoms of Parkinson's Disease, mark the patient's clinical state using the following definitions:

☐ ON: On is the typical functional state when patients are receiving medication and have a good response.

☐ OFF: Off is the typical functional state when patients have a poor response in spite of taking medications.

3c Is the patient on Levodopa ? ☐ No ☐ Yes

3.C1 If yes, minutes since last levodopa dose: __________

Copyright © 2008 Movement Disorder Society. All rights reserved.

This chart may not be copied, distributed or otherwise used in whole or in part without prior written consent of the Movement Disorder Society.

	SCORE
**3.1 SPEECH**<br><br>Instructions to examiner: Listen to the patient's free-flowing speech and engage in conversation if necessary. Suggested topics: ask about the patient's work, hobbies, exercise, or how he got to the doctor's office. Evaluate volume, modulation (prosody) and clarity, including slurring, palilalia (repetition of syllables) and tachyphemia (rapid speech, running syllables together).<br><br>0: Normal: No speech problems.<br><br>1: Slight: Loss of modulation, diction or volume, but still all words easy to understand.<br><br>2: Mild: Loss of modulation, diction, or volume, with a few words unclear, but the overall sentences easy to follow.<br><br>3: Moderate: Speech is difficult to understand to the point that some, but not most, sentences are poorly understood.<br><br>4: Severe: Most speech is difficult to understand or unintelligible.	☐
**3.2 FACIAL EXPRESSION**<br><br>Instructions to examiner: Observe the patient sitting at rest for 10 seconds, without talking and also while talking. Observe eye-blink frequency, masked facies or loss of facial expression, spontaneous smiling and parting of lips.<br><br>0: Normal: Normal facial expression.<br><br>1: Slight: Minimal masked facies manifested only by decreased frequency of blinking.<br><br>2: Mild: In addition to decreased eye-blink frequency, Masked facies present in the lower face as well, namely fewer movements around the mouth, such as less spontaneous smiling, but lips not parted.<br><br>3: Moderate: Masked facies with lips parted some of the time when the mouth is at rest.<br><br>4: Severe: Masked facies with lips parted most of the time when the mouth is at rest.	☐

Copyright © 2008 Movement Disorder Society. All rights reserved.

This chart may not be copied, distributed or otherwise used in whole or in part without prior written consent of the Movement Disorder Society.

	SCORE
**3.3 RIGIDITY**<br><br><u>Instructions to examiner</u>: Rigidity is judged on slow passive movement of major joints with the patient in a relaxed position and the examiner manipulating the limbs and neck. First, test without an activation maneuver. Test and rate neck and each limb separately. For arms, test the wrist and elbow joints simultaneously. For legs, test the hip and knee joints simultaneously. If no rigidity is detected, use an activation maneuver such as tapping fingers, fist opening/closing, or heel tapping in a limb not being tested. Explain to the patient to go as limp as possible as you test for rigidity.<br><br>0: Normal: No rigidity.<br><br>1: Slight: Rigidity only detected with activation maneuver.<br><br>2: Mild: Rigidity detected without the activation maneuver, but full range of motion is easily achieved.<br><br>3: Moderate: Rigidity detected without the activation maneuver; full range of motion is achieved with effort.<br><br>4: Severe: Rigidity detected without the activation maneuver and full range of motion not achieved.	☐ Neck<br><br>☐ RUE<br><br>☐ LUE<br><br>☐ RLE<br><br>☐ LLE
**3.4 FINGER TAPPING**<br><br><u>Instructions to examiner</u>: Each hand is tested separately. Demonstrate the task, but do not continue to perform the task while the patient is being tested. Instruct the patient to tap the index finger on the thumb 10 times as quickly AND as big as possible. Rate each side separately, evaluating speed, amplitude, hesitations, halts and decrementing amplitude.<br><br>0: Normal: No problems.<br><br>1: Slight: Any of the following: a) the regular rhythm is broken with one or two interruptions or hesitations of the tapping movement; b) slight slowing; c) the amplitude decrements near the end of the 10 taps.<br><br>2: Mild: Any of the following: a) 3 to 5 interruptions during tapping; b) mild slowing; c) the amplitude decrements midway in the 10-tap sequence.<br><br>3: Moderate: Any of the following: a) more than 5 interruptions during tapping or at least one longer arrest (freeze) in ongoing movement; b) moderate slowing; c) the amplitude decrements starting after the 1st tap.<br><br>4: Severe: Cannot or can only barely perform the task because of slowing, interruptions or decrements.	☐ R<br><br>☐ L

Copyright © 2008 Movement Disorder Society. All rights reserved.

This chart may not be copied, distributed or otherwise used in whole or in part without prior written consent of the Movement Disorder Society.

	SCORE
**3.5 HAND MOVEMENTS**<br><br><u>Instructions to examiner</u>: Test each hand separately. Demonstrate the task, but do not continue to perform the task while the patient is being tested. Instruct the patient to make a tight fist with the arm bent at the elbow so that the palm faces the examiner. Have the patient open the hand 10 times as fully AND as quickly as possible. If the patient fails to make a tight fist or to open the hand fully, remind him/her to do so. Rate each side separately, evaluating speed, amplitude, hesitations, halts and decrementing amplitude.<br><br>0: Normal: No problem.<br>1: Slight: Any of the following: a) the regular rhythm is broken with one or two interruptions or hesitations of the movement; b) slight slowing; c) the amplitude decrements near the end of the task.<br>2: Mild: Any of the following: a) 3 to 5 interruptions during the movements; b) mild slowing; c) the amplitude decrements midway in the task.<br>3: Moderate: Any of the following: a) more than 5 interruptions during the movement or at least one longer arrest (freeze) in ongoing movement; b) moderate slowing; c) the amplitude decrements starting after the 1st open-and-close sequence.<br>4: Severe: Cannot or can only barely perform the task because of slowing, interruptions or decrements.	☐ R<br><br>☐ L
**3.6 PRONATION-SUPINATION MOVEMENTS OF HANDS**<br><br><u>Instructions to examiner</u>: Test each hand separately. Demonstrate the task, but do not continue to perform the task while the patient is being tested. Instruct the patient to extend the arm out in front of his/her body with the palms down; then to turn the palm up and down alternately 10 times as fast and as fully as possible. Rate each side separately, evaluating speed, amplitude, hesitations, halts and decrementing amplitude.<br><br>0: Normal: No problems.<br>1: Slight: Any of the following: a) the regular rhythm is broken with one or two interruptions or hesitations of the movement; b) slight slowing; c) the amplitude decrements near the end of the sequence.<br>2: Mild: Any of the following: a) 3 to 5 interruptions during the movements; b) mild slowing; c) the amplitude decrements midway in the sequence.<br>3: Moderate: Any of the following: a) more than 5 interruptions during the movement or at least one longer arrest (freeze) in ongoing movement; b) moderate slowing c) the amplitude decrements starting after the 1st supination-pronation sequence.<br>4: Severe: Cannot or can only barely perform the task because of slowing, interruptions or decrements.	☐ R<br><br>☐ L

Copyright © 2008 Movement Disorder Society. All rights reserved.
This chart may not be copied, distributed or otherwise used in whole or in part without prior written consent of the Movement Disorder Society.

	SCORE
**3.7 TOE TAPPING**<br><br><u>Instructions to examiner</u>: Have the patient sit in a straight-backed chair with arms, both feet on the floor. Test each foot separately. Demonstrate the task, but do not continue to perform the task while the patient is being tested. Instruct the patient to place the heel on the ground in a comfortable position and then tap the toes 10 times as big and as fast as possible. Rate each side separately, evaluating speed, amplitude, hesitations, halts and decrementing amplitude.<br><br>0: Normal: No problem.<br><br>1: Slight: Any of the following: a) the regular rhythm is broken with one or two interruptions or hesitations of the tapping movement; b) slight slowing; c) amplitude decrements near the end of the ten taps.<br><br>2: Mild: Any of the following: a) 3 to 5 interruptions during the tapping movements; b) mild slowing; c) amplitude decrements midway in the task.<br><br>3: Moderate: Any of the following: a) more than 5 interruptions during the tapping movements or at least one longer arrest (freeze) in ongoing movement; b) moderate slowing; c) amplitude decrements after the first tap.<br><br>4: Severe: Cannot or can only barely perform the task because of slowing, interruptions or decrements.	☐ R<br><br>☐ L
**3.8 LEG AGILITY**<br><br><u>Instructions to examiner</u>: Have the patient sit in a straight-backed chair with arms. The patient should have both feet comfortably on the floor. Test each leg separately. Demonstrate the task, but do not continue to perform the task while the patient is being tested. Instruct the patient to place the foot on the ground in a comfortable position and then raise and stomp the foot on the ground 10 times as high and as fast as possible. Rate each side separately, evaluating speed, amplitude, hesitations, halts and decrementing amplitude.<br><br>0: Normal: No problems.<br><br>1: Slight: Any of the following: a) the regular rhythm is broken with one or two interruptions or hesitations of the movement; b) slight slowing; c) amplitude decrements near the end of the task.<br><br>2: Mild: Any of the following: a) 3 to 5 interruptions during the movements; b) mild slowness; c) amplitude decrements midway in the task.<br><br>3: Moderate: Any of the following: a) more than 5 interruptions during the movement or at least one longer arrest (freeze) in ongoing movement; b) moderate slowing in speed; c) amplitude decrements after the first tap.<br><br>4: Severe: Cannot or can only barely perform the task because of slowing, interruptions or decrements.	☐ R<br><br>☐ L

Copyright © 2008 Movement Disorder Society. All rights reserved.

This chart may not be copied, distributed or otherwise used in whole or in part without prior written consent of the Movement Disorder Society.

SCORE

3.9 ARISING FROM CHAIR

Instructions to examiner: Have the patient sit in a straight-backed chair with arms, with both feet on the floor and sitting back in the chair (if the patient is not too short). Ask the patient to cross his/her arms across the chest and then to stand up. If the patient is not successful, repeat this attempt a maximum up to two more times. If still unsuccessful, allow the patient to move forward in the chair to arise with arms folded across the chest. Allow only one attempt in this situation. If unsuccessful, allow the patient to push off using his/her hands on the arms of the chair. Allow a maximum of three trials of pushing off. If still not successful, assist the patient to arise. After the patient stands up, observe the posture for item 3.13

0: Normal:	No problems. Able to arise quickly without hesitation.
1: Slight:	Arising is slower than normal; or may need more than one attempt; or may need to move forward in the chair to arise. No need to use the arms of the chair.
2: Mild:	Pushes self up from arms of chair without difficulty.
3: Moderate:	Needs to push off, but tends to fall back; or may have to try more than one time using arms of chair, but can get up without help.
4: Severe:	Unable to arise without help.

☐

3.10 GAIT

Instructions to examiner: Testing gait is best performed by having the patient walking away from and towards the examiner so that both right and left sides of the body can be easily observed simultaneously. The patient should walk at least 10 meters (30 feet), then turn around and return to the examiner. This item measures multiple behaviors: stride amplitude, stride speed, height of foot lift, heel strike during walking, turning, and arm swing, but not freezing. Assess also for "freezing of gait" (next item 3.11) while patient is walking. Observe posture for item 3.13

0: Normal:	No problems.
1: Slight:	Independent walking with minor gait impairment.
2: Mild:	Independent walking but with substantial gait impairment.
3: Moderate:	Requires an assistance device for safe walking (walking stick, walker) but not a person.
4: Severe:	Cannot walk at all or only with another person's assistance.

☐

Copyright © 2008 Movement Disorder Society. All rights reserved.

This chart may not be copied, distributed or otherwise used in whole or in part without prior written consent of the Movement Disorder Society.

SCORE

3.11 FREEZING OF GAIT

Instructions to examiner: While assessing gait, also assess for the presence of any gait freezing episodes. Observe for start hesitation and stuttering movements especially when turning and reaching the end of the task. To the extent that safety permits, patients may NOT use sensory tricks during the assessment.

0: Normal: No freezing.

1: Slight: Freezes on starting, turning or walking through doorway with a single halt during any of these events, but then continues smoothly without freezing during straight walking.

2: Mild: Freezes on starting, turning or walking through doorway with more than one halt during any of these activities, but continues smoothly without freezing during straight walking.

3: Moderate: Freezes once during straight walking.

4: Severe: Freezes multiple times during straight walking.

☐

3.12 POSTURAL STABILITY

Instructions to examiner: The test examines the response to sudden body displacement produced by a quick, forceful pull on the shoulders while the patient is standing erect with eyes open and feet comfortably apart and parallel to each other. Test retropulsion. Stand behind the patient and instruct the patient on what is about to happen. Explain that s/he is allowed to take a step backwards to avoid falling. There should be a solid wall behind the examiner, at least 1-2 meters away to allow for the observation of the number of retropulsive steps. The first pull is an instructional demonstration and is purposely milder and not rated. The second time the shoulders are pulled briskly and forcefully towards the examiner with enough force to displace the center of gravity so that patient MUST take a step backwards. The examiner needs to be ready to catch the patient, but must stand sufficiently back so as to allow enough room for the patient to take several steps to recover independently. Do not allow the patient to flex the body abnormally forward in anticipation of the pull. Observe for the number of steps backwards or falling. Up to and including two steps for recovery is considered normal, so abnormal ratings begin with three steps. If the patient fails to understand the test, the examiner can repeat the test so that the rating is based on an assessment that the examiner feels reflects the patient's limitations rather than misunderstanding or lack of preparedness. Observe standing posture for item 3.13

0: Normal: No problems: Recovers with one or two steps.

1: Slight: 3-5 steps, but subject recovers unaided.

2: Mild: More than 5 steps, but subject recovers unaided.

3: Moderate: Stands safely, but with absence of postural response; falls if not caught by examiner.

4: Severe: Very unstable, tends to lose balance spontaneously or with just a gentle pull on the shoulders.

☐

Copyright © 2008 Movement Disorder Society. All rights reserved.

This chart may not be copied, distributed or otherwise used in whole or in part without prior written consent of the Movement Disorder Society.

	SCORE
**3.13 POSTURE**<br><br>Instructions to examiner: Posture is assessed with the patient standing erect after arising from a chair, during walking , and while being tested for postural reflexes. If you notice poor posture, tell the patient to stand up straight and see if the posture improves (see option 2 below). Rate the worst posture seen in these three observation points. Observe for flexion and side-to-side leaning.<br><br>0: Normal: No problems.<br>1: Slight: Not quite erect, but posture could be normal for older person.<br>2: Mild: Definite flexion, scoliosis or leaning to one side, but patient can correct posture to normal posture when asked to do so.<br>3: Moderate: Stooped posture, scoliosis or leaning to one side that cannot be corrected volitionally to a normal posture by the patient.<br>4: Severe: Flexion, scoliosis or leaning with extreme abnormality of posture.	☐
**3.14 GLOBAL SPONTANEITY OF MOVEMENT (BODY BRADYKINESIA)**<br><br>Instructions to examiner: This global rating combines all observations on slowness, hesitancy, and small amplitude and poverty of movement in general, including a reduction of gesturing and of crossing the legs. This assessment is based on the examiner's global impression after observing for spontaneous gestures while sitting, and the nature of arising and walking.<br><br>0: Normal: No problems.<br>1: Slight: Slight global slowness and poverty of spontaneous movements.<br>2: Mild: Mild global slowness and poverty of spontaneous movements.<br>3: Moderate: Moderate global slowness and poverty of spontaneous movements.<br>4: Severe: Severe global slowness and poverty of spontaneous movements.	☐
**3.15 POSTURAL TREMOR OF THE HANDS**<br><br>Instructions to examiner: All tremor, including re-emergent rest tremor, that is present in this posture is to be included in this rating. Rate each hand separately. Rate the highest amplitude seen. Instruct the patient to stretch the arms out in front of the body with palms down. The wrist should be straight and the fingers comfortably separated so that they do not touch each other. Observe this posture for 10 seconds.<br><br>0: Normal: No tremor.<br>1: Slight: Tremor is present but less than 1 cm in amplitude.<br>2: Mild: Tremor is at least 1 but less than 3 cm in amplitude.<br>3: Moderate: Tremor is at least 3 but less than 10 cm in amplitude.<br>4: Severe: Tremor is at least 10 cm in amplitude.	☐<br>R<br><br>☐<br>L

July 1, 2008 Page 24
Copyright © 2008 Movement Disorder Society. All rights reserved.
This chart may not be copied, distributed or otherwise used in whole or in part without prior written consent of the Movement Disorder Society.

SCORE

3.16 KINETIC TREMOR OF THE HANDS

Instructions to examiner: This is tested by the finger-to-nose maneuver. With the arm starting from the outstretched position, have the patient perform at least three finger-to-nose maneuvers with each hand reaching as far as possible to touch the examiner's finger. The finger-to-nose maneuver should be performed slowly enough not to hide any tremor that could occur with very fast arm movements. Repeat with the other hand, rating each hand separately. The tremor can be present throughout the movement or as the tremor reaches either target (nose or finger). Rate the highest amplitude seen.

0: Normal:	No tremor.
1: Slight:	Tremor is present but less than 1 cm in amplitude.
2: Mild:	Tremor is at least 1 but less than 3 cm in amplitude.
3: Moderate:	Tremor is at least 3 but less than 10 cm in amplitude.
4: Severe:	Tremor is at least 10 cm in amplitude.

☐ R

☐ L

3.17 REST TREMOR AMPLITUDE

Instructions to examiner: This and the next item have been placed purposefully at the end of the examination to allow the rater to gather observations on rest tremor that may appear at any time during the exam, including when quietly sitting, during walking and during activities when some body parts are moving but others are at rest. Score the maximum amplitude that is seen at any time as the final score. Rate only the amplitude and not the persistence or the intermittency of the tremor.
As part of this rating, the patient should sit quietly in a chair with the hands placed on the arms of the chair (not in the lap) and the feet comfortably supported on the floor for 10 seconds with no other directives. Rest tremor is assessed separately for all four limbs and also for the lip/jaw. Rate only the maximum amplitude that is seen at any time as the final rating.

Extremity ratings

0: Normal:	No tremor.
1: Slight.:	< 1 cm in maximal amplitude.
2: Mild:	> 1 cm but < 3 cm in maximal amplitude.
3: Moderate:	3 - 10 cm in maximal amplitude.
4: Severe:	> 10 cm in maximal amplitude.

Lip/Jaw ratings

0: Normal:	No tremor.
1: Slight:	< 1 cm in maximal amplitude.
2: Mild:	> 1 cm but < 2 cm in maximal amplitude.
3: Moderate:	> 2 cm but < 3 cm in maximal amplitude.
4: Severe:	> 3 cm in maximal amplitude.

☐ RUE

☐ LUE

☐ RLE

☐ LLE

☐ Lip/Jaw

July 1, 2008 *Copyright © 2008 Movement Disorder Society. All rights reserved.*
This chart may not be copied, distributed or otherwise used in whole or in part without prior written consent of the Movement Disorder Society.

	SCORE
**3.18 CONSTANCY OF REST TREMOR**<br><br>Instructions to examiner: This item receives one rating for all rest tremor and focuses on the constancy of rest tremor during the examination period when different body parts are variously at rest. It is rated purposefully at the end of the examination so that several minutes of information can be coalesced into the rating.<br><br>0: Normal: No tremor.<br>1: Slight: Tremor at rest is present < 25% of the entire examination period.<br>2: Mild: Tremor at rest is present 26-50% of the entire examination period.<br>3: Moderate: Tremor at rest is present 51-75% of the entire examination period.<br>4: Severe: Tremor at rest is present > 75% of the entire examination period.	☐
**DYSKINESIA IMPACT ON PART III RATINGS**<br><br>A. Were dyskinesias (chorea or dystonia) present during examination? ☐ No ☐ Yes<br><br>B. If yes, did these movements interfere with your ratings? ☐ No ☐ Yes	
**HOEHN AND YAHR STAGE**<br><br>0: Asymptomatic.<br>1: Unilateral involvement only.<br>2: Bilateral involvement without impairment of balance.<br>3: Mile to moderate involvement; some postural instability but physically independent; needs assistance to recover from pull test.<br>4: Severe disability; still able to walk or stand unassisted.<br>5: Wheelchair bound or bedridden unless aided.	☐

July 1, 2008

Copyright © 2008 Movement Disorder Society. All rights reserved.
This chart may not be copied, distributed or otherwise used in whole or in part without prior written consent of the Movement Disorder Society.

Page 26

Part IV: Motor Complications

Overview and Instructions: In this section, the rater uses historical and objective information to assess two motor complications, dyskinesias and motor fluctuations that include OFF-state dystonia. Use all information from patient, caregiver, and the examination to answer the six questions that summarize function over the past week including today. As in the other sections, rate using only integers (no half points allowed) and leave no missing ratings. If the item cannot be rated, place UR for Unable to Rate. You will need to choose some answers based on percentages, and therefore you will need to establish how many hours generally are awake hours and use this figure as the denominator for "OFF" time and Dyskinesias. For "OFF dystonia", the total "Off" time will be the denominator. Operational definitions for examiner's use.

Dyskinesias: Involuntary random movements
Words that patients often recognize for dyskinesias include "irregular jerking", "wiggling", "twitching". It is essential to stress to the patient the difference between dyskinesias and tremor, a common error when patients are assessing dyskinesias.

Dystonia: contorted posture, often with a twisting component:
Words that patients often recognize for dystonia include "spasms", "cramps", "posture".

Motor fluctuation: Variable response to medication:
Words that patients often recognize for motor fluctuation include "wearing out", "wearing off", "roller-coaster effect", "on-off", "uneven medication effects".

OFF: Typical functional state when patients have a poor response in spite of taking mediation or the typical functional response when patients are on NO treatment for parkinsonism. Words that patients often recognize include "low time", "bad time", "shaking time", "slow time", "time when my medications don't work."

ON: Typical functional state when patients are receiving medication and have a good response:
Words that patients often recognize include "good time", "walking time", "time when my medications work."

A . DYSKINESIAS [exclusive of OFF-state dystonia]

4.1 TIME SPENT WITH DYSKINESIAS — SCORE: ☐

Instructions to examiner: Determine the hours in the usual waking day and then the hours of dyskinesias. Calculate the percentage. If the patient has dyskinesias in the office, you can point them out as a reference to ensure that patients and caregivers understand what they are rating. You may also use your own acting skills to enact the dyskinetic movements you have seen in the patient before or show them dyskinetic movements typical of other patients. Exclude from this question early morning and nighttime painful dystonia.

Instructions to patient [and caregiver]. Over the past week, how many hours do you usually sleep on a daily basis, including nighttime sleep and daytime napping? Alright, if you sleep ___ hrs, you are awake ____ hrs. Out of those awake hours, how many hours in total do you have wiggling, twitching or jerking movements? Do not count the times when you have tremor, which is a regular back and forth shaking or times when you have painful foot cramps or spasms in the early morning or at nighttime. I will ask about those later. Concentrate only on these types of wiggling, jerking and irregular movements. Add up all the time during the waking day when these usually occur. How many hours ____ (use this number for your calculation).

0: Normal:	No dyskinesias.	
1: Slight:	≤ 25% of waking day.	
2: Mild:	26 - 50% of waking day.	1. Total Hours Awake: _____
3: Moderate:	51 - 75% of waking day.	2. Total Hours with Dyskinesia: _____
4: Severe:	> 75% of waking day.	3. % Dyskinesia = ((2/1)*100): _____

Copyright © 2008 Movement Disorder Society. All rights reserved.
This chart may not be copied, distributed or otherwise used in whole or in part without prior written consent of the Movement Disorder Society.

4.2 FUNCTIONAL IMPACT OF DYSKINESIAS	SCORE
Instructions to examiner: Determine the degree to which dyskinesias impact on the patient's daily function in terms of activities and social interactions. Use the patient's and caregiver's response to your question and your own observations during the office visit to arrive at the best answer.<br><br>*Instructions to patient [and caregiver]: Over the past week, did you usually have trouble doing things or being with people when these jerking movements occurred? Did they stop you from doing things or from being with people?*<br><br>0: Normal: No dyskinesias or no impact by dyskinesias on activities or social interactions.<br><br>1: Slight: Dyskinesias impact on a few activities, but the patient usually performs all activities and participates in all social interactions during dyskinetic periods.<br><br>2: Mild: Dyskinesias impact on many activities, but the patient usually performs all activities and participates in all social interactions during dyskinetic periods.<br><br>3: Moderate: Dyskinesias impact on activities to the point that the patient usually does not perform some activities or does not usually participate in some social activities during dyskinetic episodes.<br><br>4: Severe: Dyskinesias impact on function to the point that the patient usually does not perform most activities or participate in most social interactions during dyskinetic episodes.	☐
**B . MOTOR FLUCTUATIONS**	
**4.3 TIME SPENT IN THE OFF STATE**<br><br>Instructions to examiner: Use the number of waking hours derived from 4.1 and determine the hours spent in the "OFF" state. Calculate the percentage. If the patient has an OFF period in the office, you can point to this state as a reference. You may also use your knowledge of the patient to describe a typical OFF period. Additionally you may use your own acting skills to enact an OFF period you have seen in the patient before or show them OFF function typical of other patients. Mark down the typical number of OFF hours, because you will need this number for completing 4.6<br><br>*Instructions to patient [and caregiver]: Some patients with Parkinson's disease have a good effect from their medications throughout their awake hours and we call that "ON" time. Other patients take their medications but still have some hours of low time, bad time, slow time or shaking time. Doctors call these low periods "OFF" time. Over the past week, you told me before that you are generally awake ____ hrs each day. Out of these awake hours, how many hours in total do you usually have this type of low level or OFF function ____ (Use this number for your calculations).*<br><br>0: Normal: No OFF time.<br><br>1: Slight: ≤ 25% of waking day.<br><br>2: Mild: 26 - 50% of waking day.<br><br>3: Moderate: 51 - 75% of waking day.<br><br>4: Severe: > 75% of waking day.<br><br>1. Total Hours Awake: _____<br>2. Total Hours OFF: _____<br>3. % OFF = ((2/1)*100): _____	☐

July 1, 2008

Copyright © 2008 Movement Disorder Society. All rights reserved.
This chart may not be copied, distributed or otherwise used in whole or in part without prior written consent of the Movement Disorder Society.

	SCORE
**4.4 FUNCTIONAL IMPACT OF FLUCTUATIONS**<br><br>Instructions to examiner: Determine the degree to which motor fluctuations impact on the patient's daily function in terms of activities and social interactions. This question concentrates on the difference between the ON state and the OFF state. If the patient has no OFF time, the rating must be 0, but if patients have very mild fluctuations, it is still possible to be rated 0 on this item if no impact on activities occurs. Use the patient's and caregiver's response to your question and your own observations during the office visit to arrive at the best answer.<br><br>*Instructions to patient [and caregiver]: Think about when those low or "OFF" periods have occurred over the past week. Do you usually have more problems doing things or being with people than compared to the rest of the day when you feel your medications working? Are there some things you usually do during a good period that you have trouble with or stop doing during a low period?*<br><br>0: Normal: No fluctuations or No impact by fluctuations on performance of activities or social interactions.<br><br>1: Slight: Fluctuations impact on a few activities, but during OFF, the patient usually performs all activities and participates in all social interactions that typically occur during the ON state.<br><br>2: Mild: Fluctuations impact many activities, but during OFF, the patient still usually performs all activities and participates in all social interactions that typically occur during the ON state.<br><br>3: Moderate: Fluctuations impact on the performance of activities during OFF to the point that the patient usually does not perform some activities or participate in some social interactions that are performed during ON periods.<br><br>4: Severe: Fluctuations impact on function to the point that, during OFF, the patient usually does not perform most activities or participate in most social interactions that are performed during ON periods.	☐
**4.5 COMPLEXITY OF MOTOR FLUCTUATIONS**<br><br>Instructions to examiner: Determine the usual predictability of OFF function whether due to dose, time of day, food intake or other factors. Use the information provided by the patients and caregiver and supplement with your own observations. You will ask if the patient can count on them always coming at a special time, mostly coming at a special time (in which case you will probe further to separate slight from mild), only sometimes coming at a special time or are they totally unpredictable? Narrowing down the percentage will allow you to find the correct answer.<br><br>*Instructions to patient [and caregiver]: For some patients, the low or "OFF" periods happen at certain times during day or when they do activities like eating or exercising. Over the past week, do you usually know when your low periods will occur? In other words, do your low periods always come at a certain time? Do they mostly come at a certain time? Do they only sometimes come at a certain time? Are your low periods totally unpredictable?"*<br><br>0: Normal: No motor fluctuations.<br><br>1: Slight: OFF times are predictable all or almost all of the time (> 75%).<br><br>2: Mild: OFF times are predictable most of the time (51-75%).<br><br>3: Moderate: OFF times are predictable some of the time (26-50%).<br><br>4: Severe: OFF episodes are rarely predictable. ($\leq$ 25%).	☐

Copyright © 2008 Movement Disorder Society. All rights reserved.

This chart may not be copied, distributed or otherwise used in whole or in part without prior written consent of the Movement Disorder Society.

C. "OFF" DYSTONIA

4.6 PAINFUL OFF-STATE DYSTONIA

Instructions to examiner: For patients who have motor fluctuations, determine what proportion of the OFF episodes usually includes painful dystonia? You have already determined the number of hours of "OFF" time (4.3). Of these hours, determine how many are associated with dystonia and calculate the percentage. If there is no OFF time, mark 0.

Instructions to patient [and caregiver]: *In one of the questions I asked earlier, you said you generally have ___ hours of low or "OFF" time when your Parkinson's disease is under poor control. During these low or "OFF" periods, do you usually have painful cramps or spasms? Out of the total ____ hrs of this low time, if you add up all the time in a day when these painful cramps come, how many hours would this make?*

0: Normal: No dystonia OR NO OFF TIME.

1: Slight: < 25% of time in OFF state.

2: Mild: 26-50% of time in OFF state.

3: Moderate: 51-75% of time in OFF state.

4: Severe: > 75% of time in OFF state.

☐

1. Total Hours Off: _____
2. Total Off Hours w/Dystonia: _____
3. % Off Dystonia = ((2/1)*100): _____

Summary statement to patient: READ TO PATIENT

This completes my rating of your Parkinson's disease. I know the questions and tasks have taken several minutes, but I wanted to be complete and cover all possibilities. In doing so, I may have asked about problems you do not even have, and I may have mentioned problems that you may never develop at all. Not all patients develop all these problems, but because they can occur, it is important to ask all the questions to every patient. Thank you for your time and attention in completing this scale with me.

July 1, 2008 *Copyright © 2008 Movement Disorder Society. All rights reserved.* Page 30
This chart may not be copied, distributed or otherwise used in whole or in part without prior written consent of the Movement Disorder Society.

______________________	___ ___ ___	___ - ___ - ______ (mm-dd-yyyy)	___ ___ ___
Patient Name or Subject ID	Site ID	Assessment Date	Investigator's Initials

MDS UPDRS Score Sheet

1.A	Source of information	☐ Patient ☐ Caregiver ☐ Patient + Caregiver	3.3b	Rigidity– RUE	
			3.3c	Rigidity– LUE	
**Part I**			3.3d	Rigidity– RLE	
1.1	Cognitive impairment		3.3e	Rigidity– LLE	
1.2	Hallucinations and psychosis		3.4a	Finger tapping– Right hand	
1.3	Depressed mood		3.4b	Finger tapping– Left hand	
1.4	Anxious mood		3.5a	Hand movements– Right hand	
1.5	Apathy		3.5b	Hand movements– Left hand	
1.6	Features of DDS		3.6a	Pronation- supination movements– Right hand	
1.6a	Who is filling out questionnaire	☐ Patient ☐ Caregiver ☐ Patient + Caregiver	3.6b	Pronation- supination movements– Left hand	
			3.7a	Toe tapping–Right foot	
1.7	Sleep problems		3.7b	Toe tapping– Left foot	
1.8	Daytime sleepiness		3.8a	Leg agility– Right leg	
1.9	Pain and other sensations		3.8b	Leg agility– Left leg	
1.10	Urinary problems		3.9	Arising from chair	
1.11	Constipation problems		3.10	Gait	
1.12	Light headedness on standing		3.11	Freezing of gait	
1.13	Fatigue		3.12	Postural stability	
**Part II**			3.13	Posture	
2.1	Speech		3.14	Global spontaneity of movement	
2.2	Saliva and drooling		3.15a	Postural tremor– Right hand	
2.3	Chewing and swallowing		3.15b	Postural tremor– Left hand	
2.4	Eating tasks		3.16a	Kinetic tremor– Right hand	
2.5	Dressing		3.16b	Kinetic tremor– Left hand	
2.6	Hygiene		3.17a	Rest tremor amplitude– RUE	
2.7	Handwriting		3.17b	Rest tremor amplitude– LUE	
2.8	Doing hobbies and other activities		3.17c	Rest tremor amplitude– RLE	
2.9	Turning in bed		3.17d	Rest tremor amplitude– LLE	
2.10	Tremor		3.17e	Rest tremor amplitude– Lip/jaw	
2.11	Getting out of bed		3.18	Constancy of rest	
2.12	Walking and balance			Were dyskinesias presen	☐ No ☐ Yes
2.13	Freezing			Did these movements interfere with ratings?	☐ No ☐ Yes
3a	Is the patient on medication?	☐ No ☐ Yes		Hoehn and Yahr Stage	
3b	Patient's clinical state	☐ Off ☐ On	**Part IV**		
3c	Is the patient on Levodopa?	☐ No ☐ Yes	4.1	Time spent with dyskinesias	
3.C1	If yes, minutes since last dose:		4.2	Functional impact of dyskinesias	
**Part III**			4.3	Time spent in the OFF state	
3.1	Speech		4.4	Functional impact of fluctuations	
3.2	Facial expression		4.5	Complexity of motor fluctuations	
3.3a	Rigidity– Neck		4.6	Painful OFF-state dystonia	

July 1, 2008

Copyright © 2008 Movement Disorder Society. All rights reserved.
This chart may not be copied, distributed or otherwise used in whole or in part without prior written consent of the Movement Disorder Society

APPENDIX F. PATIENT GLOBAL IMPRESSION OF CHANGE (PGI-C)

The subject will independently rate the following question of Patient Global Impression of Change (PGI-C) based on his/her overall impression at Visit 5 (Week 10), Visit 6 (Week 15), and Visit 7 (Week 20) or early discontinuation.

Patient Global Impression of Change:

Compared to your condition prior to your starting on this study, how much has your condition changed with your current treatment?

❑ 1	❑ 2	❑ 3	❑ 4	❑ 5	❑ 6	❑ 7
Very Much Worse	Much Worse	Minimally Worse	No Change	Minimally Improved	Much Improved	Very Much Improved

APPENDIX G. CLINICAL GLOBAL IMPRESSION OF CHANGE (CGI-C)

The Investigator rates each subject with the following question as part of Visit 5 (Week 10), Visit 6 (Week 15), and Visit 7 (Week 20) or early discontinuation:

Clinical Global Impression of Change:

In your opinion, how much has the subject's overall condition and Parkinson's disease symptoms changed since starting on the study?

❑ 1	❑ 2	❑ 3	❑ 4	❑ 5	❑ 6	❑ 7
Very Much Worse	Much Worse	Minimally Worse	Neutral	Minimally Improved	Much Improved	Very Much Improved

Guy W. ECDEU assessment manual for psychopharmacology publication; ADM, 76-338.

Washington DC, US. Department of health, education and welfare, 1976.

APPENDIX H. PATIENT GLOBAL IMPRESSION OF SEVERITY (PGI-S)

Patient Global Impression – Severity Scale

Severity of Illness

Considering the severity of your Parkinson's disease, how severe is your condition at this time?

Severity Score:

❑ 1	❑ 2	❑ 3	❑ 4	❑ 5	❑ 6	❑ 7
Normal, not at all ill	Borderline ill	Mildly ill	Moderately ill	Markedly ill	Severely ill	Extremely severely ill

APPENDIX I. CLINICAL GLOBAL IMPRESSION OF SEVERITY (CGI-S)

The Investigator will independently rate the following question of Clinical Global Impression of Severity (CGI-S) based on his/her overall impression of the study medication at Visit 1, Visit 4, and Visit 7 or early discontinuation.

Clinical Global Impression – Severity Scale

Severity of Illness

Considering your total clinical experience with this particular PD population, how ill is the patient at this time?

Severity Score:

❑ 1	❑ 2	❑ 3	❑ 4	❑ 5	❑ 6	❑ 7
Normal, not at all ill	Borderline ill	Mildly ill	Moderately ill	Markedly ill	Severely ill	Among the most extremely ill of subjects

Guy W. ECDEU assessment manual for psychopharmacology publication; ADM, 76-338.

Washington DC, US. Department of health, education and welfare, 1976.

APPENDIX J. 39-ITEM PARKINSON'S DISEASE QUESTIONNAIRE (PDQ-39)

PDQ-39 QUESTIONNAIRE

Please complete the following

Please tick one box for each question

Due to having Parkinson's disease, how often during the last month have you....		Never	Occasionally	Sometimes	Often	Always or cannot do at all
1	Had difficulty doing the leisure activities which you would like to do?	☐	☐	☐	☐	☐
2	Had difficulty looking after your home, e.g. DIY, housework, cooking?	☐	☐	☐	☐	☐
3	Had difficulty carrying bags of shopping?	☐	☐	☐	☐	☐
4	Had problems walking half a mile?	☐	☐	☐	☐	☐
5	Had problems walking 100 yards?	☐	☐	☐	☐	☐
6	Had problems getting around the house as easily as you would like?	☐	☐	☐	☐	☐
7	Had difficulty getting around in public?	☐	☐	☐	☐	☐
8	Needed someone else to accompany you when you went out?	☐	☐	☐	☐	☐
9	Felt frightened or worried about falling over in public?	☐	☐	☐	☐	☐
10	Been confined to the house more than you would like?	☐	☐	☐	☐	☐
11	Had difficulty washing yourself?	☐	☐	☐	☐	☐
12	Had difficulty dressing yourself?	☐	☐	☐	☐	☐
13	Had problems doing up your shoe laces?	☐	☐	☐	☐	☐

Please check that you have ticked ***one box for each question*** *before going on to the next page*

Due to having Parkinson's disease, how often <u>during the last month</u> have you....

Please tick <u>one</u> box for each question

		Never	Occasionally	Sometimes	Often	Always<br>or cannot do at all
14	Had problems writing clearly?	☐	☐	☐	☐	☐
15	Had difficulty cutting up your food?	☐	☐	☐	☐	☐
16	Had difficulty holding a drink without spilling it?	☐	☐	☐	☐	☐
17	Felt depressed?	☐	☐	☐	☐	☐
18	Felt isolated and lonely?	☐	☐	☐	☐	☐
19	Felt weepy or tearful?	☐	☐	☐	☐	☐
20	Felt angry or bitter?	☐	☐	☐	☐	☐
21	Felt anxious?	☐	☐	☐	☐	☐
22	Felt worried about your future?	☐	☐	☐	☐	☐
23	Felt you had to conceal your Parkinson's from people?	☐	☐	☐	☐	☐
24	Avoided situations which involve eating or drinking in public?	☐	☐	☐	☐	☐
25	Felt embarrassed in public due to having Parkinson's disease?	☐	☐	☐	☐	☐
26	Felt worried by other people's reaction to you?	☐	☐	☐	☐	☐
27	Had problems with your close personal relationships?	☐	☐	☐	☐	☐
28	Lacked support in the ways you need from your spouse or partner?	☐	☐	☐	☐	☐
	If you do not have a spouse or partner tick here		☐			
29	Lacked support in the ways you need from your family or close friends?	☐	☐	☐	☐	☐

*Please check that you have ticked **one box for each question** before going on to the next page*

Page 4 of 12 Questionnaires for patient completion

Please tick <u>one</u> box for each question

Due to having Parkinson's disease, how often <u>during the last month</u> have you....		**Never**	**Occasionally**	**Sometimes**	**Often**	**Always**
30	Unexpectedly fallen asleep during the day?	☐	☐	☐	☐	☐
31	Had problems with your concentration, e.g. when reading or watching TV?	☐	☐	☐	☐	☐
32	Felt your memory was bad?	☐	☐	☐	☐	☐
33	Had distressing dreams or hallucinations?	☐	☐	☐	☐	☐
34	Had difficulty with your speech?	☐	☐	☐	☐	☐
35	Felt unable to communicate with people properly?	☐	☐	☐	☐	☐
36	Felt ignored by people?	☐	☐	☐	☐	☐
37	Had painful muscle cramps or spasms?	☐	☐	☐	☐	☐
38	Had aches and pains in your joints or body?	☐	☐	☐	☐	☐
39	Felt unpleasantly hot or cold?	☐	☐	☐	☐	☐

*Please check that you have ticked **one box for each question** before going on to the next page*

Thank you for completing the PDQ 39 questionnaire

APPENDIX K. GASTROPARESIS CARDINAL SYMPTOM INDEX (GCSI)

GASTROPARESIS CARDINAL SYMPTOM INDEX

This questionnaire asks you about the severity of symptoms you may have related to your gastrointestinal problem. There are no right or wrong answers. Please answer each question as accurately as possible.

For each symptom, please circle the number that best describes how severe the symptom has been during the past 2 weeks.

- If you have not experienced this symptom, circle 0.
- If the symptom has been very mild, circle 1.
- If the symptom has been mild, circle 2.
- If it has been moderate, circle 3.
- If it has been severe, circle 4.
- If it has been very severe, circle 5.

Please be sure to answer every question.

Please rate the severity of the following symptoms during the past 2 weeks.

		None	Very mild	Mild	Moderate	Severe	Very severe
1.	nausea (feeling sick to your stomach as if you were going to vomit or throw up)	0	1	2	3	4	5
2.	retching (heaving as if to vomit, but nothing comes up)	0	1	2	3	4	5
3.	vomiting	0	1	2	3	4	5
4.	stomach fullness	0	1	2	3	4	5
5.	not able to finish a normal-sized meal	0	1	2	3	4	5
6.	feeling excessively full after meals	0	1	2	3	4	5
7.	loss of appetite	0	1	2	3	4	5
8.	bloating (feeling like you need to loosen your clothes)	0	1	2	3	4	5
9.	stomach or belly visibly larger	0	1	2	3	4	5

©2002 Johnson & Johnson Pharmaceutical Services, LLC. Contact D. Dubois for permission to use.

APPENDIX L. NON-MOTOR SYMPTOM ASSESSMENT SCALE FOR PARKINSON'S DISEASE (NMSS)

Non-Motor Symptom assessment scale for Parkinson's Disease

Patient ID No: ________________________ **Initials:** ________________ **Age:** __________

Symptoms assessed over the last month. Each symptom scored with respect to:

Severity: 0 = None, 1 = Mild: symptoms present but causes little distress or disturbance to patient; 2 = Moderate: some distress or disturbance to patient; 3 = Severe: major source of distress or disturbance to patient.

Frequency: 1 = Rarely (<1/wk); 2 = Often (1/wk); 3 = Frequent (several times per week); 4 = Very Frequent (daily or all the time)

Domains will be weighed differentially. Yes/ No answers are not included in final frequency x severity calculation.
(Bracketed text in questions within the scale is included as an explanatory aid).

Domain 1: Cardiovascular including falls	Severity	Frequency	Frequency x Severity
1. Does the patient experience light-headedness, dizziness, weakness on standing from sitting or lying position?	☐	☐	☐
2. Does the patient fall because of fainting or blacking out?	☐	☐	☐
**SCORE:**			☐

Domain 2: Sleep/fatigue	Severity	Frequency	Frequency x Severity
3. Does the patient doze off or fall asleep unintentionally during daytime activities? (For example, during conversation, during mealtimes, or while watching television or reding).	☐	☐	☐
4. Does fatigue (tiredness) or lack of energy (not slowness) limit the patient's daytime activities?	☐	☐	☐
5. Does the patient have difficulties falling or staying asleep?	☐	☐	☐
6. Does the patient experience an urge to move the legs or restlessness in legs that improves with movement when he/she is sitting or lying down inactive?	☐	☐	☐
**SCORE:**			☐

Domain 3: Mood /Cognition	Severity	Frequency	Frequency x Severity
7. Has the patient lost interest in his/her surroundings?	☐	☐	☐
8. Has the patient lost interest in doing things or lack motivation to start new activities?	☐	☐	☐
9. Does the patient feel nervous, worried or frightened for no apparent reason?	☐	☐	☐
10. Does the patient seem sad or depressed or has he/she reported such feelings?	☐	☐	☐
11. Does the patient have flat moods without the normal "highs" and " lows"?	☐	☐	☐
12. Does the patient have difficulty in experiencing pleasure from their usual activities or report that they lack pleasure?	☐	☐	☐
**SCORE:**			☐

Domain 4: Perceptual problems/hallucinations	Severity	Frequency	Frequency x Severity
13. Does the patient indicate that he/she sees things that are not there?	☐	☐	☐
14. Does the patient have beliefs that you know are not true? (For example, about being harmed, being robbed or being unfaithful)	☐	☐	☐
15. Does the patient experience double vision? (2 separate real objects and not blurred vision)	☐	☐	☐
**SCORE:**			☐

	Severity	Frequency	Frequency x Severity
**Domain 5: Attention/ Memory**			
16. Does the patient have problems sustaining concentration during activities? (For example, reading or having a conversation)	☐	☐	☐
17. Does the patient forget things that he/she has been told a short time ago or events that happened in the last few days?	☐	☐	☐
18. Does the patient forget to do things? (For example, take tablets or turn off domestic appliances?)	☐	☐	☐
**SCORE:**			☐
**Domain 6: Gastrointestinal tract**			
19. Does the patient dribble saliva during the day?	☐	☐	☐
20. Does the patient having difficulty swallowing?	☐	☐	☐
21. Does the patient suffer from constipation? (Bowel action less than three times weekly)	☐	☐	☐
**SCORE:**			☐
**Domain 7: Urinary**			
22. Does the patient have difficulty holding urine? (Urgency)	☐	☐	☐
23. Does the patient have to void within 2 hours of last voiding? (Frequency)	☐	☐	☐
24. Does the patient have to get up regularly at night to pass urine? (Nocturia)	☐	☐	☐
**SCORE:**			☐
**Domain 8: Sexual function**			
25. Does the patient have altered interest in sex? (Very much increased or decreased, please underline)	☐	☐	☐
26. Does the patient have problems having sex?	☐	☐	☐
**SCORE:**			☐
**Domain 9: Miscellaneous**			
27. Does the patient suffer from pain not explained by other known conditions? (Is it related to intake of drugs and is it relieved by antiparkinson drugs?)	☐	☐	☐
28. Does the patient report a change in ability to taste or smell?	☐	☐	☐
29. Does the patient report a recent change in weight (not related to dieting)?	☐	☐	☐
30. Does the patient experience excessive sweating? (not related to hot weather)	☐	☐	☐
**SCORE:**			☐
**TOTAL SCORE:**			☐

Developed by the International Parkinson's Disease Non- Motor Group.
Contacts: ray.chaudhuri@uhl.nhs.uk or alison.forbes@uhl.nhs.uk

APPENDIX M. PARKINSON'S DISEASE SLEEP SCALE-2 (PDSS-2)

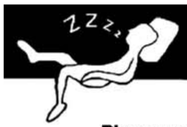

Parkinson's Disease Sleep Scale (PDSS-2)

Please rate the severity of the following based on your experiences during the past week (7 days). Please make a cross in the answer box

	Very often (This means 6 to 7 days a week)	Often (This means 4 to 5 days a week)	Sometimes (This means 2 to 3 days a week)	Occasionally (This means 1 day a week)	Never
1) Overall, did you sleep well during the last week?	☐ 0	☐ 1	☐ 2	☐ 3	☐ 4
2) Did you have difficulty falling asleep each night?	☐ 4	☐ 3	☐ 2	☐ 1	☐ 0
3) Did you have difficulty staying asleep?	☐ 4	☐ 3	☐ 2	☐ 1	☐ 0
4) Did you have restlessness of legs or arms at nights causing disruption of sleep?	☐ 4	☐ 3	☐ 2	☐ 1	☐ 0
5) Was your sleep disturbed due to an urge to move your legs or arms?	☐ 4	☐ 3	☐ 2	☐ 1	☐ 0
6) Did you suffer from distressing dreams at night?	☐ 4	☐ 3	☐ 2	☐ 1	☐ 0
7) Did you suffer from distressing hallucinations at night (seeing or hearing things that you are told do not exist)?	☐ 4	☐ 3	☐ 2	☐ 1	☐ 0
8) Did you get up at night to pass urine?	☐ 4	☐ 3	☐ 2	☐ 1	☐ 0
9) Did you feel uncomfortable at night because you were unable to turn around in bed or move due to immobility?	☐ 4	☐ 3	☐ 2	☐ 1	☐ 0
10) Did you feel pain in your arms or legs which woke you up from sleep at night?	☐ 4	☐ 3	☐ 2	☐ 1	☐ 0
11) Did you have muscle cramps in your arms or legs which woke you up whilst sleeping at night?	☐ 4	☐ 3	☐ 2	☐ 1	☐ 0
12) Did you wake early in the morning with painful posturing of arms and legs?	☐ 4	☐ 3	☐ 2	☐ 1	☐ 0
13) On waking, did you experience tremor?	☐ 4	☐ 3	☐ 2	☐ 1	☐ 0
14) Did you feel tired and sleepy after waking in the morning?	☐ 4	☐ 3	☐ 2	☐ 1	☐ 0
15) Did you wake up at night due to snoring or difficulties with breathing?	☐ 4	☐ 3	☐ 2	☐ 1	☐ 0

Trenkwalder C, Kohnen R, Högl B, Metta V, Sixel-Döring F, Frauscher B, Hülsmann J, Martinez-Martin P, Chaudhuri KR. Parkinson's disease sleep scale--validation of the revised version PDSS-2. Mov Disord. 2011;26(4):644-52.

APPENDIX N. PARKINSON ANXIETY SCALE (PAS)

The Parkinson Anxiety Scale (PAS)

(Please mark one circle for each item below)

In the past four weeks, to what extent did you experience the following symptoms?

A. Persistent Anxiety

A.1. Feeling anxious or nervous

- o Not at all, or never
- o Very mild, or rarely
- o Mild, or sometimes
- o Moderate, or often
- o Severe, or (nearly) always

A.2. Feeling tense or stressed

- o Not at all, or never
- o Very mild, or rarely
- o Mild, or sometimes
- o Moderate, or often
- o Severe, or (nearly) always

A.3. Being unable to relax

- o Not at all, or never
- o Very mild, or rarely
- o Mild, or sometimes
- o Moderate, or often
- o Severe, or (nearly) always

A.4. Excessive worrying about everyday matters

- o Not at all, or never
- o Very mild, or rarely
- o Mild, or sometimes
- o Moderate, or often
- o Severe, or (nearly) always

A.5. Fear of something bad, or even the worst, happening

- o Not at all, or never
- o Very mild, or rarely
- o Mild, or sometimes
- o Moderate, or often
- o Severe, or (nearly) always

B. Episodic Anxiety

B.1. Panic or intense fear

- Never
- Rarely
- Sometimes
- Often
- Nearly always

B.2. Shortness of breath

- Never
- Rarely
- Sometimes
- Often
- Nearly always

B.3. Heart palpitations or heart beating fast (not related to physical effort or activity)

- Never
- Rarely
- Sometimes
- Often
- Nearly always

B.4. Fear of losing control

- Never
- Rarely
- Sometimes
- Often
- Nearly always

C. Avoidance Behavior

C.1. Social situations (where one may be observed, or evaluated by others, such as speaking in public, or talking to unknown people)

- o Never
- o Rarely
- o Sometimes
- o Often
- o Nearly always

C.2. Public settings (situations from which it may be difficult or embarrassing to escape, such as queues or lines, crowds, bridges, or public transportation)

- o Never
- o Rarely
- o Sometimes
- o Often
- o Nearly always

C.3. Specific objects or situations (such as flying, heights, spiders or other animals, needles, or blood)

- o Never
- o Rarely
- o Sometimes
- o Often
- o Nearly always

Copyright of this scale and it translations is held by the authors (Leentjens AF, Dujardin K, Pontone GM, Starkstein SE, Weintraub D, and Martinez-Martin P. The Parkinson Anxiety Scale (PAS): development and validation of a new anxiety scale. Mov Disord. 2014;29(8):1035-43). The scale and its translations are in the public domain and may be used without additional permission and free of charge on the condition that its source is referenced.

APPENDIX O. COLUMBIA-SUICIDE SEVERITY RATING SCALE (C-SSRS)

COLUMBIA-SUICIDE SEVERITY RATING SCALE (C-SSRS)

Baseline/Screening Version

Version 1/14/09

Posner, K.; Brent, D.; Lucas, C.; Gould, M.; Stanley, B.; Brown, G.; Fisher, P.; Zelazny, J.; Burke, A.; Oquendo, M.; Mann, J.

Disclaimer:

This scale is intended to be used by individuals who have received training in its administration. The questions contained in the Columbia-Suicide Severity Rating Scale are suggested probes. Ultimately, the determination of the presence of suicidal ideation or behavior depends on the judgment of the individual administering the scale.

Definitions of behavioral suicidal events in this scale are based on those used in **The Columbia Suicide History Form**, *developed by John Mann, MD and Maria Oquendo, MD, Conte Center for the Neuroscience of Mental Disorders (CCNMD), New York State Psychiatric Institute, 1051 Riverside Drive, New York, NY, 10032. (Oquendo M. A., Halberstam B. & Mann J. J., Risk factors for suicidal behavior: utility and limitations of research instruments. In M.B. First [Ed.]* Standardized Evaluation in Clinical Practice, *pp. 103 -130, 2003.)*

For reprints of the C-SSRS contact Kelly Posner, Ph.D., New York State Psychiatric Institute, 1051 Riverside Drive, New York, New York, 10032; inquiries and training requirements contact posnerk@nyspi.columbia.edu

© 2008 The Research Foundation for Mental Hygiene, Inc.

SUICIDAL IDEATION		
Ask questions 1 and 2. If both are negative, proceed to "Suicidal Behavior" section. If the answer to question 2 is "yes", ask questions 3, 4 and 5. If the answer to question 1 and/or 2 is "yes", complete "Intensity of Ideation" section below.	**Lifetime: Time He/She Felt Most Suicidal**	**Past __ Months**
**1. Wish to be Dead**<br>Subject endorses thoughts about a wish to be dead or not alive anymore, or wish to fall asleep and not wake up.<br>***Have you wished you were dead or wished you could go to sleep and not wake up?***<br><br>If yes, describe:	**Yes** ☐ **No** ☐	**Yes** ☐ **No** ☐
**2. Non-Specific Active Suicidal Thoughts**<br>General non-specific thoughts of wanting to end one's life/commit suicide (e.g., *"I've thought about killing myself"*) without thoughts of ways to kill oneself/associated methods, intent, or plan during the assessment period.<br>***Have you actually had any thoughts of killing yourself?***<br><br>If yes, describe:	**Yes** ☐ **No** ☐	**Yes** ☐ **No** ☐
**3. Active Suicidal Ideation with Any Methods (Not Plan) without Intent to Act**<br>Subject endorses thoughts of suicide and has thought of at least one method during the assessment period. This is different than a specific plan with time, place or method details worked out (e.g. thought of method to kill self but not a specific plan). Includes person who would say, *"I thought about taking an overdose but I never made a specific plan as to when, where or how I would actually do it...and I would never go through with it."*<br>***Have you been thinking about how you might do this?***<br><br>If yes, describe:	**Yes** ☐ **No** ☐	**Yes** ☐ **No** ☐
**4. Active Suicidal Ideation with Some Intent to Act, without Specific Plan**<br>Active suicidal thoughts of killing oneself and subject reports having some intent to act on such thoughts, as opposed to *"I have the thoughts but I definitely will not do anything about them."*<br>***Have you had these thoughts and had some intention of acting on them?***<br><br>If yes, describe:	**Yes** ☐ **No** ☐	**Yes** ☐ **No** ☐
**5. Active Suicidal Ideation with Specific Plan and Intent**<br>Thoughts of killing oneself with details of plan fully or partially worked out and subject has some intent to carry it out.<br>***Have you started to work out or worked out the details of how to kill yourself? Do you intend to carry out this plan?***<br><br>If yes, describe:	**Yes** ☐ **No** ☐	**Yes** ☐ **No** ☐

INTENSITY OF IDEATION		
The following features should be rated with respect to the most severe type of ideation (i.e., 1-5 from above, with 1 being the least severe and 5 being the most severe). Ask about time he/she was feeling the most suicidal.<br><br>Lifetime - ***Most Severe Ideation:*** ______ (*Type # (1-5)*) ______________________ (*Description of Ideation*)<br><br>Past X Months - ***Most Severe Ideation:*** ______ (*Type # (1-5)*) ______________________ (*Description of Ideation*)	Most Severe	Most Severe
**Frequency**<br>***How many times have you had these thoughts?***<br>(1) Less than once a week (2) Once a week (3) 2-5 times in week (4) Daily or almost daily (5) Many times each day	___	___
**Duration**<br>***When you have the thoughts how long do they last?***<br>(1) Fleeting - few seconds or minutes<br>(2) Less than 1 hour/some of the time<br>(3) 1-4 hours/a lot of time<br>(4) 4-8 hours/most of day<br>(5) More than 8 hours/persistent or continuous	___	___
**Controllability**<br>***Could/can you stop thinking about killing yourself or wanting to die if you want to?***<br>(1) Easily able to control thoughts<br>(2) Can control thoughts with little difficulty<br>(3) Can control thoughts with some difficulty<br>(4) Can control thoughts with a lot of difficulty<br>(5) Unable to control thoughts<br>(0) Does not attempt to control thoughts	___	___
**Deterrents**<br>***Are there things - anyone or anything (e.g., family, religion, pain of death) - that stopped you from wanting to die or acting on thoughts of committing suicide?***<br>(1) Deterrents definitely stopped you from attempting suicide<br>(2) Deterrents probably stopped you<br>(3) Uncertain that deterrents stopped you<br>(4) Deterrents most likely did not stop you<br>(5) Deterrents definitely did not stop you<br>(0) Does not apply	___	___
**Reasons for Ideation**<br>***What sort of reasons did you have for thinking about wanting to die or killing yourself? Was it to end the pain or stop the way you were feeling (in other words you couldn't go on living with this pain or how you were feeling) or was it to get attention, revenge or a reaction from others? Or both?***<br>(1) Completely to get attention, revenge or a reaction from others<br>(2) Mostly to get attention, revenge or a reaction from others<br>(3) Equally to get attention, revenge or a reaction from others and to end/stop the pain<br>(4) Mostly to end or stop the pain (you couldn't go on living with the pain or how you were feeling)<br>(5) Completely to end or stop the pain (you couldn't go on living with the pain or how you were feeling)<br>(0) Does not apply	___	___

© 2008 Research Foundation for Mental Hygiene, Inc. C-SSRS—Baseline/Screening (Version 1/14/09) Page 1 of 2

SUICIDAL BEHAVIOR *(Check all that apply, so long as these are separate events; must ask about all types)*	**Lifetime**	**Past __ Years**
**Actual Attempt:** A potentially self-injurious act committed with at least some wish to die, *as a result of act*. Behavior was in part thought of as method to kill oneself. Intent does not have to be 100%. If there is ***any*** intent/desire to die associated with the act, then it can be considered an actual suicide attempt. ***There does not have to be any injury or harm***, just the potential for injury or harm. If person pulls trigger while gun is in mouth but gun is broken so no injury results, this is considered an attempt.<br>Inferring Intent: Even if an individual denies intent/wish to die, it may be inferred clinically from the behavior or circumstances. For example, a highly lethal act that is clearly not an accident so no other intent but suicide can be inferred (e.g., gunshot to head, jumping from window of a high floor/story). Also, if someone denies intent to die, but they thought that what they did could be lethal, intent may be inferred.<br>***Have you made a suicide attempt?***<br>***Have you done anything to harm yourself?***<br>***Have you done anything dangerous where you could have died?***<br>***What did you do?***<br>***Did you______ as a way to end your life?***<br>***Did you want to die (even a little) when you_____?***<br>***Were you trying to end your life when you _____?***<br>***Or Did you think it was possible you could have died from_____?***<br>***Or did you do it purely for other reasons / without ANY intention of killing yourself (like to relieve stress, feel better, get sympathy, or get something else to happen)?*** (Self-Injurious Behavior without suicidal intent)<br>If yes, describe:	Yes No<br>☐ ☐<br>Total # of Attempts<br>_____	Yes No<br>☐ ☐<br>Total # of Attempts<br>_____
**Has subject engaged in Non-Suicidal Self-Injurious Behavior?**	Yes No<br>☐ ☐	Yes No<br>☐ ☐
**Interrupted Attempt:** When the person is interrupted (by an outside circumstance) from starting the potentially self-injurious act *(if not for that, actual attempt would have occurred)*.<br>Overdose: Person has pills in hand but is stopped from ingesting. Once they ingest any pills, this becomes an attempt rather than an interrupted attempt. Shooting: Person has gun pointed toward self, gun is taken away by someone else, or is somehow prevented from pulling trigger. Once they pull the trigger, even if the gun fails to fire, it is an attempt. Jumping: Person is poised to jump, is grabbed and taken down from ledge. Hanging: Person has noose around neck but has not yet started to hang - is stopped from doing so.<br>***Has there been a time when you started to do something to end your life but someone or something stopped you before you actually did anything?***<br>If yes, describe:	Yes No<br>☐ ☐<br>Total # of interrupted<br>_____	Yes No<br>☐ ☐<br>Total # of interrupted<br>_____
**Aborted Attempt:** When person begins to take steps toward making a suicide attempt, but stops themselves before they actually have engaged in any self-destructive behavior. Examples are similar to interrupted attempts, except that the individual stops him/herself, instead of being stopped by something else.<br>***Has there been a time when you started to do something to try to end your life but you stopped yourself before you actually did anything?***<br>If yes, describe:	Yes No<br>☐ ☐<br>Total # of aborted<br>_____	Yes No<br>☐ ☐<br>Total # of aborted<br>_____
**Preparatory Acts or Behavior:** Acts or preparation towards imminently making a suicide attempt. This can include anything beyond a verbalization or thought, such as assembling a specific method (e.g., buying pills, purchasing a gun) or preparing for one's death by suicide (e.g., giving things away, writing a suicide note).<br>***Have you taken any steps towards making a suicide attempt or preparing to kill yourself (such as collecting pills, getting a gun, giving valuables away or writing a suicide note)?***<br>If yes, describe:	Yes No<br>☐ ☐	Yes No<br>☐ ☐
**Suicidal Behavior:** Suicidal behavior was present during the assessment period?	Yes No<br>☐ ☐	Yes No<br>☐ ☐

Answer for Actual Attempts Only	Most Recent Attempt Date:	Most Lethal Attempt Date:	Initial/First Attempt Date:
**Actual Lethality/Medical Damage:**<br>0. No physical damage or very minor physical damage (e.g., surface scratches).<br>1. Minor physical damage (e.g., lethargic speech; first-degree burns; mild bleeding; sprains).<br>2. Moderate physical damage; medical attention needed (e.g., conscious but sleepy, somewhat responsive; second-degree burns; bleeding of major vessel).<br>3. Moderately severe physical damage; *medical* hospitalization and likely intensive care required (e.g., comatose with reflexes intact; third-degree burns less than 20% of body; extensive blood loss but can recover; major fractures).<br>4. Severe physical damage; *medical* hospitalization with intensive care required (e.g., comatose without reflexes; third-degree burns over 20% of body; extensive blood loss with unstable vital signs; major damage to a vital area).<br>5. Death	*Enter Code*<br>_____	*Enter Code*<br>_____	*Enter Code*<br>_____
**Potential Lethality: Only Answer if Actual Lethality=0**<br>Likely lethality of actual attempt if no medical damage (the following examples, while having no actual medical damage, had potential for very serious lethality: put gun in mouth and pulled the trigger but gun fails to fire so no medical damage; laying on train tracks with oncoming train but pulled away before run over).<br>0 = Behavior not likely to result in injury<br>1 = Behavior likely to result in injury but not likely to cause death<br>2 = Behavior likely to result in death despite available medical care	*Enter Code*<br>_____	*Enter Code*<br>_____	*Enter Code*<br>_____

© 2008 Research Foundation for Mental Hygiene, Inc. C-SSRS—Baseline/Screening (Version 1/14/09) Page 2 of 2

COLUMBIA-SUICIDE SEVERITY RATING SCALE (C-SSRS)

Since Last Visit

Version 1/14/09

Posner, K.; Brent, D.; Lucas, C.; Gould, M.; Stanley, B.; Brown, G.; Fisher, P.; Zelazny, J.; Burke, A.; Oquendo, M.; Mann, J.

Disclaimer:

This scale is intended to be used by individuals who have received training in its administration. The questions contained in the Columbia-Suicide Severity Rating Scale are suggested probes. Ultimately, the determination of the presence of suicidal ideation or behavior depends on the judgment of the individual administering the scale.

*Definitions of behavioral suicidal events in this scale are based on those used in **The Columbia Suicide History Form**, developed by John Mann, MD and Maria Oquendo, MD, Conte Center for the Neuroscience of Mental Disorders (CCNMD), New York State Psychiatric Institute, 1051 Riverside Drive, New York, NY, 10032. (Oquendo M. A., Halberstam B. & Mann J. J., Risk factors for suicidal behavior: utility and limitations of research instruments. In M.B. First [Ed.]* Standardized Evaluation in Clinical Practice, *pp. 103 -130, 2003.)*

For reprints of the C-SSRS contact Kelly Posner, Ph.D., New York State Psychiatric Institute, 1051 Riverside Drive, New York, New York, 10032; inquiries and training requirements contact posnerk@nyspi.columbia.edu

© 2008 The Research Foundation for Mental Hygiene, Inc.

SUICIDAL IDEATION

Ask questions 1 and 2. If both are negative, proceed to "Suicidal Behavior" section. If the answer to question 2 is "yes", ask questions 3, 4 and 5. If the answer to question 1 and/or 2 is "yes", complete "Intensity of Ideation" section below.	**Since Last Visit**
**1. Wish to be Dead**<br>Subject endorses thoughts about a wish to be dead or not alive anymore, or wish to fall asleep and not wake up.<br>***Have you wished you were dead or wished you could go to sleep and not wake up?***<br><br>If yes, describe:	**Yes** ☐ **No** ☐
**2. Non-Specific Active Suicidal Thoughts**<br>General, non-specific thoughts of wanting to end one's life/commit suicide (e.g., *"I've thought about killing myself"*) without thoughts of ways to kill oneself/associated methods, intent, or plan during the assessment period.<br>***Have you actually had any thoughts of killing yourself?***<br><br>If yes, describe:	**Yes** ☐ **No** ☐
**3. Active Suicidal Ideation with Any Methods (Not Plan) without Intent to Act**<br>Subject endorses thoughts of suicide and has thought of at least one method during the assessment period. This is different than a specific plan with time, place or method details worked out (e.g., thought of method to kill self but not a specific plan). Includes person who would say, *"I thought about taking an overdose but I never made a specific plan as to when, where or how I would actually do it…and I would never go through with it."*<br>***Have you been thinking about how you might do this?***<br><br>If yes, describe:	**Yes** ☐ **No** ☐
**4. Active Suicidal Ideation with Some Intent to Act, without Specific Plan**<br>Active suicidal thoughts of killing oneself and subject reports having some intent to act on such thoughts, as opposed to *"I have the thoughts but I definitely will not do anything about them."*<br>***Have you had these thoughts and had some intention of acting on them?***<br><br>If yes, describe:	**Yes** ☐ **No** ☐
**5. Active Suicidal Ideation with Specific Plan and Intent**<br>Thoughts of killing oneself with details of plan fully or partially worked out and subject has some intent to carry it out.<br>***Have you started to work out or worked out the details of how to kill yourself? Do you intend to carry out this plan?***<br><br>If yes, describe:	**Yes** ☐ **No** ☐

INTENSITY OF IDEATION

The following features should be rated with respect to the most severe type of ideation (i.e., 1-5 from above, with 1 being the least severe and 5 being the most severe).<br><br>***Most Severe Ideation:*** ____ ________________________<br>***Type # (1-5)*** ***Description of Ideation***	Most Severe
**Frequency**<br>***How many times have you had these thoughts?***<br>(1) Less than once a week (2) Once a week (3) 2-5 times in week (4) Daily or almost daily (5) Many times each day	___
**Duration**<br>***When you have the thoughts, how long do they last?***<br>(1) Fleeting - few seconds or minutes<br>(2) Less than 1 hour/some of the time<br>(3) 1-4 hours/a lot of time<br>(4) 4-8 hours/most of day<br>(5) More than 8 hours/persistent or continuous	___
**Controllability**<br>***Could/can you stop thinking about killing yourself or wanting to die if you want to?***<br>(1) Easily able to control thoughts<br>(2) Can control thoughts with little difficulty<br>(3) Can control thoughts with some difficulty<br>(4) Can control thoughts with a lot of difficulty<br>(5) Unable to control thoughts<br>(0) Does not attempt to control thoughts	___
**Deterrents**<br>***Are there things - anyone or anything (e.g., family, religion, pain of death) - that stopped you from wanting to die or acting on thoughts of committing suicide?***<br>(1) Deterrents definitely stopped you from attempting suicide<br>(2) Deterrents probably stopped you<br>(3) Uncertain that deterrents stopped you<br>(4) Deterrents most likely did not stop you<br>(5) Deterrents definitely did not stop you<br>(0) Does not apply	___
**Reasons for Ideation**<br>***What sort of reasons did you have for thinking about wanting to die or killing yourself? Was it to end the pain or stop the way you were feeling (in other words you couldn't go on living with this pain or how you were feeling) or was it to get attention, revenge or a reaction from others? Or both?***<br>(1) Completely to get attention, revenge or a reaction from others<br>(2) Mostly to get attention, revenge or a reaction from others<br>(3) Equally to get attention, revenge or a reaction from others and to end/stop the pain<br>(4) Mostly to end or stop the pain (you couldn't go on living with the pain or how you were feeling)<br>(5) Completely to end or stop the pain (you couldn't go on living with the pain or how you were feeling)<br>(0) Does not apply	___

© 2008 Research Foundation for Mental Hygiene, Inc. C-SSRS—Since Last Visit (Version 1/14/09) Page 1 of 2

SUICIDAL BEHAVIOR *(Check all that apply, so long as these are separate events; must ask about all types)*	**Since Last Visit**
**Actual Attempt:**<br>A potentially self-injurious act committed with at least some wish to die, *as a result of act.* Behavior was in part thought of as method to kill oneself. Intent does not have to be 100%. If there is ***any*** intent/desire to die associated with the act, then it can be considered an actual suicide attempt. ***There does not have to be any injury or harm,*** just the potential for injury or harm. If person pulls trigger while gun is in mouth but gun is broken so no injury results, this is considered an attempt.<br>Inferring Intent: Even if an individual denies intent/wish to die, it may be inferred clinically from the behavior or circumstances. For example, a highly lethal act that is clearly not an accident so no other intent but suicide can be inferred (e.g., gunshot to head, jumping from window of a high floor/story). Also, if someone denies intent to die, but they thought that what they did could be lethal, intent may be inferred.<br>***Have you made a suicide attempt?***<br>***Have you done anything to harm yourself?***<br>***Have you done anything dangerous where you could have died?***<br>***What did you do?***<br>***Did you______ as a way to end your life?***<br>***Did you want to die (even a little) when you_____?***<br>***Were you trying to end your life when you _____?***<br>***Or did you think it was possible you could have died from_____?***<br>***Or did you do it purely for other reasons / without ANY intention of killing yourself (like to relieve stress, feel better, get sympathy, or get something else to happen)?*** (Self-Injurious Behavior without suicidal intent)<br>If yes, describe:	**Yes** **No**<br>☐ ☐<br>Total # of Attempts<br>_____
**Has subject engaged in Non-Suicidal Self-Injurious Behavior?**	**Yes** **No**<br>☐ ☐
**Interrupted Attempt:**<br>When the person is interrupted (by an outside circumstance) from starting the potentially self-injurious act *(if not for that, actual attempt would have occurred).*<br>Overdose: Person has pills in hand but is stopped from ingesting. Once they ingest any pills, this becomes an attempt rather than an interrupted attempt. Shooting: Person has gun pointed toward self, gun is taken away by someone else, or is somehow prevented from pulling trigger. Once they pull the trigger, even if the gun fails to fire, it is an attempt. Jumping: Person is poised to jump, is grabbed and taken down from ledge. Hanging: Person has noose around neck but has not yet started to hang - is stopped from doing so.<br>***Has there been a time when you started to do something to end your life but someone or something stopped you before you actually did anything?***<br>If yes, describe:	**Yes** **No**<br>☐ ☐<br>Total # of interrupted<br>_____
**Aborted Attempt:**<br>When person begins to take steps toward making a suicide attempt, but stops themselves before they actually have engaged in any self-destructive behavior. Examples are similar to interrupted attempts, except that the individual stops him/herself, instead of being stopped by something else.<br>***Has there been a time when you started to do something to try to end your life but you stopped yourself before you actually did anything?***<br>If yes, describe:	**Yes** **No**<br>☐ ☐<br>Total # of aborted<br>_____
**Preparatory Acts or Behavior:**<br>Acts or preparation towards imminently making a suicide attempt. This can include anything beyond a verbalization or thought, such as assembling a specific method (e.g., buying pills, purchasing a gun) or preparing for one's death by suicide (e.g., giving things away, writing a suicide note).<br>***Have you taken any steps towards making a suicide attempt or preparing to kill yourself (such as collecting pills, getting a gun, giving valuables away or writing a suicide note)?***<br>If yes, describe:	**Yes** **No**<br>☐ ☐
**Suicidal Behavior:**<br>Suicidal behavior was present during the assessment period?	**Yes** **No**<br>☐ ☐
**Suicide:**	**Yes** **No**<br>☐ ☐
Answer for Actual Attempts Only	Most Lethal Attempt Date:
**Actual Lethality/Medical Damage:**<br>0. No physical damage or very minor physical damage (e.g., surface scratches).<br>1. Minor physical damage (e.g., lethargic speech; first-degree burns; mild bleeding; sprains).<br>2. Moderate physical damage; medical attention needed (e.g., conscious but sleepy, somewhat responsive; second-degree burns; bleeding of major vessel).<br>3. Moderately severe physical damage; *medical* hospitalization and likely intensive care required (e.g., comatose with reflexes intact; third-degree burns less than 20% of body; extensive blood loss but can recover; major fractures).<br>4. Severe physical damage; *medical* hospitalization with intensive care required (e.g., comatose without reflexes; third-degree burns over 20% of body; extensive blood loss with unstable vital signs; major damage to a vital area).<br>5. Death	*Enter Code*<br>_____
**Potential Lethality: Only Answer if Actual Lethality=0**<br>Likely lethality of actual attempt if no medical damage (the following examples, while having no actual medical damage, had potential for very serious lethality: put gun in mouth and pulled the trigger but gun fails to fire so no medical damage; laying on train tracks with oncoming train but pulled away before run over).<br>0 = Behavior not likely to result in injury<br>1 = Behavior likely to result in injury but not likely to cause death<br>2 = Behavior likely to result in death despite available medical care	*Enter Code*<br>_____

© 2008 Research Foundation for Mental Hygiene, Inc. C-SSRS—Since Last Visit (Version 1/14/09) Page 2 of 2

APPENDIX P. PARKINSON'S DISEASE DIARY

PARKINSON'S DISEASE DIARY

NAME ____________________________ DATE ________________

Instructions: For each half-hour time period place one check mark to indicate your predominant states during most of that period.
ON = Time when medication is providing benefit with regard to mobility, slowness, and stiffness.
OFF = Time when medication has worn off and is no longer providing benefit with regard to mobility, slowness, and stiffness.
Dyskinesia = involuntary twisting, turning movements. These movements are an effect of medication and occur during ON time.
Non-troublesome dyskinesia does not interfere with function or cause meaningful discomfort. Troublesome dyskinesia interferes with function or causes meaningful discomfort.
Tremor is shaking back and forth and is not considered dyskinesia.

time	asleep	OFF	ON without dyskinesia	ON with non-troublesome dyskinesia	ON with troublesome dyskinesia
6:00 AM					
:30					
7:00 AM					
:30					
8:00 AM					
:30					
9:00 AM					
:30					
10:00 AM					
:30					
11:00 AM					
:30					
12:00 PM					
:30					
1:00 PM					
:30					
2:00 PM					
:30					
3:00 PM					
:30					
4:00 PM					
:30					
5:00 PM					
:30					

time	asleep	OFF	ON without dyskinesia	ON with non-troublesome dyskinesia	ON with troublesome dyskinesia
6:00 PM					
:30					
7:00 PM					
:30					
8:00 PM					
:30					
9:00 PM					
:30					
10:00 PM					
:30					
11:00 PM					
:30					
12:00 AM					
:30					
1:00 AM					
:30					
2:00 AM					
:30					
3:00 AM					
:30					
4:00 AM					
:30					
5:00 AM					
:30					

© RA Hauser 1995

APPENDIX Q. EXCIPIENTS IN IPX203, IPX203 PLACEBO, IR CD-LD, AND IR PLACEBO

IPX203 (Various Strengths)	IPX203 Placebo	IR CD-LD (25-100 mg)	IR Placebo
Microcrystalline Cellulose, NF	Microcrystalline Cellulose, NF	Crospovidone	Microcrystalline cellulose, NF
Croscarmellose Sodium, NF	Talc, USP	Hydroxypropyl Cellulose	Magnesium Stearate, NF
Magnesium Stearate, NF	Magnesium Stearate, NF	Magnesium Stearate	Quinoline yellow E104
Mannitol, USP	Sugar Spheres, NF	Microcrystalline cellulose	
Sodium Lauryl Sulfate, NF	Methacrylic acid copolymer Type A, NF	Starch (corn)	
Povidone, USP	Triethyl citrate, NF	D&C Yellow No. 10	
Cellulose Acetate	Hard gelatin capsules	Aluminum Oxide	
Copovidone, NF			
Amino Methacrylate Copolymer, NF			
Methacrylic acid copolymer Type A, NF			
Triethyl Citrate, NF			
Talc, USP			
Hard gelatin capsules			

APPENDIX R. CLINICAL LABORATORY STUDIES

HEMATOLOGY

hemoglobin	% lymphocytes	absolute lymphocytes
hematocrit	% monocytes	absolute monocytes
red blood cell count	% basophils	absolute basophils
white blood cell count	% eosinophils	absolute eosinophils
% neutrophils	absolute neutrophils	platelet count

CHEMISTRY

sodium	calcium	indirect bilirubin
potassium	phosphorous	alkaline phosphatase
chloride	albumin	alanine aminotransferase (ALT, SGPT)
carbon dioxide	total protein	aspartate aminotransferase (AST, SGOT)
blood urea nitrogen (BUN)	uric acid	creatine phosphokinase
creatinine	total bilirubin	lactate dehydrogenase
glucose	direct bilirubin	

URINALYSIS

pH	ketones	leukocyte esterase
specific gravity	microscopic exam (RBC and WBC, only when indicated)	protein
blood		
glucose		

URINE DRUG TEST

amphetamines	benzodiazepines
barbiturates	
cannabinoids	
cocaine metabolites	
opiates	
phencyclidines	

ALCOHOL BREATH TEST

PREGNANCY TEST
Urine pregnancy test (to be completed on site) for female subjects of childbearing potential.

IPX203 (CARBIDOPA-LEVODOPA) EXTENDED-RELEASE CAPSULES

IPX203-B16-02

A RANDOMIZED CONTROLLED STUDY TO COMPARE THE SAFETY AND EFFICACY OF IPX203 WITH IMMEDIATE-RELEASE CARBIDOPA-LEVODOPA IN PARKINSON'S DISEASE PATIENTS WITH MOTOR FLUCTUATIONS

SPONSOR
Impax Laboratories, Inc.,
acting through its Impax Specialty Pharma division (Impax)
30831 Huntwood Ave.
Hayward, CA 94544

Original Protocol, May 18, 2017
Amendment 1, August 30, 2017
Amendment 2, October 23, 2017
Amendment 3, December 07, 2017

CONFIDENTIALITY STATEMENT

All information provided is the property of Impax Laboratories, Inc. and may not be divulged, published or otherwise disclosed without written consent of Impax. All information provided to the Investigator by the Sponsor, including clinical observations at the investigative site, shall be held strictly confidential and confined to the clinical personnel involved in conducting the study, under the supervision of the Investigator. This includes but is not limited to preclinical data, protocols, case report forms, and verbal or written communications. This information may be related in confidence to the Institutional Review Board or other committees functioning in a similar capacity. All reports, subject samples, data published or submitted to third parties will be identified by a coded number and initials only in order to maintain subject confidentiality.

SIGNATURE PAGE

Reviewed and approved by:

[signature]	07 DEC 17
Robert Rubens, MD, MBA<br>Senior Director, Clinical Development	Date
[signature]	07 Dec 17
Barbara Pruitt, RN, BS<br>Associate Director, Clinical Operations	Date
[signature] For	07 DEC 17
Yoshiko Stowell, PhD<br>Manager, Regulatory Affairs	Date
[signature]	07 DEC 17
Phillip Dinh, PhD<br>Director, Statistics	Date
[signature]	07 DEC 17
Aravind Mittur, PhD<br>Director, Clinical Pharmacology	Date

INVESTIGATOR'S AGREEMENT

Protocol No.: IPX203-B16-02

Protocol Title: A Randomized Controlled Study to Compare the Safety and Efficacy of IPX203 with Immediate-Release Carbidopa-Levodopa in Parkinson's Disease Patients with Motor Fluctuations

I have read this protocol and agree to conduct the study as outlined herein, complying with the obligations and requirements of clinical investigators and all other requirements of International Conference on Harmonization (ICH), Good Clinical Practice (GCP), and the appropriate regulatory authority.

I will provide copies of the protocol and all pertinent information to all individuals responsible to me who assist in the conduct of this clinical study. I will discuss this material with them to ensure that they are fully informed regarding the study medication, the conduct of the study, and the obligations of confidentiality.

____________________________________ ____________________

Principal Investigator's signature Date

____

Principal Investigator's printed name

STUDY CONTACT INFORMATION

Changes in Impax study personnel listed on this page do not require a protocol amendment.

**Role**	**Name and Contact Information**
Sponsor	Impax Laboratories, Inc. acting through its Impax Specialty Pharma division (Impax)<br>30831 Huntwood Avenue<br>Hayward, CA 94544-7037<br>(510) 240-6000<br>FAX: (510) 240-6113 and (510) 240-6114
Medical Monitor	Robert Rubens, MD, MBA<br>Senior Director, Clinical Research & Development<br>Mobile: (510) 303-8207<br>Office: (510) 240-6012<br>E-mail: rrubens@impaxlabs.com
Statistician	Phillip Dinh, PhD<br>Director, Statistics<br>(510) 240-6402<br>E-mail: Phillip.Dinh@impaxlabs.com
Clinical Pharmacology	Aravind Mittur, PhD<br>Director, Clinical Pharmacology<br>(510) 240-6437<br>E-mail: amittur@impaxlabs.com
Clinical Research Associates	Barbara Pruitt, RN, BS<br>Associate Director, Clinical Operations<br>Office: (510) 240-6003<br>Mobile: (510) 329-8489<br>E-mail: bpruitt@impaxlabs.com
	Weiru Hong, MS<br>Senior CRA, Clinical Operations<br>Office: (510) 240-6078<br>Mobile: (510) 731-8309<br>E-mail: whong@impaxlabs.com
Serious Adverse Event Reporting	FAX: (866) 954-2064

1. SYNOPSIS

**Name of Sponsor/Company:** Impax Laboratories, Inc. acting through its Impax Specialty Pharma division (Impax)
**Name of Investigational Product:** IPX203 (carbidopa-levodopa) Extended-Release Capsules
**Name of Active Ingredients:** carbidopa (CD), levodopa (LD)
**Protocol Title:** A Randomized Controlled Study to Compare the Safety and Efficacy of IPX203 with Immediate-Release Carbidopa-Levodopa in Parkinson's Disease Patients with Motor Fluctuations
**Protocol No.:** IPX203-B16-02
**Study center(s):** Multicenter
**Phase of Development:** Phase 3
**Objectives:** To evaluate the safety and efficacy of IPX203 in comparison to IR CD-LD in the treatment of CD-LD-experienced subjects with Parkinson's disease (PD) who have motor fluctuations.
**Methodology:** This is a multicenter, randomized, double-blind, double-dummy, active-controlled, parallel-group study. The study will consist of a 3-week, open-label IR CD-LD dose adjustment period; a 4-week, open-label period for conversion to IPX203; followed by a 13-week double-blind treatment period with subjects randomized in a 1:1 ratio, stratified by center, to receive either IPX203 (with matching IR CD-LD placebo) or IR CD-LD (with matching IPX203 placebo).<br>Subjects will continue to take permitted non-CD-LD-based PD medications throughout the study if documented in their prestudy regimen and if dosing regimens have been stable for at least 4 weeks prior to Visit 1. A "stable dosing regimen" means no change in dose or in dosing frequency.<br>• Within 4 weeks following the Screening visit, eligible subjects will complete their PD Diaries on each of the 3 consecutive days immediately prior to Visit 1.<br>• Following Visit 1, qualified subjects will enter a 3-week, open-label IR CD-LD treatment period allowing for dose adjustment. **The dosing regimen of IR CD-LD may be adjusted during the dose adjustment period** to minimize "Off" time without causing troublesome dyskinesia. The doses and regimens of the subject's other non-CD-LD PD medications (dopamine agonists, MAO-B inhibitors, amantadine, anticholinergics) should remain stable throughout this study. Any adjustments to the IR CD-LD dosing regimen will be done in consultation with the Investigator or qualified site personnel and will be recorded. The IR CD-LD dosing regimen should be stable for at least 5 days prior to returning for Visit 2. Rescue with additional or modified doses of concomitant PD medications or with use of CD-LD products other than the dispensed study medications is not permitted and will trigger discontinuation from the study. Subjects will complete their 3-day PD Diaries on each of the 3 consecutive days immediately prior to Visit 2.<br>• Following completion of the IR CD-LD dose adjustment period, subjects will begin a 4-week open-label period for conversion to IPX203. The initial dosing regimen of IPX203 will be based on the most frequent dose of the subject's dosing regimen of IR CD-LD at the end of the dose adjustment period (Visit 2). A 25-100 mg dose of IR CD-LD converts to a 70-280 mg CD-LD dose of IPX203 (2 capsules of 35-140 mg CD-LD IPX203), and a 12.5-50 mg dose of IR CD-LD converts to a 35-140 mg CD-LD dose of IPX203, but with a longer duration of effect. It is recommended that IPX203 should be dosed approximately every 8 hours with the exception that subjects who are currently receiving a total daily dose of less than 125-500 mg

IR CD-LD at the end of the dose adjustment period will be initially administered every 12 hours. The dosing interval may be reduced to approximately every 8 hours if the subject does not achieve an acceptable duration of effect. **The dosing regimen of IPX203 may be adjusted during the dose conversion period** to achieve the optimal balance of efficacy and tolerability (minimize "Off" time without causing troublesome dyskinesia or other dopaminergic side effects). The doses and regimens of the subject's other non-CD-LD PD medications (dopamine agonists, MAO-B inhibitors, amantadine, anticholinergics) should remain stable throughout this study. The subject must be on a stable dosing regimen of IPX203 (no change in dose or in dosing frequency) for at least 5 days prior to returning for Visit 4. Any adjustments to the IPX203 dosing regimen will be done in consultation with the Investigator or qualified site personnel and will be recorded. Rescue with additional or modified doses of concomitant PD medications or with use of CD-LD products other than the dispensed study medications is not permitted and will trigger discontinuation from the study. Subjects will return to the clinic in 2 weeks for Visit 3 followed by Visit 4, 2 weeks later. Subjects will complete their 3-day PD Diaries on each of the 3 consecutive days immediately prior to Visit 4.

- Subjects who successfully complete the IPX203 dose conversion period will be randomized in 1:1 ratio, stratified by center, at Visit 4 into one of two parallel treatment arms of IPX203 (with matching IR CD-LD placebo) or IR CD-LD (with matching IPX203 placebo). The subjects will undergo 13 weeks of double-blind maintenance therapy with the stable dosing regimen established at the end of Week 3 (Visit 2) for IR CD-LD and at the end of Week 7 (Visit 4) for IPX203. Rescue with additional or modified doses of concomitant PD medications or with use of CD-LD products other than the dispensed study medications is not permitted and will trigger discontinuation from the study. Subjects will return to the clinic for 3 visits (Visits 5, 6, and 7) and will complete their 3-day PD Diaries on each of the 3 consecutive days immediately prior to each of these visits.

Number of patients (planned): Assuming approximately an 18% prerandomization drop-out, approximately 510 subjects will be enrolled to randomize 420 subjects.

Diagnosis and main criteria for inclusion:

Inclusion Criteria

- Male or female subjects diagnosed at age ≥ 40 years with PD, consistent with the United Kingdom Parkinson's Disease Society Brain Bank Diagnostic Criteria and who are being treated with stable regimens of CD-LD but experiencing motor fluctuations.
- Hoehn and Yahr Stages 1, 2, 3, or 4 in the "On" state (part of Movement Disorders Society version of the Unified Parkinson's Disease Rating Scale [MDS-UPDRS] Part III)
- Montreal Cognitive Assessment (MoCA) score ≥ 24 at Screening Visit in "On" state.
- By history, for the 4 weeks prior to Screening, the subject experiences daily "wearing-off" episodes with periods of bradykinesia in combination with at least one of rest tremor or rigidity, experiences an "Off" state upon awakening on most mornings, and reports an average of at least 2.5 cumulative hours per day of "Off" time during the waking hours.
- Able to differentiate "On" state from "Off" state as determined by at least 75% concordance with a trained rater in "On/Off" ratings for 8 ratings over a 4-hour training period. The concordance must include at least 1 "On" and 1 "Off" rating and must be achieved within two 4-hour training sessions.
- At Visit 1, review of the 3-day PD Diaries confirms the following: that the subject is able to properly complete the Diaries with valid entries; and that the subject has an average of at least 2.5 hours per day of "Off" time during waking hours over the 3 days with at least 1.5 hours of

cumulative “Off” time on each day.

- Responsive to CD-LD therapy and currently being treated on a stable regimen with CD-LD for at least 4 weeks prior to Visit 1 and:
  - Requires at least 100 mg of LD from IR CD-LD for the first morning dose
  - Requires a total daily dose of at least 400 mg of LD and takes a maximum total daily dose of 2400 mg LD, from IR CD-LD alone or IR CD-LD in combination with a single daily bedtime dose of CR CD-LD
  - Has a dosing frequency of 4 to 9 times daily of CD-LD
  - By history, typically experiences an “On” response with the first dose of IR CD-LD of the day, but the efficacy of this dose typically lasts less than 4 hours.
- At Screening, the subject has predictable “Off” periods defined by a score of 1 or 2 on Item #4.5 (Complexity of Motor Fluctuations) of the MDS-UPDRS Part IV B (Motor Fluctuations).
- At Screening, the MDS-UPDRS Part III total score in the “Off” state is at least 20 units.

Exclusion Criteria

- Used any doses of controlled-release (CR) CD-LD apart from a single daily bedtime dose within 4 weeks prior to Visit 1.
- Used any dose of Rytary for the past 4 weeks prior to Visit 1 or were considered IPX066 or Rytary failures for reasons of efficacy or safety.
- Had prior neurosurgical treatment for PD or if such procedure is planned or anticipated during the study period.
- Allergic to any excipient in the study drugs.
- History of glaucoma with intraocular pressures that are elevated despite appropriate medical management.
- History of seizure or epilepsy and experienced at least 1 seizure during the past 12 months or has not been compliant with medically recommended therapy or visits.
- History of myocardial infarction with residual atrial, nodal, or ventricular arrhythmias that are not controlled with medical and/or surgical interventions. A recent (≤ 12 months) history of myocardial infarction with secondary arrhythmias is exclusionary regardless of the therapeutic control.
- Received within 4 weeks of Screening or planning to take during participation in the clinical study:
  - Any doses of a CR CD-LD apart from a single daily bedtime dose, any doses of Rytary, additional CD (eg, Lodosyn) or benserazide (eg, Serazide), or catechol-O-methyl transferase inhibitors (entacapone or tolcapone) or medications containing these inhibitors (Stalevo),
  - Nonselective monoamine oxidase inhibitors (MAOI), apomorphine, or antidopaminergic agents, including antiemetics.
- Subjects who have previously participated in an IPX203 study.

Investigational product, dosage and mode of administration: IPX203 (carbidopa-levodopa) Extended-Release capsules, containing 35-140 mg of CD-LD and matching placebo, for oral administration.

Reference therapy, dosage and mode of administration: Immediate-Release carbidopa-levodopa (IR CD-LD) tablets containing 25-100 mg of CD-LD, and matching placebo, for oral administration.

Duration of treatment: Approximately 24 weeks, including up to 4 weeks following Screening,

3 weeks of IR CD-LD dose adjustment, 4 weeks of IPX203 dose conversion, and 13 weeks of double-blind therapy following randomization.

Criteria for evaluation:

Baseline is defined as assessments done at Visit 4 (randomization visit). Study Entry is defined as assessments done at Visit 1 (study entry visit).

Efficacy:

- Primary endpoint: Change from baseline in “Good on” time in hours per day, averaged over the PD Diary days, at the end of double-blind treatment period (Visit 7 or early termination). “Good on” time is derived from the 3-day PD Diaries and is defined as the sum of “On” time without dyskinesia and “On” time with nontroublesome dyskinesia.
- Key secondary endpoints:
  - Change from baseline in “Off” time in hours per day, averaged over the PD Diary days at the end of double-blind treatment period (Visit 7 or early termination)
  - Proportion of subjects with either “much improved” or “very much improved” in Patient Global Impression of Change (PGI-C) scores at the end of double-blind treatment period (Visit 7 or early termination)
  - Change from baseline in the MDS-UPDRS Part III at the end of double-blind treatment period (Visit 7 or early termination)
  - Change from baseline in the sum of MDS-UPDRS Parts II and III at the end of double-blind treatment period (Visit 7 or early termination)
- Additional endpoints:

  The following endpoints will be evaluated (at the post-randomization visits) as change from baseline (Visit 4) as well as change from the study entry (Visit 1), when applicable, by visits:
  - Percent “Off” time during waking hours derived from the 3-day PD Diaries
  - Average duration of each continuous “Good on” and each continuous “On”
  - Hours of (1) “Off” time (from Visit 1), (2) “Good on” time (from Visit 1), (3) “On” time with dyskinesia, (4) “On” time with troublesome dyskinesia, and (5) “On” time with nontroublesome dyskinesia, and (6) asleep time derived from the 3-day PD Diaries
  - Proportion of subjects with an improvement in “Good on” time of at least 1, 1.5, 2, 2.5, and 3 hours
  - Proportion of subjects with a reduction in “Off” time of at least 0.5, 1, 1.5, 2, 2.5, and 3 hours
  - Proportions of subjects who are “On” upon awakening and “Good on” upon awakening
  - Average time to “On” upon awakening
  - Change from baseline in the average number of motor fluctuations per day averaged over the PD Diary days. A motor fluctuation is defined as a change from “Off” to “On” state or from “On” to “Off” state.
  - MDS-UPDRS total score (sum of Parts I, II, III, and IV) and Parts I, II, and IV separately
  - MDS-UPDRS Part III and Parts II + III combined (from Visit 1)
  - MDS-UPDRS Part II Question 2.9

- PDQ-39 total score and individual domain scores
- NMSS total score and individual domains
- PDSS-2 total score and individual domains
- PDSS-2 items 9, 10, 11, 12, and 13 combined
- PAS total score and individual domains
- PGI-S
- Proportion of subjects with either "severely ill" or "extremely severely ill" on the PGI-S
- CGI-S
- Proportion of subjects with either "severely ill" or "among the most extremely ill of subjects" on the CGI-S
- PGI-C scores
- CGI-C scores
- Proportion of subjects with either "much improved" or "very much improved" on the CGI-C

- **Safety:** electrocardiograms (ECGs), clinical laboratory tests, physical examinations, Columbia-Suicide Severity Rating Scale (C-SSRS), and supine and standing orthostatic vital signs; adverse events and concomitant medications evaluated throughout the course of the study.

Statistical methods: For the primary endpoint, assuming a difference of 1 hour between IPX203 and IR CD-LD in "Good on" time and a standard deviation of the treatment difference to be 3.0 hours, a sample size of 210 per arm will be needed to ensure at least 90% power at a 0.05 significance level. Assuming approximately an 18% prerandomization drop-out, approximately 510 subjects would need to be enrolled to randomize 420 subjects.

The primary efficacy endpoint of change from baseline in "Good on" time will be analyzed using a mixed model for repeated measures (MMRM) model. The model will include baseline (Visit 4) "Good on" time as a covariate, treatment, visit, and pooled center as fixed effects, and a treatment-by-visit interaction. The model will employ an unstructured within subject covariance matrix and a restricted maximum likelihood (ReML) estimation method. The degree-of-freedom of the denominator will be estimated using the Kenward-Roger method.

The key secondary endpoints (change from baseline in "Off" time, change from baseline in MDS-UPDRS Part III, and change from baseline in the sum of the MDS-UPDRS Parts II and III) will be analyzed using MMRM models similar to the primary analysis model. The proportion of subjects with either "much improved" or "very much improved" on the PGI-C will be analyzed using Cochran-Mantel-Haenszel test to compare the two treatment groups with pooled center as a stratification factor.

In order to control the type I error rate, the primary and key secondary endpoints will be tested in a single hierarchical order: (1) change from baseline in "Good on" time, (2) change from baseline in "Off" time, (3) proportion of subjects with either "much improved" or "very much improved" in PGI-C, (4) change from baseline in the MDS-UPDRS Part III, (5) change from baseline in the sum of MDS-UPDRS Parts II and III.

Quantitative safety data will be summarized using descriptive statistics and frequency distributions. Qualitative safety data will be summarized by frequencies and percentages. All summaries will be presented by treatment arms.

2. TABLE OF CONTENTS, LIST OF TABLES, AND LIST OF FIGURES

TABLE OF CONTENTS

1. SYNOPSIS 5
2. TABLE OF CONTENTS, LIST OF TABLES, AND LIST OF FIGURES 10
3. LIST OF ABBREVIATIONS AND DEFINITIONS OF TERMS 16
4. INTRODUCTION 19
5. TRIAL OBJECTIVES 23
6. INVESTIGATIONAL PLAN 24
6.1. Overall Study Design 24
6.2. Number of Subjects 25
6.3. Treatment Assignment 26
6.4. Dosing and Dose Determination Criteria 26
6.4.1. IR CD-LD Dose Adjustment Period 26
6.4.2. IPX203 Dose Conversion Period 26
6.4.3. Double-Blind Maintenance Period 28
6.5. Criteria for Study Termination 28
7. SELECTION AND WITHDRAWAL OF SUBJECTS 29
7.1. Subject Inclusion Criteria 29
7.2. Subject Exclusion Criteria 30
7.3. Subject Withdrawal Criteria 31
8. STUDY PROCEDURES 33
8.1. Screening Visit 37
8.2. Visit 1 – Start of IR CD-LD Dose Adjustment 38
8.2.1. Prior to Visit 1 38
8.2.2. At Visit 1 38
8.2.3. Post Visit 1 39
8.3. Visits 2 (Week 3) and Visit 3 (Week 5) – IPX203 Dose Conversion 39
8.3.1. Prior to Visit 2 39
8.3.2. Prior to Visit 3 39
8.3.3. At Visits 2 and 3 39

8.3.4. Post Visits 2 and 3 40
8.4. Visit 4 (Week 7) – Randomization 40
8.4.1. Prior to Visit 4 40
8.4.2. At Visit 4....40
8.5. Visit 5 (Week 10) and Visit 6 (Week 15) 41
8.5.1. Prior to Visit 5 and 6....41
8.5.2. At Visit 5 and 6....41
8.6. Visit 7 (Week 20) – End of Study/Study Exit 42
8.6.1. Prior to Visit 7 42
8.6.2. At Visit 7....42
8.7. Early Termination....43
8.7.1. Subjects Who Terminate Prior to Randomization....43
8.7.2. Subjects Who Terminate Early after Randomization 43
8.8. Blood Volume....44
9. TREATMENT OF SUBJECTS....45
9.1. Description of Study Drug....45
9.2. Concomitant Medications....45
9.2.1. Permitted PD Medications....45
9.2.2. Prohibited Medications and Procedures 45
9.2.3. Rescue Medications....46
9.3. Treatment Compliance....46
9.4. Randomization and Blinding 46
10. STUDY DRUG MATERIALS AND MANAGEMENT 47
10.1. Study Drug....47
10.2. Study Drug Packaging and Labeling 47
10.3. Study Drug Storage....47
10.4. Study Drug Administration....48
10.5. Study Drug Dispensing and Accountability 48
10.6. Study Drug Handling and Disposal 48
11. ASSESSMENT OF EFFICACY 49
11.1. Parkinson’s Disease Diary....49
11.2. Patient and Investigator Global Assessments....49

11.3. Movement Disorders Society Version of Unified Parkinson's Disease Rating Scale 49
11.4. Additional Assessments 50
12. ASSESSMENT OF SAFETY 51
12.1. Safety Parameters 51
12.2. Adverse Events 51
12.2.1. Definition of Adverse Event 51
12.2.2. Recording Adverse Events 51
12.2.3. Follow-up 52
12.2.4. Relationship to Study Drug 52
12.2.5. Assessment of Severity 52
12.3. Serious Adverse Events 52
12.3.1. Definition of Serious Adverse Event 52
12.3.2. Reporting Serious Adverse Events 53
12.4. Pregnancy 53
12.5. Other Safety Parameters and Related Information 54
13. STATISTICS 55
13.1. Study Design and Sample Size Estimation 55
13.2. Demographics/Baseline Comparability 55
13.3. Efficacy Endpoints 55
13.4. Analysis of Efficacy Data 57
13.4.1. Primary Efficacy Endpoint 57
13.4.2. Key Secondary Efficacy Endpoints 57
13.4.3. Additional Efficacy Endpoints 58
13.5. Center Pooling Algorithm 58
13.6. Sensitivity Analyses of the Primary Endpoint and Key Secondary Endpoints 59
13.6.1. Assessing Assumptions of the Mixed Model for Repeated Measures (MMRM) 59
13.6.2. Complete Case Analysis 59
13.6.3. Single LOCF/BLOCF Imputation 59
13.6.4. Pattern-Mixture Model 60
13.7. Subgroup Analyses 61
13.8. Multiplicity Adjustments 61

13.9. Analysis Populations 62
13.9.1. Safety Analysis Set 62
13.9.2. Intent-to-Treat Analysis Set 62
13.9.3. Modified Intent-to-Treat Analysis Set 62
13.9.4. Completers Analysis Set 62
13.10. Handling of Missing Data 62
13.10.1. Missing Data for PD Diaries 62
13.10.2. Missing Data for Global Assessments (PGI-C, CGI-C, PGI-S and CGI-S) 63
13.10.3. Missing Data for MDS-UPDRS 63
13.11. Analysis of Safety 64
14. ADMINISTRATIVE PROCEDURES 65
14.1. Guidelines for Good Clinical Practice 65
14.2. Institutional Review Board Approval 65
14.3. Informed Consent 65
14.4. Study Monitoring 65
14.5. Protocol Amendments 65
14.6. Termination of Study 66
14.7. Case Report Forms 66
14.8. Investigator's Final Conduct Report 66
14.9. Records Retention 66
15. PUBLICATION POLICY 67
16. LIST OF REFERENCES 68
17. APPENDICES 70
APPENDIX A. PRESCRIBING INFORMATION FOR IR CD-LD 70
APPENDIX B. INSTRUCTIONS FOR DOSE CONVERSION TO IPX203 81
APPENDIX C. UNITED KINGDOM PARKINSON'S DISEASE SOCIETY BRAIN BANK DIAGNOSTIC CRITERIA FOR PARKINSON'S DISEASE 83
APPENDIX D. MONTREAL COGNITIVE ASSESSMENT (MOCA) 84
APPENDIX E. MOVEMENT DISORDERS SOCIETY VERSION OF UNIFIED PARKINSON'S DISEASE RATING SCALE (MDS-UPDRS) 90
APPENDIX F. PATIENT GLOBAL IMPRESSION OF CHANGE (PGI-C) 123
APPENDIX G. CLINICAL GLOBAL IMPRESSION OF CHANGE (CGI-C) 124
APPENDIX H. PATIENT GLOBAL IMPRESSION OF SEVERITY (PGI-S) 125

APPENDIX I. CLINICAL GLOBAL IMPRESSION OF SEVERITY (CGI-S)......................126
APPENDIX J. 39-ITEM PARKINSON'S DISEASE QUESTIONNAIRE (PDQ-39) 127
APPENDIX K. GASTROPARESIS CARDINAL SYMPTOM INDEX (GCSI).....................131
APPENDIX L. NON-MOTOR SYMPTOM ASSESSMENT SCALE FOR PARKINSON'S DISEASE (NMSS)..133
APPENDIX M. PARKINSON'S DISEASE SLEEP SCALE-2 (PDSS-2) 136
APPENDIX N. PARKINSON ANXIETY SCALE (PAS)...137
APPENDIX O. COLUMBIA-SUICIDE SEVERITY RATING SCALE (C-SSRS)................141
APPENDIX P. PARKINSON'S DISEASE DIARY 148
APPENDIX Q. EXCIPIENTS IN IPX203, IPX203 PLACEBO, IR CD-LD, AND IR PLACEBO 149
APPENDIX R. CLINICAL LABORATORY STUDIES 150

LIST OF TABLES

Table 1: Abbreviations and Specialist Terms 16

Table 2: LD Dosage in Study IPX203-B14-02 20

Table 3: Recommended Starting IPX203 LD Dosing Regimen Based on the Dosing Regimen of IR CD-LD at the End of the Dose Adjustment Period 27

Table 4: Events Schedule for Impax Study IPX203-B16-02 34

Table 5: Study Drugs for Study IPX203-B16-02 45

LIST OF FIGURES

Figure 1: Study Flow Chart 25

3. LIST OF ABBREVIATIONS AND DEFINITIONS OF TERMS

The following abbreviations and specialist terms are used in this study protocol.

Table 1: Abbreviations and Specialist Terms

Abbreviation or Specialist Term	Explanation
AADC	aromatic amino acid decarboxylase
ADL	activities of daily living
AE	adverse event
ANCOVA	analysis of covariance
ANOVA	analysis of variance
BLOCF	baseline observation carried forward
BMI	body mass index
CD	carbidopa
CGI-C	Clinical Global Impression of Change
CGI-S	Clinical Global Impression of Severity
CR	controlled release
CRF	case report form
C-SSRS	Columbia-Suicide Severity Rating Scale
ECG	electrocardiogram
ER	extended release
FDA	Food and Drug Administration
GCP	Good Clinical Practice
GCSI	Gastroparesis Cardinal Symptom Index
HIPAA	Health Insurance Portability and Accountability Act
ICF	informed consent form
ICH	International Conference on Harmonization
IEC	independent ethics committee

Abbreviation or Specialist Term	Explanation
IR	immediate release
IRB	institutional review board
IWRS	interactive web response system
LD	levodopa
LOCF	last observation carried forward
MAOI	monoamine oxidase inhibitors
MAR	missing at random
M-EDL	Motor Aspects of Experiences of Daily Living
MedDRA	Medical Dictionary for Regulatory Activities
MDS-UPDRS	Movement Disorders Society version of the Unified Parkinson's Disease Rating Scale
mITT	modified intent-to-treat
MMRM	mixed model for repeated measures
MNAR	missing not at random
MoCA	Montreal Cognitive Assessment
nM-EDL	Non-Motor Aspects of Experiences of Daily Living
NMSS	Non-Motor Symptom Assessment Scale
PAS	Parkinson Anxiety Scale
PD	Parkinson's disease
PDQ-39	39-item Parkinson's Disease Questionnaire
PDSS-2	Parkinson's Disease Sleep Scale-2
PGI-C	Patient Global Impression of Change
PGI-S	Patient Global Impression of Severity
PK	pharmacokinetic (adjective)<br>pharmacokinetics (singular noun)
PI	principal investigator

Abbreviation or Specialist Term	Explanation
PMM	pattern-mixture models
ReML	restricted maximum likelihood
SAE	serious adverse event
SAP	statistical analysis plan
SD	standard deviation
TEAE	treatment-emergent adverse event
US	United States

4. INTRODUCTION

Parkinson's disease (PD) is a progressive neurodegenerative disorder of the extrapyramidal nervous system. Levodopa (LD) used in combination with carbidopa (CD) is considered the gold standard for the symptomatic treatment of PD. LD is a dopamine precursor converted to dopamine by aromatic amino acid decarboxylase (AADC). Carbidopa is an AADC inhibitor that does not cross the blood-brain barrier. When used in combination with LD, CD increases the plasma half-life of LD from 50 minutes to 1.5 hours. Carbidopa inhibits the conversion of LD into dopamine in the periphery, thereby reducing the peripheral side-effects caused by dopamine and increasing the amount of LD available for transport into the brain. The administration of CD with LD reduces the dose of LD required to produce a dopaminergic response by about 75 percent (Sinemet prescribing information; Appendix A).

Due to its proven efficacy, LD is prescribed eventually to most subjects with PD. However, long-term use of LD is associated with certain complications, including "wearing-off" or "end-of-dose effect," where symptom control decreases causing the drug effects to wear off sooner. As the disease progresses further, motor complications, namely dyskinesias and motor "On/Off" fluctuations, develop in about 50% of the patients after 5 years of treatment (Fahn 1999). Such motor complications can be a significant source of disability and their management is a major unmet need in the treatment of PD.

Mechanisms underlying motor complications involving dyskinesias and "On/Off" fluctuations in PD are unclear. The pulsatile nature of standard orally administered LD is thought to contribute to the appearance of motor complications. Chronic intermittent pulsatile stimulation of the dopamine receptors that are under tonic control contributes to the development of dyskinesia in PD animal models as compared to animals treated with continuous infusion (Juncos et al 1989, Engber et al 1989, Blanchet et al 1995). In addition, unreliable absorption of LD potentially due to erratic gastric empting and variable in vivo dissolution of LD products is thought to contribute to the delay or inadequate response after oral dosing with standard CD-LD products (Melamed et al 1986, Kurlan et al 1988, Stocchi et al 1994). These findings suggest that motor complications in patients with PD may be less likely to develop with continuous dopaminergic stimulation.

Intraduodenal infusion of LD has been shown to significantly reduce motor complications and to reduce "Off" time. The findings of infusion studies in PD patients indicate that the maintenance of stable plasma LD concentrations and the avoidance of low trough levels are effective in reducing "Off" hours, increasing "On" hours without disabling dyskinesia, and reducing the severity of dyskinesia versus standard oral LD formulations (Mizuno 2007, Nilsson et al 2001, Nyholm et al 2005, Stocchi et al 2005). These findings provide a strong rationale for the development of an extended-release (ER) oral dosage form that delivers a constant LD plasma concentration in order to optimize relief of PD symptoms, and to minimize "Off" time and dyskinesia.

IPX203 is an investigational product containing CD-LD that is being developed by Impax Laboratories, Inc. through its Impax Specialty Pharma division (Impax). The primary objective of the IPX203 program is to develop an extended-release product that can attain therapeutic LD

plasma concentrations rapidly and maintain constant LD plasma concentrations for a longer duration than currently approved products with minimal peak-to-trough fluctuations. IPX203 is designed to be dosed approximately every 8 hours.

Impax characterized the PK and pharmacodynamics of IPX203 in Study IPX203-B14-02, a single dose trial in subjects with advanced PD versus IR CD-LD and Rytary (carbidopa and levodopa) extended-release capsules. Twenty-six (26) subjects were randomized with 25 subjects completing all 3 treatments. One subject discontinued study early due to subject withdrawal. The doses of IPX203 and Rytary were determined on the basis of each subject's prestudy baseline morning dose of IR CD-LD (Table 2).

Table 2: LD Dosage in Study IPX203-B14-02

Prestudy Baseline Morning IR LD (mg)	IR LD (mg)	Rytary LD (mg)	IPX203 LD (mg)
100	100	340	360
150	150	485	540
200	200	630	720
250	250	780	810

Administration of IPX203 yielded an initial increase in LD plasma concentrations that was similar to IR CD-LD but maintained LD concentrations for a longer duration than either IR CD-LD or Rytary. The bioavailability of LD (based on AUC_{∞}) from IPX203 was 88% relative to IR CD-LD and about 11% more than Rytary. Plasma exposure to LD (C_{max} and AUC_{∞}) following IPX203 increased in an approximately dose-proportional manner. Pharmacodynamic effects as measured by change from baseline scores on MDS-UPDRS Part III were consistent with the PK profiles of LD. Following IPX203 treatment, decrements in the Part III total score (reflecting improvements in motor symptoms) lasted for a longer duration than either IR CD-LD or Rytary. IPX203 provided a longer duration of effect compared with IR CD-LD and Rytary, including "Off" time and "Good on" time based on the Assessment of Subject's Motor State and on a range of improvement thresholds of the MDS-UPDRS Part III. The results were consistent with the prolonged LD plasma concentration profile with IPX203 compared to IR CD-LD or Rytary and support a dosing interval of approximately 8 hours.

Of the 26 subjects who received at least one of the 3 treatments, 9 (34.6%) reported at least one treatment-emergent adverse event (AE). None of these subjects reported a serious AE (SAE) nor did any subjects prematurely discontinue the study because of an AE. Adverse events were reported by more subjects during IR (28.0%) and IPX203 (19.2%) than during Rytary (8.0%) treatment. None of the reported AEs were classified as "severe." Adverse events reported by 2 or more subjects include: Dizziness (3 subjects), nausea (2 subjects), and hypertension (2 subjects). The numbers of subjects reporting these AEs during any single treatment period were small (0 to 2 subjects). Two subjects reported dizziness during IR CD-LD treatment and one subject each during IPX203 and Rytary treatments. Hypertension was reported by a total of 2 subjects, both reporting this AE during IPX203 and IR CD-LD treatments and 1 subject during Rytary treatment. Two subjects reported nausea only during the IR CD-LD treatment period. Of the 9 subjects reporting AEs, 6/26 (23.1%) of subjects reported AEs that were assessed as related to treatment, including all of the reports of dizziness, nausea, and dyskinesia (1 subject).

Study IPX203-B16-01 is a randomized, open-label, rater-blinded, multicenter, 2-treatment, 2-period, multiple-dose crossover study that has completed dosing. Twenty-eight (N=28) advanced PD subjects were randomized to 1 of 2 dosing sequences, with each treatment period lasting 15 days and separated by a 1-week wash-out period where subjects return to their usual stable pre-study CD-LD regimen. The objectives of this study are to compare the PK, pharmacodynamics, efficacy, and safety of IPX203 with IR CD-LD after single and multiple dosing. Subjects were permitted to take allowed non-CD-LD based PD medications throughout the study if dosing regimens had been stable for at least 4 weeks. Subjects were instructed to take their last dose of CD-LD no later than 10:00 PM on the evening prior to Day 1 of each treatment period and to withhold dosing for at least 5 hours before arriving at the site on Day 15 of each treatment period. On Day 1 of the IR CD-LD treatment period, subjects were started with a single dose of their usual prestudy first morning IR CD-LD dose. On Day 1 of the IPX203 treatment period, subjects were started with a single dose of IPX203 based on their usual prestudy first morning IR CD-LD dose using a LD conversion of 100 mg IR LD to 360 mg of IPX203 LD. During the IR CD-LD treatment period, the initial dosing regimen of IR CD-LD was the same as the subject's stable prestudy regimen. During the IPX203 treatment period, the IPX203 regimen for subsequent doses for the day was determined by identifying the most frequent prestudy IR LD dose in milligrams that the subject received in the afternoon and evening and administering IPX203 using a LD conversion of 100 mg IR LD to 270 mg of IPX203 LD. The protocol recommended that IPX203 be dosed approximately every 7 to 8 hours. During Days 1 through 9 of both treatment periods, investigators had the opportunity to adjust each subject's study medication regimen if necessary to optimize efficacy and safety. Pharmacokinetics and pharmacodynamics (MDS-UPDRS Part III and Assessments of Subject's Motor State) were periodically evaluated on Day 1 and Day 15 of each treatment period by qualified clinical staff who were blinded to dosing.

Data from this multiple-dose study confirmed the PK and pharmacodynamic results observed in the single dose study with IPX203:

- PK data from 27 subjects indicates IPX203 shows a rapid increase in LD concentrations followed by extended-release characteristics. Following IPX203; initial increases in LD concentrations were comparable to that from IR CD-LD. Bioavailability of LD following IPX203 was ~89% relative to IR CD-LD. LD plasma concentrations were sustained longer after IPX203 treatment than after IR CD-LD and support dosing every 8 hours. No accumulation of LD was evident at steady-state following IPX203 or IR CD-LD. Plasma LD concentrations following IPX203 were characterized by lower peak-to-trough fluctuation. No time-variant or time-dependent changes were noted in PK of CD or LD following IPX203.
- IPX203 demonstrated an onset of effect that was comparable to IR CD-LD in MDS-UPDRS Part III scores. IPX203 prolonged the duration over which MDS-UPDRS Part III scores were improved by prespecified threshold changes from baseline (≥4, ≥7, and ≥13 units).
- IPX203 provides a significant decrease in "Off" time and a significant increase in "Good on" time compared to IR CD-LD treatment on Day 1 and Day 15 when assessed by the Investigator's Assessment of Subject's Motor State. Subjects treated

with IPX203 did not experience a significant increase in “On” time with troublesome dyskinesia compared to IR CD-LD.

- Subjects achieved significant improvements in “Off” time, “Good on” time, and frequency of motor state fluctuations based on the 3-day PD Diaries.
- Twenty-eight subjects were enrolled in the multiple dose study and 27 subjects completed both treatments. Safety results were as follows:
  - One subject discontinued during the IPX203 treatment period due to an AE (orthostatic hypertension) that was considered possibly related to treatment.
  - A total of 39.3% (11/28) of treated subjects reported at least one treatment emergent AE, including 35.7% (10/28) during IPX203 treatment and 7.4% (2/27) during IR CD-LD treatment. Eight subjects reported AEs that were related to treatment (8 subjects during IPX203 treatment and 1 during IR CD-LD treatment).
  - Two subjects experienced serious adverse events (SAEs). One subject reported increased hypertension of mild severity during IPX203 treatment that was considered unrelated to treatment and resolved. A second subject reported moderate to severe dehydration, diarrhea, and atrial fibrillation during the washout period that were considered unrelated to treatment and resolved.
  - AEs reported in 2 or more subjects included nausea (2), dizziness (2), and dyskinesia (5), all of mild or moderate severity, and all during the IPX203 treatment.

The current protocol, Study IPX203-B16-02, is a multicenter, randomized, double-blind, double-dummy, active-controlled, parallel-group, Phase 3 study. It is designed to compare the efficacy, safety and tolerability of IPX203 with IR CD-LD following multiple doses over 13 weeks. The following IPX203 dosing guidelines will be utilized in the present study (IPX203-B16-02):

- The initial regimen of IPX203 is based on the most frequent dose of the subject’s dosing regimen of IR CD-LD at the end of dose adjustment period (Visit 2);
- A 25-100 mg dose of IR CD-LD will be converted to a 70-280 mg CD-LD dose of IPX203;
- IPX203 will be administered approximately every 8 hours for most subjects;
- Investigators may adjust the IPX203 regimen during the dose conversion period to optimize the therapeutic effect (minimize “Off” time without causing troublesome dyskinesia or other dopaminergic side effects).

The proposed dose conversion scheme for this study has been developed based on a similar dose conversion from IR CD-LD to IPX203 that was studied in the completed Phase 2a study (IPX203-B14-02, n=25) and the Phase 2b study (IPX203-B16-01, n=28), both conducted in subjects with advanced PD using similar entry criteria to the present study. The doses of IPX203 are expected to be comparable to other ER CD-LD products, such as Rytary and Duopa.

5. TRIAL OBJECTIVES

To evaluate the safety and efficacy of IPX203 in comparison to IR CD-LD in the treatment of CD-LD experienced subjects with Parkinson's disease (PD) who have motor fluctuations.

6. INVESTIGATIONAL PLAN

6.1. Overall Study Design

This is a multicenter, randomized, double-blind, double-dummy, active-controlled, parallel-group study. Subjects will continue to take permitted non-CD-LD-based PD medications throughout the study if documented in their prestudy regimen and if dosing regimens have been stable for at least 4 weeks prior to Visit 1. A "stable dosing regimen" means no change in dose or in dosing frequency. Within 4 weeks following the Screening visit, eligible subjects will complete their PD Diaries on each of the 3 consecutive days immediately prior to Visit 1.

Following Visit 1, qualified subjects will enter a 3-week, open-label IR CD-LD treatment period allowing for dose adjustment. During the IR CD-LD dose adjustment period, the initial dosing regimen of IR CD-LD will be the same as the subject's stable prestudy regimen unless the subject is taking a single daily bedtime dose of CR CD-LD, in which case, the CR CD-LD dose will be discontinued and substituted with a 1:1 milligram-equivalent dose of IR CD-LD. A "bedtime dose" is defined as the last daytime dose of CD-LD taken within 1 hour of onset of the subject's normal nighttime sleep period. **The dosing regimen of IR CD-LD may be adjusted during the dose adjustment period** to minimize "Off" time without causing troublesome dyskinesia. The doses and regimens of the subject's other non-CD-LD Parkinson's disease medications (dopamine agonists, MAO-B inhibitors, amantadine, anticholinergics) should remain stable throughout this study. Any adjustments to the IR CD-LD dosing regimen will be done in consultation with the Investigator or qualified site personnel and will be recorded. The subject must be on a stable dosing regimen (no change in dose or in dosing frequency) of IR CD-LD for at least 5 days prior to returning for Visit 2. Rescue with additional or modified doses of concomitant PD medications or with use of CD-LD products other than the dispensed study medications is not permitted and will trigger discontinuation from the study. Subjects will complete their 3-day PD Diaries on each of the 3 consecutive days immediately prior to Visit 1.

Following completion of the IR CD-LD dose adjustment period, subjects will begin a 4-week open-label period for conversion to IPX203. The initial dosing regimen of IPX203 during the conversion period will be based on the subject's dosing regimen of IR CD-LD at the end of the dose adjustment period (Visit 2) selecting the most frequent dose according to Table 3. A 25-100 mg dose of IR CD-LD converts to a 70-280 mg CD-LD dose of IPX203 but with a longer duration of effect. It is recommended that IPX203 should be dosed approximately every 8 hours with the exception that subjects who are currently receiving a total daily dose of less than 125-500 mg IR CD-LD at the end of the dose adjustment period will be initially administered IPX203 every 12 hours. The dosing interval may be reduced to approximately every 8 hours if the subject does not achieve an acceptable duration of effect. **The dosing regimen of IPX203 may be adjusted during the dose conversion period** to achieve the optimal balance of efficacy and tolerability (minimize "Off" time without causing troublesome dyskinesia or other dopaminergic side effects). Any adjustments to the IPX203 dosing regimen will be recorded. Rescue with additional or modified doses of concomitant PD medications or with use of CD-LD products other than the dispensed study medications is not permitted and will trigger discontinuation from the study. Subjects will return to the clinic in two weeks for Visit 3 followed by Visit 4 two weeks later. The subject must be on a stable dosing regimen of IPX203

(no change in dose or in dosing frequency) for at least 5 days prior to returning for Visit 4. Subjects will also be instructed to complete their 3-day PD Diaries on each of the 3 consecutive days immediately prior to Visit 4.

The study staff will call the subjects frequently (approximately every 1 to 3 days) during the IR CD-LD dose adjustment and IPX203 dose conversion periods. The calls are to ensure timely and appropriate dosing adjustments and to ensure that the subject is able to follow and adhere to the dosing instructions. The contacts may be less frequent after initial dose adjustments have been made. Any changes in the dosing regimen will be in consultation with the Investigator or qualified site personnel and will be documented.

Subjects who successfully complete the IPX203 dose conversion period will be randomized, stratified by center, in a 1:1 ratio at Visit 4 into one of two parallel treatment arms of IPX203 (with matching IR CD-LD placebo) or IR CD-LD (with matching IPX203 placebo). The subjects will undergo 13 weeks of double-blind, double-dummy maintenance therapy with the stable dosing regimen established at the end of Week 3 (Visit 2) for IR CD-LD and at the end of Week 7 (Visit 4) for IPX203. Subjects will return to the clinic for 3 visits (Visits 5, 6, and 7) and will be instructed to complete their 3-day PD Diaries on 3 consecutive days immediately prior to each of the 3 visits. Rescue with additional or modified doses of concomitant PD medications or use of CD-LD products other than the dispensed study medication is not permitted and will trigger discontinuation from the study.

Figure 1: Study Flow Chart

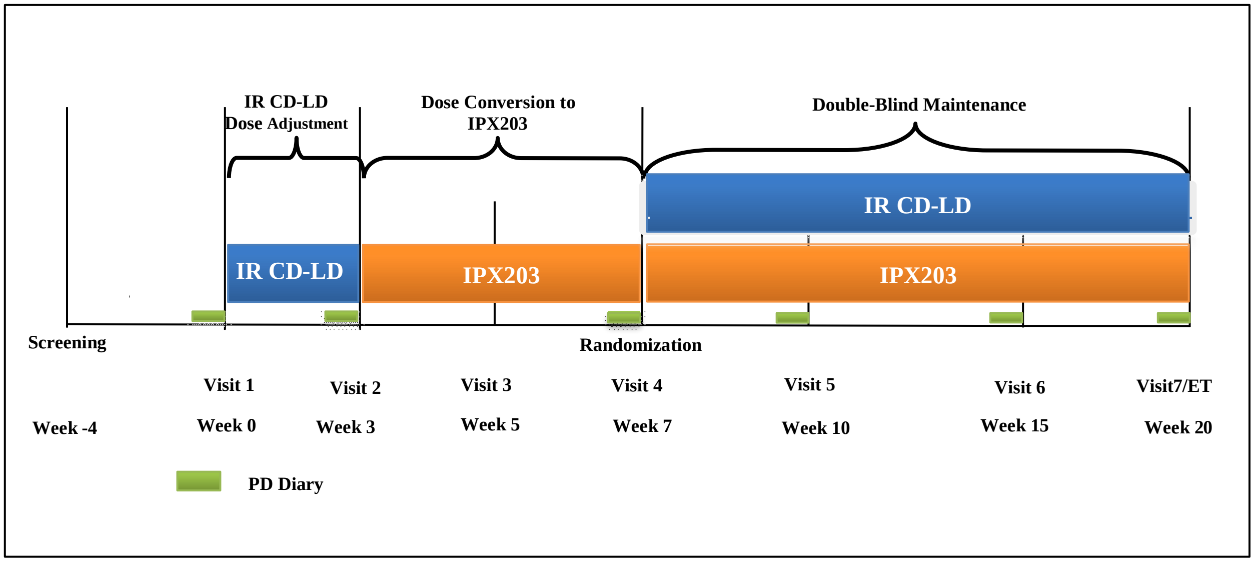

Abbreviations: IR=immediate-release, CD=carbidopa, LD=levodopa, ET=early termination

6.2. Number of Subjects

Assuming approximately an 18% prerandomization drop-out, approximately 510 subjects will be enrolled to randomize 420 subjects.

6.3. Treatment Assignment

Investigational product: IPX203 (carbidopa-levodopa) Extended-Release Capsules containing 35-140 mg of CD-LD, for oral administration.

Reference therapy: Immediate-Release carbidopa-levodopa (IR CD-LD) tablets containing 25-100 mg of CD-LD, for oral administration.

Subjects will be randomly assigned to one of two parallel treatment arms to receive either IPX203 (with matching IR CD-LD placebo) or IR CD-LD (with matching IPX203 placebo) during the double-blind maintenance therapy portion of the study.

6.4. Dosing and Dose Determination Criteria

IR CD-LD will be supplied as tablets containing 25-100 mg of CD-LD. IR CD-LD tablets may be split to achieve the required dose.

IPX203 will be supplied as capsules containing 35-140 mg of CD-LD. The suggested doses and regimen of IPX203 are intended to provide an onset of effect comparable to the subject's prestudy IR LD regimen and to extend the duration of effect. A 25-100 mg dose of IR CD-LD converts to a 70-280 mg CD-LD dose of IPX203.

6.4.1. IR CD-LD Dose Adjustment Period

During the 3-week IR CD-LD dose adjustment period, the initial dosing regimen of IR CD-LD will be the same as the subject's stable prestudy regimen unless the subject is taking a single daily bedtime dose of CR CD-LD, in which case, the CR CD-LD dose will be discontinued and substituted with a 1:1 milligram-equivalent dose of IR CD-LD. A "bedtime dose" is defined as the last daytime dose of CD-LD taken within 1 hour of onset of the subject's normal nighttime sleep period. Subjects who were receiving IR CD-LD as a 1:10 CD-LD formulation will be started on IR CD-LD with a 1:4 ratio at the same frequency and LD dose. **The dosing regimen of IR CD-LD may be adjusted during the dose adjustment period** to minimize "Off" time without causing troublesome dyskinesia. The doses and regimens of the subject's other non-CD-LD Parkinson's disease medications (dopamine agonists, MAO-B inhibitors, amantadine, anticholinergics) should remain stable throughout this study. Any adjustments to the IR CD-LD dosing regimen will be done in consultation with the Investigator or qualified site personnel and will be recorded. The subject must be on a stable dosing regimen (no change in dose or in dosing frequency) of IR CD-LD for at least 5 days prior to returning for Visit 2.

6.4.2. IPX203 Dose Conversion Period

During the 4-week IPX203 dose conversion period, the suggested initial dosing regimen of IPX203 will be based on the most frequent dose of the subject's dosing regimen of IR CD-LD at the end of the dose adjustment period (Visit 2). A 25-100 mg dose of IR CD-LD converts to a 70-280 mg CD-LD dose of IPX203, and a half tablet (12.5-50 mg dose of IR CD-LD) converts to a 35-140 mg CD-LD dose of IPX203. To facilitate conversion of subjects from IR CD-LD to IPX203, Table 3 presents recommended starting dose regimens. It is recommended that IPX203 should be dosed approximately every 8 hours with the exception that subjects who are currently receiving a total daily dose of less than 125-500 mg IR CD-LD at the end of the dose adjustment

period will be initially administered IPX203 every 12 hours. The dosing interval may be reduced to approximately every 8 hours if the subject does not achieve an acceptable duration of effect. **The dosing regimen of IPX203 may be adjusted during the dose conversion period** to achieve the optimal balance of efficacy and tolerability (minimize "Off" time without causing troublesome dyskinesia or other dopaminergic side effects). The maximum recommended daily dose of IPX203 is 600-2400 mg CD-LD. The doses and regimens of the subject's other non-CD-LD Parkinson's disease medications (dopamine agonists, MAO-B inhibitors, amantadine, anticholinergics) should remain stable throughout this study. The subject must be on a stable dosing regimen of IPX203 (no change in dose or in dosing frequency) for at least 5 days prior to returning for Visit 4.

Table 3: Recommended Starting IPX203 LD Dosing Regimen Based on the Dosing Regimen of IR CD-LD at the End of the Dose Adjustment Period

Most Frequent IR CD-LD Unit Dose (mg)	Recommended Starting IPX203 Daily Dosing Regimen CD-LD (mg) Every 8 Hours
25-100[a]	70-280 mg (2 × 35-140 mg)
>25-100 – 37.5-150	105-420 mg (3 × 35-140 mg)
>37.5-150 – 50-200	140-560 mg (4 × 35-140 mg)
>50-200	175-700 mg (5 × 35-140 mg)

[a] Subjects who are on a total daily dose of less than 125-500 mg CD-LD from IR CD-LD should be advised to initially take IPX203 every 12 hours. The dosing interval may be reduced to approximately every 8 hours if the subject does not achieve an acceptable duration of effect.

During the dose conversion to IPX203, the Investigator or site staff are advised to be in frequent contact (every 1 to 3 days) with the subject especially during the initial dose conversion to assess the need for dosage adjustment with the goal of minimizing "Off" time without causing troublesome dyskinesia. Any changes to the dosing regimen should only be made by the Investigator or qualified site personnel. If the subject experiences troublesome dyskinesias during initial dose conversion, consider reducing the dose by one capsule (35-140 mg IPX203 CD-LD) before increasing the dosing interval. If turning "On" is slow following the first morning dose, consider taking the morning dose in the fasted state and/or increasing the dose by one capsule (35-140 mg IPX203 CD-LD). If turning "On" is slow later in the day or to reduce "end-of-dose" "Off" time, consider increasing the dose by one capsule (35-140 mg IPX203 CD-LD).

When two or more IR CD-LD doses correspond to the most frequent IR CD-LD dose, the suggested IPX203 conversion should be based on the higher of the IR CD-LD doses.

A summary of the instructions for dose conversion to IPX203 is provided in Appendix B.

6.4.3. Double-Blind Maintenance Period

During the 13-week double-blind double-dummy maintenance period, subjects receive either IPX203 (with matching IR CD-LD placebo) or IR CD-LD (with matching IPX203 placebo) with the stable dosing regimen established at the end of Week 3 (Visit 2) for IR CD-LD and at the end of Week 7 (Visit 4) for IPX203.

6.5. Criteria for Study Termination

The Sponsor has the right to terminate this study and remove all study material from the study site at any time for medical or administrative reasons. The Sponsor will endeavor to give adequate notice to allow safe withdrawal of subjects from the study.

7. SELECTION AND WITHDRAWAL OF SUBJECTS

Each subject must meet all of the following inclusion and exclusion criteria to qualify for enrollment.

7.1. Subject Inclusion Criteria

1. Male or female subjects diagnosed at age ≥ 40 years with PD, consistent with the United Kingdom Parkinson's Disease Society Brain Bank Diagnostic Criteria (Appendix C) and who are being treated with stable regimens of CD-LD but experiencing motor fluctuations.
2. Able to provide written informed consent prior to the conduct of any study-specific procedures.
3. Female subjects of childbearing potential must have a negative urine pregnancy test at Screening Visit.
4. Negative urine screen for drugs of abuse and negative alcohol breath test at Screening.
5. Hoehn and Yahr Stages 1, 2, 3, or 4 in the "On" state (part of Movement Disorders Society version of the Unified Parkinson's Disease Rating Scale [MDS-UPDRS] Part III; Appendix E).
6. Agrees to use a medically acceptable method of contraception throughout the study and for 6 weeks after completing the study. Medically acceptable methods of contraception that may be used by the subject and/or partner include but are not limited to: abstinence, oral contraception, NuvaRing or transdermal systems, diaphragm with vaginal spermicide, intrauterine device, condom and partner using vaginal spermicide, surgical sterilization (6 months), progestin implant or injection, or postmenopausal female (no menstrual period for > 2 years) or vasectomy (> 6 months).
7. Montreal Cognitive Assessment (MoCA) score ≥ 24 at Screening Visit in "On" state (Appendix D).
8. By history, for the 4 weeks prior to Screening, the subject experiences daily "wearing-off" episodes with periods of bradykinesia in combination with at least one of rest tremor or rigidity, experiences an "Off" state upon awakening on most mornings, and reports an average of at least 2.5 cumulative hours per day of "Off" time during the waking hours.
9. Able to differentiate "On" state from "Off" state as determined by at least 75% concordance with a trained rater in "On/Off" ratings for 8 ratings over the 4-hour training period. The concordance must include at least 1 "On" and 1 "Off" rating and must be achieved within two 4-hour training sessions.
10. At Visit 1, review of the 3-day PD Diaries confirms the following: that the subject is able to properly complete the diaries with valid entries; and that the subject has an average of at least 2.5 hours per day of "Off" time during the waking hours over the 3 days with at least 1.5 hours of cumulative "Off" time on each day. Inability to properly complete the

diaries is indicated when more than 1 day of a diary is not returned or when more than 2 hours (4 half-hour periods) of one 24-hour diary day are missing (Appendix P).

11. Responsive to CD-LD therapy and currently being treated on a stable regimen with CD-LD for at least 4 weeks prior to Visit 1 and:
    a. Requires at least 100 mg of LD from IR CD-LD for the first morning dose
    b. Requires a total daily dose of at least 400 mg of LD and takes a maximum total daily dose of 2400 mg LD, from IR CD-LD alone or IR CD-LD in combination with a single daily bedtime dose of CR CD-LD
    c. Has a dosing frequency of 4 to 9 times daily of CD-LD
    d. By history, typically experiences an "On" response with the first dose of IR CD-LD of the day, but the efficacy of this dose typically lasts less than 4 hours.
12. At Screening, the subject has predictable "Off" periods defined by a score of 1 or 2 on Item #4.5 (Complexity of Motor Fluctuations) of the MDS-UPDRS Part IV B (Motor Fluctuations) (Appendix E).
13. At Screening, the MDS-UPDRS Part III total score in the "Off" state is at least 20 units.
14. Able and willing to comply with the protocol, including completion of diaries and availability for all study visits.

7.2. Subject Exclusion Criteria

1. Used any doses of controlled-release (CR) CD-LD apart from a single daily bedtime dose within 4 weeks prior to Visit 1.
2. Used any doses of Rytary for the past 4 weeks prior to Visit 1 or considered IPX066 or Rytary failures for reasons of efficacy or safety.
3. Received any investigational medications within 30 days or 5 times the half-life, whichever is longer, prior to Visit 1.
4. Female subjects who are currently breastfeeding or lactating.
5. Had prior neurosurgical treatment for PD or if such procedure is planned or anticipated during the study period.
6. Allergic to any excipient in the study drugs (See Appendix Q).
7. History of medical conditions or of a prior surgical procedure that would interfere with LD absorption, such as gastrectomy, proximal small-bowel resection, or bariatric surgery.
8. History of upper gastrointestinal hemorrhage in patients with peptic ulcer disease within the past 5 years.
9. History of glaucoma with intraocular pressures that are elevated despite appropriate medical management.
10. History of seizure or epilepsy and experienced at least 1 seizure during the past 12 months or has not been compliant with medically recommended therapy or visits.
11. History of myocardial infarction with residual atrial, nodal, or ventricular arrhythmias that are not controlled with medical and/or surgical interventions. A recent

(≤ 12 months) history of myocardial infarction with secondary arrhythmias is exclusionary regardless of the therapeutic control.

12. History of neuroleptic malignant syndrome or of nontraumatic rhabdomyolysis.
13. Liver enzyme values ≥ 2.5 times the upper limit of normal; or history of severe hepatic impairment.
14. Serum creatinine level ≥ 1.75 times the upper limit of normal; or requires dialysis at the time of Screening.
15. Subject with a history of malignant melanoma or with a suspicious undiagnosed skin lesion which in the opinion of the investigator could be melanoma.
16. History of drug or alcohol abuse within the 12 months prior to Screening.
17. Received within 4 weeks of Visit 1 or planning to take during participation in the clinical study:
    a. any doses of a controlled-release (CR) LD apart from a single daily bedtime dose, any doses of Rytary, additional CD (eg, Lodosyn) or benserazide (eg, Serazide), or catechol-O-methyl transferase inhibitors (entacapone or tolcapone) or medications containing these inhibitors (Stalevo),
    b. nonselective monoamine oxidase inhibitors (MAOI), apomorphine, or antidopaminergic agents, including antiemetics.
18. Treatment with any dopamine antagonist for the purposes of treating psychosis or bipolar disorder within the last 2 years or any history of psychosis within the past 10 years regardless of treatment. A brief, self-limited episode of psychosis precipitated by a medical intervention with return to normal mentation is not exclusionary. Mild PD-associated illusions are not exclusionary provided that they do not occur more than twice per week and the subject does not lose insight.
19. Employees or family members of the investigator, study site, or sponsor.
20. Subjects who have previously participated in an IPX203 study.
21. Subjects who, in the opinion of the clinical investigator, should not participate in the study.
22. Based on clinical assessment, subject does not adequately comprehend the terminology needed to complete the PD diary.

7.3. Subject Withdrawal Criteria

Site personnel should make every effort to conduct all protocol-specific procedures to complete the study. A subject may be discontinued from the study due to the following reasons:

1. Withdrawal by subject
2. Adverse event (AE)
3. Lack of efficacy
4. Study terminated by Sponsor

5. Protocol deviation
6. Noncompliance with study drug
7. Lost to follow-up
8. Death
9. Other

Subjects who withdraw early from the study will not be replaced. The reason or reasons for discontinuation will be specified and documented. Empty medication bottles and any unused study drug upon discontinuation will be collected. Study medication dispensed to a discontinued subject may not be redispensed to a different subject.

8. STUDY PROCEDURES

The procedures to be performed at each study visit are described below and summarized in Table 4.

Table 4: Events Schedule for Impax Study IPX203-B16-02

Assessment	Screening	3 Weeks of IR CD-LD Dose Adjustment	4 Weeks of IPX203 Dose Conversion			13 Weeks of Double-Blind Therapy		
		Visit 1	Visit 2	Visit 3	Visit 4 Randomization	Visit 5	Visit 6	Visit 7 / Study Exit/Early Termination[b]
Study Week[a]	**–4**	**0**	**3**	**5**	**7**	**10**	**15**	**20**
ICF & HIPAA Authorization[c]	X							
Contact IWRS	X	X	X	X	X	X	X	X
Randomization					X			
Inclusion/Exclusion	X	X						
Medical History	X							
Physical Examination	X							X
Vital Signs[d]	X	X	X	X	X	X	X	X
Height and Weight	X					X[e]		X[e]
C-SSRS[f]	X	X	X	X	X	X	X	X
Clinical Laboratory Tests[g]	X					X		X
Urine Pregnancy Test	X							
Urine Screen for Drug Abuse	X							
Alcohol Breath Test	X							
ECG	X					X		X
MoCA[h]	X							
MDS-UPDRS Parts I-IV	X[i]	X	X		X	X	X	X
PGI-C[j]						X	X	X
CGI-C[k]						X	X	X
PGI-S[l]		X			X			X

Assessment	Screening	3 Weeks of IR CD-LD Dose Adjustment	4 Weeks of IPX203 Dose Conversion			13 Weeks of Double-Blind Therapy		
		**Visit 1**	**Visit 2**	**Visit 3**	**Visit 4 Randomization**	**Visit 5**	**Visit 6**	**Visit 7 / Study Exit/Early Termination[b]**
Study Week[a]	**–4**	**0**	**3**	**5**	**7**	**10**	**15**	**20**
CGI-S[m]		X			X			X
PDQ-39[n]		X			X		X	X
GCSI[o]		X						X
NMSS[p]		X			X		X	X
PDSS-2[q]		X			X		X	X
PAS[r]		X			X		X	X
PD Diary Training; Perform Concordance Testing at Screening Only[s]	X	X	X	X	X	X	X	
Dispense PD Diaries[t]	X	X		X	X	X	X	
Review PD Diaries[u]		X	X		X	X	X	X
Reminder phone calls[v,w]	X[v]	X[w]	X[w]	X[w]	X[w]	X	X	X
Dispense study medication		X	X	X	X	X	X	
Collect empty medication bottles and any unused study drug/Perform study drug accountability			X	X	X	X	X	X
Adverse Events	X	X	X	X	X	X	X	X
Concomitant Medications	X	X	X	X	X	X	X	X

CGI-C = Clinical Global Impression of Change, CGI-S = Clinical Global Impression of Severity, C-SSRS = Columbia-Suicide Severity Rating Scale, ECG = electrocardiogram, GCSI = Gastroparesis Cardinal Symptom Index, HIPAA = Health Insurance Portability and Accountability Act, ICF = informed consent form, IWRS = interactive web response system, MDS-UPDRS = MDS version of Unified Parkinson's Disease Rating Scale, MoCA = Montreal Cognitive Assessment, PAS = Parkinson Anxiety Scale, PD = Parkinson's disease, NMSS = Non-Motor Symptom assessment scale for PD, PDQ-39 = 39-Item Parkinson's Disease Questionnaire, PDSS-2 = Parkinson's Disease Sleep Scale-2, PGI-C = Patient Global Impression of Change, PGI-S = Patient Global Impressions of Severity.

[a] The interval between Screening and Visit 1 (Day 1) should not exceed 4 weeks. Study visits should occur within ± 3 days of their specified timing.

[b] Study Exit procedures to be conducted at the end of Visit 7 or during an early termination visit.
[c] Subjects enrolled at sites in the United States (US) must sign HIPAA authorization prior to the conduct of any study-specific procedures.
[d] Record vital signs (blood pressure, heart rate, respiratory rate, and temperature [Screening and Study Exit only]) after subject has been resting supine for at least 5 minutes, then record orthostatic blood pressure and heart rate after subject has been standing for approximately 2 minutes. At Visits 1 and 4, orthostatic vital signs (blood pressure and heart rate) will be performed in triplicate, each set separated by at least 15 minutes from the previous set.
[e] Weight only.
[f] C-SSRS: Columbia Suicide Severity Rating Scale. See Appendix O.
[g] See Appendix R.
[h] Montreal Cognitive Assessment in the "On" state: see Appendix D.
[i] At Screening MDS-UPDRS Parts I through IV will be done in both the "On" and "Off" state (see Appendix E).
[j] See Appendix F.
[k] See Appendix G.
[l] See Appendix H.
[m] See Appendix I.
[n] See Appendix J.
[o] See Appendix K.
[p] See Appendix L.
[q] See Appendix M.
[r] See Appendix N.
[s] Train at Screening and then as needed at subsequent visits. Perform concordance testing at Screening.
[t] Dispense PD Diaries at Screening and Visits 1, 3, 4, 5, and 6. Call subjects 4 days prior to Visits 1, 2 and 4-7 to remind them to complete PD Diaries. Subjects record diary information for 3 consecutive days immediately prior to each of the visits (Days -3, -2, and -1). Call subjects the day prior to each visit to remind them to bring the PD Diaries, empty medication bottles, and any unused study drug to the office.
[u] Review PD Diaries at Visits 1, 2, and 4-7.
[v] Post-Screening reminder phone call: Notify individuals who successfully complete screening procedures following review of all study entry criteria and clinical laboratory results that they may continue in the study. The interval between Screening and Visit 1 should not exceed 4 weeks.
[w] Reminder phone calls for Visits 1 through 4: In addition to the calls discussed above, make regular phone calls (approximately every 1 to 3 days) to subjects throughout the IR CD-LD dose-adjustment and IPX203 dose-conversion periods to evaluate each subject's adjustment to the study medication regimen.

8.1. Screening Visit

After the subject has signed the informed consent (and HIPAA authorization for US subjects only), complete the following procedures and assessments:

- Obtain an identification number from the Interactive Web Response System (IWRS). The IWRS will assign a 6-digit ID number to each subject, consisting of a 3-digit number representing the investigative site and a 3-digit sequential subject number.
- Review and record study entry criteria (Section 7).
- Perform urine pregnancy test for females of childbearing potential.
- Perform urine screen for drugs of abuse.
- Perform alcohol breath test.
- Complete medical history.
- Perform physical examination, including height and weight.
- Assess vital signs after subject is supine for at least 5 minutes (blood pressure, heart rate, temperature and respiratory rate) and then assess orthostatic blood pressure and heart rate after subject is standing (for approximately 2 minutes).
- Record current CD-LD regimen, other PD medications and their dosing schedule, and other concomitant medications.
- Record AEs.
- Perform a 12-lead ECG.
- Administer C-SSRS (Appendix O).
- Determine MoCA Score in the “On” state (Appendix D).
- Collect blood and urine samples for clinical laboratory studies (Appendix R).
- Determine Hoehn and Yahr staging of PD in the “On” state (part of MDS-UPDRS Part III Motor Examination) (Appendix E).
- Administer MDS-UPDRS Parts I through IV in the “On” and “Off” state (Appendix E).
- Train the subject how to complete the PD Diaries to assess his/her “On” and “Off” states, including assessment of any dyskinesia. After training the subject, perform the concordance testing. The subject’s “On”/”Off” ratings must agree at least 75% of the time with the trained rater during the training sessions. That is, the subject’s “On”/”Off” ratings must agree with the trained rater’s ratings on at least 75% “On”/“Off” states in a single session to qualify for study inclusion. The 75% concordance rate must be based on at least four ratings, and must include at least one “On” and one “Off” state. The ratings should occur every 30 minutes and each session can last up to 4 hours. If the subject fails the first training session, the subject may be trained for one additional 4-hour training session.

- Dispense PD Diaries and instruct the subject to complete the PD Diaries on 3 consecutive days immediately prior to Visit 1.

Notify individuals who successfully complete screening procedures following review of all study entry criteria and clinical laboratory results.

The interval between Screening and Visit 1 (Day 1) should not exceed 4 weeks.

8.2. Visit 1 – Start of IR CD-LD Dose Adjustment

8.2.1. Prior to Visit 1

Contact the subject at least 4 days prior to Visit 1 to remind him/her to complete the 3-day PD Diaries starting 3 consecutive days immediately prior to Visit 1.

The day prior to Visit 1, remind subjects to:

- Bring their completed 3-day PD Diaries to the clinic.

8.2.2. At Visit 1

For Visit 1 complete the following procedures:

- Collect and review the subject's 3-day PD Diaries. Ensure that the subject is averaging at least 2.5 hours per day of "Off" time over 3 days and at least 1.5 hours of "Off" time on each day based on the 3 day PD Diaries. If the subject cannot properly complete the diary, eg, if more than 1 day of the diary is not returned or if more than 1 day of the diary is not valid (ie, more than 2 hours [4 half periods] of the 24-hour diary are missing), he/she will not continue in the study.
- Review inclusion and exclusion criteria to ensure that the subject continues to meet study inclusion criteria.
- Review instruction of 3-day PD Diaries if needed.
- Measure vital signs (respiratory rate, blood pressure, and heart rate after supine for at least 5 minutes, and orthostatic pulse and blood pressure after standing for approximately 2 minutes). Orthostatic vital sign measurements (blood pressure and heart rate) will be performed in triplicate, each set separated by at least 15 minutes from the previous set.
- Administer C-SSRS (Appendix O).
- Administer MDS-UPDRS Parts I through IV (Appendix E).
- Complete PDQ-39 (Appendix J).
- Complete PGI-S (Appendix H).
- Complete CGI-S (Appendix I).
- Complete GCSI (Appendix K).
- Complete Non-motor Symptom assessment scale for PD (NMSS) (Appendix L).

- Complete the Parkinson's Disease Sleep Scale-2 (PDSS-2) (Appendix M).
- Complete Parkinson Anxiety Scale (PAS) (Appendix N).
- Record any AEs and update changes in concomitant medication since the previous visit.
- Dispense PD Diaries.
- Contact IWRS and dispense study medication per IWRS instructions.

8.2.3. Post Visit 1

- Make regular phone calls (approximately every 1 to 3 days) while the IR CD-LD dose is being adjusted. The IR CD-LD dosing regimen should be stable for at least 5 days prior to returning for Visit 2.

8.3. Visits 2 (Week 3) and Visit 3 (Week 5) – IPX203 Dose Conversion

8.3.1. Prior to Visit 2

- Call subjects 4 days prior to Visit 2 and remind them to complete their PD Diaries.
- Call subjects the day prior to Visit 2 to remind them to bring the PD Diaries, empty medication bottles, and any unused study drug to the office.

8.3.2. Prior to Visit 3

- Call subjects the day prior to Visit 3 to remind them to bring back empty medication bottles and any unused study drug to the office.

8.3.3. At Visits 2 and 3

For Visits 2 and 3 complete the following procedures:

- Measure vital signs (respiratory rate, blood pressure, and heart rate after supine for at least 5 minutes, and orthostatic pulse and blood pressure after standing for approximately 2 minutes).
- Administer C-SSRS (Appendix O).
- Collect empty medication bottles and any unused study drug; perform study drug accountability.
- Record and update AEs and concomitant medications.
- Contact IWRS and dispense study medication per IWRS instructions.

<u>Additional Assessments at Visit 2 Only</u>

- Administer MDS-UPDRS Parts I through IV (Appendix E).
- Review PD Diaries. The subject will be terminated from the study if the subject does not average at least 2.5 hours per day of "Off" time over 3 days and at least 1.5 hours of "Off" time on each day based on the 3 day PD Diaries and/or if the subject cannot

properly complete the diary, eg, if more than 1 day of the diary is not returned or if more than 1 day of the diary is not valid (ie, more than 2 hours [4 half periods] of the 24 hour diary are missing).

- Ensure that the subject has been on a stable dosing regimen for at least 5 days prior to dose conversion to IPX203. If the subject is not able to achieve a stable dosing regimen lasting at least 5 days, the subject will be discontinued.
- Conduct PD Diaries training, if needed.

Additional Activities at Visit 3 Only

- Dispense PD Diaries.
- Review instruction of 3-day PD Diaries if needed.

8.3.4. Post Visits 2 and 3

Make regular phone calls (approximately every 1 to 3 days) to subjects throughout the dose conversion period, as needed, to evaluate each subject's adjustment to the study medication. The IPX203 dosing regimen should be stable for at least 5 days prior to returning for Visit 4.

8.4. Visit 4 (Week 7) – Randomization

8.4.1. Prior to Visit 4

- Call subjects 4 days prior to Visit 4 to remind them to complete their PD Diaries.
- Contact subjects 1 day prior to Visit 4 to remind them to bring back the PD Diaries, empty medication bottles, and any unused study drug to the office.

8.4.2. At Visit 4

For Visit 4 complete the following procedures:

- Review PD Diaries. At least 1 day of valid diary data (ie, less than 2 hours [4 half periods] of the 24-hour diary are missing) must be available, otherwise the subject will be terminated from the study.
- Ensure that the subject has been on a stable dosing regimen for at least 5 days prior to randomization. If the subject is not able to achieve a stable dosing regimen lasting at least 5 days, the subject will be discontinued.
- Measure vital signs (respiratory rate, blood pressure, and heart rate after supine for at least 5 minutes, and orthostatic pulse and blood pressure after standing for approximately 2 minutes). Orthostatic vital sign measurements (blood pressure and heart rate) will be performed in triplicate, each set separated by at least 15 minutes from the previous set.
- Administer C-SSRS (Appendix O).
- Administer MDS-UPDRS Parts I through IV (Appendix E).
- Complete PDQ-39 (Appendix J).

- Complete PGI-S (Appendix H).
- Complete CGI-S (Appendix I).
- Complete NMSS (Appendix L).
- Complete PDSS-2 (Appendix M).
- Complete PAS (Appendix N).
- Record and update AEs and concomitant medications.
- Conduct PD diaries training, if needed.
- Collect empty medication bottles and any unused study drug; perform study drug accountability.
- Contact IWRS for randomization and dispense medication per IWRS instructions.
- Dispense PD diaries.

8.5. Visit 5 (Week 10) and Visit 6 (Week 15)

8.5.1. Prior to Visit 5 and 6

- Call subjects 4 days prior to Visits 5 and 6 to remind them to begin recording in their PD diaries on each of the 3 consecutive days immediately prior to each of these visits.
- Call the subjects the day prior to Visits 5 and 6 to remind the subjects to bring in their PD diaries, empty medication bottles, and any unused study drug to the office.

8.5.2. At Visit 5 and 6

For Visits 5 and 6 complete the following procedures (note visit-specific tasks below):

- Measure vital signs (respiratory rate, blood pressure, and heart rate after supine for at least 5 minutes, and orthostatic pulse and blood pressure after standing for approximately 2 minutes).
- Administer C-SSRS (Appendix O).
- Administer MDS-UPDRS Parts I through IV (Appendix E).
- Complete PGI-C (Appendix F).
- Complete CGI-C (Appendix G).
- Review PD diaries.
- Conduct PD diaries training, if needed.
- Dispense PD diaries.
- Collect empty medication bottles and any unused study drug; perform study drug accountability.
- Contact IWRS and dispense study medication per IWRS instructions.

- Record any AEs and update changes in concomitant medication since the previous visit.

Additional Activities at Visit 5 Only:

- Record weight.
- Perform a 12-lead ECG.
- Collect blood and urine samples for clinical laboratory studies (Appendix R).

Additional Activities at Visit 6 Only:

- Complete PDQ-39 questionnaire (Appendix J).
- Complete NMSS (Appendix L).
- Complete the PDSS-2 (Appendix M).
- Complete PAS (Appendix N).

8.6. Visit 7 (Week 20) – End of Study/Study Exit

8.6.1. Prior to Visit 7

- Call subjects 4 days prior to Visit 7 to remind them to begin recording in their PD Diaries on each of the 3 consecutive days immediately prior to Visit 7.
- Call the subjects the day prior to Visit 7 to remind the subjects to bring in their PD Diaries, empty medication bottles, and any unused study drug to the office.

8.6.2. At Visit 7

All enrolled subjects must complete Study Exit procedures at the end of Visit 7 or during an early termination visit:

- Perform physical examination, including weight.
- Measure vital signs (temperature, respiratory rate, blood pressure, and heart rate after supine for at least 5 minutes, and orthostatic pulse and blood pressure after standing for approximately 2 minutes).
- Collect and review 3-day PD Diaries.
- Administer C-SSRS (Appendix O).
- Administer MDS-UPDRS Parts I through IV (Appendix E).
- Complete PGI-C (Appendix F).
- Complete CGI-C (Appendix G).
- Complete PDQ-39 questionnaire (Appendix J).
- Complete NMSS (Appendix L).
- Complete PDSS-2 (Appendix M).

- Complete PAS (Appendix N).
- Complete PGI-S (Appendix H).
- Complete CGI-S (Appendix I).
- Complete GCSI (Appendix K).
- Collect empty medication bottles and any unused study drug; perform study drug accountability.
- Record and update AEs and concomitant medications.
- Collect blood and urine samples for clinical laboratory studies (Appendix R).
- Perform a 12-lead ECG.
- Contact IWRS to record subject disposition.

8.7. Early Termination

8.7.1. Subjects Who Terminate Prior to Randomization

If the subject discontinues the study prior to randomization (Visit 4) the subject should complete the following assessments:

- Perform physical examination, including weight.
- Measure vital signs (temperature, respiratory rate, blood pressure, and heart rate after supine for at least 5 minutes, and orthostatic pulse and blood pressure after standing for approximately 2 minutes).
- Collect and review 3-day PD Diaries when available.
- Administer C-SSRS (Appendix O).
- Administer MDS-UPDRS Parts I through IV (Appendix E).
- Collect empty medication bottles and any unused study drug; perform study drug accountability.
- Record and update AEs and concomitant medications.
- Collect blood and urine samples for clinical laboratory studies (Appendix R).
- Perform a 12-lead ECG.
- Contact IWRS to record subject disposition.

8.7.2. Subjects Who Terminate Early after Randomization

If the subject discontinues the study after randomization (Visit 4), the subject should complete all assessments described in Section 8.6.2.

8.8. Blood Volume

Safety blood draws: Approximately 10 mL of blood will be drawn at Screening, Visit 5, and at Study Exit, for a combined total of 30 mL.

9. TREATMENT OF SUBJECTS

9.1. Description of Study Drug

Study drugs will be provided by Impax for this study:

- IPX203 (carbidopa-levodopa) Extended-Release Capsules containing 35-140 mg of CD-LD for oral administration. The CD-LD ratio is 1:4. In addition, matching placebo capsules will also be provided.
- Immediate-release carbidopa-levodopa (IR CD-LD) tablet containing 25-100 mg of CD-LD, for oral administration. In addition, matching placebo tablets will also be provided.

Table 5: Study Drugs for Study IPX203-B16-02

Investigational Product	Dosage Strength (mg CD-LD) and Form
IPX203 (carbidopa-levodopa) Extended-Release capsules	35-140 mg<br>Capsules for oral administration
IR CD-LD (carbidopa-levodopa) tablets	25-100 mg<br>Tablets for oral administration
IPX203 Placebo capsules	Capsules for oral administration
IR CD-LD Placebo tablets	Tablets for oral administration

9.2. Concomitant Medications

9.2.1. Permitted PD Medications

Concomitant therapy with amantadine, selective monoamine oxidase (MAO) type B inhibitors (eg, selegiline, rasagiline), anticholinergic PD medications (eg, benztropine, trihexyphenidyl), and/or dopamine agonists (except apomorphine) is allowed provided the doses and regimens have been stable for at least 4 weeks prior to Visit 1 and the therapy is intended to be constant throughout the course of the study.

9.2.2. Prohibited Medications and Procedures

Prohibited medications and procedures include the following:

- Received within 4 weeks of Visit 1 or planning to take during participation in the clinical study: any doses of a controlled-release (CR) CD-LD apart from a single daily bedtime dose or any doses of Rytary, additional CD (eg, Lodosyn) or benserazide (eg, Serazide), or catechol-O-methyl transferase inhibitors (entacapone or tolcapone) or medications containing these inhibitors (Stalevo).

- Rescue with additional or modified doses of concomitant PD medications or with use of CD-LD products other than the dispensed study medications is not permitted and will trigger discontinuation from the study.
- Received within 4 weeks of Visit 1 or planning to take during participation in the clinical study: nonselective MAOI, selective MAO type A inhibitor (eg, phenelzine, moclobemide, pirlindole, bifemelane, toloxatone), apomorphine, or antidopaminergic agents including antiemetics.
- Treatment with any dopamine antagonist antipsychotic agents for the purposes of psychosis or bipolar disorder within the last 2 years. Use of antipsychotics to treat conditions other than psychosis or bipolar disorders may be allowed only after consultation with the medical monitor.
- Any neurosurgical procedure for the treatment of PD during the course of the study.

A subject who reports the use of any prohibited medications or procedure will be discontinued.

All medications taken within 30 days prior to signing the informed consent form (ICF) and all concomitant medications taken during the study will be recorded on the case report form (CRF).

9.2.3. Rescue Medications

Rescue with additional or modified doses of concomitant non-CD-LD PD medications is not permitted and will trigger discontinuation from the study. During the dose adjustment and dose conversion periods, rescue with CD-LD products other than the dispensed study medications is not permitted and will trigger discontinuation from the study. No medication adjustments are allowed following randomization and during the double-blind phase of the study and will trigger discontinuation from the study.

9.3. Treatment Compliance

Study drug accountability and reconciliation will be performed by the study staff and the study monitor(s).

9.4. Randomization and Blinding

At Visit 4, subjects will be randomized, stratified by center, in a 1:1 ratio into one of two double-blind parallel treatment arms of IPX203 (and matching IR CD-LD placebo) or IR CD-LD (and matching IPX203 placebo).

10. STUDY DRUG MATERIALS AND MANAGEMENT

10.1. Study Drug

Study drugs include the investigational treatment IPX203 35-140 mg CD-LD (and matching placebo capsules) and the active comparator treatment IR 25-100 mg CD-LD, (and matching placebo tablets).

IPX203 is an extended-release (ER) capsule formulation of CD-LD. Impax will manufacture and provide the IPX203 and matching placebo.

IR CD-LD is commercially available and will be provided by Impax. Matching placebo tablets will be manufactured and provided by Impax.

10.2. Study Drug Packaging and Labeling

Impax or designee will provide study medications in bottles with appropriate labeling affixed.

Labels on the study medication may include the following information:

- name, address, and phone number of the sponsor
- pharmaceutical dosage form/route of administration, quantity of dosage units, the name/identifier, and strength/potency
- batch and/or code number to identify the contents and packaging operation
- trial reference code (protocol number)
- trial subject identification number/treatment number and where relevant, the visit number
- name of investigator
- directions for use: Take tablet(s) or capsule(s) orally with water as directed.
- for clinical trial use only
- storage information: Store at 25°C (77°F), with excursions permitted to 15°C to 30°C (59°F to 86°F). Protect from light and moisture.
- period of use (use-by date, expiry date or retest date as applicable), in month/year format and in a manner that avoids any ambiguity.
- keep out of reach of children
- caution statement: Caution: New Drug—Limited by Federal (or United States) law to investigational use.

10.3. Study Drug Storage

The clinical site should store the study drug at 25°C (77°F), with excursions permitted to 15°C to 30°C (59°F to 86°F). The study drug should be stored in a tightly closed container, protected

from light and moisture. Storage temperature excursions above 30°C (86°F) should be reported by the clinical site to Impax or its designee.

10.4. Study Drug Administration

Subjects will be instructed to take their medications with approximately 240 mL of room-temperature water. The capsules or tablets should not be crushed or chewed.

IR CD-LD tablets may be split to achieve the required doses.

10.5. Study Drug Dispensing and Accountability

The Investigator must ensure that all study medication received at the study site is inventoried and accounted for, and that dispensed study medication is recorded in the subject's source documents, the CRF, and the study medication inventory log. Site personnel must not relabel or reassign study medication to other subjects or to individuals not enrolled in the study. The study monitor verifies medication accountability during monitoring visits.

10.6. Study Drug Handling and Disposal

The Investigator must retain and properly store all partially used and unused study medication until authorized by Impax regarding disposition.

11. ASSESSMENT OF EFFICACY

11.1. Parkinson's Disease Diary

Subjects are to record "asleep," "Off," and "On" without or with (nontroublesome or troublesome) dyskinesias during waking hours every 30 minutes over a 24-hour day. In the PD Diaries, subjects are instructed to indicate for each half-hour their predominant state during most of that period. "Off" is defined as the typical functional state when the medication is no longer providing benefit with regard to mobility, slowness, and stiffness in spite of taking medications. "On" is defined as the typical functional state when a subject has received medication and the medication is providing benefit with regard to mobility, slowness, and stiffness. Dyskinesias are defined as involuntary and irregular twisting and/or turning movements. Dyskinesia movements are usually an effect of medication and occur during "On" time. Nontroublesome dyskinesias do not interfere with function or do not cause meaningful discomfort. Troublesome dyskinesias do interfere with function or do cause meaningful discomfort.

11.2. Patient and Investigator Global Assessments

- Patient Global Impression of Change (Appendix F): The patient will compare his/her condition from the start of the study on a 7-point scale ranging from "Very much worse" (1) to "Very much improved" (7) at the time of the assessment.
- Clinical Global Impression of Change (Appendix G): The clinician will compare the subjects' condition from the start of the study on a 7-point scale ranging from "Very much worse" (1) to "Very much improved" (7) at the time of the assessment.
- Patient Global Impression of Severity (Appendix H): The patient will determine the severity of the disease on a 7-point scale ranging from "Normal, not at all ill" (1) to "Extremely severely ill" (7) at the time of the assessment.
- Clinical Global Impression of Severity (Appendix I): The clinician will determine the severity of the disease on a 7-point scale ranging from "Normal, not at all ill" (1) to "Among the most extremely ill of subjects" (7) at the time of the assessment.

11.3. Movement Disorders Society Version of Unified Parkinson's Disease Rating Scale

The MDS-UPDRS has 4 parts:

- Part I: Non-Motor Aspects of Experiences of Daily Living (nM-EDL) has 2 components. Component IA contains a number of behaviors assessed by the investigator with all pertinent information from the patients and caregivers. Component IB is completed by the patient with or without help from the caregiver but independent of the investigator. These sections can be reviewed by the rater to ensure all questions are answered clearly and the rater can help explain any ambiguities.

- Part II: Motor Aspects of Experiences of Daily Living (M-EDL) is a self-administered questionnaire but can be reviewed by the investigator to ensure completeness and clarity.
- Part III: Motor Examination assesses the motor signs of PD and has instructions for the rater to give to or to demonstrate to the patient. It is completed by the rater.
- Part IV: Motor Complications integrates patient-derived information with the rater's clinical observations and judgements and is completed by the rater. It contains instructions for the rater and instructions to be read to the patient.

11.4. Additional Assessments

- Parkinson's Disease Questionnaire-39 (PDQ-39) is a self-reported questionnaire. Using the 39-items, 8 domains are defined: mobility (Questions 1-10), activities of daily living (ADL) (Questions 11-16), emotional well-being (Questions 17-22), stigma (Questions 23-26), social support (Questions 27-29), cognition (Questions 30-33), communication (Questions 34-36) and bodily discomfort (Questions 37-39).
- Non-Motor Symptom assessment scale for Parkinson's Disease (NMSS) is a 30-item investigator rated questionnaire. The NMSS contains 9 domains: cardiovascular (Questions 1, 2), sleep/fatigue (Questions 3-6), mood/cognition (Questions 7-12), perceptual problems (Questions 13-15), attention/memory (Questions 16-18), gastrointestinal (Questions 19-21), urinary (Questions 22-24), sexual function (Questions 25, 26), and miscellaneous (Questions 27-30).
- Parkinson's Disease Sleep Scale-2 (PDSS-2) is 15-item self-reported questionnaire. Three domains are defined: disturbed sleep (Questions 1-3, 8, 14), motor symptoms at night (Questions 4-6, 12, 13), PD symptoms at night (Questions 7, 9-11, 15).
- Parkinson Anxiety Scale (PAS) is a 12-item patient or observer rated questionnaire with 3 domains: persistent anxiety (Questions A.1-A.5), episodic anxiety (Questions B.1-B.4) and avoidance anxiety (Questions C.1-C.3).

12. ASSESSMENT OF SAFETY

12.1. Safety Parameters

Safety will be assessed by the following parameters:

- Electrocardiograms (ECGs), clinical laboratory tests, physical examinations, the Columbia-Suicide Severity Rating Scale (C-SSRS), and vital signs, including supine and standing orthostatic blood pressure and heart rate.
- Adverse events and concomitant medications will be evaluated throughout the course of the study.

12.2. Adverse Events

12.2.1. Definition of Adverse Event

An adverse event (adverse experience) is any untoward medical occurrence in a patient or clinical trial subject administered a medicinal product and which does not necessarily have to have a causal relationship with this treatment. An adverse event can therefore be any unfavorable and unintended sign (eg, an abnormal laboratory finding), symptom, or disease temporally associated with the use of a medicinal product, whether or not considered related to the medicinal product.

All AEs and any clinically significant physical examination findings, 12-lead ECG abnormalities, or clinical laboratory measurements occurring during the study that were not present prior to administration of study medication and that continue at Study Exit should be followed and evaluated with additional tests, if necessary, until the AEs are medically stable or resolved. Follow-up on these AEs should be recorded on the source documents and reported to Impax.

12.2.2. Recording Adverse Events

Elicit information about AEs with nonselective questions such as: “Have you experienced any changes in your health status since your last visit?” Encourage subjects to report AEs at onset.

Record information for any AE that emerges from the time the subject signs the ICF until Study Exit.

Monitor each subject closely for the development of AEs and record all such events on the AE page of the CRF. Whenever possible, group signs and symptoms that constitute a single diagnosis. For example, cough, rhinitis, and sneezing might be grouped as upper respiratory infection.

For each AE, record the onset date, severity, seriousness, relationship to study medication, date of resolution (or continuing), action taken, and outcome in the CRF. The Investigator is to make a causality assessment (relationship to study medication) for every AE.

12.2.3. Follow-up

The Investigator must follow each AE until resolved or medically stable.

12.2.4. Relationship to Study Drug

The Investigator documents his/her opinion of the relationship of the AE to the study medication as follows:

- Not Related—the experience can be readily explained by the subject's underlying medical condition or concomitant medications and no relationship exists between the study medication and the experience.
- Unlikely Related—the temporal relationship between the AE and the administration of the study medication is uncertain and it is likely that the AE can be explained by the subject's medical condition or other therapies.
- Possibly Related—there is some logical temporal relationship between the AE and the administration of the study medication and the experience is unlikely to be explained by the subject's medical condition or other therapies.
- Related—the temporal relationship is compelling between the administration of the study medication and the AE cannot be explained by the subject's medical condition or other therapies.

12.2.5. Assessment of Severity

Grade each AE for severity and note in the description of the AE. Determine the severity category of mild, moderate, or severe, as defined below, and enter the information on the AE page of the CRF.

- Mild—causing no limitation of usual activities
- Moderate—causing some limitation of usual activities
- Severe—causing inability to carry out usual activities

12.3. Serious Adverse Events

12.3.1. Definition of Serious Adverse Event

A serious adverse event (SAE) is any AE occurring at any dose that results in any of the following outcomes, regardless of relationship to the study medication:

- Death
- A life-threatening adverse drug experience
- Inpatient hospitalization or prolongation of existing hospitalization
- A persistent or significant disability/incapacity
- A congenital anomaly/birth defect

- Important medical events that may not result in death, be life-threatening, or require hospitalization may be considered an SAE when, based upon appropriate medical judgment, they may jeopardize the subject and may require medical or surgical intervention to prevent one of the outcomes listed in this definition.

12.3.2. Reporting Serious Adverse Events

Any SAE that occurs from the time the subject signs an ICF until 30 days after taking the final dose of study medication must be reported by the investigative staff to the Sponsor or the Sponsor's representative within 24 hours of knowledge of the event (see Study Contact Information).

An SAE form must be completed and sent to the Sponsor and/or the Sponsor's representative. All SAEs must also be recorded on the AE page of the CRF. Additionally, all SAEs must be reported to the institutional review board (IRB) per the IRB's requirements.

Those SAEs that are considered both serious and unexpected and related to the study drug are subject to expedited reporting. An "unexpected AE" is any AE where the nature or severity is not consistent with the current investigator brochure (IB) or if an IB is not required or available, the specificity or severity is not consistent with the provided risk information.

Unexpected fatal or life-threatening SAEs related to the study drug must be reported by the Sponsor to the appropriate regulatory authority in an expedited manner (ie, first report within 7 days of first knowledge by the Sponsor). The Sponsor will provide a final written report to that authority within 15 days of initial receipt of information on the event. The Sponsor or the Sponsor's representative will also inform all participating Investigators of the SAE.

Unexpected SAEs that are not fatal or life-threatening must be reported by the Sponsor to the appropriate regulatory authority as soon as possible but no later than 15 calendar days after first knowledge of the SAE by the Sponsor. The Sponsor or the Sponsor's representative also informs all participating Investigators of the SAE.

Subjects withdrawn from the study due to any SAE will be followed until the SAE is resolved or medically stable. Record all SAEs, regardless of severity and whether or not related to the study medication, on the appropriate page of the CRF.

The Investigator must determine whether the seriousness of the event warrants removal of the subject from the study. He/she should, in any case, institute appropriate diagnostic and therapeutic measures and keep the subject under observation for as long as is medically indicated, or refer the subject to appropriate health professionals.

12.4. Pregnancy

Any pregnancy that occurs from the time the subject signs an ICF until 30 days after taking the final dose of study medication must be reported within 24 hours to the Sponsor or the Sponsor's representative and the subject should be terminated from the study. All pregnancies will be followed through to delivery of the infant. If the subject experiences a termination of the pregnancy, it should be reported as defined in Section 12.3.2.

12.5. Other Safety Parameters and Related Information

Additional safety parameters (laboratory tests, 12-lead ECGs, physical examinations, and vital signs), the C-SSRS, the GCSI, and concomitant medications are collected as shown in the Schedule of Assessments in Table 4. Clinical laboratory assessments are listed in Appendix R.

13. STATISTICS

13.1. Study Design and Sample Size Estimation

This is a multicenter, randomized, double-blind, double-dummy, active-controlled, parallel-group study. Assuming a difference of 1 hour between IPX203 and IR CD-LD in "Good on" time and a standard deviation of the treatment differences to be 3.0 hours, a sample size of 210 subjects per arm will be needed to ensure at least 90% power at a 0.05 significance level.

Assuming approximately an 18% prerandomization drop-out, approximately 510 subjects would need to be enrolled to randomize 420 subjects.

13.2. Demographics/Baseline Comparability

The demographics and baseline characteristics will be summarized by treatment arms and overall using descriptive statistics. Demographics information includes age, sex, and race. Baseline disease characteristics include MDS-UPDRS Parts I, II, III, and IV, Hoehn and Yahr stage, MoCA scores, and age of onset of PD. Distributions of dosing information, including LD doses and years on LD, will also be summarized.

13.3. Efficacy Endpoints

- Primary endpoint: Change from baseline in "Good on" time in hours per day, averaged over the PD Diary days, at the end of double-blind treatment period (Visit 7 or early termination). "Good on" time is derived from the 3-day PD Diaries and is defined as the sum of "On" time without dyskinesia and "On" time with nontroublesome dyskinesia.
- Key secondary endpoints:
    - Change from baseline in "Off" time in hours per day, averaged over the PD Diary days at the end of double-blind treatment period (Visit 7 or early termination)
    - Proportion of subjects with either "much improved" or "very much improved" in Patient Global Impression of Change (PGI-C) scores at the end of double-blind treatment period (Visit 7 or early termination)
    - Change from baseline in the MDS-UPDRS Part III at the end of double-blind treatment period (Visit 7 or early termination)
    - Change from baseline in the sum of MDS-UPDRS Parts II and III at the end of double-blind treatment period (Visit 7 or early termination)
- Additional endpoints:

  The following endpoints will be evaluated (at the post-randomization visits) as change from baseline (Visit 4) as well as change from the study entry (Visit 1), when applicable, by visits:
    - Percent "Off" time during waking hours derived from the 3-day PD Diaries
    - Average duration of each continuous "Good on" and each continuous "On"

- Hours of (1) “Off” time (from Visit 1), (2) “Good on” time (from Visit 1), (3) “On” time with dyskinesia, (4) “On” time with troublesome dyskinesia, and (5) “On” time with nontroublesome dyskinesia, and (6) asleep time derived from the 3-day PD Diaries
- Proportion of subjects with an improvement in “Good on” time of at least 1, 1.5, 2, 2.5, and 3 hours
- Proportion of subjects with a reduction in “Off” time of at least 0.5, 1, 1.5, 2, 2.5, and 3 hours
- Proportions of subjects who are “On” upon awakening and “Good on” upon awakening
- Average time to “On” upon awakening
- Change from baseline in the average number of motor fluctuations per day averaged over the PD Diary days. A motor fluctuation is defined as a change from “Off” to “On” state or from “On” to “Off” state.
- MDS-UPDRS total score (sum of Parts I, II, III, and IV) and Parts I, II, and IV separately
- MDS-UPDRS Part III and Parts II + III combined (from Visit 1)
- MDS-UPDRS Part II Question 2.9
- PDQ-39 total score and individual domain scores
- NMSS total score and individual domains
- PDSS-2 total score and individual domains
- PDSS-2 items 9, 10, 11, 12, and 13 combined
- PAS total score and individual domains
- PGI-S
- Proportion of subjects with either “severely ill” or “extremely severely ill” on the PGI-S
- CGI-S
- Proportion of subjects with either “severely ill” or “among the most extremely ill of subjects” on the CGI-S
- PGI-C scores
- CGI-C scores
- Proportion of subjects with either “much improved” or “very much improved” on the CGI-C

13.4. Analysis of Efficacy Data

In order to control the type I error rate, the primary efficacy endpoint and key secondary efficacy endpoints will be tested in a single hierarchical order as detailed in Section 13.8.

13.4.1. Primary Efficacy Endpoint

The primary efficacy endpoint is the mean change from baseline in "Good on" time in hours per day, averaged over the PD Diary days, at the end of the double-blind therapy (Visit 7 or early termination). "Good on" time is derived from the 3-day PD Diaries. For each day, "Good on" time is calculated by adding the number of half-hour intervals in which either an "On without dyskinesia" or "On with nontroublesome dyskinesia" is checked.

The primary efficacy endpoint will be analyzed using a mixed model for repeated measures (MMRM). The model will include baseline (Visit 4) "Good on" time as a covariate, treatment, visit, and pooled center as fixed effects, and a treatment-by-visit interaction. The model will employ an unstructured within subject covariance matrix and a restricted maximum likelihood (ReML) estimation method. The degree-of-freedom of the denominator will be estimated using the Kenward-Roger method (Kenward and Roger 1997). The primary analysis population will be the modified intent-to-treat as defined in Section 13.9. Missing data will be handled as in Section 13.10.

If the model fails to converge due to the unstructured covariance matrix, a simpler covariance matrix will be employed in the order of 1) heterogeneous Toeplitz [SAS PROC MIXED type = TOEPH], 2) heterogeneous autoregressive of order 1 [type = ARH(1)], 3) heterogeneous compound symmetry [type = CSH], 4) Toeplitz [type = TOEP], 5) autoregressive of order 1 [type = AR(1)], 6) compound symmetry [type = CS]. The first covariance structure that does not have a convergence problem will be the one used for the primary analysis.

13.4.2. Key Secondary Efficacy Endpoints

The first key secondary endpoint is the mean change from baseline in "Off" time in hours per day, averaged over the PD Diary days, at the end of the double-blind therapy (Visit 7 or early termination). "Off" time is derived from the 3-day PD Diaries. For each day, "Off" time is calculated by adding the number of half-hour intervals in which an "Off" is checked. This endpoint will be analyzed using a MMRM model with baseline (Visit 4) "Off" time as a covariate, treatment, visit, and pooled center as fixed effects, and a treatment-by-visit interaction. The model will employ an unstructured within subject covariance matrix and a restricted maximum likelihood (ReML) estimation method. The degree-of-freedom of the denominator will be estimated using the Kenward-Roger method (Kenward and Roger 1997).

The proportion of subjects with either "much improved" or "very much improved" in PGI-C scores at the end of the double-blind therapy (Visit 7 or early termination), the second key secondary endpoint, will be analyzed using a Cochran-Mantel-Haenszel test to compare the two treatment groups with pooled center as a stratification factor.

The mean change from baseline in the MDS-UPDRS Part III at the end of the double-blind therapy (Visit 7 or early termination) is the third key secondary endpoint. This endpoint will be analyzed using an MMRM model with baseline (Visit 4) MDS-UPDRS Part III as a covariate,

treatment, visit, and pooled center as fixed effects, and a treatment-by-visit interaction. The model will employ an unstructured within subject covariance matrix and a restricted maximum likelihood (ReML) estimation method. The degree-of-freedom of the denominator will be estimated using the Kenward-Roger method (Kenward and Roger 1997).

The mean change from baseline in sum of the MDS-UPDRS Parts II and III at the end of the double-blind therapy (Visit 7 or early termination) is the fourth key secondary endpoint. This endpoint will be analyzed using an MMRM model with baseline (Visit 4) MDS-UPDRS Parts II and III combined as covariates, treatment, visit, and pooled center as fixed effects, and a treatment-by-visit interaction. The model will employ an unstructured within subject covariance matrix and a restricted maximum likelihood (ReML) estimation method. The degree-of-freedom of the denominator will be estimated using the Kenward-Roger method (Kenward and Roger 1997).

For the 4 key secondary endpoints, the analysis population will be the modified intent-to-treat as defined in Section 13.9. Missing data will be handled as in Section 13.10.

13.4.3. Additional Efficacy Endpoints

In general, continuous endpoints will be summarized by standard descriptive statistics (mean, standard deviation, median, minimum, and maximum). Categorical endpoints will be summarized by frequencies and percentages. Comparisons between the two arms will be explored using appropriate statistical methodologies. Details will be provided in the SAP.

The primary endpoint, key secondary endpoints, as well as other efficacy endpoints will be presented by visit over the whole blinded treatment period from Baseline (Visit 4) to the end of the double-blind treatment period (Visit 7).

Other additional efficacy endpoints collected postrandomization will be analyzed by visit in a fashion similar to the primary and key secondary endpoints.

Additionally the PGI-C and CGI-C will be analyzed using analysis of variance (ANOVA) with treatment and pooled center as factors.

13.5. Center Pooling Algorithm

The center pooling algorithm is as follows.

1. Sort centers from each country from smallest to largest based on the number of subjects in the modified intent-to-treat analysis set (mITT).
2. Centers with less than 5 mITT subjects or at least one mITT subject per treatment group will be pooled with the next smallest center in the same country until the combined center (namely, pseudo-center) has more than 5 mITT subjects and at least one mITT subject per treatment group.
3. If after pooling within the same country, the pseudo-center still has less than 5 mITT subjects or still has zero subjects in one of the treatment group, that pseudo-center will be pooled with the next smallest center in the same geographical region (Western Europe, Eastern Europe, North America).

4. If after pooling within the same geographical region, the pseudo-center still has less than 5 mITT subjects or still has zero subjects in one of the treatment group, that pseudo-center will be pooled with the next smallest center in any region.

The process continues until all pooled pseudo-centers have at least 5 mITT subjects and at least one mITT subject per treatment group. These pooled centers will be used in analyses that adjust for pooled centers.

This pooling algorithm will be detailed in the Statistical Analysis Plan (SAP).

13.6. Sensitivity Analyses of the Primary Endpoint and Key Secondary Endpoints

Sensitivity analyses will be performed with respect to the primary efficacy endpoint and continuous key secondary endpoints ("Off" time, MDS-UPDRS Part III, and MDS-UPDRS Parts II and III combined) as follows.

13.6.1. Assessing Assumptions of the Mixed Model for Repeated Measures (MMRM)

a. The normality and homoscedasticity assumptions will be examined through residual analyses. The normality and homoscedasticity assumptions will further be tested via Shapiro-Wilk (Shapiro and Wilk 1965) and Levene (Levene 1960) tests, respectively, at a 0.05 level of significance. If normality and/or homoscedasticity assumption appears violated, then:

   i. Nonparametric Wilcoxon Rank Sum test will be performed to compare the two treatment groups, with missing data imputed by the last observation carried forward (LOCF) method.

   ii. Multiple imputation rank based analysis: instead of missing data imputed by the LOCF method, in this analysis, missing data at Visit 7 will be imputed multiple times to create 50 complete datasets. The multiple imputation procedure is described in Section 13.6.4 (part of the pattern-mixture model), using $f = 0\%$. The Wilcoxon Rank Sum test will be performed on each of the 50 datasets. The results are then combined using Rubin's rule (Rubin 1987) via SAS PROC MIANALYZE.

b. Missing at Random (MAR) assumption will be evaluated as discussed in Section 13.6.4.

13.6.2. Complete Case Analysis

The primary endpoint will be analyzed using an ANCOVA model with "Good on" time at baseline (Visit 4) as a covariate, pooled center and treatment as factors. The model will be performed on subjects with both baseline "Good on" time and Visit 7 "Good on" time.

13.6.3. Single LOCF/BLOCF Imputation

The primary efficacy endpoint will be analyzed using an ANCOVA model with "Good on" time at Visit 4 as a covariate, pooled center and treatment as factors. Missing data will be imputed by the LOCF and baseline observation carried forward (BLOCF) methods. These analyses will be performed on the mITT population.

13.6.4. Pattern-Mixture Model

If an overall dropout rate postrandomization is > 15%, pattern-mixture models (PMM) will be employed to assess the robustness of the results under the missing not at random (MNAR) assumption. The pattern for PMM is defined by patients' last visit with an observed primary efficacy endpoint and the reason for dropout.

Multiple imputation with mixed missing data mechanism (MNAR for a missing data pattern and MAR for others) will be used to investigate the robustness of the primary result. Four specific data patterns will be examined:

1. Dropout at Visit 5 and reason = Lack of efficacy in IPX203 treatment arm,
2. Dropout at Visit 5 and reason = Lack of efficacy or adverse events in IPX203 treatment arm,
3. Dropout at Visit 6 and reason = Lack of efficacy in IPX203 treatment arm,
4. Dropout at Visit 6 and reason = Lack of efficacy or adverse events in IPX203 treatment arm.

The missing values will be imputed 50 times (multiple imputation) under the assumption that the distribution of the missing values is the same as that of the observed values. The PMM then investigates the departure from the MAR assumption by progressively decreasing the outcome (the "penalty") for those on IPX203 arm who fall into an assumed MNAR pattern above. For the dropout subjects on IPX203 arm that fall into one of the patterns above, the "penalty" is obtained by subtracting the imputed missing data after dropout by a factor *f*, with *f* starts from 0%, 5%, 10%, 15%, 20%, 25%, 30%, …, 100% of the treatment difference seen in the primary model. This process continues until the conclusion from the primary analysis is overturned (a tipping point). In other words, if the dropout subject is from IPX203 arm and the dropout pattern falls into one of the 4 patterns above, then the subject's imputed value will be adjusted downward by a factor *f*, where *f* goes from 0% to 100% of the treatment difference seen in the primary model. Note that if 0% is used, the analysis is essentially multiple imputation under MAR assumption. On the other hand, if 100% is used, then the analysis is essentially a "jump to reference" where outcome on IPX203 arm is assumed to be the same as outcome on IR CD-LD. After imputations, the dataset will be analyzed using an MMRM model similar to the primary analysis model. The results will then be combined using the Rubin's rule (Rubin 1987) via SAS PROC MIANALYZE.

The procedure will be carried out in SAS as follows:

a. Use Monte Carlo Markov Chain (MCMC) method in SAS PROC MI by treatment group to impute the intermittent missing data to form monotone missingness.
b. Use MAR-based multiple imputation in SAS PROC MI to impute the missing data (SAS MONOTONE statement).
c. For dropout subjects in IPX203 arm who fall into an MNAR pattern specified above, a delta which equals to *f* times the treatment difference obtained from the primary MMRM analysis at Visit 7 will be subtracted from their imputed values for all visits after the dropout ("penalizing" IPX203 arm).

d. After imputation, use the MMRM model as in the primary analysis model to analyze the complete data along with the imputed data.

e. Repeat steps a through d 50 times.

Combine results using Rubin's rule (Rubin 1987) via SAS PROC MIANALYZE.

13.7. Subgroup Analyses

The primary, key secondary endpoints, as well as overall summary of adverse events, will be examined for the following subgroups.

- Age: < 65, ≥ 65 years old at study entry
- Sex: Males, Females
- Race: Caucasians, non-Caucasians

Additionally, the following subgroups may be examined:

- Region
- Ethnicity
- Concomitant medications
- Weight
- Body mass index (BMI)
- PD duration
- Age of PD onset
- "Good On" time and "Off" time at study entry.

For all subgroup efficacy analyses, the same analysis methods as the primary and key secondary endpoints will be applied, unless the sample size in one of the subgroups becomes too small to hinder the statistical analysis. In that case, no inferential statistics will be provided for such a subgroup. The details for final subgroup analyses will be documented in the SAP.

13.8. Multiplicity Adjustments

The primary endpoint and 4 key secondary endpoints will be tested in a sequential hierarchical order as follows.

1. The primary endpoint, the mean change from baseline in "Good on" time (hours per day), will be tested first at a 0.05 level of significance.
2. If statistical significance is demonstrated, then the first key secondary endpoint, the mean change from baseline in "Off" time (hours per day), will be tested next at a 0.05 level of significance.
3. If statistical significance is demonstrated, then the second key secondary endpoint, the proportion of subjects with either "much improved" or "very much improved" on the PGI-C, will be tested next at a 0.05 level of significance.

4. If statistical significance is demonstrated, then the third key secondary endpoint, the mean change from baseline in the MDS-UPDRS Part III, will be tested at a 0.05 level of significance.
5. If statistical significance is demonstrated, then the fourth key secondary endpoint, the mean change from baseline in the sum of the MDS-UPDRS Parts II and III combined will be tested next at a 0.05 level of significance.

For the other efficacy endpoints, no adjustment will be made.

13.9. Analysis Populations

13.9.1. Safety Analysis Set

The Safety Analysis set will include all subjects who were treated with any study drug.

13.9.2. Intent-to-Treat Analysis Set

The Intent-to-treat Analysis Set will include all subjects who were randomized and treated with any study drug and have a baseline and at least one postbaseline efficacy assessment.

13.9.3. Modified Intent-to-Treat Analysis Set

The Modified Intent-to-treat Analysis Set will include all subjects who were randomized and treated and have a valid baseline PD Diary and at least one valid postrandomization PD Diary. This analysis set will be used for the primary analysis and key secondary analyses.

13.9.4. Completers Analysis Set

The Completers Analysis Set will include all subjects who were randomized and treated and complete the study.

13.10. Handling of Missing Data

13.10.1. Missing Data for PD Diaries

An MMRM approach will be used to handle missing visit data. MMRM analysis will use all available valid visit data, including subjects with some missing visit data, in order to arrive at an estimate of the mean treatment effect.

A PD Diary is valid if at least 1 day of diary data is available using the rules defined below.

Imputation of missing data for a PD Diary day will be required if a PD Diary is not completed for a full day (6 am to 5:30 am). In this case, the method of imputation will be dependent upon the amount and pattern of missing data.

- For subjects with more than 1 day of diary data, the following rules will apply:
    1. If more than 4 half-hour time intervals are missing, then that particular day will not be included in the analysis. The missing data will be handled in the MMRM model.
    2. If a one-half hour time interval is missing and the observations on either side of the time interval are not missing, then the missing time interval will be imputed by

assigning a value of the previous measurement for the first 15 minutes and the value of the next measurement for the second 15 minutes.

3. If 2, 3, or 4 consecutive half-hour time intervals are missing, and these time intervals are available from other days of the visit then the following rules will be applied:
   a. For missing time intervals on Day 1, data from Day 2 will be used for imputation for the same time intervals. If Day 2 data is also incomplete or not available, then Day 3 data will be used.
   b. For missing time intervals on Day 2, data from Day 3 will be used for imputation if available; otherwise Day 1 data will be used.
   c. Data from Day 2 will be used for imputing missing time intervals on Day 3. If data from Day 2 is not available, then Day 1 data will be used for imputation.
   d. If data at the same time period are missing across all days, then the approach will be to split the individual missing half-hour intervals into 2 periods, with the first-half interval being imputed with data from the immediate previous nonmissing time period and the second-half interval being imputed with the next nonmissing time interval.

- For subjects with only 1 day of diary data, the following rules will apply:
  1. If more than 4 half-hour time intervals are missing, then that particular day will not be included in the analysis. The missing data will be handled in the MMRM model.
  2. If a one-half hour time interval is missing and the observations on either side of the time interval are not missing, then the missing time interval will be imputed by assigning a value of the previous measurement for the first 15 minutes and the value of the next measurement for the second 15 minutes.
  3. If 2, 3, or 4 consecutive half-hour intervals are missing, then the approach will be to split the individual missing half-hour intervals into 2 periods, with the first-half interval being imputed with data from the immediate previous nonmissing time period and the second-half interval being imputed with the next nonmissing time interval.

13.10.2. Missing Data for Global Assessments (PGI-C, CGI-C, PGI-S and CGI-S)

For subjects with missing PGI-C or CGI-C for a particular visit, the data will be imputed as nonresponders (ie, not being “much improved” or “very much improved”).

For subjects with missing PGI-S or CGI-S for a particular visit, the data will be imputed as nonresponders (ie, being “severely ill” or “extremely severely ill” for PGI-S and being “severely ill” or “among the most extremely ill of subjects” for CGI-S).

13.10.3. Missing Data for MDS-UPDRS

If the MDS-UPDRS are missing for the particular visit, the missing data will be handled via the MMRM model.

If component questions are missing for a particular part of the MDS-UPDRS questionnaire, the missing items are assigned the average value for other items in that part as follows (Goetz 2015):

- For Part I (13 questions): up to 1 missing question will be imputed using the average value of the remaining 12 questions.
- For Part II (13 questions): up to 2 missing questions will be imputed using the average value of the remaining 11 questions.
- For Part III (33 questions): up to 7 missing questions will be imputed using the average value of the remaining 26 questions.
- Part IV (6 questions): no imputation is done.

If more component questions are missing than above for a particular part of the MDS-UPDRS questionnaire, the entire questionnaire will not be included in the analysis for that particular assessment. Missing data will be handled in a fashion similar to PD Diary data (Section 13.10.1) using the MMRM model.

For quality-of-life endpoints, missing responses within a questionnaire will not be imputed.

13.11. Analysis of Safety

The safety analysis will include all subjects who receive at least 1 dose of study medication. Reported AEs will be coded using the Medical Dictionary for Regulatory Activities (MedDRA). All AEs will be summarized by system organ class and preferred terms within system organ class. The severity, seriousness, and relationship to study medication will also be summarized by treatment arms. Each AE (based on preferred term) is counted once for a given subject. If the same AE occurred on multiple occasions, the highest severity and least complimentary relationship will be assumed.

The incidence of treatment-emergent AEs and serious AEs will be summarized by treatment arms.

Additionally, laboratory test data, physical examinations, vital signs, ECGs, C-SSRS, and GCSI will be summarized by treatment arms.

14. ADMINISTRATIVE PROCEDURES

14.1. Guidelines for Good Clinical Practice

This study will be conducted in accordance with principles of Good Clinical Practice (GCP) as promulgated by the ICH. Good Clinical Practice is an international ethical and scientific quality standard for designing, conducting, recording, and reporting trials that involve the participation of human subjects. Compliance with this standard provides public assurance that the rights, safety, and well-being of human subjects are protected under current ethical principles, and that the clinical trial data are credible. Current GCP standards may be found in ICH Guidance E6 (Good Clinical Practice: Consolidated Guidance). This guidance describes the principles of GCP and the obligations of the institutional review board (IRB), the Investigator and the Sponsor in conducting this study in accordance with those principles.

14.2. Institutional Review Board Approval

The review of this protocol by an IRB and the performance of all aspects of the study, including the methods used for obtaining informed consent, must be in accordance with principles enunciated in the ICH and GCP Guidelines and by the appropriate regulatory authorities.

The Investigator is responsible for preparing documents for submission to the relevant IRB and obtaining written approval for this study. Institutional Review Board approval must be obtained prior to the initiation of the study. The Investigator's continued participation in the study is contingent on renewing approval with the IRB at least annually.

14.3. Informed Consent

Site personnel should prepare an Informed Consent Form (ICF) incorporating the necessary elements of consent. The ICF is to be approved by Impax prior to submission to the IRB. The Investigator or his/her staff must explain the nature of the investigation and the risks involved to each subject prior to screening, and obtain a signed ICF. The subject should also be informed that he/she is free to voluntarily withdraw from the study at any time.

14.4. Study Monitoring

Impax representatives or designees will conduct site visits to the investigational facilities for the purpose of monitoring the study. The Investigator agrees to allow the monitor to inspect the drug storage area, study drug stocks, drug accountability records, subject charts and study source documents, and other records relevant to study conduct. The Investigator must permit access to such records if a regulatory or compliance audit is required.

14.5. Protocol Amendments

All amendments to the protocol must be documented in writing, reviewed and approved by the Sponsor and Investigator, and submitted to the IRB for approval prior to implementation. If the protocol amendment substantially alters the study design or potential risk to the subject, a new

written ICF for continued participation in the study must be obtained from each subject affected by the change.

14.6. Termination of Study

The Sponsor has the right to terminate this study and remove all study material from the site at any time for medical or administrative reasons. In this event, the Sponsor will endeavor to give adequate notice to allow safe withdrawal of subjects from the study.

14.7. Case Report Forms

Site personnel should collect and record data for the study as source documents, and transfer the data into the CRF.

The Investigator must ensure that complete data for the clinical study are collected and accurately documented in the appropriate sections of the CRF and adequately supported by the appropriate source documentation. In addition, it is the Investigator’s responsibility to provide signatures where requested indicating concurrence with data in the CRF.

14.8. Investigator’s Final Conduct Report

At the completion of the study, the Investigator must provide Impax a copy of the final conduct report that was submitted to their IRB, including a review of AEs.

14.9. Records Retention

International Conference on Harmonization, GCP, and US FDA guidelines require that essential documents be retained until at least 2 years after the last approval of a marketing application and until there are no pending or contemplated marketing applications, or at least 2 years have elapsed since the formal discontinuation of clinical development of the investigational product.

However, the essential documents should be retained for a longer period if required by the applicable regulatory requirements or by an agreement with the Sponsor. Records should never be destroyed without written approval from the Sponsor.

If an Investigator leaves the institution, he/she must transfer responsibilities for record retention to another individual willing to accept them. The Investigator must notify the Sponsor in writing of the transfer of study documents before the transfer of the study documents.

15. PUBLICATION POLICY

Study results may not be published without prior written approval from Impax.

16. LIST OF REFERENCES

Blanchet PJ, Calon F, Martel JC, et al. Continuous administration decreases and pulsatile administration increases behavioral sensitivity to a novel dopamine D2 agonist (U-91356A) in MPTP-exposed monkeys. J Pharmacol Exp Ther. 1995;272:854-859.

Engber TM, Susel Z, Juncos JL, Chase TN. Continuous and intermittent levodopa differentially affect rotation induced by D-1 and D-2 dopamine agonists. European Journal of Pharmacology. 1989;168:291-298.

Fahn S. Parkinson disease, the effect of levodopa, and the ELLDOPA trial. Earlier vs Later L DOPA. Arch Neurol. 1999 May;56(5):529-35.

Food and Drug Administration 2003. Guidance for Industry. Bioavailability and Bioequivalence Studies for Orally Administered Drug Products — General Considerations. U.S. Department of Health and Human Services. Food and Drug Administration. Center for Drug Evaluation and Research (CDER). March 2003.

Goetz CG, Luo S, Wang L, Tilley BC, LaPelle NR, Stebbins GT. Handling missing values in the MDS-UPDRS. Mov Disord. 2015 Oct;30(12):1632-8.

Juncos JL, Engber TM, Raisman R, et al. Continuous and intermittent levodopa differentially affect basal ganglia function. Ann Neurol. 1989;25:473-478.

Kenward MG and Roger JH. Small sample inference for fixed effects from restricted maximum likelihood. Biometrics. 1997;53:983-997.

Kurlan R, Rothfield KP, Woodward WR, Nutt JG, Miller C, Lichter D, et al. Erratic gastric emptying of levodopa may cause “random” fluctuations of parkinsonian mobility. Neurology. 1988;38(3):419-21.

Levene H. In contributions to probability and statistics: Essays in honor of Harold Hotelling, I. Olkin et al. eds. Stanford University Press. 1960: 278-292.

Melamed E, Bitton V, Zelig O. Delayed onset of responses to single doses of L-dopa in parkinsonian fluctuators on long-term L-dopa therapy. Clin Neuropharmacol. 1986;9(2):182-8.

Mizuno Y. Where do we stand in the treatment of Parkinson's disease? Neurology. 2007;254:13-18.

Nilsson D, Nyholm D, Aquilonius S-M. Duodenal levodopa infusion in Parkinson's disease--long-term experience. Acta Neurologica Scandinavica. 2001;104:343-348.

Nyholm D, Nilsson Remahl AI, Dizdar N, et al. Duodenal levodopa infusion monotherapy vs oral polypharmacy in advanced Parkinson disease. Neurology. 2005;64:216-223.

Rubin DB. Multiple Imputation in Nonresponse in Surveys. San Francisco, CA: Wiley; 1987.

Shapiro SS and Wilk MB. An analysis of variance test for normality (complete samples). Biometrika. 1965;52(3-4): 591-611.

Sinemet (Carbidopa-Levodopa) Tablets Prescribing Information. Whitehouse Station, NJ: Manufactured by Merck & Co., Inc.; Marketed by Bristol-Myers Squibb Company; 2013.

Stocchi F, Quinn NP, Barbato L, Patsalos PN, O'Connel MT, Ruggieri S, et al. Comparison between a fast and a slow release preparation of levodopa and a combination of the two: a clinical and pharmacokinetic study. Clinical Neuropharmacol. 1994;17(1):38-44.

Stocchi F, Vacca L, et al. Intermittent vs continuous levodopa administration in patients with advanced Parkinson disease: a clinical and pharmacokinetic study. Arch Neurol. 2005:62(6):905-910.

17. APPENDICES

APPENDIX A. PRESCRIBING INFORMATION FOR IR CD-LD

SINEMET®
(carbidopa levodopa)
Tablets

DESCRIPTION

SINEMET® (carbidopa levodopa) is a combination of carbidopa and levodopa for the treatment of Parkinson's disease and syndrome.

Carbidopa, an inhibitor of aromatic amino acid decarboxylation, is a white, crystalline compound, slightly soluble in water, with a molecular weight of 244.3. It is designated chemically as (—)-L-α-hydrazino-α-methyl-β-(3,4-dihydroxybenzene) propanoic acid monohydrate. Its empirical formula is $C_{10}H_{14}N_2O_4 \cdot H_2O$, and its structural formula is:

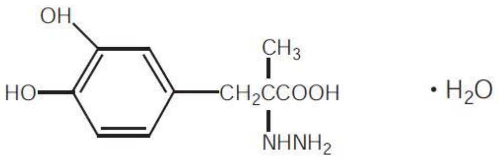

Tablet content is expressed in terms of anhydrous carbidopa which has a molecular weight of 226.3.

Levodopa, an aromatic amino acid, is a white, crystalline compound, slightly soluble in water, with a molecular weight of 197.2. It is designated chemically as (—)-L-α-amino-β-(3,4-dihydroxybenzene) propanoic acid. Its empirical formula is $C_9H_{11}NO_4$, and its structural formula is:

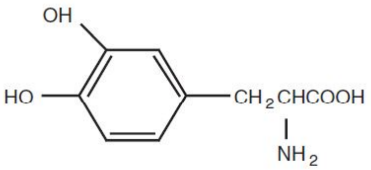

SINEMET is supplied as tablets in three strengths:

SINEMET 25-100, containing 25 mg of carbidopa and 100 mg of levodopa.

SINEMET 10-100, containing 10 mg of carbidopa and 100 mg of levodopa.

SINEMET 25-250, containing 25 mg of carbidopa and 250 mg of levodopa.

Inactive ingredients are hydroxypropyl cellulose, pregelatinized starch, crospovidone, microcrystalline cellulose, and magnesium stearate. SINEMET 10-100 and 25-250 Tablets also contain FD&C Blue #2/Indigo Carmine AL. SINEMET 25-100 Tablets also contain D&C Yellow #10 Lake.

CLINICAL PHARMACOLOGY

Mechanism of Action

Parkinson's disease is a progressive, neurodegenerative disorder of the extrapyramidal nervous system affecting the mobility and control of the skeletal muscular system. Its characteristic features include resting tremor, rigidity, and bradykinetic movements. Symptomatic treatments, such as levodopa therapies, may permit the patient better mobility.

Current evidence indicates that symptoms of Parkinson's disease are related to depletion of dopamine in the corpus striatum. Administration of dopamine is ineffective in the treatment of Parkinson's disease apparently because it does not cross the blood-brain barrier. However, levodopa, the metabolic precursor of dopamine, does cross the blood-brain barrier, and presumably is converted to dopamine in the brain. This is thought to be the mechanism whereby levodopa relieves symptoms of Parkinson's disease.

Pharmacodynamics

When levodopa is administered orally, it is rapidly decarboxylated to dopamine in extracerebral tissues so that only a small portion of a given dose is transported unchanged to the central nervous system. For this reason, large doses of levodopa are required for adequate therapeutic effect, and these may often be accompanied by nausea and other adverse reactions, some of which are attributable to dopamine formed in extracerebral tissues.

Since levodopa competes with certain amino acids for transport across the gut wall, the absorption of levodopa may be impaired in some patients on a high protein diet.

Carbidopa inhibits decarboxylation of peripheral levodopa. It does not cross the blood-brain barrier and does not affect the metabolism of levodopa within the central nervous system.

The incidence of levodopa-induced nausea and vomiting is less with SINEMET than with levodopa. In many patients, this reduction in nausea and vomiting will permit more rapid dosage titration.

Since its decarboxylase inhibiting activity is limited to extracerebral tissues, administration of carbidopa with levodopa makes more levodopa available for transport to the brain.

Pharmacokinetics

Carbidopa reduces the amount of levodopa required to produce a given response by about 75% and, when administered with levodopa, increases both plasma levels and the plasma half-life of levodopa, and decreases plasma and urinary dopamine and homovanillic acid.

The plasma half-life of levodopa is about 50 minutes, without carbidopa. When carbidopa and levodopa are administered together, the half-life of levodopa is increased to about 1.5 hours. At steady state, the bioavailability of carbidopa from SINEMET tablets is approximately 99% relative to the concomitant administration of carbidopa and levodopa.

In clinical pharmacologic studies, simultaneous administration of carbidopa and levodopa produced greater urinary excretion of levodopa in proportion to the excretion of dopamine than administration of the two drugs at separate times.

Pyridoxine hydrochloride (vitamin B_6), in oral doses of 10 mg to 25 mg, may reverse the effects of levodopa by increasing the rate of aromatic amino acid decarboxylation. Carbidopa inhibits this action of pyridoxine; therefore, SINEMET can be given to patients receiving supplemental pyridoxine (vitamin B_6).

Special Populations

Geriatric: A study in eight young healthy subjects (21-22 yr) and eight elderly healthy subjects (69-76 yr) showed that the absolute bioavailability of levodopa was similar between young and elderly subjects following oral administration of levodopa and carbidopa. However, the systemic exposure (AUC) of levodopa was increased by 55% in elderly subjects compared to young subjects. Based on another study in forty patients with Parkinson's disease, there was a correlation between age of patients and the increase of AUC of levodopa following administration of levodopa and an inhibitor of peripheral dopa decarboxylase. AUC of levodopa was increased by 28% in elderly patients (≥ 65 yr) compared to young patients (< 65 yr). Additionally, mean value of Cmax for levodopa was increased by 24% in elderly patients (≥ 65 yr) compared to young patients (< 65 yr) (see PRECAUTIONS, *Geriatric Use*).

The AUC of carbidopa was increased in elderly subjects (n=10, 65-76 yr) by 29% compared to young subjects (n=24, 23-64 yr) following IV administration of 50 mg levodopa with carbidopa (50 mg). This increase is not considered a clinically significant impact.

INDICATIONS AND USAGE

SINEMET is indicated in the treatment of Parkinson's disease, post-encephalitic parkinsonism, and symptomatic parkinsonism that may follow carbon monoxide intoxication or manganese intoxication.

Carbidopa allows patients treated for Parkinson's disease to use much lower doses of levodopa. Some patients who responded poorly to levodopa have improved on SINEMET. This is most likely due to decreased peripheral decarboxylation of levodopa caused by administration of carbidopa rather than by a primary effect of carbidopa on the nervous system. Carbidopa has not been shown to enhance the intrinsic efficacy of levodopa.

Carbidopa may also reduce nausea and vomiting and permit more rapid titration of levodopa.

CONTRAINDICATIONS

Nonselective monoamine oxidase (MAO) inhibitors are contraindicated for use with SINEMET. These inhibitors must be discontinued at least two weeks prior to initiating therapy with SINEMET. SINEMET may be administered concomitantly with the manufacturer's recommended dose of an MAO inhibitor with selectivity for MAO type B (e.g., selegiline HCl) (see PRECAUTIONS, *Drug Interactions*).

SINEMET is contraindicated in patients with known hypersensitivity to any component of this drug, and in patients with narrow-angle glaucoma.

WARNINGS

When SINEMET is to be given to patients who are being treated with levodopa, levodopa must be discontinued at least twelve hours before therapy with SINEMET is started. In order to reduce adverse reactions, it is necessary to individualize therapy. See DOSAGE AND ADMINISTRATION section before initiating therapy.

The addition of carbidopa with levodopa in the form of SINEMET reduces the peripheral effects (nausea, vomiting) due to decarboxylation of levodopa; however, carbidopa does not decrease the adverse reactions due to the central effects of levodopa. Because carbidopa permits more levodopa to reach the brain and more dopamine to be formed, certain adverse central nervous system (CNS) effects, e.g., dyskinesias (involuntary movements), may occur at lower dosages and sooner with SINEMET than with levodopa alone.

All patients should be observed carefully for the development of depression with concomitant suicidal tendencies.

SINEMET should be administered cautiously to patients with severe cardiovascular or pulmonary disease, bronchial asthma, renal, hepatic or endocrine disease.

As with levodopa, care should be exercised in administering SINEMET to patients with a history of myocardial infarction who have residual atrial, nodal, or ventricular arrhythmias. In such patients, cardiac function should be monitored with particular care during the period of initial dosage adjustment, in a facility with provisions for intensive cardiac care.

As with levodopa, treatment with SINEMET may increase the possibility of upper gastrointestinal hemorrhage in patients with a history of peptic ulcer.

Falling Asleep During Activities of Daily Living and Somnolence

Patients taking SINEMET alone or with other dopaminergic drugs have reported suddenly falling asleep without prior warning of sleepiness while engaged in activities of daily living (includes operation of motor vehicles). Road traffic accidents attributed to sudden sleep onset have been reported. Although many patients reported somnolence while on dopaminergic medications, there have been reports of road traffic accidents attributed to sudden onset of sleep in which the patient did not perceive any warning signs, such as excessive drowsiness, and believed that they were alert immediately prior to the event. Sudden onset of sleep has been reported to occur as long as one year after the initiation of treatment.

Falling asleep while engaged in activities of daily living usually occurs in patients experiencing pre-existing somnolence, although some patients may not give such a history. For this reason, prescribers should reassess patients for drowsiness or sleepiness especially since some of the events occur well after the start of treatment. Prescribers should be aware that patients may not acknowledge drowsiness or sleepiness until directly questioned about drowsiness or sleepiness during specific activities. Patients should be advised to exercise caution while driving or operating machines during treatment with SINEMET. Patients who have already experienced somnolence or an episode of sudden sleep onset should not participate in these activities during treatment with SINEMET.

Before initiating treatment with SINEMET, advise patients about the potential to develop drowsiness and ask specifically about factors that may increase the risk for somnolence with SINEMET such as the use of concomitant sedating medications and the presence of sleep disorders. Consider discontinuing SINEMET in patients who report significant daytime sleepiness or episodes of falling asleep during

activities that require active participation (e.g., conversations, eating, etc.). If treatment with SINEMET continues, patients should be advised not to drive and to avoid other potentially dangerous activities that might result in harm if the patients become somnolent. There is insufficient information to establish that dose reduction will eliminate episodes of falling asleep while engaged in activities of daily living.

Hyperpyrexia and Confusion

Sporadic cases of a symptom complex resembling neuroleptic malignant syndrome (NMS) have been reported in association with dose reductions or withdrawal of certain antiparkinsonian agents such as levodopa, carbidopa levodopa, or carbidopa levodopa extended release. Therefore, patients should be observed carefully when the dosage of levodopa is reduced abruptly or discontinued, especially if the patient is receiving neuroleptics.

NMS is an uncommon but life-threatening syndrome characterized by fever or hyperthermia. Neurological findings, including muscle rigidity, involuntary movements, altered consciousness, mental status changes; other disturbances, such as autonomic dysfunction, tachycardia, tachypnea, sweating, hyper- or hypotension; laboratory findings, such as creatine phosphokinase elevation, leukocytosis, myoglobinuria, and increased serum myoglobin have been reported.

The early diagnosis of this condition is important for the appropriate management of these patients. Considering NMS as a possible diagnosis and ruling out other acute illnesses (e.g., pneumonia, systemic infection, etc.) is essential. This may be especially complex if the clinical presentation includes both serious medical illness and untreated or inadequately treated extrapyramidal signs and symptoms (EPS). Other important considerations in the differential diagnosis include central anticholinergic toxicity, heat stroke, drug fever, and primary central nervous system (CNS) pathology.

The management of NMS should include: 1) intensive symptomatic treatment and medical monitoring and 2) treatment of any concomitant serious medical problems for which specific treatments are available. Dopamine agonists, such as bromocriptine, and muscle relaxants, such as dantrolene, are often used in the treatment of NMS; however, their effectiveness has not been demonstrated in controlled studies.

PRECAUTIONS

General

As with levodopa, periodic evaluations of hepatic, hematopoietic, cardiovascular, and renal function are recommended during extended therapy.

Patients with chronic wide-angle glaucoma may be treated cautiously with SINEMET provided the intraocular pressure is well-controlled and the patient is monitored carefully for changes in intraocular pressure during therapy.

Dyskinesia

Levodopa alone, as well as SINEMET, is associated with dyskinesias. The occurrence of dyskinesias may require dosage reduction.

Hallucinations / Psychotic-Like Behavior

Hallucinations and psychotic-like behavior have been reported with dopaminergic medications. In general, hallucinations present shortly after the initiation of therapy and may be responsive to dose reduction in levodopa. Hallucinations may be accompanied by confusion and to a lesser extent sleep disorder (insomnia) and excessive dreaming.

SINEMET may have similar effects on thinking and behavior. This abnormal thinking and behavior may present with one or more symptoms, including paranoid ideation, delusions, hallucinations, confusion, psychotic-like behavior, disorientation, aggressive behavior, agitation, and delirium.

Ordinarily, patients with a major psychotic disorder should not be treated with SINEMET, because of the risk of exacerbating psychosis. In addition, certain medications used to treat psychosis may exacerbate the symptoms of Parkinson's disease and may decrease the effectiveness of SINEMET.

Impulse Control / Compulsive Behaviors

Reports of patients taking dopaminergic medications (medications that increase central dopaminergic tone), suggest that patients may experience an intense urge to gamble, increased sexual urges, intense

urges to spend money, binge eating, and/or other intense urges, and the inability to control these urges. In some cases, although not all, these urges were reported to have stopped when the dose was reduced or the medication was discontinued. Because patients may not recognize these behaviors as abnormal, it is important for prescribers to specifically ask patients or the caregivers about the development of new or increased gambling urges, sexual urges, uncontrolled spending or other urges while being treated with SINEMET. Physicians should consider dose reduction or stopping the medication if a patient develops such urges while taking SINEMET [see *Information for Patients*].

Melanoma

Epidemiological studies have shown that patients with Parkinson's disease have a higher risk (2- to approximately 6-fold higher) of developing melanoma than the general population. Whether the increased risk observed was due to Parkinson's disease or other factors, such as drugs used to treat Parkinson's disease, is unclear.

For the reasons stated above, patients and providers are advised to monitor for melanomas frequently and on a regular basis when using SINEMET for any indication. Ideally, periodic skin examinations should be performed by appropriately qualified individuals (e.g., dermatologists).

Information for Patients

The patient should be informed that SINEMET is an immediate-release formulation of carbidopa levodopa that is designed to begin release of ingredients within 30 minutes. It is important that SINEMET be taken at regular intervals according to the schedule outlined by the physician. The patient should be cautioned not to change the prescribed dosage regimen and not to add any additional antiparkinson medications, including other carbidopa levodopa preparations, without first consulting the physician.

Patients should be advised that sometimes a 'wearing-off' effect may occur at the end of the dosing interval. The physician should be notified if such response poses a problem to lifestyle.

Patients should be advised that occasionally, dark color (red, brown, or black) may appear in saliva, urine, or sweat after ingestion of SINEMET. Although the color appears to be clinically insignificant, garments may become discolored.

The patient should be advised that a change in diet to foods that are high in protein may delay the absorption of levodopa and may reduce the amount taken up in the circulation. Excessive acidity also delays stomach emptying, thus delaying the absorption of levodopa. Iron salts (such as in multivitamin tablets) may also reduce the amount of levodopa available to the body. The above factors may reduce the clinical effectiveness of the levodopa or carbidopa levodopa therapy.

Patients should be alerted to the possibility of sudden onset of sleep during daily activities, in some cases without awareness or warning signs, when they are taking dopaminergic agents, including levodopa. Patients should be advised to exercise caution while driving or operating machinery and that if they have experienced somnolence and/or sudden sleep onset, they must refrain from these activities. (See WARNINGS, Falling Asleep During Activities of Daily Living and Somnolence.)

There have been reports of patients experiencing intense urges to gamble, increased sexual urges, and other intense urges, and the inability to control these urges while taking one or more of the medications that increase central dopaminergic tone and that are generally used for the treatment of Parkinson's disease, including SINEMET. Although it is not proven that the medications caused these events, these urges were reported to have stopped in some cases when the dose was reduced or the medication was stopped. Prescribers should ask patients about the development of new or increased gambling urges, sexual urges or other urges while being treated with SINEMET. Patients should inform their physician if they experience new or increased gambling urges, increased sexual urges, or other intense urges while taking SINEMET. Physicians should consider dose reduction or stopping the medication if a patient develops such urges while taking SINEMET (See PRECAUTIONS, Impulse Control / Compulsive Behaviors).

Laboratory Tests

Abnormalities in laboratory tests may include elevations of liver function tests such as alkaline phosphatase, SGOT (AST), SGPT (ALT), lactic dehydrogenase (LDH), and bilirubin. Abnormalities in blood urea nitrogen (BUN) and positive Coombs test have also been reported. Commonly, levels of blood urea nitrogen, creatinine, and uric acid are lower during administration of SINEMET than with levodopa.

SINEMET may cause a false-positive reaction for urinary ketone bodies when a test tape is used for determination of ketonuria. This reaction will not be altered by boiling the urine specimen. False-negative tests may result with the use of glucose-oxidase methods of testing for glucosuria.

Cases of falsely diagnosed pheochromocytoma in patients on carbidopa levodopa therapy have been reported very rarely. Caution should be exercised when interpreting the plasma and urine levels of catecholamines and their metabolites in patients on levodopa or carbidopa levodopa therapy.

Drug Interactions

Caution should be exercised when the following drugs are administered concomitantly with SINEMET.

Symptomatic postural hypotension occurred when SINEMET was added to the treatment of a patient receiving antihypertensive drugs. Therefore, when therapy with SINEMET is started, dosage adjustment of the antihypertensive drug may be required.

For patients receiving MAO inhibitors (Type A or B), see CONTRAINDICATIONS. Concomitant therapy with selegiline and carbidopa levodopa may be associated with severe orthostatic hypotension not attributable to carbidopa levodopa alone (see CONTRAINDICATIONS).

There have been rare reports of adverse reactions, including hypertension and dyskinesia, resulting from the concomitant use of tricyclic antidepressants and SINEMET.

Dopamine D_2 receptor antagonists (e.g., phenothiazines, butyrophenones, risperidone) and isoniazid may reduce the therapeutic effects of levodopa. In addition, the beneficial effects of levodopa in Parkinson's disease have been reported to be reversed by phenytoin and papaverine. Patients taking these drugs with SINEMET should be carefully observed for loss of therapeutic response.

Use of SINEMET with dopamine-depleting agents (e.g., reserpine and tetrabenazine) or other drugs known to deplete monoamine stores is not recommended.

SINEMET and iron salts or multivitamins containing iron salts should be coadministered with caution. Iron salts can form chelates with levodopa and carbidopa and consequently reduce the bioavailability of carbidopa and levodopa.

Although metoclopramide may increase the bioavailability of levodopa by increasing gastric emptying, metoclopramide may also adversely affect disease control by its dopamine receptor antagonistic properties.

Carcinogenesis, Mutagenesis, Impairment of Fertility

In a two-year bioassay of SINEMET, no evidence of carcinogenicity was found in rats receiving doses of approximately two times the maximum daily human dose of carbidopa and four times the maximum daily human dose of levodopa.

In reproduction studies with SINEMET, no effects on fertility were found in rats receiving doses of approximately two times the maximum daily human dose of carbidopa and four times the maximum daily human dose of levodopa.

Pregnancy

Pregnancy Category C. No teratogenic effects were observed in a study in mice receiving up to 20 times the maximum recommended human dose of SINEMET. There was a decrease in the number of live pups delivered by rats receiving approximately two times the maximum recommended human dose of carbidopa and approximately five times the maximum recommended human dose of levodopa during organogenesis. SINEMET caused both visceral and skeletal malformations in rabbits at all doses and ratios of carbidopa/levodopa tested, which ranged from 10 times/5 times the maximum recommended human dose of carbidopa/levodopa to 20 times/10 times the maximum recommended human dose of carbidopa/levodopa.

There are no adequate or well-controlled studies in pregnant women. It has been reported from individual cases that levodopa crosses the human placental barrier, enters the fetus, and is metabolized. Carbidopa concentrations in fetal tissue appeared to be minimal. Use of SINEMET in women of childbearing potential requires that the anticipated benefits of the drug be weighed against possible hazards to mother and child.

Nursing Mothers

Levodopa has been detected in human milk. Caution should be exercised when SINEMET is administered to a nursing woman.

Pediatric Use

Safety and effectiveness in pediatric patients have not been established. Use of the drug in patients below the age of 18 is not recommended.

Geriatric Use

In the clinical efficacy trials for SINEMET, almost half of the patients were older than 65, but few were older than 75. No overall meaningful differences in safety or effectiveness were observed between these subjects and younger subjects, but greater sensitivity of some older individuals to adverse drug reactions such as hallucinations cannot be ruled out. There is no specific dosing recommendation based upon clinical pharmacology data as SINEMET is titrated as tolerated for clinical effect.

ADVERSE REACTIONS

The most common adverse reactions reported with SINEMET have included dyskinesias, such as choreiform, dystonic, and other involuntary movements, and nausea.

The following other adverse reactions have been reported with SINEMET:

Body as a Whole

Chest pain, asthenia.

Cardiovascular

Cardiac irregularities, hypotension, orthostatic effects including orthostatic hypotension, hypertension, syncope, phlebitis, palpitation.

Gastrointestinal

Dark saliva, gastrointestinal bleeding, development of duodenal ulcer, anorexia, vomiting, diarrhea, constipation, dyspepsia, dry mouth, taste alterations.

Hematologic

Agranulocytosis, hemolytic and non-hemolytic anemia, thrombocytopenia, leukopenia.

Hypersensitivity

Angioedema, urticaria, pruritus, Henoch-Schönlein purpura, bullous lesions (including pemphigus-like reactions).

Musculoskeletal

Back pain, shoulder pain, muscle cramps.

Nervous System/Psychiatric

Psychotic episodes including delusions, hallucinations, and paranoid ideation, bradykinetic episodes ("on-off" phenomenon), confusion, agitation, dizziness, somnolence, dream abnormalities including nightmares, insomnia, paresthesia, headache, depression with or without development of suicidal tendencies, dementia, pathological gambling, increased libido including hypersexuality, impulse control symptoms. Convulsions also have occurred; however, a causal relationship with SINEMET has not been established.

Respiratory

Dyspnea, upper respiratory infection.

Skin

Rash, increased sweating, alopecia, dark sweat.

Urogenital

Urinary tract infection, urinary frequency, dark urine.

Laboratory Tests

Decreased hemoglobin and hematocrit; abnormalities in alkaline phosphatase, SGOT (AST), SGPT (ALT), LDH, bilirubin, BUN, Coombs test; elevated serum glucose; white blood cells, bacteria, and blood in the urine.

Other adverse reactions that have been reported with levodopa alone and with various carbidopa levodopa formulations, and may occur with SINEMET are:

Body as a Whole

Abdominal pain and distress, fatigue.

Cardiovascular
Myocardial infarction.
Gastrointestinal
Gastrointestinal pain, dysphagia, sialorrhea, flatulence, bruxism, burning sensation of the tongue, heartburn, hiccups.
Metabolic
Edema, weight gain, weight loss.
Musculoskeletal
Leg pain.
Nervous System/Psychiatric
Ataxia, extrapyramidal disorder, falling, anxiety, gait abnormalities, nervousness, decreased mental acuity, memory impairment, disorientation, euphoria, blepharospasm (which may be taken as an early sign of excess dosage; consideration of dosage reduction may be made at this time), trismus, increased tremor, numbness, muscle twitching, activation of latent Horner's syndrome, peripheral neuropathy.
Respiratory
Pharyngeal pain, cough.
Skin
Malignant melanoma (see also CONTRAINDICATIONS), flushing.
Special Senses
Oculogyric crises, diplopia, blurred vision, dilated pupils.
Urogenital
Urinary retention, urinary incontinence, priapism.
Miscellaneous
Bizarre breathing patterns, faintness, hoarseness, malaise, hot flashes, sense of stimulation.
Laboratory Tests
Decreased white blood cell count and serum potassium; increased serum creatinine and uric acid; protein and glucose in urine.

OVERDOSAGE

Management of acute overdosage with SINEMET is the same as management of acute overdosage with levodopa. Pyridoxine is not effective in reversing the actions of SINEMET.

General supportive measures should be employed, along with immediate gastric lavage. Intravenous fluids should be administered judiciously and an adequate airway maintained. Electrocardiographic monitoring should be instituted and the patient carefully observed for the development of arrhythmias; if required, appropriate antiarrhythmic therapy should be given. The possibility that the patient may have taken other drugs as well as SINEMET should be taken into consideration. To date, no experience has been reported with dialysis; hence, its value in overdosage is not known.

Based on studies in which high doses of levodopa and/or carbidopa were administered, a significant proportion of rats and mice given single oral doses of levodopa of approximately 1500-2000 mg/kg are expected to die. A significant proportion of infant rats of both sexes are expected to die at a dose of 800 mg/kg. A significant proportion of rats are expected to die after treatment with similar doses of carbidopa. The addition of carbidopa in a 1:10 ratio with levodopa increases the dose at which a significant proportion of mice are expected to die to 3360 mg/kg.

DOSAGE AND ADMINISTRATION

The optimum daily dosage of SINEMET must be determined by careful titration in each patient. SINEMET tablets are available in a 1:4 ratio of carbidopa to levodopa (SINEMET 25-100) as well as 1:10 ratio (SINEMET 25-250 and SINEMET 10-100). Tablets of the two ratios may be given separately or combined as needed to provide the optimum dosage.

Studies show that peripheral dopa decarboxylase is saturated by carbidopa at approximately 70 to 100 mg a day. Patients receiving less than this amount of carbidopa are more likely to experience nausea and vomiting.

Usual Initial Dosage

Dosage is best initiated with one tablet of SINEMET 25-100 three times a day. This dosage schedule provides 75 mg of carbidopa per day. Dosage may be increased by one tablet every day or every other day, as necessary, until a dosage of eight tablets of SINEMET 25-100 a day is reached.

If SINEMET 10-100 is used, dosage may be initiated with one tablet three or four times a day. However, this will not provide an adequate amount of carbidopa for many patients. Dosage may be increased by one tablet every day or every other day until a total of eight tablets (2 tablets q.i.d.) is reached.

How to Transfer Patients from Levodopa

Levodopa must be discontinued at least twelve hours before starting SINEMET. A daily dosage of SINEMET should be chosen that will provide approximately 25% of the previous levodopa dosage. Patients who are taking less than 1500 mg of levodopa a day should be started on one tablet of SINEMET 25-100 three or four times a day. The suggested starting dosage for most patients taking more than 1500 mg of levodopa is one tablet of SINEMET 25-250 three or four times a day.

Maintenance

Therapy should be individualized and adjusted according to the desired therapeutic response. At least 70 to 100 mg of carbidopa per day should be provided. When a greater proportion of carbidopa is required, one tablet of SINEMET 25-100 may be substituted for each tablet of SINEMET 10-100. When more levodopa is required, SINEMET 25-250 should be substituted for SINEMET 25-100 or SINEMET 10-100. If necessary, the dosage of carbidopa levodopa 25-250 may be increased by one-half or one tablet every day or every other day to a maximum of eight tablets a day. Experience with total daily dosages of carbidopa greater than 200 mg is limited.

Because both therapeutic and adverse responses occur more rapidly with SINEMET than with levodopa alone, patients should be monitored closely during the dose adjustment period. Specifically, involuntary movements will occur more rapidly with SINEMET than with levodopa. The occurrence of involuntary movements may require dosage reduction. Blepharospasm may be a useful early sign of excess dosage in some patients.

Addition of Other Antiparkinsonian Medications

Standard drugs for Parkinson's disease, other than levodopa without a decarboxylase inhibitor, may be used concomitantly while SINEMET is being administered, although dosage adjustments may be required.

Interruption of Therapy

Sporadic cases of hyperpyrexia and confusion have been associated with dose reductions and withdrawal of SINEMET. Patients should be observed carefully if abrupt reduction or discontinuation of SINEMET is required, especially if the patient is receiving neuroleptics. (See WARNINGS.)

If general anesthesia is required, SINEMET may be continued as long as the patient is permitted to take fluids and medication by mouth. If therapy is interrupted temporarily, the patient should be observed for symptoms resembling NMS, and the usual daily dosage may be administered as soon as the patient is able to take oral medication.

HOW SUPPLIED

No. 3916A — SINEMET 25-100 Tablets are yellow, round, uncoated tablets, that are coded "650" on one side and plain on the other. They are supplied as follows:

NDC 0006-3916-68 bottles of 100.

No. 3915 — SINEMET 10-100 Tablets are light dapple-blue, round, uncoated tablets, that are coded "647" on one side and plain on the other. They are supplied as follows:

NDC 0006-3915-68 bottles of 100.

No. 3917 — SINEMET 25-250 Tablets are light dapple-blue, round, uncoated tablets, that are coded "654" on one side and plain on the other. They are supplied as follows:

NDC 0006-3917-68 bottles of 100.

Storage and Handling

Store at 25°C (77°F), excursions permitted to 15-30°C (59-86°F) [see USP Controlled Room Temperature]. Store in a tightly closed container, protected from light and moisture.

Dispense in a tightly closed, light-resistant container.

Manufactured for: Merck Sharp & Dohme Corp., a subsidiary of
MERCK & CO., INC., Whitehouse Station, NJ 08889, USA

Manufactured by:
Mylan Pharmaceuticals, Inc.
Morgantown, WV 26505, USA

Copyright © 1996-2014 Merck Sharp & Dohme Corp., a subsidiary of **Merck & Co., Inc.**
All rights reserved.

Revised: 07/2014

uspi-mk0295b-t-1407r003

Rx Only

APPENDIX B. INSTRUCTIONS FOR DOSE CONVERSION TO IPX203

The goal of the dose conversion period is to establish a dosing regimen for IPX203 that minimizes "Off" time without causing troublesome dyskinesias.

The initial dose of IPX203 is based on the subject's most frequent IR CD-LD dose established during the 3-week IR CD-LD dose adjustment period.

Table B-1 Recommended Starting IPX203 LD Dosing Regimen Based on the Dosing Regimen of IR CD-LD at the End of the Dose Adjustment Period

Most Frequent IR CD-LD Unit Dose (mg)	Recommended Starting IPX203 Daily Dosing Regimen CD-LD (mg) Every 8 Hours
25-100[a]	70-280 mg (2 × 35-140 mg)
>25-100 – 37.5-150	105-420 mg (3 × 35-140 mg)
>37.5-150 – 50-200	140-560 mg (4 × 35-140 mg)
>50-200	175-700 mg (5 × 35-140 mg)

[a] Subjects who are on a total daily dose of less than 125-500 mg CD-LD from IR CD-LD should be advised to initially take IPX203 every 12 hours. The dosing interval may be reduced to approximately every 8 hours if the subject does not achieve an acceptable duration of effect.

Conversion Instructions:

1. Convert the subject's most frequent daily dose of IR CD-LD to the corresponding dose of IPX203 according to the above table. It is recommended that the subject takes IPX203 doses approximately every 8 hours apart (for example, a subject may take IPX203 at 6 AM, 2 PM, and 10 PM). Some subjects may benefit from a shorter or longer dosing interval. The dosing interval may vary but should not be more frequent than every 6 hours. The maximum recommended daily dose of IPX203 is 600-2400 mg CD-LD.
2. Subjects who are on a total daily dose of less than 125-500 mg CD-LD from IR CD-LD should be advised to initially take IPX203 every 12 hours. The dosing interval may be reduced to approximately every 8 hours if the subject does not achieve an acceptable duration of effect.
3. The Investigator or their staff are advised to be in telephone contact with the subject, especially during the initial dose conversion to assess the need for dosage adjustment with the goal of minimizing "Off" time without causing troublesome dyskinesia or other dopaminergic side effects. Calls to the subject can be reduced appropriately when the subject reaches a stable dosing regimen.
4. If dose adjustment is necessary, consider the following options recognizing that the number of capsules at each dose may be varied to achieve an optimal response.

   a. If turning “On” is slow following the first morning dose, consider taking the morning IPX203 dose in the fasted state and/or increasing the dose by one capsule (35-140 mg IPX203 CD-LD).
   b. If turning “On” is slow later in the day or to reduce “end-of-dose” “Off” time, consider increasing the dose by one capsule (35-140 mg IPX203 CD-LD) before reducing the dosing interval.

5. In case of troublesome dyskinesias, use the following guidelines:
   a. Consider reducing the dose by one capsule (35-140 mg IPX203 CD-LD).
   b. Consider increasing the dosing interval.
6. The subject must be on a stable dosing regimen of IPX203 (no change in dose or in dosing frequency) for at least 5 days prior to Visit 4 (randomization).

APPENDIX C. UNITED KINGDOM PARKINSON'S DISEASE SOCIETY BRAIN BANK DIAGNOSTIC CRITERIA FOR PARKINSON'S DISEASE

**Step 1: Diagnosis of Parkinsonism**
Bradykinesia **and** at least one of the following:<br>• Muscular rigidity<br>• 4–6 Hz resting tremor<br>• postural instability not caused by primary visual, vestibular, cerebellar or Proprioceptive dysfunction
**Step 2: Features tending to exclude Parkinson's disease as the cause of Parkinsonism**
• History of repeated strokes with stepwise progression of parkinsonian features<br>• History of repeated head injury<br>• History of definite encephalitis<br>• Neuroleptic treatment at onset of symptoms<br>• >1 affected relatives<br>• Sustained remission<br>• Strictly unilateral features after 3 years<br>• Supranuclear gaze palsy<br>• Cerebellar signs<br>• Early severe autonomic involvement<br>• Early severe dementia with disturbances of memory, language and praxis<br>• Babinski's sign<br>• Presence of a cerebral tumour or communicating hydrocephalus on computed tomography scan<br>• Negative response to large doses of levodopa (if malabsorption excluded)<br>• MPTP exposure
**Step 3: Features that support a diagnosis of Parkinson's disease (three or more required for diagnosis of definite Parkinson's disease)**
• Unilateral onset<br>• Rest tremor present<br>• Progressive disorder<br>• Persistent asymmetry affecting the side of onset most<br>• Excellent (70–100%) response to levodopa<br>• Severe levodopa-induced chorea<br>• Levodopa response for ≥5 years<br>• Clinical course of ≥10 years

APPENDIX D. MONTREAL COGNITIVE ASSESSMENT (MOCA)

Montreal Cognitive Assessment
(MoCA)

Administration and Scoring Instructions

The Montreal Cognitive Assessment (MoCA) was designed as a rapid screening instrument for mild cognitive dysfunction. It assesses different cognitive domains: attention and concentration, executive functions, memory, language, visuoconstructional skills, conceptual thinking, calculations, and orientation. Time to administer the MoCA is approximately 10 minutes. The total possible score is 30 points; a score of 26 or above is considered normal.

1. Alternating Trail Making:

Administration: The examiner instructs the subject: *"Please draw a line, going from a number to a letter in ascending order. Begin here* [point to (1)] *and draw a line from 1 then to A then to 2 and so on. End here* [point to (E)]."

Scoring: Allocate one point if the subject successfully draws the following pattern:
1 –A- 2- B- 3- C- 4- D- 5- E, without drawing any lines that cross. Any error that is not immediately self-corrected earns a score of 0.

2. Visuoconstructional Skills (Cube):

Administration: The examiner gives the following instructions, pointing to the **cube**: "*Copy this drawing as accurately as you can, in the space below*".

Scoring: One point is allocated for a correctly executed drawing.
• Drawing must be three-dimensional
• All lines are drawn
• No line is added
• Lines are relatively parallel and their length is similar (rectangular prisms are accepted)

A point is not assigned if any of the above-criteria are not met.

3. Visuoconstructional Skills (Clock):

Administration: Indicate the right third of the space and give the following instructions: *"Draw a clock. Put in all the numbers and set the time to 10 past 11".*

Scoring: One point is allocated for each of the following three criteria:
• Contour (1 pt.): the clock face must be a circle with only minor distortion acceptable (e.g., slight imperfection on closing the circle);
• Numbers (1 pt.): all clock numbers must be present with no additional numbers; numbers must be in the correct order and placed in the approximate quadrants on the clock face; Roman numerals are acceptable; numbers can be placed outside the circle contour;
• Hands (1 pt.): there must be two hands jointly indicating the correct time; the hour hand must be clearly shorter than the minute hand; hands must be centred within the clock face with their junction close to the clock centre.

A point is not assigned for a given element if any of the above-criteria are not met.

© Z. Nasreddine MD

4. Naming:

Administration: Beginning on the left, point to each figure and say: *"Tell me the name of this animal".*

Scoring: One point each is given for the following responses: (1) lion (2) rhinoceros or rhino (3) camel or dromedary.

5. Memory:

Administration: The examiner reads a list of 5 words at a rate of one per second, giving the following instructions: *"This is a memory test. I am going to read a list of words that you will have to remember now and later on. Listen carefully. When I am through, tell me as many words as you can remember. It doesn't matter in what order you say them".* Mark a check in the allocated space for each word the subject produces on this first trial. When the subject indicates that (s)he has finished (has recalled all words), or can recall no more words, read the list a second time with the following instructions: *"I am going to read the same list for a second time. Try to remember and tell me as many words as you can, including words you said the first time."* Put a check in the allocated space for each word the subject recalls after the second trial.
At the end of the second trial, inform the subject that (s)he will be asked to recall these words again by saying, *"I will ask you to recall those words again at the end of the test."*

Scoring: No points are given for Trials One and Two.

6. Attention:

Forward Digit Span: Administration: Give the following instruction: "*I am going to say some numbers and when I am through, repeat them to me exactly as I said them*". Read the five number sequence at a rate of one digit per second.

Backward Digit Span: Administration: Give the following instruction: "*Now I am going to say some more numbers, but when I am through you must repeat them to me in the backwards order.*" Read the three number sequence at a rate of one digit per second.

Scoring: Allocate one point for each sequence correctly repeated, (*N.B.*: the correct response for the backwards trial is 2-4-7).

Vigilance: Administration: The examiner reads the list of letters at a rate of one per second, after giving the following instruction: "*I am going to read a sequence of letters. Every time I say the letter A, tap your hand once. If I say a different letter, do not tap your hand*".

Scoring: Give one point if there is zero to one errors (an error is a tap on a wrong letter or a failure to tap on letter A).

© Z. Nasreddine MD *www.mocatest.org*

Serial 7s: Administration: The examiner gives the following instruction: *"Now, I will ask you to count by subtracting seven from 100, and then, keep subtracting seven from your answer until I tell you to stop."* Give this instruction twice if necessary.

Scoring: This item is scored out of 3 points. Give no (0) points for no correct subtractions, 1 point for one correction subtraction, 2 points for two-to-three correct subtractions, and 3 points if the participant successfully makes four or five correct subtractions. Count each correct subtraction of 7 beginning at 100. Each subtraction is evaluated independently; that is, if the participant responds with an incorrect number but continues to correctly subtract 7 from it, give a point for each correct subtraction. For example, a participant may respond "92 – 85 – 78 – 71 – 64" where the "92" is incorrect, but all subsequent numbers are subtracted correctly. This is one error and the item would be given a score of 3.

7. **Sentence repetition:**

Administration: The examiner gives the following instructions: *"I am going to read you a sentence. Repeat it after me, exactly as I say it* [pause]: ***I only know that John is the one to help today."*** Following the response, say: *"Now I am going to read you another sentence. Repeat it after me, exactly as I say it* [pause]: ***The cat always hid under the couch when dogs were in the room."***

Scoring: Allocate 1 point for each sentence correctly repeated. Repetition must be exact. Be alert for errors that are omissions (e.g., omitting "only", "always") and substitutions/additions (e.g., "John is the one who helped today;" substituting "hides" for "hid", altering plurals, etc.).

8. **Verbal fluency:**

Administration: The examiner gives the following instruction: *"Tell me as many words as you can think of that begin with a certain letter of the alphabet that I will tell you in a moment. You can say any kind of word you want, except for proper nouns (like Bob or Boston), numbers, or words that begin with the same sound but have a different suffix, for example, love, lover, loving. I will tell you to stop after one minute. Are you ready?* [Pause] *Now, tell me as many words as you can think of that begin with the letter F.* [time for 60 sec]. *Stop."*

Scoring: Allocate one point if the subject generates 11 words or more in 60 sec. Record the subject's response in the bottom or side margins.

9. **Abstraction:**

Administration: The examiner asks the subject to explain what each pair of words has in common, starting with the example: *"Tell me how an orange and a banana are alike".* If the subject answers in a concrete manner, then say only one additional time: *"Tell me another way in which those items are alike".* If the subject does not give the appropriate response *(fruit)*, say, *"Yes, and they are also both fruit."* Do not give any additional instructions or clarification. After the practice trial, say: *"Now, tell me how a train and a bicycle are alike".* Following the response, administer the second trial, saying: *"Now tell me how a ruler and a watch are alike".* Do not give any additional instructions or prompts.

Scoring: Only the last two item pairs are scored. Give 1 point to each item pair correctly answered. The following responses are acceptable:
Train-bicycle = means of transportation, means of travelling, you take trips in both;
Ruler-watch = measuring instruments, used to measure.
The following responses are **not** acceptable: Train-bicycle = they have wheels; Ruler-watch = they have numbers.

10. Delayed recall:

Administration: The examiner gives the following instruction: *"I read some words to you earlier, which I asked you to remember. Tell me as many of those words as you can remember."* Make a check mark (√) for each of the words correctly recalled spontaneously without any cues, in the allocated space.

Scoring: **Allocate 1 point for each word recalled freely without any cues.**

Optional:
Following the delayed free recall trial, prompt the subject with the semantic category cue provided below for any word not recalled. Make a check mark (√) in the allocated space if the subject remembered the word with the help of a category or multiple-choice cue. Prompt all non-recalled words in this manner. If the subject does not recall the word after the category cue, give him/her a multiple choice trial, using the following example instruction, *"Which of the following words do you think it was, NOSE, FACE, or HAND?"*
Use the following category and/or multiple-choice cues for each word, when appropriate:

FACE:	category cue: part of the body	multiple choice: nose, face, hand
VELVET:	category cue: type of fabric	multiple choice: denim, cotton, velvet
CHURCH:	category cue: type of building	multiple choice: church, school, hospital
DAISY:	category cue: type of flower	multiple choice: rose, daisy, tulip
RED:	category cue: a colour	multiple choice: red, blue, green

Scoring: **No points are allocated for words recalled with a cue.** A cue is used for clinical information purposes only and can give the test interpreter additional information about the type of memory disorder. For memory deficits due to retrieval failures, performance can be improved with a cue. For memory deficits due to encoding failures, performance does not improve with a cue.

11. Orientation:

Administration: The examiner gives the following instructions: "Tell me the date today". If the subject does not give a complete answer, then prompt accordingly by saying: *"Tell me the [year, month, exact date, and day of the week]."* Then say: *"Now, tell me the name of this place, and which city it is in."*

Scoring: Give one point for each item correctly answered. The subject must tell the exact date and the exact place (name of hospital, clinic, office). No points are allocated if subject makes an error of one day for the day and date.

TOTAL SCORE: Sum all subscores listed on the right-hand side. Add one point for an individual who has 12 years or fewer of formal education, for a possible maximum of 30 points. A final total score of 26 and above is considered normal.

© Z. Nasreddine MD
www.mocatest.org

NAME :
Education : Date of birth :
Sex : DATE :

MONTREAL COGNITIVE ASSESSMENT (MOCA)

Version 7.1 Original Version

VISUOSPATIAL / EXECUTIVE

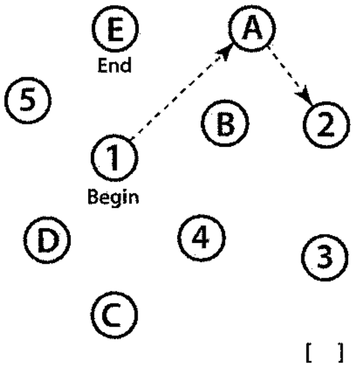

[ ]

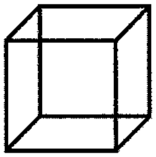

Copy cube

[ ]

Draw CLOCK (Ten past eleven)
(3 points)

[ ] Contour [ ] Numbers [ ] Hands

POINTS: ___/5

NAMING

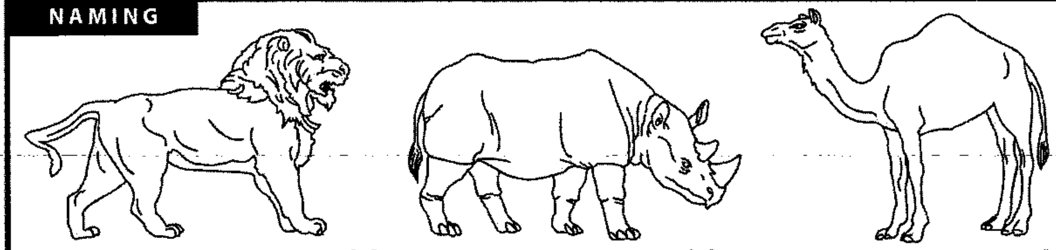

[ ] [ ] [ ]

___/3

MEMORY Read list of words, subject must repeat them. Do 2 trials, even if 1st trial is successful. Do a recall after 5 minutes.

	FACE	VELVET	CHURCH	DAISY	RED
1st trial					
2nd trial					

No points

ATTENTION Read list of digits (1 digit/ sec.).

Subject has to repeat them in the forward order [ ] 2 1 8 5 4
Subject has to repeat them in the backward order [ ] 7 4 2

___/2

Read list of letters. The subject must tap with his hand at each letter A. No points if ≥ 2 errors

[ ] F B A C M N A A J K L B A F A K D E A A A J A M O F A A B

___/1

Serial 7 subtraction starting at 100 [ ] 93 [ ] 86 [ ] 79 [ ] 72 [ ] 65

4 or 5 correct subtractions: **3 pts**, 2 or 3 correct: **2 pts**, 1 correct: **1 pt**, 0 correct: **0 pt**

___/3

LANGUAGE Repeat : I only know that John is the one to help today. [ ]
The cat always hid under the couch when dogs were in the room. [ ]

___/2

Fluency / Name maximum number of words in one minute that begin with the letter F [ ] ____ (N ≥ 11 words)

___/1

ABSTRACTION Similarity between e.g. banana - orange = fruit [ ] train – bicycle [ ] watch - ruler

___/2

DELAYED RECALL

	Has to recall words WITH NO CUE	FACE [ ]	VELVET [ ]	CHURCH [ ]	DAISY [ ]	RED [ ]	Points for UNCUED recall only
Optional	Category cue						
	Multiple choice cue						

___/5

ORIENTATION [ ] Date [ ] Month [ ] Year [ ] Day [ ] Place [ ] City

___/6

© Z.Nasreddine MD **www.mocatest.org** Normal ≥26 / 30

TOTAL ___/30

Add 1 point if ≤ 12 yr edu

Administered by:________________________________

APPENDIX E. MOVEMENT DISORDERS SOCIETY VERSION OF UNIFIED PARKINSON'S DISEASE RATING SCALE (MDS-UPDRS)

MDS-UPDRS Permissions

Permission is required to use the MDS-developed Rating Scales (with the exception of personal/individual use). Reproduction, translation, modification, sale, or distribution of any portion of the MDS Rating Scales is strictly prohibited. MDS Rating Scales may not be incorporated into clinical trials, training or certification programs or materials, software programs, or otherwise except through use of the Permissions Request Form and payment of applicable fees.

Continue to p. 2 to view the MDS-UPDRS

MDS-UPDRS

The *Movement* Disorder Society (MDS)-sponsored new version of the UPDRS is founded on the critique that was formulated by the Task Force for Rating Scales in Parkinson's disease (*Mov Disord* 2003;18:738-750). Thereafter, the MDS recruited a Chairperson to organize a program to provide the Movement Disorder community with a new version of the UPDRS that would maintain the overall format of the original UPDRS, but address issues identified in the critique as weaknesses and ambiguities. The Chairperson identified subcommittees with chairs and members. Each part was written by the appropriate subcommittee members and then reviewed and ratified by the entire group. These members are listed below.

The MDS-UPDRS has four parts: Part I (non-motor experiences of daily living), Part II (motor experiences of daily living, Part III (motor examination) and Part IV (motor complications). Part I has two components: IA concerns a number of behaviors that are assessed by the investigator with all pertinent information from patients and caregivers, and IB is completed by the patient with or without the aid of the caregiver, but independently of the investigator. These sections can, however, be reviewed by the rater to ensure that all questions are answered clearly and the rater can help explain any perceived ambiguities. Part II is designed to be a self-administered questionnaire like Part IB, but can be reviewed by the investigator to ensure completeness and clarity. Of note, the official versions of Part IA, Part IB and Part II of the MDS-UPDRS do not have separate on or off ratings. However, for individual programs or protocols the same questions can be used separately for on and off. Part III has instructions for the rater to give or demonstrate to the patient; it is completed by the rater. Part IV has instructions for the rater and also instructions to be read to the patient. This part integrates patient-derived information with the rater's clinical observations and judgments and is completed by the rater.

The authors of this new version are:

Chairperson: Christopher G. Goetz
Part I: Werner Poewe (chair), Bruno Dubois, Anette Schrag
Part II: Matthew B. Stern (chair), Anthony E. Lang, Peter A. LeWitt
Part III: Stanley Fahn (chair), Joseph Jankovic, C. Warren Olanow
Part IV: Pablo Martinez-Martin (chair), Andrew Lees, Olivier Rascol, Bob van Hilten
Development Standards: Glenn T. Stebbins (chair), Robert Holloway, David Nyenhuis
Appendices: Cristina Sampaio (chair), Richard Dodel, Jaime Kulisevsky
Statistical Testing: Barbara Tilley (chair), Sue Leurgans, Jean Teresi,
Consultant: Stephanie Shaftman, Nancy LaPelle

Contact person: Christopher G. Goetz, MD
Rush University Medical Center
1725 W. Harrison Street, Suite 755
Chicago, IL USA 60612

Telephone 312-942-8016
Email: cgoetz@rush.edu

July 1, 2008

Copyright © 2008 Movement Disorder Society. All rights reserved.
This chart may not be copied, distributed or otherwise used in whole or in part without prior written consent of the Movement Disorder Society.

Part I: Non-Motor Aspects of Experiences of Daily Living (nM-EDL)

Overview: This portion of the scale assesses the non-motor impact of Parkinson's disease (PD) on patients' experiences of daily living. There are 13 questions. Part 1A is administered by the rater (six questions) and focuses on complex behaviors. Part 1B is a component of the self-administered Patient Questionnaire that covers seven questions on non-motor experiences of daily living.

Part 1A:
In administering Part IA, the examiner should use the following guidelines:

1. Mark at the top of the form the primary data source as patient, caregiver, or patient and caregiver in equal proportion.
2. The response to each item should refer to a period encompassing the prior week including the day on which the information is collected.
3. All items must have an integer rating (no half points, no missing scores). In the event that an item does not apply or cannot be rated (e.g., amputee who cannot walk), the item is marked UR for Unable to Rate.
4. The answers should reflect the usual level of function and words such as "usually", "generally", "most of the time" can be used with patients.
5. Each question has a text for you to read (Instructions to patients/caregiver). After that statement, you can elaborate and probe based on the target symptoms outlined in the Instructions to examiner. You should NOT READ the RATING OPTIONS to the patient/caregiver, because these are written in medical terminology. From the interview and probing, you will use your medical judgment to arrive at the best response.
6. Patients may have co-morbidities and other medical conditions that can affect their function. You and the patient must rate the problem as it exists and do not attempt to separate elements due to Parkinson's disease from other conditions.

EXAMPLE OF NAVIGATING THROUGH THE RESPONSE OPTIONS FOR PART 1A

Suggested strategies for obtaining the most accurate answer:
After reading the instructions to the patient, you will need to probe the entire domain under discussion to determine Normal vs. problematic: If your questions do not identify any problem in this domain, record 0 and move on to the next question.

If your questions identify a problem in this domain, you should work next with a reference anchor at the mid-range (option 2 or Mild) to find out if the patient functions at this level, better or worse. You will not be reading the choices of responses to the patient as the responses use clinical terminology. You will be asking enough probing questions to determine the response that should be coded.

Work up and down the options with the patient to identify the most accurate response, giving a final check by excluding the options above and below the selected response.

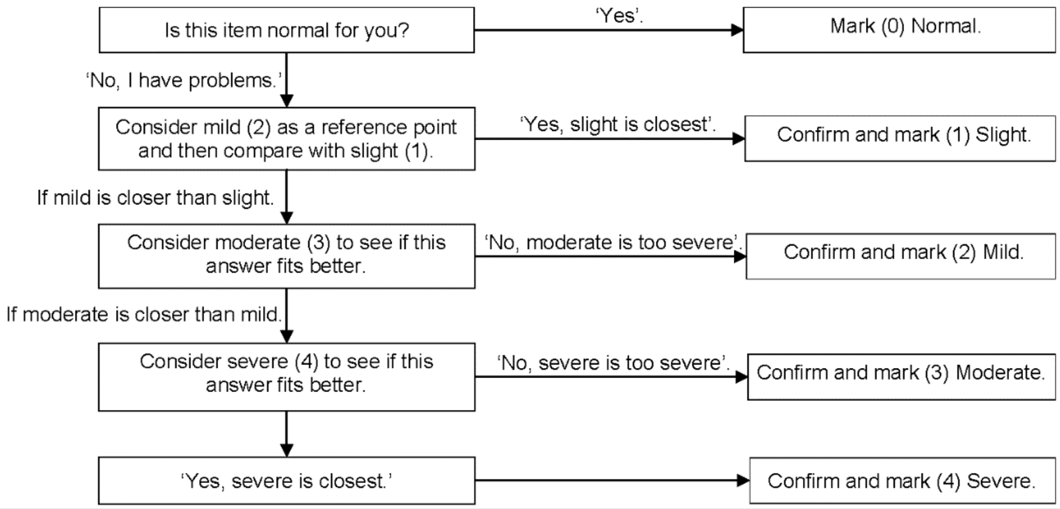

July 1, 2008 *Copyright © 2008 Movement Disorder Society. All rights reserved.* Page 2
This chart may not be copied, distributed or otherwise used in whole or in part without prior written consent of the Movement Disorder Society.

___ ___ ___ ___ ___ ___ ___ ___ Patient Name or Subject ID	___ ___ ___ Site ID	___ ___ - ___ ___ - ___ ___ ___ ___ (mm-dd-yyyy) Assessment Date	___ ___ ___ Investigator's Initials

MDS UPDRS

Part I: Non-Motor Aspects of Experiences of Daily Living (nM-EDL)

Part 1A: Complex behaviors: [completed by rater]

Primary source of information:

☐ Patient ☐ Caregiver ☐ Patient and Caregiver in Equal Proportion

To be read to the patient: I am going to ask you six questions about behaviors that you may or may not experience. Some questions concern common problems and some concern uncommon ones. If you have a problem in one of the areas, please choose the best response that describes how you have felt MOST OF THE TIME during the PAST WEEK. If you are not bothered by a problem, you can simply respond NO. I am trying to be thorough, so I may ask questions that have nothing to do with you.

1.1 COGNITIVE IMPAIRMENT — **SCORE** ☐

Instructions to examiner: Consider all types of altered level of cognitive function including cognitive slowing, impaired reasoning, memory loss, deficits in attention and orientation. Rate their impact on activities of daily living as perceived by the patient and/or caregiver.

Instructions to patients [and caregiver]: Over the past week have you had problems remembering things, following conversations, paying attention, thinking clearly, or finding your way around the house or in town? [If yes, examiner asks patient or caregiver to elaborate and probes for information]

0: Normal: No cognitive impairment.

1: Slight: Impairment appreciated by patient or caregiver with no concrete interference with the patient's ability to carry out normal activities and social interactions.

2: Mild: Clinically evident cognitive dysfunction, but only minimal interference with the patient's ability to carry out normal activities and social interactions.

3: Moderate: Cognitive deficits interfere with but do not preclude the patient's ability to carry out normal activities and social interactions.

4: Severe: Cognitive dysfunction precludes the patient's ability to carry out normal activities and social interactions.

July 1, 2008

Copyright © 2008 Movement Disorder Society. All rights reserved.
This chart may not be copied, distributed or otherwise used in whole or in part without prior written consent of the Movement Disorder Society.

Page 3

	SCORE
**1.2 HALLUCINATIONS AND PSYCHOSIS**<br><br>Instructions to examiner: Consider both illusions (misinterpretations of real stimuli) and hallucinations (spontaneous false sensations). Consider all major sensory domains (visual, auditory, tactile, olfactory and gustatory). Determine presence of unformed (for example sense of presence or fleeting false impressions) as well as formed (fully developed and detailed) sensations. Rate the patients insight into hallucinations and identify delusions and psychotic thinking.<br><br>*Instructions to patients [and caregiver]: Over the past week have you seen, heard, smelled or felt things that were not really there?* [If yes, examiner asks patient or caregiver to elaborate and probes for information]<br><br>0: Normal: No hallucinations or psychotic behaviour.<br><br>1: Slight: Illusions or non-formed hallucinations, but patient recognizes them without loss of insight.<br><br>2: Mild: Formed hallucinations independent of environmental stimuli. No loss of insight.<br><br>3: Moderate: Formed hallucinations with loss of insight.<br><br>4: Severe: Patient has delusions or paranoia.	☐
**1.3 DEPRESSED MOOD**<br><br>Instructions to examiner: Consider low mood, sadness, hopelessness, feelings of emptiness or loss of enjoyment. Determine their presence and duration over the past week and rate their interference with the patient's ability to carry out daily routines and engage in social interactions.<br><br>*Instruction to the patient (and caregiver): Over the past week have you felt low, sad, hopeless or unable to enjoy things? If yes, was this feeling for longer than one day at a time? Did it make it difficult for you carry out your usual activities or to be with people?* If yes, examiner asks patient or caregiver to elaborate and probes for information]<br><br>0: Normal: No depressed mood.<br><br>1: Slight: Episodes of depressed mood that are not sustained for more than one day at a time. No interference with patient's ability to carry out normal activities and social interactions.<br><br>2: Mild: Depressed mood that is sustained over days, but without interference with normal activities and social interactions.<br><br>3: Moderate: Depressed mood that interferes with, but does not preclude, the patient's ability to carry out normal activities and social interactions.<br><br>4: Severe: Depressed mood precludes patient's ability to carry out normal activities and social interactions.	☐

July 1, 2008 *Copyright © 2008 Movement Disorder Society. All rights reserved.* Page 4
This chart may not be copied, distributed or otherwise used in whole or in part without prior written consent of the Movement Disorder Society.

	SCORE
**1.4 ANXIOUS MOOD**<br><br><u>Instructions to examiner:</u> Determine nervous, tense, worried or anxious feelings (including panic attacks) over the past week and rate their duration and interference with the patient's ability to carry out daily routines and engage in social interactions.<br><br>*<u>Instructions to patients [and caregiver]:</u> Over the past week have you felt nervous, worried or tense? If yes, was this feeling for longer than one day at a time? Did it make it difficult for you to follow your usual activities or to be with other people*? [If yes, examiner asks patient or caregiver to elaborate and probes for information.]<br><br>0: Normal: No anxious feelings.<br><br>1: Slight: Anxious feelings present but not sustained for more than one day at a time. No interference with patient's ability to carry out normal activities and social interactions.<br><br>2: Mild: Anxious feelings are sustained over more than one day at a time, but without interference with patient's ability to carry out normal activities and social interactions.<br><br>3: Moderate: Anxious feelings interfere with, but do not preclude, the patient's ability to carry out normal activities and social interactions.<br><br>4: Severe: Anxious feelings preclude patient's ability to carry out normal activities and social interactions.	☐
**1.5 APATHY**<br><br><u>Instructions to examiner:</u> Consider level of spontaneous activity, assertiveness, motivation and initiative and rate the impact of reduced levels on performance of daily routines and social interactions. Here the examiner should attempt to distinguish between apathy and similar symptoms that are best explained by depression.<br><br>*<u>Instructions to patients (and caregiver):</u> Over the past week, have you felt indifferent to doing activities or being with people?* If yes, examiner asks patient or caregiver to elaborate and probes for information.]<br><br>0: Normal: No apathy.<br><br>1: Slight: Apathy appreciated by patient and/or caregiver, but no interference with daily activities and social interactions.<br><br>2: Mild: Apathy interferes with isolated activities and social interactions.<br><br>3: Moderate: Apathy interferes with most activities and social interactions.<br><br>4: Severe: Passive and withdrawn, complete loss of initiative.	☐

July 1, 2008 *Copyright © 2008 Movement Disorder Society. All rights reserved.* Page 5
This chart may not be copied, distributed or otherwise used in whole or in part without prior written consent of the Movement Disorder Society.

1.6 FEATURES OF DOPAMINE DYSREGULATION SYNDROME	SCORE
<u>Instructions to examiner</u>: Consider involvement in a variety of activities including atypical or excessive gambling (e.g. casinos or lottery tickets), atypical or excessive sexual drive or interests (e.g., unusual interest in pornography, masturbation, sexual demands on partner), other repetitive activities (e.g. hobbies, dismantling objects, sorting or organizing), or taking extra non-prescribed medication for non-physical reasons (i.e., addictive behavior). Rate the impact of such abnormal activities/behaviors on the patient's personal life and on his family and social relations (including need to borrow money or other financial difficulties like withdrawal of credit cards, major family conflicts, lost time from work, or missed meals or sleep because of the activity).<br>*<u>Instructions to patients [and caregiver]</u>: Over the past week, have you had unusually strong urges that are hard to control? Do you feel driven to do or think about something and find it hard to stop?* [Give patient examples such as gambling, cleaning, using the computer, taking extra medicine, obsessing about food or sex, all depending on the patients.<br><br>0: Normal: No problems present.<br>1: Slight: Problems are present but usually do not cause any difficulties for the patient or family/caregiver.<br>2: Mild: Problems are present and usually cause a few difficulties in the patient's personal and family life.<br>3: Moderate: Problems are present and usually cause a lot of difficulties in the patient's personal and family life.<br>4: Severe: Problems are present and preclude the patient's ability to carry out normal activities or social interactions or to maintain previous standards in personal and family life.	☐

The remaining questions in Part I (Non-motor Experiences of Daily Living) [Sleep, Daytime Sleepiness, Pain and Other Sensation, Urinary Problems, Constipation Problems, Lightheadedness on Standing, and Fatigue] are in the **Patient Questionnaire** along with all questions in Part II [Motor Experiences of Daily Living].

Copyright © 2008 Movement Disorder Society. All rights reserved.

This chart may not be copied, distributed or otherwise used in whole or in part without prior written consent of the Movement Disorder Society.

Patient Questionnaire:

Instructions:

This questionnaire will ask you about your experiences of daily living.

There are 20 questions. We are trying to be thorough, and some of these questions may therefore not apply to you now or ever. If you do not have the problem, simply mark 0 for NO.

Please read each one carefully and read all answers before selecting the one that best applies to you.

We are interested in your average or usual function over the past week including today. Some patients can do things better at one time of the day than at others. However, only one answer is allowed for each question, so please mark the answer that best describes what you can do most of the time.

You may have other medical conditions besides Parkinson's disease. Do not worry about separating Parkinson's disease from other conditions. Just answer the question with your best response.

Use only 0, 1, 2, 3, 4 for answers, nothing else. Do not leave any blanks.

Your doctor or nurse can review the questions with you, but this questionnaire is for patients to complete, either alone or with their caregivers.

Who is filling out this questionnaire (check the best answer):

☐ Patient ☐ Caregiver ☐ Patient and Caregiver in Equal Proportion

Copyright © 2008 Movement Disorder Society. All rights reserved.

This chart may not be copied, distributed or otherwise used in whole or in part without prior written consent of the Movement Disorder Society.

Part I: Non-Motor Aspects of Experiences of Daily Living (nM-EDL)

SCORE

1.7 SLEEP PROBLEMS

Over the past week, have you had trouble going to sleep at night or staying asleep through the night? Consider how rested you felt after waking up in the morning.

0:	Normal:	No problems.
1:	Slight:	Sleep problems are present but usually do not cause trouble getting a full night of sleep.
2:	Mild:	Sleep problems usually cause some difficulties getting a full night of sleep.
3:	Moderate:	Sleep problems cause a lot of difficulties getting a full night of sleep, but I still usually sleep for more than half the night.
4:	Severe:	I usually do not sleep for most of the night.

☐

1.8 DAYTIME SLEEPINESS

Over the past week, have you had trouble staying awake during the daytime?

0:	Normal:	No daytime sleepiness.
1:	Slight:	Daytime sleepiness occurs but I can resist and I stay awake.
2:	Mild:	Sometimes I fall asleep when alone and relaxing. For example, while reading or watching TV.
3:	Moderate:	I sometimes fall asleep when I should not. For example, while eating or talking with other people.
4:	Severe:	I often fall asleep when I should not. For example, while eating or talking with other people.

☐

Copyright © 2008 Movement Disorder Society. All rights reserved.
This chart may not be copied, distributed or otherwise used in whole or in part without prior written consent of the Movement Disorder Society.

SCORE

1.9 PAIN AND OTHER SENSATIONS

Over the past week, have you had uncomfortable feelings in your body like pain, aches tingling or cramps?

0:	Normal:	No uncomfortable feelings.
1:	Slight:	I have these feelings. However, I can do things and be with other people without difficulty.
2:	Mild:	These feelings cause some problems when I do things or am with other people.
3:	Moderate:	These feelings cause a lot of problems, but they do not stop me from doing things or being with other people.
4:	Severe:	These feelings stop me from doing things or being with other people.

☐

1.10 URINARY PROBLEMS

Over the past week, have you had trouble with urine control? For example, an urgent need to urinate, a need to urinate too often, or urine accidents?

0:	Normal:	No urine control problems.
1:	Slight:	I need to urinate often or urgently. However, these problems do not cause difficulties with my daily activities.
2:	Mild:	Urine problems cause some difficulties with my daily activities. However, I do not have urine accidents.
3:	Moderate:	Urine problems cause a lot of difficulties with my daily activities, including urine accidents.
4:	Severe:	I cannot control my urine and use a protective garment or have a bladder tube.

☐

Copyright © 2008 Movement Disorder Society. All rights reserved.

This chart may not be copied, distributed or otherwise used in whole or in part without prior written consent of the Movement Disorder Society.

	SCORE
**1.11 CONSTIPATION PROBLEMS**<br><br>Over the past week have you had constipation troubles that cause you difficulty moving your bowels?<br><br>0: Normal: No constipation.<br><br>1: Slight: I have been constipated. I use extra effort to move my bowels. However, this problem does not disturb my activities or my being comfortable.<br><br>2: Mild: Constipation causes me to have some troubles doing things or being comfortable.<br><br>3: Moderate: Constipation causes me to have a lot of trouble doing things or being comfortable. However, it does not stop me from doing anything.<br><br>4: Severe: I usually need physical help from someone else to empty my bowels.	☐
**1.12 LIGHT HEADEDNESS ON STANDING**<br><br>Over the past week, have you felt faint, dizzy or foggy when you stand up after sitting or lying down?<br><br>0: Normal: No dizzy or foggy feelings.<br><br>1: Slight: Dizzy or foggy feelings occur. However, they do not cause me troubles doing things.<br><br>2: Mild: Dizzy or foggy feelings cause me to hold on to something, but I do not need to sit or lie back down.<br><br>3: Moderate: Dizzy or foggy feelings cause me to sit or lie down to avoid fainting or falling.<br><br>4: Severe: Dizzy or foggy feelings cause me to fall or faint.	☐

Copyright © 2008 Movement Disorder Society. All rights reserved.

This chart may not be copied, distributed or otherwise used in whole or in part without prior written consent of the Movement Disorder Society.

SCORE

1.13 FATIGUE

Over the past week, have you usually felt fatigued? This feeling is not part of being sleepy or sad

0:	Normal:	No fatigue.
1:	Slight:	Fatigue occurs. However it does not cause me troubles doing things or being with people.
2:	Mild:	Fatigue causes me some troubles doing things or being with people.
3:	Moderate:	Fatigue causes me a lot of troubles doing things or being with people. However, it does not stop me from doing anything.
4:	Severe:	Fatigue stops me from doing things or being with people.

☐

Part II: Motor Aspects of Experiences of Daily Living (M-EDL)

2.1 SPEECH

Over the past week, have you had problems with your speech?

0:	Normal:	Not at all (no problems).
1:	Slight:	My speech is soft, slurred or uneven, but it does not cause others to ask me to repeat myself.
2:	Mild:	My speech causes people to ask me to occasionally repeat myself, but not everyday.
3:	Moderate:	My speech is unclear enough that others ask me to repeat myself every day even though most of my speech is understood.
4:	Severe:	Most or all of my speech cannot be understood.

☐

Copyright © 2008 Movement Disorder Society. All rights reserved.

This chart may not be copied, distributed or otherwise used in whole or in part without prior written consent of the Movement Disorder Society.

SCORE

2.2 SALIVA & DROOLING

Over the past week, have you usually had too much saliva during when you are awake or when you sleep?

0: Normal: Not at all (no problems).

1: Slight: I have too much saliva, but do not drool.

2: Mild: I have some drooling during sleep, but none when I am awake.

3: Moderate: I have some drooling when I am awake, but I usually do not need tissues or a handkerchief.

4: Severe: I have so much drooling that I regularly need to use tissues or a handkerchief to protect my clothes.

☐

2.3 CHEWING AND SWALLOWING

Over the past week, have you usually had problems swallowing pills or eating meals? Do you need your pills cut or crushed or your meals to be made soft, chopped or blended to avoid choking?

0: Normal: No problems.

1: Slight: I am aware of slowness in my chewing or increased effort at swallowing, but I do not choke or need to have my food specially prepared.

2: Mild: I need to have my pills cut or my food specially prepared because of chewing or swallowing problems, but I have not choked over the past week.

3: Moderate. I choked at least once in the past week.

4: Severe: Because of chewing and swallowing problems, I need a feeding tube.

☐

Copyright © 2008 Movement Disorder Society. All rights reserved.

This chart may not be copied, distributed or otherwise used in whole or in part without prior written consent of the Movement Disorder Society.

	SCORE
**2.4 EATING TASKS**<br><br>Over the past week, have you usually had troubles handling your food and using eating utensils? For example, do you have trouble handling finger foods or using forks, knifes, spoons, chopsticks?<br><br>0: Normal: Not at all (No problems).<br><br>1: Slight: I am slow, but I do not need any help handling my food and have not had food spills while eating.<br><br>2: Mild: I am slow with my eating and have occasional food spills. I may need help with a few tasks such as cutting meat.<br><br>3: Moderate: I need help with many eating tasks but can manage some alone.<br><br>4: Severe: I need help for most or all eating tasks.	☐
**2.5 DRESSING**<br><br>Over the past week, have you usually had problems dressing? For example, are you slow or do you need help with buttoning, using zippers, putting on or taking off your clothes or jewelry?<br><br>0: Normal: Not at all (no problems).<br><br>1: Slight: I am slow but I do not need help.<br><br>2: Mild: I am slow and need help for a few dressing tasks (buttons, bracelets).<br><br>3: Moderate: I need help for many dressing tasks.<br><br>4: Severe: I need help for most or all dressing tasks.	☐

Copyright © 2008 Movement Disorder Society. All rights reserved.
This chart may not be copied, distributed or otherwise used in whole or in part without prior written consent of the Movement Disorder Society.

	SCORE
**2.6 HYGIENE**<br><br>Over the past week, have you usually been slow or do you need help with washing, bathing, shaving, brushing teeth, combing your hair or with other personal hygiene?<br><br>0: Normal: Not at all (no problems).<br>1: Slight: I am slow but I do not need any help.<br>2: Mild: I need someone else to help me with some hygiene tasks.<br>3: Moderate: I need help for many hygiene tasks.<br>4: Severe: I need help for most or all of my hygiene tasks.	☐
**2.7 HANDWRITING**<br><br>Over the past week, have people usually had trouble reading your handwriting?<br><br>0: Normal: Not at all (no problems).<br>1: Slight: My writing is slow, clumsy or uneven, but all words are clear.<br>2: Mild: Some words are unclear and difficult to read.<br>3: Moderate: Many words are unclear and difficult to read.<br>4: Severe: Most or all words cannot be read.	☐
**2.8 DOING HOBBIES AND OTHER ACTIVITIES**<br><br>Over the past week, have you usually had trouble doing your hobbies or other things that you like to do?<br><br>0: Normal: Not at all (no problems).<br>1: Slight: I am a bit slow but do these activities easily.<br>2: Mild: I have some difficulty doing these activities.<br>3: Moderate: I have major problems doing these activities, but still do most.<br>4: Severe: I am unable to do most or all of these activities.	☐

Copyright © 2008 Movement Disorder Society. All rights reserved.
This chart may not be copied, distributed or otherwise used in whole or in part without prior written consent of the Movement Disorder Society.

	SCORE
**2.9 TURNING IN BED**<br><br>Over the past week, do you usually have trouble turning over in bed?<br><br>0: Normal: Not at all (no problems).<br>1: Slight: I have a bit of trouble turning, but I do not need any help.<br>2: Mild I have a lot of trouble turning and need occasional help from someone else.<br>3: Moderate: To turn over I often need help from someone else.<br>4: Severe: I am unable to turn over without help from someone else.	☐
**2.10 TREMOR**<br><br>Over the past week, have you usually had shaking or tremor?<br><br>0: Normal: Not at all. I have no shaking or tremor.<br>1: Slight: Shaking or tremor occurs but does not cause problems with any activities.<br>2: Mild: Shaking or tremor causes problems with only a few activities.<br>3: Moderate: Shaking or tremor causes problems with many of my daily activities.<br>4: Severe: Shaking or tremor causes problems with most or all activities.	☐
**2.11 GETTING OUT OF BED, A CAR, OR A DEEP CHAIR**<br><br>Over the past week, have you usually had trouble getting out of bed, a car seat, or a deep chair?<br><br>0: Normal: Not at all (no problems).<br>1: Slight: I am slow or awkward, but I usually can do it on my first try.<br>2: Mild: I need more than one try to get up or need occasional help.<br>3: Moderate: I sometimes need help to get up, but most times I can still do it on my own.<br>4: Severe: I need help most or all of the time.	☐

Copyright © 2008 Movement Disorder Society. All rights reserved.

This chart may not be copied, distributed or otherwise used in whole or in part without prior written consent of the Movement Disorder Society.

	SCORE
**2.12 WALKING AND BALANCE**<br><br>Over the past week, have you usually had problems with balance and walking?<br><br>0: Normal: Not at all (no problems).<br><br>1: Slight: I am slightly slow or may drag a leg. I never use a walking aid.<br><br>2: Mild: I occasionally use a walking aid, but I do not need any help from another person.<br><br>3: Moderate: I usually use a walking aid (cane, walker) to walk safely without falling. However, I do not usually need the support of another person.<br><br>4: Severe: I usually use the support of another persons to walk safely without falling.	☐
**2.13 FREEZING**<br><br>Over the past week, on your usual day when walking, do you suddenly stop or freeze as if your feet are stuck to the floor.<br><br>0: Normal: Not at all (no problems).<br><br>1: Slight: I briefly freeze but I can easily start walking again. I do not need help from someone else or a walking aid (cane or walker) because of freezing.<br><br>2: Mild: I freeze and have trouble starting to walk again, but I do not need someone's help or a walking aid (cane or walker) because of freezing.<br><br>3: Moderate: When I freeze I have a lot of trouble starting to walk again and, because of freezing, I sometimes need to use a walking aid or need someone else's help.<br><br>4: Severe: Because of freezing, most or all of the time, I need to use a walking aid or someone's help.	☐

This completes the questionnaire. We may have asked about problems you do not even have, and may have mentioned problems that you may never develop at all. Not all patients develop all these problems, but because they can occur, it is important to ask all the questions to every patient. Thank you for your time and attention in completing this questionnaire.

Copyright © 2008 Movement Disorder Society. All rights reserved.

This chart may not be copied, distributed or otherwise used in whole or in part without prior written consent of the Movement Disorder Society.

Part III: Motor Examination

Overview: This portion of the scale assesses the motor signs of PD. In administering Part III of the MDS-UPDRS the examiner should comply with the following guidelines:

At the top of the form, mark whether the patient is on medication for treating the symptoms of Parkinson's disease and, if on levodopa, the time since the last dose.

Also, if the patient is receiving medication for treating the symptoms of Parkinson's Disease, mark the patient's clinical state using the following definitions:
ON is the typical functional state when patients are receiving medication and have a good response.
OFF is the typical functional state when patients have a poor response in spite of taking medications.

The investigator should "rate what you see". Admittedly, concurrent medical problems such as stroke, paralysis, arthritis, contracture, and orthopedic problems such as hip or knee replacement and scoliosis may interfere with individual items in the motor examination. In situations where it is absolutely impossible to test (e.g., amputations, plegia, limb in a cast), use the notation "**UR**" for Unable to Rate. Otherwise, rate the performance of each task as the patient performs in the context of co-morbidities.

All items must have an integer rating (no half points, no missing ratings).

Specific instructions are provided for the testing of each item. These should be followed in all instances. The investigator demonstrates while describing tasks the patient is to perform and rates function immediately thereafter. For Global Spontaneous Movement and Rest Tremor items (3.14 and 3.17), these items have been placed purposefully at the end of the scale because clinical information pertinent to the score will be obtained throughout the entire examination.

At the end of the rating, indicate if dyskinesia (chorea or dystonia) was present at the time of the examination, and if so, whether these movements interfered with the motor examination.

3a Is the patient on medication for treating the symptoms of Parkinson's Disease? ☐ No ☐ Yes

3b If the patient is receiving medication for treating the symptoms of Parkinson's Disease, mark the patient's clinical state using the following definitions:

☐ ON: On is the typical functional state when patients are receiving medication and have a good response.

☐ OFF: Off is the typical functional state when patients have a poor response in spite of taking medications.

3c Is the patient on Levodopa ? ☐ No ☐ Yes

3.C1 If yes, minutes since last levodopa dose: __________

Copyright © 2008 Movement Disorder Society. All rights reserved.

This chart may not be copied, distributed or otherwise used in whole or in part without prior written consent of the Movement Disorder Society.

	SCORE
**3.1 SPEECH**<br><br><u>Instructions to examiner</u>: Listen to the patient's free-flowing speech and engage in conversation if necessary. Suggested topics: ask about the patient's work, hobbies, exercise, or how he got to the doctor's office. Evaluate volume, modulation (prosody) and clarity, including slurring, palilalia (repetition of syllables) and tachyphemia (rapid speech, running syllables together).<br><br>0: Normal: No speech problems.<br>1: Slight: Loss of modulation, diction or volume, but still all words easy to understand.<br>2: Mild: Loss of modulation, diction, or volume, with a few words unclear, but the overall sentences easy to follow.<br>3: Moderate: Speech is difficult to understand to the point that some, but not most, sentences are poorly understood.<br>4: Severe: Most speech is difficult to understand or unintelligible.	☐
**3.2 FACIAL EXPRESSION**<br><br><u>Instructions to examiner</u>: Observe the patient sitting at rest for 10 seconds, without talking and also while talking. Observe eye-blink frequency, masked facies or loss of facial expression, spontaneous smiling and parting of lips.<br><br>0: Normal: Normal facial expression.<br>1: Slight: Minimal masked facies manifested only by decreased frequency of blinking.<br>2: Mild: In addition to decreased eye-blink frequency, Masked facies present in the lower face as well, namely fewer movements around the mouth, such as less spontaneous smiling, but lips not parted.<br>3: Moderate: Masked facies with lips parted some of the time when the mouth is at rest.<br>4: Severe: Masked facies with lips parted most of the time when the mouth is at rest.	☐

July 1, 2008
Copyright © 2008 Movement Disorder Society. All rights reserved.
This chart may not be copied, distributed or otherwise used in whole or in part without prior written consent of the Movement Disorder Society.
Page 18

	SCORE
**3.3 RIGIDITY**<br><br><u>Instructions to examiner</u>: Rigidity is judged on slow passive movement of major joints with the patient in a relaxed position and the examiner manipulating the limbs and neck. First, test without an activation maneuver. Test and rate neck and each limb separately. For arms, test the wrist and elbow joints simultaneously. For legs, test the hip and knee joints simultaneously. If no rigidity is detected, use an activation maneuver such as tapping fingers, fist opening/closing, or heel tapping in a limb not being tested. Explain to the patient to go as limp as possible as you test for rigidity.<br><br>0: Normal: No rigidity.<br>1: Slight: Rigidity only detected with activation maneuver.<br>2: Mild: Rigidity detected without the activation maneuver, but full range of motion is easily achieved.<br>3: Moderate: Rigidity detected without the activation maneuver; full range of motion is achieved with effort.<br>4: Severe: Rigidity detected without the activation maneuver and full range of motion not achieved.	☐ Neck<br>☐ RUE<br>☐ LUE<br>☐ RLE<br>☐ LLE
**3.4 FINGER TAPPING**<br><br><u>Instructions to examiner</u>: Each hand is tested separately. Demonstrate the task, but do not continue to perform the task while the patient is being tested. Instruct the patient to tap the index finger on the thumb 10 times as quickly AND as big as possible. Rate each side separately, evaluating speed, amplitude, hesitations, halts and decrementing amplitude.<br><br>0: Normal: No problems.<br>1: Slight: Any of the following: a) the regular rhythm is broken with one or two interruptions or hesitations of the tapping movement; b) slight slowing; c) the amplitude decrements near the end of the 10 taps.<br>2: Mild: Any of the following: a) 3 to 5 interruptions during tapping; b) mild slowing; c) the amplitude decrements midway in the 10-tap sequence.<br>3: Moderate: Any of the following: a) more than 5 interruptions during tapping or at least one longer arrest (freeze) in ongoing movement; b) moderate slowing; c) the amplitude decrements starting after the 1st tap.<br>4: Severe: Cannot or can only barely perform the task because of slowing, interruptions or decrements.	☐ R<br>☐ L

Copyright © 2008 Movement Disorder Society. All rights reserved.

This chart may not be copied, distributed or otherwise used in whole or in part without prior written consent of the Movement Disorder Society.

SCORE

3.5 HAND MOVEMENTS

Instructions to examiner: Test each hand separately. Demonstrate the task, but do not continue to perform the task while the patient is being tested. Instruct the patient to make a tight fist with the arm bent at the elbow so that the palm faces the examiner. Have the patient open the hand 10 times as fully AND as quickly as possible. If the patient fails to make a tight fist or to open the hand fully, remind him/her to do so. Rate each side separately, evaluating speed, amplitude, hesitations, halts and decrementing amplitude.

0: Normal: No problem.

1: Slight: Any of the following: a) the regular rhythm is broken with one or two interruptions or hesitations of the movement; b) slight slowing; c) the amplitude decrements near the end of the task.

2: Mild: Any of the following: a) 3 to 5 interruptions during the movements; b) mild slowing; c) the amplitude decrements midway in the task.

3: Moderate: Any of the following: a) more than 5 interruptions during the movement or at least one longer arrest (freeze) in ongoing movement; b) moderate slowing; c) the amplitude decrements starting after the 1st open-and-close sequence.

4: Severe: Cannot or can only barely perform the task because of slowing, interruptions or decrements.

☐ R

☐ L

3.6 PRONATION-SUPINATION MOVEMENTS OF HANDS

Instructions to examiner: Test each hand separately. Demonstrate the task, but do not continue to perform the task while the patient is being tested. Instruct the patient to extend the arm out in front of his/her body with the palms down; then to turn the palm up and down alternately 10 times as fast and as fully as possible. Rate each side separately, evaluating speed, amplitude, hesitations, halts and decrementing amplitude.

0: Normal: No problems.

1: Slight: Any of the following: a) the regular rhythm is broken with one or two interruptions or hesitations of the movement; b) slight slowing; c) the amplitude decrements near the end of the sequence.

2: Mild: Any of the following: a) 3 to 5 interruptions during the movements; b) mild slowing; c) the amplitude decrements midway in the sequence.

3: Moderate: Any of the following: a) more than 5 interruptions during the movement or at least one longer arrest (freeze) in ongoing movement; b) moderate slowing c) the amplitude decrements starting after the 1st supination-pronation sequence.

4: Severe: Cannot or can only barely perform the task because of slowing, interruptions or decrements.

☐ R

☐ L

Copyright © 2008 Movement Disorder Society. All rights reserved.

This chart may not be copied, distributed or otherwise used in whole or in part without prior written consent of the Movement Disorder Society.

	SCORE
**3.7 TOE TAPPING**<br><br><u>Instructions to examiner</u>: Have the patient sit in a straight-backed chair with arms, both feet on the floor. Test each foot separately. Demonstrate the task, but do not continue to perform the task while the patient is being tested. Instruct the patient to place the heel on the ground in a comfortable position and then tap the toes 10 times as big and as fast as possible. Rate each side separately, evaluating speed, amplitude, hesitations, halts and decrementing amplitude.<br><br>0: Normal: No problem.<br><br>1: Slight: Any of the following: a) the regular rhythm is broken with one or two interruptions or hesitations of the tapping movement; b) slight slowing; c) amplitude decrements near the end of the ten taps.<br><br>2: Mild: Any of the following: a) 3 to 5 interruptions during the tapping movements; b) mild slowing; c) amplitude decrements midway in the task.<br><br>3: Moderate: Any of the following: a) more than 5 interruptions during the tapping movements or at least one longer arrest (freeze) in ongoing movement; b) moderate slowing; c) amplitude decrements after the first tap.<br><br>4: Severe: Cannot or can only barely perform the task because of slowing, interruptions or decrements.	☐ R<br><br>☐ L
**3.8 LEG AGILITY**<br><br><u>Instructions to examiner</u>: Have the patient sit in a straight-backed chair with arms. The patient should have both feet comfortably on the floor. Test each leg separately. Demonstrate the task, but do not continue to perform the task while the patient is being tested. Instruct the patient to place the foot on the ground in a comfortable position and then raise and stomp the foot on the ground 10 times as high and as fast as possible. Rate each side separately, evaluating speed, amplitude, hesitations, halts and decrementing amplitude.<br><br>0: Normal: No problems.<br><br>1: Slight: Any of the following: a) the regular rhythm is broken with one or two interruptions or hesitations of the movement; b) slight slowing; c) amplitude decrements near the end of the task.<br><br>2: Mild: Any of the following: a) 3 to 5 interruptions during the movements; b) mild slowness; c) amplitude decrements midway in the task.<br><br>3: Moderate: Any of the following: a) more than 5 interruptions during the movement or at least one longer arrest (freeze) in ongoing movement; b) moderate slowing in speed; c) amplitude decrements after the first tap.<br><br>4: Severe: Cannot or can only barely perform the task because of slowing, interruptions or decrements.	☐ R<br><br>☐ L

Copyright © 2008 Movement Disorder Society. All rights reserved.

This chart may not be copied, distributed or otherwise used in whole or in part without prior written consent of the Movement Disorder Society.

SCORE

3.9 ARISING FROM CHAIR

Instructions to examiner: Have the patient sit in a straight-backed chair with arms, with both feet on the floor and sitting back in the chair (if the patient is not too short). Ask the patient to cross his/her arms across the chest and then to stand up. If the patient is not successful, repeat this attempt a maximum up to two more times. If still unsuccessful, allow the patient to move forward in the chair to arise with arms folded across the chest. Allow only one attempt in this situation. If unsuccessful, allow the patient to push off using his/her hands on the arms of the chair. Allow a maximum of three trials of pushing off. If still not successful, assist the patient to arise. After the patient stands up, observe the posture for item 3.13

0: Normal: No problems. Able to arise quickly without hesitation.

1: Slight: Arising is slower than normal; or may need more than one attempt; or may need to move forward in the chair to arise. No need to use the arms of the chair.

2: Mild: Pushes self up from arms of chair without difficulty.

3: Moderate: Needs to push off, but tends to fall back; or may have to try more than one time using arms of chair, but can get up without help.

4: Severe: Unable to arise without help.

☐

3.10 GAIT

Instructions to examiner: Testing gait is best performed by having the patient walking away from and towards the examiner so that both right and left sides of the body can be easily observed simultaneously. The patient should walk at least 10 meters (30 feet), then turn around and return to the examiner. This item measures multiple behaviors: stride amplitude, stride speed, height of foot lift, heel strike during walking, turning, and arm swing, but not freezing. Assess also for “freezing of gait” (next item 3.11) while patient is walking. Observe posture for item 3.13

0: Normal: No problems.

1: Slight: Independent walking with minor gait impairment.

2: Mild: Independent walking but with substantial gait impairment.

3: Moderate: Requires an assistance device for safe walking (walking stick, walker) but not a person.

4: Severe: Cannot walk at all or only with another person’s assistance.

☐

Copyright © 2008 Movement Disorder Society. All rights reserved.

This chart may not be copied, distributed or otherwise used in whole or in part without prior written consent of the Movement Disorder Society.

	SCORE
**3.11 FREEZING OF GAIT**<br><br>Instructions to examiner: While assessing gait, also assess for the presence of any gait freezing episodes. Observe for start hesitation and stuttering movements especially when turning and reaching the end of the task. To the extent that safety permits, patients may NOT use sensory tricks during the assessment.<br><br>0: Normal: No freezing.<br>1: Slight: Freezes on starting, turning or walking through doorway with a single halt during any of these events, but then continues smoothly without freezing during straight walking.<br>2: Mild: Freezes on starting, turning or walking through doorway with more than one halt during any of these activities, but continues smoothly without freezing during straight walking.<br>3: Moderate: Freezes once during straight walking.<br>4: Severe: Freezes multiple times during straight walking.	☐
**3.12 POSTURAL STABILITY**<br><br>Instructions to examiner: The test examines the response to sudden body displacement produced by a quick, forceful pull on the shoulders while the patient is standing erect with eyes open and feet comfortably apart and parallel to each other. Test retropulsion. Stand behind the patient and instruct the patient on what is about to happen. Explain that s/he is allowed to take a step backwards to avoid falling. There should be a solid wall behind the examiner, at least 1-2 meters away to allow for the observation of the number of retropulsive steps. The first pull is an instructional demonstration and is purposely milder and not rated. The second time the shoulders are pulled briskly and forcefully towards the examiner with enough force to displace the center of gravity so that patient MUST take a step backwards. The examiner needs to be ready to catch the patient, but must stand sufficiently back so as to allow enough room for the patient to take several steps to recover independently. Do not allow the patient to flex the body abnormally forward in anticipation of the pull. Observe for the number of steps backwards or falling. Up to and including two steps for recovery is considered normal, so abnormal ratings begin with three steps. If the patient fails to understand the test, the examiner can repeat the test so that the rating is based on an assessment that the examiner feels reflects the patient's limitations rather than misunderstanding or lack of preparedness. Observe standing posture for item 3.13<br><br>0: Normal: No problems: Recovers with one or two steps.<br>1: Slight: 3-5 steps, but subject recovers unaided.<br>2: Mild: More than 5 steps, but subject recovers unaided.<br>3: Moderate: Stands safely, but with absence of postural response; falls if not caught by examiner.<br>4: Severe: Very unstable, tends to lose balance spontaneously or with just a gentle pull on the shoulders.	☐

July 1, 2008 *Copyright © 2008 Movement Disorder Society. All rights reserved.* Page 23
This chart may not be copied, distributed or otherwise used in whole or in part without prior written consent of the Movement Disorder Society.

	SCORE
**3.13 POSTURE**<br><br>Instructions to examiner: Posture is assessed with the patient standing erect after arising from a chair, during walking , and while being tested for postural reflexes. If you notice poor posture, tell the patient to stand up straight and see if the posture improves (see option 2 below). Rate the worst posture seen in these three observation points. Observe for flexion and side-to-side leaning.<br><br>0: Normal: No problems.<br>1: Slight: Not quite erect, but posture could be normal for older person.<br>2: Mild: Definite flexion, scoliosis or leaning to one side, but patient can correct posture to normal posture when asked to do so.<br>3: Moderate: Stooped posture, scoliosis or leaning to one side that cannot be corrected volitionally to a normal posture by the patient.<br>4: Severe: Flexion, scoliosis or leaning with extreme abnormality of posture.	☐
**3.14 GLOBAL SPONTANEITY OF MOVEMENT (BODY BRADYKINESIA)**<br><br>Instructions to examiner: This global rating combines all observations on slowness, hesitancy, and small amplitude and poverty of movement in general, including a reduction of gesturing and of crossing the legs. This assessment is based on the examiner's global impression after observing for spontaneous gestures while sitting, and the nature of arising and walking.<br><br>0: Normal: No problems.<br>1: Slight: Slight global slowness and poverty of spontaneous movements.<br>2: Mild: Mild global slowness and poverty of spontaneous movements.<br>3: Moderate: Moderate global slowness and poverty of spontaneous movements.<br>4: Severe: Severe global slowness and poverty of spontaneous movements.	☐
**3.15 POSTURAL TREMOR OF THE HANDS**<br><br>Instructions to examiner: All tremor, including re-emergent rest tremor, that is present in this posture is to be included in this rating. Rate each hand separately. Rate the highest amplitude seen. Instruct the patient to stretch the arms out in front of the body with palms down. The wrist should be straight and the fingers comfortably separated so that they do not touch each other. Observe this posture for 10 seconds.<br><br>0: Normal: No tremor.<br>1: Slight: Tremor is present but less than 1 cm in amplitude.<br>2: Mild: Tremor is at least 1 but less than 3 cm in amplitude.<br>3: Moderate: Tremor is at least 3 but less than 10 cm in amplitude.<br>4: Severe: Tremor is at least 10 cm in amplitude.	☐<br>R<br><br>☐<br>L

July 1, 2008 Page 24

Copyright © 2008 Movement Disorder Society. All rights reserved.
This chart may not be copied, distributed or otherwise used in whole or in part without prior written consent of the Movement Disorder Society.

	SCORE
**3.16 KINETIC TREMOR OF THE HANDS**<br><br>Instructions to examiner: This is tested by the finger-to-nose maneuver. With the arm starting from the outstretched position, have the patient perform at least three finger-to-nose maneuvers with each hand reaching as far as possible to touch the examiner's finger. The finger-to-nose maneuver should be performed slowly enough not to hide any tremor that could occur with very fast arm movements. Repeat with the other hand, rating each hand separately. The tremor can be present throughout the movement or as the tremor reaches either target (nose or finger). Rate the highest amplitude seen.<br><br>0: Normal: No tremor.<br>1: Slight: Tremor is present but less than 1 cm in amplitude.<br>2: Mild: Tremor is at least 1 but less than 3 cm in amplitude.<br>3: Moderate: Tremor is at least 3 but less than 10 cm in amplitude.<br>4: Severe: Tremor is at least 10 cm in amplitude.	☐ R<br>☐ L
**3.17 REST TREMOR AMPLITUDE**<br><br>Instructions to examiner: This and the next item have been placed purposefully at the end of the examination to allow the rater to gather observations on rest tremor that may appear at any time during the exam, including when quietly sitting, during walking and during activities when some body parts are moving but others are at rest. Score the maximum amplitude that is seen at any time as the final score. Rate only the amplitude and not the persistence or the intermittency of the tremor.<br>As part of this rating, the patient should sit quietly in a chair with the hands placed on the arms of the chair (not in the lap) and the feet comfortably supported on the floor for 10 seconds with no other directives. Rest tremor is assessed separately for all four limbs and also for the lip/jaw. Rate only the maximum amplitude that is seen at any time as the final rating.<br><br>Extremity ratings<br>0: Normal: No tremor.<br>1: Slight.: < 1 cm in maximal amplitude.<br>2: Mild: > 1 cm but < 3 cm in maximal amplitude.<br>3: Moderate: 3 - 10 cm in maximal amplitude.<br>4: Severe: > 10 cm in maximal amplitude.<br><br>Lip/Jaw ratings<br>0: Normal: No tremor.<br>1: Slight: < 1 cm in maximal amplitude.<br>2: Mild: > 1 cm but < 2 cm in maximal amplitude.<br>3: Moderate: > 2 cm but < 3 cm in maximal amplitude.<br>4: Severe: > 3 cm in maximal amplitude.	☐ RUE<br>☐ LUE<br>☐ RLE<br>☐ LLE<br>☐ Lip/Jaw

July 1, 2008 *Copyright © 2008 Movement Disorder Society. All rights reserved.* Page 25
This chart may not be copied, distributed or otherwise used in whole or in part without prior written consent of the Movement Disorder Society.

3.18 CONSTANCY OF REST TREMOR	SCORE
Instructions to examiner: This item receives one rating for all rest tremor and focuses on the constancy of rest tremor during the examination period when different body parts are variously at rest. It is rated purposefully at the end of the examination so that several minutes of information can be coalesced into the rating.<br><br>0: Normal: No tremor.<br>1: Slight: Tremor at rest is present < 25% of the entire examination period.<br>2: Mild: Tremor at rest is present 26-50% of the entire examination period.<br>3: Moderate: Tremor at rest is present 51-75% of the entire examination period.<br>4: Severe: Tremor at rest is present > 75% of the entire examination period.	☐

DYSKINESIA IMPACT ON PART III RATINGS

A. Were dyskinesias (chorea or dystonia) present during examination? ☐ No ☐ Yes

B. If yes, did these movements interfere with your ratings? ☐ No ☐ Yes

HOEHN AND YAHR STAGE	
0: Asymptomatic.<br>1: Unilateral involvement only.<br>2: Bilateral involvement without impairment of balance.<br>3: Mile to moderate involvement; some postural instability but physically independent; needs assistance to recover from pull test.<br>4: Severe disability; still able to walk or stand unassisted.<br>5: Wheelchair bound or bedridden unless aided.	☐

Copyright © 2008 Movement Disorder Society. All rights reserved.

This chart may not be copied, distributed or otherwise used in whole or in part without prior written consent of the Movement Disorder Society.

Part IV: Motor Complications

Overview and Instructions: In this section, the rater uses historical and objective information to assess two motor complications, dyskinesias and motor fluctuations that include OFF-state dystonia. Use all information from patient, caregiver, and the examination to answer the six questions that summarize function over the past week including today. As in the other sections, rate using only integers (no half points allowed) and leave no missing ratings. If the item cannot be rated, place UR for Unable to Rate. You will need to choose some answers based on percentages, and therefore you will need to establish how many hours generally are awake hours and use this figure as the denominator for "OFF" time and Dyskinesias. For "OFF dystonia", the total "Off" time will be the denominator. Operational definitions for examiner's use.

Dyskinesias: Involuntary random movements
Words that patients often recognize for dyskinesias include "irregular jerking", "wiggling", "twitching". It is essential to stress to the patient the difference between dyskinesias and tremor, a common error when patients are assessing dyskinesias.

Dystonia: contorted posture, often with a twisting component:
Words that patients often recognize for dystonia include "spasms", "cramps", "posture".

Motor fluctuation: Variable response to medication:
Words that patients often recognize for motor fluctuation include "wearing out", "wearing off", "roller-coaster effect", "on-off", "uneven medication effects".

OFF: Typical functional state when patients have a poor response in spite of taking mediation or the typical functional response when patients are on NO treatment for parkinsonism. Words that patients often recognize include "low time", "bad time", "shaking time", "slow time", "time when my medications don't work."

ON: Typical functional state when patients are receiving medication and have a good response:
Words that patients often recognize include "good time", "walking time", "time when my medications work."

A . DYSKINESIAS [exclusive of OFF-state dystonia]

4.1 TIME SPENT WITH DYSKINESIAS — **SCORE** ☐

Instructions to examiner: Determine the hours in the usual waking day and then the hours of dyskinesias. Calculate the percentage. If the patient has dyskinesias in the office, you can point them out as a reference to ensure that patients and caregivers understand what they are rating. You may also use your own acting skills to enact the dyskinetic movements you have seen in the patient before or show them dyskinetic movements typical of other patients. Exclude from this question early morning and nighttime painful dystonia.

Instructions to patient [and caregiver]. Over the past week, how many hours do you usually sleep on a daily basis, including nighttime sleep and daytime napping? Alright, if you sleep ___ hrs, you are awake ____ hrs. Out of those awake hours, how many hours in total do you have wiggling, twitching or jerking movements? Do not count the times when you have tremor, which is a regular back and forth shaking or times when you have painful foot cramps or spasms in the early morning or at nighttime. I will ask about those later. Concentrate only on these types of wiggling, jerking and irregular movements. Add up all the time during the waking day when these usually occur. How many hours ____ (use this number for your calculation).

0: Normal:	No dyskinesias.	
1: Slight:	≤ 25% of waking day.	
2: Mild:	26 - 50% of waking day.	1. Total Hours Awake: _____
3: Moderate:	51 - 75% of waking day.	2. Total Hours with Dyskinesia: _____
4: Severe:	> 75% of waking day.	3. % Dyskinesia = ((2/1)*100): _____

Copyright © 2008 Movement Disorder Society. All rights reserved.
This chart may not be copied, distributed or otherwise used in whole or in part without prior written consent of the Movement Disorder Society.

SCORE

4.2 FUNCTIONAL IMPACT OF DYSKINESIAS

Instructions to examiner: Determine the degree to which dyskinesias impact on the patient's daily function in terms of activities and social interactions. Use the patient's and caregiver's response to your question and your own observations during the office visit to arrive at the best answer.

Instructions to patient [and caregiver]: Over the past week, did you usually have trouble doing things or being with people when these jerking movements occurred? Did they stop you from doing things or from being with people?

0: Normal:	No dyskinesias or no impact by dyskinesias on activities or social interactions.
1: Slight:	Dyskinesias impact on a few activities, but the patient usually performs all activities and participates in all social interactions during dyskinetic periods.
2: Mild:	Dyskinesias impact on many activities, but the patient usually performs all activities and participates in all social interactions during dyskinetic periods.
3: Moderate:	Dyskinesias impact on activities to the point that the patient usually does not perform some activities or does not usually participate in some social activities during dyskinetic episodes.
4: Severe:	Dyskinesias impact on function to the point that the patient usually does not perform most activities or participate in most social interactions during dyskinetic episodes.

☐

B . MOTOR FLUCTUATIONS

4.3 TIME SPENT IN THE OFF STATE

Instructions to examiner: Use the number of waking hours derived from 4.1 and determine the hours spent in the "OFF" state. Calculate the percentage. If the patient has an OFF period in the office, you can point to this state as a reference. You may also use your knowledge of the patient to describe a typical OFF period. Additionally you may use your own acting skills to enact an OFF period you have seen in the patient before or show them OFF function typical of other patients. Mark down the typical number of OFF hours, because you will need this number for completing 4.6

Instructions to patient [and caregiver]: Some patients with Parkinson's disease have a good effect from their medications throughout their awake hours and we call that "ON" time. Other patients take their medications but still have some hours of low time, bad time, slow time or shaking time. Doctors call these low periods "OFF" time. Over the past week, you told me before that you are generally awake ____ hrs each day. Out of these awake hours, how many hours in total do you usually have this type of low level or OFF function ____ (Use this number for your calculations).

0: Normal:	No OFF time.
1: Slight:	≤ 25% of waking day.
2: Mild:	26 - 50% of waking day.
3: Moderate:	51 - 75% of waking day.
4: Severe:	> 75% of waking day.

1. Total Hours Awake: _____
2. Total Hours OFF: _____
3. % OFF = ((2/1)*100): _____

☐

July 1, 2008

Copyright © 2008 Movement Disorder Society. All rights reserved.
This chart may not be copied, distributed or otherwise used in whole or in part without prior written consent of the Movement Disorder Society.

	SCORE
**4.4 FUNCTIONAL IMPACT OF FLUCTUATIONS**<br><br>Instructions to examiner: Determine the degree to which motor fluctuations impact on the patient's daily function in terms of activities and social interactions. This question concentrates on the difference between the ON state and the OFF state. If the patient has no OFF time, the rating must be 0, but if patients have very mild fluctuations, it is still possible to be rated 0 on this item if no impact on activities occurs. Use the patient's and caregiver's response to your question and your own observations during the office visit to arrive at the best answer.<br><br>*Instructions to patient [and caregiver]: Think about when those low or "OFF" periods have occurred over the past week. Do you usually have more problems doing things or being with people than compared to the rest of the day when you feel your medications working? Are there some things you usually do during a good period that you have trouble with or stop doing during a low period?*<br><br>0: Normal: No fluctuations or No impact by fluctuations on performance of activities or social interactions.<br><br>1: Slight: Fluctuations impact on a few activities, but during OFF, the patient usually performs all activities and participates in all social interactions that typically occur during the ON state.<br><br>2: Mild: Fluctuations impact many activities, but during OFF, the patient still usually performs all activities and participates in all social interactions that typically occur during the ON state.<br><br>3: Moderate: Fluctuations impact on the performance of activities during OFF to the point that the patient usually does not perform some activities or participate in some social interactions that are performed during ON periods.<br><br>4: Severe: Fluctuations impact on function to the point that, during OFF, the patient usually does not perform most activities or participate in most social interactions that are performed during ON periods.	☐
**4.5 COMPLEXITY OF MOTOR FLUCTUATIONS**<br><br>Instructions to examiner: Determine the usual predictability of OFF function whether due to dose, time of day, food intake or other factors. Use the information provided by the patients and caregiver and supplement with your own observations. You will ask if the patient can count on them always coming at a special time, mostly coming at a special time (in which case you will probe further to separate slight from mild), only sometimes coming at a special time or are they totally unpredictable? Narrowing down the percentage will allow you to find the correct answer.<br><br>*Instructions to patient [and caregiver]: For some patients, the low or "OFF" periods happen at certain times during day or when they do activities like eating or exercising. Over the past week, do you usually know when your low periods will occur? In other words, do your low periods always come at a certain time? Do they mostly come at a certain time? Do they only sometimes come at a certain time? Are your low periods totally unpredictable?"*<br><br>0: Normal: No motor fluctuations.<br><br>1: Slight: OFF times are predictable all or almost all of the time (> 75%).<br><br>2: Mild: OFF times are predictable most of the time (51-75%).<br><br>3: Moderate: OFF times are predictable some of the time (26-50%).<br><br>4: Severe: OFF episodes are rarely predictable. ($\leq$ 25%).	☐

July 1, 2008 Copyright © 2008 Movement Disorder Society. All rights reserved. Page 29
This chart may not be copied, distributed or otherwise used in whole or in part without prior written consent of the Movement Disorder Society.

C. "OFF" DYSTONIA

4.6 PAINFUL OFF-STATE DYSTONIA

Instructions to examiner: For patients who have motor fluctuations, determine what proportion of the OFF episodes usually includes painful dystonia? You have already determined the number of hours of "OFF" time (4.3). Of these hours, determine how many are associated with dystonia and calculate the percentage. If there is no OFF time, mark 0.

Instructions to patient [and caregiver]: In one of the questions I asked earlier, you said you generally have ___ hours of low or "OFF" time when your Parkinson's disease is under poor control. During these low or "OFF" periods, do you usually have painful cramps or spasms? Out of the total ____ hrs of this low time, if you add up all the time in a day when these painful cramps come, how many hours would this make?

0: Normal: No dystonia OR NO OFF TIME.

1: Slight: < 25% of time in OFF state.

2: Mild: 26-50% of time in OFF state.

3: Moderate: 51-75% of time in OFF state.

4: Severe: > 75% of time in OFF state.

☐

1. Total Hours Off: _____
2. Total Off Hours w/Dystonia: _____
3. % Off Dystonia = ((2/1)*100): _____

Summary statement to patient: READ TO PATIENT

This completes my rating of your Parkinson's disease. I know the questions and tasks have taken several minutes, but I wanted to be complete and cover all possibilities. In doing so, I may have asked about problems you do not even have, and I may have mentioned problems that you may never develop at all. Not all patients develop all these problems, but because they can occur, it is important to ask all the questions to every patient. Thank you for your time and attention in completing this scale with me.

July 1, 2008 *Copyright © 2008 Movement Disorder Society. All rights reserved.*
This chart may not be copied, distributed or otherwise used in whole or in part without prior written consent of the Movement Disorder Society. Page 30

______________	___ ___ ___	__ __ - __ __ - __ __ __ __<br>(mm-dd-yyyy)	___ ___ ___
Patient Name or Subject ID	Site ID	Assessment Date	Investigator's Initials

MDS UPDRS Score Sheet

1.A	Source of information	☐ Patient<br>☐ Caregiver<br>☐ Patient + Caregiver	3.3b	Rigidity– RUE	
			3.3c	Rigidity– LUE	
**Part I**			3.3d	Rigidity– RLE	
1.1	Cognitive impairment		3.3e	Rigidity– LLE	
1.2	Hallucinations and psychosis		3.4a	Finger tapping– Right hand	
1.3	Depressed mood		3.4b	Finger tapping– Left hand	
1.4	Anxious mood		3.5a	Hand movements– Right hand	
1.5	Apathy		3.5b	Hand movements– Left hand	
1.6	Features of DDS		3.6a	Pronation- supination movements– Right hand	
1.6a	Who is filling out questionnaire	☐ Patient<br>☐ Caregiver<br>☐ Patient + Caregiver	3.6b	Pronation- supination movements– Left hand	
			3.7a	Toe tapping–Right foot	
1.7	Sleep problems		3.7b	Toe tapping– Left foot	
1.8	Daytime sleepiness		3.8a	Leg agility– Right leg	
1.9	Pain and other sensations		3.8b	Leg agility– Left leg	
1.10	Urinary problems		3.9	Arising from chair	
1.11	Constipation problems		3.10	Gait	
1.12	Light headedness on standing		3.11	Freezing of gait	
1.13	Fatigue		3.12	Postural stability	
**Part II**			3.13	Posture	
2.1	Speech		3.14	Global spontaneity of movement	
2.2	Saliva and drooling		3.15a	Postural tremor– Right hand	
2.3	Chewing and swallowing		3.15b	Postural tremor– Left hand	
2.4	Eating tasks		3.16a	Kinetic tremor– Right hand	
2.5	Dressing		3.16b	Kinetic tremor– Left hand	
2.6	Hygiene		3.17a	Rest tremor amplitude– RUE	
2.7	Handwriting		3.17b	Rest tremor amplitude– LUE	
2.8	Doing hobbies and other activities		3.17c	Rest tremor amplitude– RLE	
2.9	Turning in bed		3.17d	Rest tremor amplitude– LLE	
2.10	Tremor		3.17e	Rest tremor amplitude– Lip/jaw	
2.11	Getting out of bed		3.18	Constancy of rest	
2.12	Walking and balance			Were dyskinesias presen	☐ No ☐ Yes
2.13	Freezing			Did these movements interfere with ratings?	☐ No ☐ Yes
3a	Is the patient on medication?	☐ No ☐ Yes		Hoehn and Yahr Stage	
3b	Patient's clinical state	☐ Off ☐ On	**Part IV**		
3c	Is the patient on Levodopa?	☐ No ☐ Yes	4.1	Time spent with dyskinesias	
3.C1	If yes, minutes since last dose:		4.2	Functional impact of dyskinesias	
**Part III**			4.3	Time spent in the OFF state	
3.1	Speech		4.4	Functional impact of fluctuations	
3.2	Facial expression		4.5	Complexity of motor fluctuations	
3.3a	Rigidity– Neck		4.6	Painful OFF-state dystonia	

July 1, 2008

Copyright © 2008 Movement Disorder Society. All rights reserved.
This chart may not be copied, distributed or otherwise used in whole or in part without prior written consent of the Movement Disorder Society

APPENDIX F. PATIENT GLOBAL IMPRESSION OF CHANGE (PGI-C)

The subject will independently rate the following question of Patient Global Impression of Change (PGI-C) based on his/her overall impression at Visit 5 (Week 10), Visit 6 (Week 15), and Visit 7 (Week 20) or early discontinuation.

Patient Global Impression of Change:

Compared to your condition prior to your starting on this study, how much has your condition changed with your current treatment?

❑ 1	❑ 2	❑ 3	❑ 4	❑ 5	❑ 6	❑ 7
Very Much Worse	Much Worse	Minimally Worse	No Change	Minimally Improved	Much Improved	Very Much Improved

APPENDIX G. CLINICAL GLOBAL IMPRESSION OF CHANGE (CGI-C)

The Investigator rates each subject with the following question as part of Visit 5 (Week 10), Visit 6 (Week 15), and Visit 7 (Week 20) or early discontinuation:

Clinical Global Impression of Change:

In your opinion, how much has the subject's overall condition and Parkinson's disease symptoms changed since starting on the study?

❑ 1	❑ 2	❑ 3	❑ 4	❑ 5	❑ 6	❑ 7
Very Much Worse	Much Worse	Minimally Worse	Neutral	Minimally Improved	Much Improved	Very Much Improved

Guy W. ECDEU assessment manual for psychopharmacology publication; ADM, 76-338.

Washington DC, US. Department of health, education and welfare, 1976.

APPENDIX H. PATIENT GLOBAL IMPRESSION OF SEVERITY (PGI-S)

Patient Global Impression – Severity Scale

Severity of Illness

Considering the severity of your Parkinson's disease, how severe is your condition at this time?

Severity Score:

❑ 1	❑ 2	❑ 3	❑ 4	❑ 5	❑ 6	❑ 7
Normal, not at all ill	Borderline ill	Mildly ill	Moderately ill	Markedly ill	Severely ill	Extremely severely ill

APPENDIX I. CLINICAL GLOBAL IMPRESSION OF SEVERITY (CGI-S)

The Investigator will independently rate the following question of Clinical Global Impression of Severity (CGI-S) based on his/her overall impression of the study medication at Visit 1, Visit 4, and Visit 7 or early discontinuation.

Clinical Global Impression – Severity Scale

Severity of Illness

Considering your total clinical experience with this particular PD population, how ill is the patient at this time?

Severity Score:

❑ 1	❑ 2	❑ 3	❑ 4	❑ 5	❑ 6	❑ 7
Normal, not at all ill	Borderline ill	Mildly ill	Moderately ill	Markedly ill	Severely ill	Among the most extremely ill of subjects

Guy W. ECDEU assessment manual for psychopharmacology publication; ADM, 76-338.

Washington DC, US. Department of health, education and welfare, 1976.

APPENDIX J. 39-ITEM PARKINSON'S DISEASE QUESTIONNAIRE (PDQ-39)

PDQ-39 QUESTIONNAIRE

Please complete the following

Please tick one box for each question

Due to having Parkinson's disease, how often during the last month have you....		Never	Occasionally	Sometimes	Often	Always or cannot do at all
1	Had difficulty doing the leisure activities which you would like to do?	☐	☐	☐	☐	☐
2	Had difficulty looking after your home, e.g. DIY, housework, cooking?	☐	☐	☐	☐	☐
3	Had difficulty carrying bags of shopping?	☐	☐	☐	☐	☐
4	Had problems walking half a mile?	☐	☐	☐	☐	☐
5	Had problems walking 100 yards?	☐	☐	☐	☐	☐
6	Had problems getting around the house as easily as you would like?	☐	☐	☐	☐	☐
7	Had difficulty getting around in public?	☐	☐	☐	☐	☐
8	Needed someone else to accompany you when you went out?	☐	☐	☐	☐	☐
9	Felt frightened or worried about falling over in public?	☐	☐	☐	☐	☐
10	Been confined to the house more than you would like?	☐	☐	☐	☐	☐
11	Had difficulty washing yourself?	☐	☐	☐	☐	☐
12	Had difficulty dressing yourself?	☐	☐	☐	☐	☐
13	Had problems doing up your shoe laces?	☐	☐	☐	☐	☐

*Please check that you have ticked **one box for each question** before going on to the next page*

Due to having Parkinson's disease, how often <u>during the last month</u> have you....

Please tick <u>one</u> box for each question

		Never	Occasionally	Sometimes	Often	Always<br>or cannot do at all
14	Had problems writing clearly?	☐	☐	☐	☐	☐
15	Had difficulty cutting up your food?	☐	☐	☐	☐	☐
16	Had difficulty holding a drink without spilling it?	☐	☐	☐	☐	☐
17	Felt depressed?	☐	☐	☐	☐	☐
18	Felt isolated and lonely?	☐	☐	☐	☐	☐
19	Felt weepy or tearful?	☐	☐	☐	☐	☐
20	Felt angry or bitter?	☐	☐	☐	☐	☐
21	Felt anxious?	☐	☐	☐	☐	☐
22	Felt worried about your future?	☐	☐	☐	☐	☐
23	Felt you had to conceal your Parkinson's from people?	☐	☐	☐	☐	☐
24	Avoided situations which involve eating or drinking in public?	☐	☐	☐	☐	☐
25	Felt embarrassed in public due to having Parkinson's disease?	☐	☐	☐	☐	☐
26	Felt worried by other people's reaction to you?	☐	☐	☐	☐	☐
27	Had problems with your close personal relationships?	☐	☐	☐	☐	☐
28	Lacked support in the ways you need from your spouse or partner?	☐	☐	☐	☐	☐
	If you do not have a spouse or partner tick here		☐			
29	Lacked support in the ways you need from your family or close friends?	☐	☐	☐	☐	☐

*Please check that you have ticked **one box for each question** before going on to the next page*

Due to having Parkinson's disease, how often <u>during the last month</u> have you....	Never	Occasionally	Sometimes	Often	Always
30 Unexpectedly fallen asleep during the day?	☐	☐	☐	☐	☐
31 Had problems with your concentration, e.g. when reading or watching TV?	☐	☐	☐	☐	☐
32 Felt your memory was bad?	☐	☐	☐	☐	☐
33 Had distressing dreams or hallucinations?	☐	☐	☐	☐	☐
34 Had difficulty with your speech?	☐	☐	☐	☐	☐
35 Felt unable to communicate with people properly?	☐	☐	☐	☐	☐
36 Felt ignored by people?	☐	☐	☐	☐	☐
37 Had painful muscle cramps or spasms?	☐	☐	☐	☐	☐
38 Had aches and pains in your joints or body?	☐	☐	☐	☐	☐
39 Felt unpleasantly hot or cold?	☐	☐	☐	☐	☐

Please tick <u>one</u> box for each question

Please check that you have ticked ***one box for each question*** *before going on to the next page*

Thank you for completing the PDQ 39 questionnaire

APPENDIX K. GASTROPARESIS CARDINAL SYMPTOM INDEX (GCSI)

GASTROPARESIS CARDINAL SYMPTOM INDEX

This questionnaire asks you about the severity of symptoms you may have related to your gastrointestinal problem. There are no right or wrong answers. Please answer each question as accurately as possible.

For each symptom, please circle the number that best describes how severe the symptom has been during the past 2 weeks.

- If you have not experienced this symptom, circle 0.
- If the symptom has been very mild, circle 1.
- If the symptom has been mild, circle 2.
- If it has been moderate, circle 3.
- If it has been severe, circle 4.
- If it has been very severe, circle 5.

Please be sure to answer every question.

Please rate the severity of the following symptoms during the past 2 weeks.

		None	Very mild	Mild	Moderate	Severe	Very severe
1.	nausea (feeling sick to your stomach as if you were going to vomit or throw up)	0	1	2	3	4	5
2.	retching (heaving as if to vomit, but nothing comes up)	0	1	2	3	4	5
3.	vomiting	0	1	2	3	4	5
4.	stomach fullness	0	1	2	3	4	5
5.	not able to finish a normal-sized meal	0	1	2	3	4	5
6.	feeling excessively full after meals	0	1	2	3	4	5
7.	loss of appetite	0	1	2	3	4	5
8.	bloating (feeling like you need to loosen your clothes)	0	1	2	3	4	5
9.	stomach or belly visibly larger	0	1	2	3	4	5

©2002 Johnson & Johnson Pharmaceutical Services, LLC. Contact D. Dubois for permission to use.

APPENDIX L. NON-MOTOR SYMPTOM ASSESSMENT SCALE FOR PARKINSON'S DISEASE (NMSS)

Non-Motor Symptom assessment scale for Parkinson's Disease

Patient ID No: ________________________ **Initials:** ________________ **Age:** __________

Symptoms assessed over the last month. Each symptom scored with respect to:

Severity: 0 = None, 1 = Mild: symptoms present but causes little distress or disturbance to patient; 2 = Moderate: some distress or disturbance to patient; 3 = Severe: major source of distress or disturbance to patient.

Frequency: 1 = Rarely (<1/wk); 2 = Often (1/wk); 3 = Frequent (several times per week); 4 = Very Frequent (daily or all the time)

Domains will be weighed differentially. Yes/ No answers are not included in final frequency x severity calculation.
(Bracketed text in questions within the scale is included as an explanatory aid).

**Domain 1: Cardiovascular including falls**	**Severity**	**Frequency**	**Frequency x Severity**
1. Does the patient experience light-headedness, dizziness, weakness on standing from sitting or lying position?	☐	☐	☐
2. Does the patient fall because of fainting or blacking out?	☐	☐	☐
**SCORE:**			☐

**Domain 2: Sleep/fatigue**			
3. Does the patient doze off or fall asleep unintentionally during daytime activities? (For example, during conversation, during mealtimes, or while watching television or reding).	☐	☐	☐
4. Does fatigue (tiredness) or lack of energy (not slowness) limit the patient's daytime activities?	☐	☐	☐
5. Does the patient have difficulties falling or staying asleep?	☐	☐	☐
6. Does the patient experience an urge to move the legs or restlessness in legs that improves with movement when he/she is sitting or lying down inactive?	☐	☐	☐
**SCORE:**			☐

**Domain 3: Mood /Cognition**			
7. Has the patient lost interest in his/her surroundings?	☐	☐	☐
8. Has the patient lost interest in doing things or lack motivation to start new activities?	☐	☐	☐
9. Does the patient feel nervous, worried or frightened for no apparent reason?	☐	☐	☐
10. Does the patient seem sad or depressed or has he/she reported such feelings?	☐	☐	☐
11. Does the patient have flat moods without the normal "highs" and " lows"?	☐	☐	☐
12. Does the patient have difficulty in experiencing pleasure from their usual activities or report that they lack pleasure?	☐	☐	☐
**SCORE:**			☐

**Domain 4: Perceptual problems/hallucinations**			
13. Does the patient indicate that he/she sees things that are not there?	☐	☐	☐
14. Does the patient have beliefs that you know are not true? (For example, about being harmed, being robbed or being unfaithful)	☐	☐	☐
15. Does the patient experience double vision? (2 separate real objects and not blurred vision)	☐	☐	☐
**SCORE:**			☐

	Severity	Frequency	Frequency x Severity
**Domain 5: Attention/ Memory**			
16. Does the patient have problems sustaining concentration during activities? (For example, reading or having a conversation)	☐	☐	☐
17. Does the patient forget things that he/she has been told a short time ago or events that happened in the last few days?	☐	☐	☐
18. Does the patient forget to do things? (For example, take tablets or turn off domestic appliances?)	☐	☐	☐
**SCORE:**			☐
**Domain 6: Gastrointestinal tract**			
19. Does the patient dribble saliva during the day?	☐	☐	☐
20. Does the patient having difficulty swallowing?	☐	☐	☐
21. Does the patient suffer from constipation? (Bowel action less than three times weekly)	☐	☐	☐
**SCORE:**			☐
**Domain 7: Urinary**			
22. Does the patient have difficulty holding urine? (Urgency)	☐	☐	☐
23. Does the patient have to void within 2 hours of last voiding? (Frequency)	☐	☐	☐
24. Does the patient have to get up regularly at night to pass urine? (Nocturia)	☐	☐	☐
**SCORE:**			☐
**Domain 8: Sexual function**			
25. Does the patient have altered interest in sex? (Very much increased or decreased, please underline)	☐	☐	☐
26. Does the patient have problems having sex?	☐	☐	☐
**SCORE:**			☐
**Domain 9: Miscellaneous**			
27. Does the patient suffer from pain not explained by other known conditions? (Is it related to intake of drugs and is it relieved by antiparkinson drugs?)	☐	☐	☐
28. Does the patient report a change in ability to taste or smell?	☐	☐	☐
29. Does the patient report a recent change in weight (not related to dieting)?	☐	☐	☐
30. Does the patient experience excessive sweating? (not related to hot weather)	☐	☐	☐
**SCORE:**			☐

TOTAL SCORE: ☐

Developed by the International Parkinson's Disease Non- Motor Group.
Contacts: ray.chaudhuri@uhl.nhs.uk or alison.forbes@uhl.nhs.uk

APPENDIX M. PARKINSON'S DISEASE SLEEP SCALE-2 (PDSS-2)

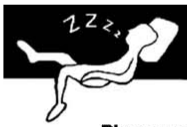

Parkinson's Disease Sleep Scale (PDSS-2)

Please rate the severity of the following based on your experiences during the past week (7 days). Please make a cross in the answer box

	Very often (This means 6 to 7 days a week)	Often (This means 4 to 5 days a week)	Sometimes (This means 2 to 3 days a week)	Occasionally (This means 1 day a week)	Never
1) Overall, did you sleep well during the last week?	☐ 0	☐ 1	☐ 2	☐ 3	☐ 4
2) Did you have difficulty falling asleep each night?	☐ 4	☐ 3	☐ 2	☐ 1	☐ 0
3) Did you have difficulty staying asleep?	☐ 4	☐ 3	☐ 2	☐ 1	☐ 0
4) Did you have restlessness of legs or arms at nights causing disruption of sleep?	☐ 4	☐ 3	☐ 2	☐ 1	☐ 0
5) Was your sleep disturbed due to an urge to move your legs or arms?	☐ 4	☐ 3	☐ 2	☐ 1	☐ 0
6) Did you suffer from distressing dreams at night?	☐ 4	☐ 3	☐ 2	☐ 1	☐ 0
7) Did you suffer from distressing hallucinations at night (seeing or hearing things that you are told do not exist)?	☐ 4	☐ 3	☐ 2	☐ 1	☐ 0
8) Did you get up at night to pass urine?	☐ 4	☐ 3	☐ 2	☐ 1	☐ 0
9) Did you feel uncomfortable at night because you were unable to turn around in bed or move due to immobility?	☐ 4	☐ 3	☐ 2	☐ 1	☐ 0
10) Did you feel pain in your arms or legs which woke you up from sleep at night?	☐ 4	☐ 3	☐ 2	☐ 1	☐ 0
11) Did you have muscle cramps in your arms or legs which woke you up whilst sleeping at night?	☐ 4	☐ 3	☐ 2	☐ 1	☐ 0
12) Did you wake early in the morning with painful posturing of arms and legs?	☐ 4	☐ 3	☐ 2	☐ 1	☐ 0
13) On waking, did you experience tremor?	☐ 4	☐ 3	☐ 2	☐ 1	☐ 0
14) Did you feel tired and sleepy after waking in the morning?	☐ 4	☐ 3	☐ 2	☐ 1	☐ 0
15) Did you wake up at night due to snoring or difficulties with breathing?	☐ 4	☐ 3	☐ 2	☐ 1	☐ 0

Trenkwalder C, Kohnen R, Högl B, Metta V, Sixel-Döring F, Frauscher B, Hülsmann J, Martinez-Martin P, Chaudhuri KR. Parkinson's disease sleep scale--validation of the revised version PDSS-2. Mov Disord. 2011;26(4):644-52.

APPENDIX N. PARKINSON ANXIETY SCALE (PAS)

The Parkinson Anxiety Scale (PAS)

(Please mark one circle for each item below)

In the past four weeks, to what extent did you experience the following symptoms?

A. Persistent Anxiety

A.1. Feeling anxious or nervous

- o Not at all, or never
- o Very mild, or rarely
- o Mild, or sometimes
- o Moderate, or often
- o Severe, or (nearly) always

A.2. Feeling tense or stressed

- o Not at all, or never
- o Very mild, or rarely
- o Mild, or sometimes
- o Moderate, or often
- o Severe, or (nearly) always

A.3. Being unable to relax

- o Not at all, or never
- o Very mild, or rarely
- o Mild, or sometimes
- o Moderate, or often
- o Severe, or (nearly) always

A.4. Excessive worrying about everyday matters

- o Not at all, or never
- o Very mild, or rarely
- o Mild, or sometimes
- o Moderate, or often
- o Severe, or (nearly) always

A.5. Fear of something bad, or even the worst, happening

- o Not at all, or never
- o Very mild, or rarely
- o Mild, or sometimes
- o Moderate, or often
- o Severe, or (nearly) always

B. Episodic Anxiety

B.1. Panic or intense fear

- Never
- Rarely
- Sometimes
- Often
- Nearly always

B.2. Shortness of breath

- Never
- Rarely
- Sometimes
- Often
- Nearly always

B.3. Heart palpitations or heart beating fast (not related to physical effort or activity)

- Never
- Rarely
- Sometimes
- Often
- Nearly always

B.4. Fear of losing control

- Never
- Rarely
- Sometimes
- Often
- Nearly always

C. Avoidance Behavior

C.1. Social situations (where one may be observed, or evaluated by others, such as speaking in public, or talking to unknown people)

- Never
- Rarely
- Sometimes
- Often
- Nearly always

C.2. Public settings (situations from which it may be difficult or embarrassing to escape, such as queues or lines, crowds, bridges, or public transportation)

- Never
- Rarely
- Sometimes
- Often
- Nearly always

C.3. Specific objects or situations (such as flying, heights, spiders or other animals, needles, or blood)

- Never
- Rarely
- Sometimes
- Often
- Nearly always

Copyright of this scale and it translations is held by the authors (Leentjens AF, Dujardin K, Pontone GM, Starkstein SE, Weintraub D, and Martinez-Martin P. The Parkinson Anxiety Scale (PAS): development and validation of a new anxiety scale. Mov Disord. 2014;29(8):1035-43). The scale and its translations are in the public domain and may be used without additional permission and free of charge on the condition that its source is referenced.

APPENDIX O. COLUMBIA-SUICIDE SEVERITY RATING SCALE (C-SSRS)

COLUMBIA-SUICIDE SEVERITY RATING SCALE (C-SSRS)

Baseline/Screening Version

Version 1/14/09

Posner, K.; Brent, D.; Lucas, C.; Gould, M.; Stanley, B.; Brown, G.; Fisher, P.; Zelazny, J.; Burke, A.; Oquendo, M.; Mann, J.

Disclaimer:

This scale is intended to be used by individuals who have received training in its administration. The questions contained in the Columbia-Suicide Severity Rating Scale are suggested probes. Ultimately, the determination of the presence of suicidal ideation or behavior depends on the judgment of the individual administering the scale.

Definitions of behavioral suicidal events in this scale are based on those used in **The Columbia Suicide History Form**, *developed by John Mann, MD and Maria Oquendo, MD, Conte Center for the Neuroscience of Mental Disorders (CCNMD), New York State Psychiatric Institute, 1051 Riverside Drive, New York, NY, 10032. (Oquendo M. A., Halberstam B. & Mann J. J., Risk factors for suicidal behavior: utility and limitations of research instruments. In M.B. First [Ed.]* Standardized Evaluation in Clinical Practice, *pp. 103 -130, 2003.)*

For reprints of the C-SSRS contact Kelly Posner, Ph.D., New York State Psychiatric Institute, 1051 Riverside Drive, New York, New York, 10032; inquiries and training requirements contact posnerk@nyspi.columbia.edu

© 2008 The Research Foundation for Mental Hygiene, Inc.

SUICIDAL IDEATION		
Ask questions 1 and 2. If both are negative, proceed to "Suicidal Behavior" section. If the answer to question 2 is "yes", ask questions 3, 4 and 5. If the answer to question 1 and/or 2 is "yes", complete "Intensity of Ideation" section below.	**Lifetime: Time He/She Felt Most Suicidal**	**Past __ Months**
**1. Wish to be Dead**<br>Subject endorses thoughts about a wish to be dead or not alive anymore, or wish to fall asleep and not wake up.<br>***Have you wished you were dead or wished you could go to sleep and not wake up?***<br><br>If yes, describe:	**Yes** ☐ **No** ☐	**Yes** ☐ **No** ☐
**2. Non-Specific Active Suicidal Thoughts**<br>General non-specific thoughts of wanting to end one's life/commit suicide (e.g., *"I've thought about killing myself"*) without thoughts of ways to kill oneself/associated methods, intent, or plan during the assessment period.<br>***Have you actually had any thoughts of killing yourself?***<br><br>If yes, describe:	**Yes** ☐ **No** ☐	**Yes** ☐ **No** ☐
**3. Active Suicidal Ideation with Any Methods (Not Plan) without Intent to Act**<br>Subject endorses thoughts of suicide and has thought of at least one method during the assessment period. This is different than a specific plan with time, place or method details worked out (e.g. thought of method to kill self but not a specific plan). Includes person who would say, *"I thought about taking an overdose but I never made a specific plan as to when, where or how I would actually do it…and I would never go through with it."*<br>***Have you been thinking about how you might do this?***<br><br>If yes, describe:	**Yes** ☐ **No** ☐	**Yes** ☐ **No** ☐
**4. Active Suicidal Ideation with Some Intent to Act, without Specific Plan**<br>Active suicidal thoughts of killing oneself and subject reports having <u>some intent to act on such thoughts</u>, as opposed to *"I have the thoughts but I definitely will not do anything about them."*<br>***Have you had these thoughts and had some intention of acting on them?***<br><br>If yes, describe:	**Yes** ☐ **No** ☐	**Yes** ☐ **No** ☐
**5. Active Suicidal Ideation with Specific Plan and Intent**<br>Thoughts of killing oneself with details of plan fully or partially worked out and subject has some intent to carry it out.<br>***Have you started to work out or worked out the details of how to kill yourself? Do you intend to carry out this plan?***<br><br>If yes, describe:	**Yes** ☐ **No** ☐	**Yes** ☐ **No** ☐
INTENSITY OF IDEATION		
The following features should be rated with respect to the most severe type of ideation (i.e., 1-5 from above, with 1 being the least severe and 5 being the most severe). Ask about time he/she was feeling the most suicidal.<br><br><u>Lifetime</u> - ***Most Severe Ideation:*** ______ *Type # (1-5)* ____________ *Description of Ideation*<br><br><u>Past X Months</u> - ***Most Severe Ideation:*** ______ *Type # (1-5)* ____________ *Description of Ideation*	Most Severe	Most Severe
**Frequency**<br>***How many times have you had these thoughts?***<br>(1) Less than once a week (2) Once a week (3) 2-5 times in week (4) Daily or almost daily (5) Many times each day	___	___
**Duration**<br>***When you have the thoughts how long do they last?***<br>(1) Fleeting - few seconds or minutes<br>(2) Less than 1 hour/some of the time<br>(3) 1-4 hours/a lot of time<br>(4) 4-8 hours/most of day<br>(5) More than 8 hours/persistent or continuous	___	___
**Controllability**<br>***Could/can you stop thinking about killing yourself or wanting to die if you want to?***<br>(1) Easily able to control thoughts<br>(2) Can control thoughts with little difficulty<br>(3) Can control thoughts with some difficulty<br>(4) Can control thoughts with a lot of difficulty<br>(5) Unable to control thoughts<br>(0) Does not attempt to control thoughts	___	___
**Deterrents**<br>***Are there things - anyone or anything (e.g., family, religion, pain of death) - that stopped you from wanting to die or acting on thoughts of committing suicide?***<br>(1) Deterrents definitely stopped you from attempting suicide<br>(2) Deterrents probably stopped you<br>(3) Uncertain that deterrents stopped you<br>(4) Deterrents most likely did not stop you<br>(5) Deterrents definitely did not stop you<br>(0) Does not apply	___	___
**Reasons for Ideation**<br>***What sort of reasons did you have for thinking about wanting to die or killing yourself? Was it to end the pain or stop the way you were feeling (in other words you couldn't go on living with this pain or how you were feeling) or was it to get attention, revenge or a reaction from others? Or both?***<br>(1) Completely to get attention, revenge or a reaction from others<br>(2) Mostly to get attention, revenge or a reaction from others<br>(3) Equally to get attention, revenge or a reaction from others and to end/stop the pain<br>(4) Mostly to end or stop the pain (you couldn't go on living with the pain or how you were feeling)<br>(5) Completely to end or stop the pain (you couldn't go on living with the pain or how you were feeling)<br>(0) Does not apply	___	___

© 2008 Research Foundation for Mental Hygiene, Inc. C-SSRS—Baseline/Screening (Version 1/14/09) Page 1 of 2

SUICIDAL BEHAVIOR *(Check all that apply, so long as these are separate events; must ask about all types)*	**Lifetime**	**Past __ Years**
**Actual Attempt:**<br>A potentially self-injurious act committed with at least some wish to die, *as a result of act*. Behavior was in part thought of as method to kill oneself. Intent does not have to be 100%. If there is ***any*** intent/desire to die associated with the act, then it can be considered an actual suicide attempt. ***There does not have to be any injury or harm***, just the potential for injury or harm. If person pulls trigger while gun is in mouth but gun is broken so no injury results, this is considered an attempt.<br>Inferring Intent: Even if an individual denies intent/wish to die, it may be inferred clinically from the behavior or circumstances. For example, a highly lethal act that is clearly not an accident so no other intent but suicide can be inferred (e.g., gunshot to head, jumping from window of a high floor/story). Also, if someone denies intent to die, but they thought that what they did could be lethal, intent may be inferred.<br>***Have you made a suicide attempt?***<br>***Have you done anything to harm yourself?***<br>***Have you done anything dangerous where you could have died?***<br>***What did you do?***<br>***Did you______ as a way to end your life?***<br>***Did you want to die (even a little) when you_____?***<br>***Were you trying to end your life when you _____?***<br>***Or Did you think it was possible you could have died from_____?***<br>***Or did you do it purely for other reasons / without ANY intention of killing yourself (like to relieve stress, feel better, get sympathy, or get something else to happen)?*** (Self-Injurious Behavior without suicidal intent)<br>If yes, describe:	Yes ☐ No ☐<br>Total # of Attempts<br>_____	Yes ☐ No ☐<br>Total # of Attempts<br>_____
**Has subject engaged in Non-Suicidal Self-Injurious Behavior?**	Yes ☐ No ☐	Yes ☐ No ☐
**Interrupted Attempt:**<br>When the person is interrupted (by an outside circumstance) from starting the potentially self-injurious act *(if not for that, actual attempt would have occurred).*<br>Overdose: Person has pills in hand but is stopped from ingesting. Once they ingest any pills, this becomes an attempt rather than an interrupted attempt. Shooting: Person has gun pointed toward self, gun is taken away by someone else, or is somehow prevented from pulling trigger. Once they pull the trigger, even if the gun fails to fire, it is an attempt. Jumping: Person is poised to jump, is grabbed and taken down from ledge. Hanging: Person has noose around neck but has not yet started to hang - is stopped from doing so.<br>***Has there been a time when you started to do something to end your life but someone or something stopped you before you actually did anything?***<br>If yes, describe:	Yes ☐ No ☐<br>Total # of interrupted<br>_____	Yes ☐ No ☐<br>Total # of interrupted<br>_____
**Aborted Attempt:**<br>When person begins to take steps toward making a suicide attempt, but stops themselves before they actually have engaged in any self-destructive behavior. Examples are similar to interrupted attempts, except that the individual stops him/herself, instead of being stopped by something else.<br>***Has there been a time when you started to do something to try to end your life but you stopped yourself before you actually did anything?***<br>If yes, describe:	Yes ☐ No ☐<br>Total # of aborted<br>_____	Yes ☐ No ☐<br>Total # of aborted<br>_____
**Preparatory Acts or Behavior:**<br>Acts or preparation towards imminently making a suicide attempt. This can include anything beyond a verbalization or thought, such as assembling a specific method (e.g., buying pills, purchasing a gun) or preparing for one's death by suicide (e.g., giving things away, writing a suicide note).<br>***Have you taken any steps towards making a suicide attempt or preparing to kill yourself (such as collecting pills, getting a gun, giving valuables away or writing a suicide note)?***<br>If yes, describe:	Yes ☐ No ☐	Yes ☐ No ☐
**Suicidal Behavior:**<br>Suicidal behavior was present during the assessment period?	Yes ☐ No ☐	Yes ☐ No ☐

Answer for Actual Attempts Only	Most Recent Attempt Date:	Most Lethal Attempt Date:	Initial/First Attempt Date:
**Actual Lethality/Medical Damage:**<br>0. No physical damage or very minor physical damage (e.g., surface scratches).<br>1. Minor physical damage (e.g., lethargic speech; first-degree burns; mild bleeding; sprains).<br>2. Moderate physical damage; medical attention needed (e.g., conscious but sleepy, somewhat responsive; second-degree burns; bleeding of major vessel).<br>3. Moderately severe physical damage; *medical* hospitalization and likely intensive care required (e.g., comatose with reflexes intact; third-degree burns less than 20% of body; extensive blood loss but can recover; major fractures).<br>4. Severe physical damage; *medical* hospitalization with intensive care required (e.g., comatose without reflexes; third-degree burns over 20% of body; extensive blood loss with unstable vital signs; major damage to a vital area).<br>5. Death	*Enter Code*<br>_____	*Enter Code*<br>_____	*Enter Code*<br>_____
**Potential Lethality: Only Answer if Actual Lethality=0**<br>Likely lethality of actual attempt if no medical damage (the following examples, while having no actual medical damage, had potential for very serious lethality: put gun in mouth and pulled the trigger but gun fails to fire so no medical damage; laying on train tracks with oncoming train but pulled away before run over).<br>0 = Behavior not likely to result in injury<br>1 = Behavior likely to result in injury but not likely to cause death<br>2 = Behavior likely to result in death despite available medical care	*Enter Code*<br>_____	*Enter Code*<br>_____	*Enter Code*<br>_____

© 2008 Research Foundation for Mental Hygiene, Inc.

COLUMBIA-SUICIDE SEVERITY RATING SCALE (C-SSRS)

Since Last Visit

Version 1/14/09

Posner, K.; Brent, D.; Lucas, C.; Gould, M.; Stanley, B.; Brown, G.; Fisher, P.; Zelazny, J.; Burke, A.; Oquendo, M.; Mann, J.

Disclaimer:

This scale is intended to be used by individuals who have received training in its administration. The questions contained in the Columbia-Suicide Severity Rating Scale are suggested probes. Ultimately, the determination of the presence of suicidal ideation or behavior depends on the judgment of the individual administering the scale.

Definitions of behavioral suicidal events in this scale are based on those used in **The Columbia Suicide History Form**, *developed by John Mann, MD and Maria Oquendo, MD, Conte Center for the Neuroscience of Mental Disorders (CCNMD), New York State Psychiatric Institute, 1051 Riverside Drive, New York, NY, 10032. (Oquendo M. A., Halberstam B. & Mann J. J., Risk factors for suicidal behavior: utility and limitations of research instruments. In M.B. First [Ed.]* Standardized Evaluation in Clinical Practice, *pp. 103 -130, 2003.)*

For reprints of the C-SSRS contact Kelly Posner, Ph.D., New York State Psychiatric Institute, 1051 Riverside Drive, New York, New York, 10032; inquiries and training requirements contact posnerk@nyspi.columbia.edu

© 2008 The Research Foundation for Mental Hygiene, Inc.

SUICIDAL IDEATION

Ask questions 1 and 2. If both are negative, proceed to "Suicidal Behavior" section. If the answer to question 2 is "yes", ask questions 3, 4 and 5. If the answer to question 1 and/or 2 is "yes", complete "Intensity of Ideation" section below.	**Since Last Visit**
**1. Wish to be Dead**<br>Subject endorses thoughts about a wish to be dead or not alive anymore, or wish to fall asleep and not wake up.<br>***Have you wished you were dead or wished you could go to sleep and not wake up?***<br><br>If yes, describe:	**Yes** ☐ **No** ☐
**2. Non-Specific Active Suicidal Thoughts**<br>General, non-specific thoughts of wanting to end one's life/commit suicide (e.g., *"I've thought about killing myself"*) without thoughts of ways to kill oneself/associated methods, intent, or plan during the assessment period.<br>***Have you actually had any thoughts of killing yourself?***<br><br>If yes, describe:	**Yes** ☐ **No** ☐
**3. Active Suicidal Ideation with Any Methods (Not Plan) without Intent to Act**<br>Subject endorses thoughts of suicide and has thought of at least one method during the assessment period. This is different than a specific plan with time, place or method details worked out (e.g., thought of method to kill self but not a specific plan). Includes person who would say, *"I thought about taking an overdose but I never made a specific plan as to when, where or how I would actually do it…and I would never go through with it."*<br>***Have you been thinking about how you might do this?***<br><br>If yes, describe:	**Yes** ☐ **No** ☐
**4. Active Suicidal Ideation with Some Intent to Act, without Specific Plan**<br>Active suicidal thoughts of killing oneself and subject reports having some intent to act on such thoughts, as opposed to *"I have the thoughts but I definitely will not do anything about them."*<br>***Have you had these thoughts and had some intention of acting on them?***<br><br>If yes, describe:	**Yes** ☐ **No** ☐
**5. Active Suicidal Ideation with Specific Plan and Intent**<br>Thoughts of killing oneself with details of plan fully or partially worked out and subject has some intent to carry it out.<br>***Have you started to work out or worked out the details of how to kill yourself? Do you intend to carry out this plan?***<br><br>If yes, describe:	**Yes** ☐ **No** ☐

INTENSITY OF IDEATION

The following features should be rated with respect to the most severe type of ideation (i.e., 1-5 from above, with 1 being the least severe and 5 being the most severe).<br><br>***Most Severe Ideation:*** ____ ____________________<br>*Type # (1-5)* *Description of Ideation*	Most Severe
**Frequency**<br>***How many times have you had these thoughts?***<br>(1) Less than once a week (2) Once a week (3) 2-5 times in week (4) Daily or almost daily (5) Many times each day	____
**Duration**<br>***When you have the thoughts, how long do they last?***<br>(1) Fleeting - few seconds or minutes<br>(2) Less than 1 hour/some of the time<br>(3) 1-4 hours/a lot of time<br>(4) 4-8 hours/most of day<br>(5) More than 8 hours/persistent or continuous	____
**Controllability**<br>***Could/can you stop thinking about killing yourself or wanting to die if you want to?***<br>(1) Easily able to control thoughts<br>(2) Can control thoughts with little difficulty<br>(3) Can control thoughts with some difficulty<br>(4) Can control thoughts with a lot of difficulty<br>(5) Unable to control thoughts<br>(0) Does not attempt to control thoughts	____
**Deterrents**<br>***Are there things - anyone or anything (e.g., family, religion, pain of death) - that stopped you from wanting to die or acting on thoughts of committing suicide?***<br>(1) Deterrents definitely stopped you from attempting suicide<br>(2) Deterrents probably stopped you<br>(3) Uncertain that deterrents stopped you<br>(4) Deterrents most likely did not stop you<br>(5) Deterrents definitely did not stop you<br>(0) Does not apply	____
**Reasons for Ideation**<br>***What sort of reasons did you have for thinking about wanting to die or killing yourself? Was it to end the pain or stop the way you were feeling (in other words you couldn't go on living with this pain or how you were feeling) or was it to get attention, revenge or a reaction from others? Or both?***<br>(1) Completely to get attention, revenge or a reaction from others<br>(2) Mostly to get attention, revenge or a reaction from others<br>(3) Equally to get attention, revenge or a reaction from others and to end/stop the pain<br>(4) Mostly to end or stop the pain (you couldn't go on living with the pain or how you were feeling)<br>(5) Completely to end or stop the pain (you couldn't go on living with the pain or how you were feeling)<br>(0) Does not apply	____

© 2008 Research Foundation for Mental Hygiene, Inc. C-SSRS—Since Last Visit (Version 1/14/09) Page 1 of 2

SUICIDAL BEHAVIOR *(Check all that apply, so long as these are separate events; must ask about all types)*	**Since Last Visit**
**Actual Attempt:**<br>A potentially self-injurious act committed with at least some wish to die, *as a result of act.* Behavior was in part thought of as method to kill oneself. Intent does not have to be 100%. If there is ***any*** intent/desire to die associated with the act, then it can be considered an actual suicide attempt. ***There does not have to be any injury or harm,*** just the potential for injury or harm. If person pulls trigger while gun is in mouth but gun is broken so no injury results, this is considered an attempt.<br>Inferring Intent: Even if an individual denies intent/wish to die, it may be inferred clinically from the behavior or circumstances. For example, a highly lethal act that is clearly not an accident so no other intent but suicide can be inferred (e.g., gunshot to head, jumping from window of a high floor/story). Also, if someone denies intent to die, but they thought that what they did could be lethal, intent may be inferred.<br>***Have you made a suicide attempt?***<br>***Have you done anything to harm yourself?***<br>***Have you done anything dangerous where you could have died?***<br>***What did you do?***<br>***Did you______ as a way to end your life?***<br>***Did you want to die (even a little) when you_____?***<br>***Were you trying to end your life when you _____?***<br>***Or did you think it was possible you could have died from_____?***<br>***Or did you do it purely for other reasons / without ANY intention of killing yourself (like to relieve stress, feel better, get sympathy, or get something else to happen)?*** (Self-Injurious Behavior without suicidal intent)<br>If yes, describe:	**Yes** **No**<br>☐ ☐<br><br>Total # of Attempts<br>_____
**Has subject engaged in Non-Suicidal Self-Injurious Behavior?**	**Yes** **No**<br>☐ ☐
**Interrupted Attempt:**<br>When the person is interrupted (by an outside circumstance) from starting the potentially self-injurious act *(if not for that, actual attempt would have occurred).*<br>Overdose: Person has pills in hand but is stopped from ingesting. Once they ingest any pills, this becomes an attempt rather than an interrupted attempt. Shooting: Person has gun pointed toward self, gun is taken away by someone else, or is somehow prevented from pulling trigger. Once they pull the trigger, even if the gun fails to fire, it is an attempt. Jumping: Person is poised to jump, is grabbed and taken down from ledge. Hanging: Person has noose around neck but has not yet started to hang - is stopped from doing so.<br>***Has there been a time when you started to do something to end your life but someone or something stopped you before you actually did anything?***<br>If yes, describe:	**Yes** **No**<br>☐ ☐<br><br>Total # of interrupted<br>_____
**Aborted Attempt:**<br>When person begins to take steps toward making a suicide attempt, but stops themselves before they actually have engaged in any self-destructive behavior. Examples are similar to interrupted attempts, except that the individual stops him/herself, instead of being stopped by something else.<br>***Has there been a time when you started to do something to try to end your life but you stopped yourself before you actually did anything?***<br>If yes, describe:	**Yes** **No**<br>☐ ☐<br><br>Total # of aborted<br>_____
**Preparatory Acts or Behavior:**<br>Acts or preparation towards imminently making a suicide attempt. This can include anything beyond a verbalization or thought, such as assembling a specific method (e.g., buying pills, purchasing a gun) or preparing for one's death by suicide (e.g., giving things away, writing a suicide note).<br>***Have you taken any steps towards making a suicide attempt or preparing to kill yourself (such as collecting pills, getting a gun, giving valuables away or writing a suicide note)?***<br>If yes, describe:	**Yes** **No**<br>☐ ☐
**Suicidal Behavior:**<br>Suicidal behavior was present during the assessment period?	**Yes** **No**<br>☐ ☐
**Suicide:**	**Yes** **No**<br>☐ ☐
Answer for Actual Attempts Only	Most Lethal Attempt Date:
**Actual Lethality/Medical Damage:**<br>0. No physical damage or very minor physical damage (e.g., surface scratches).<br>1. Minor physical damage (e.g., lethargic speech; first-degree burns; mild bleeding; sprains).<br>2. Moderate physical damage; medical attention needed (e.g., conscious but sleepy, somewhat responsive; second-degree burns; bleeding of major vessel).<br>3. Moderately severe physical damage; *medical* hospitalization and likely intensive care required (e.g., comatose with reflexes intact; third-degree burns less than 20% of body; extensive blood loss but can recover; major fractures).<br>4. Severe physical damage; *medical* hospitalization with intensive care required (e.g., comatose without reflexes; third-degree burns over 20% of body; extensive blood loss with unstable vital signs; major damage to a vital area).<br>5. Death	*Enter Code*<br>_____
**Potential Lethality: Only Answer if Actual Lethality=0**<br>Likely lethality of actual attempt if no medical damage (the following examples, while having no actual medical damage, had potential for very serious lethality: put gun in mouth and pulled the trigger but gun fails to fire so no medical damage; laying on train tracks with oncoming train but pulled away before run over).<br>0 = Behavior not likely to result in injury<br>1 = Behavior likely to result in injury but not likely to cause death<br>2 = Behavior likely to result in death despite available medical care	*Enter Code*<br>_____

© 2008 Research Foundation for Mental Hygiene, Inc. C-SSRS—Since Last Visit (Version 1/14/09) Page 2 of 2

APPENDIX P. PARKINSON'S DISEASE DIARY

PARKINSON'S DISEASE DIARY

NAME ______________________________ DATE ________________

Instructions: For each half-hour time period place one check mark to indicate your predominant states during most of that period.
ON = Time when medication is providing benefit with regard to mobility, slowness, and stiffness.
OFF = Time when medication has worn off and is no longer providing benefit with regard to mobility, slowness, and stiffness.
Dyskinesia = involuntary twisting, turning movements. These movements are an effect of medication and occur during ON time.
Non-troublesome dyskinesia does not interfere with function or cause meaningful discomfort. Troublesome dyskinesia interferes with function or causes meaningful discomfort.
Tremor is shaking back and forth and is not considered dyskinesia.

time	asleep	OFF	ON without dyskinesia	ON with non-troublesome dyskinesia	ON with troublesome dyskinesia
6:00 AM					
:30					
7:00 AM					
:30					
8:00 AM					
:30					
9:00 AM					
:30					
10:00 AM					
:30					
11:00 AM					
:30					
12:00 PM					
:30					
1:00 PM					
:30					
2:00 PM					
:30					
3:00 PM					
:30					
4:00 PM					
:30					
5:00 PM					
:30					

time	asleep	OFF	ON without dyskinesia	ON with non-troublesome dyskinesia	ON with troublesome dyskinesia
6:00 PM					
:30					
7:00 PM					
:30					
8:00 PM					
:30					
9:00 PM					
:30					
10:00 PM					
:30					
11:00 PM					
:30					
12:00 AM					
:30					
1:00 AM					
:30					
2:00 AM					
:30					
3:00 AM					
:30					
4:00 AM					
:30					
5:00 AM					
:30					

© RA Hauser 1995

APPENDIX Q. EXCIPIENTS IN IPX203, IPX203 PLACEBO, IR CD-LD, AND IR PLACEBO

IPX203 (Various Strengths)	IPX203 Placebo	IR CD-LD (25-100 mg)	IR Placebo
Microcrystalline Cellulose, NF	Microcrystalline Cellulose, NF	Crospovidone	Microcrystalline cellulose, NF
Croscarmellose Sodium, NF	Talc, USP	Hydroxypropyl Cellulose	Magnesium Stearate, NF
Magnesium Stearate, NF	Magnesium Stearate, NF	Magnesium Stearate	Quinoline yellow E104
Mannitol, USP	Sugar Spheres, NF	Microcrystalline cellulose	
Sodium Lauryl Sulfate, NF	Methacrylic acid copolymer Type A, NF	Starch (corn)	
Povidone, USP	Triethyl citrate, NF	D&C Yellow No. 10	
Cellulose Acetate	Hard gelatin capsules	Aluminum Oxide	
Copovidone, NF			
Amino Methacrylate Copolymer, NF			
Methacrylic acid copolymer Type A, NF			
Triethyl Citrate, NF			
Talc, USP			
Hard gelatin capsules			

APPENDIX R. CLINICAL LABORATORY STUDIES

HEMATOLOGY

hemoglobin	% lymphocytes	absolute lymphocytes
hematocrit	% monocytes	absolute monocytes
red blood cell count	% basophils	absolute basophils
white blood cell count	% eosinophils	absolute eosinophils
% neutrophils	absolute neutrophils	platelet count

CHEMISTRY

sodium	calcium	indirect bilirubin
potassium	phosphorous	alkaline phosphatase
chloride	albumin	alanine aminotransferase (ALT, SGPT)
carbon dioxide	total protein	aspartate aminotransferase (AST, SGOT)
blood urea nitrogen (BUN)	uric acid	creatine phosphokinase
creatinine	total bilirubin	lactate dehydrogenase
glucose	direct bilirubin	

URINALYSIS

pH	ketones	leukocyte esterase
specific gravity	microscopic exam (RBC and WBC, only when indicated)	protein
blood		
glucose		

URINE DRUG TEST

amphetamines	benzodiazepines
barbiturates	
cannabinoids	
cocaine metabolites	
opiates	
phencyclidines	

ALCOHOL BREATH TEST

PREGNANCY TEST
Urine pregnancy test (to be completed on site) for female subjects of childbearing potential.

IPX203 (CARBIDOPA-LEVODOPA) EXTENDED-RELEASE CAPSULES

IPX203-B16-02

A RANDOMIZED CONTROLLED STUDY TO COMPARE THE SAFETY AND EFFICACY OF IPX203 WITH IMMEDIATE-RELEASE CARBIDOPA-LEVODOPA IN PARKINSON'S DISEASE PATIENTS WITH MOTOR FLUCTUATIONS

SPONSOR

Impax Laboratories, LLC

400 Crossing Boulevard, Third Floor

Bridgewater, NJ 08807-2863

Original Protocol, May 18, 2017

Amendment 1, August 30, 2017

Amendment 2, October 23, 2017

Amendment 3, December 07, 2017

Amendment 4, September 28, 2018

CONFIDENTIALITY STATEMENT

All information provided is the property of Impax Laboratories, LLC and may not be divulged, published or otherwise disclosed without written consent of Impax. All information provided to the Investigator by the Sponsor, including clinical observations at the investigative site, shall be held strictly confidential and confined to the clinical personnel involved in conducting the study, under the supervision of the Investigator. This includes but is not limited to preclinical data, protocols, case report forms, and verbal or written communications. This information may be related in confidence to the Institutional Review Board or other committees functioning in a similar capacity. All reports, subject samples, data published or submitted to third parties will be identified by a coded number and initials only in order to maintain subject confidentiality.

SIGNATURE PAGE

Reviewed and approved by:

[signature]	28 SEP 2018
Robert Rubens, MD, MBA<br>Senior Director, Clinical Development	Date
[signature]	28 Sep 2018
Barbara Pruitt, RN, BS<br>Associate Director, Clinical Operations	Date
[signature]	28 Sep 18
Yoshiko Stowell, PhD<br>Manager, Regulatory Affairs	Date
[signature]	01 OCT 2018
Sarita Khanna, PhD<br>Senior Director, Biostatistics	Date
[signature]	28 SEP 2018
Nishit Modi, PhD<br>Vice President, Clinical Pharmacology	Date

INVESTIGATOR'S AGREEMENT

Protocol No.: IPX203-B16-02

Protocol Title: A Randomized Controlled Study to Compare the Safety and Efficacy of IPX203 with Immediate-Release Carbidopa-Levodopa in Parkinson's Disease Patients with Motor Fluctuations

I have read this protocol and agree to conduct the study as outlined herein, complying with the obligations and requirements of clinical investigators and all other requirements of International Conference on Harmonization (ICH), Good Clinical Practice (GCP), and the appropriate regulatory authority.

I will provide copies of the protocol and all pertinent information to all individuals responsible to me who assist in the conduct of this clinical study. I will discuss this material with them to ensure that they are fully informed regarding the study medication, the conduct of the study, and the obligations of confidentiality.

______________________________________ ____________________

Principal Investigator's signature Date

____

Principal Investigator's printed name

STUDY CONTACT INFORMATION

Changes in Impax study personnel listed on this page do not require a protocol amendment.

Role	Name and Contact Information
Sponsor	Impax Laboratories, LLC<br>400 Crossing Boulevard, Third Floor<br>Bridgewater, NJ 08807-2863<br>(732) 595-4600
Medical Monitor	Robert Rubens, MD, MBA<br>Senior Director, Clinical Research & Development<br>Mobile: (510) 303-8207<br>Office: (510) 240-6012<br>E-mail: rrubens@impaxlabs.com
Statistician	Sarita Khanna, PhD<br>Senior Director, Biostatistics<br>(510) 240-6425<br>E-mail: skhanna@impaxlabs.com
Clinical Pharmacology	Nishit Modi, PhD<br>Vice President, Clinical Pharmacology<br>(510) 240-6004<br>E-mail: NModi@impaxlabs.com
Clinical Research Associates	Barbara Pruitt, RN, BS<br>Associate Director, Clinical Operations<br>Office: (510) 240-6003<br>Mobile: (510) 329-8489<br>E-mail: bpruitt@impaxlabs.com
Serious Adverse Event Reporting	FAX: (866) 954-2064

1. SYNOPSIS

**Name of Sponsor/Company:** Impax Laboratories, LLC (Impax)
**Name of Investigational Product:** IPX203 (carbidopa-levodopa) Extended-Release Capsules
**Name of Active Ingredients:** carbidopa (CD), levodopa (LD)
**Protocol Title:** A Randomized Controlled Study to Compare the Safety and Efficacy of IPX203 with Immediate-Release Carbidopa-Levodopa in Parkinson's Disease Patients with Motor Fluctuations
**Protocol No.:** IPX203-B16-02
**Study center(s):** Multicenter
**Phase of Development:** Phase 3
**Objectives:** To evaluate the safety and efficacy of IPX203 in comparison to IR CD-LD in the treatment of CD-LD-experienced subjects with Parkinson's disease (PD) who have motor fluctuations.
**Methodology:** This is a multicenter, randomized, double-blind, double-dummy, active-controlled, parallel-group study. The study will consist of a 3-week, open-label IR CD-LD dose adjustment period; a 4-week, open-label period for conversion to IPX203; followed by a 13-week double-blind treatment period with subjects randomized in a 1:1 ratio, stratified by center, to receive either IPX203 (with matching IR CD-LD placebo) or IR CD-LD (with matching IPX203 placebo).<br>Subjects will continue to take permitted non-CD-LD-based PD medications throughout the study if documented in their prestudy regimen and if dosing regimens have been stable for at least 4 weeks prior to Visit 1. A "stable dosing regimen" means no change in dose or in dosing frequency.<br>• Within 4 weeks following the Screening visit, eligible subjects will complete their PD Diaries on each of the 3 consecutive days immediately prior to Visit 1.<br>• Following Visit 1, qualified subjects will enter a 3-week, open-label IR CD-LD treatment period allowing for dose adjustment. **The dosing regimen of IR CD-LD may be adjusted during the dose adjustment period** to minimize "Off" time without causing troublesome dyskinesia. The doses and regimens of the subject's other non-CD-LD PD medications (dopamine agonists, MAO-B inhibitors, amantadine, anticholinergics) should remain stable throughout this study. Any adjustments to the IR CD-LD dosing regimen will be done in consultation with the Investigator or qualified site personnel and will be recorded. The IR CD-LD dosing regimen should be stable for at least 5 days prior to returning for Visit 2. Rescue with additional or modified doses of concomitant PD medications or with use of CD-LD products other than the dispensed study medications is not permitted and will trigger discontinuation from the study. Subjects will complete their 3-day PD Diaries on each of the 3 consecutive days immediately prior to Visit 2.<br>• Following completion of the IR CD-LD dose adjustment period, subjects will begin a 4-week open-label period for conversion to IPX203. The initial dosing regimen of IPX203 will be based on the most frequent dose of the subject's dosing regimen of IR CD-LD at the end of the dose adjustment period (Visit 2). A 25-100 mg dose of IR CD-LD converts to a 70-280 mg CD-LD dose of IPX203 (2 capsules of 35-140 mg CD-LD IPX203), and a 12.5-50 mg dose of IR CD-LD converts to a 35-140 mg CD-LD dose of IPX203, but with a longer duration of effect. It is recommended that IPX203 should be dosed approximately every 8 hours with the exception that subjects who are currently receiving a total daily dose of less than 125-500 mg IR CD-LD at the end of the dose adjustment period will be initially administered every

12 hours. The dosing interval may be reduced to approximately every 8 hours if the subject does not achieve an acceptable duration of effect. **The dosing regimen of IPX203 may be adjusted during the dose conversion period** to achieve the optimal balance of efficacy and tolerability (minimize "Off" time without causing troublesome dyskinesia or other dopaminergic side effects). The doses and regimens of the subject's other non-CD-LD PD medications (dopamine agonists, MAO-B inhibitors, amantadine, anticholinergics) should remain stable throughout this study. The subject must be on a stable dosing regimen of IPX203 (no change in dose or in dosing frequency) for at least 5 days prior to returning for Visit 4. Any adjustments to the IPX203 dosing regimen will be done in consultation with the Investigator or qualified site personnel and will be recorded. Rescue with additional or modified doses of concomitant PD medications or with use of CD-LD products other than the dispensed study medications is not permitted and will trigger discontinuation from the study. Subjects will return to the clinic in 2 weeks for Visit 3 followed by Visit 4, 2 weeks later. Subjects will complete their 3-day PD Diaries on each of the 3 consecutive days immediately prior to Visit 4.

- Subjects who successfully complete the IPX203 dose conversion period will be randomized in 1:1 ratio, stratified by center, at Visit 4 into one of two parallel treatment arms of IPX203 (with matching IR CD-LD placebo) or IR CD-LD (with matching IPX203 placebo). The subjects will undergo 13 weeks of double-blind maintenance therapy with the stable dosing regimen established at the end of Week 3 (Visit 2) for IR CD-LD and at the end of Week 7 (Visit 4) for IPX203. Rescue with additional or modified doses of concomitant PD medications or with use of CD-LD products other than the dispensed study medications is not permitted and will trigger discontinuation from the study. Subjects will return to the clinic for 3 visits (Visits 5, 6, and 7) and will complete their 3-day PD Diaries on each of the 3 consecutive days immediately prior to each of these visits.

Number of patients (planned): Assuming approximately an 18% prerandomization drop-out, approximately 510 subjects will be enrolled to randomize 420 subjects.

Diagnosis and main criteria for inclusion:

Inclusion Criteria

- Male or female subjects diagnosed at age ≥ 40 years with PD, consistent with the United Kingdom Parkinson's Disease Society Brain Bank Diagnostic Criteria and who are being treated with stable regimens of CD-LD but experiencing motor fluctuations.
- Hoehn and Yahr Stages 1, 2, 3, or 4 in the "On" state (part of Movement Disorders Society version of the Unified Parkinson's Disease Rating Scale [MDS-UPDRS] Part III)
- Montreal Cognitive Assessment (MoCA) score ≥ 24 at Screening Visit in "On" state.
- By history, for the 4 weeks prior to Screening, the subject experiences daily "wearing-off" episodes with periods of bradykinesia in combination with at least one of rest tremor or rigidity, experiences an "Off" state upon awakening on most mornings, and reports an average of at least 2.5 cumulative hours per day of "Off" time during the waking hours.
- Able to differentiate "On" state from "Off" state as determined by at least 75% concordance with a trained rater in "On/Off" ratings for 8 ratings over a 4-hour training period. The concordance must include at least 1 "On" and 1 "Off" rating and must be achieved within two 4-hour training sessions.
- At Visit 1, review of the 3-day PD Diaries confirms the following: that the subject is able to properly complete the Diaries with valid entries; and that the subject has an average of at least 2.5 hours per day of "Off" time during waking hours over the 3 days with at least 1.5 hours of cumulative "Off" time on each day.

- Responsive to CD-LD therapy and currently being treated on a stable regimen with CD-LD for at least 4 weeks prior to Visit 1 and:
  - Requires at least 100 mg of LD from IR CD-LD for the first morning dose
  - Requires a total daily dose of at least 400 mg of LD and takes a maximum total daily dose of 2400 mg LD, from IR CD-LD alone or IR CD-LD in combination with a single daily bedtime dose of CR CD-LD
  - Has a dosing frequency of 4 to 9 times daily of CD-LD
  - By history, typically experiences an “On” response with the first dose of IR CD-LD of the day, but the efficacy of this dose typically lasts less than 4 hours.
- At Screening, the subject has predictable “Off” periods defined by a score of 1 or 2 on Item #4.5 (Complexity of Motor Fluctuations) of the MDS-UPDRS Part IV B (Motor Fluctuations).
- At Screening, the MDS-UPDRS Part III total score in the “Off” state is at least 20 units.

Exclusion Criteria

- Used any doses of controlled-release (CR) CD-LD apart from a single daily bedtime dose within 4 weeks prior to Visit 1.
- Used any dose of Rytary for the past 4 weeks prior to Visit 1 or were considered IPX066 or Rytary failures for reasons of efficacy or safety.
- Had prior neurosurgical treatment for PD or if such procedure is planned or anticipated during the study period.
- Allergic to any excipient in the study drugs.
- History of glaucoma with intraocular pressures that are elevated despite appropriate medical management.
- History of seizure or epilepsy and experienced at least 1 seizure during the past 12 months or has not been compliant with medically recommended therapy or visits.
- History of myocardial infarction with residual atrial, nodal, or ventricular arrhythmias that are not controlled with medical and/or surgical interventions. A recent (≤ 12 months) history of myocardial infarction with secondary arrhythmias is exclusionary regardless of the therapeutic control.
- Received within 4 weeks of Screening or planning to take during participation in the clinical study:
  - Any doses of a CR CD-LD apart from a single daily bedtime dose, any doses of Rytary, additional CD (eg, Lodosyn) or benserazide (eg, Serazide), or catechol-O-methyl transferase inhibitors (entacapone or tolcapone) or medications containing these inhibitors (Stalevo),
  - Nonselective monoamine oxidase inhibitors (MAOI), apomorphine, or antidopaminergic agents, including antiemetics.
- Subjects who have previously participated in an IPX203 study.

Investigational product, dosage and mode of administration: IPX203 (carbidopa-levodopa) Extended-Release capsules, containing 35-140 mg of CD-LD and matching placebo, for oral administration.

Reference therapy, dosage and mode of administration: Immediate-Release carbidopa-levodopa (IR CD-LD) tablets containing 25-100 mg of CD-LD, and matching placebo, for oral administration.

Duration of treatment: Approximately 24 weeks, including up to 4 weeks following Screening, 3 weeks of IR CD-LD dose adjustment, 4 weeks of IPX203 dose conversion, and 13 weeks of double-

blind therapy following randomization.

Criteria for evaluation:

Baseline is defined as assessments done at Visit 4 (randomization visit). Study Entry is defined as assessments done at Visit 1 (study entry visit).

Efficacy:

- Primary endpoint: Change from baseline in "Good on" time in hours per day, averaged over the PD Diary days, at the end of double-blind treatment period (Visit 7 or early termination). "Good on" time is derived from the 3-day PD Diaries and is defined as the sum of "On" time without dyskinesia and "On" time with nontroublesome dyskinesia.
- Key secondary endpoints:
  - Change from baseline in "Off" time in hours per day, averaged over the PD Diary days at the end of double-blind treatment period (Visit 7 or early termination)
  - Proportion of subjects with either "much improved" or "very much improved" in Patient Global Impression of Change (PGI-C) scores at the end of double-blind treatment period (Visit 7 or early termination)
  - Change from baseline in the MDS-UPDRS Part III at the end of double-blind treatment period (Visit 7 or early termination)
  - Change from baseline in the sum of MDS-UPDRS Parts II and III at the end of double-blind treatment period (Visit 7 or early termination)
- Additional endpoints:

  The following endpoints will be evaluated (at the post-randomization visits) as change from baseline (Visit 4) as well as change from the study entry (Visit 1), when applicable, by visits:
  - Percent "Off" time during waking hours derived from the 3-day PD Diaries
  - Average duration of each continuous "Good on" and each continuous "On"
  - Hours of (1) "Off" time (from Visit 1), (2) "Good on" time (from Visit 1), (3) "On" time with dyskinesia, (4) "On" time with troublesome dyskinesia, and (5) "On" time with nontroublesome dyskinesia, and (6) asleep time derived from the 3-day PD Diaries
  - Proportion of subjects with an improvement in "Good on" time of at least 1, 1.5, 2, 2.5, and 3 hours
  - Proportion of subjects with a reduction in "Off" time of at least 0.5, 1, 1.5, 2, 2.5, and 3 hours
  - Proportions of subjects who are "On" upon awakening and "Good on" upon awakening
  - Average time to "On" upon awakening
  - Change from baseline in the average number of motor fluctuations per day averaged over the PD Diary days. A motor fluctuation is defined as a change from "Off" to "On" state or from "On" to "Off" state.
  - MDS-UPDRS total score (sum of Parts I, II, III, and IV) and Parts I, II, and IV separately
  - MDS-UPDRS Part III and Parts II + III combined (from Visit 1)
  - MDS-UPDRS Part II Question 2.9
  - PDQ-39 total score and individual domain scores

- NMSS total score and individual domains
- PDSS-2 total score and individual domains
- PDSS-2 items 9, 10, 11, 12, and 13 combined
- PAS total score and individual domains
- PGI-S
- Proportion of subjects with either “severely ill” or “extremely severely ill” on the PGI-S
- CGI-S
- Proportion of subjects with either “severely ill” or “among the most extremely ill of subjects” on the CGI-S
- PGI-C scores
- CGI-C scores
- Proportion of subjects with either “much improved” or “very much improved” on the CGI-C

- **Safety:** electrocardiograms (ECGs), clinical laboratory tests, physical examinations, Columbia-Suicide Severity Rating Scale (C-SSRS), and supine and standing orthostatic vital signs; adverse events and concomitant medications evaluated throughout the course of the study.

Statistical methods: For the primary endpoint, assuming a difference of 1 hour between IPX203 and IR CD-LD in “Good on” time and a standard deviation of the treatment difference to be 3.0 hours, a sample size of 210 per arm will be needed to ensure at least 90% power at a 0.05 significance level. Assuming approximately an 18% prerandomization drop-out, approximately 510 subjects would need to be enrolled to randomize 420 subjects.

The primary efficacy endpoint of change from baseline in “Good on” time will be analyzed using a mixed model for repeated measures (MMRM) model. The model will include baseline (Visit 4) “Good on” time as a covariate, treatment, visit, and pooled center as fixed effects, and a treatment-by-visit interaction. The model will employ an unstructured within subject covariance matrix and a restricted maximum likelihood (ReML) estimation method. The degree-of-freedom of the denominator will be estimated using the Kenward-Roger method.

The key secondary endpoints (change from baseline in “Off” time, change from baseline in MDS-UPDRS Part III, and change from baseline in the sum of the MDS-UPDRS Parts II and III) will be analyzed using MMRM models similar to the primary analysis model. The proportion of subjects with either “much improved” or “very much improved” on the PGI-C will be analyzed using Cochran-Mantel-Haenszel test to compare the two treatment groups with pooled center as a stratification factor.

In order to control the type I error rate, the primary and key secondary endpoints will be tested in a single hierarchical order: (1) change from baseline in “Good on” time, (2) change from baseline in “Off” time, (3) proportion of subjects with either “much improved” or “very much improved” in PGI-C, (4) change from baseline in the MDS-UPDRS Part III, (5) change from baseline in the sum of MDS-UPDRS Parts II and III.

Quantitative safety data will be summarized using descriptive statistics and frequency distributions. Qualitative safety data will be summarized by frequencies and percentages. All summaries will be presented by treatment arms.

2. TABLE OF CONTENTS, LIST OF TABLES, AND LIST OF FIGURES

TABLE OF CONTENTS

1. SYNOPSIS 5
2. TABLE OF CONTENTS, LIST OF TABLES, AND LIST OF FIGURES 10
3. LIST OF ABBREVIATIONS AND DEFINITIONS OF TERMS 16
4. INTRODUCTION 19
5. TRIAL OBJECTIVES 23
6. INVESTIGATIONAL PLAN 24
6.1. Overall Study Design 24
6.2. Number of Subjects 25
6.3. Treatment Assignment 26
6.4. Dosing and Dose Determination Criteria 26
6.4.1. IR CD-LD Dose Adjustment Period 26
6.4.2. IPX203 Dose Conversion Period 26
6.4.3. Double-Blind Maintenance Period 28
6.5. Criteria for Study Termination 28
7. SELECTION AND WITHDRAWAL OF SUBJECTS 29
7.1. Subject Inclusion Criteria 29
7.2. Subject Exclusion Criteria 30
7.3. Subject Withdrawal Criteria 31
8. STUDY PROCEDURES 33
8.1. Screening Visit 37
8.2. Visit 1 – Start of IR CD-LD Dose Adjustment 38
8.2.1. Prior to Visit 1 38
8.2.2. At Visit 1 38
8.2.3. Post Visit 1 39
8.3. Visits 2 (Week 3) and Visit 3 (Week 5) – IPX203 Dose Conversion 39
8.3.1. Prior to Visit 2 39
8.3.2. Prior to Visit 3 39
8.3.3. At Visits 2 and 3 39

8.3.4. Post Visits 2 and 3 40
8.4. Visit 4 (Week 7) – Randomization 40
8.4.1. Prior to Visit 4 40
8.4.2. At Visit 4....................40
8.5. Visit 5 (Week 10) and Visit 6 (Week 15) 41
8.5.1. Prior to Visit 5 and 6....................41
8.5.2. At Visit 5 and 6....................41
8.6. Visit 7 (Week 20) – End of Study/Study Exit 42
8.6.1. Prior to Visit 7 42
8.6.2. At Visit 7....................42
8.7. Early Termination....................43
8.7.1. Subjects Who Terminate Prior to Randomization 43
8.7.2. Subjects Who Terminate Early after Randomization 44
8.8. Blood Volume....................44
9. TREATMENT OF SUBJECTS....................45
9.1. Description of Study Drug....................45
9.2. Concomitant Medications....................45
9.2.1. Permitted PD Medications....................45
9.2.2. Prohibited Medications and Procedures 45
9.2.3. Rescue Medications....................46
9.3. Treatment Compliance....................46
9.4. Randomization and Blinding 46
10. STUDY DRUG MATERIALS AND MANAGEMENT 47
10.1. Study Drug....................47
10.2. Study Drug Packaging and Labeling 47
10.3. Study Drug Storage....................47
10.4. Study Drug Administration....................48
10.5. Study Drug Dispensing and Accountability 48
10.6. Study Drug Handling and Disposal 48
11. ASSESSMENT OF EFFICACY 49
11.1. Parkinson’s Disease Diary....................49
11.2. Patient and Investigator Global Assessments 49

11.3. Movement Disorders Society Version of Unified Parkinson's Disease Rating Scale 49
11.4. Additional Assessments 50
12. ASSESSMENT OF SAFETY 51
12.1. Safety Parameters 51
12.2. Adverse Events 51
12.2.1. Definition of Adverse Event 51
12.2.2. Recording Adverse Events 51
12.2.3. Follow-up 52
12.2.4. Relationship to Study Drug 52
12.2.5. Assessment of Severity 52
12.3. Serious Adverse Events 52
12.3.1. Definition of Serious Adverse Event 52
12.3.2. Reporting Serious Adverse Events 53
12.4. Pregnancy 53
12.5. Other Safety Parameters and Related Information 54
13. STATISTICS 55
13.1. Study Design and Sample Size Estimation 55
13.2. Demographics/Baseline Comparability 55
13.3. Efficacy Endpoints 55
13.4. Analysis of Efficacy Data 57
13.4.1. Primary Efficacy Endpoint 57
13.4.2. Key Secondary Efficacy Endpoints 57
13.4.3. Additional Efficacy Endpoints 58
13.5. Center Pooling Algorithm 58
13.6. Sensitivity Analyses of the Primary Endpoint and Key Secondary Endpoints 59
13.6.1. Assessing Assumptions of the Mixed Model for Repeated Measures (MMRM) 59
13.6.2. Complete Case Analysis 59
13.6.3. Single LOCF/BLOCF Imputation 59
13.6.4. Pattern-Mixture Model 60
13.7. Subgroup Analyses 61
13.8. Multiplicity Adjustments 61

13.9. Analysis Populations 62
13.9.1. Safety Analysis Set 62
13.9.2. Intent-to-Treat Analysis Set 62
13.9.3. Modified Intent-to-Treat Analysis Set 62
13.9.4. Completers Analysis Set 62
13.10. Handling of Missing Data 62
13.10.1. Missing Data for PD Diaries 62
13.10.2. Missing Data for Global Assessments (PGI-C, CGI-C, PGI-S and CGI-S) 63
13.10.3. Missing Data for MDS-UPDRS 63
13.11. Analysis of Safety 64
14. ADMINISTRATIVE PROCEDURES 65
14.1. Guidelines for Good Clinical Practice 65
14.2. Institutional Review Board Approval 65
14.3. Informed Consent 65
14.4. Study Monitoring 65
14.5. Protocol Amendments 65
14.6. Termination of Study 66
14.7. Case Report Forms 66
14.8. Investigator’s Final Conduct Report 66
14.9. Records Retention 66
15. PUBLICATION POLICY 67
16. LIST OF REFERENCES 68
17. APPENDICES 70
APPENDIX A. PRESCRIBING INFORMATION FOR IR CD-LD 70
APPENDIX B. INSTRUCTIONS FOR DOSE CONVERSION TO IPX203 82
APPENDIX C. UNITED KINGDOM PARKINSON’S DISEASE SOCIETY BRAIN BANK DIAGNOSTIC CRITERIA FOR PARKINSON’S DISEASE 84
APPENDIX D. MONTREAL COGNITIVE ASSESSMENT (MOCA) 85
APPENDIX E. MOVEMENT DISORDERS SOCIETY VERSION OF UNIFIED PARKINSON’S DISEASE RATING SCALE (MDS-UPDRS) 91
APPENDIX F. PATIENT GLOBAL IMPRESSION OF CHANGE (PGI-C) 125
APPENDIX G. CLINICAL GLOBAL IMPRESSION OF CHANGE (CGI-C) 126
APPENDIX H. PATIENT GLOBAL IMPRESSION OF SEVERITY (PGI-S) 127

APPENDIX I. CLINICAL GLOBAL IMPRESSION OF SEVERITY (CGI-S)......................128
APPENDIX J. 39-ITEM PARKINSON'S DISEASE QUESTIONNAIRE (PDQ-39) 129
APPENDIX K. GASTROPARESIS CARDINAL SYMPTOM INDEX (GCSI).....................135
APPENDIX L. NON-MOTOR SYMPTOM ASSESSMENT SCALE FOR PARKINSON'S DISEASE (NMSS)..137
APPENDIX M. PARKINSON'S DISEASE SLEEP SCALE-2 (PDSS-2) 140
APPENDIX N. PARKINSON ANXIETY SCALE (PAS)...142
APPENDIX O. COLUMBIA-SUICIDE SEVERITY RATING SCALE (C-SSRS)................146
APPENDIX P. PARKINSON'S DISEASE DIARY 153
APPENDIX Q. EXCIPIENTS IN IPX203, IPX203 PLACEBO, IR CD-LD, AND IR PLACEBO 157
APPENDIX R. CLINICAL LABORATORY STUDIES 158

LIST OF TABLES

Table 1: Abbreviations and Specialist Terms 16
Table 2: LD Dosage in Study IPX203-B14-02 20
Table 3: Recommended Starting IPX203 LD Dosing Regimen Based on the Dosing Regimen of IR CD-LD at the End of the Dose Adjustment Period 27
Table 4: Events Schedule for Impax Study IPX203-B16-02 34
Table 5: Study Drugs for Study IPX203-B16-02 45

LIST OF FIGURES

Figure 1: Study Flow Chart 25

3. LIST OF ABBREVIATIONS AND DEFINITIONS OF TERMS

The following abbreviations and specialist terms are used in this study protocol.

Table 1: Abbreviations and Specialist Terms

Abbreviation or Specialist Term	Explanation
AADC	aromatic amino acid decarboxylase
ADL	activities of daily living
AE	adverse event
ANCOVA	analysis of covariance
ANOVA	analysis of variance
BLOCF	baseline observation carried forward
BMI	body mass index
CD	carbidopa
CGI-C	Clinical Global Impression of Change
CGI-S	Clinical Global Impression of Severity
CR	controlled release
CRF	case report form
C-SSRS	Columbia-Suicide Severity Rating Scale
ECG	electrocardiogram
ER	extended release
FDA	Food and Drug Administration
GCP	Good Clinical Practice
GCSI	Gastroparesis Cardinal Symptom Index
HIPAA	Health Insurance Portability and Accountability Act
ICF	informed consent form
ICH	International Conference on Harmonization
IEC	independent ethics committee

Abbreviation or Specialist Term	Explanation
IR	immediate release
IRB	institutional review board
IWRS	interactive web response system
LD	levodopa
LOCF	last observation carried forward
MAOI	monoamine oxidase inhibitors
MAR	missing at random
M-EDL	Motor Aspects of Experiences of Daily Living
MedDRA	Medical Dictionary for Regulatory Activities
MDS-UPDRS	Movement Disorders Society version of the Unified Parkinson's Disease Rating Scale
mITT	modified intent-to-treat
MMRM	mixed model for repeated measures
MNAR	missing not at random
MoCA	Montreal Cognitive Assessment
nM-EDL	Non-Motor Aspects of Experiences of Daily Living
NMSS	Non-Motor Symptom Assessment Scale
PAS	Parkinson Anxiety Scale
PD	Parkinson's disease
PDQ-39	39-item Parkinson's Disease Questionnaire
PDSS-2	Parkinson's Disease Sleep Scale-2
PGI-C	Patient Global Impression of Change
PGI-S	Patient Global Impression of Severity
PK	pharmacokinetic (adjective)<br>pharmacokinetics (singular noun)
PI	principal investigator

Abbreviation or Specialist Term	Explanation
PMM	pattern-mixture models
ReML	restricted maximum likelihood
SAE	serious adverse event
SAP	statistical analysis plan
SD	standard deviation
TEAE	treatment-emergent adverse event
US	United States

4. INTRODUCTION

Parkinson's disease (PD) is a progressive neurodegenerative disorder of the extrapyramidal nervous system. Levodopa (LD) used in combination with carbidopa (CD) is considered the gold standard for the symptomatic treatment of PD. LD is a dopamine precursor converted to dopamine by aromatic amino acid decarboxylase (AADC). Carbidopa is an AADC inhibitor that does not cross the blood-brain barrier. When used in combination with LD, CD increases the plasma half-life of LD from 50 minutes to 1.5 hours. Carbidopa inhibits the conversion of LD into dopamine in the periphery, thereby reducing the peripheral side-effects caused by dopamine and increasing the amount of LD available for transport into the brain. The administration of CD with LD reduces the dose of LD required to produce a dopaminergic response by about 75 percent (Sinemet prescribing information; Appendix A).

Due to its proven efficacy, LD is prescribed eventually to most subjects with PD. However, long-term use of LD is associated with certain complications, including "wearing-off" or "end-of-dose effect," where symptom control decreases causing the drug effects to wear off sooner. As the disease progresses further, motor complications, namely dyskinesias and motor "On/Off" fluctuations, develop in about 50% of the patients after 5 years of treatment (Fahn 1999). Such motor complications can be a significant source of disability and their management is a major unmet need in the treatment of PD.

Mechanisms underlying motor complications involving dyskinesias and "On/Off" fluctuations in PD are unclear. The pulsatile nature of standard orally administered LD is thought to contribute to the appearance of motor complications. Chronic intermittent pulsatile stimulation of the dopamine receptors that are under tonic control contributes to the development of dyskinesia in PD animal models as compared to animals treated with continuous infusion (Juncos et al 1989, Engber et al 1989, Blanchet et al 1995). In addition, unreliable absorption of LD potentially due to erratic gastric empting and variable in vivo dissolution of LD products is thought to contribute to the delay or inadequate response after oral dosing with standard CD-LD products (Melamed et al 1986, Kurlan et al 1988, Stocchi et al 1994). These findings suggest that motor complications in patients with PD may be less likely to develop with continuous dopaminergic stimulation.

Intraduodenal infusion of LD has been shown to significantly reduce motor complications and to reduce "Off" time. The findings of infusion studies in PD patients indicate that the maintenance of stable plasma LD concentrations and the avoidance of low trough levels are effective in reducing "Off" hours, increasing "On" hours without disabling dyskinesia, and reducing the severity of dyskinesia versus standard oral LD formulations (Mizuno 2007, Nilsson et al 2001, Nyholm et al 2005, Stocchi et al 2005). These findings provide a strong rationale for the development of an extended-release (ER) oral dosage form that delivers a constant LD plasma concentration in order to optimize relief of PD symptoms, and to minimize "Off" time and dyskinesia.

IPX203 is an investigational product containing CD-LD that is being developed by Impax Laboratories, LLC (Impax). The primary objective of the IPX203 program is to develop an extended-release product that can attain therapeutic LD plasma concentrations rapidly and

maintain constant LD plasma concentrations for a longer duration than currently approved products with minimal peak-to-trough fluctuations. IPX203 is designed to be dosed approximately every 8 hours.

Impax characterized the PK and pharmacodynamics of IPX203 in Study IPX203-B14-02, a single dose trial in subjects with advanced PD versus IR CD-LD and Rytary (carbidopa and levodopa) extended-release capsules. Twenty-six (26) subjects were randomized with 25 subjects completing all 3 treatments. One subject discontinued study early due to subject withdrawal. The doses of IPX203 and Rytary were determined on the basis of each subject's prestudy baseline morning dose of IR CD-LD (Table 2).

Table 2: LD Dosage in Study IPX203-B14-02

Prestudy Baseline Morning IR LD (mg)	IR LD (mg)	Rytary LD (mg)	IPX203 LD (mg)
100	100	340	360
150	150	485	540
200	200	630	720
250	250	780	810

Administration of IPX203 yielded an initial increase in LD plasma concentrations that was similar to IR CD-LD but maintained LD concentrations for a longer duration than either IR CD-LD or Rytary. The bioavailability of LD (based on AUC_{∞}) from IPX203 was 88% relative to IR CD-LD and about 11% more than Rytary. Plasma exposure to LD (C_{max} and AUC_{∞}) following IPX203 increased in an approximately dose-proportional manner. Pharmacodynamic effects as measured by change from baseline scores on MDS-UPDRS Part III were consistent with the PK profiles of LD. Following IPX203 treatment, decrements in the Part III total score (reflecting improvements in motor symptoms) lasted for a longer duration than either IR CD-LD or Rytary. IPX203 provided a longer duration of effect compared with IR CD-LD and Rytary, including "Off" time and "Good on" time based on the Assessment of Subject's Motor State and on a range of improvement thresholds of the MDS-UPDRS Part III. The results were consistent with the prolonged LD plasma concentration profile with IPX203 compared to IR CD-LD or Rytary and support a dosing interval of approximately 8 hours.

Of the 26 subjects who received at least one of the 3 treatments, 9 (34.6%) reported at least one treatment-emergent adverse event (AE). None of these subjects reported a serious AE (SAE) nor did any subjects prematurely discontinue the study because of an AE. Adverse events were reported by more subjects during IR (28.0%) and IPX203 (19.2%) than during Rytary (8.0%) treatment. None of the reported AEs were classified as "severe." Adverse events reported by 2 or more subjects include: Dizziness (3 subjects), nausea (2 subjects), and hypertension (2 subjects). The numbers of subjects reporting these AEs during any single treatment period were small (0 to 2 subjects). Two subjects reported dizziness during IR CD-LD treatment and one subject each during IPX203 and Rytary treatments. Hypertension was reported by a total of 2 subjects, both reporting this AE during IPX203 and IR CD-LD treatments and 1 subject during Rytary treatment. Two subjects reported nausea only during the IR CD-LD treatment period. Of the 9 subjects reporting AEs, 6/26 (23.1%) of subjects reported AEs that were assessed as related to treatment, including all of the reports of dizziness, nausea, and dyskinesia (1 subject).

Study IPX203-B16-01 is a randomized, open-label, rater-blinded, multicenter, 2-treatment, 2-period, multiple-dose crossover study that has completed dosing. Twenty-eight (N=28) advanced PD subjects were randomized to 1 of 2 dosing sequences, with each treatment period lasting 15 days and separated by a 1-week wash-out period where subjects return to their usual stable pre-study CD-LD regimen. The objectives of this study are to compare the PK, pharmacodynamics, efficacy, and safety of IPX203 with IR CD-LD after single and multiple dosing. Subjects were permitted to take allowed non-CD-LD based PD medications throughout the study if dosing regimens had been stable for at least 4 weeks. Subjects were instructed to take their last dose of CD-LD no later than 10:00 PM on the evening prior to Day 1 of each treatment period and to withhold dosing for at least 5 hours before arriving at the site on Day 15 of each treatment period. On Day 1 of the IR CD-LD treatment period, subjects were started with a single dose of their usual prestudy first morning IR CD-LD dose. On Day 1 of the IPX203 treatment period, subjects were started with a single dose of IPX203 based on their usual prestudy first morning IR CD-LD dose using a LD conversion of 100 mg IR LD to 360 mg of IPX203 LD. During the IR CD-LD treatment period, the initial dosing regimen of IR CD-LD was the same as the subject's stable prestudy regimen. During the IPX203 treatment period, the IPX203 regimen for subsequent doses for the day was determined by identifying the most frequent prestudy IR LD dose in milligrams that the subject received in the afternoon and evening and administering IPX203 using a LD conversion of 100 mg IR LD to 270 mg of IPX203 LD. The protocol recommended that IPX203 be dosed approximately every 7 to 8 hours. During Days 1 through 9 of both treatment periods, investigators had the opportunity to adjust each subject's study medication regimen if necessary to optimize efficacy and safety. Pharmacokinetics and pharmacodynamics (MDS-UPDRS Part III and Assessments of Subject's Motor State) were periodically evaluated on Day 1 and Day 15 of each treatment period by qualified clinical staff who were blinded to dosing.

Data from this multiple-dose study confirmed the PK and pharmacodynamic results observed in the single dose study with IPX203:

- PK data from 27 subjects indicates IPX203 shows a rapid increase in LD concentrations followed by extended-release characteristics. Following IPX203; initial increases in LD concentrations were comparable to that from IR CD-LD. Bioavailability of LD following IPX203 was ~89% relative to IR CD-LD. LD plasma concentrations were sustained longer after IPX203 treatment than after IR CD-LD and support dosing every 8 hours. No accumulation of LD was evident at steady-state following IPX203 or IR CD-LD. Plasma LD concentrations following IPX203 were characterized by lower peak-to-trough fluctuation. No time-variant or time-dependent changes were noted in PK of CD or LD following IPX203.
- IPX203 demonstrated an onset of effect that was comparable to IR CD-LD in MDS-UPDRS Part III scores. IPX203 prolonged the duration over which MDS-UPDRS Part III scores were improved by prespecified threshold changes from baseline (≥4, ≥7, and ≥13 units).
- IPX203 provides a significant decrease in "Off" time and a significant increase in "Good on" time compared to IR CD-LD treatment on Day 1 and Day 15 when assessed by the Investigator's Assessment of Subject's Motor State. Subjects treated

with IPX203 did not experience a significant increase in “On” time with troublesome dyskinesia compared to IR CD-LD.

- Subjects achieved significant improvements in “Off” time, “Good on” time, and frequency of motor state fluctuations based on the 3-day PD Diaries.
- Twenty-eight subjects were enrolled in the multiple dose study and 27 subjects completed both treatments. Safety results were as follows:
  - One subject discontinued during the IPX203 treatment period due to an AE (orthostatic hypertension) that was considered possibly related to treatment.
  - A total of 39.3% (11/28) of treated subjects reported at least one treatment emergent AE, including 35.7% (10/28) during IPX203 treatment and 7.4% (2/27) during IR CD-LD treatment. Eight subjects reported AEs that were related to treatment (8 subjects during IPX203 treatment and 1 during IR CD-LD treatment).
  - Two subjects experienced serious adverse events (SAEs). One subject reported increased hypertension of mild severity during IPX203 treatment that was considered unrelated to treatment and resolved. A second subject reported moderate to severe dehydration, diarrhea, and atrial fibrillation during the washout period that were considered unrelated to treatment and resolved.
  - AEs reported in 2 or more subjects included nausea (2), dizziness (2), and dyskinesia (5), all of mild or moderate severity, and all during the IPX203 treatment.

The current protocol, Study IPX203-B16-02, is a multicenter, randomized, double-blind, double-dummy, active-controlled, parallel-group, Phase 3 study. It is designed to compare the efficacy, safety and tolerability of IPX203 with IR CD-LD following multiple doses over 13 weeks. The following IPX203 dosing guidelines will be utilized in the present study (IPX203-B16-02):

- The initial regimen of IPX203 is based on the most frequent dose of the subject’s dosing regimen of IR CD-LD at the end of dose adjustment period (Visit 2);
- A 25-100 mg dose of IR CD-LD will be converted to a 70-280 mg CD-LD dose of IPX203;
- IPX203 will be administered approximately every 8 hours for most subjects;
- Investigators may adjust the IPX203 regimen during the dose conversion period to optimize the therapeutic effect (minimize “Off” time without causing troublesome dyskinesia or other dopaminergic side effects).

The proposed dose conversion scheme for this study has been developed based on a similar dose conversion from IR CD-LD to IPX203 that was studied in the completed Phase 2a study (IPX203-B14-02, n=25) and the Phase 2b study (IPX203-B16-01, n=28), both conducted in subjects with advanced PD using similar entry criteria to the present study. The doses of IPX203 are expected to be comparable to other ER CD-LD products, such as Rytary and Duopa.

5. TRIAL OBJECTIVES

To evaluate the safety and efficacy of IPX203 in comparison to IR CD-LD in the treatment of CD-LD experienced subjects with Parkinson's disease (PD) who have motor fluctuations.

6. INVESTIGATIONAL PLAN

6.1. Overall Study Design

This is a multicenter, randomized, double-blind, double-dummy, active-controlled, parallel-group study. Subjects will continue to take permitted non-CD-LD-based PD medications throughout the study if documented in their prestudy regimen and if dosing regimens have been stable for at least 4 weeks prior to Visit 1. A "stable dosing regimen" means no change in dose or in dosing frequency. Within 4 weeks following the Screening visit, eligible subjects will complete their PD Diaries on each of the 3 consecutive days immediately prior to Visit 1.

Following Visit 1, qualified subjects will enter a 3-week, open-label IR CD-LD treatment period allowing for dose adjustment. During the IR CD-LD dose adjustment period, the initial dosing regimen of IR CD-LD will be the same as the subject's stable prestudy regimen unless the subject is taking a single daily bedtime dose of CR CD-LD, in which case, the CR CD-LD dose will be discontinued and substituted with a 1:1 milligram-equivalent dose of IR CD-LD. A "bedtime dose" is defined as the last daytime dose of CD-LD taken within 1 hour of onset of the subject's normal nighttime sleep period. **The dosing regimen of IR CD-LD may be adjusted during the dose adjustment period** to minimize "Off" time without causing troublesome dyskinesia. The doses and regimens of the subject's other non-CD-LD Parkinson's disease medications (dopamine agonists, MAO-B inhibitors, amantadine, anticholinergics) should remain stable throughout this study. Any adjustments to the IR CD-LD dosing regimen will be done in consultation with the Investigator or qualified site personnel and will be recorded. The subject must be on a stable dosing regimen (no change in dose or in dosing frequency) of IR CD-LD for at least 5 days prior to returning for Visit 2. Rescue with additional or modified doses of concomitant PD medications or with use of CD-LD products other than the dispensed study medications is not permitted and will trigger discontinuation from the study. Subjects will complete their 3-day PD Diaries on each of the 3 consecutive days immediately prior to Visit 1.

Following completion of the IR CD-LD dose adjustment period, subjects will begin a 4-week open-label period for conversion to IPX203. The initial dosing regimen of IPX203 during the conversion period will be based on the subject's dosing regimen of IR CD-LD at the end of the dose adjustment period (Visit 2) selecting the most frequent dose according to Table 3. A 25-100 mg dose of IR CD-LD converts to a 70-280 mg CD-LD dose of IPX203 but with a longer duration of effect. It is recommended that IPX203 should be dosed approximately every 8 hours with the exception that subjects who are currently receiving a total daily dose of less than 125-500 mg IR CD-LD at the end of the dose adjustment period will be initially administered IPX203 every 12 hours. The dosing interval may be reduced to approximately every 8 hours if the subject does not achieve an acceptable duration of effect. **The dosing regimen of IPX203 may be adjusted during the dose conversion period** to achieve the optimal balance of efficacy and tolerability (minimize "Off" time without causing troublesome dyskinesia or other dopaminergic side effects). Any adjustments to the IPX203 dosing regimen will be recorded. Rescue with additional or modified doses of concomitant PD medications or with use of CD-LD products other than the dispensed study medications is not permitted and will trigger discontinuation from the study. Subjects will return to the clinic in two weeks for Visit 3 followed by Visit 4 two weeks later. The subject must be on a stable dosing regimen of IPX203

(no change in dose or in dosing frequency) for at least 5 days prior to returning for Visit 4. Subjects will also be instructed to complete their 3-day PD Diaries on each of the 3 consecutive days immediately prior to Visit 4.

The study staff will call the subjects frequently (approximately every 1 to 3 days) during the IR CD-LD dose adjustment and IPX203 dose conversion periods. The calls are to ensure timely and appropriate dosing adjustments and to ensure that the subject is able to follow and adhere to the dosing instructions. The contacts may be less frequent after initial dose adjustments have been made. Any changes in the dosing regimen will be in consultation with the Investigator or qualified site personnel and will be documented.

Subjects who successfully complete the IPX203 dose conversion period will be randomized, stratified by center, in a 1:1 ratio at Visit 4 into one of two parallel treatment arms of IPX203 (with matching IR CD-LD placebo) or IR CD-LD (with matching IPX203 placebo). The subjects will undergo 13 weeks of double-blind, double-dummy maintenance therapy with the stable dosing regimen established at the end of Week 3 (Visit 2) for IR CD-LD and at the end of Week 7 (Visit 4) for IPX203. Subjects will return to the clinic for 3 visits (Visits 5, 6, and 7) and will be instructed to complete their 3-day PD Diaries on 3 consecutive days immediately prior to each of the 3 visits. Rescue with additional or modified doses of concomitant PD medications or use of CD-LD products other than the dispensed study medication is not permitted and will trigger discontinuation from the study.

Figure 1: Study Flow Chart

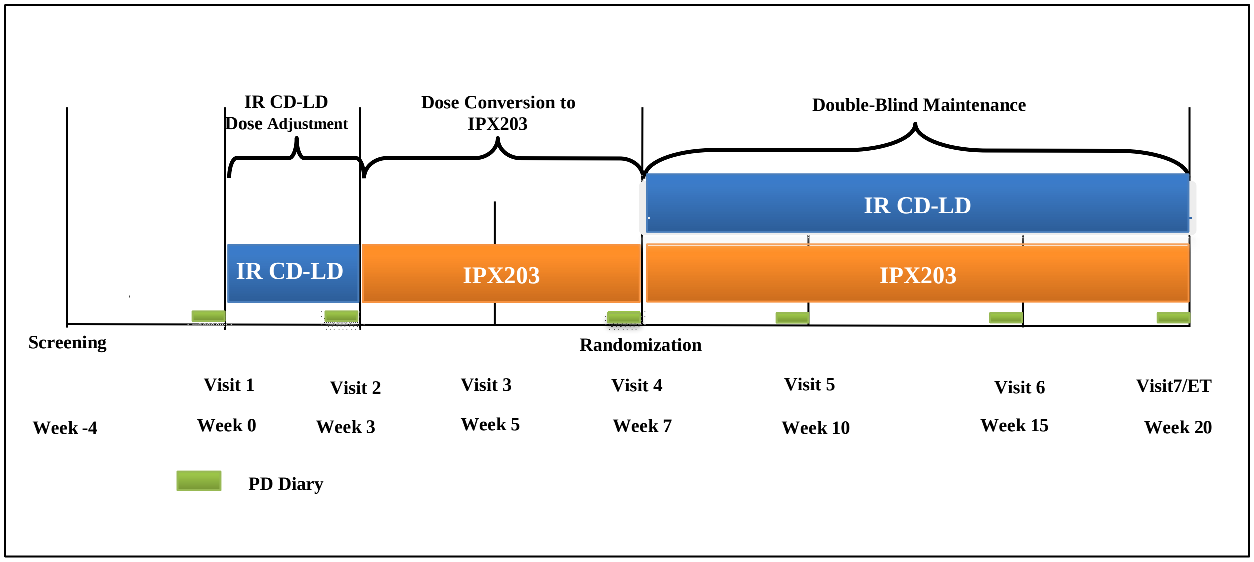

Abbreviations: IR=immediate-release, CD=carbidopa, LD=levodopa, ET=early termination

6.2. Number of Subjects

Assuming approximately an 18% prerandomization drop-out, approximately 510 subjects will be enrolled to randomize 420 subjects.

6.3. Treatment Assignment

Investigational product: IPX203 (carbidopa-levodopa) Extended-Release Capsules containing 35-140 mg of CD-LD, for oral administration.

Reference therapy: Immediate-Release carbidopa-levodopa (IR CD-LD) tablets containing 25-100 mg of CD-LD, for oral administration.

Subjects will be randomly assigned to one of two parallel treatment arms to receive either IPX203 (with matching IR CD-LD placebo) or IR CD-LD (with matching IPX203 placebo) during the double-blind maintenance therapy portion of the study.

6.4. Dosing and Dose Determination Criteria

IR CD-LD will be supplied as tablets containing 25-100 mg of CD-LD. IR CD-LD tablets may be split to achieve the required dose.

IPX203 will be supplied as capsules containing 35-140 mg of CD-LD. The suggested doses and regimen of IPX203 are intended to provide an onset of effect comparable to the subject's prestudy IR LD regimen and to extend the duration of effect. A 25-100 mg dose of IR CD-LD converts to a 70-280 mg CD-LD dose of IPX203.

6.4.1. IR CD-LD Dose Adjustment Period

During the 3-week IR CD-LD dose adjustment period, the initial dosing regimen of IR CD-LD will be the same as the subject's stable prestudy regimen unless the subject is taking a single daily bedtime dose of CR CD-LD, in which case, the CR CD-LD dose will be discontinued and substituted with a 1:1 milligram-equivalent dose of IR CD-LD. A "bedtime dose" is defined as the last daytime dose of CD-LD taken within 1 hour of onset of the subject's normal nighttime sleep period. Subjects who were receiving IR CD-LD as a 1:10 CD-LD formulation will be started on IR CD-LD with a 1:4 ratio at the same frequency and LD dose. **The dosing regimen of IR CD-LD may be adjusted during the dose adjustment period** to minimize "Off" time without causing troublesome dyskinesia. The doses and regimens of the subject's other non-CD-LD Parkinson's disease medications (dopamine agonists, MAO-B inhibitors, amantadine, anticholinergics) should remain stable throughout this study. Any adjustments to the IR CD-LD dosing regimen will be done in consultation with the Investigator or qualified site personnel and will be recorded. The subject must be on a stable dosing regimen (no change in dose or in dosing frequency) of IR CD-LD for at least 5 days prior to returning for Visit 2.

6.4.2. IPX203 Dose Conversion Period

During the 4-week IPX203 dose conversion period, the suggested initial dosing regimen of IPX203 will be based on the most frequent dose of the subject's dosing regimen of IR CD-LD at the end of the dose adjustment period (Visit 2). A 25-100 mg dose of IR CD-LD converts to a 70-280 mg CD-LD dose of IPX203, and a half tablet (12.5-50 mg dose of IR CD-LD) converts to a 35-140 mg CD-LD dose of IPX203. To facilitate conversion of subjects from IR CD-LD to IPX203, Table 3 presents recommended starting dose regimens. It is recommended that IPX203 should be dosed approximately every 8 hours with the exception that subjects who are currently receiving a total daily dose of less than 125-500 mg IR CD-LD at the end of the dose adjustment

period will be initially administered IPX203 every 12 hours. The dosing interval may be reduced to approximately every 8 hours if the subject does not achieve an acceptable duration of effect. **The dosing regimen of IPX203 may be adjusted during the dose conversion period** to achieve the optimal balance of efficacy and tolerability (minimize "Off" time without causing troublesome dyskinesia or other dopaminergic side effects). The maximum recommended daily dose of IPX203 is 600-2400 mg CD-LD. The doses and regimens of the subject's other non-CD-LD Parkinson's disease medications (dopamine agonists, MAO-B inhibitors, amantadine, anticholinergics) should remain stable throughout this study. The subject must be on a stable dosing regimen of IPX203 (no change in dose or in dosing frequency) for at least 5 days prior to returning for Visit 4.

Table 3: Recommended Starting IPX203 LD Dosing Regimen Based on the Dosing Regimen of IR CD-LD at the End of the Dose Adjustment Period

Most Frequent IR CD-LD Unit Dose (mg)	Recommended Starting IPX203 Daily Dosing Regimen CD-LD (mg) Every 8 Hours
25-100[a]	70-280 mg (2 × 35-140 mg)
>25-100 – 37.5-150	105-420 mg (3 × 35-140 mg)
>37.5-150 – 50-200	140-560 mg (4 × 35-140 mg)
>50-200	175-700 mg (5 × 35-140 mg)

[a] Subjects who are on a total daily dose of less than 125-500 mg CD-LD from IR CD-LD should be advised to initially take IPX203 every 12 hours. The dosing interval may be reduced to approximately every 8 hours if the subject does not achieve an acceptable duration of effect.

During the dose conversion to IPX203, the Investigator or site staff are advised to be in frequent contact (every 1 to 3 days) with the subject especially during the initial dose conversion to assess the need for dosage adjustment with the goal of minimizing "Off" time without causing troublesome dyskinesia. Any changes to the dosing regimen should only be made by the Investigator or qualified site personnel. If the subject experiences troublesome dyskinesias during initial dose conversion, consider reducing the dose by one capsule (35-140 mg IPX203 CD-LD) before increasing the dosing interval. If turning "On" is slow following the first morning dose, consider taking the morning dose in the fasted state and/or increasing the dose by one capsule (35-140 mg IPX203 CD-LD). If turning "On" is slow later in the day or to reduce "end-of-dose" "Off" time, consider increasing the dose by one capsule (35-140 mg IPX203 CD-LD).

When two or more IR CD-LD doses correspond to the most frequent IR CD-LD dose, the suggested IPX203 conversion should be based on the higher of the IR CD-LD doses.

A summary of the instructions for dose conversion to IPX203 is provided in Appendix B.

6.4.3. Double-Blind Maintenance Period

During the 13-week double-blind double-dummy maintenance period, subjects receive either IPX203 (with matching IR CD-LD placebo) or IR CD-LD (with matching IPX203 placebo) with the stable dosing regimen established at the end of Week 3 (Visit 2) for IR CD-LD and at the end of Week 7 (Visit 4) for IPX203.

6.5. Criteria for Study Termination

The Sponsor has the right to terminate this study and remove all study material from the study site at any time for medical or administrative reasons. The Sponsor will endeavor to give adequate notice to allow safe withdrawal of subjects from the study.

7. SELECTION AND WITHDRAWAL OF SUBJECTS

Subjects must meet all of the following inclusion criteria to qualify for enrollment. Subjects who have any of the following exclusion criteria will not be enrolled in the study.

7.1. Subject Inclusion Criteria

1. Male or female subjects diagnosed at age ≥ 40 years with PD, consistent with the United Kingdom Parkinson's Disease Society Brain Bank Diagnostic Criteria (Appendix C) and who are being treated with stable regimens of CD-LD but experiencing motor fluctuations.
2. Able to provide written informed consent prior to the conduct of any study-specific procedures.
3. Female subjects of childbearing potential must have a negative urine pregnancy test at Screening Visit.
4. Negative urine screen for drugs of abuse and negative alcohol breath test at Screening.
5. Hoehn and Yahr Stages 1, 2, 3, or 4 in the "On" state (part of Movement Disorders Society version of the Unified Parkinson's Disease Rating Scale [MDS-UPDRS] Part III; Appendix E).
6. Agrees to use a medically acceptable method of contraception throughout the study and for 6 weeks after completing the study. Medically acceptable methods of contraception that may be used by the subject and/or partner include but are not limited to: abstinence, oral contraception, NuvaRing or transdermal systems, diaphragm with vaginal spermicide, intrauterine device, condom and partner using vaginal spermicide, surgical sterilization (6 months), progestin implant or injection, or postmenopausal female (no menstrual period for > 2 years) or vasectomy (> 6 months).
7. Montreal Cognitive Assessment (MoCA) score ≥ 24 at Screening Visit in "On" state (Appendix D).
8. By history, for the 4 weeks prior to Screening, the subject experiences daily "wearing-off" episodes with periods of bradykinesia in combination with at least one of rest tremor or rigidity, experiences an "Off" state upon awakening on most mornings, and reports an average of at least 2.5 cumulative hours per day of "Off" time during the waking hours.
9. Able to differentiate "On" state from "Off" state as determined by at least 75% concordance with a trained rater in "On/Off" ratings for 8 ratings over the 4-hour training period. The concordance must include at least 1 "On" and 1 "Off" rating and must be achieved within two 4-hour training sessions.
10. At Visit 1, review of the 3-day PD Diaries confirms the following: that the subject is able to properly complete the diaries with valid entries; and that the subject has an average of at least 2.5 hours per day of "Off" time during the waking hours over the 3 days with at least 1.5 hours of cumulative "Off" time on each day. Inability to properly complete the

diaries is indicated when more than 1 day of a diary is not returned or when more than 2 hours (4 half-hour periods) of one 24-hour diary day are missing (Appendix P).

11. Responsive to CD-LD therapy and currently being treated on a stable regimen with CD-LD for at least 4 weeks prior to Visit 1 and:
    a. Requires at least 100 mg of LD from IR CD-LD for the first morning dose
    b. Requires a total daily dose of at least 400 mg of LD and takes a maximum total daily dose of 2400 mg LD, from IR CD-LD alone or IR CD-LD in combination with a single daily bedtime dose of CR CD-LD
    c. Has a dosing frequency of 4 to 9 times daily of CD-LD
    d. By history, typically experiences an “On” response with the first dose of IR CD-LD of the day, but the efficacy of this dose typically lasts less than 4 hours.
12. At Screening, the subject has predictable “Off” periods defined by a score of 1 or 2 on Item #4.5 (Complexity of Motor Fluctuations) of the MDS-UPDRS Part IV B (Motor Fluctuations) (Appendix E).
13. At Screening, the MDS-UPDRS Part III total score in the “Off” state is at least 20 units.
14. Able and willing to comply with the protocol, including completion of diaries and availability for all study visits.

7.2. Subject Exclusion Criteria

1. Used any doses of controlled-release (CR) CD-LD apart from a single daily bedtime dose within 4 weeks prior to Visit 1.
2. Used any doses of Rytary for the past 4 weeks prior to Visit 1 or considered IPX066 or Rytary failures for reasons of efficacy or safety.
3. Received any investigational medications within 30 days or 5 times the half-life, whichever is longer, prior to Visit 1.
4. Female subjects who are currently breastfeeding or lactating.
5. Had prior neurosurgical treatment for PD or if such procedure is planned or anticipated during the study period.
6. Allergic to any excipient in the study drugs (See Appendix Q).
7. History of medical conditions or of a prior surgical procedure that would interfere with LD absorption, such as gastrectomy, proximal small-bowel resection, or bariatric surgery.
8. History of upper gastrointestinal hemorrhage in patients with peptic ulcer disease within the past 5 years.
9. History of glaucoma with intraocular pressures that are elevated despite appropriate medical management.
10. History of seizure or epilepsy and experienced at least 1 seizure during the past 12 months or has not been compliant with medically recommended therapy or visits.
11. History of myocardial infarction with residual atrial, nodal, or ventricular arrhythmias that are not controlled with medical and/or surgical interventions. A recent

(≤ 12 months) history of myocardial infarction with secondary arrhythmias is exclusionary regardless of the therapeutic control.

12. History of neuroleptic malignant syndrome or of nontraumatic rhabdomyolysis.
13. Liver enzyme values ≥ 2.5 times the upper limit of normal; or history of severe hepatic impairment.
14. Serum creatinine level ≥ 1.75 times the upper limit of normal; or requires dialysis at the time of Screening.
15. Subject with a history of malignant melanoma or with a suspicious undiagnosed skin lesion which in the opinion of the investigator could be melanoma.
16. History of drug or alcohol abuse within the 12 months prior to Screening.
17. Received within 4 weeks of Visit 1 or planning to take during participation in the clinical study:
    a. any doses of a controlled-release (CR) LD apart from a single daily bedtime dose, any doses of Rytary, additional CD (eg, Lodosyn) or benserazide (eg, Serazide), or catechol-O-methyl transferase inhibitors (entacapone or tolcapone) or medications containing these inhibitors (Stalevo),
    b. nonselective monoamine oxidase inhibitors (MAOI), apomorphine, or antidopaminergic agents, including antiemetics.
18. Treatment with any dopamine antagonist for the purposes of treating psychosis or bipolar disorder within the last 2 years or any history of psychosis within the past 10 years regardless of treatment. A brief, self-limited episode of psychosis precipitated by a medical intervention with return to normal mentation is not exclusionary. Mild PD-associated illusions are not exclusionary provided that they do not occur more than twice per week and the subject does not lose insight.
19. Employees or family members of the investigator, study site, or sponsor.
20. Subjects who have previously participated in an IPX203 study.
21. Subjects who, in the opinion of the clinical investigator, should not participate in the study.
22. Based on clinical assessment, subject does not adequately comprehend the terminology needed to complete the PD diary.

7.3. Subject Withdrawal Criteria

Site personnel should make every effort to conduct all protocol-specific procedures to complete the study. A subject may be discontinued from the study due to the following reasons:

1. Withdrawal by subject
2. Adverse event (AE)
3. Lack of efficacy
4. Study terminated by Sponsor

5. Protocol deviation
6. Noncompliance with study drug
7. Lost to follow-up
8. Death
9. Other

Subjects who withdraw early from the study will not be replaced. The reason or reasons for discontinuation will be specified and documented. Empty medication bottles and any unused study drug upon discontinuation will be collected. Study medication dispensed to a discontinued subject may not be redispensed to a different subject.

8. STUDY PROCEDURES

The procedures to be performed at each study visit are described below and summarized in Table 4.

Table 4: Events Schedule for Impax Study IPX203-B16-02

		3 Weeks of IR CD-LD Dose Adjustment	4 Weeks of IPX203 Dose Conversion			13 Weeks of Double-Blind Therapy		
**Assessment**	**Screening**	**Visit 1**	**Visit 2**	**Visit 3**	**Visit 4 Randomization**	**Visit 5**	**Visit 6**	**Visit 7 / Study Exit/Early Termination[b]**
Study Week[a]	**–4**	**0**	**3**	**5**	**7**	**10**	**15**	**20**
ICF & HIPAA Authorization[c]	X							
Contact IWRS	X	X	X	X	X	X	X	X
Randomization					X			
Inclusion/Exclusion	X	X						
Medical History	X							
Physical Examination	X							X
Vital Signs[d]	X	X	X	X	X	X	X	X
Height and Weight	X					X[e]		X[e]
C-SSRS[f]	X	X	X	X	X	X	X	X
Clinical Laboratory Tests[g]	X					X		X
Urine Pregnancy Test	X							
Urine Screen for Drug Abuse	X							
Alcohol Breath Test	X							
ECG	X					X		X
MoCA[h]	X							
MDS-UPDRS Parts I-IV	X[i]	X	X		X	X	X	X
PGI-C[j]						X	X	X
CGI-C[k]						X	X	X
PGI-S[l]		X			X			X

Assessment	Screening	3 Weeks of IR CD-LD Dose Adjustment	4 Weeks of IPX203 Dose Conversion			13 Weeks of Double-Blind Therapy		
		Visit 1	Visit 2	Visit 3	Visit 4 Randomization	Visit 5	Visit 6	Visit 7 / Study Exit/Early Termination[b]
Study Week[a]	**–4**	**0**	**3**	**5**	**7**	**10**	**15**	**20**
CGI-S[m]		X			X			X
PDQ-39[n]		X			X		X	X
GCSI[o]		X						X
NMSS[p]		X			X		X	X
PDSS-2[q]		X			X		X	X
PAS[r]		X			X		X	X
PD Diary Training; Perform Concordance Testing at Screening Only[s]	X	X	X	X	X	X	X	
Dispense PD Diaries[t]	X	X		X	X	X	X	
Review PD Diaries[u]		X	X		X	X	X	X
Reminder phone calls[v,w]	X[v]	X[w]	X[w]	X[w]	X[w]	X	X	X
Dispense study medication		X	X	X	X	X	X	
Collect empty medication bottles and any unused study drug/Perform study drug accountability			X	X	X	X	X	X
Adverse Events	X	X	X	X	X	X	X	X
Concomitant Medications	X	X	X	X	X	X	X	X

CGI-C = Clinical Global Impression of Change, CGI-S = Clinical Global Impression of Severity, C-SSRS = Columbia-Suicide Severity Rating Scale, ECG = electrocardiogram, GCSI = Gastroparesis Cardinal Symptom Index, HIPAA = Health Insurance Portability and Accountability Act, ICF = informed consent form, IWRS = interactive web response system, MDS-UPDRS = MDS version of Unified Parkinson's Disease Rating Scale, MoCA = Montreal Cognitive Assessment, PAS = Parkinson Anxiety Scale, PD = Parkinson's disease, NMSS = Non-Motor Symptom assessment scale for PD, PDQ-39 = 39-Item Parkinson's Disease Questionnaire, PDSS-2 = Parkinson's Disease Sleep Scale-2, PGI-C = Patient Global Impression of Change, PGI-S = Patient Global Impressions of Severity.

[a] The interval between Screening and Visit 1 (Day 1) should not exceed 4 weeks. Study visits should occur within ± 3 days of their specified timing.

[b] Study Exit procedures to be conducted at the end of Visit 7 or during an early termination visit.
[c] Subjects enrolled at sites in the United States (US) must sign HIPAA authorization prior to the conduct of any study-specific procedures.
[d] Record vital signs (blood pressure, heart rate, respiratory rate, and temperature [Screening and Study Exit only]) after subject has been resting supine for at least 5 minutes, then record orthostatic blood pressure and heart rate after subject has been standing for approximately 2 minutes. At Visits 1 and 4, orthostatic vital signs (blood pressure and heart rate) will be performed in triplicate, each set separated by at least 15 minutes from the previous set.
[e] Weight only.
[f] C-SSRS: Columbia Suicide Severity Rating Scale. See Appendix O.
[g] See Appendix R.
[h] Montreal Cognitive Assessment in the "On" state: see Appendix D.
[i] At Screening MDS-UPDRS Parts I through IV will be done in both the "On" and "Off" state (see Appendix E).
[j] See Appendix F.
[k] See Appendix G.
[l] See Appendix H.
[m] See Appendix I.
[n] See Appendix J.
[o] See Appendix K.
[p] See Appendix L.
[q] See Appendix M.
[r] See Appendix N.
[s] Train at Screening and then as needed at subsequent visits. Perform concordance testing at Screening.
[t] Dispense PD Diaries at Screening and Visits 1, 3, 4, 5, and 6. Call subjects 4 days prior to Visits 1, 2 and 4-7 to remind them to complete PD Diaries. Subjects record diary information for 3 consecutive days immediately prior to each of the visits (Days -3, -2, and -1). Call subjects the day prior to each visit to remind them to bring the PD Diaries, empty medication bottles, and any unused study drug to the office.
[u] Review PD Diaries at Visits 1, 2, and 4-7.
[v] Post-Screening reminder phone call: Notify individuals who successfully complete screening procedures following review of all study entry criteria and clinical laboratory results that they may continue in the study. The interval between Screening and Visit 1 should not exceed 4 weeks.
[w] Reminder phone calls for Visits 1 through 4: In addition to the calls discussed above, make regular phone calls (approximately every 1 to 3 days) to subjects throughout the IR CD-LD dose-adjustment and IPX203 dose-conversion periods to evaluate each subject's adjustment to the study medication regimen.

8.1. Screening Visit

After the subject has signed the informed consent (and HIPAA authorization for US subjects only), complete the following procedures and assessments:

- Obtain an identification number from the Interactive Web Response System (IWRS). The IWRS will assign a 6-digit ID number to each subject, consisting of a 3-digit number representing the investigative site and a 3-digit sequential subject number.
- Review and record study entry criteria (Section 7).
- Perform urine pregnancy test for females of childbearing potential.
- Perform urine screen for drugs of abuse.
- Perform alcohol breath test.
- Complete medical history.
- Perform physical examination, including height and weight.
- Assess vital signs after subject is supine for at least 5 minutes (blood pressure, heart rate, temperature and respiratory rate) and then assess orthostatic blood pressure and heart rate after subject is standing (for approximately 2 minutes).
- Record current CD-LD regimen, other PD medications and their dosing schedule, and other concomitant medications.
- Record AEs.
- Perform a 12-lead ECG.
- Administer C-SSRS (Appendix O).
- Determine MoCA Score in the "On" state (Appendix D).
- Collect blood and urine samples for clinical laboratory studies (Appendix R).
- Determine Hoehn and Yahr staging of PD in the "On" state (part of MDS-UPDRS Part III Motor Examination) (Appendix E).
- Administer MDS-UPDRS Parts I through IV in the "On" and "Off" state (Appendix E).
- Train the subject how to complete the PD Diaries to assess his/her "On" and "Off" states, including assessment of any dyskinesia. After training the subject, perform the concordance testing. The subject's "On"/"Off" ratings must agree at least 75% of the time with the trained rater during the training sessions. That is, the subject's "On"/"Off" ratings must agree with the trained rater's ratings on at least 75% "On"/"Off" states in a single session to qualify for study inclusion. The 75% concordance rate must be based on 8 ratings, and must include at least one "On" and one "Off" state. The ratings should occur every 30 minutes and each session should last up to 4 hours. If the subject fails the first training session, the subject may be trained for one additional 4-hour training session. This repeat testing should not be

performed on the same day as the first session and can take place from 1 day to 3 weeks post Screening visit.

- Dispense PD Diaries and instruct the subject to complete the PD Diaries on 3 consecutive days immediately prior to Visit 1.

Notify individuals who successfully complete screening procedures following review of all study entry criteria and clinical laboratory results.

The interval between Screening and Visit 1 (Day 1) should not exceed 4 weeks.

8.2. Visit 1 – Start of IR CD-LD Dose Adjustment

8.2.1. Prior to Visit 1

Contact the subject at least 4 days prior to Visit 1 to remind him/her to complete the 3-day PD Diaries starting 3 consecutive days immediately prior to Visit 1.

The day prior to Visit 1, remind subjects to:

- Bring their completed 3-day PD Diaries to the clinic.

8.2.2. At Visit 1

For Visit 1 complete the following procedures:

- Collect and review the subject's 3-day PD Diaries. Ensure that the subject is averaging at least 2.5 hours per day of "Off" time over 3 days and at least 1.5 hours of "Off" time on each day based on the 3 day PD Diaries. If the subject cannot properly complete the diary, eg, if more than 1 day of the diary is not returned or if more than 1 day of the diary is not valid (ie, more than 2 hours [4 half periods] of the 24-hour diary are missing), he/she will not continue in the study.
- Review inclusion and exclusion criteria to ensure that the subject continues to meet these criteria.
- Review instruction of 3-day PD Diaries if needed.
- Measure vital signs (respiratory rate, blood pressure, and heart rate after supine for at least 5 minutes, and orthostatic pulse and blood pressure after standing for approximately 2 minutes). Orthostatic vital sign measurements (blood pressure and heart rate) will be performed in triplicate, each set separated by at least 15 minutes from the previous set.
- Administer C-SSRS (Appendix O).
- Administer MDS-UPDRS Parts I through IV (Appendix E).
- Complete PDQ-39 (Appendix J).
- Complete PGI-S (Appendix H).
- Complete CGI-S (Appendix I).
- Complete GCSI (Appendix K).

- Complete Non-motor Symptom assessment scale for PD (NMSS) (Appendix L).
- Complete the Parkinson's Disease Sleep Scale-2 (PDSS-2) (Appendix M).
- Complete Parkinson Anxiety Scale (PAS) (Appendix N).
- Record any AEs and update changes in concomitant medication since the previous visit.
- Dispense PD Diaries.
- Contact IWRS and dispense study medication per IWRS instructions.

8.2.3. Post Visit 1

- Make regular phone calls (approximately every 1 to 3 days) while the IR CD-LD dose is being adjusted. The IR CD-LD dosing regimen should be stable for at least 5 days prior to returning for Visit 2.

8.3. Visits 2 (Week 3) and Visit 3 (Week 5) – IPX203 Dose Conversion

8.3.1. Prior to Visit 2

- Call subjects 4 days prior to Visit 2 and remind them to complete their PD Diaries.
- Call subjects the day prior to Visit 2 to remind them to bring the PD Diaries, empty medication bottles, and any unused study drug to the office.

8.3.2. Prior to Visit 3

- Call subjects the day prior to Visit 3 to remind them to bring back empty medication bottles and any unused study drug to the office.

8.3.3. At Visits 2 and 3

For Visits 2 and 3 complete the following procedures:

- Measure vital signs (respiratory rate, blood pressure, and heart rate after supine for at least 5 minutes, and orthostatic pulse and blood pressure after standing for approximately 2 minutes).
- Administer C-SSRS (Appendix O).
- Collect empty medication bottles and any unused study drug; perform study drug accountability.
- Record and update AEs and concomitant medications.
- Contact IWRS and dispense study medication per IWRS instructions.

Additional Assessments at Visit 2 Only

- Administer MDS-UPDRS Parts I through IV (Appendix E).

- Review PD Diaries. The subject will be terminated from the study if the subject does not average at least 2.5 hours per day of “Off” time over 3 days and at least 1.5 hours of “Off” time on each day based on the 3 day PD Diaries and/or if the subject cannot properly complete the diary, eg, if more than 1 day of the diary is not returned or if more than 1 day of the diary is not valid (ie, more than 2 hours [4 half periods] of the 24 hour diary are missing).
- Ensure that the subject has been on a stable dosing regimen for at least 5 days prior to dose conversion to IPX203. If the subject is not able to achieve a stable dosing regimen lasting at least 5 days, the subject will be discontinued.
- Conduct PD Diaries training, if needed.

<u>Additional Activities at Visit 3 Only</u>

- Dispense PD Diaries.
- Review instruction of 3-day PD Diaries if needed.

8.3.4. Post Visits 2 and 3

Make regular phone calls (approximately every 1 to 3 days) to subjects throughout the dose conversion period, as needed, to evaluate each subject’s adjustment to the study medication. The IPX203 dosing regimen should be stable for at least 5 days prior to returning for Visit 4.

8.4. Visit 4 (Week 7) – Randomization

8.4.1. Prior to Visit 4

- Call subjects 4 days prior to Visit 4 to remind them to complete their PD Diaries.
- Contact subjects 1 day prior to Visit 4 to remind them to bring back the PD Diaries, empty medication bottles, and any unused study drug to the office.

8.4.2. At Visit 4

For Visit 4 complete the following procedures:

- Review PD Diaries. At least 1 day of valid diary data (ie, less than 2 hours [4 half periods] of the 24-hour diary are missing) must be available, otherwise the subject will be terminated from the study.
- Ensure that the subject has been on a stable dosing regimen for at least 5 days prior to randomization. If the subject is not able to achieve a stable dosing regimen lasting at least 5 days, the subject will be discontinued.
- Measure vital signs (respiratory rate, blood pressure, and heart rate after supine for at least 5 minutes, and orthostatic pulse and blood pressure after standing for approximately 2 minutes). Orthostatic vital sign measurements (blood pressure and heart rate) will be performed in triplicate, each set separated by at least 15 minutes from the previous set.
- Administer C-SSRS (Appendix O).

- Administer MDS-UPDRS Parts I through IV (Appendix E).
- Complete PDQ-39 (Appendix J).
- Complete PGI-S (Appendix H).
- Complete CGI-S (Appendix I).
- Complete NMSS (Appendix L).
- Complete PDSS-2 (Appendix M).
- Complete PAS (Appendix N).
- Record and update AEs and concomitant medications.
- Conduct PD diaries training, if needed.
- Collect empty medication bottles and any unused study drug; perform study drug accountability.
- Contact IWRS for randomization and dispense medication per IWRS instructions.
- Dispense PD diaries.

8.5. Visit 5 (Week 10) and Visit 6 (Week 15)

8.5.1. Prior to Visit 5 and 6

- Call subjects 4 days prior to Visits 5 and 6 to remind them to begin recording in their PD diaries on each of the 3 consecutive days immediately prior to each of these visits.
- Call the subjects the day prior to Visits 5 and 6 to remind the subjects to bring in their PD diaries, empty medication bottles, and any unused study drug to the office.

8.5.2. At Visit 5 and 6

For Visits 5 and 6 complete the following procedures (note visit-specific tasks below):

- Measure vital signs (respiratory rate, blood pressure, and heart rate after supine for at least 5 minutes, and orthostatic pulse and blood pressure after standing for approximately 2 minutes).
- Administer C-SSRS (Appendix O).
- Administer MDS-UPDRS Parts I through IV (Appendix E).
- Complete PGI-C (Appendix F).
- Complete CGI-C (Appendix G).
- Review PD diaries.
- Conduct PD diaries training, if needed.
- Dispense PD diaries.

- Collect empty medication bottles and any unused study drug; perform study drug accountability.
- Contact IWRS and dispense study medication per IWRS instructions.
- Record any AEs and update changes in concomitant medication since the previous visit.

Additional Activities at Visit 5 Only:

- Record weight.
- Perform a 12-lead ECG.
- Collect blood and urine samples for clinical laboratory studies (Appendix R).

Additional Activities at Visit 6 Only:

- Complete PDQ-39 questionnaire (Appendix J).
- Complete NMSS (Appendix L).
- Complete the PDSS-2 (Appendix M).
- Complete PAS (Appendix N).

8.6. Visit 7 (Week 20) – End of Study/Study Exit

8.6.1. Prior to Visit 7

- Call subjects 4 days prior to Visit 7 to remind them to begin recording in their PD Diaries on each of the 3 consecutive days immediately prior to Visit 7.
- Call the subjects the day prior to Visit 7 to remind the subjects to bring in their PD Diaries, empty medication bottles, and any unused study drug to the office.

8.6.2. At Visit 7

All enrolled subjects must complete Study Exit procedures at the end of Visit 7 or during an early termination visit:

- Perform physical examination, including weight.
- Measure vital signs (temperature, respiratory rate, blood pressure, and heart rate after supine for at least 5 minutes, and orthostatic pulse and blood pressure after standing for approximately 2 minutes).
- Collect and review 3-day PD Diaries.
- Administer C-SSRS (Appendix O).
- Administer MDS-UPDRS Parts I through IV (Appendix E).
- Complete PGI-C (Appendix F).
- Complete CGI-C (Appendix G).

- Complete PDQ-39 questionnaire (Appendix J).
- Complete NMSS (Appendix L).
- Complete PDSS-2 (Appendix M).
- Complete PAS (Appendix N).
- Complete PGI-S (Appendix H).
- Complete CGI-S (Appendix I).
- Complete GCSI (Appendix K).
- Collect empty medication bottles and any unused study drug; perform study drug accountability.
- Record and update AEs and concomitant medications.
- Collect blood and urine samples for clinical laboratory studies (Appendix R).
- Perform a 12-lead ECG.
- Contact IWRS to record subject disposition.

8.7. Early Termination

8.7.1. Subjects Who Terminate Prior to Randomization

If the subject discontinues the study prior to randomization (Visit 4) the subject should complete the following assessments:

- Perform physical examination, including weight.
- Measure vital signs (temperature, respiratory rate, blood pressure, and heart rate after supine for at least 5 minutes, and orthostatic pulse and blood pressure after standing for approximately 2 minutes).
- Collect and review 3-day PD Diaries when available.
- Administer C-SSRS (Appendix O).
- Administer MDS-UPDRS Parts I through IV (Appendix E).
- Collect empty medication bottles and any unused study drug; perform study drug accountability.
- Record and update AEs and concomitant medications.
- Collect blood and urine samples for clinical laboratory studies (Appendix R).
- Perform a 12-lead ECG.
- Contact IWRS to record subject disposition.

8.7.2. Subjects Who Terminate Early after Randomization

If the subject discontinues the study after randomization (Visit 4), the subject should complete all assessments described in Section 8.6.2.

8.8. Blood Volume

Safety blood draws: Approximately 10 mL of blood will be drawn at Screening, Visit 5, and at Study Exit, for a combined total of 30 mL.

9. TREATMENT OF SUBJECTS

9.1. Description of Study Drug

Study drugs will be provided by Impax for this study:

- IPX203 (carbidopa-levodopa) Extended-Release Capsules containing 35-140 mg of CD-LD for oral administration. The CD-LD ratio is 1:4. In addition, matching placebo capsules will also be provided.
- Immediate-release carbidopa-levodopa (IR CD-LD) tablet containing 25-100 mg of CD-LD, for oral administration. In addition, matching placebo tablets will also be provided.

Table 5: Study Drugs for Study IPX203-B16-02

Investigational Product	Dosage Strength (mg CD-LD) and Form
IPX203 (carbidopa-levodopa) Extended-Release capsules	35-140 mg<br>Capsules for oral administration
IR CD-LD (carbidopa-levodopa) tablets	25-100 mg<br>Tablets for oral administration
IPX203 Placebo capsules	Capsules for oral administration
IR CD-LD Placebo tablets	Tablets for oral administration

9.2. Concomitant Medications

9.2.1. Permitted PD Medications

Concomitant therapy with amantadine, selective monoamine oxidase (MAO) type B inhibitors (eg, selegiline, rasagiline), anticholinergic PD medications (eg, benztropine, trihexyphenidyl), and/or dopamine agonists (except apomorphine) is allowed provided the doses and regimens have been stable for at least 4 weeks prior to Visit 1 and the therapy is intended to be constant throughout the course of the study.

9.2.2. Prohibited Medications and Procedures

Prohibited medications and procedures include the following:

- Received within 4 weeks of Visit 1 or planning to take during participation in the clinical study: any doses of a controlled-release (CR) CD-LD apart from a single daily bedtime dose or any doses of Rytary, additional CD (eg, Lodosyn) or benserazide (eg, Serazide), or catechol-O-methyl transferase inhibitors (entacapone or tolcapone) or medications containing these inhibitors (Stalevo).

- Rescue with additional or modified doses of concomitant PD medications or with use of CD-LD products other than the dispensed study medications is not permitted and will trigger discontinuation from the study.
- Received within 4 weeks of Visit 1 or planning to take during participation in the clinical study: nonselective MAOI, selective MAO type A inhibitor (eg, phenelzine, moclobemide, pirlindole, bifemelane, toloxatone), apomorphine, or antidopaminergic agents including antiemetics.
- Treatment with any dopamine antagonist antipsychotic agents for the purposes of psychosis or bipolar disorder within the last 2 years. Use of antipsychotics to treat conditions other than psychosis or bipolar disorders may be allowed only after consultation with the medical monitor.
- Any neurosurgical procedure for the treatment of PD during the course of the study.

A subject who reports the use of any prohibited medications or procedure will be discontinued.

All medications taken within 30 days prior to signing the informed consent form (ICF) and all concomitant medications taken during the study will be recorded on the case report form (CRF).

9.2.3. Rescue Medications

Rescue with additional or modified doses of concomitant non-CD-LD PD medications is not permitted and will trigger discontinuation from the study. During the dose adjustment and dose conversion periods, rescue with CD-LD products other than the dispensed study medications is not permitted and will trigger discontinuation from the study. No medication adjustments are allowed following randomization and during the double-blind phase of the study and will trigger discontinuation from the study.

9.3. Treatment Compliance

Study drug accountability and reconciliation will be performed by the study staff and the study monitor(s).

9.4. Randomization and Blinding

At Visit 4, subjects will be randomized, stratified by center, in a 1:1 ratio into one of two double-blind parallel treatment arms of IPX203 (and matching IR CD-LD placebo) or IR CD-LD (and matching IPX203 placebo).

10. STUDY DRUG MATERIALS AND MANAGEMENT

10.1. Study Drug

Study drugs include the investigational treatment IPX203 35-140 mg CD-LD (and matching placebo capsules) and the active comparator treatment IR 25-100 mg CD-LD, (and matching placebo tablets).

IPX203 is an extended-release (ER) capsule formulation of CD-LD. Impax will manufacture and provide the IPX203 and matching placebo.

IR CD-LD is commercially available and will be provided by Impax. Matching placebo tablets will be manufactured and provided by Impax.

10.2. Study Drug Packaging and Labeling

Impax or designee will provide study medications in bottles with appropriate labeling affixed.

Labels on the study medication may include the following information:

- name, address, and phone number of the sponsor
- pharmaceutical dosage form/route of administration, quantity of dosage units, the name/identifier, and strength/potency
- batch and/or code number to identify the contents and packaging operation
- trial reference code (protocol number)
- trial subject identification number/treatment number and where relevant, the visit number
- name of investigator
- directions for use: Take tablet(s) or capsule(s) orally with water as directed.
- for clinical trial use only
- storage information: Store at 25°C (77°F), with excursions permitted to 15°C to 30°C (59°F to 86°F). Protect from light and moisture.
- period of use (use-by date, expiry date or retest date as applicable), in month/year format and in a manner that avoids any ambiguity.
- keep out of reach of children
- caution statement: Caution: New Drug—Limited by Federal (or United States) law to investigational use.

10.3. Study Drug Storage

The clinical site should store the study drug at 25°C (77°F), with excursions permitted to 15°C to 30°C (59°F to 86°F). The study drug should be stored in a tightly closed container, protected

from light and moisture. Storage temperature excursions above 30°C (86°F) should be reported by the clinical site to Impax or its designee.

10.4. Study Drug Administration

Subjects will be instructed to take their medications with approximately 240 mL of room-temperature water. The capsules or tablets should not be crushed or chewed.

IR CD-LD tablets may be split to achieve the required doses.

10.5. Study Drug Dispensing and Accountability

The Investigator must ensure that all study medication received at the study site is inventoried and accounted for, and that dispensed study medication is recorded in the subject's source documents, the CRF, and the study medication inventory log. Site personnel must not relabel or reassign study medication to other subjects or to individuals not enrolled in the study. The study monitor verifies medication accountability during monitoring visits.

10.6. Study Drug Handling and Disposal

The Investigator must retain and properly store all partially used and unused study medication until authorized by Impax regarding disposition.

11. ASSESSMENT OF EFFICACY

11.1. Parkinson's Disease Diary

Subjects are to record "asleep," "Off," and "On" without or with (nontroublesome or troublesome) dyskinesias during waking hours every 30 minutes over a 24-hour day. In the PD Diaries, subjects are instructed to indicate for each half-hour their predominant state during most of that period. "Off" is defined as the typical functional state when the medication is no longer providing benefit with regard to mobility, slowness, and stiffness in spite of taking medications. "On" is defined as the typical functional state when a subject has received medication and the medication is providing benefit with regard to mobility, slowness, and stiffness. Dyskinesias are defined as involuntary and irregular twisting and/or turning movements. Dyskinesia movements are usually an effect of medication and occur during "On" time. Nontroublesome dyskinesias do not interfere with function or do not cause meaningful discomfort. Troublesome dyskinesias do interfere with function or do cause meaningful discomfort.

11.2. Patient and Investigator Global Assessments

- Patient Global Impression of Change (Appendix F): The patient will compare his/her condition from the start of the study on a 7-point scale ranging from "Very much worse" (1) to "Very much improved" (7) at the time of the assessment.
- Clinical Global Impression of Change (Appendix G): The clinician will compare the subjects' condition from the start of the study on a 7-point scale ranging from "Very much worse" (1) to "Very much improved" (7) at the time of the assessment.
- Patient Global Impression of Severity (Appendix H): The patient will determine the severity of the disease on a 7-point scale ranging from "Normal, not at all ill" (1) to "Extremely severely ill" (7) at the time of the assessment.
- Clinical Global Impression of Severity (Appendix I): The clinician will determine the severity of the disease on a 7-point scale ranging from "Normal, not at all ill" (1) to "Among the most extremely ill of subjects" (7) at the time of the assessment.

11.3. Movement Disorders Society Version of Unified Parkinson's Disease Rating Scale

The MDS-UPDRS has 4 parts:

- Part I: Non-Motor Aspects of Experiences of Daily Living (nM-EDL) has 2 components. Component IA contains a number of behaviors assessed by the investigator with all pertinent information from the patients and caregivers. Component IB is completed by the patient with or without help from the caregiver but independent of the investigator. These sections can be reviewed by the rater to ensure all questions are answered clearly and the rater can help explain any ambiguities.

- Part II: Motor Aspects of Experiences of Daily Living (M-EDL) is a self-administered questionnaire but can be reviewed by the investigator to ensure completeness and clarity.
- Part III: Motor Examination assesses the motor signs of PD and has instructions for the rater to give to or to demonstrate to the patient. It is completed by the rater.
- Part IV: Motor Complications integrates patient-derived information with the rater's clinical observations and judgements and is completed by the rater. It contains instructions for the rater and instructions to be read to the patient.

11.4. Additional Assessments

- Parkinson's Disease Questionnaire-39 (PDQ-39) is a self-reported questionnaire. Using the 39-items, 8 domains are defined: mobility (Questions 1-10), activities of daily living (ADL) (Questions 11-16), emotional well-being (Questions 17-22), stigma (Questions 23-26), social support (Questions 27-29), cognition (Questions 30-33), communication (Questions 34-36) and bodily discomfort (Questions 37-39).
- Non-Motor Symptom assessment scale for Parkinson's Disease (NMSS) is a 30-item investigator rated questionnaire. The NMSS contains 9 domains: cardiovascular (Questions 1, 2), sleep/fatigue (Questions 3-6), mood/cognition (Questions 7-12), perceptual problems (Questions 13-15), attention/memory (Questions 16-18), gastrointestinal (Questions 19-21), urinary (Questions 22-24), sexual function (Questions 25, 26), and miscellaneous (Questions 27-30).
- Parkinson's Disease Sleep Scale-2 (PDSS-2) is 15-item self-reported questionnaire. Three domains are defined: disturbed sleep (Questions 1-3, 8, 14), motor symptoms at night (Questions 4-6, 12, 13), PD symptoms at night (Questions 7, 9-11, 15).
- Parkinson Anxiety Scale (PAS) is a 12-item patient or observer rated questionnaire with 3 domains: persistent anxiety (Questions A.1-A.5), episodic anxiety (Questions B.1-B.4) and avoidance anxiety (Questions C.1-C.3).

12. ASSESSMENT OF SAFETY

12.1. Safety Parameters

Safety will be assessed by the following parameters:

- Electrocardiograms (ECGs), clinical laboratory tests, physical examinations, the Columbia-Suicide Severity Rating Scale (C-SSRS), and vital signs, including supine and standing orthostatic blood pressure and heart rate.
- Adverse events and concomitant medications will be evaluated throughout the course of the study.

12.2. Adverse Events

12.2.1. Definition of Adverse Event

An adverse event (adverse experience) is any untoward medical occurrence in a patient or clinical trial subject administered a medicinal product and which does not necessarily have to have a causal relationship with this treatment. An adverse event can therefore be any unfavorable and unintended sign (eg, an abnormal laboratory finding), symptom, or disease temporally associated with the use of a medicinal product, whether or not considered related to the medicinal product.

All AEs and any clinically significant physical examination findings, 12-lead ECG abnormalities, or clinical laboratory measurements occurring during the study that were not present prior to administration of study medication and that continue at Study Exit should be followed and evaluated with additional tests, if necessary, until the AEs are medically stable or resolved. Follow-up on these AEs should be recorded on the source documents and reported to Impax.

12.2.2. Recording Adverse Events

Elicit information about AEs with nonselective questions such as: “Have you experienced any changes in your health status since your last visit?” Encourage subjects to report AEs at onset.

Record information for any AE that emerges from the time the subject signs the ICF until Study Exit.

Monitor each subject closely for the development of AEs and record all such events on the AE page of the CRF. Whenever possible, group signs and symptoms that constitute a single diagnosis. For example, cough, rhinitis, and sneezing might be grouped as upper respiratory infection.

For each AE, record the onset date, severity, seriousness, relationship to study medication, date of resolution (or continuing), action taken, and outcome in the CRF. The Investigator is to make a causality assessment (relationship to study medication) for every AE.

12.2.3. Follow-up

The Investigator must follow each AE until resolved or medically stable.

12.2.4. Relationship to Study Drug

The Investigator documents his/her opinion of the relationship of the AE to the study medication as follows:

- Not Related—the experience can be readily explained by the subject's underlying medical condition or concomitant medications and no relationship exists between the study medication and the experience.
- Unlikely Related—the temporal relationship between the AE and the administration of the study medication is uncertain and it is likely that the AE can be explained by the subject's medical condition or other therapies.
- Possibly Related—there is some logical temporal relationship between the AE and the administration of the study medication and the experience is unlikely to be explained by the subject's medical condition or other therapies.
- Related—the temporal relationship is compelling between the administration of the study medication and the AE cannot be explained by the subject's medical condition or other therapies.

12.2.5. Assessment of Severity

Grade each AE for severity and note in the description of the AE. Determine the severity category of mild, moderate, or severe, as defined below, and enter the information on the AE page of the CRF.

- Mild—causing no limitation of usual activities
- Moderate—causing some limitation of usual activities
- Severe—causing inability to carry out usual activities

12.3. Serious Adverse Events

12.3.1. Definition of Serious Adverse Event

A serious adverse event (SAE) is any AE occurring at any dose that results in any of the following outcomes, regardless of relationship to the study medication:

- Death
- A life-threatening adverse drug experience
- Inpatient hospitalization or prolongation of existing hospitalization
- A persistent or significant disability/incapacity
- A congenital anomaly/birth defect

- Important medical events that may not result in death, be life-threatening, or require hospitalization may be considered an SAE when, based upon appropriate medical judgment, they may jeopardize the subject and may require medical or surgical intervention to prevent one of the outcomes listed in this definition.

12.3.2. Reporting Serious Adverse Events

Any SAE that occurs from the time the subject signs an ICF until 30 days after taking the final dose of study medication must be reported by the investigative staff to the Sponsor or the Sponsor's representative within 24 hours of knowledge of the event (see Study Contact Information).

An SAE form must be completed and sent to the Sponsor and/or the Sponsor's representative. All SAEs must also be recorded on the AE page of the CRF. Additionally, all SAEs must be reported to the institutional review board (IRB) per the IRB's requirements.

Those SAEs that are considered both serious and unexpected and related to the study drug are subject to expedited reporting. An "unexpected AE" is any AE where the nature or severity is not consistent with the current investigator brochure (IB) or if an IB is not required or available, the specificity or severity is not consistent with the provided risk information.

Unexpected fatal or life-threatening SAEs related to the study drug must be reported by the Sponsor to the appropriate regulatory authority in an expedited manner (ie, first report within 7 days of first knowledge by the Sponsor). The Sponsor will provide a final written report to that authority within 15 days of initial receipt of information on the event. The Sponsor or the Sponsor's representative will also inform all participating Investigators of the SAE.

Unexpected SAEs that are not fatal or life-threatening must be reported by the Sponsor to the appropriate regulatory authority as soon as possible but no later than 15 calendar days after first knowledge of the SAE by the Sponsor. The Sponsor or the Sponsor's representative also informs all participating Investigators of the SAE.

Subjects withdrawn from the study due to any SAE will be followed until the SAE is resolved or medically stable. Record all SAEs, regardless of severity and whether or not related to the study medication, on the appropriate page of the CRF.

The Investigator must determine whether the seriousness of the event warrants removal of the subject from the study. He/she should, in any case, institute appropriate diagnostic and therapeutic measures and keep the subject under observation for as long as is medically indicated, or refer the subject to appropriate health professionals.

12.4. Pregnancy

Any pregnancy that occurs from the time the subject signs an ICF until 30 days after taking the final dose of study medication must be reported within 24 hours to the Sponsor or the Sponsor's representative and the subject should be terminated from the study. All pregnancies will be followed through to delivery of the infant. If the subject experiences a termination of the pregnancy, it should be reported as defined in Section 12.3.2.

12.5. Other Safety Parameters and Related Information

Additional safety parameters (laboratory tests, 12-lead ECGs, physical examinations, and vital signs), the C-SSRS, the GCSI, and concomitant medications are collected as shown in the Schedule of Assessments in Table 4. Clinical laboratory assessments are listed in Appendix R.

13. STATISTICS

13.1. Study Design and Sample Size Estimation

This is a multicenter, randomized, double-blind, double-dummy, active-controlled, parallel-group study. Assuming a difference of 1 hour between IPX203 and IR CD-LD in "Good on" time and a standard deviation of the treatment differences to be 3.0 hours, a sample size of 210 subjects per arm will be needed to ensure at least 90% power at a 0.05 significance level.

Assuming approximately an 18% prerandomization drop-out, approximately 510 subjects would need to be enrolled to randomize 420 subjects.

13.2. Demographics/Baseline Comparability

The demographics and baseline characteristics will be summarized by treatment arms and overall using descriptive statistics. Demographics information includes age, sex, and race. Baseline disease characteristics include MDS-UPDRS Parts I, II, III, and IV, Hoehn and Yahr stage, MoCA scores, and age of onset of PD. Distributions of dosing information, including LD doses and years on LD, will also be summarized.

13.3. Efficacy Endpoints

- Primary endpoint: Change from baseline in "Good on" time in hours per day, averaged over the PD Diary days, at the end of double-blind treatment period (Visit 7 or early termination). "Good on" time is derived from the 3-day PD Diaries and is defined as the sum of "On" time without dyskinesia and "On" time with nontroublesome dyskinesia.
- Key secondary endpoints:
  - Change from baseline in "Off" time in hours per day, averaged over the PD Diary days at the end of double-blind treatment period (Visit 7 or early termination)
  - Proportion of subjects with either "much improved" or "very much improved" in Patient Global Impression of Change (PGI-C) scores at the end of double-blind treatment period (Visit 7 or early termination)
  - Change from baseline in the MDS-UPDRS Part III at the end of double-blind treatment period (Visit 7 or early termination)
  - Change from baseline in the sum of MDS-UPDRS Parts II and III at the end of double-blind treatment period (Visit 7 or early termination)
- Additional endpoints:

  The following endpoints will be evaluated (at the post-randomization visits) as change from baseline (Visit 4) as well as change from the study entry (Visit 1), when applicable, by visits:
  - Percent "Off" time during waking hours derived from the 3-day PD Diaries
  - Average duration of each continuous "Good on" and each continuous "On"

- Hours of (1) “Off” time (from Visit 1), (2) “Good on” time (from Visit 1), (3) “On” time with dyskinesia, (4) “On” time with troublesome dyskinesia, and (5) “On” time with nontroublesome dyskinesia, and (6) asleep time derived from the 3-day PD Diaries
- Proportion of subjects with an improvement in “Good on” time of at least 1, 1.5, 2, 2.5, and 3 hours
- Proportion of subjects with a reduction in “Off” time of at least 0.5, 1, 1.5, 2, 2.5, and 3 hours
- Proportions of subjects who are “On” upon awakening and “Good on” upon awakening
- Average time to “On” upon awakening
- Change from baseline in the average number of motor fluctuations per day averaged over the PD Diary days. A motor fluctuation is defined as a change from “Off” to “On” state or from “On” to “Off” state.
- MDS-UPDRS total score (sum of Parts I, II, III, and IV) and Parts I, II, and IV separately
- MDS-UPDRS Part III and Parts II + III combined (from Visit 1)
- MDS-UPDRS Part II Question 2.9
- PDQ-39 total score and individual domain scores
- NMSS total score and individual domains
- PDSS-2 total score and individual domains
- PDSS-2 items 9, 10, 11, 12, and 13 combined
- PAS total score and individual domains
- PGI-S
- Proportion of subjects with either “severely ill” or “extremely severely ill” on the PGI-S
- CGI-S
- Proportion of subjects with either “severely ill” or “among the most extremely ill of subjects” on the CGI-S
- PGI-C scores
- CGI-C scores
- Proportion of subjects with either “much improved” or “very much improved” on the CGI-C

13.4. Analysis of Efficacy Data

In order to control the type I error rate, the primary efficacy endpoint and key secondary efficacy endpoints will be tested in a single hierarchical order as detailed in Section 13.8.

13.4.1. Primary Efficacy Endpoint

The primary efficacy endpoint is the mean change from baseline in "Good on" time in hours per day, averaged over the PD Diary days, at the end of the double-blind therapy (Visit 7 or early termination). "Good on" time is derived from the 3-day PD Diaries. For each day, "Good on" time is calculated by adding the number of half-hour intervals in which either an "On without dyskinesia" or "On with nontroublesome dyskinesia" is checked.

The primary efficacy endpoint will be analyzed using a mixed model for repeated measures (MMRM). The model will include baseline (Visit 4) "Good on" time as a covariate, treatment, visit, and pooled center as fixed effects, and a treatment-by-visit interaction. The model will employ an unstructured within subject covariance matrix and a restricted maximum likelihood (ReML) estimation method. The degree-of-freedom of the denominator will be estimated using the Kenward-Roger method (Kenward and Roger 1997). The primary analysis population will be the modified intent-to-treat as defined in Section 13.9. Missing data will be handled as in Section 13.10.

If the model fails to converge due to the unstructured covariance matrix, a simpler covariance matrix will be employed in the order of 1) heterogeneous Toeplitz [SAS PROC MIXED type = TOEPH], 2) heterogeneous autoregressive of order 1 [type = ARH(1)], 3) heterogeneous compound symmetry [type = CSH], 4) Toeplitz [type = TOEP], 5) autoregressive of order 1 [type = AR(1)], 6) compound symmetry [type = CS]. The first covariance structure that does not have a convergence problem will be the one used for the primary analysis.

13.4.2. Key Secondary Efficacy Endpoints

The first key secondary endpoint is the mean change from baseline in "Off" time in hours per day, averaged over the PD Diary days, at the end of the double-blind therapy (Visit 7 or early termination). "Off" time is derived from the 3-day PD Diaries. For each day, "Off" time is calculated by adding the number of half-hour intervals in which an "Off" is checked. This endpoint will be analyzed using a MMRM model with baseline (Visit 4) "Off" time as a covariate, treatment, visit, and pooled center as fixed effects, and a treatment-by-visit interaction. The model will employ an unstructured within subject covariance matrix and a restricted maximum likelihood (ReML) estimation method. The degree-of-freedom of the denominator will be estimated using the Kenward-Roger method (Kenward and Roger 1997).

The proportion of subjects with either "much improved" or "very much improved" in PGI-C scores at the end of the double-blind therapy (Visit 7 or early termination), the second key secondary endpoint, will be analyzed using a Cochran-Mantel-Haenszel test to compare the two treatment groups with pooled center as a stratification factor.

The mean change from baseline in the MDS-UPDRS Part III at the end of the double-blind therapy (Visit 7 or early termination) is the third key secondary endpoint. This endpoint will be analyzed using an MMRM model with baseline (Visit 4) MDS-UPDRS Part III as a covariate,

treatment, visit, and pooled center as fixed effects, and a treatment-by-visit interaction. The model will employ an unstructured within subject covariance matrix and a restricted maximum likelihood (ReML) estimation method. The degree-of-freedom of the denominator will be estimated using the Kenward-Roger method (Kenward and Roger 1997).

The mean change from baseline in sum of the MDS-UPDRS Parts II and III at the end of the double-blind therapy (Visit 7 or early termination) is the fourth key secondary endpoint. This endpoint will be analyzed using an MMRM model with baseline (Visit 4) MDS-UPDRS Parts II and III combined as covariates, treatment, visit, and pooled center as fixed effects, and a treatment-by-visit interaction. The model will employ an unstructured within subject covariance matrix and a restricted maximum likelihood (ReML) estimation method. The degree-of-freedom of the denominator will be estimated using the Kenward-Roger method (Kenward and Roger 1997).

For the 4 key secondary endpoints, the analysis population will be the modified intent-to-treat as defined in Section 13.9. Missing data will be handled as in Section 13.10.

13.4.3. Additional Efficacy Endpoints

In general, continuous endpoints will be summarized by standard descriptive statistics (mean, standard deviation, median, minimum, and maximum). Categorical endpoints will be summarized by frequencies and percentages. Comparisons between the two arms will be explored using appropriate statistical methodologies. Details will be provided in the SAP.

The primary endpoint, key secondary endpoints, as well as other efficacy endpoints will be presented by visit over the whole blinded treatment period from Baseline (Visit 4) to the end of the double-blind treatment period (Visit 7).

Other additional efficacy endpoints collected postrandomization will be analyzed by visit in a fashion similar to the primary and key secondary endpoints.

Additionally the PGI-C and CGI-C will be analyzed using analysis of variance (ANOVA) with treatment and pooled center as factors.

13.5. Center Pooling Algorithm

The center pooling algorithm is as follows.

1. Sort centers from each country from smallest to largest based on the number of subjects in the modified intent-to-treat analysis set (mITT).
2. Centers with less than 5 mITT subjects or at least one mITT subject per treatment group will be pooled with the next smallest center in the same country until the combined center (namely, pseudo-center) has more than 5 mITT subjects and at least one mITT subject per treatment group.
3. If after pooling within the same country, the pseudo-center still has less than 5 mITT subjects or still has zero subjects in one of the treatment group, that pseudo-center will be pooled with the next smallest center in the same geographical region (Western Europe, Eastern Europe, North America).

4. If after pooling within the same geographical region, the pseudo-center still has less than 5 mITT subjects or still has zero subjects in one of the treatment group, that pseudo-center will be pooled with the next smallest center in any region.

The process continues until all pooled pseudo-centers have at least 5 mITT subjects and at least one mITT subject per treatment group. These pooled centers will be used in analyses that adjust for pooled centers.

This pooling algorithm will be detailed in the Statistical Analysis Plan (SAP).

13.6. Sensitivity Analyses of the Primary Endpoint and Key Secondary Endpoints

Sensitivity analyses will be performed with respect to the primary efficacy endpoint and continuous key secondary endpoints ("Off" time, MDS-UPDRS Part III, and MDS-UPDRS Parts II and III combined) as follows.

13.6.1. Assessing Assumptions of the Mixed Model for Repeated Measures (MMRM)

a. The normality and homoscedasticity assumptions will be examined through residual analyses. The normality and homoscedasticity assumptions will further be tested via Shapiro-Wilk (Shapiro and Wilk 1965) and Levene (Levene 1960) tests, respectively, at a 0.05 level of significance. If normality and/or homoscedasticity assumption appears violated, then:

   i. Nonparametric Wilcoxon Rank Sum test will be performed to compare the two treatment groups, with missing data imputed by the last observation carried forward (LOCF) method.

   ii. Multiple imputation rank based analysis: instead of missing data imputed by the LOCF method, in this analysis, missing data at Visit 7 will be imputed multiple times to create 50 complete datasets. The multiple imputation procedure is described in Section 13.6.4 (part of the pattern-mixture model), using $f = 0\%$. The Wilcoxon Rank Sum test will be performed on each of the 50 datasets. The results are then combined using Rubin's rule (Rubin 1987) via SAS PROC MIANALYZE.

b. Missing at Random (MAR) assumption will be evaluated as discussed in Section 13.6.4.

13.6.2. Complete Case Analysis

The primary endpoint will be analyzed using an ANCOVA model with "Good on" time at baseline (Visit 4) as a covariate, pooled center and treatment as factors. The model will be performed on subjects with both baseline "Good on" time and Visit 7 "Good on" time.

13.6.3. Single LOCF/BLOCF Imputation

The primary efficacy endpoint will be analyzed using an ANCOVA model with "Good on" time at Visit 4 as a covariate, pooled center and treatment as factors. Missing data will be imputed by the LOCF and baseline observation carried forward (BLOCF) methods. These analyses will be performed on the mITT population.

13.6.4. Pattern-Mixture Model

If an overall dropout rate postrandomization is > 15%, pattern-mixture models (PMM) will be employed to assess the robustness of the results under the missing not at random (MNAR) assumption. The pattern for PMM is defined by patients' last visit with an observed primary efficacy endpoint and the reason for dropout.

Multiple imputation with mixed missing data mechanism (MNAR for a missing data pattern and MAR for others) will be used to investigate the robustness of the primary result. Four specific data patterns will be examined:

1. Dropout at Visit 5 and reason = Lack of efficacy in IPX203 treatment arm,
2. Dropout at Visit 5 and reason = Lack of efficacy or adverse events in IPX203 treatment arm,
3. Dropout at Visit 6 and reason = Lack of efficacy in IPX203 treatment arm,
4. Dropout at Visit 6 and reason = Lack of efficacy or adverse events in IPX203 treatment arm.

The missing values will be imputed 50 times (multiple imputation) under the assumption that the distribution of the missing values is the same as that of the observed values. The PMM then investigates the departure from the MAR assumption by progressively decreasing the outcome (the "penalty") for those on IPX203 arm who fall into an assumed MNAR pattern above. For the dropout subjects on IPX203 arm that fall into one of the patterns above, the "penalty" is obtained by subtracting the imputed missing data after dropout by a factor *f*, with *f* starts from 0%, 5%, 10%, 15%, 20%, 25%, 30%, …, 100% of the treatment difference seen in the primary model. This process continues until the conclusion from the primary analysis is overturned (a tipping point). In other words, if the dropout subject is from IPX203 arm and the dropout pattern falls into one of the 4 patterns above, then the subject's imputed value will be adjusted downward by a factor *f*, where *f* goes from 0% to 100% of the treatment difference seen in the primary model. Note that if 0% is used, the analysis is essentially multiple imputation under MAR assumption. On the other hand, if 100% is used, then the analysis is essentially a "jump to reference" where outcome on IPX203 arm is assumed to be the same as outcome on IR CD-LD. After imputations, the dataset will be analyzed using an MMRM model similar to the primary analysis model. The results will then be combined using the Rubin's rule (Rubin 1987) via SAS PROC MIANALYZE.

The procedure will be carried out in SAS as follows:

a. Use Monte Carlo Markov Chain (MCMC) method in SAS PROC MI by treatment group to impute the intermittent missing data to form monotone missingness.
b. Use MAR-based multiple imputation in SAS PROC MI to impute the missing data (SAS MONOTONE statement).
c. For dropout subjects in IPX203 arm who fall into an MNAR pattern specified above, a delta which equals to *f* times the treatment difference obtained from the primary MMRM analysis at Visit 7 will be subtracted from their imputed values for all visits after the dropout ("penalizing" IPX203 arm).

d. After imputation, use the MMRM model as in the primary analysis model to analyze the complete data along with the imputed data.

e. Repeat steps a through d 50 times.

Combine results using Rubin's rule (Rubin 1987) via SAS PROC MIANALYZE.

13.7. Subgroup Analyses

The primary, key secondary endpoints, as well as overall summary of adverse events, will be examined for the following subgroups.

- Age: < 65, ≥ 65 years old at study entry
- Sex: Males, Females
- Race: Caucasians, non-Caucasians

Additionally, the following subgroups may be examined:

- Region
- Ethnicity
- Concomitant medications
- Weight
- Body mass index (BMI)
- PD duration
- Age of PD onset
- "Good On" time and "Off" time at study entry.

For all subgroup efficacy analyses, the same analysis methods as the primary and key secondary endpoints will be applied, unless the sample size in one of the subgroups becomes too small to hinder the statistical analysis. In that case, no inferential statistics will be provided for such a subgroup. The details for final subgroup analyses will be documented in the SAP.

13.8. Multiplicity Adjustments

The primary endpoint and 4 key secondary endpoints will be tested in a sequential hierarchical order as follows.

1. The primary endpoint, the mean change from baseline in "Good on" time (hours per day), will be tested first at a 0.05 level of significance.
2. If statistical significance is demonstrated, then the first key secondary endpoint, the mean change from baseline in "Off" time (hours per day), will be tested next at a 0.05 level of significance.
3. If statistical significance is demonstrated, then the second key secondary endpoint, the proportion of subjects with either "much improved" or "very much improved" on the PGI-C, will be tested next at a 0.05 level of significance.

4. If statistical significance is demonstrated, then the third key secondary endpoint, the mean change from baseline in the MDS-UPDRS Part III, will be tested at a 0.05 level of significance.
5. If statistical significance is demonstrated, then the fourth key secondary endpoint, the mean change from baseline in the sum of the MDS-UPDRS Parts II and III combined will be tested next at a 0.05 level of significance.

For the other efficacy endpoints, no adjustment will be made.

13.9. Analysis Populations

13.9.1. Safety Analysis Set

The Safety Analysis set will include all subjects who were treated with any study drug.

13.9.2. Intent-to-Treat Analysis Set

The Intent-to-treat Analysis Set will include all subjects who were randomized and treated with any study drug and have a baseline and at least one postbaseline efficacy assessment.

13.9.3. Modified Intent-to-Treat Analysis Set

The Modified Intent-to-treat Analysis Set will include all subjects who were randomized and treated and have a valid baseline PD Diary and at least one valid postrandomization PD Diary. This analysis set will be used for the primary analysis and key secondary analyses.

13.9.4. Completers Analysis Set

The Completers Analysis Set will include all subjects who were randomized and treated and complete the study.

13.10. Handling of Missing Data

13.10.1. Missing Data for PD Diaries

An MMRM approach will be used to handle missing visit data. MMRM analysis will use all available valid visit data, including subjects with some missing visit data, in order to arrive at an estimate of the mean treatment effect.

A PD Diary is valid if at least 1 day of diary data is available using the rules defined below.

Imputation of missing data for a PD Diary day will be required if a PD Diary is not completed for a full day (6 am to 5:30 am). In this case, the method of imputation will be dependent upon the amount and pattern of missing data.

- For subjects with more than 1 day of diary data, the following rules will apply:
    1. If more than 4 half-hour time intervals are missing, then that particular day will not be included in the analysis. The missing data will be handled in the MMRM model.
    2. If a one-half hour time interval is missing and the observations on either side of the time interval are not missing, then the missing time interval will be imputed by

assigning a value of the previous measurement for the first 15 minutes and the value of the next measurement for the second 15 minutes.

3. If 2, 3, or 4 consecutive half-hour time intervals are missing, and these time intervals are available from other days of the visit then the following rules will be applied:
   a. For missing time intervals on Day 1, data from Day 2 will be used for imputation for the same time intervals. If Day 2 data is also incomplete or not available, then Day 3 data will be used.
   b. For missing time intervals on Day 2, data from Day 3 will be used for imputation if available; otherwise Day 1 data will be used.
   c. Data from Day 2 will be used for imputing missing time intervals on Day 3. If data from Day 2 is not available, then Day 1 data will be used for imputation.
   d. If data at the same time period are missing across all days, then the approach will be to split the individual missing half-hour intervals into 2 periods, with the first-half interval being imputed with data from the immediate previous nonmissing time period and the second-half interval being imputed with the next nonmissing time interval.

- For subjects with only 1 day of diary data, the following rules will apply:
  1. If more than 4 half-hour time intervals are missing, then that particular day will not be included in the analysis. The missing data will be handled in the MMRM model.
  2. If a one-half hour time interval is missing and the observations on either side of the time interval are not missing, then the missing time interval will be imputed by assigning a value of the previous measurement for the first 15 minutes and the value of the next measurement for the second 15 minutes.
  3. If 2, 3, or 4 consecutive half-hour intervals are missing, then the approach will be to split the individual missing half-hour intervals into 2 periods, with the first-half interval being imputed with data from the immediate previous nonmissing time period and the second-half interval being imputed with the next nonmissing time interval.

13.10.2. Missing Data for Global Assessments (PGI-C, CGI-C, PGI-S and CGI-S)

For subjects with missing PGI-C or CGI-C for a particular visit, the data will be imputed as nonresponders (ie, not being “much improved” or “very much improved”).

For subjects with missing PGI-S or CGI-S for a particular visit, the data will be imputed as nonresponders (ie, being “severely ill” or “extremely severely ill” for PGI-S and being “severely ill” or “among the most extremely ill of subjects” for CGI-S).

13.10.3. Missing Data for MDS-UPDRS

If the MDS-UPDRS are missing for the particular visit, the missing data will be handled via the MMRM model.

If component questions are missing for a particular part of the MDS-UPDRS questionnaire, the missing items are assigned the average value for other items in that part as follows (Goetz 2015):

- For Part I (13 questions): up to 1 missing question will be imputed using the average value of the remaining 12 questions.
- For Part II (13 questions): up to 2 missing questions will be imputed using the average value of the remaining 11 questions.
- For Part III (33 questions): up to 7 missing questions will be imputed using the average value of the remaining 26 questions.
- Part IV (6 questions): no imputation is done.

If more component questions are missing than above for a particular part of the MDS-UPDRS questionnaire, the entire questionnaire will not be included in the analysis for that particular assessment. Missing data will be handled in a fashion similar to PD Diary data (Section 13.10.1) using the MMRM model.

For quality-of-life endpoints, missing responses within a questionnaire will not be imputed.

13.11. Analysis of Safety

The safety analysis will include all subjects who receive at least 1 dose of study medication. Reported AEs will be coded using the Medical Dictionary for Regulatory Activities (MedDRA). All AEs will be summarized by system organ class and preferred terms within system organ class. The severity, seriousness, and relationship to study medication will also be summarized by treatment arms. Each AE (based on preferred term) is counted once for a given subject. If the same AE occurred on multiple occasions, the highest severity and least complimentary relationship will be assumed.

The incidence of treatment-emergent AEs and serious AEs will be summarized by treatment arms.

Additionally, laboratory test data, physical examinations, vital signs, ECGs, C-SSRS, and GCSI will be summarized by treatment arms.

14. ADMINISTRATIVE PROCEDURES

14.1. Guidelines for Good Clinical Practice

This study will be conducted in accordance with principles of Good Clinical Practice (GCP) as promulgated by the ICH. Good Clinical Practice is an international ethical and scientific quality standard for designing, conducting, recording, and reporting trials that involve the participation of human subjects. Compliance with this standard provides public assurance that the rights, safety, and well-being of human subjects are protected under current ethical principles, and that the clinical trial data are credible. Current GCP standards may be found in ICH Guidance E6 (Good Clinical Practice: Consolidated Guidance). This guidance describes the principles of GCP and the obligations of the institutional review board (IRB), the Investigator and the Sponsor in conducting this study in accordance with those principles.

14.2. Institutional Review Board Approval

The review of this protocol by an IRB and the performance of all aspects of the study, including the methods used for obtaining informed consent, must be in accordance with principles enunciated in the ICH and GCP Guidelines and by the appropriate regulatory authorities.

The Investigator is responsible for preparing documents for submission to the relevant IRB and obtaining written approval for this study. Institutional Review Board approval must be obtained prior to the initiation of the study. The Investigator's continued participation in the study is contingent on renewing approval with the IRB at least annually.

14.3. Informed Consent

Site personnel should prepare an Informed Consent Form (ICF) incorporating the necessary elements of consent. The ICF is to be approved by Impax prior to submission to the IRB. The Investigator or his/her staff must explain the nature of the investigation and the risks involved to each subject prior to screening, and obtain a signed ICF. The subject should also be informed that he/she is free to voluntarily withdraw from the study at any time.

14.4. Study Monitoring

Impax representatives or designees will conduct site visits to the investigational facilities for the purpose of monitoring the study. The Investigator agrees to allow the monitor to inspect the drug storage area, study drug stocks, drug accountability records, subject charts and study source documents, and other records relevant to study conduct. The Investigator must permit access to such records if a regulatory or compliance audit is required.

14.5. Protocol Amendments

All amendments to the protocol must be documented in writing, reviewed and approved by the Sponsor and Investigator, and submitted to the IRB for approval prior to implementation. If the protocol amendment substantially alters the study design or potential risk to the subject, a new

written ICF for continued participation in the study must be obtained from each subject affected by the change.

14.6. Termination of Study

The Sponsor has the right to terminate this study and remove all study material from the site at any time for medical or administrative reasons. In this event, the Sponsor will endeavor to give adequate notice to allow safe withdrawal of subjects from the study.

14.7. Case Report Forms

Site personnel should collect and record data for the study as source documents, and transfer the data into the CRF.

The Investigator must ensure that complete data for the clinical study are collected and accurately documented in the appropriate sections of the CRF and adequately supported by the appropriate source documentation. In addition, it is the Investigator's responsibility to provide signatures where requested indicating concurrence with data in the CRF.

14.8. Investigator's Final Conduct Report

At the completion of the study, the Investigator must provide Impax a copy of the final conduct report that was submitted to their IRB, including a review of AEs.

14.9. Records Retention

International Conference on Harmonization, GCP, and US FDA guidelines require that essential documents be retained until at least 2 years after the last approval of a marketing application and until there are no pending or contemplated marketing applications, or at least 2 years have elapsed since the formal discontinuation of clinical development of the investigational product.

However, the essential documents should be retained for a longer period if required by the applicable regulatory requirements or by an agreement with the Sponsor. Records should never be destroyed without written approval from the Sponsor.

If an Investigator leaves the institution, he/she must transfer responsibilities for record retention to another individual willing to accept them. The Investigator must notify the Sponsor in writing of the transfer of study documents before the transfer of the study documents.

15. PUBLICATION POLICY

Study results may not be published without prior written approval from Impax.

16. LIST OF REFERENCES

Blanchet PJ, Calon F, Martel JC, et al. Continuous administration decreases and pulsatile administration increases behavioral sensitivity to a novel dopamine D2 agonist (U-91356A) in MPTP-exposed monkeys. J Pharmacol Exp Ther. 1995;272:854-859.

Engber TM, Susel Z, Juncos JL, Chase TN. Continuous and intermittent levodopa differentially affect rotation induced by D-1 and D-2 dopamine agonists. European Journal of Pharmacology. 1989;168:291-298.

Fahn S. Parkinson disease, the effect of levodopa, and the ELLDOPA trial. Earlier vs Later L DOPA. Arch Neurol. 1999 May;56(5):529-35.

Food and Drug Administration 2003. Guidance for Industry. Bioavailability and Bioequivalence Studies for Orally Administered Drug Products — General Considerations. U.S. Department of Health and Human Services. Food and Drug Administration. Center for Drug Evaluation and Research (CDER). March 2003.

Goetz CG, Luo S, Wang L, Tilley BC, LaPelle NR, Stebbins GT. Handling missing values in the MDS-UPDRS. Mov Disord. 2015 Oct;30(12):1632-8.

Juncos JL, Engber TM, Raisman R, et al. Continuous and intermittent levodopa differentially affect basal ganglia function. Ann Neurol. 1989;25:473-478.

Kenward MG and Roger JH. Small sample inference for fixed effects from restricted maximum likelihood. Biometrics. 1997;53:983-997.

Kurlan R, Rothfield KP, Woodward WR, Nutt JG, Miller C, Lichter D, et al. Erratic gastric emptying of levodopa may cause “random” fluctuations of parkinsonian mobility. Neurology. 1988;38(3):419-21.

Levene H. In contributions to probability and statistics: Essays in honor of Harold Hotelling, I. Olkin et al. eds. Stanford University Press. 1960: 278-292.

Melamed E, Bitton V, Zelig O. Delayed onset of responses to single doses of L-dopa in parkinsonian fluctuators on long-term L-dopa therapy. Clin Neuropharmacol. 1986;9(2):182-8.

Mizuno Y. Where do we stand in the treatment of Parkinson's disease? Neurology. 2007;254:13-18.

Nilsson D, Nyholm D, Aquilonius S-M. Duodenal levodopa infusion in Parkinson's disease--long-term experience. Acta Neurologica Scandinavica. 2001;104:343-348.

Nyholm D, Nilsson Remahl AI, Dizdar N, et al. Duodenal levodopa infusion monotherapy vs oral polypharmacy in advanced Parkinson disease. Neurology. 2005;64:216-223.

Rubin DB. Multiple Imputation in Nonresponse in Surveys. San Francisco, CA: Wiley; 1987.

Shapiro SS and Wilk MB. An analysis of variance test for normality (complete samples). Biometrika. 1965;52(3-4): 591-611.

Sinemet (Carbidopa-Levodopa) Tablets Prescribing Information. Whitehouse Station, NJ: Manufactured by Merck & Co., Inc.; Marketed by Bristol-Myers Squibb Company; 2013.

Stocchi F, Quinn NP, Barbato L, Patsalos PN, O'Connel MT, Ruggieri S, et al. Comparison between a fast and a slow release preparation of levodopa and a combination of the two: a clinical and pharmacokinetic study. Clinical Neuropharmacol. 1994;17(1):38-44.

Stocchi F, Vacca L, et al. Intermittent vs continuous levodopa administration in patients with advanced Parkinson disease: a clinical and pharmacokinetic study. Arch Neurol. 2005:62(6):905-910.

17. APPENDICES

APPENDIX A. PRESCRIBING INFORMATION FOR IR CD-LD

SINEMET - carbidopa and levodopa tablet
Merck Sharp & Dohme Corp.

SINEMET®
(carbidopa levodopa)
Tablets

DESCRIPTION

SINEMET® (carbidopa levodopa) is a combination of carbidopa and levodopa for the treatment of Parkinson's disease and syndrome.

Carbidopa, an inhibitor of aromatic amino acid decarboxylation, is a white, crystalline compound, slightly soluble in water, with a molecular weight of 244.3. It is designated chemically as (—)-L-α-hydrazino-α-methyl-β-(3,4-dihydroxybenzene) propanoic acid monohydrate. Its empirical formula is $C_{10}H_{14}N_2O_4 \cdot H_2O$, and its structural formula is:

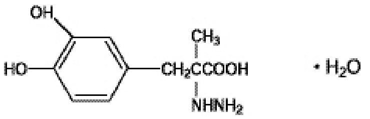

Tablet content is expressed in terms of anhydrous carbidopa which has a molecular weight of 226.3.

Levodopa, an aromatic amino acid, is a white, crystalline compound, slightly soluble in water, with a molecular weight of 197.2. It is designated chemically as (—)-L-α-amino-β-(3,4-dihydroxybenzene) propanoic acid. Its empirical formula is $C_9H_{11}NO_4$, and its structural formula is:

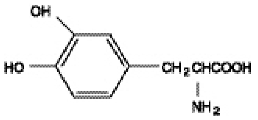

SINEMET is supplied as tablets in three strengths:

SINEMET 25-100, containing 25 mg of carbidopa and 100 mg of levodopa.

SINEMET 10-100, containing 10 mg of carbidopa and 100 mg of levodopa.

SINEMET 25-250, containing 25 mg of carbidopa and 250 mg of levodopa.

Inactive ingredients are hydroxypropyl cellulose, pregelatinized starch, crospovidone, microcrystalline cellulose, and magnesium stearate. SINEMET 10-100 and 25-250 Tablets also contain FD&C Blue #2/Indigo Carmine AL. SINEMET 25-100 Tablets also contain D&C Yellow #10 Lake.

CLINICAL PHARMACOLOGY

Mechanism of Action

Parkinson's disease is a progressive, neurodegenerative disorder of the extrapyramidal nervous system affecting the mobility and control of the skeletal muscular system. Its characteristic features include resting tremor, rigidity, and bradykinetic movements. Symptomatic treatments, such as levodopa therapies, may permit the patient better mobility.

Current evidence indicates that symptoms of Parkinson's disease are related to depletion of dopamine in the corpus striatum. Administration of dopamine is ineffective in the treatment of Parkinson's disease apparently because it does not cross the blood-brain barrier. However, levodopa, the metabolic precursor of dopamine, does cross the blood-brain barrier, and presumably is converted to dopamine in the brain. This is thought to be the mechanism whereby levodopa relieves symptoms of Parkinson's disease.

Pharmacodynamics

When levodopa is administered orally, it is rapidly decarboxylated to dopamine in extracerebral tissues so that only a small portion of a given dose is transported unchanged to the central nervous system. For this reason, large doses of levodopa are required for adequate therapeutic effect, and these may often be accompanied by nausea and other adverse reactions, some of which are attributable to dopamine formed in extracerebral tissues.

Since levodopa competes with certain amino acids for transport across the gut wall, the absorption of levodopa may be impaired in some patients on a high protein diet.

Carbidopa inhibits decarboxylation of peripheral levodopa. It does not cross the blood-brain barrier and does not affect the metabolism of levodopa within the central nervous system.

The incidence of levodopa-induced nausea and vomiting is less with SINEMET than with levodopa. In many patients, this reduction in nausea and vomiting will permit more rapid dosage titration.

Since its decarboxylase inhibiting activity is limited to extracerebral tissues, administration of carbidopa with levodopa makes more levodopa available for transport to the brain.

Pharmacokinetics

Carbidopa reduces the amount of levodopa required to produce a given response by about 75% and, when administered with levodopa, increases both plasma levels and the plasma half-life of levodopa, and decreases plasma and urinary dopamine and homovanillic acid.

The plasma half-life of levodopa is about 50 minutes, without carbidopa. When carbidopa and levodopa are administered together, the half-life of levodopa is increased to about 1.5 hours. At steady state, the bioavailability of carbidopa from SINEMET tablets is approximately 99% relative to the concomitant administration of carbidopa and levodopa.

In clinical pharmacologic studies, simultaneous administration of carbidopa and levodopa produced greater urinary excretion of levodopa in proportion to the excretion of dopamine than administration of the two drugs at separate times.

Pyridoxine hydrochloride (vitamin B_6), in oral doses of 10 mg to 25 mg, may reverse the effects of levodopa by increasing the rate of aromatic amino acid decarboxylation. Carbidopa inhibits this action of pyridoxine; therefore, SINEMET can be given to patients receiving supplemental pyridoxine (vitamin B_6).

Special Populations

Geriatric

A study in eight young healthy subjects (21-22 yr) and eight elderly healthy subjects (69-76 yr) showed that the absolute bioavailability of levodopa was similar between young and elderly subjects following oral administration of levodopa and carbidopa. However, the systemic exposure (AUC) of levodopa was increased by 55% in elderly subjects compared to young subjects. Based on another study in forty patients with Parkinson's disease, there was a correlation between age of patients and the increase of AUC of levodopa following administration of levodopa and an inhibitor of peripheral dopa decarboxylase. AUC of levodopa was increased by 28% in elderly patients (≥ 65 yr) compared to young patients (< 65 yr). Additionally, mean value of Cmax for levodopa was increased by 24% in

elderly patients (≥ 65 yr) compared to young patients (< 65 yr) (see PRECAUTIONS, Geriatric Use).

The AUC of carbidopa was increased in elderly subjects (n=10, 65-76 yr) by 29% compared to young subjects (n=24, 23-64 yr) following IV administration of 50 mg levodopa with carbidopa (50 mg). This increase is not considered a clinically significant impact.

INDICATIONS AND USAGE

SINEMET is indicated in the treatment of Parkinson's disease, post-encephalitic parkinsonism, and symptomatic parkinsonism that may follow carbon monoxide intoxication or manganese intoxication.

Carbidopa allows patients treated for Parkinson's disease to use much lower doses of levodopa. Some patients who responded poorly to levodopa have improved on SINEMET. This is most likely due to decreased peripheral decarboxylation of levodopa caused by administration of carbidopa rather than by a primary effect of carbidopa on the nervous system. Carbidopa has not been shown to enhance the intrinsic efficacy of levodopa.

Carbidopa may also reduce nausea and vomiting and permit more rapid titration of levodopa.

CONTRAINDICATIONS

Nonselective monoamine oxidase (MAO) inhibitors are contraindicated for use with SINEMET. These inhibitors must be discontinued at least two weeks prior to initiating therapy with SINEMET. SINEMET may be administered concomitantly with the manufacturer's recommended dose of an MAO inhibitor with selectivity for MAO type B (e.g., selegiline HCl) (see PRECAUTIONS, Drug Interactions).

SINEMET is contraindicated in patients with known hypersensitivity to any component of this drug, and in patients with narrow-angle glaucoma.

WARNINGS

When SINEMET is to be given to patients who are being treated with levodopa, levodopa must be discontinued at least twelve hours before therapy with SINEMET is started. In order to reduce adverse reactions, it is necessary to individualize therapy. See DOSAGE AND ADMINISTRATION **section before initiating therapy.**

The addition of carbidopa with levodopa in the form of SINEMET reduces the peripheral effects (nausea, vomiting) due to decarboxylation of levodopa; however, carbidopa does not decrease the adverse reactions due to the central effects of levodopa. Because carbidopa permits more levodopa to reach the brain and more dopamine to be formed, certain adverse central nervous system (CNS) effects, e.g., dyskinesias (involuntary movements), may occur at lower dosages and sooner with SINEMET than with levodopa alone.

All patients should be observed carefully for the development of depression with concomitant suicidal tendencies.

SINEMET should be administered cautiously to patients with severe cardiovascular or pulmonary disease, bronchial asthma, renal, hepatic or endocrine disease.

As with levodopa, care should be exercised in administering SINEMET to patients with a history of myocardial infarction who have residual atrial, nodal, or ventricular arrhythmias. In such patients, cardiac function should be monitored with particular care during the period of initial dosage adjustment, in a facility with provisions for intensive cardiac care.

As with levodopa, treatment with SINEMET may increase the possibility of upper gastrointestinal hemorrhage in patients with a history of peptic ulcer.

Falling Asleep During Activities of Daily Living and Somnolence

Patients taking SINEMET alone or with other dopaminergic drugs have reported suddenly falling asleep without prior warning of sleepiness while engaged in activities of daily living (includes operation of motor vehicles). Road traffic accidents attributed to sudden sleep onset have been reported. Although many patients reported somnolence while on dopaminergic medications, there have been reports of road traffic accidents attributed to sudden onset of sleep in which the patient did not perceive any warning signs, such as excessive drowsiness, and believed that they were alert immediately prior to the event. Sudden onset of sleep has been reported to occur as long as one year after the initiation of treatment.

Falling asleep while engaged in activities of daily living usually occurs in patients experiencing pre-existing somnolence, although some patients may not give such a history. For this reason, prescribers should reassess patients for drowsiness or sleepiness especially since some of the events occur well after the start of treatment. Prescribers should be aware that patients may not acknowledge drowsiness or sleepiness until directly questioned about drowsiness or sleepiness during specific activities. Patients should be advised to exercise caution while driving or operating machines during treatment with SINEMET. Patients who have already experienced somnolence or an episode of sudden sleep onset should not participate in these activities during treatment with SINEMET.

Before initiating treatment with SINEMET, advise patients about the potential to develop drowsiness and ask specifically about factors that may increase the risk for somnolence with SINEMET such as the use of concomitant sedating medications and the presence of sleep disorders. Consider discontinuing SINEMET in patients who report significant daytime sleepiness or episodes of falling asleep during activities that require active participation (e.g., conversations, eating, etc.). If treatment with SINEMET continues, patients should be advised not to drive and to avoid other potentially dangerous activities that might result in harm if the patients become somnolent. There is insufficient information to establish that dose reduction will eliminate episodes of falling asleep while engaged in activities of daily living.

Hyperpyrexia and Confusion

Sporadic cases of a symptom complex resembling neuroleptic malignant syndrome (NMS) have been reported in association with dose reductions or withdrawal of certain antiparkinsonian agents such as levodopa, carbidopa levodopa, or carbidopa levodopa extended release. Therefore, patients should be observed carefully when the dosage of levodopa is reduced abruptly or discontinued, especially if the patient is receiving neuroleptics.

NMS is an uncommon but life-threatening syndrome characterized by fever or hyperthermia. Neurological findings, including muscle rigidity, involuntary movements, altered consciousness, mental status changes; other disturbances, such as autonomic dysfunction, tachycardia, tachypnea, sweating, hyper- or hypotension; laboratory findings, such as creatine phosphokinase elevation, leukocytosis, myoglobinuria, and increased serum myoglobin have been reported.

The early diagnosis of this condition is important for the appropriate management of these patients. Considering NMS as a possible diagnosis and ruling out other acute illnesses (e.g., pneumonia, systemic infection, etc.) is essential. This may be especially complex if the clinical presentation includes both serious medical illness and untreated or inadequately treated extrapyramidal signs and symptoms (EPS). Other important considerations in the differential diagnosis include central anticholinergic toxicity, heat stroke, drug fever, and primary central nervous system (CNS) pathology.

The management of NMS should include: 1) intensive symptomatic treatment and medical monitoring and 2) treatment of any concomitant serious medical problems for which specific treatments are available. Dopamine agonists, such as bromocriptine, and muscle relaxants, such as dantrolene, are often used in the treatment of NMS; however, their effectiveness has not been demonstrated in controlled studies.

PRECAUTIONS

General

As with levodopa, periodic evaluations of hepatic, hematopoietic, cardiovascular, and renal function are recommended during extended therapy.

Patients with chronic wide-angle glaucoma may be treated cautiously with SINEMET provided the intraocular pressure is well-controlled and the patient is monitored carefully for changes in intraocular pressure during therapy.

Dyskinesia

Levodopa alone, as well as SINEMET, is associated with dyskinesias. The occurrence of dyskinesias may require dosage reduction.

Hallucinations / Psychotic-Like Behavior

Hallucinations and psychotic-like behavior have been reported with dopaminergic medications. In general, hallucinations present shortly after the initiation of therapy and may be responsive to dose reduction in levodopa. Hallucinations may be accompanied by confusion and to a lesser extent sleep disorder (insomnia) and excessive dreaming.

SINEMET may have similar effects on thinking and behavior. This abnormal thinking and behavior may present with one or more symptoms, including paranoid ideation, delusions, hallucinations, confusion, psychotic-like behavior, disorientation, aggressive behavior, agitation, and delirium.

Ordinarily, patients with a major psychotic disorder should not be treated with SINEMET, because of the risk of exacerbating psychosis. In addition, certain medications used to treat psychosis may exacerbate the symptoms of Parkinson's disease and may decrease the effectiveness of SINEMET.

Impulse Control / Compulsive Behaviors

Reports of patients taking dopaminergic medications (medications that increase central dopaminergic tone), suggest that patients may experience an intense urge to gamble, increased sexual urges, intense urges to spend money, binge eating, and/or other intense urges, and the inability to control these urges. In some cases, although not all, these urges were reported to have stopped when the dose was reduced or the medication was discontinued. Because patients may not recognize these behaviors as abnormal, it is important for prescribers to specifically ask patients or the caregivers about the development of new or increased gambling urges, sexual urges, uncontrolled spending or other urges while being treated with SINEMET. Physicians should consider dose reduction or stopping the medication if a patient develops such urges while taking SINEMET [see *Information for Patients*].

Melanoma

Epidemiological studies have shown that patients with Parkinson's disease have a higher risk (2- to approximately 6-fold higher) of developing melanoma than the general population. Whether the increased risk observed was due to Parkinson's disease or other factors, such as drugs used to treat Parkinson's disease, is unclear.

For the reasons stated above, patients and providers are advised to monitor for melanomas frequently and on a regular basis when using SINEMET for any indication. Ideally, periodic skin examinations should be performed by appropriately qualified individuals (e.g., dermatologists).

Information for Patients

The patient should be informed that SINEMET is an immediate-release formulation of carbidopa levodopa that is designed to begin release of ingredients within 30 minutes. It is important that SINEMET be taken at regular intervals according to the schedule outlined by the physician. The patient should be cautioned not to change the prescribed dosage regimen and not to add any additional antiparkinson medications, including other carbidopa levodopa preparations, without first consulting the physician.

Patients should be advised that sometimes a 'wearing-off' effect may occur at the end of the dosing interval. The physician should be notified if such response poses a problem to lifestyle.

Patients should be advised that occasionally, dark color (red, brown, or black) may appear in saliva, urine, or sweat after ingestion of SINEMET. Although the color appears to be clinically insignificant, garments may become discolored.

The patient should be advised that a change in diet to foods that are high in protein may delay the absorption of levodopa and may reduce the amount taken up in the circulation. Excessive acidity also delays stomach emptying, thus delaying the absorption of levodopa. Iron salts (such as in multivitamin tablets) may also reduce the amount of levodopa available to the body. The above factors may reduce the clinical effectiveness of the levodopa or carbidopa levodopa therapy.

Patients should be alerted to the possibility of sudden onset of sleep during daily activities, in some cases without awareness or warning signs, when they are taking dopaminergic agents, including levodopa. Patients should be advised to exercise caution while driving or operating machinery and that if they have experienced somnolence and/or sudden sleep onset, they must refrain from these activities. (See WARNINGS, Falling Asleep During Activities of Daily Living and Somnolence.)

There have been reports of patients experiencing intense urges to gamble, increased sexual urges, and other intense urges, and the inability to control these urges while taking one or more of the medications that increase central dopaminergic tone and that are generally used for the treatment of Parkinson's disease, including SINEMET. Although it is not proven that the medications caused these events, these urges were reported to have stopped in some cases when the dose was reduced or the medication was stopped. Prescribers should ask patients about the development of new or increased gambling urges, sexual urges or other urges while being treated with SINEMET. Patients should inform their physician if they experience new or increased gambling urges, increased sexual urges, or other intense urges while taking SINEMET. Physicians should consider dose reduction or stopping the medication if a patient develops such urges while taking SINEMET (See PRECAUTIONS, Impulse Control / Compulsive Behaviors).

Laboratory Tests

Abnormalities in laboratory tests may include elevations of liver function tests such as alkaline phosphatase, SGOT (AST), SGPT (ALT), lactic dehydrogenase (LDH), and bilirubin. Abnormalities in blood urea nitrogen (BUN) and positive Coombs test have also been reported. Commonly, levels of blood urea nitrogen, creatinine, and uric acid are lower during administration of SINEMET than with levodopa.

SINEMET may cause a false-positive reaction for urinary ketone bodies when a test tape is used for determination of ketonuria. This reaction will not be altered by boiling the urine specimen. False-negative tests may result with the use of glucose-oxidase methods of testing for glucosuria.

Cases of falsely diagnosed pheochromocytoma in patients on carbidopa levodopa therapy have been reported very rarely. Caution should be exercised when interpreting the plasma and urine levels of catecholamines and their metabolites in patients on levodopa or carbidopa levodopa therapy.

Drug Interactions

Caution should be exercised when the following drugs are administered concomitantly with SINEMET.

Symptomatic postural hypotension occurred when SINEMET was added to the treatment of a patient receiving antihypertensive drugs. Therefore, when therapy with SINEMET is started, dosage adjustment of the antihypertensive drug may be required.

For patients receiving MAO inhibitors (Type A or B), see CONTRAINDICATIONS. Concomitant therapy with selegiline and carbidopa levodopa may be associated with severe orthostatic hypotension not attributable to carbidopa levodopa alone (see CONTRAINDICATIONS).

There have been rare reports of adverse reactions, including hypertension and dyskinesia, resulting from the concomitant use of tricyclic antidepressants and SINEMET.

Dopamine D_2 receptor antagonists (e.g., phenothiazines, butyrophenones, risperidone) and isoniazid may reduce the therapeutic effects of levodopa. In addition, the beneficial effects of levodopa in Parkinson's disease have been reported to be reversed by phenytoin and papaverine. Patients taking these drugs with SINEMET should be carefully observed for loss of therapeutic response.

Use of SINEMET with dopamine-depleting agents (e.g., reserpine and tetrabenazine) or other drugs known to deplete monoamine stores is not recommended.

SINEMET and iron salts or multivitamins containing iron salts should be coadministered with caution. Iron salts can form chelates with levodopa and carbidopa and consequently reduce the bioavailability of carbidopa and levodopa.

Although metoclopramide may increase the bioavailability of levodopa by increasing gastric emptying, metoclopramide may also adversely affect disease control by its dopamine receptor antagonistic properties.

Carcinogenesis, Mutagenesis, Impairment of Fertility

In a two-year bioassay of SINEMET, no evidence of carcinogenicity was found in rats receiving doses of approximately two times the maximum daily human dose of carbidopa and four times the maximum daily human dose of levodopa.

In reproduction studies with SINEMET, no effects on fertility were found in rats receiving doses of approximately two times the maximum daily human dose of carbidopa and four times the maximum daily human dose of levodopa.

Pregnancy

No teratogenic effects were observed in a study in mice receiving up to 20 times the maximum recommended human dose of SINEMET. There was a decrease in the number of live pups delivered by rats receiving approximately two times the maximum recommended human dose of carbidopa and approximately five times the maximum recommended human dose of levodopa during organogenesis. SINEMET caused both visceral and skeletal malformations in rabbits at all doses and ratios of carbidopa/levodopa tested, which ranged from 10 times/5 times the maximum recommended human dose of carbidopa/levodopa to 20 times/10 times the maximum recommended human dose of carbidopa/levodopa.

There are no adequate or well-controlled studies in pregnant women. It has been reported from individual cases that levodopa crosses the human placental barrier, enters the fetus, and is metabolized. Carbidopa concentrations in fetal tissue appeared to be minimal. Use of SINEMET in women of childbearing potential requires that the anticipated benefits of the drug be weighed against possible hazards to mother and child.

Nursing Mothers

Levodopa has been detected in human milk. Caution should be exercised when SINEMET is administered to a nursing woman.

Pediatric Use

Safety and effectiveness in pediatric patients have not been established. Use of the drug in patients below the age of 18 is not recommended.

Geriatric Use

In the clinical efficacy trials for SINEMET, almost half of the patients were older than 65, but few were older than 75. No overall meaningful differences in safety or effectiveness were observed between

these subjects and younger subjects, but greater sensitivity of some older individuals to adverse drug reactions such as hallucinations cannot be ruled out. There is no specific dosing recommendation based upon clinical pharmacology data as SINEMET is titrated as tolerated for clinical effect.

ADVERSE REACTIONS

The most common adverse reactions reported with SINEMET have included dyskinesias, such as choreiform, dystonic, and other involuntary movements, and nausea.

The following other adverse reactions have been reported with SINEMET:

Body as a Whole

Chest pain, asthenia.

Cardiovascular

Cardiac irregularities, hypotension, orthostatic effects including orthostatic hypotension, hypertension, syncope, phlebitis, palpitation.

Gastrointestinal

Dark saliva, gastrointestinal bleeding, development of duodenal ulcer, anorexia, vomiting, diarrhea, constipation, dyspepsia, dry mouth, taste alterations.

Hematologic

Agranulocytosis, hemolytic and non-hemolytic anemia, thrombocytopenia, leukopenia.

Hypersensitivity

Angioedema, urticaria, pruritus, Henoch-Schönlein purpura, bullous lesions (including pemphigus-like reactions).

Musculoskeletal

Back pain, shoulder pain, muscle cramps.

Nervous System/Psychiatric

Psychotic episodes including delusions, hallucinations, and paranoid ideation, bradykinetic episodes ("on-off" phenomenon), confusion, agitation, dizziness, somnolence, dream abnormalities including nightmares, insomnia, paresthesia, headache, depression with or without development of suicidal tendencies, dementia, pathological gambling, increased libido including hypersexuality, impulse control symptoms. Convulsions also have occurred; however, a causal relationship with SINEMET has not been established.

Respiratory

Dyspnea, upper respiratory infection.

Skin

Rash, increased sweating, alopecia, dark sweat.

Urogenital

Urinary tract infection, urinary frequency, dark urine.

Laboratory Tests

Decreased hemoglobin and hematocrit; abnormalities in alkaline phosphatase, SGOT (AST), SGPT (ALT), LDH, bilirubin, BUN, Coombs test; elevated serum glucose; white blood cells, bacteria, and blood in the urine.

Other adverse reactions that have been reported with levodopa alone and with various carbidopa

levodopa formulations, and may occur with SINEMET are:

Body as a Whole

Abdominal pain and distress, fatigue.

Cardiovascular

Myocardial infarction.

Gastrointestinal

Gastrointestinal pain, dysphagia, sialorrhea, flatulence, bruxism, burning sensation of the tongue, heartburn, hiccups.

Metabolic

Edema, weight gain, weight loss.

Musculoskeletal

Leg pain.

Nervous System/Psychiatric

Ataxia, extrapyramidal disorder, falling, anxiety, gait abnormalities, nervousness, decreased mental acuity, memory impairment, disorientation, euphoria, blepharospasm (which may be taken as an early sign of excess dosage; consideration of dosage reduction may be made at this time), trismus, increased tremor, numbness, muscle twitching, activation of latent Horner's syndrome, peripheral neuropathy.

Respiratory

Pharyngeal pain, cough.

Skin

Malignant melanoma, flushing.

Special Senses

Oculogyric crises, diplopia, blurred vision, dilated pupils.

Urogenital

Urinary retention, urinary incontinence, priapism.

Miscellaneous

Bizarre breathing patterns, faintness, hoarseness, malaise, hot flashes, sense of stimulation.

Laboratory Tests

Decreased white blood cell count and serum potassium; increased serum creatinine and uric acid; protein and glucose in urine.

OVERDOSAGE

Management of acute overdosage with SINEMET is the same as management of acute overdosage with levodopa. Pyridoxine is not effective in reversing the actions of SINEMET.

General supportive measures should be employed, along with immediate gastric lavage. Intravenous fluids should be administered judiciously and an adequate airway maintained. Electrocardiographic monitoring should be instituted and the patient carefully observed for the development of arrhythmias; if required, appropriate antiarrhythmic therapy should be given. The possibility that the patient may have taken other drugs as well as SINEMET should be taken into consideration. To date, no experience has been reported with dialysis; hence, its value in overdosage is not known.

Based on studies in which high doses of levodopa and/or carbidopa were administered, a significant proportion of rats and mice given single oral doses of levodopa of approximately 1500-2000 mg/kg are expected to die. A significant proportion of infant rats of both sexes are expected to die at a dose of 800 mg/kg. A significant proportion of rats are expected to die after treatment with similar doses of carbidopa. The addition of carbidopa in a 1:10 ratio with levodopa increases the dose at which a significant proportion of mice are expected to die to 3360 mg/kg.

DOSAGE AND ADMINISTRATION

The optimum daily dosage of SINEMET must be determined by careful titration in each patient. SINEMET tablets are available in a 1:4 ratio of carbidopa to levodopa (SINEMET 25-100) as well as 1:10 ratio (SINEMET 25-250 and SINEMET 10-100). Tablets of the two ratios may be given separately or combined as needed to provide the optimum dosage.

Studies show that peripheral dopa decarboxylase is saturated by carbidopa at approximately 70 to 100 mg a day. Patients receiving less than this amount of carbidopa are more likely to experience nausea and vomiting.

Usual Initial Dosage

Dosage is best initiated with one tablet of SINEMET 25-100 three times a day. This dosage schedule provides 75 mg of carbidopa per day. Dosage may be increased by one tablet every day or every other day, as necessary, until a dosage of eight tablets of SINEMET 25-100 a day is reached.

If SINEMET 10-100 is used, dosage may be initiated with one tablet three or four times a day. However, this will not provide an adequate amount of carbidopa for many patients. Dosage may be increased by one tablet every day or every other day until a total of eight tablets (2 tablets q.i.d.) is reached.

How to Transfer Patients from Levodopa

Levodopa must be discontinued at least twelve hours before starting SINEMET. A daily dosage of SINEMET should be chosen that will provide approximately 25% of the previous levodopa dosage. Patients who are taking less than 1500 mg of levodopa a day should be started on one tablet of SINEMET 25-100 three or four times a day. The suggested starting dosage for most patients taking more than 1500 mg of levodopa is one tablet of SINEMET 25-250 three or four times a day.

Maintenance

Therapy should be individualized and adjusted according to the desired therapeutic response. At least 70 to 100 mg of carbidopa per day should be provided. When a greater proportion of carbidopa is required, one tablet of SINEMET 25-100 may be substituted for each tablet of SINEMET 10-100. When more levodopa is required, SINEMET 25-250 should be substituted for SINEMET 25-100 or SINEMET 10-100. If necessary, the dosage of carbidopa levodopa 25-250 may be increased by one-half or one tablet every day or every other day to a maximum of eight tablets a day. Experience with total daily dosages of carbidopa greater than 200 mg is limited.

Because both therapeutic and adverse responses occur more rapidly with SINEMET than with levodopa alone, patients should be monitored closely during the dose adjustment period. Specifically, involuntary movements will occur more rapidly with SINEMET than with levodopa. The occurrence of involuntary movements may require dosage reduction. Blepharospasm may be a useful early sign of excess dosage in some patients.

Addition of Other Antiparkinsonian Medications

Standard drugs for Parkinson's disease, other than levodopa without a decarboxylase inhibitor, may be used concomitantly while SINEMET is being administered, although dosage adjustments may be

required.

Interruption of Therapy

Sporadic cases of hyperpyrexia and confusion have been associated with dose reductions and withdrawal of SINEMET. Patients should be observed carefully if abrupt reduction or discontinuation of SINEMET is required, especially if the patient is receiving neuroleptics. (See WARNINGS.)

If general anesthesia is required, SINEMET may be continued as long as the patient is permitted to take fluids and medication by mouth. If therapy is interrupted temporarily, the patient should be observed for symptoms resembling NMS, and the usual daily dosage may be administered as soon as the patient is able to take oral medication.

HOW SUPPLIED

No. 3916A — SINEMET 25-100 Tablets are yellow, round, uncoated tablets, that are coded "650" on one side and plain on the other. They are supplied as follows:

NDC 0006-3916-68 bottles of 100.

No. 3915 — SINEMET 10-100 Tablets are light dapple-blue, round, uncoated tablets, that are coded "647" on one side and plain on the other. They are supplied as follows:

NDC 0006-3915-68 bottles of 100.

No. 3917 — SINEMET 25-250 Tablets are light dapple-blue, round, uncoated tablets, that are coded "654" on one side and plain on the other. They are supplied as follows:

NDC 0006-3917-68 bottles of 100.

Storage and Handling

Store at 25°C (77°F), excursions permitted to 15-30°C (59-86°F) [see USP Controlled Room Temperature]. Store in a tightly closed container, protected from light and moisture.

Dispense in a tightly closed, light-resistant container.

Manufactured for: Merck Sharp & Dohme Corp., a subsidiary of
MERCK & CO., INC., Whitehouse Station, NJ 08889, USA

Manufactured by:
Mylan Pharmaceuticals, Inc.
Morgantown, WV 26505, USA

For patent information: www.merck.com/product/patent/home.html

Copyright © 1996-2018 Merck Sharp & Dohme Corp., a subsidiary of **Merck & Co., Inc.**
All rights reserved.

Revised: 04/2018

uspi-mk0295b-t-1804r004

Rx Only

APPENDIX B. INSTRUCTIONS FOR DOSE CONVERSION TO IPX203

The goal of the dose conversion period is to establish a dosing regimen for IPX203 that minimizes “Off” time without causing troublesome dyskinesias.

The initial dose of IPX203 is based on the subject’s most frequent IR CD-LD dose established during the 3-week IR CD-LD dose adjustment period.

Table B-1 Recommended Starting IPX203 LD Dosing Regimen Based on the Dosing Regimen of IR CD-LD at the End of the Dose Adjustment Period

Most Frequent IR CD-LD Unit Dose (mg)	Recommended Starting IPX203 Daily Dosing Regimen CD-LD (mg) Every 8 Hours
25-100[a]	70-280 mg (2 × 35-140 mg)
>25-100 – 37.5-150	105-420 mg (3 × 35-140 mg)
>37.5-150 – 50-200	140-560 mg (4 × 35-140 mg)
>50-200	175-700 mg (5 × 35-140 mg)

[a] Subjects who are on a total daily dose of less than 125-500 mg CD-LD from IR CD-LD should be advised to initially take IPX203 every 12 hours. The dosing interval may be reduced to approximately every 8 hours if the subject does not achieve an acceptable duration of effect.

Conversion Instructions:

1. Convert the subject’s most frequent daily dose of IR CD-LD to the corresponding dose of IPX203 according to the above table. It is recommended that the subject takes IPX203 doses approximately every 8 hours apart (for example, a subject may take IPX203 at 6 AM, 2 PM, and 10 PM). Some subjects may benefit from a shorter or longer dosing interval. The dosing interval may vary but should not be more frequent than every 6 hours. The maximum recommended daily dose of IPX203 is 600-2400 mg CD-LD.
2. Subjects who are on a total daily dose of less than 125-500 mg CD-LD from IR CD-LD should be advised to initially take IPX203 every 12 hours. The dosing interval may be reduced to approximately every 8 hours if the subject does not achieve an acceptable duration of effect.
3. The Investigator or their staff are advised to be in telephone contact with the subject, especially during the initial dose conversion to assess the need for dosage adjustment with the goal of minimizing “Off” time without causing troublesome dyskinesia or other dopaminergic side effects. Calls to the subject can be reduced appropriately when the subject reaches a stable dosing regimen.
4. If dose adjustment is necessary, consider the following options recognizing that the number of capsules at each dose may be varied to achieve an optimal response.

   a. If turning "On" is slow following the first morning dose, consider taking the morning IPX203 dose in the fasted state and/or increasing the dose by one capsule (35-140 mg IPX203 CD-LD).
   b. If turning "On" is slow later in the day or to reduce "end-of-dose" "Off" time, consider increasing the dose by one capsule (35-140 mg IPX203 CD-LD) before reducing the dosing interval.

5. In case of troublesome dyskinesias, use the following guidelines:
   a. Consider reducing the dose by one capsule (35-140 mg IPX203 CD-LD).
   b. Consider increasing the dosing interval.
6. The subject must be on a stable dosing regimen of IPX203 (no change in dose or in dosing frequency) for at least 5 days prior to Visit 4 (randomization).

APPENDIX C. UNITED KINGDOM PARKINSON'S DISEASE SOCIETY BRAIN BANK DIAGNOSTIC CRITERIA FOR PARKINSON'S DISEASE

Step 1: Diagnosis of Parkinsonism

Bradykinesia **and** at least one of the following:

- Muscular rigidity
- 4–6 Hz resting tremor
- postural instability not caused by primary visual, vestibular, cerebellar or Proprioceptive dysfunction

Step 2: Features tending to exclude Parkinson's disease as the cause of Parkinsonism

- History of repeated strokes with stepwise progression of parkinsonian features
- History of repeated head injury
- History of definite encephalitis
- Neuroleptic treatment at onset of symptoms
- >1 affected relatives
- Sustained remission
- Strictly unilateral features after 3 years
- Supranuclear gaze palsy
- Cerebellar signs
- Early severe autonomic involvement
- Early severe dementia with disturbances of memory, language and praxis
- Babinski's sign
- Presence of a cerebral tumour or communicating hydrocephalus on computed tomography scan
- Negative response to large doses of levodopa (if malabsorption excluded)
- MPTP exposure

Step 3: Features that support a diagnosis of Parkinson's disease (three or more required for diagnosis of definite Parkinson's disease)

- Unilateral onset
- Rest tremor present
- Progressive disorder
- Persistent asymmetry affecting the side of onset most
- Excellent (70–100%) response to levodopa
- Severe levodopa-induced chorea
- Levodopa response for ≥5 years
- Clinical course of ≥10 years

APPENDIX D. MONTREAL COGNITIVE ASSESSMENT (MOCA)

Montreal Cognitive Assessment
(MoCA)

Administration and Scoring Instructions

The Montreal Cognitive Assessment (MoCA) was designed as a rapid screening instrument for mild cognitive dysfunction. It assesses different cognitive domains: attention and concentration, executive functions, memory, language, visuoconstructional skills, conceptual thinking, calculations, and orientation. Time to administer the MoCA is approximately 10 minutes. The total possible score is 30 points; a score of 26 or above is considered normal.

1. Alternating Trail Making:

Administration: The examiner instructs the subject: *"Please draw a line, going from a number to a letter in ascending order. Begin here* [point to (1)] *and draw a line from 1 then to A then to 2 and so on. End here* [point to (E)]."

Scoring: Allocate one point if the subject successfully draws the following pattern:
1 –A- 2- B- 3- C- 4- D- 5- E, without drawing any lines that cross. Any error that is not immediately self-corrected earns a score of 0.

2. Visuoconstructional Skills (Cube):

Administration: The examiner gives the following instructions, pointing to the **cube**: "*Copy this drawing as accurately as you can, in the space below*".

Scoring: One point is allocated for a correctly executed drawing.
• Drawing must be three-dimensional
• All lines are drawn
• No line is added
• Lines are relatively parallel and their length is similar (rectangular prisms are accepted)

A point is not assigned if any of the above-criteria are not met.

3. Visuoconstructional Skills (Clock):

Administration: Indicate the right third of the space and give the following instructions: *"Draw a clock. Put in all the numbers and set the time to 10 past 11".*

Scoring: One point is allocated for each of the following three criteria:
• Contour (1 pt.): the clock face must be a circle with only minor distortion acceptable (e.g., slight imperfection on closing the circle);
• Numbers (1 pt.): all clock numbers must be present with no additional numbers; numbers must be in the correct order and placed in the approximate quadrants on the clock face; Roman numerals are acceptable; numbers can be placed outside the circle contour;
• Hands (1 pt.): there must be two hands jointly indicating the correct time; the hour hand must be clearly shorter than the minute hand; hands must be centred within the clock face with their junction close to the clock centre.

A point is not assigned for a given element if any of the above-criteria are not met.

© Z. Nasreddine MD

4. Naming:

Administration: Beginning on the left, point to each figure and say: *"Tell me the name of this animal".*

Scoring: One point each is given for the following responses: (1) lion (2) rhinoceros or rhino (3) camel or dromedary.

5. Memory:

Administration: The examiner reads a list of 5 words at a rate of one per second, giving the following instructions: *"This is a memory test. I am going to read a list of words that you will have to remember now and later on. Listen carefully. When I am through, tell me as many words as you can remember. It doesn't matter in what order you say them".* Mark a check in the allocated space for each word the subject produces on this first trial. When the subject indicates that (s)he has finished (has recalled all words), or can recall no more words, read the list a second time with the following instructions: *"I am going to read the same list for a second time. Try to remember and tell me as many words as you can, including words you said the first time."* Put a check in the allocated space for each word the subject recalls after the second trial.
At the end of the second trial, inform the subject that (s)he will be asked to recall these words again by saying, *"I will ask you to recall those words again at the end of the test."*

Scoring: No points are given for Trials One and Two.

6. Attention:

Forward Digit Span: Administration: Give the following instruction: "*I am going to say some numbers and when I am through, repeat them to me exactly as I said them*". Read the five number sequence at a rate of one digit per second.

Backward Digit Span: Administration: Give the following instruction: "*Now I am going to say some more numbers, but when I am through you must repeat them to me in the backwards order.*" Read the three number sequence at a rate of one digit per second.

Scoring: Allocate one point for each sequence correctly repeated, (*N.B.*: the correct response for the backwards trial is 2-4-7).

Vigilance: Administration: The examiner reads the list of letters at a rate of one per second, after giving the following instruction: "*I am going to read a sequence of letters. Every time I say the letter A, tap your hand once. If I say a different letter, do not tap your hand*".

Scoring: Give one point if there is zero to one errors (an error is a tap on a wrong letter or a failure to tap on letter A).

Serial 7s: Administration: The examiner gives the following instruction: *"Now, I will ask you to count by subtracting seven from 100, and then, keep subtracting seven from your answer until I tell you to stop."* Give this instruction twice if necessary.

Scoring: This item is scored out of 3 points. Give no (0) points for no correct subtractions, 1 point for one correction subtraction, 2 points for two-to-three correct subtractions, and 3 points if the participant successfully makes four or five correct subtractions. Count each correct subtraction of 7 beginning at 100. Each subtraction is evaluated independently; that is, if the participant responds with an incorrect number but continues to correctly subtract 7 from it, give a point for each correct subtraction. For example, a participant may respond "92 – 85 – 78 – 71 – 64" where the "92" is incorrect, but all subsequent numbers are subtracted correctly. This is one error and the item would be given a score of 3.

7. **Sentence repetition:**

Administration: The examiner gives the following instructions: *"I am going to read you a sentence. Repeat it after me, exactly as I say it* [pause]: ***I only know that John is the one to help today."*** Following the response, say: *"Now I am going to read you another sentence. Repeat it after me, exactly as I say it* [pause]: ***The cat always hid under the couch when dogs were in the room."***

Scoring: Allocate 1 point for each sentence correctly repeated. Repetition must be exact. Be alert for errors that are omissions (e.g., omitting "only", "always") and substitutions/additions (e.g., "John is the one who helped today;" substituting "hides" for "hid", altering plurals, etc.).

8. **Verbal fluency:**

Administration: The examiner gives the following instruction: *"Tell me as many words as you can think of that begin with a certain letter of the alphabet that I will tell you in a moment. You can say any kind of word you want, except for proper nouns (like Bob or Boston), numbers, or words that begin with the same sound but have a different suffix, for example, love, lover, loving. I will tell you to stop after one minute. Are you ready?* [Pause] *Now, tell me as many words as you can think of that begin with the letter F.* [time for 60 sec]. *Stop."*

Scoring: Allocate one point if the subject generates 11 words or more in 60 sec. Record the subject's response in the bottom or side margins.

9. **Abstraction:**

Administration: The examiner asks the subject to explain what each pair of words has in common, starting with the example: *"Tell me how an orange and a banana are alike".* If the subject answers in a concrete manner, then say only one additional time: *"Tell me another way in which those items are alike".* If the subject does not give the appropriate response *(fruit)*, say, *"Yes, and they are also both fruit."* Do not give any additional instructions or clarification. After the practice trial, say: *"Now, tell me how a train and a bicycle are alike".* Following the response, administer the second trial, saying: *"Now tell me how a ruler and a watch are alike".* Do not give any additional instructions or prompts.

Scoring: Only the last two item pairs are scored. Give 1 point to each item pair correctly answered. The following responses are acceptable:
Train-bicycle = means of transportation, means of travelling, you take trips in both;
Ruler-watch = measuring instruments, used to measure.
The following responses are **not** acceptable: Train-bicycle = they have wheels; Ruler-watch = they have numbers.

10. Delayed recall:

Administration: The examiner gives the following instruction: *"I read some words to you earlier, which I asked you to remember. Tell me as many of those words as you can remember."* Make a check mark (√) for each of the words correctly recalled spontaneously without any cues, in the allocated space.

Scoring: **Allocate 1 point for each word recalled freely without any cues.**

Optional:
Following the delayed free recall trial, prompt the subject with the semantic category cue provided below for any word not recalled. Make a check mark (√) in the allocated space if the subject remembered the word with the help of a category or multiple-choice cue. Prompt all non-recalled words in this manner. If the subject does not recall the word after the category cue, give him/her a multiple choice trial, using the following example instruction, *"Which of the following words do you think it was, NOSE, FACE, or HAND?"*
Use the following category and/or multiple-choice cues for each word, when appropriate:

FACE:	category cue: part of the body	multiple choice: nose, face, hand
VELVET:	category cue: type of fabric	multiple choice: denim, cotton, velvet
CHURCH:	category cue: type of building	multiple choice: church, school, hospital
DAISY:	category cue: type of flower	multiple choice: rose, daisy, tulip
RED:	category cue: a colour	multiple choice: red, blue, green

Scoring: **No points are allocated for words recalled with a cue.** A cue is used for clinical information purposes only and can give the test interpreter additional information about the type of memory disorder. For memory deficits due to retrieval failures, performance can be improved with a cue. For memory deficits due to encoding failures, performance does not improve with a cue.

11. Orientation:

Administration: The examiner gives the following instructions: "Tell me the date today". If the subject does not give a complete answer, then prompt accordingly by saying: *"Tell me the [year, month, exact date, and day of the week]."* Then say: *"Now, tell me the name of this place, and which city it is in."*

Scoring: Give one point for each item correctly answered. The subject must tell the exact date and the exact place (name of hospital, clinic, office). No points are allocated if subject makes an error of one day for the day and date.

TOTAL SCORE: Sum all subscores listed on the right-hand side. Add one point for an individual who has 12 years or fewer of formal education, for a possible maximum of 30 points. A final total score of 26 and above is considered normal.

© Z. Nasreddine MD
www.mocatest.org

MONTREAL COGNITIVE ASSESSMENT (MOCA)

Version 7.1 Original Version

NAME :
Education :
Sex :
Date of birth :
DATE :

VISUOSPATIAL / EXECUTIVE

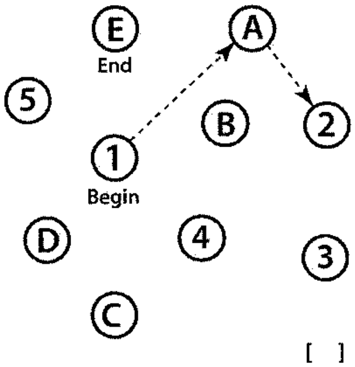

[ ]

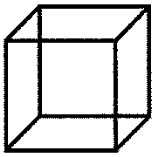

Copy cube

[ ]

Draw CLOCK (Ten past eleven)
(3 points)

[ ] Contour [ ] Numbers [ ] Hands

POINTS: ___/5

NAMING

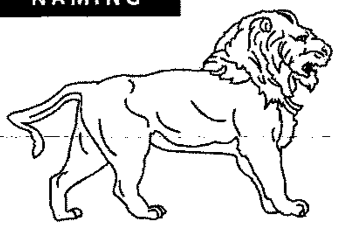

[ ]

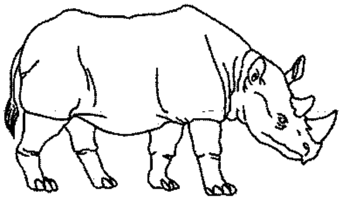

[ ]

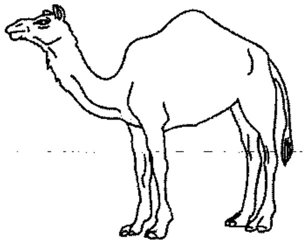

[ ]

___/3

MEMORY

Read list of words, subject must repeat them. Do 2 trials, even if 1st trial is successful. Do a recall after 5 minutes.

	FACE	VELVET	CHURCH	DAISY	RED
1st trial					
2nd trial					

No points

ATTENTION

Read list of digits (1 digit/ sec.).
Subject has to repeat them in the forward order [ ] 2 1 8 5 4
Subject has to repeat them in the backward order [ ] 7 4 2
___/2

Read list of letters. The subject must tap with his hand at each letter A. No points if ≥ 2 errors
[ ] F B A C M N A A J K L B A F A K D E A A A J A M O F A A B
___/1

Serial 7 subtraction starting at 100 [ ] 93 [ ] 86 [ ] 79 [ ] 72 [ ] 65
4 or 5 correct subtractions: **3 pts**, 2 or 3 correct: **2 pts**, 1 correct: **1 pt**, 0 correct: **0 pt**
___/3

LANGUAGE

Repeat : I only know that John is the one to help today. [ ]
The cat always hid under the couch when dogs were in the room. [ ]
___/2

Fluency / Name maximum number of words in one minute that begin with the letter F [ ] _____ (N ≥ 11 words)
___/1

ABSTRACTION

Similarity between e.g. banana - orange = fruit [ ] train – bicycle [ ] watch - ruler
___/2

DELAYED RECALL

	FACE	VELVET	CHURCH	DAISY	RED	
Has to recall words **WITH NO CUE**	[ ]	[ ]	[ ]	[ ]	[ ]	Points for UNCUED recall only
**Optional** Category cue						
Multiple choice cue						

___/5

ORIENTATION

[ ] Date [ ] Month [ ] Year [ ] Day [ ] Place [ ] City
___/6

© Z.Nasreddine MD
www.mocatest.org
Normal ≥26 / 30
TOTAL ___/30
Add 1 point if ≤ 12 yr edu

Administered by:____________________________

APPENDIX E. MOVEMENT DISORDERS SOCIETY VERSION OF UNIFIED PARKINSON'S DISEASE RATING SCALE (MDS-UPDRS)

START TIME ___:___ *(hh:mm, 24-hr clock)*

MDS-UPDRS

Given formatting concerns, this MDS-UPDRS source document does not track the Patient ID on each page of the assessment. DO NOT remove the staple binding this MDS-UPDRS packet.

July 1, 2008

Copyright © 2008 International Parkinson and Movement Disorder Society. All rights reserved
This scale may not be copied, distributed or otherwise used in whole or in part without prior written consent of the International Parkinson and Movement Disorder Society

MDS-UPDRS Permissions

Permission is required to use the MDS-developed Rating Scales (with the exception of personal/individual use). Reproduction, translation, modification, sale, or distribution of any portion of the MDS Rating Scales is strictly prohibited. MDS Rating Scales may not be incorporated into clinical trials, training or certification programs or materials, software programs, or otherwise except through use of the Permissions Request Form and payment of applicable fees.

Continue to p. 3 to view the MDS-UPDRS

MDS-UPDRS

The *Movement* Disorder Society (MDS)-sponsored new version of the UPDRS is founded on the critique that was formulated by the Task Force for Rating Scales in Parkinson's disease (*Mov Disord* 2003;18:738-750). Thereafter, the MDS recruited a Chairperson to organize a program to provide the Movement Disorder community with a new version of the UPDRS that would maintain the overall format of the original UPDRS, but address issues identified in the critique as weaknesses and ambiguities. The Chairperson identified subcommittees with chairs and members. Each part was written by the appropriate subcommittee members and then reviewed and ratified by the entire group. These members are listed below.

The MDS-UPDRS has four parts: Part I (non-motor experiences of daily living), Part II (motor experiences of daily living), Part III (motor examination) and Part IV (motor complications). Part I has two components: IA concerns a number of behaviors that are assessed by the investigator with all pertinent information from patients and caregivers, and IB is completed by the patient with or without the aid of the caregiver, but independently of the investigator. These sections can, however, be reviewed by the rater to ensure that all questions are answered clearly and the rater can help explain any perceived ambiguities. Part II is designed to be a self-administered questionnaire like Part IB, but can be reviewed by the investigator to ensure completeness and clarity. Of note, the official versions of Part IA, Part IB and Part II of the MDS-UPDRS do not have separate on or off ratings. However, for individual programs or protocols the same questions can be used separately for on and off. Part III has instructions for the rater to give or demonstrate to the patient; it is completed by the rater. Part IV has instructions for the rater and also instructions to be read to the patient. This part integrates patient-derived information with the rater's clinical observations and judgments and is completed by the rater.

The authors of this new version are:

Chairperson: Christopher G. Goetz

Part I: Werner Poewe (chair), Bruno Dubois, Anette Schrag
Part II: Matthew B. Stern (chair), Anthony E. Lang, Peter A. LeWitt
Part III: Stanley Fahn (chair), Joseph Jankovic, C. Warren Olanow
Part IV: Pablo Martinez-Martin (chair), Andrew Lees, Olivier Rascol, Bob van Hilten
Development Standards: Glenn T. Stebbins (chair), Robert Holloway, David Nyenhuis
Appendices: Cristina Sampaio (chair), Richard Dodel, Jaime Kulisevsky
Statistical Testing: Barbara Tilley (chair), Sue Leurgans, Jean Teresi,
Consultant: Stephanie Shaftman, Nancy LaPelle

Contact person: Christopher G. Goetz, MD
Rush University Medical Center
1725 W. Harrison Street, Suite 755
Chicago, IL USA 60612

Telephone 312-942-8016
Email: cgoetz@rush.edu

July 1, 2008

Copyright © 2008 International Parkinson and Movement Disorder Society. All rights reserved.

This scale may not be copied, distributed or otherwise used in whole or in part without prior written consent of the International Parkinson and Movement Disorder Society

Part I: Non-Motor Aspects of Experiences of Daily Living (nM-EDL)

Overview: This portion of the scale assesses the non-motor impact of Parkinson's disease (PD) on patients' experiences of daily living. There are 13 questions. Part 1A is administered by the rater (six questions) and focuses on complex behaviors. Part 1B is a component of the self-administered Patient Questionnaire that covers seven questions on non-motor experiences of daily living.

Part 1A:
In administering Part IA, the examiner should use the following guidelines:

1. Mark at the top of the form the primary data source as patient, caregiver, or patient and caregiver in equal proportion.
2. The response to each item should refer to a period encompassing the prior week including the day on which the information is collected.
3. All items must have an integer rating (no half points, no missing scores). In the event that an item does not apply or cannot be rated (e.g., amputee who cannot walk), the item is marked UR for Unable to Rate.
4. The answers should reflect the usual level of function and words such as "usually", "generally", "most of the time" can be used with patients.
5. Each question has a text for you to read (Instructions to patients/caregiver). After that statement, you can elaborate and probe based on the target symptoms outlined in the Instructions to examiner. You should NOT READ the RATING OPTIONS to the patient/caregiver, because these are written in medical terminology. From the interview and probing, you will use your medical judgment to arrive at the best response.
6. Patients may have co-morbidities and other medical conditions that can affect their function. You and the patient must rate the problem as it exists and do not attempt to separate elements due to Parkinson's disease from other conditions.

EXAMPLE OF NAVIGATING THROUGH THE RESPONSE OPTIONS FOR PART 1A

Suggested strategies for obtaining the most accurate answer:
After reading the instructions to the patient, you will need to probe the entire domain under discussion to determine Normal vs. problematic: If your questions do not identify any problem in this domain, record 0 and move on to the next question.

If your questions identify a problem in this domain, you should work next with a reference anchor at the mid-range (option 2 or Mild) to find out if the patient functions at this level, better or worse. <u>You will not be reading the choices of responses to the patient as the responses use clinical terminology. You will be asking enough probing questions to determine the response that should be coded.</u>

Work up and down the options with the patient to identify the most accurate response, giving a final check by excluding the options above and below the selected response.

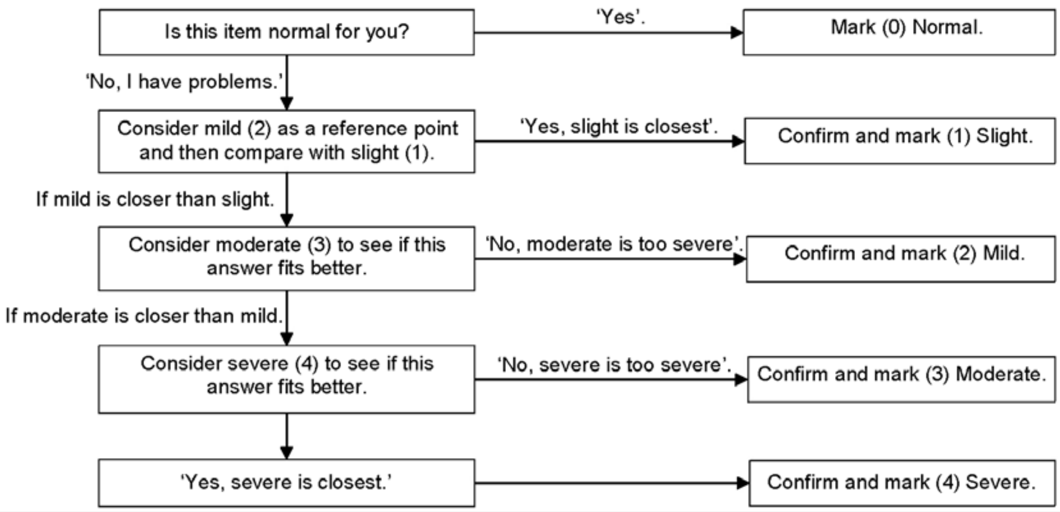

July 1, 2008 *Copyright © 2008 International Parkinson and Movement Disorder Society. All rights reserved* Page 3
This scale may not be copied, distributed or otherwise used in whole or in part without prior written consent of the International Parkinson and Movement Disorder Society

MDS UPDRS
Part I: Non-Motor Aspects of Experiences of Daily Living (nM-EDL)

Part 1A: Complex behaviors: [completed by rater]

Primary source of information:

☐ Patient ☐ Caregiver ☐ Patient and Caregiver in Equal Proportion

To be read to the patient: I am going to ask you six questions about behaviors that you may or may not experience. Some questions concern common problems and some concern uncommon ones. If you have a problem in one of the areas, please choose the best response that describes how you have felt MOST OF THE TIME during the PAST WEEK. If you are not bothered by a problem, you can simply respond NO. I am trying to be thorough, so I may ask questions that have nothing to do with you.

1.1 COGNITIVE IMPAIRMENT	SCORE
Instructions to examiner: Consider all types of altered level of cognitive function including cognitive slowing, impaired reasoning, memory loss, deficits in attention and orientation. Rate their impact on activities of daily living as perceived by the patient and/or caregiver.	
Instructions to patients [and caregiver]: Over the past week have you had problems remembering things, following conversations, paying attention, thinking clearly, or finding your way around the house or in town? [If yes, examiner asks patient or caregiver to elaborate and probes for information.]	
0: Normal: No cognitive impairment.	
1: Slight: Impairment appreciated by patient or caregiver with no concrete interference with the patient's ability to carry out normal activities and social interactions.	☐
2: Mild: Clinically evident cognitive dysfunction, but only minimal interference with the patient's ability to carry out normal activities and social interactions.	
3: Moderate: Cognitive deficits interfere with but do not preclude the patient's ability to carry out normal activities and social interactions.	
4: Severe: Cognitive dysfunction precludes the patient's ability to carry out normal activities and social interactions.	

July 1, 2008 *Copyright © 2008 International Parkinson and Movement Disorder Society. All rights reserved.* Page 4
This scale may not be copied, distributed or otherwise used in whole or in part without prior written consent of the International Parkinson and Movement Disorder Society

	SCORE
**1.2 HALLUCINATIONS AND PSYCHOSIS**<br><br>Instructions to examiner: Consider both illusions (misinterpretations of real stimuli) and hallucinations (spontaneous false sensations). Consider all major sensory domains (visual, auditory, tactile, olfactory and gustatory). Determine presence of unformed (for example sense of presence or fleeting false impressions) as well as formed (fully developed and detailed) sensations. Rate the patient's insight into hallucinations and identify delusions and psychotic thinking.<br><br>*Instructions to patients [and caregiver]: Over the past week have you seen, heard, smelled or felt things that were not really there?* [If yes, examiner asks patient or caregiver to elaborate and probes for information.]<br><br>0: Normal: No hallucinations or psychotic behavior.<br>1: Slight: Illusions or non-formed hallucinations, but patient recognizes them without loss of insight.<br>2: Mild: Formed hallucinations independent of environmental stimuli. No loss of insight.<br>3: Moderate: Formed hallucinations with loss of insight.<br>4: Severe: Patient has delusions or paranoia.	☐
**1.3 DEPRESSED MOOD**<br><br>Instructions to examiner: Consider low mood, sadness, hopelessness, feelings of emptiness or loss of enjoyment. Determine their presence and duration over the past week and rate their interference with the patient's ability to carry out daily routines and engage in social interactions.<br><br>*Instruction to the patient (and caregiver): Over the past week have you felt low, sad, hopeless or unable to enjoy things? If yes, was this feeling for longer than one day at a time? Did it make it difficult for you carry out your usual activities or to be with people?* [If yes, examiner asks patient or caregiver to elaborate and probes for information.]<br><br>0: Normal: No depressed mood.<br>1: Slight: Episodes of depressed mood that are not sustained for more than one day at a time. No interference with patient's ability to carry out normal activities and social interactions.<br>2: Mild: Depressed mood that is sustained over days, but without interference with normal activities and social interactions.<br>3: Moderate: Depressed mood that interferes with, but does not preclude, the patient's ability to carry out normal activities and social interactions.<br>4: Severe: Depressed mood precludes patient's ability to carry out normal activities and social interactions.	☐

Copyright © 2008 International Parkinson and Movement Disorder Society. All rights reserved.

This scale may not be copied, distributed or otherwise used in whole or in part without prior written consent of the International Parkinson and Movement Disorder Society

	SCORE
**1.4 ANXIOUS MOOD**<br><br><u>Instructions to examiner</u>: Determine nervous, tense, worried or anxious feelings (including panic attacks) over the past week and rate their duration and interference with the patient's ability to carry out daily routines and engage in social interactions.<br><br>*<u>Instructions to patients [and caregiver]</u>: Over the past week have you felt nervous, worried or tense? If yes, was this feeling for longer than one day at a time? Did it make it difficult for you to follow your usual activities or to be with other people*? [If yes, examiner asks patient or caregiver to elaborate and probes for information.]<br><br>0: Normal: No anxious feelings.<br><br>1: Slight: Anxious feelings present but not sustained for more than one day at a time. No interference with patient's ability to carry out normal activities and social interactions.<br><br>2: Mild: Anxious feelings are sustained over more than one day at a time, but without interference with patient's ability to carry out normal activities and social interactions.<br><br>3: Moderate: Anxious feelings interfere with, but do not preclude, the patient's ability to carry out normal activities and social interactions.<br><br>4: Severe: Anxious feelings preclude patient's ability to carry out normal activities and social interactions.	☐
**1.5 APATHY**<br><br><u>Instructions to examiner</u>: Consider level of spontaneous activity, assertiveness, motivation and initiative and rate the impact of reduced levels on performance of daily routines and social interactions. Here the examiner should attempt to distinguish between apathy and similar symptoms that are best explained by depression.<br><br>*<u>Instructions to patients (and caregiver)</u>: Over the past week, have you felt indifferent to doing activities or being with people*? [If yes, examiner asks patient or caregiver to elaborate and probes for information.]<br><br>0: Normal: No apathy.<br><br>1: Slight: Apathy appreciated by patient and/or caregiver, but no interference with daily activities and social interactions.<br><br>2: Mild: Apathy interferes with isolated activities and social interactions.<br><br>3: Moderate: Apathy interferes with most activities and social interactions.<br><br>4: Severe: Passive and withdrawn, complete loss of initiative.	☐

Copyright © 2008 International Parkinson and Movement Disorder Society. All rights reserved.

This scale may not be copied, distributed or otherwise used in whole or in part without prior written consent of the International Parkinson and Movement Disorder Society

**1.6 FEATURES OF DOPAMINE DYSREGULATION SYNDROME**	**SCORE**
Instructions to examiner: Consider involvement in a variety of activities including atypical or excessive gambling (e.g. casinos or lottery tickets), atypical or excessive sexual drive or interests (e.g., unusual interest in pornography, masturbation, sexual demands on partner), other repetitive activities (e.g. hobbies, dismantling objects, sorting or organizing), or taking extra non-prescribed medication for non-physical reasons (i.e., addictive behavior). Rate the impact of such abnormal activities/behaviors on the patient's personal life and on his family and social relations (including need to borrow money or other financial difficulties like withdrawal of credit cards, major family conflicts, lost time from work, or missed meals or sleep because of the activity).<br>*Instructions to patients [and caregiver]: Over the past week, have you had unusually strong urges that are hard to control? Do you feel driven to do or think about something and find it hard to stop?* [Give patient examples such as gambling, cleaning, using the computer, taking extra medicine, obsessing about food or sex, all depending on the patients.]<br><br>0: Normal: No problems present.<br>1: Slight: Problems are present but usually do not cause any difficulties for the patient or family/caregiver.<br>2: Mild: Problems are present and usually cause a few difficulties in the patient's personal and family life.<br>3: Moderate: Problems are present and usually cause a lot of difficulties in the patient's personal and family life.<br>4: Severe: Problems are present and preclude the patient's ability to carry out normal activities or social interactions or to maintain previous standards in personal and family life.	☐

The remaining questions in Part I (Non-motor Experiences of Daily Living) [Sleep, Daytime Sleepiness, Pain and Other Sensation, Urinary Problems, Constipation Problems, Lightheadedness on Standing, and Fatigue] are in the **Patient Questionnaire** along with all questions in Part II [Motor Experiences of Daily Living].

 Copyright © 2008 International Parkinson and Movement Disorder Society. All rights reserved.
This scale may not be copied, distributed or otherwise used in whole or in part without prior written consent of the International Parkinson and Movement Disorder Society

Patient Questionnaire:

Instructions:

This questionnaire will ask you about your experiences of daily living.

There are 20 questions. We are trying to be thorough, and some of these questions may therefore not apply to you now or ever. If you do not have the problem, simply mark 0 for NO.

Please read each one carefully and read all answers before selecting the one that best applies to you.

We are interested in your average or usual function over the past week including today. Some patients can do things better at one time of the day than at others. However, only one answer is allowed for each question, so please mark the answer that best describes what you can do most of the time.

You may have other medical conditions besides Parkinson's disease. Do not worry about separating Parkinson's disease from other conditions. Just answer the question with your best response.

Use only 0, 1, 2, 3, 4 for answers, nothing else. Do not leave any blanks.

Your doctor or nurse can review the questions with you, but this questionnaire is for patients to complete, either alone or with their caregivers.

Who is filling out this questionnaire (check the best answer):

☐ Patient ☐ Caregiver ☐ Patient and Caregiver in Equal Proportion

Copyright © 2008 International Parkinson and Movement Disorder Society. All rights reserved.

This scale may not be copied, distributed or otherwise used in whole or in part without prior written consent of the International Parkinson and Movement Disorder Society

Part I: Non-Motor Aspects of Experiences of Daily Living (nM-EDL)

	SCORE
**1.7 SLEEP PROBLEMS**<br><br>Over the past week, have you had trouble going to sleep at night or staying asleep through the night? Consider how rested you felt after waking up in the morning.<br><br>0: Normal: No problems.<br>1: Slight: Sleep problems are present but usually do not cause trouble getting a full night of sleep.<br>2: Mild: Sleep problems usually cause some difficulties getting a full night of sleep.<br>3: Moderate: Sleep problems cause a lot of difficulties getting a full night of sleep, but I still usually sleep for more than half the night.<br>4: Severe: I usually do not sleep for most of the night.	☐
**1.8 DAYTIME SLEEPINESS**<br><br>Over the past week, have you had trouble staying awake during the daytime?<br><br>0: Normal: No daytime sleepiness.<br>1: Slight: Daytime sleepiness occurs but I can resist and I stay awake.<br>2: Mild: Sometimes I fall asleep when alone and relaxing. For example, while reading or watching TV.<br>3: Moderate: I sometimes fall asleep when I should not. For example, while eating or talking with other people.<br>4: Severe: I often fall asleep when I should not. For example, while eating or talking with other people.	☐

July 1, 2008
Copyright © 2008 International Parkinson and Movement Disorder Society. All rights reserved.
Page 9
This scale may not be copied, distributed or otherwise used in whole or in part without prior written consent of the International Parkinson and Movement Disorder Society

	SCORE
**1.9 PAIN AND OTHER SENSATIONS**<br><br>Over the past week, have you had uncomfortable feelings in your body like pain, aches tingling or cramps?<br><br>0: Normal: No uncomfortable feelings.<br><br>1: Slight: I have these feelings. However, I can do things and be with other people without difficulty.<br><br>2: Mild: These feelings cause some problems when I do things or am with other people.<br><br>3: Moderate: These feelings cause a lot of problems, but they do not stop me from doing things or being with other people.<br><br>4: Severe: These feelings stop me from doing things or being with other people.	☐
**1.10 URINARY PROBLEMS**<br><br>Over the past week, have you had trouble with urine control? For example, an urgent need to urinate, a need to urinate too often, or urine accidents?<br><br>0: Normal: No urine control problems.<br><br>1: Slight: I need to urinate often or urgently. However, these problems do not cause difficulties with my daily activities.<br><br>2: Mild: Urine problems cause some difficulties with my daily activities. However, I do not have urine accidents.<br><br>3: Moderate: Urine problems cause a lot of difficulties with my daily activities, including urine accidents.<br><br>4: Severe: I cannot control my urine and use a protective garment or have a bladder tube.	☐

Copyright © 2008 International Parkinson and Movement Disorder Society. All rights reserved.

This scale may not be copied, distributed or otherwise used in whole or in part without prior written consent of the International Parkinson and Movement Disorder Society

	SCORE
**1.11 CONSTIPATION PROBLEMS**<br><br>Over the past week have you had constipation troubles that cause you difficulty moving your bowels?<br><br>0: Normal: No constipation.<br><br>1: Slight: I have been constipated. I use extra effort to move my bowels. However, this problem does not disturb my activities or my being comfortable.<br><br>2: Mild: Constipation causes me to have some troubles doing things or being comfortable.<br><br>3: Moderate: Constipation causes me to have a lot of trouble doing things or being comfortable. However, it does not stop me from doing anything.<br><br>4: Severe: I usually need physical help from someone else to empty my bowels.	☐
**1.12 LIGHT HEADEDNESS ON STANDING**<br><br>Over the past week, have you felt faint, dizzy or foggy when you stand up after sitting or lying down?<br><br>0: Normal: No dizzy or foggy feelings.<br><br>1: Slight: Dizzy or foggy feelings occur. However, they do not cause me troubles doing things.<br><br>2: Mild: Dizzy or foggy feelings cause me to hold on to something, but I do not need to sit or lie back down.<br><br>3: Moderate: Dizzy or foggy feelings cause me to sit or lie down to avoid fainting or falling.<br><br>4: Severe: Dizzy or foggy feelings cause me to fall or faint.	☐

Copyright © 2008 International Parkinson and Movement Disorder Society. All rights reserved.

This scale may not be copied, distributed or otherwise used in whole or in part without prior written consent of the International Parkinson and Movement Disorder Society

SCORE

1.13 FATIGUE

Over the past week, have you usually felt fatigued? This feeling is not part of being sleepy or sad.

0: Normal: No fatigue.

1: Slight: Fatigue occurs. However it does not cause me troubles doing things or being with people.

2: Mild: Fatigue causes me some troubles doing things or being with people.

3: Moderate: Fatigue causes me a lot of troubles doing things or being with people. However, it does not stop me from doing anything.

4: Severe: Fatigue stops me from doing things or being with people.

☐

Part II: Motor Aspects of Experiences of Daily Living (M-EDL)

2.1 SPEECH

Over the past week, have you had problems with your speech?

0: Normal: Not at all (no problems).

1: Slight: My speech is soft, slurred or uneven, but it does not cause others to ask me to repeat myself.

2: Mild: My speech causes people to ask me to occasionally repeat myself, but not everyday.

3: Moderate: My speech is unclear enough that others ask me to repeat myself every day even though most of my speech is understood.

4: Severe: Most or all of my speech cannot be understood.

☐

Copyright © 2008 International Parkinson and Movement Disorder Society. All rights reserved.

This scale may not be copied, distributed or otherwise used in whole or in part without prior written consent of the International Parkinson and Movement Disorder Society

	SCORE
**2.2 SALIVA AND DROOLING**<br><br>Over the past week, have you usually had too much saliva during when you are awake or when you sleep?<br><br>0: Normal: Not at all (no problems).<br>1: Slight: I have too much saliva, but do not drool.<br>2: Mild: I have some drooling during sleep, but none when I am awake.<br>3: Moderate: I have some drooling when I am awake, but I usually do not need tissues or a handkerchief.<br>4: Severe: I have so much drooling that I regularly need to use tissues or a handkerchief to protect my clothes.	☐
**2.3 CHEWING AND SWALLOWING**<br><br>Over the past week, have you usually had problems swallowing pills or eating meals? Do you need your pills cut or crushed or your meals to be made soft, chopped or blended to avoid choking?<br><br>0: Normal: No problems.<br>1: Slight: I am aware of slowness in my chewing or increased effort at swallowing, but I do not choke or need to have my food specially prepared.<br>2: Mild: I need to have my pills cut or my food specially prepared because of chewing or swallowing problems, but I have not choked over the past week.<br>3: Moderate. I choked at least once in the past week.<br>4: Severe: Because of chewing and swallowing problems, I need a feeding tube.	☐

July 1, 2008 Copyright © 2008 International Parkinson and Movement Disorder Society. All rights reserved. Page 13
This scale may not be copied, distributed or otherwise used in whole or in part without prior written consent of the International Parkinson and Movement Disorder Society

	SCORE
**2.4 EATING TASKS**<br><br>Over the past week, have you usually had troubles handling your food and using eating utensils? For example, do you have trouble handling finger foods or using forks, knives, spoons, chopsticks?<br><br>0: Normal: Not at all (no problems).<br><br>1: Slight: I am slow, but I do not need any help handling my food and have not had food spills while eating.<br><br>2: Mild: I am slow with my eating and have occasional food spills. I may need help with a few tasks such as cutting meat.<br><br>3: Moderate: I need help with many eating tasks but can manage some alone.<br><br>4: Severe: I need help for most or all eating tasks.	☐
**2.5 DRESSING**<br><br>Over the past week, have you usually had problems dressing? For example, are you slow or do you need help with buttoning, using zippers, putting on or taking off your clothes or jewelry?<br><br>0: Normal: Not at all (no problems).<br><br>1: Slight: I am slow but I do not need help.<br><br>2: Mild: I am slow and need help for a few dressing tasks (buttons, bracelets).<br><br>3: Moderate: I need help for many dressing tasks.<br><br>4: Severe: I need help for most or all dressing tasks.	☐

Copyright © 2008 International Parkinson and Movement Disorder Society. All rights reserved.

This scale may not be copied, distributed or otherwise used in whole or in part without prior written consent of the International Parkinson and Movement Disorder Society

	SCORE
**2.6 HYGIENE**<br><br>Over the past week, have you usually been slow or do you need help with washing, bathing, shaving, brushing teeth, combing your hair or with other personal hygiene?<br><br>0: Normal: Not at all (no problems).<br>1: Slight: I am slow but I do not need any help.<br>2: Mild: I need someone else to help me with some hygiene tasks.<br>3: Moderate: I need help for many hygiene tasks.<br>4: Severe: I need help for most or all of my hygiene tasks.	☐
**2.7 HANDWRITING**<br><br>Over the past week, have people usually had trouble reading your handwriting?<br><br>0: Normal: Not at all (no problems).<br>1: Slight: My writing is slow, clumsy or uneven, but all words are clear.<br>2: Mild: Some words are unclear and difficult to read.<br>3: Moderate: Many words are unclear and difficult to read.<br>4: Severe: Most or all words cannot be read.	☐
**2.8 DOING HOBBIES AND OTHER ACTIVITIES**<br><br>Over the past week, have you usually had trouble doing your hobbies or other things that you like to do?<br><br>0: Normal: Not at all (no problems).<br>1: Slight: I am a bit slow but do these activities easily.<br>2: Mild: I have some difficulty doing these activities.<br>3: Moderate: I have major problems doing these activities, but still do most.<br>4: Severe: I am unable to do most or all of these activities.	☐

July 1, 2008 *Copyright © 2008 International Parkinson and Movement Disorder Society. All rights reserved.* Page 15
This scale may not be copied, distributed or otherwise used in whole or in part without prior written consent of the International Parkinson and Movement Disorder Society

	SCORE
**2.9 TURNING IN BED**<br><br>Over the past week, do you usually have trouble turning over in bed?<br><br>0: Normal: Not at all (no problems).<br>1: Slight: I have a bit of trouble turning, but I do not need any help.<br>2: Mild I have a lot of trouble turning and need occasional help from someone else.<br>3: Moderate: To turn over I often need help from someone else.<br>4: Severe: I am unable to turn over without help from someone else.	☐
**2.10 TREMOR**<br><br>Over the past week, have you usually had shaking or tremor?<br><br>0: Normal: Not at all. I have no shaking or tremor.<br>1: Slight: Shaking or tremor occurs but does not cause problems with any activities.<br>2: Mild: Shaking or tremor causes problems with only a few activities.<br>3: Moderate: Shaking or tremor causes problems with many of my daily activities.<br>4: Severe: Shaking or tremor causes problems with most or all activities.	☐
**2.11 GETTING OUT OF BED, A CAR, OR A DEEP CHAIR**<br><br>Over the past week, have you usually had trouble getting out of bed, a car seat, or a deep chair?<br><br>0: Normal: Not at all (no problems).<br>1: Slight: I am slow or awkward, but I usually can do it on my first try.<br>2: Mild: I need more than one try to get up or need occasional help.<br>3: Moderate: I sometimes need help to get up, but most times I can still do it on my own.<br>4: Severe: I need help most or all of the time.	☐

Copyright © 2008 International Parkinson and Movement Disorder Society. All rights reserved.

This scale may not be copied, distributed or otherwise used in whole or in part without prior written consent of the International Parkinson and Movement Disorder Society

	SCORE
**2.12 WALKING AND BALANCE**<br><br>Over the past week, have you usually had problems with balance and walking?<br><br>0: Normal: Not at all (no problems).<br>1: Slight: I am slightly slow or may drag a leg. I never use a walking aid.<br>2: Mild: I occasionally use a walking aid, but I do not need any help from another person.<br>3: Moderate: I usually use a walking aid (cane, walker) to walk safely without falling. However, I do not usually need the support of another person.<br>4: Severe: I usually use the support of another person to walk safely without falling.	☐
**2.13 FREEZING**<br><br>Over the past week, on your usual day when walking, do you suddenly stop or freeze as if your feet are stuck to the floor.<br><br>0: Normal: Not at all (no problems).<br>1: Slight: I briefly freeze but I can easily start walking again. I do not need help from someone else or a walking aid (cane or walker) because of freezing.<br>2: Mild: I freeze and have trouble starting to walk again, but I do not need someone's help or a walking aid (cane or walker) because of freezing.<br>3: Moderate: When I freeze I have a lot of trouble starting to walk again and, because of freezing, I sometimes need to use a walking aid or need someone else's help.<br>4: Severe: Because of freezing, most or all of the time, I need to use a walking aid or someone's help.	☐

This completes the questionnaire. We may have asked about problems you do not even have, and may have mentioned problems that you may never develop at all. Not all patients develop all these problems, but because they can occur, it is important to ask all the questions to every patient. Thank you for your time and attention in completing this questionnaire.

 Copyright © 2008 International Parkinson and Movement Disorder Society. All rights reserved.
This scale may not be copied, distributed or otherwise used in whole or in part without prior written consent of the International Parkinson and Movement Disorder Society

Part III: Motor Examination

Overview: This portion of the scale assesses the motor signs of PD. In administering Part III of the MDS-UPDRS the examiner should comply with the following guidelines:

At the top of the form, mark whether the patient is on medication for treating the symptoms of Parkinson's disease and, if on levodopa, the time since the last dose.

Also, if the patient is receiving medication for treating the symptoms of Parkinson's Disease, mark the patient's clinical state using the following definitions:

ON is the typical functional state when patients are receiving medication and have a good response.
OFF is the typical functional state when patients have a poor response in spite of taking medications.

The investigator should "rate what you see". Admittedly, concurrent medical problems such as stroke, paralysis, arthritis, contracture, and orthopedic problems such as hip or knee replacement and scoliosis may interfere with individual items in the motor examination. In situations where it is absolutely impossible to test (e.g., amputations, plegia, limb in a cast), use the notation "**UR**" for Unable to Rate. Otherwise, rate the performance of each task as the patient performs in the context of co-morbidities.

All items must have an integer rating (no half points, no missing ratings).

Specific instructions are provided for the testing of each item. These should be followed in all instances. The investigator demonstrates while describing tasks the patient is to perform and rates function immediately thereafter. For Global Spontaneous Movement and Rest Tremor items (3.14 and 3.17), these items have been placed purposefully at the end of the scale because clinical information pertinent to the score will be obtained throughout the entire examination.

At the end of the rating, indicate if dyskinesia (chorea or dystonia) was present at the time of the examination, and if so, whether these movements interfered with the motor examination.

3a Is the patient on medication for treating the symptoms of Parkinson's Disease? ☐ No ☐ Yes

3b If the patient is receiving medication for treating the symptoms of Parkinson's Disease, mark the patient's clinical state using the following definitions:

☐ ON: On is the typical functional state when patients are receiving medication and have a good response.

☐ OFF: Off is the typical functional state when patients have a poor response in spite of taking medications.

3c Is the patient on Levodopa ? ☐ No ☐ Yes

3.C1 If yes, minutes since last levodopa dose: __________

Copyright © 2008 International Parkinson and Movement Disorder Society. All rights reserved

This scale may not be copied, distributed or otherwise used in whole or in part without prior written consent of the International Parkinson and Movement Disorder Society

3.1 SPEECH	SCORE
Instructions to examiner: Listen to the patient's free-flowing speech and engage in conversation if necessary. Suggested topics: ask about the patient's work, hobbies, exercise, or how he got to the doctor's office. Evaluate volume, modulation (prosody) and clarity, including slurring, palilalia (repetition of syllables) and tachyphemia (rapid speech, running syllables together).<br><br>0: Normal: No speech problems.<br>1: Slight: Loss of modulation, diction or volume, but still all words easy to understand.<br>2: Mild: Loss of modulation, diction, or volume, with a few words unclear, but the overall sentences easy to follow.<br>3: Moderate: Speech is difficult to understand to the point that some, but not most, sentences are poorly understood.<br>4: Severe: Most speech is difficult to understand or unintelligible.	☐
**3.2 FACIAL EXPRESSION**<br><br>Instructions to examiner: Observe the patient sitting at rest for 10 seconds, without talking and also while talking. Observe eye-blink frequency, masked facies or loss of facial expression, spontaneous smiling and parting of lips.<br><br>0: Normal: Normal facial expression.<br>1: Slight: Minimal masked facies manifested only by decreased frequency of blinking.<br>2: Mild: In addition to decreased eye-blink frequency, Masked facies present in the lower face as well, namely fewer movements around the mouth, such as less spontaneous smiling, but lips not parted.<br>3: Moderate: Masked facies with lips parted some of the time when the mouth is at rest.<br>4: Severe: Masked facies with lips parted most of the time when the mouth is at rest.	☐

Copyright © 2008 International Parkinson and Movement Disorder Society. All rights reserved.

This scale may not be copied, distributed or otherwise used in whole or in part without prior written consent of the International Parkinson and Movement Disorder Society

SCORE

3.3 RIGIDITY

Instructions to examiner: Rigidity is judged on slow passive movement of major joints with the patient in a relaxed position and the examiner manipulating the limbs and neck. First, test without an activation maneuver. Test and rate neck and each limb separately. For arms, test the wrist and elbow joints simultaneously. For legs, test the hip and knee joints simultaneously. If no rigidity is detected, use an activation maneuver such as tapping fingers, fist opening/closing, or heel tapping in a limb not being tested. Explain to the patient to go as limp as possible as you test for rigidity.

0: Normal: No rigidity.

1: Slight: Rigidity only detected with activation maneuver.

2: Mild: Rigidity detected without the activation maneuver, but full range of motion is easily achieved.

3: Moderate: Rigidity detected without the activation maneuver; full range of motion is achieved with effort.

4: Severe: Rigidity detected without the activation maneuver and full range of motion not achieved.

☐ Neck

☐ RUE

☐ LUE

☐ RLE

☐ LLE

3.4 FINGER TAPPING

Instructions to examiner: Each hand is tested separately. Demonstrate the task, but do not continue to perform the task while the patient is being tested. Instruct the patient to tap the index finger on the thumb 10 times as quickly AND as big as possible. Rate each side separately, evaluating speed, amplitude, hesitations, halts and decrementing amplitude.

0: Normal: No problems.

1: Slight: Any of the following: a) the regular rhythm is broken with one or two interruptions or hesitations of the tapping movement; b) slight slowing; c) the amplitude decrements near the end of the 10 taps.

2: Mild: Any of the following: a) 3 to 5 interruptions during tapping; b) mild slowing; c) the amplitude decrements midway in the 10-tap sequence.

3: Moderate: Any of the following: a) more than 5 interruptions during tapping or at least one longer arrest (freeze) in ongoing movement; b) moderate slowing; c) the amplitude decrements starting after the 1st tap.

4: Severe: Cannot or can only barely perform the task because of slowing, interruptions or decrements.

☐ R

☐ L

Copyright © 2008 International Parkinson and Movement Disorder Society. All rights reserved.

This scale may not be copied, distributed or otherwise used in whole or in part without prior written consent of the International Parkinson and Movement Disorder Society

SCORE

3.5 HAND MOVEMENTS

Instructions to examiner: Test each hand separately. Demonstrate the task, but do not continue to perform the task while the patient is being tested. Instruct the patient to make a tight fist with the arm bent at the elbow so that the palm faces the examiner. Have the patient open the hand 10 times as fully AND as quickly as possible. If the patient fails to make a tight fist or to open the hand fully, remind him/ her to do so. Rate each side separately, evaluating speed, amplitude, hesitations, halts and decrementing amplitude.

0: Normal: No problem.

1: Slight: Any of the following: a) the regular rhythm is broken with one or two interruptions or hesitations of the movement; b) slight slowing; c) the amplitude decrements near the end of the task.

2: Mild: Any of the following: a) 3 to 5 interruptions during the movements; b) mild slowing; c) the amplitude decrements midway in the task.

3: Moderate: Any of the following: a) more than 5 interruptions during the movement or at least one longer arrest (freeze) in ongoing movement; b) moderate slowing; c) the amplitude decrements starting after the 1st open-and-close sequence.

4: Severe: Cannot or can only barely perform the task because of slowing, interruptions or decrements.

R

L

3.6 PRONATION-SUPINATION MOVEMENTS OF HANDS

Instructions to examiner: Test each hand separately. Demonstrate the task, but do not continue to perform the task while the patient is being tested. Instruct the patient to extend the arm out in front of his/her body with the palms down; then to turn the palm up and down alternately 10 times as fast and as fully as possible. Rate each side separately, evaluating speed, amplitude, hesitations, halts and decrementing amplitude.

0: Normal: No problems.

1: Slight: Any of the following: a) the regular rhythm is broken with one or two interruptions or hesitations of the movement; b) slight slowing; c) the amplitude decrements near the end of the sequence.

2: Mild: Any of the following: a) 3 to 5 interruptions during the movements; b) mild slowing; c) the amplitude decrements midway in the sequence.

3: Moderate: Any of the following: a) more than 5 interruptions during the movement or at least one longer arrest (freeze) in ongoing movement; b) moderate slowing c) the amplitude decrements starting after the 1st supination-pronation sequence.

4: Severe: Cannot or can only barely perform the task because of slowing, interruptions or decrements.

R

L

Copyright © 2008 International Parkinson and Movement Disorder Society. All rights reserved.

This scale may not be copied, distributed or otherwise used in whole or in part without prior written consent of the International Parkinson and Movement Disorder Society

	SCORE
**3.7 TOE TAPPING**<br><br><u>Instructions to examiner</u>: Have the patient sit in a straight-backed chair with arms, both feet on the floor. Test each foot separately. Demonstrate the task, but do not continue to perform the task while the patient is being tested. Instruct the patient to place the heel on the ground in a comfortable position and then tap the toes 10 times as big and as fast as possible. Rate each side separately, evaluating speed, amplitude, hesitations, halts and decrementing amplitude.<br><br>0: Normal: No problem.<br><br>1: Slight: Any of the following: a) the regular rhythm is broken with one or two interruptions or hesitations of the tapping movement; b) slight slowing; c) amplitude decrements near the end of the ten taps.<br><br>2: Mild: Any of the following: a) 3 to 5 interruptions during the tapping movements; b) mild slowing; c) amplitude decrements midway in the task.<br><br>3: Moderate: Any of the following: a) more than 5 interruptions during the tapping movements or at least one longer arrest (freeze) in ongoing movement; b) moderate slowing; c) amplitude decrements after the first tap.<br><br>4: Severe: Cannot or can only barely perform the task because of slowing, interruptions or decrements.	☐ R<br><br>☐ L
**3.8 LEG AGILITY**<br><br><u>Instructions to examiner</u>: Have the patient sit in a straight-backed chair with arms. The patient should have both feet comfortably on the floor. Test each leg separately. Demonstrate the task, but do not continue to perform the task while the patient is being tested. Instruct the patient to place the foot on the ground in a comfortable position and then raise and stomp the foot on the ground 10 times as high and as fast as possible. Rate each side separately, evaluating speed, amplitude, hesitations, halts and decrementing amplitude.<br><br>0: Normal: No problems.<br><br>1: Slight: Any of the following: a) the regular rhythm is broken with one or two interruptions or hesitations of the movement; b) slight slowing; c) amplitude decrements near the end of the task.<br><br>2: Mild: Any of the following: a) 3 to 5 interruptions during the movements; b) mild slowness; c) amplitude decrements midway in the task.<br><br>3: Moderate: Any of the following: a) more than 5 interruptions during the movement or at least one longer arrest (freeze) in ongoing movement; b) moderate slowing in speed; c) amplitude decrements after the first tap.<br><br>4: Severe: Cannot or can only barely perform the task because of slowing, interruptions or decrements.	☐ R<br><br>☐ L

Copyright © 2008 International Parkinson and Movement Disorder Society. All rights reserved.

This scale may not be copied, distributed or otherwise used in whole or in part without prior written consent of the International Parkinson and Movement Disorder Society.

	SCORE
**3.9 ARISING FROM CHAIR**<br><br>Instructions to examiner: Have the patient sit in a straight-backed chair with arms, with both feet on the floor and sitting back in the chair (if the patient is not too short). Ask the patient to cross his/her arms across the chest and then to stand up. If the patient is not successful, repeat this attempt a maximum up to two more times. If still unsuccessful, allow the patient to move forward in the chair to arise with arms folded across the chest. Allow only one attempt in this situation. If unsuccessful, allow the patient to push off using his/her hands on the arms of the chair. Allow a maximum of three trials of pushing off. If still not successful, assist the patient to arise. After the patient stands up, observe the posture for item 3.13.<br><br>0: Normal: No problems. Able to arise quickly without hesitation.<br>1: Slight: Arising is slower than normal; or may need more than one attempt; or may need to move forward in the chair to arise. No need to use the arms of the chair.<br>2: Mild: Pushes self up from arms of chair without difficulty.<br>3: Moderate: Needs to push off, but tends to fall back; or may have to try more than one time using arms of chair, but can get up without help.<br>4: Severe: Unable to arise without help.	☐
**3.10 GAIT**<br><br>Instructions to examiner: Testing gait is best performed by having the patient walking away from and towards the examiner so that both right and left sides of the body can be easily observed simultaneously. The patient should walk at least 10 meters (30 feet), then turn around and return to the examiner. This item measures multiple behaviors: stride amplitude, stride speed, height of foot lift, heel strike during walking, turning, and arm swing, but not freezing. Assess also for "freezing of gait" (next item 3.11) while patient is walking. Observe posture for item 3.13.<br><br>0: Normal: No problems.<br>1: Slight: Independent walking with minor gait impairment.<br>2: Mild: Independent walking but with substantial gait impairment.<br>3: Moderate: Requires an assistance device for safe walking (walking stick, walker) but not a person.<br>4: Severe: Cannot walk at all or only with another person's assistance.	☐

 Copyright © 2008 International Parkinson and Movement Disorder Society. All rights reserved.
This scale may not be copied, distributed or otherwise used in whole or in part without prior written consent of the International Parkinson and Movement Disorder Society

	SCORE
**3.11 FREEZING OF GAIT**<br><br><u>Instructions to examiner</u>: While assessing gait, also assess for the presence of any gait freezing episodes. Observe for start hesitation and stuttering movements especially when turning and reaching the end of the task. To the extent that safety permits, patients may NOT use sensory tricks during the assessment.<br><br>0: Normal: No freezing.<br><br>1: Slight: Freezes on starting, turning or walking through doorway with a single halt during any of these events, but then continues smoothly without freezing during straight walking.<br><br>2: Mild: Freezes on starting, turning or walking through doorway with more than one halt during any of these activities, but continues smoothly without freezing during straight walking.<br><br>3: Moderate: Freezes once during straight walking.<br><br>4: Severe: Freezes multiple times during straight walking.	☐
**3.12 POSTURAL STABILITY**<br><br><u>Instructions to examiner</u>: The test examines the response to sudden body displacement produced by a <u>quick, forceful</u> pull on the shoulders while the patient is standing erect with eyes open and feet comfortably apart and parallel to each other. Test retropulsion. Stand behind the patient and instruct the patient on what is about to happen. Explain that s/he is allowed to take a step backwards to avoid falling. There should be a solid wall behind the examiner, at least 1-2 meters away to allow for the observation of the number of retropulsive steps. The first pull is an instructional demonstration and is purposely milder and not rated. The second time the shoulders are pulled briskly and forcefully towards the examiner with enough force to displace the center of gravity so that patient MUST take a step backwards. The examiner needs to be ready to catch the patient, but must stand sufficiently back so as to allow enough room for the patient to take several steps to recover independently. Do not allow the patient to flex the body abnormally forward in anticipation of the pull. Observe for the number of steps backwards or falling. Up to and including two steps for recovery is considered normal, so abnormal ratings begin with three steps. If the patient fails to understand the test, the examiner can repeat the test so that the rating is based on an assessment that the examiner feels reflects the patient's limitations rather than misunderstanding or lack of preparedness. Observe standing posture for item 3.13<br><br>0: Normal: No problems: Recovers with one or two steps.<br><br>1: Slight: 3-5 steps, but subject recovers unaided.<br><br>2: Mild: More than 5 steps, but subject recovers unaided.<br><br>3: Moderate: Stands safely, but with absence of postural response; falls if not caught by examiner.<br><br>4: Severe: Very unstable, tends to lose balance spontaneously or with just a gentle pull on the shoulders.	☐

Copyright © 2008 International Parkinson and Movement Disorder Society. All rights reserved.

This scale may not be copied, distributed or otherwise used in whole or in part without prior written consent of the International Parkinson and Movement Disorder Society

SCORE

3.13 POSTURE

Instructions to examiner: Posture is assessed with the patient standing erect after arising from a chair, during walking, and while being tested for postural reflexes. If you notice poor posture, tell the patient to stand up straight and see if the posture improves (see option 2 below). Rate the worst posture seen in these three observation points. Observe for flexion and side-to-side leaning.

0:	Normal:	No problems.
1:	Slight:	Not quite erect, but posture could be normal for older person.
2:	Mild:	Definite flexion, scoliosis or leaning to one side, but patient can correct posture to normal posture when asked to do so.
3:	Moderate:	Stooped posture, scoliosis or leaning to one side that cannot be corrected volitionally to a normal posture by the patient.
4:	Severe:	Flexion, scoliosis or leaning with extreme abnormality of posture.

3.14 GLOBAL SPONTANEITY OF MOVEMENT (BODY BRADYKINESIA)

Instructions to examiner: This global rating combines all observations on slowness, hesitancy, and small amplitude and poverty of movement in general, including a reduction of gesturing and of crossing the legs. This assessment is based on the examiner's global impression after observing for spontaneous gestures while sitting, and the nature of arising and walking.

0:	Normal:	No problems.
1:	Slight:	Slight global slowness and poverty of spontaneous movements.
2:	Mild:	Mild global slowness and poverty of spontaneous movements.
3:	Moderate:	Moderate global slowness and poverty of spontaneous movements.
4:	Severe:	Severe global slowness and poverty of spontaneous movements.

3.15 POSTURAL TREMOR OF THE HANDS

Instructions to examiner: All tremor, including re-emergent rest tremor, that is present in this posture is to be included in this rating. Rate each hand separately. Rate the highest amplitude seen. Instruct the patient to stretch the arms out in front of the body with palms down. The wrist should be straight and the fingers comfortably separated so that they do not touch each other. Observe this posture for 10 seconds.

0:	Normal:	No tremor.
1:	Slight:	Tremor is present but less than 1 cm in amplitude.
2:	Mild:	Tremor is at least 1 but less than 3 cm in amplitude.
3:	Moderate:	Tremor is at least 3 but less than 10 cm in amplitude.
4:	Severe:	Tremor is at least 10 cm in amplitude.

R

L

Copyright © 2008 International Parkinson and Movement Disorder Society. All rights reserved.

This scale may not be copied, distributed or otherwise used in whole or in part without prior written consent of the International Parkinson and Movement Disorder Society

SCORE

3.16 KINETIC TREMOR OF THE HANDS

Instructions to examiner: This is tested by the finger-to-nose maneuver. With the arm starting from the outstretched position, have the patient perform at least three finger-to-nose maneuvers with each hand reaching as far as possible to touch the examiner's finger. The finger-to-nose maneuver should be performed slowly enough not to hide any tremor that could occur with very fast arm movements. Repeat with the other hand, rating each hand separately. The tremor can be present throughout the movement or as the tremor reaches either target (nose or finger). Rate the highest amplitude seen.

0: Normal:	No tremor.
1: Slight:	Tremor is present but less than 1 cm in amplitude.
2: Mild:	Tremor is at least 1 but less than 3 cm in amplitude.
3: Moderate:	Tremor is at least 3 but less than 10 cm in amplitude.
4: Severe:	Tremor is at least 10 cm in amplitude.

R

L

3.17 REST TREMOR AMPLITUDE

Instructions to examiner: This and the next item have been placed purposefully at the end of the examination to allow the rater to gather observations on rest tremor that may appear at any time during the exam, including when quietly sitting, during walking and during activities when some body parts are moving but others are at rest. Score the maximum amplitude that is seen at any time as the final score. Rate only the amplitude and not the persistence or the intermittency of the tremor.
As part of this rating, the patient should sit quietly in a chair with the hands placed on the arms of the chair (not in the lap) and the feet comfortably supported on the floor for 10 seconds with no other directives. Rest tremor is assessed separately for all four limbs and also for the lip/jaw. Rate only the maximum amplitude that is seen at any time as the final rating.

Extremity ratings

0: Normal:	No tremor.
1: Slight:	≤ 1 cm in maximal amplitude.
2: Mild:	> 1 cm but < 3 cm in maximal amplitude.
3: Moderate:	3 - 10 cm in maximal amplitude.
4: Severe:	> 10 cm in maximal amplitude.

Lip/Jaw ratings

0: Normal:	No tremor.
1: Slight:	≤ 1 cm in maximal amplitude.
2: Mild:	> 1 cm but ≤ 2 cm in maximal amplitude.
3: Moderate:	> 2 cm but ≤ 3 cm in maximal amplitude.
4: Severe:	> 3 cm in maximal amplitude.

RUE

LUE

RLE

LLE

Lip/Jaw

Copyright © 2008 International Parkinson and Movement Disorder Society. All rights reserved.

This scale may not be copied, distributed or otherwise used in whole or in part without prior written consent of the International Parkinson and Movement Disorder Society

	SCORE
**3.18 CONSTANCY OF REST TREMOR**<br><br><u>Instructions to examiner</u>: This item receives one rating for all rest tremor and focuses on the constancy of rest tremor during the examination period when different body parts are variously at rest. It is rated purposefully at the end of the examination so that several minutes of information can be coalesced into the rating.<br><br>0: Normal: No tremor.<br>1: Slight: Tremor at rest is present ≤ 25% of the entire examination period.<br>2: Mild: Tremor at rest is present 26-50% of the entire examination period.<br>3: Moderate: Tremor at rest is present 51-75% of the entire examination period.<br>4: Severe: Tremor at rest is present > 75% of the entire examination period.	☐
**DYSKINESIA IMPACT ON PART III RATINGS**<br><br>A. Were dyskinesias (chorea or dystonia) present during examination? ☐ No ☐ Yes<br>B. If yes, did these movements interfere with your ratings? ☐ No ☐ Yes	
**HOEHN AND YAHR STAGE**<br><br>0: Asymptomatic.<br>1: Unilateral involvement only.<br>2: Bilateral involvement without impairment of balance.<br>3: Mild to moderate involvement; some postural instability but physically independent; needs assistance to recover from pull test.<br>4: Severe disability; still able to walk or stand unassisted.<br>5: Wheelchair bound or bedridden unless aided.	☐

July 1, 2008 Copyright © 2008 International Parkinson and Movement Disorder Society. All rights reserved. Page 27
This scale may not be copied, distributed or otherwise used in whole or in part without prior written consent of the International Parkinson and Movement Disorder Society

Part IV: Motor Complications

Overview and Instructions: In this section, the rater uses historical and objective information to assess two motor complications, dyskinesias and motor fluctuations that include OFF-state dystonia. Use all information from patient, caregiver, and the examination to answer the six questions that summarize function over the past week including today. As in the other sections, rate using only integers (no half points allowed) and leave no missing ratings. If the item cannot be rated, place UR for Unable to Rate. You will need to choose some answers based on percentages, and therefore you will need to establish how many hours generally are awake hours and use this figure as the denominator for "OFF" time and dyskinesias. For "OFF dystonia", the total "Off" time will be the denominator. Operational definitions for examiner's use.

Dyskinesias: Involuntary random movements
Words that patients often recognize for dyskinesias include "irregular jerking", "wiggling", "twitching". It is essential to stress to the patient the difference between dyskinesias and tremor, a common error when patients are assessing dyskinesias.

Dystonia: contorted posture, often with a twisting component:
Words that patients often recognize for dystonia include "spasms", "cramps", "posture".

Motor fluctuation: Variable response to medication:
Words that patients often recognize for motor fluctuation include "wearing out", "wearing off", "roller-coaster effect", "on-off", "uneven medication effects".

OFF: Typical functional state when patients have a poor response in spite of taking mediation or the typical functional response when patients are on NO treatment for parkinsonism. Words that patients often recognize include "low time", "bad time", "shaking time", "slow time", "time when my medications don't work."

ON: Typical functional state when patients are receiving medication and have a good response:
Words that patients often recognize include "good time", "walking time", "time when my medications work."

A. DYSKINESIAS [exclusive of OFF-state dystonia]

4.1 TIME SPENT WITH DYSKINESIAS

SCORE

Instructions to examiner: Determine the hours in the usual waking day and then the hours of dyskinesias. Calculate the percentage. If the patient has dyskinesias in the office, you can point them out as a reference to ensure that patients and caregivers understand what they are rating. You may also use your own acting skills to enact the dyskinetic movements you have seen in the patient before or show them dyskinetic movements typical of other patients. Exclude from this question early morning and nighttime painful dystonia.

Instructions to patient [and caregiver]: Over the past week, how many hours do you usually sleep on a daily basis, including nighttime sleep and daytime napping? Alright, if you sleep ___ hrs, you are awake ____ hrs. Out of those awake hours, how many hours in total do you have wiggling, twitching or jerking movements? Do not count the times when you have tremor, which is a regular back and forth shaking or times when you have painful foot cramps or spasms in the early morning or at nighttime. I will ask about those later. Concentrate only on these types of wiggling, jerking and irregular movements. Add up all the time during the waking day when these usually occur. How many hours ____ (use this number for your calculations).

0: Normal: No dyskinesias.

1: Slight: ≤ 25% of waking day.

2: Mild: 26 - 50% of waking day.

3: Moderate: 51 - 75% of waking day.

4: Severe: > 75% of waking day.

1. Total Hours Awake: ____
2. Total Hours with Dyskinesia: ____
3. % Dyskinesia = ((2/1)*100): ____

Copyright © 2008 International Parkinson and Movement Disorder Society. All rights reserved.

This scale may not be copied, distributed or otherwise used in whole or in part without prior written consent of the International Parkinson and Movement Disorder Society

	SCORE
**4.2 FUNCTIONAL IMPACT OF DYSKINESIAS**<br><br>Instructions to examiner: Determine the degree to which dyskinesias impact on the patient's daily function in terms of activities and social interactions. Use the patient's and caregiver's response to your question and your own observations during the office visit to arrive at the best answer.<br><br>*Instructions to patient [and caregiver]: Over the past week, did you usually have trouble doing things or being with people when these jerking movements occurred? Did they stop you from doing things or from being with people?*<br><br>0: Normal: No dyskinesias or no impact by dyskinesias on activities or social interactions.<br><br>1: Slight: Dyskinesias impact on a few activities, but the patient usually performs all activities and participates in all social interactions during dyskinetic periods.<br><br>2: Mild: Dyskinesias impact on many activities, but the patient usually performs all activities and participates in all social interactions during dyskinetic periods.<br><br>3: Moderate: Dyskinesias impact on activities to the point that the patient usually does not perform some activities or does not usually participate in some social activities during dyskinetic episodes.<br><br>4: Severe: Dyskinesias impact on function to the point that the patient usually does not perform most activities or participate in most social interactions during dyskinetic episodes.	☐
**B. MOTOR FLUCTUATIONS**	
**4.3 TIME SPENT IN THE OFF STATE**<br><br>Instructions to examiner: Use the number of waking hours derived from 4.1 and determine the hours spent in the "OFF" state. Calculate the percentage. If the patient has an OFF period in the office, you can point to this state as a reference. You may also use your knowledge of the patient to describe a typical OFF period. Additionally you may use your own acting skills to enact an OFF period you have seen in the patient before or show them OFF function typical of other patients. Mark down the typical number of OFF hours, because you will need this number for completing 4.6.<br><br>*Instructions to patient [and caregiver]: Some patients with Parkinson's disease have a good effect from their medications throughout their awake hours and we call that "ON" time. Other patients take their medications but still have some hours of low time, bad time, slow time or shaking time. Doctors call these low periods "OFF" time. Over the past week, you told me before that you are general awake ____ hrs each day. Out of these awake hours, how many hours in total do you usually have this type of low level or OFF function ____ (use this number for your calculations).*<br><br>0: Normal: No OFF time.<br><br>1: Slight: ≤ 25% of waking day.<br><br>2: Mild: 26 - 50% of waking day.<br><br>3: Moderate: 51 - 75% of waking day.<br><br>4: Severe: > 75% of waking day.<br><br>1. Total Hours Awake: ____<br>2. Total Hours OFF: ____<br>3. % OFF = ((2/1)*100): ____	☐

July 1, 2008 Copyright © 2008 International Parkinson and Movement Disorder Society. All rights reserved. Page 29
This scale may not be copied, distributed or otherwise used in whole or in part without prior written consent of the International Parkinson and Movement Disorder Society

	SCORE
**4.4 FUNCTIONAL IMPACT OF FLUCTUATIONS**<br><br><u>Instructions to examiner</u>: Determine the degree to which motor fluctuations impact on the patient's daily function in terms of activities and social interactions. This question concentrates on the difference between the ON state and the OFF state. If the patient has no OFF time, the rating must be 0, but if patients have very mild fluctuations, it is still possible to be rated 0 on this item if no impact on activities occurs. Use the patient's and caregiver's response to your question and your own observations during the office visit to arrive at the best answer.<br><br>*<u>Instructions to patient [and caregiver]</u>: Think about when those low or "OFF" periods have occurred over the past week. Do you usually have more problems doing things or being with people than compared to the rest of the day when you feel your medications working? Are there some things you usually do during a good period that you have trouble with or stop doing during a low period?*<br><br>0: Normal: No fluctuations or No impact by fluctuations on performance of activities or social interactions.<br><br>1: Slight: Fluctuations impact on a few activities, but during OFF, the patient usually performs all activities and participates in all social interactions that typically occur during the ON state.<br><br>2: Mild: Fluctuations impact many activities, but during OFF, the patient still usually performs all activities and participates in all social interactions that typically occur during the ON state.<br><br>3: Moderate: Fluctuations impact on the performance of activities during OFF to the point that the patient usually does not perform some activities or participate in some social interactions that are performed during ON periods.<br><br>4: Severe: Fluctuations impact on function to the point that, during OFF, the patient usually does not perform most activities or participate in most social interactions that are performed during ON periods.	☐
**4.5 COMPLEXITY OF MOTOR FLUCTUATIONS**<br><br><u>Instructions to examiner</u>: Determine the usual predictability of OFF function whether due to dose, time of day, food intake or other factors. Use the information provided by the patients and caregiver and supplement with your own observations. You will ask if the patient can count on them always coming at a special time, mostly coming at a special time (in which case you will probe further to separate slight from mild), only sometimes coming at a special time or are they totally unpredictable? Narrowing down the percentage will allow you to find the correct answer.<br><br>*<u>Instructions to patient [and caregiver]</u>: For some patients, the low or "OFF" periods happen at certain times during day or when they do activities like eating or exercising. Over the past week, do you usually know when your low periods will occur? In other words, do your low periods <u>always</u> come at a certain time? Do they <u>mostly</u> come at a certain time? Do they <u>only sometimes</u> come at a certain time? Are your low periods totally unpredictable?"*<br><br>0: Normal: No motor fluctuations.<br><br>1: Slight: OFF times are predictable all or almost all of the time (> 75%).<br><br>2: Mild: OFF times are predictable most of the time (51-75%).<br><br>3: Moderate: OFF times are predictable some of the time (26-50%).<br><br>4: Severe: OFF episodes are rarely predictable (≤ 25%).	☐

Copyright © 2008 International Parkinson and Movement Disorder Society. All rights reserved.

This scale may not be copied, distributed or otherwise used in whole or in part without prior written consent of the International Parkinson and Movement Disorder Society

C. "OFF" DYSTONIA

4.6 PAINFUL OFF-STATE DYSTONIA

Instructions to examiner: For patients who have motor fluctuations, determine what proportion of the OFF episodes usually includes painful dystonia? You have already determined the number of hours of "OFF" time (4.3). Of these hours, determine how many are associated with dystonia and calculate the percentage. If there is no OFF time, mark 0.

Instructions to patient [and caregiver]: In one of the questions I asked earlier, you said you generally have ____ hours of low or "OFF" time when your Parkinson's disease is under poor control. During these low or "OFF" periods, do you usually have painful cramps or spasms? Out of the total ____ hrs of this low time, if you add up all the time in a day when these painful cramps come, how many hours would this make?

0: Normal: No dystonia OR NO OFF TIME.

1: Slight: ≤ 25% of time in OFF state.

2: Mild: 26-50% of time in OFF state.

3: Moderate: 51-75% of time in OFF state.

4: Severe: > 75% of time in OFF state.

☐

1. Total Hours Off: _____
2. Total Off Hours w/Dystonia: _____
3. % Off Dystonia = ((2/1)*100): _____

Summary statement to patient: READ TO PATIENT

This completes my rating of your Parkinson's disease. I know the questions and tasks have taken several minutes, but I wanted to be complete and cover all possibilities. In doing so, I may have asked about problems you do not even have, and I may have mentioned problems that you may never develop at all. Not all patients develop all these problems, but because they can occur, it is important to ask all the questions to every patient. Thank you for your time and attention in completing this scale with me.

July 1, 2008 Copyright © 2008 International Parkinson and Movement Disorder Society. All rights reserved. Page 31
This scale may not be copied, distributed or otherwise used in whole or in part without prior written consent of the International Parkinson and Movement Disorder Society

MDS UPDRS Score Sheet

1.A	Source of information	☐ Patient<br>☐ Caregiver<br>☐ Patient + Caregiver	3.3b	Rigidity– RUE	
			3.3c	Rigidity– LUE	
**Part I**			3.3d	Rigidity– RLE	
1.1	Cognitive impairment		3.3e	Rigidity– LLE	
1.2	Hallucinations and psychosis		3.4a	Finger tapping– Right hand	
1.3	Depressed mood		3.4b	Finger tapping– Left hand	
1.4	Anxious mood		3.5a	Hand movements– Right hand	
1.5	Apathy		3.5b	Hand movements– Left hand	
1.6	Features of DDS		3.6a	Pronation- supination movements– Right hand	
1.6a	Who is filling out questionnaire	☐ Patient<br>☐ Caregiver<br>☐ Patient + Caregiver	3.6b	Pronation- supination movements– Left hand	
			3.7a	Toe tapping– Right foot	
1.7	Sleep problems		3.7b	Toe tapping– Left foot	
1.8	Daytime sleepiness		3.8a	Leg agility– Right leg	
1.9	Pain and other sensations		3.8b	Leg agility– Left leg	
1.10	Urinary problems		3.9	Arising from chair	
1.11	Constipation problems		3.10	Gait	
1.12	Light headedness on standing		3.11	Freezing of gait	
1.13	Fatigue		3.12	Postural stability	
**Part II**			3.13	Posture	
2.1	Speech		3.14	Global spontaneity of movement	
2.2	Saliva and drooling		3.15a	Postural tremor– Right hand	
2.3	Chewing and swallowing		3.15b	Postural tremor– Left hand	
2.4	Eating tasks		3.16a	Kinetic tremor– Right hand	
2.5	Dressing		3.16b	Kinetic tremor– Left hand	
2.6	Hygiene		3.17a	Rest tremor amplitude– RUE	
2.7	Handwriting		3.17b	Rest tremor amplitude– LUE	
2.8	Doing hobbies and other activities		3.17c	Rest tremor amplitude– RLE	
2.9	Turning in bed		3.17d	Rest tremor amplitude– LLE	
2.10	Tremor		3.17e	Rest tremor amplitude– Lip/jaw	
2.11	Getting out of bed		3.18	Constancy of rest	
2.12	Walking and balance			Were dyskinesias present?	☐ No ☐ Yes
2.13	Freezing			Did these movements interfere with ratings?	☐ No ☐ Yes
3a	Is the patient on medication?	☐ No ☐ Yes		Hoehn and Yahr Stage	
3b	Patient's clinical state	☐ Off ☐ On	**Part IV**		
3c	Is the patient on Levodopa?	☐ No ☐ Yes	4.1	Time spent with dyskinesias	
3.C1	If yes, minutes since last dose:		4.2	Functional impact of dyskinesias	
**Part III**			4.3	Time spent in the OFF state	
3.1	Speech		4.4	Functional impact of fluctuations	
3.2	Facial expression		4.5	Complexity of motor fluctuations	
3.3a	Rigidity– Neck		4.6	Painful OFF-state dystonia	

July 1, 2008 Page 32

Copyright © 2008 International Parkinson and Movement Disorder Society. All rights reserved.
This scale may not be copied, distributed or otherwise used in whole or in part without prior written consent of the International Parkinson and Movement Disorder Society

APPENDIX F. PATIENT GLOBAL IMPRESSION OF CHANGE (PGI-C)

The subject will independently rate the following question of Patient Global Impression of Change (PGI-C) based on his/her overall impression at Visit 5 (Week 10), Visit 6 (Week 15), and Visit 7 (Week 20) or early discontinuation.

Patient Global Impression of Change:

Compared to your condition prior to your starting on this study, how much has your condition changed with your current treatment?

❑ 1	❑ 2	❑ 3	❑ 4	❑ 5	❑ 6	❑ 7
Very Much Worse	Much Worse	Minimally Worse	No Change	Minimally Improved	Much Improved	Very Much Improved

APPENDIX G. CLINICAL GLOBAL IMPRESSION OF CHANGE (CGI-C)

The Investigator rates each subject with the following question as part of Visit 5 (Week 10), Visit 6 (Week 15), and Visit 7 (Week 20) or early discontinuation:

Clinical Global Impression of Change:

In your opinion, how much has the subject's overall condition and Parkinson's disease symptoms changed since starting on the study?

❑ 1	❑ 2	❑ 3	❑ 4	❑ 5	❑ 6	❑ 7
Very Much Worse	Much Worse	Minimally Worse	Neutral	Minimally Improved	Much Improved	Very Much Improved

Guy W. ECDEU assessment manual for psychopharmacology publication; ADM, 76-338.

Washington DC, US. Department of health, education and welfare, 1976.

APPENDIX H. PATIENT GLOBAL IMPRESSION OF SEVERITY (PGI-S)

Patient Global Impression – Severity Scale

Severity of Illness

Considering the severity of your Parkinson's disease, how severe is your condition at this time?

Severity Score:

❑ 1	❑ 2	❑ 3	❑ 4	❑ 5	❑ 6	❑ 7
Normal,<br>not at all ill	Borderline<br>ill	Mildly<br>ill	Moderately<br>ill	Markedly<br>ill	Severely<br>ill	Extremely<br>severely ill

APPENDIX I. CLINICAL GLOBAL IMPRESSION OF SEVERITY (CGI-S)

The Investigator will independently rate the following question of Clinical Global Impression of Severity (CGI-S) based on his/her overall impression of the study medication at Visit 1, Visit 4, and Visit 7 or early discontinuation.

Clinical Global Impression – Severity Scale

Severity of Illness

Considering your total clinical experience with this particular PD population, how ill is the patient at this time?

Severity Score:

❑ 1	❑ 2	❑ 3	❑ 4	❑ 5	❑ 6	❑ 7
Normal,<br>not at all ill	Borderline<br>ill	Mildly<br>ill	Moderately<br>ill	Markedly<br>ill	Severely<br>ill	Among the<br>most extremely<br>ill of subjects

Guy W. ECDEU assessment manual for psychopharmacology publication; ADM, 76-338.

Washington DC, US. Department of health, education and welfare, 1976.

APPENDIX J. 39-ITEM PARKINSON'S DISEASE QUESTIONNAIRE (PDQ-39)

Parkinson's Disease Quality of Life Questionnaire (PDQ-39)

Due to having Parkinson's disease,
how often during the last month have you…

*Please **check one box** for each question*

		Never	Occasionally	Sometimes	Often	Always or cannot do at all
**1.**	had difficulty doing the leisure activities you would like to do?	☐	☐	☐	☐	☐
**2.**	had difficulty looking after your home, for example, housework, cooking or yardwork?	☐	☐	☐	☐	☐
**3.**	had difficulty carrying grocery bags?	☐	☐	☐	☐	☐
**4.**	had problems walking half a mile?	☐	☐	☐	☐	☐
**5.**	had problems walking 100 yards (approximately 1 block)?	☐	☐	☐	☐	☐
**6.**	had problems getting around the house as easily as you would like?	☐	☐	☐	☐	☐
**7.**	had difficulty getting around in public places?	☐	☐	☐	☐	☐
**8.**	needed someone else to accompany you when you went out?	☐	☐	☐	☐	☐

Please verify that you have ***checked one box for each question***
before going on to the next page.

PDQ-39 © Copyright, Oxford University Innovation Limited 1993. All Rights Reserved.
The authors, being Professor Crispin Jenkinson, Professor Ray Fitzpatrick and Ms Viv Peto, have asserted their moral rights. Formatted from Final English (US) PDQ-39 1996

Due to having Parkinson's disease,
how often during the last month have you…

*Please **check one box** for each question*

		Never	Occasionally	Sometimes	Often	Always or cannot do at all
**9.**	felt frightened or worried about falling in public?	☐	☐	☐	☐	☐
**10.**	been confined to the house more than you would like?	☐	☐	☐	☐	☐
**11.**	had difficulty showering and bathing?	☐	☐	☐	☐	☐
**12.**	had difficulty dressing?	☐	☐	☐	☐	☐
**13.**	had difficulty with buttons or shoelaces?	☐	☐	☐	☐	☐
**14.**	had problems writing clearly?	☐	☐	☐	☐	☐
**15.**	had difficulty cutting up your food?	☐	☐	☐	☐	☐
**16.**	had difficulty holding a drink without spilling it?	☐	☐	☐	☐	☐
**17.**	felt depressed?	☐	☐	☐	☐	☐
**18.**	felt isolated and lonely?	☐	☐	☐	☐	☐

Please verify that you have ***checked one box for each question***
before going on to the next page.

PDQ-39 © Copyright, Oxford University Innovation Limited 1993. All Rights Reserved.
The authors, being Professor Crispin Jenkinson, Professor Ray Fitzpatrick and Ms Viv Peto, have asserted their moral rights. Formatted from Final English (US) PDQ-39 1996

Due to having Parkinson's disease,
how often during the last month have you…

*Please **check one box** for each question*

		Never	Occasionally	Sometimes	Often	Always
**19.**	felt weepy or tearful?	☐	☐	☐	☐	☐
**20.**	felt angry or bitter?	☐	☐	☐	☐	☐
**21.**	felt anxious?	☐	☐	☐	☐	☐
**22.**	felt worried about your future?	☐	☐	☐	☐	☐
**23.**	felt you had to hide your Parkinson's from people?	☐	☐	☐	☐	☐
**24.**	avoided situations which involve eating or drinking in public?	☐	☐	☐	☐	☐
**25.**	felt embarrassed in public due to having Parkinson's disease?	☐	☐	☐	☐	☐
**26.**	felt worried about other people's reaction to you?	☐	☐	☐	☐	☐
**27.**	had problems with your close personal relationships?	☐	☐	☐	☐	☐

Please verify that you have ***checked one box for each question***
before going on to the next page.

PDQ-39 © Copyright, Oxford University Innovation Limited 1993. All Rights Reserved.
The authors, being Professor Crispin Jenkinson, Professor Ray Fitzpatrick and Ms Viv Peto, have asserted their moral rights. Formatted from Final English (US) PDQ-39 1996

Due to having Parkinson's disease,
how often during the last month have you…

*Please **check one box** for each question*

		Never	Occasionally	Sometimes	Often	Always
**28.**	lacked the support you needed from your spouse or partner? *If you do not have a spouse or Partner, please check here* ☐	☐	☐	☐	☐	☐
**29.**	lacked the support you needed from your family or close friends?	☐	☐	☐	☐	☐
**30.**	unexpectedly fallen asleep during the day?	☐	☐	☐	☐	☐
**31.**	had problems with your concentration, for example when reading or watching TV?	☐	☐	☐	☐	☐
**32.**	felt your memory was failing?	☐	☐	☐	☐	☐
**33.**	had distressing dreams or hallucinations?	☐	☐	☐	☐	☐
**34.**	had difficulty speaking?	☐	☐	☐	☐	☐
**35.**	felt unable to communicate effectively?	☐	☐	☐	☐	☐
**36.**	felt ignored by people?	☐	☐	☐	☐	☐

Please verify that you have ***checked one box for each question***
before going on to the next page.

PDQ-39 © Copyright, Oxford University Innovation Limited 1993. All Rights Reserved.
The authors, being Professor Crispin Jenkinson, Professor Ray Fitzpatrick and Ms Viv Peto, have asserted their moral rights. Formatted from Final English (US) PDQ-39 1996

Due to having Parkinson's disease,
how often during the last month have you…

*Please **check one box** for each question*

		Never	Occasionally	Sometimes	Often	Always
**37.**	had painful muscle cramps or spasms?	☐	☐	☐	☐	☐
**38.**	had aches and pains in your joints or body?	☐	☐	☐	☐	☐
**39.**	felt uncomfortably hot or cold?	☐	☐	☐	☐	☐

Please verify that you have ***checked one box for each question*****.**

Thank you for completing the questionnaire.

PDQ-39 © Copyright, Oxford University Innovation Limited 1993. All Rights Reserved.
The authors, being Professor Crispin Jenkinson, Professor Ray Fitzpatrick and Ms Viv Peto, have asserted their moral rights. Formatted from Final English (US) PDQ-39 1996

APPENDIX K. GASTROPARESIS CARDINAL SYMPTOM INDEX (GCSI)

GCSI

This questionnaire asks you about the severity of symptoms you may have related to your gastrointestinal problem. There are no right or wrong answers. Please answer each question as accurately as possible.

For each symptom, please *circle the number* that best describes how *severe* the symptom has been during the past 2 weeks. If you have not experienced this symptom, circle 0. If the symptom has been very mild, circle 1. If the symptom has been mild, circle 2. If it has been moderate, circle 3. If it has been severe, circle 4. If it has been very severe, circle 5. Please be sure to answer every question.

Please rate the severity of the following symptoms during the past 2 weeks.

	None	Very Mild	Mild	Moderate	Severe	VerySevere
1. nausea (feeling sick to your stomach as if you were going to vomit or throw up)	0	1	2	3	4	5
2. retching (heaving as if to vomit, but nothing comes up)	0	1	2	3	4	5
3. vomiting	0	1	2	3	4	5
4. stomach fullness	0	1	2	3	4	5
5. not able to finish a normal-sized meal	0	1	2	3	4	5
6. feeling excessively full after meals	0	1	2	3	4	5
7. loss of appetite	0	1	2	3	4	5
8. bloating (feeling like you need to loosen your clothes)	0	1	2	3	4	5
9. stomach or belly visibly larger	0	1	2	3	4	5

Revicki DA, Rentz AM, Dubois D, Kahrilas P, Stanghellini V, Talley NJ, Tack J. Gastroparesis Cardinal Symptom Index (GCSI): development and validation of a patient reported assessment of severity of gastroparesis symptoms. Qual Life Res. 2004;13(4):833-44

Revicki DA, Rentz AM, Dubois D, Kahrilas P, Stanghellini V, Talley NJ, Tack J. Development and validation of a patient-assessed gastroparesis symptom severity measure: the Gastroparesis Cardinal Symptom Index. Aliment Pharmacol Ther. 2003;18(1):141-50

GCSI © 2001 Mapi Research Trust, All rights reserved
The Questionnaire contact information and permission to use: Mapi Research Trust, Lyon, France – Internet: https://eprovide.mapi-trust.org

APPENDIX L. NON-MOTOR SYMPTOM ASSESSMENT SCALE FOR PARKINSON'S DISEASE (NMSS)

Non-Motor Symptom assessment scale for Parkinson's Disease

Patient ID No: ________________________ **Initials:** ________________ **Age:** __________

Symptoms assessed over the last month. Each symptom scored with respect to:

Severity: 0 = None, 1 = Mild: symptoms present but causes little distress or disturbance to patient; 2 = Moderate: some distress or disturbance to patient; 3 = Severe: major source of distress or disturbance to patient.

Frequency: 1 = Rarely (<1/wk); 2 = Often (1/wk); 3 = Frequent (several times per week); 4 = Very Frequent (daily or all the time)

Domains will be weighed differentially. Yes/ No answers are not included in final frequency x severity calculation.
(Bracketed text in questions within the scale is included as an explanatory aid).

	Severity	Frequency	Frequency x Severity
**Domain 1: Cardiovascular including falls**			
1. Does the patient experience light-headedness, dizziness, weakness on standing from sitting or lying position?	☐	☐	☐
2. Does the patient fall because of fainting or blacking out?	☐	☐	☐
**SCORE:**			☐
**Domain 2: Sleep/fatigue**			
3. Does the patient doze off or fall asleep unintentionally during daytime activities? (For example, during conversation, during mealtimes, or while watching television or reding).	☐	☐	☐
4. Does fatigue (tiredness) or lack of energy (not slowness) limit the patient's daytime activities?	☐	☐	☐
5. Does the patient have difficulties falling or staying asleep?	☐	☐	☐
6. Does the patient experience an urge to move the legs or restlessness in legs that improves with movement when he/she is sitting or lying down inactive?	☐	☐	☐
**SCORE:**			☐
**Domain 3: Mood /Cognition**			
7. Has the patient lost interest in his/her surroundings?	☐	☐	☐
8. Has the patient lost interest in doing things or lack motivation to start new activities?	☐	☐	☐
9. Does the patient feel nervous, worried or frightened for no apparent reason?	☐	☐	☐
10. Does the patient seem sad or depressed or has he/she reported such feelings?	☐	☐	☐
11. Does the patient have flat moods without the normal "highs" and " lows"?	☐	☐	☐
12. Does the patient have difficulty in experiencing pleasure from their usual activities or report that they lack pleasure?	☐	☐	☐
**SCORE:**			☐
**Domain 4: Perceptual problems/hallucinations**			
13. Does the patient indicate that he/she sees things that are not there?	☐	☐	☐
14. Does the patient have beliefs that you know are not true? (For example, about being harmed, being robbed or being unfaithful)	☐	☐	☐
15. Does the patient experience double vision? (2 separate real objects and not blurred vision)	☐	☐	☐
**SCORE:**			☐

	Severity	Frequency	Frequency x Severity
**Domain 5: Attention/ Memory**			
16. Does the patient have problems sustaining concentration during activities? (For example, reading or having a conversation)	☐	☐	☐
17. Does the patient forget things that he/she has been told a short time ago or events that happened in the last few days?	☐	☐	☐
18. Does the patient forget to do things? (For example, take tablets or turn off domestic appliances?)	☐	☐	☐
**SCORE:**			☐
**Domain 6: Gastrointestinal tract**			
19. Does the patient dribble saliva during the day?	☐	☐	☐
20. Does the patient having difficulty swallowing?	☐	☐	☐
21. Does the patient suffer from constipation? (Bowel action less than three times weekly)	☐	☐	☐
**SCORE:**			☐
**Domain 7: Urinary**			
22. Does the patient have difficulty holding urine? (Urgency)	☐	☐	☐
23. Does the patient have to void within 2 hours of last voiding? (Frequency)	☐	☐	☐
24. Does the patient have to get up regularly at night to pass urine? (Nocturia)	☐	☐	☐
**SCORE:**			☐
**Domain 8: Sexual function**			
25. Does the patient have altered interest in sex? (Very much increased or decreased, please underline)	☐	☐	☐
26. Does the patient have problems having sex?	☐	☐	☐
**SCORE:**			☐
**Domain 9: Miscellaneous**			
27. Does the patient suffer from pain not explained by other known conditions? (Is it related to intake of drugs and is it relieved by antiparkinson drugs?)	☐	☐	☐
28. Does the patient report a change in ability to taste or smell?	☐	☐	☐
29. Does the patient report a recent change in weight (not related to dieting)?	☐	☐	☐
30. Does the patient experience excessive sweating? (not related to hot weather)	☐	☐	☐
**SCORE:**			☐

TOTAL SCORE: ☐

Developed by the International Parkinson's Disease Non- Motor Group.
Contacts: ray.chaudhuri@uhl.nhs.uk or alison.forbes@uhl.nhs.uk

APPENDIX M. PARKINSON'S DISEASE SLEEP SCALE-2 (PDSS-2)

Parkinson's Disease Sleep Scale (PDSS-2)

Please rate the severity of the following based on your experiences during the past week (7 days). Please circle your answer.

Very often	(This means 6 to 7 days a week)
Often	(This means 4 to 5 days a week)
Sometimes	(This means 2 to 3 days a week)
Occasionally	(This means 1 day a week)
Never	

	Very often	Often	Sometimes	Occasionally	Never
1. Overall, did you sleep well during the last week?	0	1	2	3	4
2. Did you have difficulty falling asleep each night?	4	3	2	1	0
3. Did you have difficulty staying asleep?	4	3	2	1	0
4. Did you have restlessness of legs or arms at nights causing disruption of sleep?	4	3	2	1	0
5. Was your sleep disturbed due to an urge to move your legs or arms?	4	3	2	1	0
6. Did you suffer from distressing dreams at night?	4	3	2	1	0
7. Did you suffer from distressing hallucinations at night (seeing or hearing things that do not exist)?	4	3	2	1	0
8. Did you get up at night to urinate?	4	3	2	1	0
9. Did you feel uncomfortable at night because you were unable to turn over in bed or move due to immobility?	4	3	2	1	0
10. Did you feel pain in your arms or legs which woke you up while you were sleeping during the night?	4	3	2	1	0
11. Did you have muscle cramps in your arms or legs which woke you up while you were sleeping during the night?	4	3	2	1	0
12. Did you wake up earlier than usual with painful posturing of arms and legs?	4	3	2	1	0
13. On waking in the morning or during the night, did you experience tremor?	4	3	2	1	0
14. Did you feel tired and sleepy after waking up in the morning?	4	3	2	1	0
15. Did you wake up at night due to snoring or difficulties with breathing?	4	3	2	1	0

PDSS-2 © Ray Chaudhuri, Claudia Trenkwalder 2010, All rights reserved

PDSS-2 - United States/English - Version of 30 Jul 15 - Mapi.
[illegible]

Trenkwalder C, Kohnen R, Högl B, Metta V, Sixel-Döring F, Frauscher B, Hülsmann J, Martinez-Martin P, Chaudhuri KR. Parkinson's disease sleep scale--validation of the revised version PDSS-2. Mov Disord. 2011;26(4):644-52.

APPENDIX N. PARKINSON ANXIETY SCALE (PAS)

The Parkinson Anxiety Scale (PAS); English version

A. Persistent anxiety

Please mark one circle for each item below

In the past four weeks, to what extent did you experience the following symptoms?

A.1. Feeling anxious or nervous
- 0. Not at all, or never
- 1. Very mild, or rarely
- 2. Mild, or sometimes
- 3. Moderate, or often
- 4. Severe, or (nearly) always

A.2. Feeling tense or stressed
- 0. Not at all, or never
- 1. Very mild, or rarely
- 2. Mild, or sometimes
- 3. Moderate, or often
- 4. Severe, or (nearly) always

A.3. Being unable to relax
- 0. Not at all, or never
- 1. Very mild, or rarely
- 2. Mild, or sometimes
- 3. Moderate, or often
- 4. Severe, or (nearly) always

A.4. Excessive worrying about everyday matters
- 0. Not at all, or never
- 1. Very mild, or rarely
- 2. Mild, or sometimes
- 3. Moderate, or often
- 4. Severe, or (nearly) always

A.5. Fear of something bad, or even the worst, happening
- 0. Not at all, or never
- 1. Very mild, or rarely
- 2. Mild, or sometimes
- 3. Moderate, or often
- 4. Severe, or (nearly) always

Leentjens AFG, Dujardin D, Pontone GM, Starkstein SE, Weintraub D, Martinez-Martin P. The Parkinson Anxiety Scale (PAS): development and validation of a new anxiety scale Mov. Disord 2014;29:1035-43. doi: 10.1002/mds.25919.

B. Episodic anxiety

Please mark one circle for each item below

In the past four weeks, did you experience episodes of the following symptoms?

B.1. Panic or intense fear
0. Never
1. Rarely
2. Sometimes
3. Often
4. Nearly always

B.2. Shortness of breath
0. Never
1. Rarely
2. Sometimes
3. Often
4. Nearly always

B.3. Heart palpitations or heart beating fast (not related to physical effort or activity)
0. Never
1. Rarely
2. Sometimes
3. Often
4. Nearly always

B.4. Fear of losing control
0. Never
1. Rarely
2. Sometimes
3. Often
4. Nearly always

C. Avoidance behavior

Please mark one circle for each item below

In the past four weeks, to what extent did you fear or avoid the following situations?

C.1. Social situations (where one may be observed, or evaluated by others, such as speaking in public, or talking to unknown people)

0. Never
1. Rarely
2. Sometimes
3. Often
4. Nearly always

C.2. Public settings (situations from which it may be difficult or embarrassing to escape, such as queues or lines, crowds, bridges, or public transportation)

0. Never
1. Rarely
2. Sometimes
3. Often
4. Nearly always

C.3. Specific objects or situations (such as flying, heights, spiders or other animals, needles, or blood)

0. Never
1. Rarely
2. Sometimes
3. Often
4. Nearly always

APPENDIX O. COLUMBIA-SUICIDE SEVERITY RATING SCALE (C-SSRS)

COLUMBIA-SUICIDE SEVERITY RATING SCALE (C-SSRS)

Baseline/Screening Version

Version 1/14/09

Posner, K.; Brent, D.; Lucas, C.; Gould, M.; Stanley, B.; Brown, G.; Fisher, P.; Zelazny, J.; Burke, A.; Oquendo, M.; Mann, J.

Disclaimer:

This scale is intended to be used by individuals who have received training in its administration. The questions contained in the Columbia-Suicide Severity Rating Scale are suggested probes. Ultimately, the determination of the presence of suicidal ideation or behavior depends on the judgment of the individual administering the scale.

Definitions of behavioral suicidal events in this scale are based on those used in **The Columbia Suicide History Form**, *developed by John Mann, MD and Maria Oquendo, MD, Conte Center for the Neuroscience of Mental Disorders (CCNMD), New York State Psychiatric Institute, 1051 Riverside Drive, New York, NY, 10032. (Oquendo M. A., Halberstam B. & Mann J. J., Risk factors for suicidal behavior: utility and limitations of research instruments. In M.B. First [Ed.]* Standardized Evaluation in Clinical Practice, *pp. 103 -130, 2003.)*

For reprints of the C-SSRS contact Kelly Posner, Ph.D., New York State Psychiatric Institute, 1051 Riverside Drive, New York, New York, 10032; inquiries and training requirements contact posnerk@nyspi.columbia.edu

© 2008 The Research Foundation for Mental Hygiene, Inc.

SUICIDAL IDEATION		
Ask questions 1 and 2. If both are negative, proceed to "Suicidal Behavior" section. If the answer to question 2 is "yes", ask questions 3, 4 and 5. If the answer to question 1 and/or 2 is "yes", complete "Intensity of Ideation" section below.	**Lifetime: Time He/She Felt Most Suicidal**	**Past __ Months**
**1. Wish to be Dead**<br>Subject endorses thoughts about a wish to be dead or not alive anymore, or wish to fall asleep and not wake up.<br>***Have you wished you were dead or wished you could go to sleep and not wake up?***<br><br>If yes, describe:	**Yes** ☐ **No** ☐	**Yes** ☐ **No** ☐
**2. Non-Specific Active Suicidal Thoughts**<br>General non-specific thoughts of wanting to end one's life/commit suicide (e.g., *"I've thought about killing myself"*) without thoughts of ways to kill oneself/associated methods, intent, or plan during the assessment period.<br>***Have you actually had any thoughts of killing yourself?***<br><br>If yes, describe:	**Yes** ☐ **No** ☐	**Yes** ☐ **No** ☐
**3. Active Suicidal Ideation with Any Methods (Not Plan) without Intent to Act**<br>Subject endorses thoughts of suicide and has thought of at least one method during the assessment period. This is different than a specific plan with time, place or method details worked out (e.g. thought of method to kill self but not a specific plan). Includes person who would say, *"I thought about taking an overdose but I never made a specific plan as to when, where or how I would actually do it…and I would never go through with it."*<br>***Have you been thinking about how you might do this?***<br><br>If yes, describe:	**Yes** ☐ **No** ☐	**Yes** ☐ **No** ☐
**4. Active Suicidal Ideation with Some Intent to Act, without Specific Plan**<br>Active suicidal thoughts of killing oneself and subject reports having some intent to act on such thoughts, as opposed to *"I have the thoughts but I definitely will not do anything about them."*<br>***Have you had these thoughts and had some intention of acting on them?***<br><br>If yes, describe:	**Yes** ☐ **No** ☐	**Yes** ☐ **No** ☐
**5. Active Suicidal Ideation with Specific Plan and Intent**<br>Thoughts of killing oneself with details of plan fully or partially worked out and subject has some intent to carry it out.<br>***Have you started to work out or worked out the details of how to kill yourself? Do you intend to carry out this plan?***<br><br>If yes, describe:	**Yes** ☐ **No** ☐	**Yes** ☐ **No** ☐
INTENSITY OF IDEATION		
The following features should be rated with respect to the most severe type of ideation (i.e., 1-5 from above, with 1 being the least severe and 5 being the most severe). Ask about time he/she was feeling the most suicidal.<br><br>Lifetime - ***Most Severe Ideation:*** ______ *Type # (1-5)* ____________ *Description of Ideation*<br><br>Past X Months - ***Most Severe Ideation:*** ______ *Type # (1-5)* ____________ *Description of Ideation*	Most Severe	Most Severe
**Frequency**<br>***How many times have you had these thoughts?***<br>(1) Less than once a week (2) Once a week (3) 2-5 times in week (4) Daily or almost daily (5) Many times each day	___	___
**Duration**<br>***When you have the thoughts how long do they last?***<br>(1) Fleeting - few seconds or minutes<br>(2) Less than 1 hour/some of the time<br>(3) 1-4 hours/a lot of time<br>(4) 4-8 hours/most of day<br>(5) More than 8 hours/persistent or continuous	___	___
**Controllability**<br>***Could/can you stop thinking about killing yourself or wanting to die if you want to?***<br>(1) Easily able to control thoughts<br>(2) Can control thoughts with little difficulty<br>(3) Can control thoughts with some difficulty<br>(4) Can control thoughts with a lot of difficulty<br>(5) Unable to control thoughts<br>(0) Does not attempt to control thoughts	___	___
**Deterrents**<br>***Are there things - anyone or anything (e.g., family, religion, pain of death) - that stopped you from wanting to die or acting on thoughts of committing suicide?***<br>(1) Deterrents definitely stopped you from attempting suicide<br>(2) Deterrents probably stopped you<br>(3) Uncertain that deterrents stopped you<br>(4) Deterrents most likely did not stop you<br>(5) Deterrents definitely did not stop you<br>(0) Does not apply	___	___
**Reasons for Ideation**<br>***What sort of reasons did you have for thinking about wanting to die or killing yourself? Was it to end the pain or stop the way you were feeling (in other words you couldn't go on living with this pain or how you were feeling) or was it to get attention, revenge or a reaction from others? Or both?***<br>(1) Completely to get attention, revenge or a reaction from others<br>(2) Mostly to get attention, revenge or a reaction from others<br>(3) Equally to get attention, revenge or a reaction from others and to end/stop the pain<br>(4) Mostly to end or stop the pain (you couldn't go on living with the pain or how you were feeling)<br>(5) Completely to end or stop the pain (you couldn't go on living with the pain or how you were feeling)<br>(0) Does not apply	___	___

© 2008 Research Foundation for Mental Hygiene, Inc. C-SSRS—Baseline/Screening (Version 1/14/09) Page 1 of 2

SUICIDAL BEHAVIOR *(Check all that apply, so long as these are separate events; must ask about all types)*	Lifetime	Past __ Years
**Actual Attempt:**<br>A potentially self-injurious act committed with at least some wish to die, *as a result of act.* Behavior was in part thought of as method to kill oneself. Intent does not have to be 100%. If there is ***any*** intent/desire to die associated with the act, then it can be considered an actual suicide attempt. ***There does not have to be any injury or harm,*** just the potential for injury or harm. If person pulls trigger while gun is in mouth but gun is broken so no injury results, this is considered an attempt.<br>Inferring Intent: Even if an individual denies intent/wish to die, it may be inferred clinically from the behavior or circumstances. For example, a highly lethal act that is clearly not an accident so no other intent but suicide can be inferred (e.g., gunshot to head, jumping from window of a high floor/story). Also, if someone denies intent to die, but they thought that what they did could be lethal, intent may be inferred.<br>***Have you made a suicide attempt?***<br>***Have you done anything to harm yourself?***<br>***Have you done anything dangerous where you could have died?***<br>***What did you do?***<br>***Did you______ as a way to end your life?***<br>***Did you want to die (even a little) when you_____?***<br>***Were you trying to end your life when you _____?***<br>***Or Did you think it was possible you could have died from_____?***<br>***Or did you do it purely for other reasons / without ANY intention of killing yourself (like to relieve stress, feel better, get sympathy, or get something else to happen)?*** (Self-Injurious Behavior without suicidal intent)<br>If yes, describe:	Yes No<br>☐ ☐<br>Total # of Attempts<br>_____	Yes No<br>☐ ☐<br>Total # of Attempts<br>_____
**Has subject engaged in Non-Suicidal Self-Injurious Behavior?**	Yes No<br>☐ ☐	Yes No<br>☐ ☐
**Interrupted Attempt:**<br>When the person is interrupted (by an outside circumstance) from starting the potentially self-injurious act *(if not for that, actual attempt would have occurred).*<br>Overdose: Person has pills in hand but is stopped from ingesting. Once they ingest any pills, this becomes an attempt rather than an interrupted attempt. Shooting: Person has gun pointed toward self, gun is taken away by someone else, or is somehow prevented from pulling trigger. Once they pull the trigger, even if the gun fails to fire, it is an attempt. Jumping: Person is poised to jump, is grabbed and taken down from ledge. Hanging: Person has noose around neck but has not yet started to hang - is stopped from doing so.<br>***Has there been a time when you started to do something to end your life but someone or something stopped you before you actually did anything?***<br>If yes, describe:	Yes No<br>☐ ☐<br>Total # of interrupted<br>_____	Yes No<br>☐ ☐<br>Total # of interrupted<br>_____
**Aborted Attempt:**<br>When person begins to take steps toward making a suicide attempt, but stops themselves before they actually have engaged in any self-destructive behavior. Examples are similar to interrupted attempts, except that the individual stops him/herself, instead of being stopped by something else.<br>***Has there been a time when you started to do something to try to end your life but you stopped yourself before you actually did anything?***<br>If yes, describe:	Yes No<br>☐ ☐<br>Total # of aborted<br>_____	Yes No<br>☐ ☐<br>Total # of aborted<br>_____
**Preparatory Acts or Behavior:**<br>Acts or preparation towards imminently making a suicide attempt. This can include anything beyond a verbalization or thought, such as assembling a specific method (e.g., buying pills, purchasing a gun) or preparing for one's death by suicide (e.g., giving things away, writing a suicide note).<br>***Have you taken any steps towards making a suicide attempt or preparing to kill yourself (such as collecting pills, getting a gun, giving valuables away or writing a suicide note)?***<br>If yes, describe:	Yes No<br>☐ ☐	Yes No<br>☐ ☐
**Suicidal Behavior:**<br>Suicidal behavior was present during the assessment period?	Yes No<br>☐ ☐	Yes No<br>☐ ☐

Answer for Actual Attempts Only	Most Recent Attempt Date:	Most Lethal Attempt Date:	Initial/First Attempt Date:
**Actual Lethality/Medical Damage:**<br>0. No physical damage or very minor physical damage (e.g., surface scratches).<br>1. Minor physical damage (e.g., lethargic speech; first-degree burns; mild bleeding; sprains).<br>2. Moderate physical damage; medical attention needed (e.g., conscious but sleepy, somewhat responsive; second-degree burns; bleeding of major vessel).<br>3. Moderately severe physical damage; *medical* hospitalization and likely intensive care required (e.g., comatose with reflexes intact; third-degree burns less than 20% of body; extensive blood loss but can recover; major fractures).<br>4. Severe physical damage; *medical* hospitalization with intensive care required (e.g., comatose without reflexes; third-degree burns over 20% of body; extensive blood loss with unstable vital signs; major damage to a vital area).<br>5. Death	*Enter Code*<br>_____	*Enter Code*<br>_____	*Enter Code*<br>_____
**Potential Lethality: Only Answer if Actual Lethality=0**<br>Likely lethality of actual attempt if no medical damage (the following examples, while having no actual medical damage, had potential for very serious lethality: put gun in mouth and pulled the trigger but gun fails to fire so no medical damage; laying on train tracks with oncoming train but pulled away before run over).<br>0 = Behavior not likely to result in injury<br>1 = Behavior likely to result in injury but not likely to cause death<br>2 = Behavior likely to result in death despite available medical care	*Enter Code*<br>_____	*Enter Code*<br>_____	*Enter Code*<br>_____

© 2008 Research Foundation for Mental Hygiene, Inc. C-SSRS—Baseline/Screening (Version 1/14/09) Page 2 of 2

COLUMBIA-SUICIDE SEVERITY RATING SCALE (C-SSRS)

Since Last Visit

Version 1/14/09

Posner, K.; Brent, D.; Lucas, C.; Gould, M.; Stanley, B.; Brown, G.; Fisher, P.; Zelazny, J.; Burke, A.; Oquendo, M.; Mann, J.

Disclaimer:

This scale is intended to be used by individuals who have received training in its administration. The questions contained in the Columbia-Suicide Severity Rating Scale are suggested probes. Ultimately, the determination of the presence of suicidal ideation or behavior depends on the judgment of the individual administering the scale.

Definitions of behavioral suicidal events in this scale are based on those used in **The Columbia Suicide History Form**, *developed by John Mann, MD and Maria Oquendo, MD, Conte Center for the Neuroscience of Mental Disorders (CCNMD), New York State Psychiatric Institute, 1051 Riverside Drive, New York, NY, 10032. (Oquendo M. A., Halberstam B. & Mann J. J., Risk factors for suicidal behavior: utility and limitations of research instruments. In M.B. First [Ed.]* Standardized Evaluation in Clinical Practice, *pp. 103 -130, 2003.)*

For reprints of the C-SSRS contact Kelly Posner, Ph.D., New York State Psychiatric Institute, 1051 Riverside Drive, New York, New York, 10032; inquiries and training requirements contact posnerk@nyspi.columbia.edu

© 2008 The Research Foundation for Mental Hygiene, Inc.

SUICIDAL IDEATION

Ask questions 1 and 2. If both are negative, proceed to "Suicidal Behavior" section. If the answer to question 2 is "yes", ask questions 3, 4 and 5. If the answer to question 1 and/or 2 is "yes", complete "Intensity of Ideation" section below.	**Since Last Visit**
**1. Wish to be Dead**<br>Subject endorses thoughts about a wish to be dead or not alive anymore, or wish to fall asleep and not wake up.<br>***Have you wished you were dead or wished you could go to sleep and not wake up?***<br><br>If yes, describe:	**Yes** **No**<br>☐ ☐
**2. Non-Specific Active Suicidal Thoughts**<br>General, non-specific thoughts of wanting to end one's life/commit suicide (e.g., *"I've thought about killing myself"*) without thoughts of ways to kill oneself/associated methods, intent, or plan during the assessment period.<br>***Have you actually had any thoughts of killing yourself?***<br><br>If yes, describe:	**Yes** **No**<br>☐ ☐
**3. Active Suicidal Ideation with Any Methods (Not Plan) without Intent to Act**<br>Subject endorses thoughts of suicide and has thought of at least one method during the assessment period. This is different than a specific plan with time, place or method details worked out (e.g., thought of method to kill self but not a specific plan). Includes person who would say, *"I thought about taking an overdose but I never made a specific plan as to when, where or how I would actually do it…and I would never go through with it."*<br>***Have you been thinking about how you might do this?***<br><br>If yes, describe:	**Yes** **No**<br>☐ ☐
**4. Active Suicidal Ideation with Some Intent to Act, without Specific Plan**<br>Active suicidal thoughts of killing oneself and subject reports having some intent to act on such thoughts, as opposed to *"I have the thoughts but I definitely will not do anything about them."*<br>***Have you had these thoughts and had some intention of acting on them?***<br><br>If yes, describe:	**Yes** **No**<br>☐ ☐
**5. Active Suicidal Ideation with Specific Plan and Intent**<br>Thoughts of killing oneself with details of plan fully or partially worked out and subject has some intent to carry it out.<br>***Have you started to work out or worked out the details of how to kill yourself? Do you intend to carry out this plan?***<br><br>If yes, describe:	**Yes** **No**<br>☐ ☐

INTENSITY OF IDEATION

The following features should be rated with respect to the most severe type of ideation (i.e., 1-5 from above, with 1 being the least severe and 5 being the most severe).<br><br>***Most Severe Ideation:*** _____ ____________________________<br>*Type # (1-5)* *Description of Ideation*	Most Severe
**Frequency**<br>***How many times have you had these thoughts?***<br>(1) Less than once a week (2) Once a week (3) 2-5 times in week (4) Daily or almost daily (5) Many times each day	____
**Duration**<br>***When you have the thoughts, how long do they last?***<br>(1) Fleeting - few seconds or minutes<br>(2) Less than 1 hour/some of the time<br>(3) 1-4 hours/a lot of time<br>(4) 4-8 hours/most of day<br>(5) More than 8 hours/persistent or continuous	____
**Controllability**<br>***Could/can you stop thinking about killing yourself or wanting to die if you want to?***<br>(1) Easily able to control thoughts<br>(2) Can control thoughts with little difficulty<br>(3) Can control thoughts with some difficulty<br>(4) Can control thoughts with a lot of difficulty<br>(5) Unable to control thoughts<br>(0) Does not attempt to control thoughts	____
**Deterrents**<br>***Are there things - anyone or anything (e.g., family, religion, pain of death) - that stopped you from wanting to die or acting on thoughts of committing suicide?***<br>(1) Deterrents definitely stopped you from attempting suicide<br>(2) Deterrents probably stopped you<br>(3) Uncertain that deterrents stopped you<br>(4) Deterrents most likely did not stop you<br>(5) Deterrents definitely did not stop you<br>(0) Does not apply	____
**Reasons for Ideation**<br>***What sort of reasons did you have for thinking about wanting to die or killing yourself? Was it to end the pain or stop the way you were feeling (in other words you couldn't go on living with this pain or how you were feeling) or was it to get attention, revenge or a reaction from others? Or both?***<br>(1) Completely to get attention, revenge or a reaction from others<br>(2) Mostly to get attention, revenge or a reaction from others<br>(3) Equally to get attention, revenge or a reaction from others and to end/stop the pain<br>(4) Mostly to end or stop the pain (you couldn't go on living with the pain or how you were feeling)<br>(5) Completely to end or stop the pain (you couldn't go on living with the pain or how you were feeling)<br>(0) Does not apply	____

© 2008 Research Foundation for Mental Hygiene, Inc. C-SSRS—Since Last Visit (Version 1/14/09) Page 1 of 2

SUICIDAL BEHAVIOR *(Check all that apply, so long as these are separate events; must ask about all types)*	**Since Last Visit**
**Actual Attempt:** A potentially self-injurious act committed with at least some wish to die, *as a result of act.* Behavior was in part thought of as method to kill oneself. Intent does not have to be 100%. If there is ***any*** intent/desire to die associated with the act, then it can be considered an actual suicide attempt. ***There does not have to be any injury or harm,*** just the potential for injury or harm. If person pulls trigger while gun is in mouth but gun is broken so no injury results, this is considered an attempt.<br>Inferring Intent: Even if an individual denies intent/wish to die, it may be inferred clinically from the behavior or circumstances. For example, a highly lethal act that is clearly not an accident so no other intent but suicide can be inferred (e.g., gunshot to head, jumping from window of a high floor/story). Also, if someone denies intent to die, but they thought that what they did could be lethal, intent may be inferred.<br>***Have you made a suicide attempt?***<br>***Have you done anything to harm yourself?***<br>***Have you done anything dangerous where you could have died?***<br>***What did you do?***<br>***Did you______ as a way to end your life?***<br>***Did you want to die (even a little) when you_____?***<br>***Were you trying to end your life when you _____?***<br>***Or did you think it was possible you could have died from_____?***<br>***Or did you do it purely for other reasons / without ANY intention of killing yourself (like to relieve stress, feel better, get sympathy, or get something else to happen)?*** (Self-Injurious Behavior without suicidal intent)<br>If yes, describe:	**Yes No**<br>☐ ☐<br>Total # of Attempts<br>_____
**Has subject engaged in Non-Suicidal Self-Injurious Behavior?**	**Yes No**<br>☐ ☐
**Interrupted Attempt:** When the person is interrupted (by an outside circumstance) from starting the potentially self-injurious act *(if not for that, actual attempt would have occurred).*<br>Overdose: Person has pills in hand but is stopped from ingesting. Once they ingest any pills, this becomes an attempt rather than an interrupted attempt. Shooting: Person has gun pointed toward self, gun is taken away by someone else, or is somehow prevented from pulling trigger. Once they pull the trigger, even if the gun fails to fire, it is an attempt. Jumping: Person is poised to jump, is grabbed and taken down from ledge. Hanging: Person has noose around neck but has not yet started to hang - is stopped from doing so.<br>***Has there been a time when you started to do something to end your life but someone or something stopped you before you actually did anything?***<br>If yes, describe:	**Yes No**<br>☐ ☐<br>Total # of interrupted<br>_____
**Aborted Attempt:** When person begins to take steps toward making a suicide attempt, but stops themselves before they actually have engaged in any self-destructive behavior. Examples are similar to interrupted attempts, except that the individual stops him/herself, instead of being stopped by something else.<br>***Has there been a time when you started to do something to try to end your life but you stopped yourself before you actually did anything?***<br>If yes, describe:	**Yes No**<br>☐ ☐<br>Total # of aborted<br>_____
**Preparatory Acts or Behavior:** Acts or preparation towards imminently making a suicide attempt. This can include anything beyond a verbalization or thought, such as assembling a specific method (e.g., buying pills, purchasing a gun) or preparing for one's death by suicide (e.g., giving things away, writing a suicide note).<br>***Have you taken any steps towards making a suicide attempt or preparing to kill yourself (such as collecting pills, getting a gun, giving valuables away or writing a suicide note)?***<br>If yes, describe:	**Yes No**<br>☐ ☐
**Suicidal Behavior:** Suicidal behavior was present during the assessment period?	**Yes No**<br>☐ ☐
**Suicide:**	**Yes No**<br>☐ ☐
Answer for Actual Attempts Only	Most Lethal Attempt Date:
**Actual Lethality/Medical Damage:**<br>0. No physical damage or very minor physical damage (e.g., surface scratches).<br>1. Minor physical damage (e.g., lethargic speech; first-degree burns; mild bleeding; sprains).<br>2. Moderate physical damage; medical attention needed (e.g., conscious but sleepy, somewhat responsive; second-degree burns; bleeding of major vessel).<br>3. Moderately severe physical damage; *medical* hospitalization and likely intensive care required (e.g., comatose with reflexes intact; third-degree burns less than 20% of body; extensive blood loss but can recover; major fractures).<br>4. Severe physical damage; *medical* hospitalization with intensive care required (e.g., comatose without reflexes; third-degree burns over 20% of body; extensive blood loss with unstable vital signs; major damage to a vital area).<br>5. Death	*Enter Code*<br>_____
**Potential Lethality: Only Answer if Actual Lethality=0** Likely lethality of actual attempt if no medical damage (the following examples, while having no actual medical damage, had potential for very serious lethality: put gun in mouth and pulled the trigger but gun fails to fire so no medical damage; laying on train tracks with oncoming train but pulled away before run over).<br>0 = Behavior not likely to result in injury<br>1 = Behavior likely to result in injury but not likely to cause death<br>2 = Behavior likely to result in death despite available medical care	*Enter Code*<br>_____

© 2008 Research Foundation for Mental Hygiene, Inc. C-SSRS—Since Last Visit (Version 1/14/09) Page 2 of 2

APPENDIX P. PARKINSON'S DISEASE DIARY

PARKINSON'S DISEASE DIARY, IPX203-B16-02

INSTRUCTIONS FOR COMPLETING PARKINSON'S DISEASE DIARY

For each half-hour time period place one check mark to indicate your predominant states during most of that period.

ON = Time when medication is providing benefit with regard to mobility, slowness, and stiffness.

OFF = Time when medication has worn off and is no longer providing benefit with regard to mobility, slowness, and stiffness.

Dyskinesia = Involuntary twisting, turning movements. These movements are an effect of medication and occur during ON time.

Non-troublesome dyskinesia does not interfere with function or cause meaningful discomfort.

Troublesome dyskinesia interferes with function or causes meaningful discomfort.

Tremor is shaking back and forth and is not considered dyskinesia.

Table of Contents

Parkinson's Disease Diary for First 24-hour Period....Page 2
Parkinson's Disease Diary for Second 24-hour Period....Page 3
Parkinson's Disease Diary for Third 24-hour Period....Page 4

EXAMPLE OF DIARY COMPLETION

Time	Asleep	OFF	ON without dyskinesia	ON with non-troublesome dyskinesia	ON with troublesome dyskinesia	
6:00 AM	√					Correct
:30	√	√				Incorrect
7:00 AM		√				Correct
:30		√				Correct
8:00 AM						Incorrect
:30		√			√	Incorrect
9:00 AM			√			Correct

Hauser et al. J Clin Neurophar 2000; 23:75-81
Impax_IPX203-B16-02_PD Diary_English(US)_Version 1.2_14Sep2018
CONFIDENTIAL
Page 1 of 4

Start Date DD-MMM-YYYY: ____________________________________

Instructions: For each half-hour time period place one check mark to indicate your predominant status during most of that period.
ON = Time when medication is providing benefit with regard to mobility, slowness, and stiffness.
OFF = Time when medication is not providing benefit with regard to mobility, slowness, and stiffness
Dyskinesia = Involuntary twisting, turning movements. These movements are an effect of medication and occur during ON time.
Non-troublesome dyskinesia does not interfere with function or cause meaningful discomfort.
Troublesome dyskinesia interferes with function or causes meaningful discomfort.
Tremor is shaking back and forth and is not considered dyskinesia.

time	asleep	OFF	ON without dyskinesia	ON with non-troublesome dyskinesia	ON with troublesome dyskinesia
6:00 AM					
:30					
7:00 AM					
:30					
8:00 AM					
:30					
9:00 AM					
:30					
10:00 AM					
:30					
11:00 AM					
:30					
12:00 PM					
:30					
1:00 PM					
:30					
2:00 PM					
:30					
3:00 PM					
:30					
4:00 PM					
:30					
5:00 PM					
:30					

time	asleep	OFF	ON without dyskinesia	ON with non-troublesome dyskinesia	ON with troublesome dyskinesia
6:00 PM					
:30					
7:00 PM					
:30					
8:00 PM					
:30					
9:00 PM					
:30					
10:00 PM					
:30					
11:00 PM					
:30					
12:00 AM					
:30					
1:00 AM					
:30					
2:00 AM					
:30					
3:00 AM					
:30					
4:00 AM					
:30					
5:00 AM					
:30					

Copyright © The University of South Florida, 2000. All rights reserved

Hauser et al. J Clin Neurophar 2000; 23:75-81

Start Date DD-MMM-YYYY: ______________________________________

Instructions: For each half-hour time period place one check mark to indicate your predominant status during most of that period.
ON = Time when medication is providing benefit with regard to mobility, slowness, and stiffness.
OFF = Time when medication is not providing benefit with regard to mobility, slowness, and stiffness
Dyskinesia = Involuntary twisting, turning movements. These movements are an effect of medication and occur during ON time.
Non-troublesome dyskinesia does not interfere with function or cause meaningful discomfort.
Troublesome dyskinesia interferes with function or causes meaningful discomfort.
Tremor is shaking back and forth and is not considered dyskinesia.

time	asleep	OFF	ON without dyskinesia	ON with non-troublesome dyskinesia	ON with troublesome dyskinesia
6:00 AM					
:30					
7:00 AM					
:30					
8:00 AM					
:30					
9:00 AM					
:30					
10:00 AM					
:30					
11:00 AM					
:30					
12:00 PM					
:30					
1:00 PM					
:30					
2:00 PM					
:30					
3:00 PM					
:30					
4:00 PM					
:30					
5:00 PM					
:30					

time	asleep	OFF	ON without dyskinesia	ON with non-troublesome dyskinesia	ON with troublesome dyskinesia
6:00 PM					
:30					
7:00 PM					
:30					
8:00 PM					
:30					
9:00 PM					
:30					
10:00 PM					
:30					
11:00 PM					
:30					
12:00 AM					
:30					
1:00 AM					
:30					
2:00 AM					
:30					
3:00 AM					
:30					
4:00 AM					
:30					
5:00 AM					
:30					

Copyright © The University of South Florida, 2000. All rights reserved

Hauser et al. J Clin Neurophar 2000; 23:75-81

Start Date DD-MMM-YYYY: ____

Instructions: For each half-hour time period place <u>one</u> check mark to indicate your predominant status during most of that period.
ON = Time when medication is providing benefit with regard to mobility, slowness, and stiffness.
OFF = Time when medication is not providing benefit with regard to mobility, slowness, and stiffness
Dyskinesia = Involuntary twisting, turning movements. These movements are an effect of medication and occur during ON time.
Non-troublesome dyskinesia does <u>not</u> interfere with function or cause meaningful discomfort.
Troublesome dyskinesia interferes with function or causes meaningful discomfort.
Tremor is shaking back and forth and is not considered dyskinesia.

time	asleep	OFF	ON without dyskinesia	ON with non-troublesome dyskinesia	ON with troublesome dyskinesia
6:00 AM					
:30					
7:00 AM					
:30					
8:00 AM					
:30					
9:00 AM					
:30					
10:00 AM					
:30					
11:00 AM					
:30					
12:00 PM					
:30					
1:00 PM					
:30					
2:00 PM					
:30					
3:00 PM					
:30					
4:00 PM					
:30					
5:00 PM					
:30					

time	asleep	OFF	ON without dyskinesia	ON with non-troublesome dyskinesia	ON with troublesome dyskinesia
6:00 PM					
:30					
7:00 PM					
:30					
8:00 PM					
:30					
9:00 PM					
:30					
10:00 PM					
:30					
11:00 PM					
:30					
12:00 AM					
:30					
1:00 AM					
:30					
2:00 AM					
:30					
3:00 AM					
:30					
4:00 AM					
:30					
5:00 AM					
:30					

Copyright © The University of South Florida, 2000. All rights reserved

Hauser et al. J Clin Neurophar 2000; 23:75-81

APPENDIX Q. EXCIPIENTS IN IPX203, IPX203 PLACEBO, IR CD-LD, AND IR PLACEBO

**IPX203 (Various Strengths)**	**IPX203 Placebo**	**IR CD-LD (25-100 mg)**	**IR Placebo**
Microcrystalline Cellulose, NF	Microcrystalline Cellulose, NF	Crospovidone	Microcrystalline cellulose, NF
Croscarmellose Sodium, NF	Talc, USP	Hydroxypropyl Cellulose	Magnesium Stearate, NF
Magnesium Stearate, NF	Magnesium Stearate, NF	Magnesium Stearate	Quinoline yellow E104
Mannitol, USP	Sugar Spheres, NF	Microcrystalline cellulose	
Sodium Lauryl Sulfate, NF	Methacrylic acid copolymer Type A, NF	Starch (corn)	
Povidone, USP	Triethyl citrate, NF	D&C Yellow No. 10	
Cellulose Acetate	Hard gelatin capsules	Aluminum Oxide	
Copovidone, NF			
Amino Methacrylate Copolymer, NF			
Methacrylic acid copolymer Type A, NF			
Triethyl Citrate, NF			
Talc, USP			
Hard gelatin capsules			

APPENDIX R. CLINICAL LABORATORY STUDIES

HEMATOLOGY

hemoglobin	% lymphocytes	absolute lymphocytes
hematocrit	% monocytes	absolute monocytes
red blood cell count	% basophils	absolute basophils
white blood cell count	% eosinophils	absolute eosinophils
% neutrophils	absolute neutrophils	platelet count

CHEMISTRY

sodium	calcium	indirect bilirubin
potassium	phosphorous	alkaline phosphatase
chloride	albumin	alanine aminotransferase (ALT, SGPT)
carbon dioxide	total protein	aspartate aminotransferase (AST, SGOT)
blood urea nitrogen (BUN)	uric acid	creatine phosphokinase
creatinine	total bilirubin	lactate dehydrogenase
glucose	direct bilirubin	

URINALYSIS

pH	ketones	leukocyte esterase
specific gravity	microscopic exam (RBC and WBC, only when indicated)	protein
blood		
glucose		

URINE DRUG TEST

amphetamines	benzodiazepines
barbiturates	
cannabinoids	
cocaine metabolites	
opiates	
phencyclidines	

ALCOHOL BREATH TEST

PREGNANCY TEST
Urine pregnancy test (to be completed on site) for female subjects of childbearing potential.
