# Supplementary material for: IPX203 vs Immediate-Release Carbidopa-Levodopa for the Treatment of Motor Fluctuations in Parkinson Disease: The RISE-PD Randomized Clinical Trial
Source: JAMA Neurol. 2023 Aug 14;80(10):1062–9. doi: 10.1001/jamaneurol.2023.2679 (PMC10425876; doi:10.1001/jamaneurol.2023.2679)
Supplement: Supplement 2. — eTable 1. Demographics and Study Entry Characteristics eTable 2. Data Summary of Efficacy Endpoints eTable 3. Levodopa Dosages and Dosing Frequencies in the Double-Blind Period, Randomized Analysis Set eFigure. Change from Baseline in “Off” Time, mITT Analysis Set [file jamaneurol-e232679-s002.pdf]

Supplemental Online Content

Hauser RA, Espay AJ, Ellenbogen AL, et al. IPX203 vs immediate-release carbidopa-levodopa for the treatment of motor fluctuations in Parkinson disease: the RISE-PD randomized clinical trial. *JAMA Neurol*. Published online August 14, 2023. doi:10.1001/jamaneurol.2023.2679

eTable 1. Demographics and Study Entry Characteristics

eTable 2. Data Summary of Efficacy Endpoints

eTable 3. Levodopa Dosages and Dosing Frequencies in the Double-Blind Period, Randomized Analysis Set

eFigure. Change from Baseline in “Off” Time, mITT Analysis Set

This supplemental material has been provided by the authors to give readers additional information about their work.

© 2023 Hauser RA et al. *JAMA Neurology.*

eTable 1. Demographics and Study Entry Characteristics

Demographic/characteristic	Total (n=630)	Not randomized (n=124)	IPX203 (n=256)	IR CD-LD (n=250)
Age (y), mean (SD)	66.5 (8.95)	67.3 (9)	66.1 (9.02)	66.5 (8.85)
Gender, males, n (%)	396 (62.9)	60 (48.4%)	159 (62.1%)	177 (70.8%)
Gender, females, n (%)	234 (37.1)	64 (51.6)	97 (37.9)	73 (29.2)
Age of onset of PD (y), mean (SD)	58 (9.42)	58 (9.72)	57.7 (9.13)	58.3 (9.60)
Hoehn and Yahr 2/3/4, %	90.5	90.3	89.4	91.6
Screening MoCA score, mean (SD)	27.1 (1.81)	26.8 (1.87)	27.3 (1.80)	27.2 (1.78)
MDS-UPDRS Part III (On), mean (SD)	24.7 (15.66)	24.1 (15.34)	24.7 (15.96)	24.9 (15.56)
MDS-UPDRS Part III (Off), mean (SD)	45.2 (14.33)	43.4 (15.47)	45.5 (13.96)	45.6 (14.11)
"Off" Time (h), mean (SD)	6.13 (2.21)	6.24 (2.36)	6.12 (2.16)	6.08 (2.18)
"Good On" time (h), mean (SD)	9.56 (2.27)	9.55 (2.46)	9.52 (2.21)	9.60 (2.34)
Total daily LD, mean (SD)	797.8 (335.30)	836.9 (338.93)	792.3 (329.10)	784.1 (339.64)
Total daily IR LD, mean (SD)	754.7 (322.87)	791.7 (321.01)	746 (320.82)	745.2 (325.84)

Abbreviations: PD, Parkinson's Disease; MoCA, MoCA, Montreal Cognitive Assessment; MDS-UPDRS, Movement Disorders Society-Unified Parkinson's Disease Rating Scale; LD, levodopa; IR CD-LD, immediate-release carbidopa-levodopa.

© 2023 Hauser RA et al. *JAMA Neurology.*

eTable 2: Data Summary of Efficacy Endpoints

Endpoints	IPX203 (N=249)	IR CD-LD (N=246)	Difference (IPX203 vs. IR CD-LD)	p value
**Primary endpoint**				
Change from baseline in “Good on” time, LS mean	-0.5	-1.03	0.53	0.02[a]
**Key secondary endpoints**				
Change from baseline in “Off” time, LS mean	0.38	0.86	-0.48	0.03[a]
Patients self-rated “Much Improved” or “Very Much Improved” on PGI-C, %[c]	29.7	18.8	10.9	0.002[b]
Change from baseline in MDS-UPDRS Part III score, LS mean[c]	0.8	0.8	0	0.96[a]
Change from baseline in sum of MDS-UPDRS Part II and III scores, LS mean[c]	1.7	1.8	0	0.97[a]
**Post-hoc analysis**				
“Good on” time (h) per dose, LS mean	3.76	2.21	1.55	<0.0001[a]

Abbreviations: IR CD-LD, immediate-release carbidopa-levodopa; LS, least square; MDS-UPDRS, Movement Disorders Society-Unified Parkinson’s Disease Rating Scale; PGI-C, Patient Global Impression of Change.

[a] p value for the hypothesis of equal LS means.

[b] p value from the Cochran-Mantel-Haenszel test stratified by pooled center comparing the proportion of much or very much improved patients between the treatment groups.

[c] IPX203, N=256; IR CD-LD, N=250.

© 2023 Hauser RA et al. *JAMA Neurology.*

eTable 3: Levodopa Dosages and Dosing Frequencies in the Double-Blind Period, Randomized Analysis Set

Characteristic Statistic	IR CD-LD (N=250)	IPX203 (N=256)
**Total daily dose of LD (mg)**		
N	250	256
Mean (SD)	856.94 (378.68)	1488.05 (592.78)
Median (min, max)	800 (400, 2100)	1400 (420, 3360)
**Daily dosing frequency (times/day)**		
N	250	256
Mean (SD)	5.01 (1.19)	3.04 (0.40)
Median (min, max)	5 (3, 10)	3 (2, 4)

Abbreviations: IR CD-LD, immediate-release carbidopa-levodopa; LD, levodopa.

© 2023 Hauser RA et al. *JAMA Neurology.*

eFigure 1: Change from Baseline in "Off" Time, mITT Analysis Set

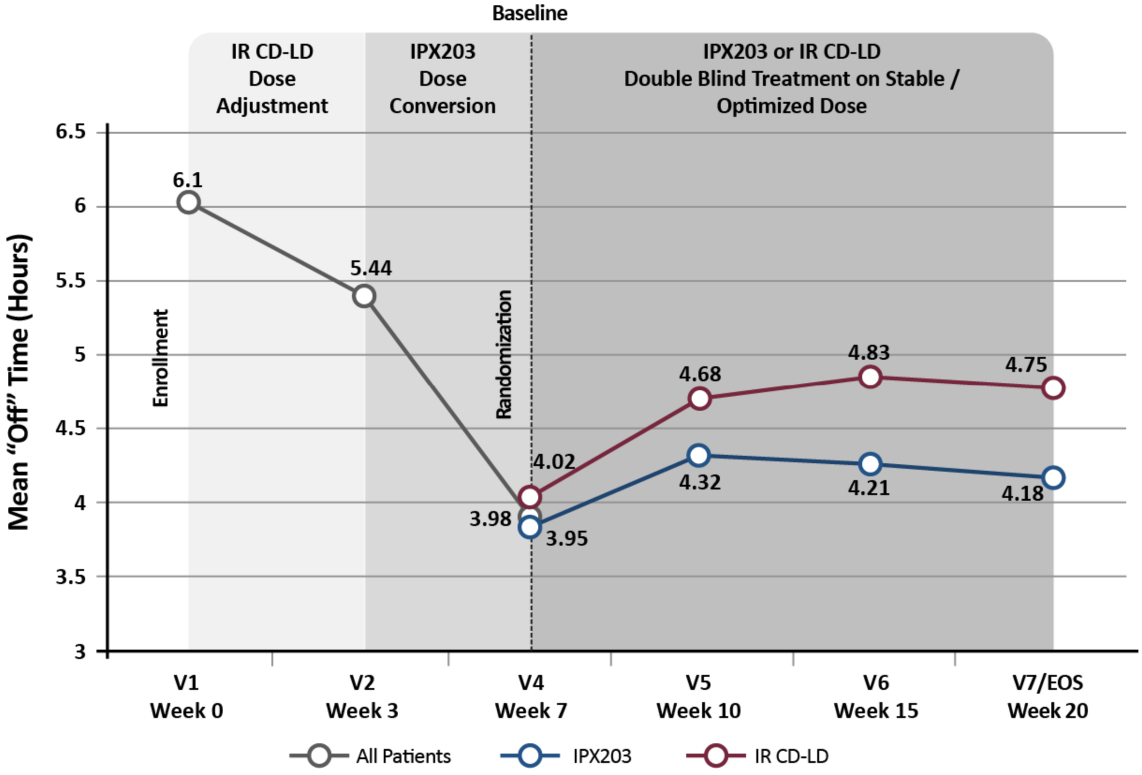

Abbreviations: EOS, end of study; IR CD-LD, immediate-release carbidopa-levodopa; mITT, modified intent-to-treat; V, Visit.

© 2023 Hauser RA et al. *JAMA Neurology.*
