# Supplementary material for: IPX203 vs Immediate-Release Carbidopa-Levodopa for the Treatment of Motor Fluctuations in Parkinson Disease: The RISE-PD Randomized Clinical Trial
Source: JAMA Neurol. 2023 Aug 14;80(10):1062–9. doi: 10.1001/jamaneurol.2023.2679 (PMC10425876; doi:10.1001/jamaneurol.2023.2679)
Supplement: Supplement 3. — Data sharing statement [file jamaneurol-e232679-s003.pdf]

Data Sharing Statement

Hauser. IPX203 vs Immediate-Release Carbidopa-Levodopa for the Treatment of Motor Fluctuations in Parkinson Disease. *JAMA Neurol*. Published August 14, 2023. doi:10.1001/jamaneurol.2023.2679

Data
Data available: Yes
Data types: Deidentified participant data
How to access data: Hester Visser <Hester.Visser@amneal.com>
When available: With publication

Supporting Documents
Document types: None

Additional Information
Who can access the data: researchers whose proposed use of the data has been approved
Types of analyses: for a specified purpose
Mechanisms of data availability: after request in writing to sponsor and after approval by sponsor
